# *Cauliflower mosaic virus* Transcriptome Reveals a Complex Alternative Splicing Pattern

**DOI:** 10.1371/journal.pone.0132665

**Published:** 2015-07-10

**Authors:** Clément Bouton, Angèle Geldreich, Laëtitia Ramel, Lyubov A. Ryabova, Maria Dimitrova, Mario Keller

**Affiliations:** Institut de Biologie Moléculaire des Plantes du CNRS, Université de Strasbourg, Strasbourg, France; University of Basel, SWITZERLAND

## Abstract

The plant pararetrovirus *Cauliflower mosaic virus* (CaMV) uses alternative splic-ing to generate several isoforms from its polycistronic pregenomic 35S RNA. This pro-cess has been shown to be essential for infectivity. Previous works have identified four splice donor sites and a single splice acceptor site in the 35S RNA 5’ region and sug-gested that the main role of CaMV splicing is to downregulate expression of open read-ing frames (ORFs) I and II. In this study, we show that alternative splicing is a conserved process among CaMV isolates. In Cabb B-JI and Cabb-S isolates, splicing frequently leads to different fusion between ORFs, particularly between ORF I and II. The corresponding P1P2 fusion proteins expressed in *E*. *coli* interact with viral proteins P2 and P3 *in vitro*. However, they are detected neither during infection nor upon transient expression *in planta*, which suggests rapid degradation after synthesis and no important biological role in the CaMV infectious cycle. To gain a better understanding of the functional relevance of 35S RNA alternative splicing in CaMV infectivity, we inactivated the previously described splice sites. All the splicing mutants were as pathogenic as the corresponding wild-type isolate. Through RT-PCR-based analysis we demonstrate that CaMV 35S RNA exhibits a complex splicing pattern, as we identify new splice donor and acceptor sites whose selection leads to more than thirteen 35S RNA isoforms in infected turnip plants. Inactivating splice donor or acceptor sites is not lethal for the virus, since disrupted sites are systematically rescued by the activation of cryptic and/or seldom used splice sites. Taken together, our data depict a conserved, complex and flexible process, involving multiple sites, that ensures splicing of 35S RNA.

## Introduction

Alternative RNA splicing is intensively performed by eukaryotes to increase the proteome diversity through the formation of numerous mRNA isoforms from a primary transcript and to regulate the expression of proteins in different organs and cell types [1–3]. Splicing can also enhance the expression level of protein coding genes because the spliceosome, the multi-subunit ribonucleoprotein complex catalyzing splicing, is tightly connected to the transcriptional machinery [4] and promotes mRNA export in mammalian cells [5]. Many animal DNA viruses, complex retroviruses [6] and type A *influenza virus* use this strategy to express their proteins during the infectious cycle [7]. In the animal pararetroviruses *Hepatitis B* virus (HBV) and *Duck hepatitis virus* (DHBV), some alternative splicing events lead to fusion between viral open reading frames (ORFs) but the roles of the corresponding fusion proteins remain elusive. However, it has been noticed that splicing is enhanced during chronic infection, notably prior to development of hepatocarcinoma, suggesting that spliced RNAs may contribute to the pathogenesis of *Hepadnaviruses* [8,9]. Splicing rarely occurs with plant viruses, essentially because the vast majority have an RNA genome and a strictly cytoplasmic replication cycle, except for the *Nucleorhabdovirus* genus members. However, several plant DNA viruses from the *Geminiviridae* and *Caulimoviridae* families perform splicing to express some of their proteins [10,11]. The introns found in their genomes perfectly follow the GU…AG rule defined for nuclear mRNA introns, and they possess AU-rich sequences as described for plant introns [12]. In *Mastreviruses* (family *Geminiviridae*), splicing of the C transcript generates an mRNA that codes for the fusion protein Rep which is required to initiate DNA replication by rolling circle mechanism [10]. In the *Caulimoviridae* family, whose members are all pararetroviruses, splicing has only been described so far in two genera—*Tungrovirus* and *Caulimovirus*—and it has been proposed to enhance the expression of distal ORFs in the spliced RNAs. In *Rice tungro bacilliform virus* (RTBV), the polycistronic 35S RNA undergoes a single splicing event that leads to removal of a large intron (6.3 kb), resulting in a monocistronic mRNA specific for protein P4 [13]. In *Figwort mosaic virus* (FMV), splicing of the polycistronic 35S RNA creates an in-frame fusion between the 5’ end of ORF IV and the 3’ region of ORF V [14]. However, the biological relevance of splicing in RTBV and FMV has never been investigated.

**Fig 1:**
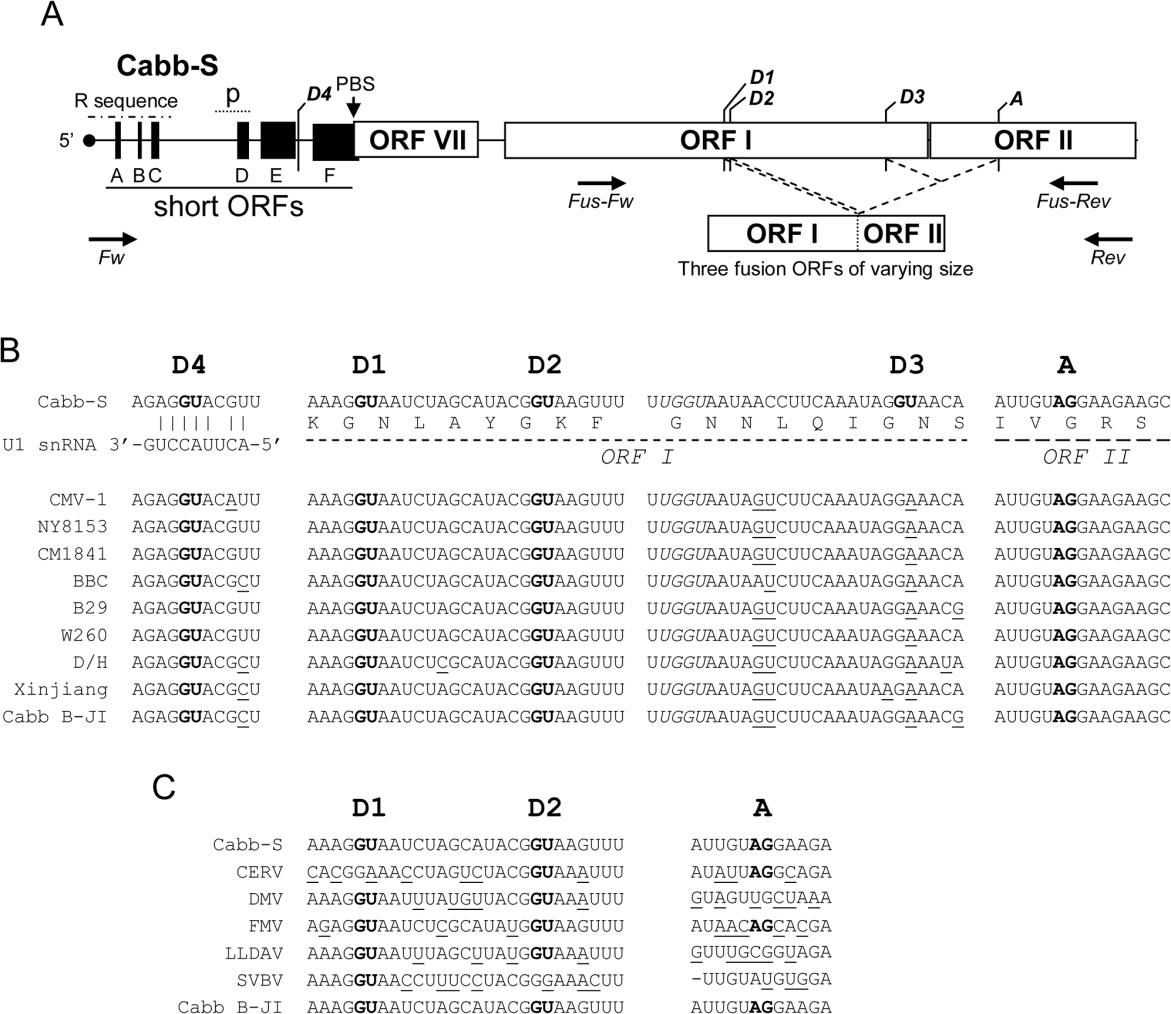
Conservation of 35S RNA splice sites among CaMV isolates and other *Caulimoviruses*. (A) Map of the 5’ region (about 2,500 nucleotides) of 35S RNA of Cabb-S isolate. Empty boxes represent major ORFs, while black boxes represent short ORFs (sORFs) in the leader region. D1 to D4 are the splice donor sites and A, the acceptor splice site described by Kiss-László *et al*. (1995). Dotted lines mark the splicing events leading to in-frame fusions between the 5’ and 3’ extremities of ORF I and ORF II, respectively. The putative encapsidation signal (p) and the primer binding site (PBS) where reverse transcription is initiated are shown. The repeated sequence R corresponds to an approximately 180 nucleotide-long sequence found in both termini of the 35S RNA. The positions of the forward (Fw) and reverse (Rev) primers used to amplify the 5’ part of the 35S RNA and the positions of the primers *Fus-Fw* and *Fus-Rev* used to assess the presence of P1P2 fusion protein mRNA in transient expression assays are indicated by opposite arrows. (B) Alignment of 35S RNA sequences from various CaMV isolates. The sequences surrounding Cabb-S splice sites are shown. The GU and AG nucleotides of donor and acceptor sites, respectively, are in bold. Nucleotides differing from the Cabb-S sequence are underlined. The UGGU sequence which is totally conserved among CaMV isolates is in italics. Amino acids encoded by the regions containing the spliced sites are provided below the nucleotide sequences. The interaction between U1 snRNA and the exon-intron junction is shown for D4 site. (C) Alignment of the 35S RNA sequence from *Caulimovirus* species.

*Cauliflower mosaic virus* (CaMV) is the type member of the genus *Caulimovirus*. Its double-stranded DNA genome is replicated by reverse transcription of the full-length pregenomic 35S RNA [15]. This polycistronic RNA also serves as mRNA for the production of a complete set of proteins thanks to translational reinitiation mediated by the multifunctional protein P6/transactivator–viroplasmin (P6/TAV), which is expressed from the subgenomic 19S RNA [16]. Splicing occurs in the 35S RNA of CaMV-S (Japan) isolate, as evidenced by the characterization of a viral DNA carrying a deletion that perfectly corresponds to an intron in infected turnip plants [17]. The 35S RNA of CaMV Cabb-S (Strasbourg) isolate undergoes alternative splicing that generates four spliced isoforms in the course of its infectious cycle [18]. Spliced RNAs represent approximately 70% of total viral RNA in CaMV-infected plants [18], thus implying that splicing is regulated, as in retroviruses, to preserve a fraction of full-length 35S RNA. Alternative splicing in Cabb-S involves four splice donor sites, one located within the 5’ untranslated region (UTR) and three in ORF I, and a single splice acceptor site within ORF II (Fig 1A). Three spliced 35S RNAs contain a fusion ORF between the 5’ part of ORF I which encodes the movement protein (P1) involved in the cell-to-cell spread of CaMV [19] and the 3’ region of ORF II which encodes a factor (P2) involved in the transmission of CaMV by aphids [20]. Up to now, no function has been assigned to the fusion protein P1P2. Splicing has been shown to be essential for CaMV Cabb-S, since mutations that inactivate the unique splice acceptor site totally abolish infectivity in turnip plants [18,21]. It has been hypothesized that alternative splicing of the 35S RNA provides appropriate mRNAs to enhance the expression of the virion-associated protein (VAP) and the capsid protein (CP) encoded by ORFs III and IV, respectively, and modulates the expression of ORF II [21] and ORF I. Indeed, it has been shown that splicing of the 35S RNA is dispensable in CaMV mutants harboring a complete deletion of ORF II. In addition, the acceptor site has been found to be no longer required if ORF II is replaced by a foreign ORF (i.e. ORF coding for thioredoxin) or if mutations introduced in ORF II lead to a protein P2 which does not accumulate in infected cells [21]. Therefore, a function of 35S RNA splicing would be to downregulate ORF II, whose excessive expression would be toxic for CaMV [21]. However, a role has yet to be assigned to CaMV alternative splicing, since a single splicing event should be sufficient to downregulate the expression of ORFs I and II and simultaneously to permit an efficient translation of downstream ORFs III and IV.

In this study, we show that alternative splicing is a conserved phenomenon among CaMV isolates and that it is more complex than previously described for Cabb-S isolate. We identify new splice donor and acceptor sites that allow the production of at least thirteen spliced 35S RNA isoforms during infection. The fusion proteins P1P2 encoded by some spliced RNAs do not seem to be important for the virus since they do not accumulate upon infection and preventing their production has no effect on the virus infectivity. We show that inactivation of splice sites by mutagenesis is not lethal for CaMV since alternative splicing is kept functional by activation of cryptic splice donor and acceptor sites, emphasizing the key role of this process in CaMV biology.

## Materials and Methods

### Sequence alignments

Complete sequences of CaMV isolates and *Caulimovirus* species were retrieved from Genbank. Accession numbers of the CaMV isolate genomes are the following: Cabb-S (NC_001497.1), CMV-1 (M90543.1), NY8153 (M90541.1), CM1841 (V00140.1), BBC (M90542.1), B29 (X79465.1), W260 (JF809616.1), D/H (M10376.1), Xinjiang (AF140604.1), Cabb B-JI (KJ716236). Accession numbers of the aligned *Caulimovirus* genomes are the following: *Carnation etched ring virus* (CERV; NC_003498.1), *Dahlia mosaic virus* (DMV; NC_018616.1), *Figwort mosaic virus* (FMV; NC_003554.1), *Lamium leaf distortion associated virus* (LLDAV; NC_010737.1), *Strawberry vein banding virus* (SVBV; NC_001725.1). Multiple sequence alignments were performed using Clustal Omega program [22] with default settings.

### Host plant and virus inoculation

Three-leaf stage turnips (*Brassica rapa* cv. Tokyo) were mechanically inoculated with 8 μg of linearized recombinant plasmids containing mutated or wild-type genome of two CaMV isolates, Cabb-S [23] (*Sal*I-linearized pCa37) and Cabb B-JI [24] (*Sal*I-linearized pMD324 or *Spe*I-linearized pBS-BJI). Inoculated plants were grown in a greenhouse under long-day conditions (16 h/22°C day-8 h/18°C night).

### Plasmids

All donor and acceptor site mutants were generated by standard site-directed mutagenesis from pMD324 [24] (donor site mutants of the Cabb B-JI isolate), pBS-BJI, kindly provided by Martin Drucker [25], (acceptor site mutants of the Cabb B-JI isolate) or pCa37 (acceptor site mutants of the Cabb-S isolate). The D2-A intron from pMD324 was deleted by PCR to create the P1P2(D2) coding sequence. Human influenza hemagglutinin (HA) and myc-tagged green fluorescent protein (GFP) and P1P2(D2) coding sequences were generated with the Gateway cloning system (Invitrogen) from binary vectors pGWB14, 15, 17 and 18 [26]. To fuse the glutathione S-transferase (GST) to P1P2(D2), the P1P2(D2) cloning sequence was amplified by PCR with the following oligonucleotides (5’-CAAGGTACCGGATCCATGGATTTGTATCCAGAAGAAAATA-3’ and 5’-CAAGAGCTCGTCGACTTAGCCAATAATATTCTTTAATCCTT-3’) and cloned via *Bam*HI and *Sal*I into the pGEX-6P-1 vector (GE Healthcare). Coding sequences of the viral proteins P1, P2, P3 and P6/TAV were amplified with the following oligonucleotides (5’- CAAGGTACCGGATCCATGGATTTGTATCCAGAAGAAAATA-3’ and 5’-CAAGAGCTCGTCGACTTATTCTCCACAGATTTCTTTTAATTT-3’ for P1, 5’-CAAGGTACCGGATCCATGAGCATTACGGGTCAACCG-3’ and 5’- CAAGAGCTCGTCGACTTAGCCAATAATATTCTTTAATCCTT-3’ for P2, 5’-CAAGGTACCGGATCCATGGCTAATCTTAATCAAATCCAAAAA-3’ and 5’-CAAGAGCTCGTCGACCTAAAATTGATTCGGCCAT CCTG-3’ for P3, 5’-CAAGGTACCGGATCCATGGAGAACATAGAAAAACT-3’ and 5’-CAAGAGCTCGT CGACTCAATCCACTTGCTTTGAAG-3’ for P6/TAV) and cloned into the *Kpn*I and *Sac*I restriction sites of the pETK vector [27] which allows N-terminal fusion to the coding sequence RRASV targeted by the bovine heart muscle kinase (HMK). All constructs were verified by sequencing.

### Total RNA extraction and northern-blotting

Three discs (diameter: 1 cm) from turnip systemic leaves harvested at 25 dpi were ground in a Precellys 24 homogenizer (Bertin Technologies). Total RNA was extracted using TRI-Reagent (Sigma-Aldrich) according to the manufacturer’s instructions and solubilized in sterile water. 15 μg of RNA were separated by electrophoresis on an agarose (0.8%)-urea (7 M) gel in Tris-acetate-EDTA buffer (40 mM Tris-acetate, 1 mM EDTA). An RNA ladder (ssRNA ladder, New England Biolabs) comprising fragments from 0.5 to 9 kb in length was used as a size standard. After electrophoresis, the RNA was electro-transferred onto a nylon membrane (Amersham Hybond-N+; GE Healthcare) in 0.5x Tris-borate-EDTA buffer (45 mM Tris-borate, 1 mM EDTA) at 350 mA for 75 min at 4°C and UV-crosslinked at 1,200 mJ in a Stratalinker 2400 (Stratagene). The membranes were pre-hybridized in PerfectHyb Plus buffer (Sigma-Aldrich) at 42°C for 15 min and incubated overnight at 42°C with ^32^P-radiolabeled DNA oligonucleotide probes (“int”: 5’-CTACAATCTCATTGAGGTTGTTAAGATGA-3’; “ex”: 5’- CTTCCATATTCCGAGTAAGCTTCTTC-3’). Non-specifically bound probes were removed by washing the membranes in 5x saline-sodium citrate buffer (SSC; 0.75 M NaCl, 75 mM saline-sodium, pH 7), 0.1% sodium-dodecyl-sulfate (SDS) and in 2x SSC, 0.1% SDS at 50°C for 30 min and 5 min, respectively. The ratio between the different RNA isoforms was determined by densitometry analysis performed with the software ImageJ [28].

### RT-PCR

2.5 μg of total RNA were treated with 2.5 U of DNase I (Promega) at 37°C for 30 min and extracted with TRI-Reagent. cDNAs were synthetized with the SuperScript III reverse transcriptase (Invitrogen) in the presence of oligo(dT)_18_ primer and used as template for the GoTaq Flexi DNA polymerase (Promega). For PCR, primers targeting the 5’ region of the 35S RNA were 5’-CAAGCGCGCTCTAGACTGAAATCACCAGTCTCTCTC-3’ and 5’-CAAGAGCTCCTGCAGTTAGCCAATAATATTCTTTAATCCTT-3’. Primers to amplify the GFP coding sequence were 5’-CTTAGCAAGGGCGAGGAGCTGT-3’ and 5’-CTTGTACAGCTCGTCCATGCC-3’ and the ones to amplifly the ORF encoding P1P2(D2) were 5’-CGAGATTCACTACTGCGTC-3’ (*Fus-Fw*) and 5’-CAACTGCAGTCTAGACTTTTAATTCTAGTATTTT-3’ (*Fus-Rev*).

### Analysis of spliced 35S RNA

The 5’ part of the 35S RNA was amplified by RT-PCR using the conditions described above. Amplicons were fractionated on a 1% agarose gel, extracted with the NucleoSpin Gel and PCR Clean-up kits (Macherey-Nagel) and cloned into pGEM-T Easy vector (Promega). In the case of acceptor mutants, the PCR following the RT reaction was performed with the Phusion High-Fidelity DNA polymerase (Thermo Scientific) and the amplicons were cloned into pJET1.2 vector (Thermo Scientific). Before sequencing the clones, we maximized their diversity as follows. The insert for each clone was amplified by PCR, using the primers targeting the 5’ part of 35S RNA as described above. PCR products were fractionated by agarose gel electrophoresis and clones having amplicons with the same length were excluded to prevent redundancy between inserts. Remaining clones were then sequenced and DNA sequences were aligned with the 35S RNA sequence using the Serial Cloner 2.6 software.

### Protein analysis

Three discs (1 cm diameter) of systemic or agroinfiltrated leaves were ground in 80 μl 250 mM Tris-HCl, pH 7.75 1 mM DL-Dithiothreitol and mixed with 40 μl of extraction buffer (75 mM Tris-HCl, pH 6.8, 9 M urea, 4.3% SDS, and 7.5% β-mercaptoethanol). Extracts were denatured at 95°C for 5 min. Proteins were separated by SDS-polyacrylamide gel electrophoresis (SDS-PAGE) and electrotransferred to a polyvinylidene fluoride membrane (PVDF; Immobilon-P, Merck Millipore). Anti-P1, kindly provided by Andy Maule (John Innes Center, Norwich, England), anti-P2, kindly provided by Stéphane Blanc (INRA, Montpellier, France), anti-P3 [29], anti-P4 [30] and anti-P6/TAV [31] polyclonal antisera, previously obtained and tested in our laboratory, were used at a 1:10,000 dilution. Monoclonal anti-HA (Thermo Scientific Pierce) and polyclonal anti-myc (Santa Cruz Biothechnology) antibodies were used at 1:10,000 and 1:2,500 dilutions, respectively. Horseradish peroxidase-labeled secondary antibodies were used at a 1:20,000 dilution and revealed with Lumi-Light Plus ECL (Roche). Chemiluminescence was visualized with a Fusion FX (Vilber Lourmat) acquisition system. Alternatively, alkaline phosphatase-labeled secondary antibodies were used at a 1:5,000 dilution and immunocomplexes were revealed by using 5-bromo-4-chloro-3-indolyl-phosphate and nitro blue tetrazolium (Promega).

### Transient expression assays by agroinfiltration

HA- and myc-tagged P1P2(D2) or GFP coding vectors were transferred into *Agrobacterium tumefaciens* GV3101. Transformed *Agrobacteria* were grown overnight at 28°C in Luria-Bertani broth supplemented with 10 mM 2-(N-Morpholino)ethanesulfonic acid (MES) and 40 μM acetosyringone in the presence of appropriate antibiotics. Overnight cultures were pelleted at 3,500 X *g* for 10 min, suspended in 10 mM MES, 10 mM MgCl_2_ and 150 μM acetosyringone and incubated at room temperature for 5 h under constant shaking. Cultures were diluted at 0.5 OD and mixed with the same volume of a 0.5 OD *Agrobacterium* culture carrying the pBIN61-P19 vector [32] prior to infiltration. Six to eight week-old *Nicotiana benthamiana* plants were infiltrated using a needleless syringe. All samples were harvested at 2.5 days post infiltration.

### Recombinant expression and labeling of viral proteins

Recombinant viral proteins P1, P2, P3, P6/TAV and GST-P1P2(D2) were expressed in *Escherichia coli* BL21(DE3)pLysS transformed with the respective plasmids (see “Plasmids” section for details concerning their construction). Their expression was induced in bacteria culture in exponential phase by adding 1mM Isopropyl β-D-1-thiogalactopyranoside. Bacteria were then grown at 28°C for 4 h, pelleted and suspended in heart muscle kinase (HMK) buffer (20 mM Tris-HCl, pH 7.5, 100 mM NaCl, and 12 mM MgCl_2_). After being sonicated 3 times for 10 s, lysates were centrifuged at 11,000 X *g*, for 10 min at 4°C and the inclusions bodies were suspended in 0.5 mL HMK buffer. 30 μl of the inclusion bodies suspension were phosphorylated in the presence of 25 μCi [γ-^32^P]ATP and 25 U of HMK (Sigma-Aldrich) in 150 μl HMK buffer, at room temperature for 1.5 h. ATP in excess was eliminated using an illustra MicroSpin G-25 Column (GE Healthcare).

### Far Western assays

The GST-P1P2(D2)-containing bacterial fraction was separated by SDS-PAGE, electro-transferred to a PVDF membrane (Immobilon-P; Merck Millipore) and saturated in phosphate-buffered saline 1x (137 mM NaCl, 2.7 mM KCl, 10 mM Na_2_HPO_4_, 2 mM KH_2_PO_4_), Tween 20 1%, milk 5% at room temperature for 12 h. The membrane was incubated either with ^32^P-radiolabelled viral proteins or with purified virus particles at 4°C for 12 h and washed in phosphate-buffered saline 1x-Tween 20 1% for 10 min. The interaction between the fusion protein and virus particles was detected with anti-P4 polyclonal antibodies (1:10,000 dilution) and alkaline phosphatase-labeled secondary antibodies (1:5,000 dilution).

### Protoplast isolation and immunolabeling

Protoplasts were isolated at 21 dpi from healthy or infected turnip plants as follows. Leaves were chopped and incubated in an enzyme solution (0.4 M mannitol, 20 mM KCl, 20 mM 2-(N-morpholino)ethanesulfonic acid, 1.5% cellulase R10 and 0.4% macerozyme R10 (Yakult Pharmaceutical)) at 25°C for 4 h. Leaf debris were removed by filtrating the digestion medium through two layers of Miracloth (Merck Millipore). To eliminate the enzymes, the protoplasts were collected by centrifugation (100 X *g*, for 2 min, without brake) and gently suspended in 0.5 M mannitol. This washing step was repeated three times. Fixation and immunolabeling of isolated protoplasts were performed as described by Haas and colleagues [33], except that cells were fixed in a 0.5 M mannitol and 1% glutaraldehyde containing medium. Rabbit polyclonal anti-P2 antibodies and mouse anti-rabbit IgG conjugated to Alexa 488 (Molecular Probes) were used to label the fixed protoplasts. Immunolabeled protoplasts were observed with a LSM700 confocal microscope (Zeiss).

## Results

### *In silico* analysis reveals that splice sites of 35S RNA are conserved among CaMV isolates and other *Caulimoviruses*

The comparison of the complete DNA sequence of ten CaMV isolates revealed that the 5’ part of their pregenomic 35S RNA possesses three out of the four splice donor sites described for CaMV Cabb-S isolate (D4 in the 5’ UTR and D1 and D2 in ORF I; nomenclature from [18]), and the splice acceptor site (A in ORF II). The exon-intron boundaries are conserved in all the sequenced CaMV isolates (Fig 1B): AG**GU** sequence for D1 and D4, CG**GU** for D2 and **AG**GA for A (the 5’ and 3’ invariant dinucleotides characteristic of the eukaryotic intron extremities are in bold). Moreover, these three sites are totally conserved (99–100% identity) in all the 67 genome sequences that have recently been released [34] (S1 Text). By contrast, the donor site D3 used for splicing in Cabb-S [18] is not present in the other isolates where the dinucleotide GU is replaced by GA. 35S RNA contains a putative donor site located 18 nucleotides upstream of D3 and bearing a UG**GU** sequence that is totally conserved among all CaMV isolates. The corresponding exon-intron junction anneals well with the U1 small nuclear RNA (U1 snRNA; Fig 1B) of higher plants [35] but its use by the spliceosome has never been reported. Several other cryptic donor and acceptor sites are present along the 5’ region of CaMV pregenomic RNA. Analysis of the 5’ part of the 35S RNA sequence of some other members of the *Caulimovirus* genus showed that D1 and/or D2 as well as the acceptor site are also present in *Figwort mosaic virus* and *Carnation etched ring virus* (Fig 1C). A statistical comparison between the nucleotide sequences of D1, D2 (the 8 nucleotides of the exon-intron boundaries annealing to U1 snRNA were compared) and A (the 15 nucleotides recognized by splicing factors at the 3’ end of the introns were compared) splice sites shows that they are well conserved among the *Caulimoviruses*, with a nucleotide identity of 50%, 75% and 46%, respectively, compared to the general coding sequence which has a nucleotide identity of only 33% in ORF I and 26% in ORF II. The conservation of the splice sites implies that splicing or alternative splicing could also be important for the infectivity of the *Caulimoviruses*.

Taken together, *in silico* analyses strongly suggest that alternative splicing of the pregenomic 35S RNA is a conserved phenomenon among CaMV isolates and that it may also occur in other *Caulimovirus* species.

### 35S RNA of CaMV Cabb B-JI isolate undergoes alternative splicing

**Fig 2:**
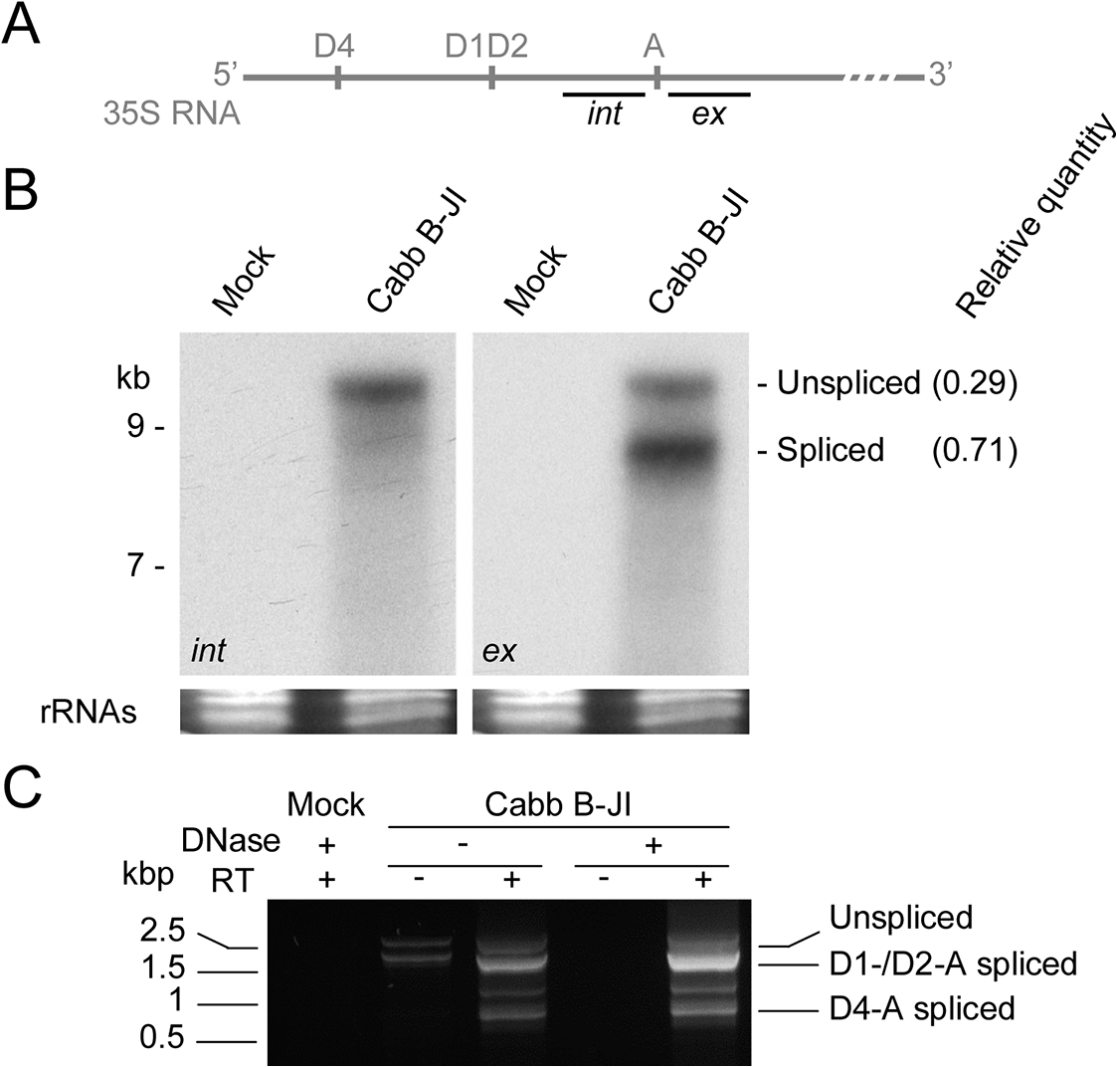
35S RNA of CaMV Cabb B-JI isolate is alternatively spliced. (A) Oligonucleotide probes targeting the intronic (*int*) or the exonic (*ex*) region surrounding the acceptor site were used to detect 35S RNA and its putative spliced isoforms. (B) Northern-blotting analysis of turnip total RNA extracted at 21 dpi. RNA was separated by electrophoresis on an agarose (0.8%)-urea (7 M) gel, transferred onto a nylon membrane and probed with ^32^P-labeled *int* or *ex* probes. Relative ratio between the unspliced 35S RNA and its spliced isoforms is indicated. (C) RT-PCR on turnip total RNA extracted at 21 dpi. The 5’ region of 35S RNA was amplified and the products separated on agarose gel.

A spliced 35S RNA corresponding to D1-A isoform was previously identified in CaMV Cabb B-JI-infected *Arabidopsis thaliana* [36]. To investigate in detail 35S RNA splicing of this CaMV isolate during the infectious cycle, total RNA was extracted from CaMV-infected turnip plants at 21 dpi, separated by agarose-urea gel electrophoresis and analyzed by northern blot. In all our agarose-urea gel electrophoresis experiments, the theoretically 8.2 kb-long full length 35S RNA migrated slower than the 9 kb-long standard RNA fragment (Fig 2B). This might be due to the urea concentration (7M) in the gels which would be insufficient to completely denature the strongly folded 35 RNA leader sequence. The full-length 35S RNA was detected with an oligonucleotide probe (*int*) targeting a sequence immediately upstream of the splice acceptor site (Fig 2A and 2B). Full-length and smaller 35S RNA-derived versions were detected with a probe (*ex*) that is complementary to a sequence immediately downstream of the splice acceptor site (Fig 2A and 2B). A densitometry analysis revealed that the smaller 35S RNA molecules corresponded to 70% of the global pool of 35S RNA. No band was present when RNA from mock-inoculated plants was tested with both probes (Fig 2B). RT-PCR on total RNA was performed to amplify the 5’ region of the 35S RNA encompassing the putative splicing sites (Fig 2C). Analysis of the amplification products by electrophoresis showed that the major amplicons were consistent with the predicted sizes of cDNAs corresponding to the non-spliced 35S RNA (2.4 kbp) and its D1-, D2- (1.8 kbp) and D4- (0.8 kbp) spliced isoforms. Sequencing of these amplicons unambiguously showed that they correspond to the three predicted 35S RNA spliced isoforms and, consequently, that CaMV Cabb B-JI follows a similar alternative splicing pattern to Cabb-S isolate, except for the D3 site. As expected from the *in silico* analysis, a cDNA corresponding to D3-A spliced RNA described for Cabb-S could never be amplified from RNAs extracted from Cabb B-JI-infected turnips, confirming that this splice donor site does not exist in this isolate. Additional uncharacterized 1.1 and 0.7 kbp-long amplicons were observed, raising the possibility that some other spliced and/or deleted isoforms of 35S RNA might exist (see below). Amplification products were also obtained in the minus-DNase/minus-RT lane but not in the plus-DNase/minus-RT lane (Fig 2C), indicating the presence of contaminating viral DNA. The profile observed after gel electrophoresis was similar to the ones obtained after RT, and sequencing revealed deletions identical to D1- and D2-A splicing indicating that some spliced isoforms are reverse-transcribed upon infection.

### Fusion proteins P1P2 are undetectable in infected plants and dispensable for infectivity

**Fig 3:**
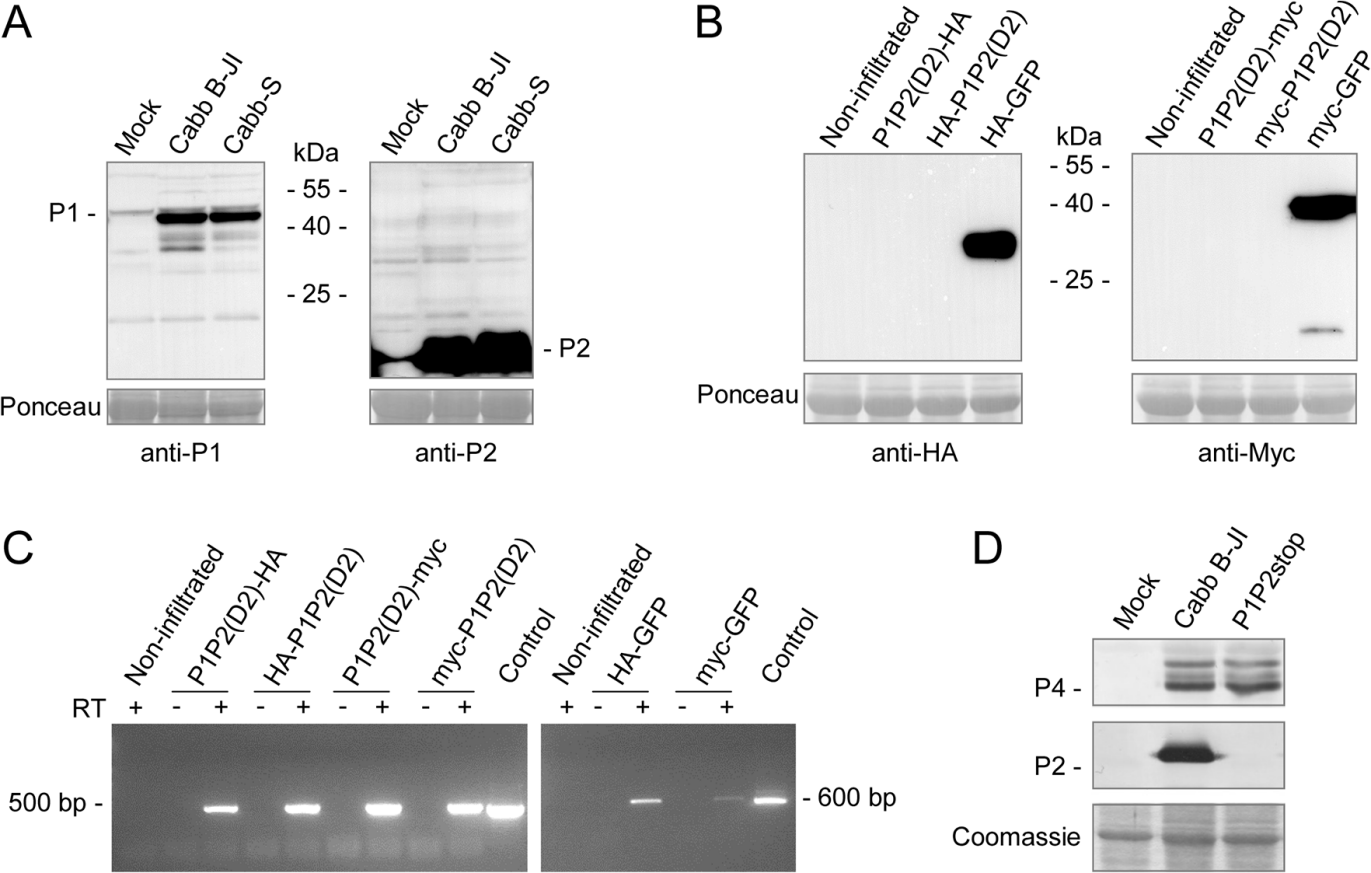
P1P2 fusion proteins are unstable in plants, both in a viral context and in transient expression assays, and are dispensable for infectivity. (A) Immunodetection of P1P2 fusion proteins in CaMV Cabb B-JI- or Cabb-S-infected turnip plants. Total proteins were analyzed by SDS-PAGE and western blotting was performed with anti-P1 and anti-P2 polyclonal antibodies. (B) Immunodetection of HA- or myc-tagged P1P2(D2) protein upon *Agrobacterium tumefaciens*-mediated transient expression assay in *Nicotiana benthamiana* leaves. Total proteins were extracted from agroinfiltrated leaves and the presence of P1P2(D2) fusion protein was assessed by western assays with anti-HA or anti-myc antibodies. Leaves agroinfiltrated with HA- or myc-GFP expression vectors were used as positive controls. Blots in (A) and (B) are overexposed deliberately to maximize chances of detection of P1P2 proteins. (C) Tagged-P1P2(D2) and GFP transcripts were assessed by RT-PCR on total RNA from infiltrated leaves. The recombinant plasmid pP1P2(D2) used for expression of the fusion protein in *E*. *coli* and pCK-EGFP [27] were used as controls. (D) Immunodetection of viral proteins P2 and P4 in systemic leaves of Cabb B-JI- or P1P2stop-infected turnip plants at 21 dpi.

The selection of splice donor sites D1 and D2 (and D3 in Cabb-S isolate) and acceptor site A creates fusion ORFs between the 5’ part of ORF I (whose size varies depending on the donor site) and the 3’ part of ORF II, which remains unchanged in the fusion ORFs. The resulting P1P2(D1) and P1P2(D2) fusion proteins have a theoretical molecular mass of about 31 kDa. It has been shown that P2 self-interacts [37], interacts with P3 [38] and P6/TAV [39]. P1, in addition to oligomerizing to form tubules [19] allowing cell-to-cell movement, also interacts with P3 [40] and P6/TAV [41]. We therefore hypothesized that the fusion proteins, if expressed, could interact with viral partners of full-length P1 and/or P2 proteins. To assess the putative interactions between P1P2 fusion proteins and viral proteins, we performed far western experiments on GST-P1P2(D2) fusion protein expressed in *E*. *coli*, using recombinant and radiolabelled viral proteins P1, P2, P3 and P6/TAV or purified CaMV particles as overlay. GST-P1P2(D2) interacted with purified viral particles, P2 and P3, but neither with P1 nor with P6/TAV (S1 Fig) whereas GST alone did not interact with any of these proteins. Surprisingly and in spite of numerous attempts, fusion P1P2 proteins were not detected in CaMV-infected turnip plants when we used polyclonal anti-P1 and anti-P2 antibodies, whereas the P1 protein (46 kDa), its cleavage product (38 kDa) [42], P2 (18 kDa) (Fig 3A) as well as the P1P2(D2) fusion protein when expressed in *E*. *coli* (S2 Fig) were revealed with these antibodies (Fig 3A), suggesting that the fusion proteins are weakly expressed and/or degraded shortly after their synthesis. Moreover, the expression of HA- and myc-tagged P1P2(D2) was investigated by agroinfiltration of *Nicotiana benthamiana* leaves. Agrobacteria carrying plasmids encoding HA- or myc-eGFP were used to infiltrate control leaves. Both control proteins were always revealed with antibodies raised against their tag. However, the tagged P1P2(D2) proteins were not immunodetected in crude protein extracts (Fig 3B) although their mRNAs were detected by RT-PCR in the infiltrated leaves (Fig 3C), suggesting that these proteins are rapidly degraded after their expression. This degradation might be plant- and/or eukaryote-specific since fusion proteins can be expressed in *E*. *coli*. To assess whether preventing the expression of full-length P1P2 proteins would be deleterious for CaMV infectivity, we developed a mutant called P1P2stop in which we inserted a stop codon a few nucleotides downstream of the acceptor site (the sequence **AG**GAAGAAGCUUACUCGGA is replaced with **AG**GAAGAAGCUUACUCUGA, the acceptor site is in bold and the inserted stop codon is underlined). Mutations were not introduced in ORF I to study the biological relevance of P1P2 fusion proteins because they impair CaMV systemic movement. The P1P2stop mutant was as infectious as wt Cabb B-JI in turnip plants and the capsid protein P4 accumulated at similar level in systemic leaves (Fig 3D). This indicates that the P2 part of the P1P2 fusion proteins is dispensable for infectivity, at least in our experimental conditions. However, this does not exclude the possibility for a role of the P1 part in the viral cycle.

### A cryptic donor site rescues splicing upon mutation of D1, D2 and D4 sites

**Fig 4:**
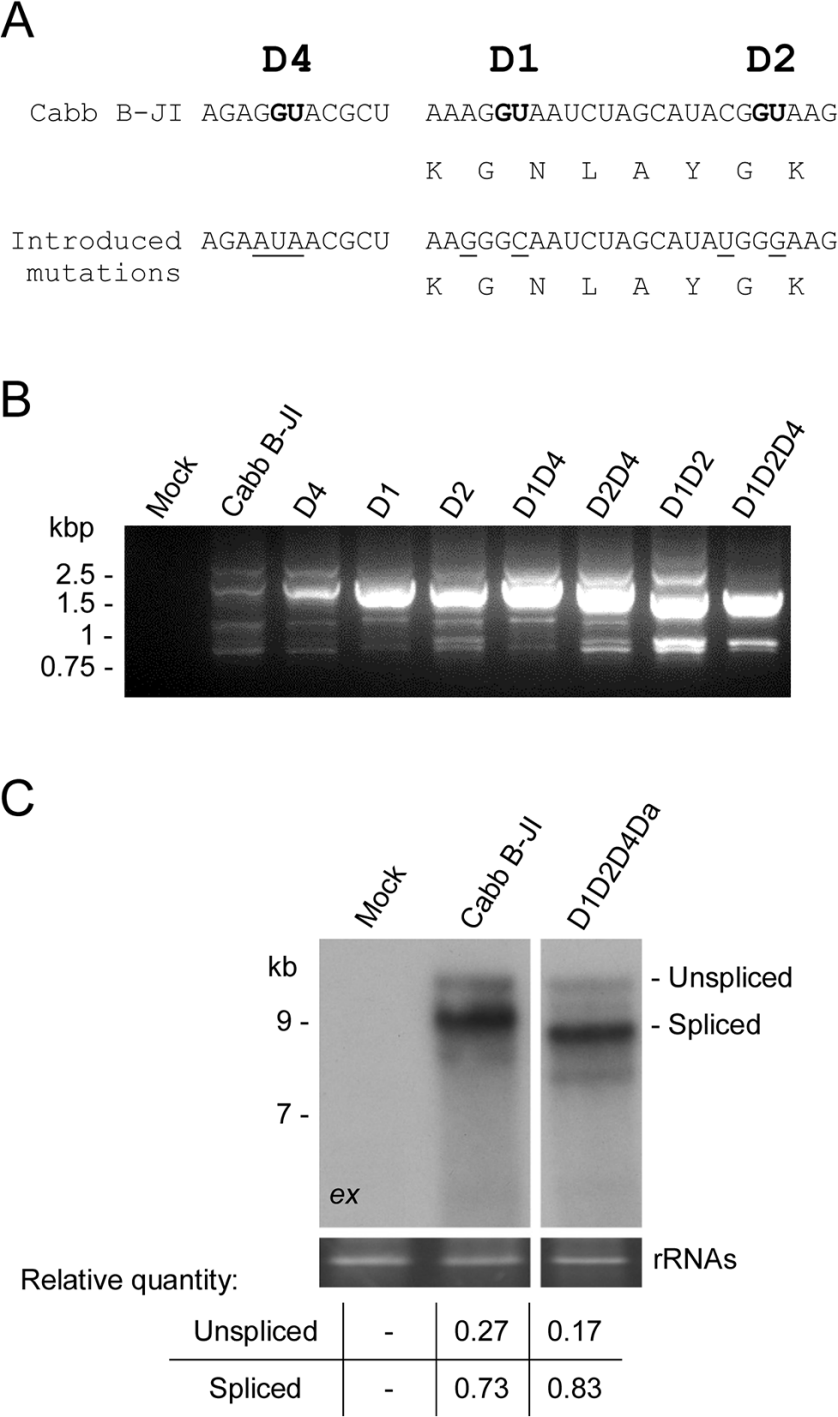
Mutations of splice donor sites are rescued by the use of a cryptic donor site. (A) Exon-intron junctions of splice donor sites of CaMV Cabb B-JI were mutated independently or in combination. The GU donor sites are in bold, and mutated nucleotides are underlined. Corresponding amino acids are provided below the nucleotide sequences surrounding D1 and D2 sites. (B) Analysis by RT-PCR of the splicing pattern of the donor site mutants. Total RNA from infected turnips was extracted at 21 dpi and the 5’ region (about 2,500 nts) of 35S RNA was amplified. Amplicons were separated by agarose gel electrophoresis. (C) Northern-blotting analysis of total RNA extracted from turnip plants infected with wt CaMV and the quadruple mutant D1D2D4Da using the radiolabelled *ex* probe, as described in Fig 2. Relative ratio between the unspliced 35S RNA and its spliced isoforms is indicated.

**Fig 5:**
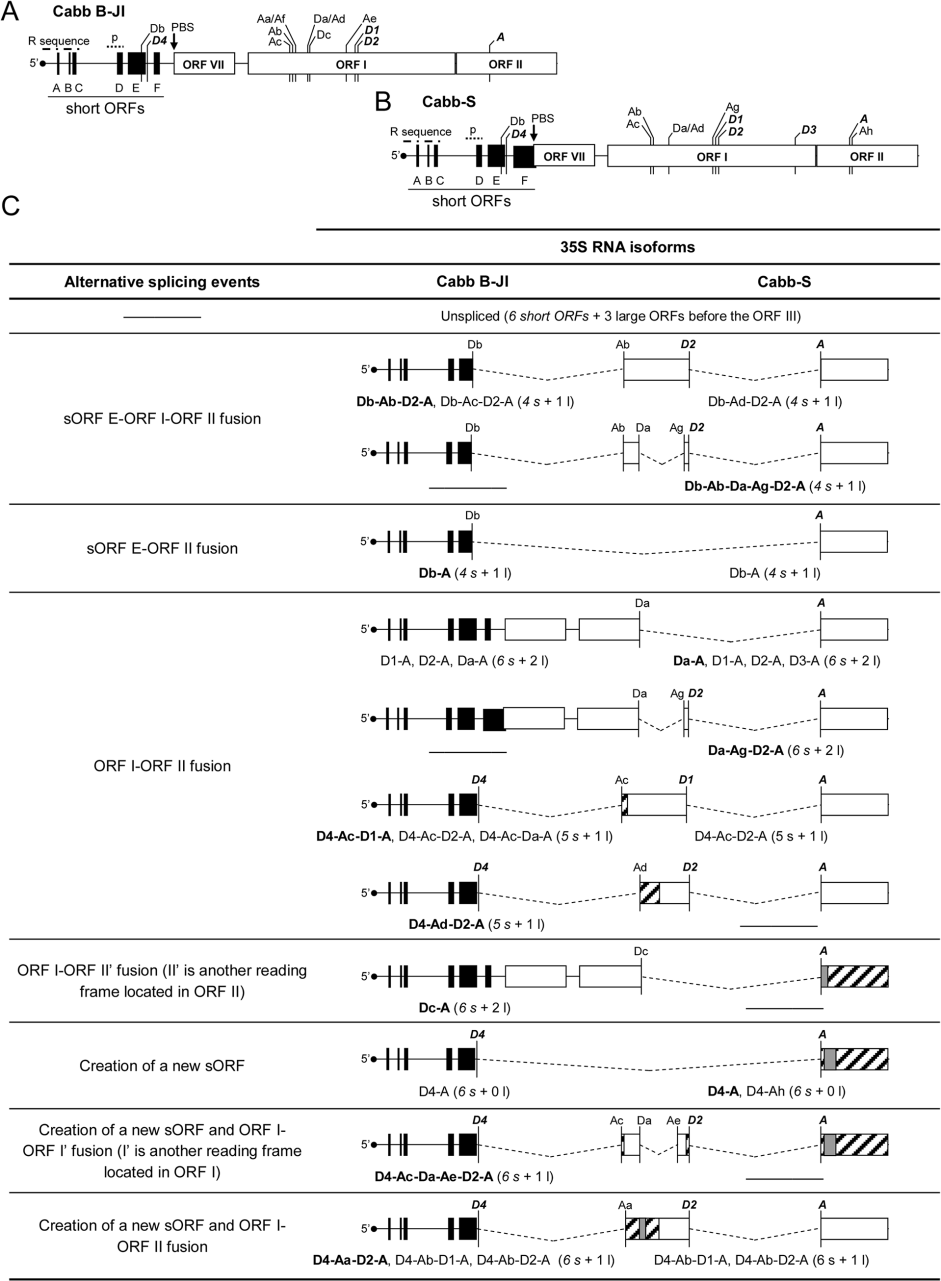
The 5’ part of Cabb B-JI and Cabb-S 35S RNA contains numerous splice sites. The 5’ region of Cabb B-JI (A) and Cabb-S (B) 35S RNA (about 2,500 nts) is schematized. Previously discovered sites are in bold italic. The putative encapsidation signal (p) and the primer binding site (PBS) where reverse transcription is initiated are shown. The repeated sequence R corresponds to an approximately 180 nucleotide-long sequence found in both termini of the 35S RNA. (C) Splicing events and corresponding isoforms in Cabb B-JI and Cabb-S isolates. Splicing is represented by dotted lines. For each event, isoforms are indicated and one or several representative isoforms (in bold) are schematized. Isoforms that are very similar to the ones schematized are only indicated. The number of short (*s*) and large (l) ORFs that are upstream of ORF III is given in brackets. Regions of ORFs I or II that are no longer coding in some spliced isoforms are hatched. Coding sequences located within ORFs I or II but in a different reading frame are in grey.

Previous studies have suggested that splicing of the 35S RNA is essential for CaMV to downregulate the expression of P1 and P2 [18,21]. However, as one donor site should be sufficient for this purpose, and because all CaMV isolates have conserved the D1, D2 and D4 donor sites, we hypothesized that alternative splicing must have other regulatory functions. To assess the biological relevance of the splice donor sites, we inactivated them by site-directed mutagenesis in the viral vector pMD324 containing the complete Cabb B-JI genome. The mutations introduced into the exon-intron junctions weakened the interaction with U1 snRNA but, in the case of D1 and D2, maintained the coding sequence of ORF I in order to safeguard the function of movement protein P1 (Fig 4A). Single, double and triple mutants were generated, and the resulting plasmids were mechanically inoculated in three-leaf stage turnip plants. Ten plants were inoculated with each splicing mutant in four independent experiments. All CaMV mutants were infectious on turnip plants including, surprisingly, the triple mutant D1D2D4 that was expected to be lethal for the virus due to predicted abolition of splicing [18,21]. Two weeks after inoculation, all turnip plants expressed the same systemic symptoms (mosaic, vein-clearing and stunting) as plants inoculated with the wild-type (wt) CaMV genome did, and no delay in symptom appearance was observed. Sequencing of viral DNA extracted from systemic leaves did not reveal any reversion of mutations. The 35S RNA 5’ region encompassing the donor and acceptor sites was amplified by RT-PCR. Amplification products from wt CaMV, all the single mutants, D1D4 and D2D4 double mutants were analyzed by gel electrophoresis and revealed an almost similar pattern, displaying a major band (1.8 to 1.7 kbp) and several minor bands, among which the unspliced 35S RNA (2.4 kbp) and some uncharacterized amplicons (1,2 and 0.9 kbp) that could arise from unknown splicing events (Fig 4B). A 0.9 kbp-long amplicon was notably detected for the mutants where D2 site was disrupted (Fig 4B, lanes D2, D2D4, D1D2 and D1D2D4). The major band obtained with D1D2 and D1D2D4 mutants was smaller (1.5 kbp) than the one observed with the other splicing mutants. This amplicon was extracted from the agarose gel, cloned and sequenced. The sequence corresponded to a new spliced isoform of 35S RNA involving a donor site that is located at position 653 in ORF I (donor site Da, Table 1 and Fig 5A) and the splice acceptor site A. This splicing event, as for D1 and D2, created an in-frame fusion between ORF I and ORF II.

**Table 1:** Splice donor and acceptor sites found in Cabb B-JI and Cabb-S strains by RT-PCR. 85 and 44 clones from two independent experiments were sequenced for Cabb B-JI and Cabb-S strains, respectively. Sites Da to Dc and Aa to Af were named according to their order of discovery in Cabb B-JI strain.

	Cabb B-JI	Cabb-S
Splice donor sites (position)	D1 (872); D2 (887); D4 (7915); Da (653); Db (7890); Dc (658)	D1 (872); D2 (887); D3 (1247); D4 (7909); Da (653); Db (7884)
Splice acceptor sites (position)	A (1509); Aa (584); Ab (577); Ac (565); Ad (652); Ae (830); Af (587)	A (1508); Ab (577); Ac (565); Ad (652); Ag (864); Ah (1515)

In conclusion, our results show that the donor splice sites of the 35S RNA are not relevant *per se* for CaMV infectivity. On the other hand, splicing itself appears to be, since the virus used a cryptic splice donor site upon their inactivation.

### CaMV Cabb B-JI and Cabb-S 35S RNA exhibit a complex splicing pattern

CaMV was still infectious when a quadruple mutant D1D2D4Da was inoculated in turnip plants (for immunodetection of viral proteins, see S3 Fig), strongly suggesting the presence of other splice sites in the 35S RNA. Total RNA from Cabb B-JI- or D1D2D4Da mutant-infected turnip plants were extracted and analyzed by northern blot using the *ex* probe in the conditions described above. Full-length 35S RNA and smaller 35S RNA molecules corresponding to spliced 35S RNA were detected in turnip plants infected with the quadruple mutant D1D2D4Da (Fig 4C, *ex* probe). The main pool of spliced RNAs migrated slightly faster than the spliced 35S RNAs present in plants infected with the wt virus. A densitometry analysis indicated that the spliced 35 RNA represented 83% of the 35S RNA global population in D1D2D4Da mutant-infected turnip plants (Fig 4C). These results prompted us to investigate in detail the splicing taking place within Cabb B-JI 35S RNA by focusing on its 5’ part, since amplification by RT-PCR of the sequence located downstream of the acceptor site did not reveal any spliced RNA (S4 Fig). Amplicons were obtained by RT-PCR performed on DNase-treated total RNA from turnip plants infected with wt Cabb B-JI, using a pair of primers flanking the 5’ region, from the 35S RNA 5’ terminus to the end of ORF II. After cloning in pGEM-T Easy vector and sequencing, sequences of the amplicons were aligned with 35S RNA. In addition to the spliced RNAs described above, we characterized twelve new spliced RNA isoforms that arose from the alternative use of two other donor sites (Db and Dc) and six acceptor sites (Aa to Af; Table 1 and Fig 5A and 5C). All these new splice sites conform to splice donor and acceptor site consensus sequences GU…AG and were named according to order of discovery. Donor site Db is located in sORF E of the 5’ UTR close to the D4 site, whereas donor site Dc and the six new acceptor sites are in ORF I (Fig 5A). Spliced isoforms resulted from five single, nine double and one triple splicing events, and contained various fusion ORFs involving sORF E, ORF I, ORF II and sequences in other reading frames localized in ORF I and ORF II regions (Fig 5C). Some of these new spliced isoforms could correspond to certain uncharacterized RT-PCR amplicons shown in Figs 2C and 4B. Single spliced 35S RNAs found in turnip plants infected with the CaMV Cabb B-JI isolate indicate the use of donor sites D1, D2 and D4, at almost the same frequency, and acceptor site A. In few cases, the donor sites, Db and Dc, and site A were used to generate single spliced 35S RNAs (Fig 5C and S1 Table). Double spliced isoforms were produced systematically through splicing events involving donor sites D4 and Db present in the leader region, with a strong preference for D4 in combination with Aa, Ab, Ac or Ad acceptor sites located at the 5’ end of ORF I. Splicing of the second intron in the double spliced isoforms preferentially involved donor site D2 and acceptor site A (Fig 5C and S1 Table). Remarkably, the use of donor site Db, which is localized in the 5’-UTR, also leads to the formation of fusion ORFs. In fact, several isoforms possessing fusion ORFs involving sORF E were detected (for example isoforms Db-A and Db-Ab-D2-A; Fig 5C). When we analyzed the viral RNAs extracted from turnip plants infected with CaMV Cabb-S isolate, we characterized nine new spliced isoforms (Fig 5C) thus demonstrating that the splicing pattern of the 35S RNA 5’ region of this isolate is more complex than previously reported [18,21]. Several splice sites are located at the same positions as in Cabb B-JI (sites Da, Db, Ab, Ac and Ad; Fig 5A and 5B, Table 1). We found that single spliced 35S RNAs in turnip plants infected with Cabb-S isolate arose from the use, at high frequency, of donor sites D1 and D2 in association with acceptor site A. In contrast to the situation found in the Cabb B-JI isolate, D4 was only rarely used. The splice acceptor site Ah (position 1515) which is located downstream of the splice acceptor site A (position 1508) also was found to be selected for splicing while in turnip plants infected with Cabb B-JI isolate this acceptor site was inactive except upon inactivation of splice site A (see below). In CaMV-infected Cabb-S-plants we found few single spliced isoforms resulting from the use of the donor site Da located in ORF I and site A (Fig 5C and S1 Table). The double spliced 35S RNAs were generated by using either the D4 or Db donor sites in the leader sequence in combination with acceptor sites Ab, Ac, Ad or Ag, or the D1 or D2 donor sites together with acceptor site A. Surprisingly, the splicing of the 5’ proximal intron in double spliced RNAs involved sites Da and Ag located in ORF I, thus indicating that the leader region is preserved in these isoforms (Fig 5C and S1 Table). In some sequenced clones and for both isolates, it is noteworthy that we found deletions unrelated to splicing. Some of them involved borders around the two sequence discontinuities Δ1 and Δ3 that are remnants of the reverse transcription process and located at the beginning of ORF VII and the end of ORF II, respectively [43]. This implies that recombination occurs between these discontinuities and the size of such recombinants is consistent with some of the short amplicons (0.7 to 0.9 kbp) observed in Figs 2C and 4B.

Taken together, the CaMV transcriptome analysis clearly demonstrates that the 35S RNAs of CaMV Cabb B-JI and Cabb-S isolates both exhibit a complex alternative splicing pattern involving multiple splice donor and acceptor sites, most of them being located within ORF I.

### Cryptic splice acceptor sites are activated to maintain splicing of CaMV 35S RNA

**Fig 6:**
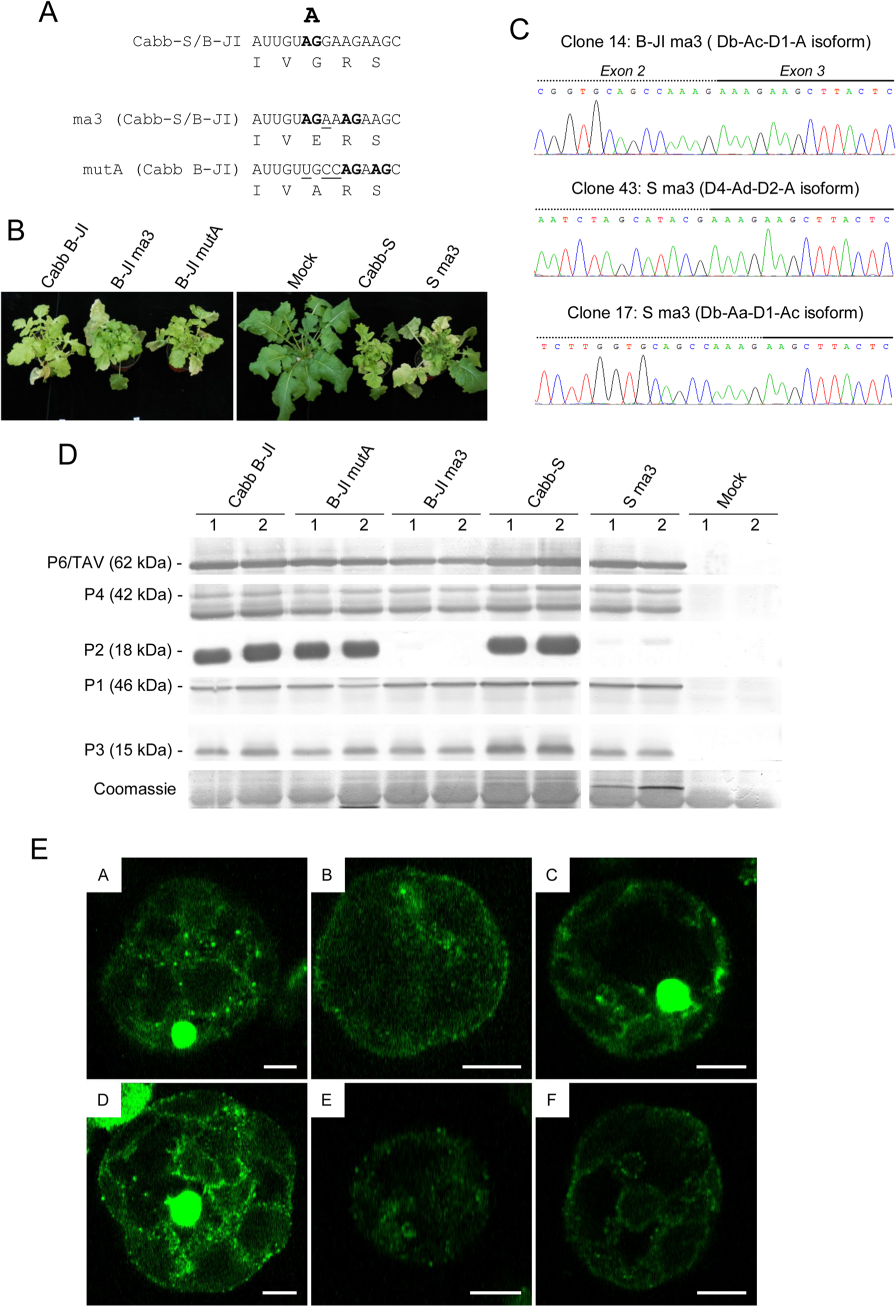
Mutations of splice acceptor site A lead to the use of downstream AG dinucleotides as acceptor sites. (A) Splice acceptor site A was mutated both in Cabb-S and Cabb B-JI isolates. The AG dinucleotides used as acceptor sites in the wild type and mutant viruses are in bold; mutated nucleotides are underlined. Corresponding amino acids are provided below the nucleotide sequences. (B) Symptoms on turnip plants at 45 dpi. (C) Sequences at the second splice junction of three isoforms cloned from splice acceptor mutant-infected turnips (B-JI ma3 and S ma3) after RT-PCR amplification on total RNA. Exons 2 and 3 are represented by a dotted line and a solid one, respectively. (D) Immunodetection of viral proteins P1, P2, P3, P4 and P6/TAV in CaMV Cabb B-JI-, Cabb B-JI mutA-, Cabb B-JI ma3-, Cabb S- or Cabb Sma3-infected turnips plants. Total proteins from systemic leaves were analyzed by SDS-PAGE, and western blotting was performed with polyclonal antibodies. Two independent replicates are shown. (E) Electron-lucent viroplasms are not detected in ma3 mutant-infected cells. Protoplasts isolated from Cabb B-JI- (A), B-JI ma3- (B), B-JI mutA- (C), Cabb-S- (D), S-ma3-infected turnip plants (E) or from healthy plants (F) were fixed then immunolabeled with rabbit anti-P2 antibodies and mouse anti-rabbit antibodies conjugated to Alexa 488. Fluorescence images were collected by confocal laser scanning microscopy. Scale bars: 5 μm.

The fact that disruption of splice donor sites can be rescued by the use of other sites and the characterization of several splice acceptor sites suggested that acceptor site A is not as crucial for infectivity as it was previously stated [18,21]. We therefore introduced a mutation previously considered sufficient to prevent the use of the acceptor site A in Cabb B-JI genome (**AG** G to AGA, mutant JI-ma3; Fig 6A). In our experimental conditions, turnip plants inoculated with the mutant JI-ma3 developed the same systemic symptoms (mosaic, vein clearing and stunting) as those inoculated with the wild-type isolate (Fig 6B) without any delay. To determine whether the behavior of this mutant was isolate-specific, we tested the infectivity of a Cabb-S genome containing the same mutation (mutant S ma3; Fig 6A). In conflict with previous reports [18,21], the S ma3 mutant was as infectious as the wt Cabb-S virus on turnip plants (Fig 6B). Sequencing of the 5’ part of 35S RNA in the viral progeny of both isolates showed neither a reversion of the introduced mutations nor other modifications such as deletion and/or introduction of a stop codon in ORF II, which could have explained infectivity of these mutants. In fact, RT-PCR amplification and sequencing of the 5' region of the CaMV 35S RNA isoforms revealed that splice acceptor site A (position 1509 in Cabb B-JI and 1508 in Cabb-S) was still active despite the introduced mutation (Fig 6A and 6C, clones 14 and 43). For some isoforms, the AG dinucleotide immediately downstream (acceptor site Ah, Fig 5B) was used as acceptor site (Fig 6A and 6C, clone 17). Viral proteins P1, P3, P4 and P6/TAV accumulated at similar levels in ma3-mutant infected turnip plants compared to plant infected by the wt viruses, except for P2 which was not (Fig 6D, lanes B-JI ma3) or barely detected (Fig 6D, lanes S ma3). Consistently, we did not detected any electron-lucent viroplasms (also called transmission bodies), which are inclusion bodies containing all the P2 protein [44], in protoplasts isolated from B-JI ma3- and S ma3-infected turnip plants (Fig 6E). To fully disrupt site A, we introduced a mutation into the dinucleotide AG (**AG** G to UGC, mutant B-JI mutA; Fig 6A) and inoculated this mutant in turnip plants. Plants inoculated with B-JI mutA expressed typical CaMV systemic symptoms with no delay compared to Cabb B-JI-infected plants (Fig 6B) and they accumulated similar quantities of viral proteins, including P2 (Fig 6D and 6E). Sequence analysis of the spliced viral RNAs extracted from systemic leaves confirmed mutation at site A and revealed that two other downstream AG dinucleotides were used as acceptor sites for splicing (Fig 6A).

In conclusion, these results show that CaMV maintains splicing of its 35S RNA thanks to the use of cryptic splice acceptor sites in ORF II, thus explaining why inactivation of acceptor site A was not lethal for CaMV. They also reinforce the hypothesis that splicing of 35S RNA is necessary for CaMV replication.

## Discussion

Alternative splicing of 35S RNA potentially occurs in many CaMV isolates since they possess three of the four splice donor sites and the splice acceptor site described for Cabb-S isolate [18]. Some of these splice sites have also been found in the 35S RNA of other members of the *Caulimovirus* genus, suggesting that splicing might be a key common process for these viruses. Analysis of amplified cDNAs obtained by RT-PCR of viral RNAs extracted from Cabb B-JI-infected turnip plants revealed that the splicing pattern of 35S RNA is more complex than previously described for Cabb-S isolate: in fact, we identified sixteen isoforms resulting from the use of six splice donor sites and seven acceptor sites. Such complexity was also observed in Cabb-S isolate where thirteen spliced isoforms of 35S RNA were found. Our data shows that spliced isoforms represents about 70% of the total pool of 35S RNA molecules of Cabb B-JI isolate, which is similar to what was reported for Cabb-S isolate [18]. Whether some spliced isoforms are more abundant than others is still an open question which seems to be very difficult to assess with current methods given the number of isoforms, the similarity—in some cases—of their sizes and the fact that several multiply spliced isoforms can have identical exon-exon junctions (for instance, D4-Ac-D2-A and Db-Ac-D2-A isoforms). We never detected spliced RNAs arising from splicing events downstream of ORF II, which is in contrast with the behavior observed in FMV infection, where splicing occurs in the 3' part of the 35S RNA, leading to an in-frame fusion between the 5' region of ORF IV and the 3' region of ORF V that code for the capsid protein and reverse transcriptase, respectively [14]. In theory, the spliced RNAs can be used as template for the reverse-transcriptase since they possess at their 5' and 3' ends a repeated region (R region) involved in intra- or inter-molecular replication jumps, the polypurine tracts for the synthesis of the second DNA strand and, except for the isoforms where splicing involved the donor sites located upstream of ORF VII, the primer binding site (PBS) where the cellular Met-tRNA initiator used as primer by the reverse transcriptase binds the 35S RNA [45,46]. Previous data and the present work indicate that some spliced RNAs are indeed reverse transcribed in CaMV-infected cells into double-stranded circular DNA molecules, excluding the above described RNA isoforms deprived of PBS by the splicing event. We characterized DNA molecules having the same sequence as D1-A and D2-A spliced isoforms and deletion mutants resulting from reverse transcription of a spliced RNA have also been observed in plants infected with CaMV-S Japan isolate [17] and in plants infected with FMV [14]. Once produced, it is possible that these deleted genomes reach the nucleus of host cells and participate in the production of spliced-like RNAs upon transcription and artificially increase the pool of spliced RNAs among the global population of viral RNAs. In this sense, it is interesting to note that small circular CaMV DNA molecules of various sizes have been detected through electron microscopy in the nuclei isolated from CaMV-infected turnip plants [47]. Some of these molecules may correspond to reverse transcription products of spliced RNAs. The deleted genomes can also be encapsidated and corresponding viral particles have been isolated from FMV- and CaMV-infected plants [14,17,36]. However, whether these deleted genomes arising from reverse transcription of spliced 35S RNAs play a role upon infection is unknown.

Multiple spliced RNA isoforms arising from single-, double- or triple-splicing events were characterized in CaMV-infected plants. Almost all spliced RNAs contained a new ORF coming from a fusion involving the sORF E, the ORFs I and II or even other reading frames located within ORFs I and II. A complex population of spliced viral transcripts comprising at least 15 spliced isoforms was also characterized in patients chronically infected by hepatitis B virus (HBV) [48,49]. Singly spliced RNAs code for a fusion protein which is involved in the HBV pathogenesis and may participate in the hemostatic abnormality observed in HBV-related liver disease [49,50], whereas the doubly spliced RNAs encode a novel protein that is able to activate transcription [51]. In CaMV, the fused ORF I-ORF II found in some 35S RNA isoforms code for chimeric proteins (P1P2) formed by the N-terminus of P1 and the C-terminus of P2. The function, if any, of these fusion proteins remains unknown and our data indicate that they are dispensable for infectivity in standard growth conditions. It has been hypothesized that these proteins could play a role in the systemic movement of CaMV-infected plants growing under specific conditions, especially at high light intensity [18,52]. Here we show that the fusion protein P1P2(D2) is able to interact *in vitro*, in farwestern experiments, with P2, P3 and purified virions. In the latter case, the interaction is probably mediated by P3, since this protein is associated with viral particles [53]. These interactions, which should operate through the P2 C-terminus [37,38] of the fusion proteins, could hinder the systemic spread and the viral transmission by aphids through competition with interactions involving the full-length P1, P3 and viral particles required for intracellular and cell-to-cell movements of CaMV [40,54], and through prevention of the formation of the viral transmission complex [55]. The very existence of such interactions upon infection is doubtful given that the putative P1P2 proteins were not detected in CaMV-infected plants, using anti-P1 and anti-P2 antibodies in parallel (antibodies directed specifically against the peptide forming the P1P2 junctions were not available). This suggests that the fusion proteins are not expressed from spliced RNAs during CaMV infection or, alternatively, that they are rapidly degraded upon their synthesis *in planta*. This last hypothesis is reinforced by the fact that myc-/HA-tagged or GFP-fused P1P2 proteins were never detected in agroinfiltrated *Nicotiana benthamiana* leaves, whereas their mRNAs were detected by RT-PCR. These proteins might be degraded by the proteasome since analysis of their amino acid sequence did not reveal any instability sequence (PEST) similar to those found in the CaMV P4 protein [56]. In this way, the rapid degradation of P1P2 would prevent putative interactions with P1, P3 or viral particles and so avoid interference with the activities of full-length P1 and P2 proteins. Actually, the present data speak in favor of a structural rather than functional meaning of the multiple fusion ORFs generated upon alternative splicing. In-frame fusions in the 5’ part of the 35S RNA allow both to downregulate expression of ORFs I and II, and to prevent the appearance of multiple ORFs, which could be deleterious for the P6/TAV-mediated translation of the downstream ORFs. Indeed, none of the isoforms we detected contained more ORFs than the unspliced 35S RNA.

Another aim of our study was to understand the functional relevance of 35S RNA alternative splicing in CaMV pathogenesis. We noticed that the splice donor sites D1 and D2 in ORF I are situated within a GGT triplet coding for glycine, even if this amino acid can be specified by four GGN codons. This could hint toward a selective pressure to conserve both the splice sites and the glycine codons (Fig 1B). Glycine residues at these positions are found in the movement protein of all CaMV isolates and two other *Caulimoviruses*, FMV and *Carnation Etched Ring virus* [42]. They evidently play an important role in cell-to-cell movement, since mutations within the donor site leading to their substitution by another amino acid abolish CaMV infectivity (data not shown). We expected that modifications of the alternative splicing scheme would alter the virulence of CaMV on host plants. However, individual or collective mutation of the first described donor sites [18] did not alter the pathogenicity of Cabb B-JI isolate in turnips. Similarly, mutation of CaMV Cabb-S donor sites did not modify the phenotype of infected turnip plants. Apparently, mutation of splice sites stimulates the selection of seldom-used sites and/or activates cryptic splice sites (Fig 4B) to maintain a high level of spliced RNAs in infected tissues and an almost constant ratio between unspliced and spliced 35S RNAs (Fig 4C). The mechanism(s) that preserve(s) a fraction of 35S RNA from splicing and stimulate(s) the use of cryptic sites when the D1, D2 and D4 splice sites are inactivated is still unclear. A similar activation of cryptic splice sites following the inactivation of a donor site has been observed in the CaMV-S isolate [17] and in *Mastrevirus Maize Streak Virus* [10] infections. Paradoxically, while the multiplicity of splice sites in CaMV seems to speak in favor of the possible existence of other roles for 35S RNA alternative splicing outside the suggested downregulation of the expression of aphid transmission factor P2 and movement protein P1 [18,21], the same multiplicity makes a study of any of these roles very difficult to assess. The presence of several splice acceptor sites also explains why mutations disrupting site A of CaMV Cabb B-JI and Cabb-S isolates are not lethal for the virus in our experimental conditions. These observations contradict previous experiments, in which Cabb-S isolate was not infectious in turnip plants when the use of splice acceptor site A was prevented [18], except for two plants which finally displayed symptoms with a three-week delay. Sequencing of the viral genome revealed that the mutated acceptor site was not restored, but a small deletion (58 bp) introducing a premature termination codon in ORF II was found [18]. This deletion did not remove a downstream cryptic splice acceptor site [18] which is also found in the isolate CM4-184, a naturally occurring deletion mutant lacking 461 nucleotides of ORF II [57]. Thus, this splice site might be activated in both the revertant mutant to restore its viability and in CM4-184, since this natural mutant is fully infectious. We observed neither reversion of mutations nor any modification in the ORF II of our mutants upon infection. Therefore, the contradiction between the present observations and the previously published ones [18,21] might be due to different experimental conditions (e.g. the amount of plasmid used for inoculation, turnip plant cultivars or plant growth conditions) since the same Cabb-S isolate was used in both cases. It is not excluded that a mutation was introduced during the construction of the earlier splice acceptor mutant [18,21] and that this has led to a loss of CaMV infectivity. Interestingly, we did not detect any P2 in the ma3 mutant-infected turnip plants, whatever the isolate. This could be due to the glycine to glutamic acid substitution introduced by the mutation since these amino-acids have very different chemical properties and it has already been observed that only one amino-acid substitution can destabilize P2 [37]. In the case of the B-JI mutA mutant, where P2 accumulates at a similar level compared to the wt Cabb B-JI, the modifications induced by the glycine to alanine substitution could not be sufficient to destabilize P2. Our results show that nearby AG dinucleotides can be used as rescue acceptor sites upon disruption of acceptor site A. This suggests that alternative splicing in CaMV achieves strong versatility through multiplicity of splice sites within the 5’ part of 35 S RNA and flexibility in their use, thus constituting a robust means to downregulate expression of ORFs I and II and/or temporally or quantitatively control the expression of ORFs III and IV.

Alternative splicing could have other roles besides downregulating the expression of ORFs I and II. Recently, it was shown in Arabidopsis that spliced RNAs are less effective substrates for RNA silencing mediated by the RNA-dependent RNA polymerase RDR6, compared to intron-less transcripts [58,59]. During CaMV infection, the 35S RNA is subject to degradation by Dicer-like proteins [60–62], but processing of endogenous RDR6-derived double-stranded RNA is impaired, suggesting that introns partially preserve viral RNAs from excessive degradation by the silencing machinery. Furthermore, the splice donor sites located in the leader region (Db and D4) might be involved in the suppression of premature polyadenylation at the 5' end of the 35S RNA, as described for *Retroviridae* HIV-1 [63,64] and foamy viruses [65]. The U1 small nuclear ribonucleoprotein (U1 snRNP), in addition to its role in the splicing process, interferes with the pre-mRNA 3’ end processing and protects some pre-mRNAs from premature polyadenylation [66]. This regulatory mechanism ensures transcriptome integrity by regulating mRNA length [67]. In HIV-1 and foamy viruses, the polyadenylation signal (poly(A) signal) is found at both extremities of the viral progenome, in the redundant 5’ and 3’ long terminal repeats (LTRs). It has been shown that the poly(A) signal at the 5’ LTR is skipped through a mechanism involving the U1 snRNP-mediated recognition of a splice donor site located a few hundred nucleotides upstream (HIV-1) [63] or downstream (foamy viruses) [65], thus avoiding a premature termination of transcription. The CaMV poly(A) signal is located about 180 nucleotides downstream of the transcription start site, so polyadenylation needs to be regulated to allow transcription of the complete genome. The Db and/or D4 donor sites are about 300 nucleotides downstream of the poly(A) signal. The putative role of D4 and Db in the regulation of CaMV premature polyadenylation should therefore be assessed in the future.

The splice donor and acceptor sites described in the present study allow CaMV to produce multiple spliced 35S RNAs, which do not encode for P1 and P2 proteins while still being appropriate mRNAs for P6/TAV-mediated expression of viral proteins via translation reinitiation. Any inactivation of a splice site would be ineffective in impairing CaMV infectivity. The synthesis of intron-deleted DNA by reverse transcription could be a strategy to increase the population of spliced RNAs in a spliceosome-independent fashion. Whether CaMV alternative splicing is regulated by *cis*-elements (splicing enhancers and silencers) is another point which must be elucidated in the future, to gain insight on the temporal regulation and the biological relevance of the various 35S RNA spliced isoforms.

## Supporting Information

S1 Fig P1P2(D2) fusion protein can interact with viral proteins *in vitro*. Far-western assays on GST or GST-P1P2(D2) expressed in *E*. *coli*. Recombinant proteins were submitted to SDS-PAGE and transferred to PVDF membranes. ^32^P-labeled recombinant P1, P2, P3 and TAV proteins or purified virions were used as overlays.
(TIF)

S2 Fig Detection of *E*. *coli*-expressed P1P2(D2) fusion protein by western-blot. Recombinant GST, GST-P1P2(D2), P1P2(D2) fusion protein and non-transformed *E*. *coli* crude extract were separated by SDS-PAGE and transferred to PVDF membranes. Polyclonal anti-P1 and anti-P2 antibodies were used as primary antibodies.
(TIF)

S3 Fig Viral proteins P2 and P4 accumulate at similar levels in D1D2D4Da mutant and wt Cabb-B-JI-infected turnip plants. Proteins were extracted from systemic leaves at 21 dpi and analyzed by western-blot. Polyclonal anti-P4 and anti-P2 antibodies were used as primary antibodies.
(TIF)

S4 Fig RT-PCRs covering the full 35S RNA do not reveal any splicing events in its 3’ part. (A) Schematic representation indicating the regions amplified by RT-PCR. *A*: from positions 164 to 2404, *B*: from positions 844 to 3234, *C*: from positions 1704 to 4204, *D*: from positions 2544 to 4974, *E*: from positions 3404 to 5384, *F*: from positions 4204 to 6404, *G*: from positions 4904 to 7434, *H*: from positions 6404 to 794. (B) RT-PCR products were separated on agarose gel. The control lanes correspond to PCRs performed on viral vectors, using the same primers. Primers targeting region *H* are designed to specifically amplify contaminating viral DNA.
(TIF)

S1 Table Sequenced spliced isoforms from RT-PCR amplicons. The 5’ part of the 35S RNA from CaMV Cabb B-JI- or Cabb-S-infected turnip plants was amplified by RT-PCR. Amplicons were fractionated on agarose gel, extracted and cloned into pGEM-T Easy vector. In order to maximize the diversity of the sequenced spliced isoforms, inserted 35S RNA fragments were selected prior to sequencing (see “Materials and Methods”).
(TIF)

S1 Text Splice sites are conserved among 67 recently sequenced isolates. Nucleotides corresponding to donor and acceptor sites of Cabb-S isolate are in bold. Donor sites D1 and D2 are in page 17, the donor site D3 is in page 23 and the acceptor site A is in page 27.
(DOCX)
