# Supplementary figures and images for: Cauliflower mosaic virus Transcriptome Reveals a Complex Alternative Splicing Pattern
Source: PLoS One. 2015 Jul 10;10(7):e0132665. doi: 10.1371/journal.pone.0132665 (PMC4498817; doi:10.1371/journal.pone.0132665)

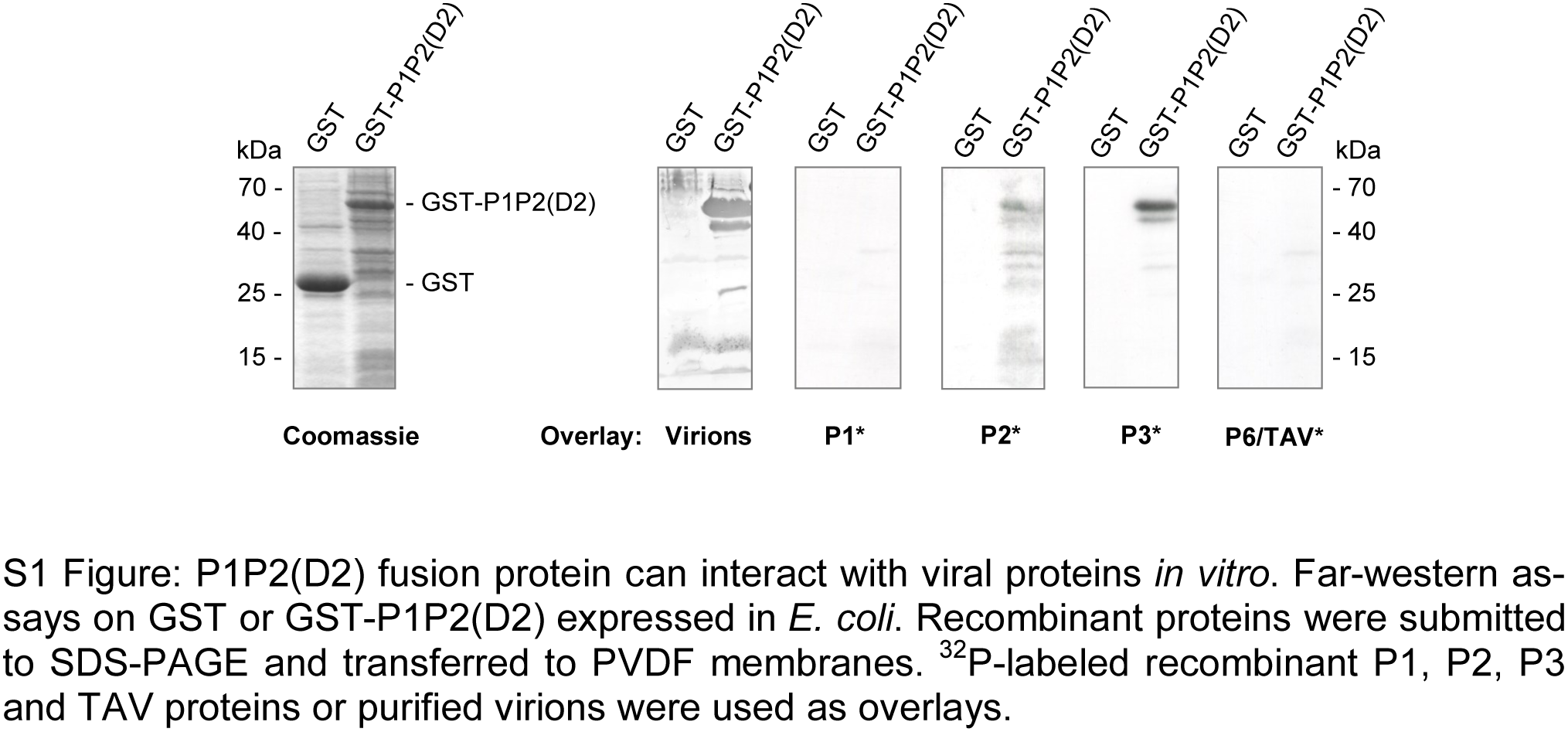

Supplement: S1 Fig — Far-western assays on GST or GST-P1P2(D2) expressed in E. coli. Recombinant proteins were submitted to SDS-PAGE and transferred to PVDF membranes. 32P-labeled recombinant P1, P2, P3 and TAV proteins or purified virions were used as overlays. (TIF) [file pone.0132665.s001.tif]

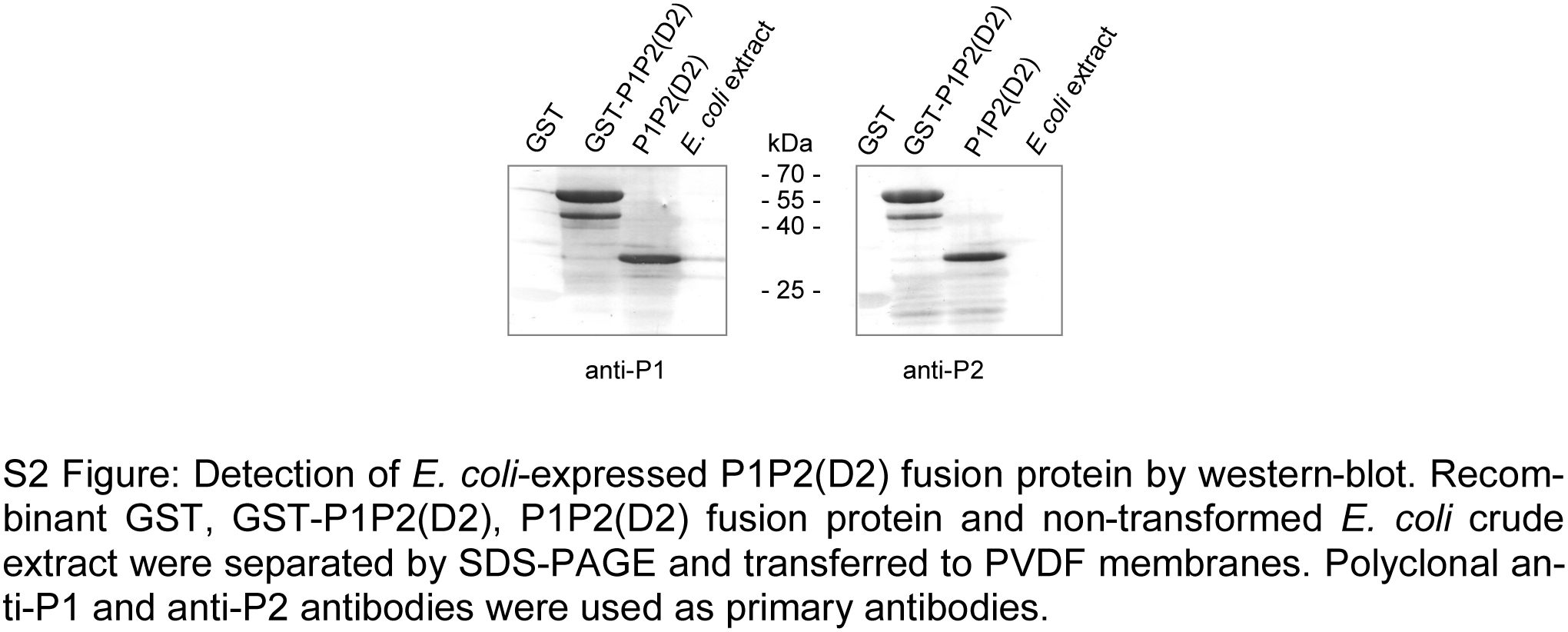

Supplement: S2 Fig — Recombinant GST, GST-P1P2(D2), P1P2(D2) fusion protein and non-transformed E. coli crude extract were separated by SDS-PAGE and transferred to PVDF membranes. Polyclonal anti-P1 and anti-P2 antibodies were used as primary antibodies. (TIF) [file pone.0132665.s002.tif]

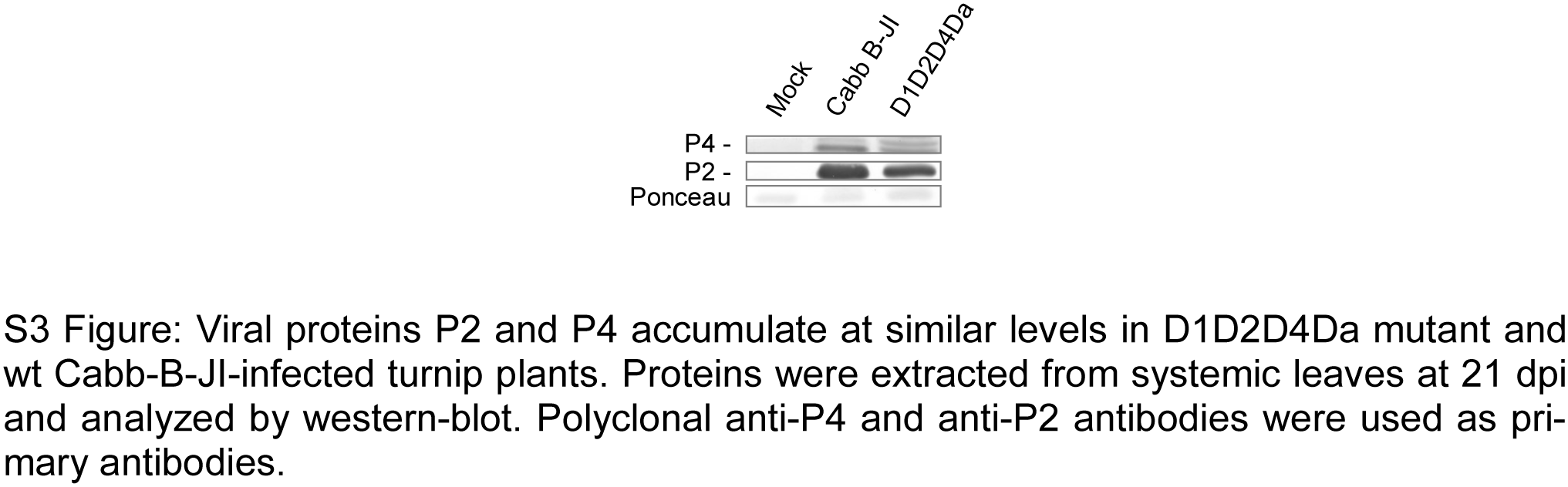

Supplement: S3 Fig — Proteins were extracted from systemic leaves at 21 dpi and analyzed by western-blot. Polyclonal anti-P4 and anti-P2 antibodies were used as primary antibodies. (TIF) [file pone.0132665.s003.tif]

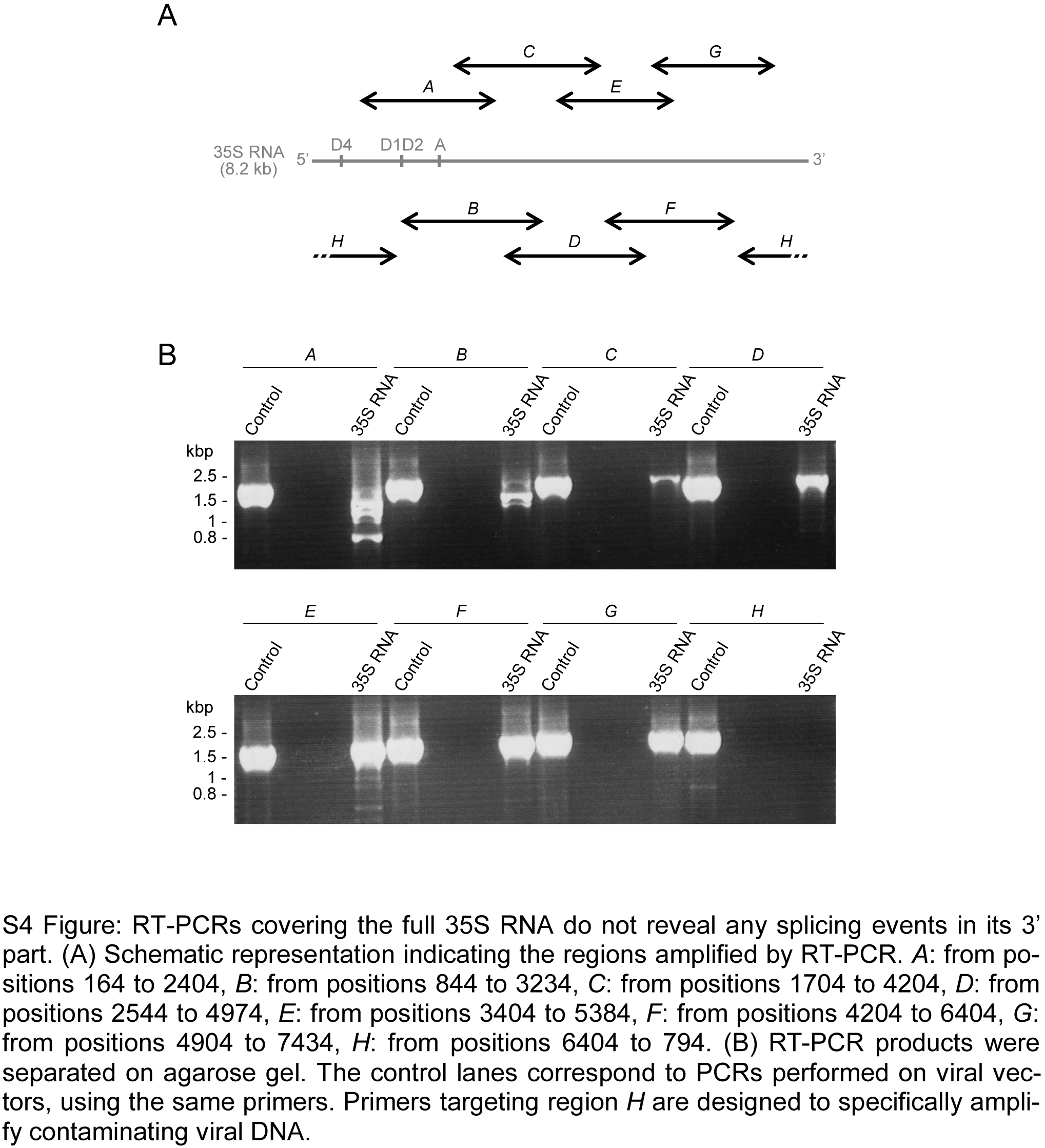

Supplement: S4 Fig — (A) Schematic representation indicating the regions amplified by RT-PCR. A: from positions 164 to 2404, B: from positions 844 to 3234, C: from positions 1704 to 4204, D: from positions 2544 to 4974, E: from positions 3404 to 5384, F: from positions 4204 to 6404, G: from positions 4904 to 7434, H: from positions 6404 to 794. (B) RT-PCR products were separated on agarose gel. The control lanes correspond to PCRs performed on viral vectors, using the same primers. Primers targeting region H are designed to specifically amplify contaminating viral DNA. (TIF) [file pone.0132665.s004.tif]

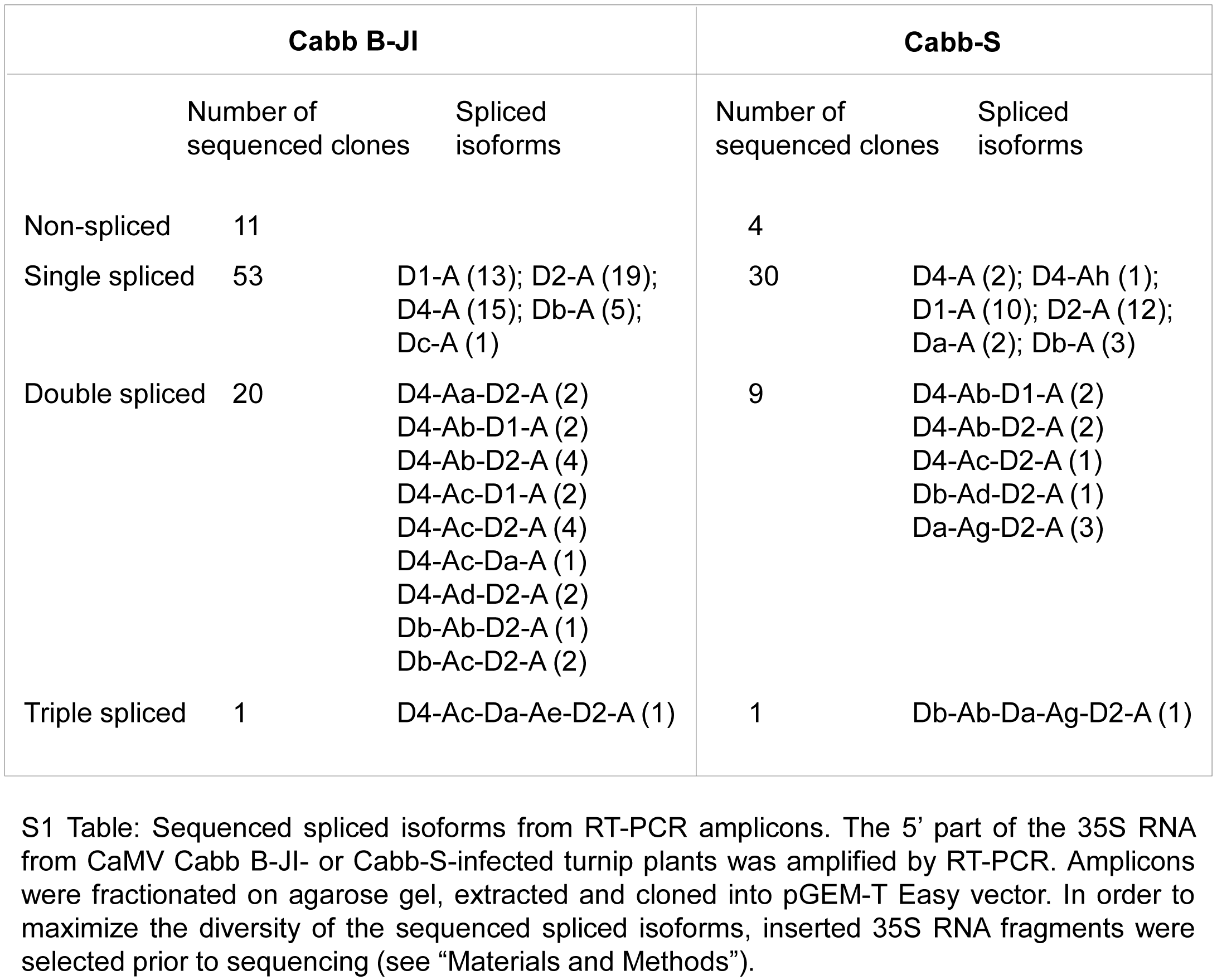

Supplement: S1 Table — The 5’ part of the 35S RNA from CaMV Cabb B-JI- or Cabb-S-infected turnip plants was amplified by RT-PCR. Amplicons were fractionated on agarose gel, extracted and cloned into pGEM-T Easy vector. In order to maximize the diversity of the sequenced spliced isoforms, inserted 35S RNA fragments were selected prior to sequencing (see “Materials and Methods”). (TIF) [file pone.0132665.s005.tif]
