# Supplementary material for: Cauliflower mosaic virus Transcriptome Reveals a Complex Alternative Splicing Pattern
Source: PLoS One. 2015 Jul 10;10(7):e0132665. doi: 10.1371/journal.pone.0132665 (PMC4498817; doi:10.1371/journal.pone.0132665)
Supplement: S1 Text — Nucleotides corresponding to donor and acceptor sites of Cabb-S isolate are in bold. Donor sites D1 and D2 are in page 17, the donor site D3 is in page 23 and the acceptor site A is in page 27. (DOCX) [file pone.0132665.s006.docx]

S1 Text: Splice sites are conserved among 67 recently sequenced isolates. Nucleotides corresponding to donor and acceptor sites of Cabb-S isolate are in bold. Donor sites D1 and D2 are in page 17, the donor site D3 is in page 23 and the acceptor site A is in page 27.

Cabb-S|NC_001497.1| --------------------------------------------GGTATCAGAGCCATGA

CRO180A|AB863192.1| --------------------------------------------GGTATCAGAGCCATGA

GRC83|AB863193.1| --------------------------------------------GGTATCAGAGCCATGA

GRC84B|AB863194.1| --------------------------------------------GGTATCAGAGCCATGA

GRC86B|AB863195.1| --------------------------------------------GGTATCAGAGCCATGA

GRC86D|AB863196.1| --------------------------------------------GGTATCAGAGCCATGA

GRC87E|AB863197.1| --------------------------------------------GGTATCAGAGCCATGA

GRC87G|AB863198.1| --------------------------------------------GGTATCAGAGCCATGA

GRC91B|AB863199.1| --------------------------------------------GGTATCAGAGCCATGA

GRC92A|AB863200.1| --------------------------------------------GGTATCAGAGCCATGA

GRC92C|AB863201.1| --------------------------------------------GGTATCAGAGCCATGA

GRC92D|AB863202.1| --------------------------------------------GGTATCAGAGCCATGA

IRN1|AB863136.1| --------------------------------------------GGTATCAGAGCCATGA

IRN2|AB863137.1| --------------------------------------------GGTATCAGAGCCATGA

IRN3|AB863138.1| --------------------------------------------GGTATCAGAGCCATGA

IRN4|AB863139.1| --------------------------------------------GGTATCAGAGCCATGA

IRN5|AB863140.1| --------------------------------------------GGTATCAGAGCCATGA

IRN6|AB863141.1| --------------------------------------------GGTATCAGAGCCATGA

IRN7|AB863142.1| --------------------------------------------GGTATCAGAGCCATGA

IRN8|AB863143.1| --------------------------------------------GGTATCAGAGCCATGA

IRN9|AB863144.1| --------------------------------------------GGTATCAGAGCCATGA

IRN10|AB863145.1| --------------------------------------------GGTATCAGAGCCATGA

IRN11|AB863146.1| --------------------------------------------GGTATCAGAGCCATGA

IRN12|AB863147.1| --------------------------------------------GGTATCAGAGCCATGA

IRN13|AB863148.1| --------------------------------------------GGTATCAGAGCCATGA

IRN14|AB863149.1| --------------------------------------------GGTATCAGAGCCATGA

IRN15|AB863150.1| --------------------------------------------GGTATCAGAGCCATGA

IRN16|AB863151.1| --------------------------------------------GGTATCAGAGCCATGA

IRN17|AB863152.1| --------------------------------------------GGTATCAGAGCCATGA

IRN18|AB863153.1| --------------------------------------------GGTATCAGAGCCATGA

IRN19|AB863154.1| --------------------------------------------GGTATCAGAGCCATGA

IRN20|AB863155.1| --------------------------------------------GGTATCAGAGCCATGA

IRN21|AB863156.1| --------------------------------------------GGTATCAGAGCCATGA

JPNHGB340|AB863157.1| --------------------------------------------GGTATCAGAGCCATGA

JPNKWB778|AB863158.1| --------------------------------------------GGTATCAGAGCCATGA

JPNM|AB863159.1| --------------------------------------------GGTATCAGAGCCATGA

JPNN|AB863160.1| -------------GGTATCATTGCTTATCAGTGTTTAAAAAATTGGTATCAGAGCCATGA

JPNS1|AB863161.1| --------------------------------------------GGTATCAGAGCCATGA

JPNS2|AB863162.1| GGTATCAGAGCATGGTATTATAGCAATACCATGTATAAAAAATTGGTATCAGAGCCATGA

JPNTKD762|AB863163.1| --------------------------------------------GGTATCAGAGCCATGA

JPNUV1|AB863164.1| --------------------------------------------GGTATCAGAGCCATGA

JPNUV26|AB863165.1| --------------------------------------------GGTATCAGAGCCATGA

TUR1|AB863166.1| --------------------------------------------GGTATCAGAGCCATGA

TUR2|AB863167.1| --------------------------------------------GGTATCAGAGCCATGA

TUR4|AB863168.1| --------------------------------------------GGTATCAGAGCCATGA

TUR5|AB863169.1| --------------------------------------------GGTATCAGAGCCATGA

TUR12|AB863170.1| --------------------------------------------GGTATCAGAGCCATGA

TUR34|AB863171.1| --------------------------------------------GGTATCAGAGCCATGA

TUR50|AB863172.1| --------------------------------------------GGTATCAGAGCCATGA

TUR59|AB863173.1| --------------------------------------------GGTATCAGAGCCATGA

TUR69|AB863174.1| --------------------------------------------GGTATCAGAGCCATGA

TUR81|AB863175.1| --------------------------------------------GGTATCAGAGCCATGA

TUR84|AB863176.1| --------------------------------------------GGTATCAGAGCCATGA

TUR94|AB863177.1| --------------------------------------------GGTATCAGAGCCATGA

TUR213|AB863178.1| --------------------------------------------GGTATCAGAGCCATGA

TUR214|AB863179.1| --------------------------------------------GGTATCAGAGCCATGA

TUR216|AB863180.1| --------------------------------------------GGTATCAGAGCCATGA

TUR220|AB863181.1| --------------------------------------------GGTATCAGAGCCATGA

TUR239|AB863182.1| --------------------------------------------GGTATCAGAGCCATGA

TUR244|AB863183.1| --------------------------------------------GGTATCAGAGCCATGA

TUR249|AB863184.1| --------------------------------------------GGTATCAGAGCCATGA

TUR263|AB863185.1| --------------------------------------------GGTATCAGAGCCATGA

TUR278|AB863186.1| --------------------------------------------GGTATCAGAGCCATGA

TUR279|AB863187.1| --------------------------------------------GGTATCAGAGCCATGA

TUR285|AB863188.1| --------------------------------------------GGTATCAGAGCCATGA

TUR289|AB863189.1| --------------------------------------------GGTATCAGAGCCATGA

TUR303|AB863190.1| --------------------------------------------GGTATCAGAGCCATGA

TUR306|AB863191.1| --------------------------------------------GGTATCAGAGCCATGG

Cabb-S|NC_001497.1| ATCGGTTTAAGACCAAAACTCAAGAGGGTAAAACCTCACCAAAATACGAAAGAGTTCTTA

CRO180A|AB863192.1| ATAGGTCTATGACCAAAACTCAAGAGGATAAAACCTCATCAAAATACCAAAGAGTTCTTA

GRC83|AB863193.1| ATAGGTCTAAGACCATAACTCAAGAGGGTAAAACCTCATCAAAATACCAAAGAGTTCTTA

GRC84B|AB863194.1| ATAGGTCTAAGACCATAACTCAAGAGGGTAAAACCTCATCAAAATACCAAAGAGTTCTTA

GRC86B|AB863195.1| ATAGGTCTAAGACCATAACTCAAGAGGGTAAAACCTCATCAAAATATCAAAGAGTTCTTA

GRC86D|AB863196.1| ATAGGTCTAAGACCATAACTCAAGAGGGTAAAACCTCACCAATCAATCAAAGAGTTCTTA

GRC87E|AB863197.1| ATAGGTCTAAGACCATAACTCAAGAGGGTAAAACCTCATCAATTAATCAAAGAGTTCTTA

GRC87G|AB863198.1| ATAGGTCTAAGACCATAACTCAAGAGGGTAAAACCTCATCAATTAATCAAAGAGTTCTTA

GRC91B|AB863199.1| ATAGGTCTAAGACCATAACTCAAGAGGGTAAAACCTCATCAATCAGTCAAAGAGTTCTTA

GRC92A|AB863200.1| ATAGGTCTATGACCAAAACTCAAGAGGGTAAAACCTCATCAATCAATCAAAGAATTCTTA

GRC92C|AB863201.1| ATAGGTCTATGACCAAAACTCAAGAGGGTAAAACCTCATCAATCAATCAAAGAGTTCTTA

GRC92D|AB863202.1| ATAGGTCTAAGACCATAACTCAAGAGGGTAAAACCTCATCAACCAATCAAAGAGTTCTTA

IRN1|AB863136.1| ATAGGTCTAAGACCATAACTCAAGAGGGTAAAACCTCATCAAAATACCAAAGAGTTCTTA

IRN2|AB863137.1| ATAGGTCTAAGACCATAACTCAAGAGGGTAAAACCTCATCAAAATACCAAAGAGTTCTTA

IRN3|AB863138.1| ATAGGTCTAAGACCATAACTCAAGAGGGTAAAACCTCATCAAAATACCAAAGAGTTCTTA

IRN4|AB863139.1| ATAGGTCTAAGACCATAACTCAAGAGGGTAAAACCTCATCAAAATACCAAAGAGTTCTTA

IRN5|AB863140.1| CTAGGTCTAAGACCATAACTCAAGAGGGTAAAACCTCATCAAAATACCAAAGAGTTCTTA

IRN6|AB863141.1| ATAGGTCTAAGACCATAACTCAAGAGGGTAAAACCTCATCAAAATACCAAAGAGTTCTTA

IRN7|AB863142.1| ATAGGTCTAAGACCATAACTCAAGAGGGTAAAACCTCATCAAAATACCAAAGAGTTCTTA

IRN8|AB863143.1| ATAGGTCTAAGACCATAACTCAAGAGGGTAAAACCTCATCAAAATACCAAAGAGTTCTTA

IRN9|AB863144.1| ATAGGTCTAAGACCATAACTCAAGAGGGTAAAACCTCATCAAAATACCAAAGAGTTCTTA

IRN10|AB863145.1| ATAGGTCTAAGACCACAACTCAAGAGGATAAAACCTCATCAAAATACCAAAGAGTTCTTA

IRN11|AB863146.1| ATAGGTCTAAGACCATAACTCAAGAGGATAAAACCTCATCAAAATACCAAAGAGTTCTTA

IRN12|AB863147.1| ATAGGTCTAAGACCATAACTCAAGAGGGTAAAACCTCATCAAAATACCAAAGAGTTCTTA

IRN13|AB863148.1| ATAGGTCTAAGACCATAACTCAAGAGGGTAAAACCTCATCAAAATACCAAAGAGTTCTTA

IRN14|AB863149.1| ATAGGTCTAAGACCATAACTCAAGAGGGTAAAACCTCATCAAAATACCAAAGAGTTCTTA

IRN15|AB863150.1| ATAGGTCTAAGACCATAACTCAAGAGGGTAAAACCTCATCAAAATACCAAAGAGTTCTTA

IRN16|AB863151.1| ATAGGTCTAAGACCTTAACTCAAGAGGGTAAAACCTCATCAAAATACCAAAGAGTTCTTA

IRN17|AB863152.1| ATAGGTCTAAGACCATAACTCAAGAGGGTAAAACCTCATCAAAATACCAAAGAGTTCTTA

IRN18|AB863153.1| ATAGGTCTAAGACCATAACTCAAGAGGGTAAAACCTCACCAAAATACCAAAGAGTTCTTA

IRN19|AB863154.1| ATAGGTCTAAGACCATAACTCAAGAGGGTAAAACCTCATCAAAATACCAAAGAGTTCTTA

IRN20|AB863155.1| ATAGGTCTAAGACCATAACTCAAGAGGGTAAAACCTCATCAAAATACCAAAGAGTTCTTA

IRN21|AB863156.1| ATAGGTCTAAGACCATAACTCAAGAGGGTAAAACCTCATTAAAATACCAAAGAGTTCTTA

JPNHGB340|AB863157.1| ATAGGTCTATAACCAAAACTCAAGAGGATAAAACCTCACCAAAATACGAAAGAGTTCTTA

JPNKWB778|AB863158.1| ATAGGTCTATGACCAAAACTCAAGAGGATAAAACCTCATCAAAATACGAAAGAGTTCTTA

JPNM|AB863159.1| ATAGGTCTATGACCAAAACTCAAGAGGATAAATCCTCACCAAAATACGAAAGAGTTCTTA

JPNN|AB863160.1| ATCGGTTTAACGACCAAACTCTAGAGGATAAAACCTCACCAAAAAACCAAAGAGTTCTTA

JPNS1|AB863161.1| ATCGGTTTAAAAACCAAACTCTAGAGGATAAAACCTCACCAAAAAACCAAAGAGTTCTTA

JPNS2|AB863162.1| ATCGGTTTAAAAACCAAACTCTAGAGGATAAAACCTCACCAAAAAACCAAAGAGTTCTTA

JPNTKD762|AB863163.1| ATAGGTCTATGACCAAAACTCAAGAGGATAAAACCTCACCAAAATACGAAAGAGTTCTTA

JPNUV1|AB863164.1| ATAGGTCTATGACCAAAACTCAAGAGGATAAATCCTCACCAAAATACGAAAGAGTTCTTA

JPNUV26|AB863165.1| ATAGGTCTATGACCAAAACTCAAGAGGATAAATCCTCACCAAAATACGAAAGAGTTCTTA

TUR1|AB863166.1| ATAGGTCTAAGACCATAACTCAAGAGGGTAAAACCTCATCAAAATATGAAAGAGTTCTTA

TUR2|AB863167.1| ATAGGTCTAAGACCATAACTCAAGAGGATAAAACCTCATCAAAATACCAAAGAGTTCTTA

TUR4|AB863168.1| ATAGGTCTAAGACCATAACTCAAGAGGATAAAACCTCATCAAAATACCAAAGAGTTCTTA

TUR5|AB863169.1| ATAGGTCTAAGACCATAACTCAAGAGGGTAAAACCTCATCAAAATACCAAAGAGTTCTTA

TUR12|AB863170.1| ATAGGTCTAAGACCACAACTCAAGAGGGAAAAACCTCATCAAAATACCAAAGAGTTCTTA

TUR34|AB863171.1| ATAGGTTTAAGACCATAACTCAAGAGGGTAAAACCTCATCAAAATACCAAAGAGTCCTTA

TUR50|AB863172.1| ATAGGTCTAAGACCATAACTCAAGAGGATAAAACCTCATCAAAATACCAAAGAGTTCTTA

TUR59|AB863173.1| ATAGGTCTAAGACCATAACTCAAGAGGATAAAACCTCATCAAAATACCAAAGAGTTCTTA

TUR69|AB863174.1| ATAGGTCTAAGACTATAACTCAAGAGGGTAAAACCTCATCAAAACACCAAAGAGTTCCTA

TUR81|AB863175.1| ATAGGTCTAAGACCA------TAGAGGATAAAACCTCATCAAAATACCAAAGAGTTCTTA

TUR84|AB863176.1| ATAGGTCTAAGACCATAACCAAAGAGGATAAAACCTCATCAAAATACCAAAGAGTTCTTA

TUR94|AB863177.1| ATAGGTCTATGACCAAAACTCAAGAGGATAAAACCTCATCAAAATACCAAAGAGTTCTTA

TUR213|AB863178.1| ATAGGTCTAAGACCATAACTCAAGAGGGTAAAACCTCATCAAAATATGAAAGAGTTCTTA

TUR214|AB863179.1| ATAGGTCTAAGACCATAACTCAAGAGGGTAAAATCTCATCAAAAAACCAAAGAGTTCTTA

TUR216|AB863180.1| ATCGGTTTAAGACCAAAACTCAAGAGAGTAAAACCTCATCAATAAACCAAAGAGTTCTTA

TUR220|AB863181.1| ATAGGTCTAAGACCATAACCAAAGAGGATAAAACCTCATCAAAATACCAAAGAGTTCTTA

TUR239|AB863182.1| ATAGGTCTAAGACCATAACTCAAGAGGGTAAAACCTCATCAAAAAACCAAAGAGTTCTTA

TUR244|AB863183.1| ATAGGTCTAAGACCATAACTCAAGAGGGTAAAACCTCATCAAAATACCAAAGAGTTCTTA

TUR249|AB863184.1| ATAGGTCTAAGACCATAACTCAAGAGGGTAAAACCTCATCAAAATACAAAAGAGTTCTTA

TUR263|AB863185.1| ATAGGTCTAAGACCATAACTCAAGAGGATAAAACCTCATCAAAATACCAAAGAGTTCTTA

TUR278|AB863186.1| ATAGGTCTAAGACCATAACTCAAGAGGGTAAAACCTCATCAAAATGCCAAAGAGTTCTTA

TUR279|AB863187.1| ATAGGTCTAAGACCATAACCAAAGAGGATAAAACCTCATCAAAATACCAAAGAGTTCTTA

TUR285|AB863188.1| ATAGGTCTATGACCAAAACTCAAGAGGATAAAACCTCATCAAAATACCAAAGAGTTCTTA

TUR289|AB863189.1| ATAGGTCTAAGACCATAACTCAAGAGGGTAAAACCTCATCAAAATACCAAAGAGTTCTTA

TUR303|AB863190.1| ATAGGTCTAAGACCATAACTCAAGAGGGTAAAACCTCATCAAAATACGAAAGAGTTCTTA

TUR306|AB863191.1| ACAGGTCTAAGACCATAACTCAAGAGGGTAAAACCTCATCAAAATACCAAAGAGTTCTTA

Cabb-S|NC_001497.1| ACTCTAAAAATAAAAGATCTTTCAAGATCAAACATAGTTCCCTCACACCGGTGACCGACA

CRO180A|AB863192.1| ACTCTAAAGATAAAAGATCTTTCAAGATCAAAAATAGTTCCCTCACACCGGTGACCGACA

GRC83|AB863193.1| ACTCTAAAGATAAACGATCTGTTTGGATCGAAACTAGTTCCTCGACAACGGGTTGCGACA

GRC84B|AB863194.1| ACTCTAAAGATAAACGATCTGTTTGGATCGAAACTAGTTCCTCGACAACGGGTTGCGACA

GRC86B|AB863195.1| ACTCTAAAATTAAACGATCTGTTTGGATCGAAACTAGTTCCTCAGCAACGGGTTGCGACA

GRC86D|AB863196.1| ACTCTAAAATTAAACGATCTGTTTGGATCGAAACTAGTTCCTCAGCAACGGGTTGCGACA

GRC87E|AB863197.1| ACTCTAAAATTAAACGATCTGTTTGGATCGAAACTAGTTCCTCAGCAACGGGTTGCGACA

GRC87G|AB863198.1| ACTCTAAAATTAAACGATCTGTTTGGATCGAAACTAGTTCCTCAGCAACGGGTTGCGACA

GRC91B|AB863199.1| ACTCTAAAATTAAACGATCTGTTTGGATCGAAACTAGTTCCTCAGCAACGGGTTGCGACA

GRC92A|AB863200.1| ACTCTAAAATTAAACGATCTGTTTGGATCGAAACTAGTTCCTCAGCAACGGGTTGCGACA

GRC92C|AB863201.1| ACTCTAAAGATAAACGATCTGTTTGGATCGAAACTAGTTCCTCGACAACGGGTTGCGACA

GRC92D|AB863202.1| ACTCTAAAATTAAACGATCTGTTTGGATCGAAACTAGTTCCTCAGCAACGGGTTGCGACA

IRN1|AB863136.1| ACTCTAAAGATAAAAGATCCTTCAAGATCAAAAATAGTTCCCTCACACCGGTGACCGACA

IRN2|AB863137.1| ACTCTAAAGATAAAAGATCTTTCAAGATCAAAAATAGTTCCCTCACACCGGTGACCGACA

IRN3|AB863138.1| ACTCTAAAGATAAAAGATCCTTCAAGATCAAAAATAGTTCCCTCACACCGGTGACCGACA

IRN4|AB863139.1| ACTCTAAAGATAAAAGATCTTTCAAGATCAAACATAGTTCTCTCACACCGGTGACCGACA

IRN5|AB863140.1| ACTCTAAAGATAAAAGATCTTTCAAGATCAAACATAGTTCCCTCACACCGGTGACCGACA

IRN6|AB863141.1| ACTCTAAAGATAAAAGATCTTTCAAGATCAAAAATAGTTCCCTCACACCGGTGACCGACA

IRN7|AB863142.1| ACTCTAAAAATAAAAGATCTTTCAAGATCAAACATAGTTCCCTCACACCGGTGACCGACA

IRN8|AB863143.1| ACTCTAAAGATAAAAGATCTTTTAAGATCAAACATAGTTCCCTCACACCGGTGACCGACA

IRN9|AB863144.1| ACTCTAAATATAAAAGATCTTTCAAGATCAAACATAGTTCCCTCACACCGGTGACCGACA

IRN10|AB863145.1| ACTCTAAAGATAAAAGATCTTTCAAGATCAAACATAGTTCCCTCACACCGGTGACCGACA

IRN11|AB863146.1| ACTCTAAAGATAAAAGATCTTTCAAGATCAAAAATAGTTCCCTCACACCGGTGACCGACA

IRN12|AB863147.1| ACTCTAAAGATAAAAGATCTTTCAAGATCAAGCATAGTTCCCTCACACCGGTGACCGACA

IRN13|AB863148.1| ACTCTAAAGATAAAAGATCTTTCAAGATCAAAAATAGTTCCCTCACACCGGTGACCGACA

IRN14|AB863149.1| ACTCTAAAGATAAGAGATCTTTCAAGATCAAACATAGTTCCCTCACACCGGTGACCGACA

IRN15|AB863150.1| ACTCTAAAGATAAAAGATCTTTCAAGATCAAACATAGTTCCCTCACACCGGTGACCGACA

IRN16|AB863151.1| ACTCTAAAGATAAAAGATCTTTCAAGATCAAATATAGTTCCCTCACACCGGTGACCGACA

IRN17|AB863152.1| ACTCTAAAGATAAAAGATCTTTCAAGATCAAACATAGTTCCCTCACACCGGTGACCGACA

IRN18|AB863153.1| ACTCTAAATATAAAAGATCTTTCAAGATCAAAACTAGTTCCCTCACACCGGTGACCGACA

IRN19|AB863154.1| ACTCTAAAGATAAAAGATCTTTCAAGATCAAACATAGTTCCCTCACACCGGTGACCGACA

IRN20|AB863155.1| ACTCTAAAGATAAAAGATCTTTCAAGATCAAACATAGTTCCCTCACTCCGGTGACCGACA

IRN21|AB863156.1| ACTCTAAAGATAAAAGATCTTTCAAGATCAAAAATAGTTCCCTCACACCGGTGACCGACA

JPNHGB340|AB863157.1| ACTCTAAAGATAAAAGATCTTTTAAGATCAAAACTAGTTCCCTCACACCGGTGACCGACA

JPNKWB778|AB863158.1| ACTCTAAAGATAAAAGATCTTTCAAGATCAAAACTAGTTCCCTCACACCGGTGACCGACA

JPNM|AB863159.1| ACTCTAAAGGAAAAAGATCTTTCAAGATCAAAACTAGTTCCCTCACACCGGTGACCGACA

JPNN|AB863160.1| ACTCTAAAGATAAAAGATCTTTCAAGATCAACAATAGTTCCCTCACACCGATGGCCGACA

JPNS1|AB863161.1| ACTCTAAAGATAAAAGATCTTTCAAGATCAACAATAGTTCCCTCACACCGATGGCCGACA

JPNS2|AB863162.1| ACTCTAAAGATAAAAGATCTTTCAAGATCAACAATAGTTCCCTCACACCGATGGCCGACA

JPNTKD762|AB863163.1| ACTCTAAAGATAAAAGATCTTTCAAGATCAAAACTAGTTCCCTCACACCGGTGACCGACA

JPNUV1|AB863164.1| ACTCTAAAGGTAAAAGATCTTTCAAGATCAAAACTAGTTCCCTCACACCGGTGACCGACA

JPNUV26|AB863165.1| ACTCTAAAGGTAAAAGATCTTTCAAGATCAAAACTAGTTCCCTCACACCGGTGACCGACA

TUR1|AB863166.1| ACTCTAAAGATAAAAGATCTTTCAAGATCAAAATTAGTTCCCTCACACCGGTGACCGACA

TUR2|AB863167.1| ACTCTAAAGATAAAAGATCTTTCAAGATCAAACATAGTTCCCTCACACCGGTGACCGACA

TUR4|AB863168.1| ACTCTAAAGATAAAAGATCTTTCAAGATCAAACATAGTTCCCTCACACCGGTGACCGACA

TUR5|AB863169.1| ACTCTAAAGATAAAAGATCTTTCAAGATCAAAATTAGTTCCCTCACACCGGTGACCGACA

TUR12|AB863170.1| ACTCTAAAAATAAAAGATCTTTCAAGATCAAAACTAGTTCCCTCACATCGGTGACCGACA

TUR34|AB863171.1| ACTCTAAAGATAAAAGATCTTTCAAGATCAAAAATAGTTCCCTCACACCGGTGACCGACA

TUR50|AB863172.1| ACTCTAAATATAAAAGATCTTTCAAGATCAAAAATAGTTCCCTCACACCGGTGACCGACA

TUR59|AB863173.1| ACTCTAAAGATAAACGATCTTTCAAGATCAAAACTAGTTCCCTCACACCGGTGACCGACA

TUR69|AB863174.1| ACTCTAAAGACAAAAGATCTTTCAAGATCAAACATAGTTCCCTCACACCGGTGACCGACA

TUR81|AB863175.1| ACTCTAAAGATAAAAGATCTTTCAAGATCAAACATAGTTCCCTCACACCGGTGACCGACA

TUR84|AB863176.1| ACTCTAAAGATAAAAGATCTTTCAAGATCAAACATAGTTCCCTCACACCGGTGACCGACA

TUR94|AB863177.1| ACTCTAAAGATAAAAGATCTTTCAAGATCAAAATTAGTTCCCTCACACCGGTGACCGACA

TUR213|AB863178.1| ACTCTAAAGATAAAAGATCTTTCAAGATCAAAATTAGTTCCCTCACACCGGTGACCGACA

TUR214|AB863179.1| ACTCTAAAGATAAAAGATCTTTCAAGATCAAAAATAGTTCCCTCACACCGGTGACCGACA

TUR216|AB863180.1| ACTCTAAAAATAAAAGATCTTTCAAGATCAAAACTAGTTCCTCAGCAACGGGTTGCGACA

TUR220|AB863181.1| ACTCTAAAGATAAAAGATCTTTCAAGATCAAACATAGTTCCCTCACACCGGTGACCGACA

TUR239|AB863182.1| ACTCTAAAGATAAAAGATCTTTCAAGATCAAACATAGTTCCCTCACACCGGTGACCGACA

TUR244|AB863183.1| ACTCTAAAGATAAAAGATCTTTCAAGATCAAACATAGTTCCCTCACACCGGTGACCGACA

TUR249|AB863184.1| ATTCTAAAGATAAAAGATCTTTCAAGATCAAAAATAGTTCCCTCACACCGGTGACCGACA

TUR263|AB863185.1| ACTCTAAAGATAAAAGATCTTTCAAGATCGAAATTAGTTCCCTCACACCGGTGACCGACA

TUR278|AB863186.1| GCTCTAAAGATAAAAGATCTTTCAAGATCAAACATAGTTCCCTCACACCGGTGACCGACA

TUR279|AB863187.1| ACTCTAAAGATAAAAGATCTTTCAAGATCAAACATAGTTCCCTCACACCGGTGACCGACA

TUR285|AB863188.1| ACTCTAAAAATAAAAGATCTTTCAAGATCAAACATAGTTCCCTCACACCGGTGACCGACA

TUR289|AB863189.1| ACTCTAAAGATAAAAGATCTTTCAAGATCGAAACTAGTTCCCTCACACCGGTGACCGATA

TUR303|AB863190.1| ACTCTAAAGATAAAAGATCTTTTAAGATCAAACATAGTTCCCTCACACCGGTGACCGACA

TUR306|AB863191.1| ACTCTAAAGATAAAAGATCTTTCAAGATCAAAATCAGTTCCCTCACACCGGTGACCGACA

Cabb-S|NC_001497.1| GGATTACCACCGTAAGGTTTCAGAACAACATCGAAAGCGTTTACGCCAACTTCGACTCTC

CRO180A|AB863192.1| GGTTTACCACCGTAAGGTTTCAGAACAACATCGAATGCGTTTACGCCAACTTCGACTCTC

GRC83|AB863193.1| GCACATCCACCGTTAAGTTTCAGAACAACATCGAATGCGTTTACGCCAACTTCGACTCTC

GRC84B|AB863194.1| GCACATCCACCGTTGAGTTTCAGAACAACATCGAATGCGTTTACGCCAACTTCGACTCTC

GRC86B|AB863195.1| GCACATCCACCGTTGAGTTTCAGAACAACATCGAATGCGTTTACGCCAACTTCGACTCTC

GRC86D|AB863196.1| GCACATCCACCGTTGAGTTTCAGAACAACATCGAATGCGTTTACGCCAACTTCGACTCTC

GRC87E|AB863197.1| GCACATCCACCGTTGAGTTTCAGAACAACATCGAATGCGTTTACGCCAACTTCGACTCTC

GRC87G|AB863198.1| GCACATCCACCGTTGAGTTTCAGAACAACATCGAATGCGTTTACGCCAACTTCGACTCTC

GRC91B|AB863199.1| GCACATCCACCGTTGAGTTTCAGAACAACATCGAATGCGTTTACGCCAACTTCGACTCTC

GRC92A|AB863200.1| GCACATCCACCGTTGAGTTTCAGAACAACATCGAATGCGTTTACGCCAACTTCGACTCTC

GRC92C|AB863201.1| GCACATCCACCGTTGAGTTTCAGAACAACATCGAACGCGTTTACGCCAACTTCGACTCTC

GRC92D|AB863202.1| GCACATCCACCGTTGAGTTTCAGAACAACATCGAATGCGTTTACGCCAACTTCGACTCTC

IRN1|AB863136.1| GGTTTACCACCGTAAGGTTTCAGAACAACATCGAATGCGTTTACGCCAACTTCGACTCTC

IRN2|AB863137.1| GGTTTACCACCGTAAGGTTTCAGAACAACATCGAATGCGTTTACGCCAACTTCGACTCTC

IRN3|AB863138.1| GGTTTACCACCGTAAGGTTTCAGAACAACATCGAATGCGTTTACGCCAACTTCGACTCTC

IRN4|AB863139.1| GGTTTACCACCGTAAGGTTTCAGAACAACATCGAAAGCGTTTACGCCAACTTCGACTCTC

IRN5|AB863140.1| GGTTTACCACCGTAAGGTTTCAGAACAACATCGAATGCGTTTACGCCAACTTCGACTCTC

IRN6|AB863141.1| GGTTAACCACCGTAAGGTTTCAGAACAACATCGAATGCGTTTACGCCAACTTCGACTCTC

IRN7|AB863142.1| GGTTTACCACCGTAAGGTTTCAGAACAACATCGAATGCGTTTACGCCAACTTCGACTCTC

IRN8|AB863143.1| GGTTTACCACCGTAAGGTTTCAGAACAACATCGAATGCGTTTACGCCAACTTCGACTCTC

IRN9|AB863144.1| GGTTTACCACCGTAAGGTTTCAGAACAACATCGAATGCGTTTACGCCAACTTCGACTCTC

IRN10|AB863145.1| GGTTTACCACCGTAAGGTTTCAGAACAACATCGAATGCGTTTACGCCAACTTCGACTCTC

IRN11|AB863146.1| GGTTTACCACCGTAAGGTTTCAGAACAACATCGAATGCGTTTACGCCAACTTCGACTCTC

IRN12|AB863147.1| GGTTTACCACCGTAAGGTTTCAGAACAACATCGAATGCGTTTACGCCAACTTCGACTCTC

IRN13|AB863148.1| GGTTAACCACCGTAAGGTTTCAGAACAACATCGAATGCGTTTACGCCAACTTCGACTCTC

IRN14|AB863149.1| GGTTTACCACCGTAAGGTTTCAGAACAACATCGAATGCGTTTACGCCAACTTCGACTCTC

IRN15|AB863150.1| GGTTTACCACCGTAAGGTTTCAGAACAACATCGAATGCGTTTACGCCAACTTCGACTCTC

IRN16|AB863151.1| GGTTTACCACCGTAAGGTTTCAGAACAACATCGAATGCGTTTACGCCAACTTCGACTCTC

IRN17|AB863152.1| GGTTTACCACCGTAAGGTTTCAGAACAACATCGAATGCGTTTACGCCAACTTCGACTCTC

IRN18|AB863153.1| GGTTTACCACCGTAAGGTTTCAGAACAACATCGAATGCGTTTACGCCAACTTCAACTCTC

IRN19|AB863154.1| GGTTTACCACCGTAAGGTTTCAGAACAACATCGAATGCGTTTACGCCAACTTCGACTCTC

IRN20|AB863155.1| GGTTTACCACCGTAAGGTTTCAGAACAACATCGAATGCGTTTACGCCAACTTCGACTCTC

IRN21|AB863156.1| GGTTAACCACCGTAAGGTTTCAGAACAACATCGAATGCGTTTACGCCAACTTCGACTCTC

JPNHGB340|AB863157.1| GGTTTACCACCGTAAGGTTTCAGAACAACATCGAAAGCGTTTACGCCAACTTCGACTCTC

JPNKWB778|AB863158.1| GGTTTACCACCGTAAGGTTTCAGAACAACATCGAAAGCGTTTACGCCAACTTCGACTCTC

JPNM|AB863159.1| GGTTTACCACCGTAAGGTTTCAGAACAACATCGAATGCGTTTACGCCAACTTCGACTCTC

JPNN|AB863160.1| GGTTCATCACCGTAAGGTTTAAGAACAACATCGAAAGCGTTTACGCCAACTTCGACTCTC

JPNS1|AB863161.1| GGTTCATCACCGTAAGGTTTCAGAACAACATCGAAAGCGTTTACGCCAACTTCGACTCTC

JPNS2|AB863162.1| GGTTCATCACCGTAAGGTTTCAGAACAACATCGAAAGCGTTTACGCCAACTTCGACTCTC

JPNTKD762|AB863163.1| GGTTTACCACCGTAAGGTTTCAGAACAACATCGAAAGCGTTTACGCCAACTTCGACTCTC

JPNUV1|AB863164.1| GGTTTACCACCGTAAGGTTTCAGAACAACATCGAATGCGTTTACGCCAACTTCGACTCTC

JPNUV26|AB863165.1| GGTTTACCACCGTAAGGTTTCAGAACAACATCGAATGCGTTTACGCCAACTTCGACTCTC

TUR1|AB863166.1| GGTTTACCACCGTAAGGTTTCAGAACAACATCGAATGCGTTTACGCCAACTTCGACTCTC

TUR2|AB863167.1| GGTTTACCACCGTAAGGTTTCAGAACAACATCGAATGCGTTTACGCCAACTTCGACTCTC

TUR4|AB863168.1| GGTTTACCACCGTAAGGTTTCAGAACAACATCGAATGCGTTTACGCCAACTTCGACTCTC

TUR5|AB863169.1| GGTTTACCACCGTAAGGTTTCAGAACAACATCGAATGCGTTTACGCCAACTTCGACTCTC

TUR12|AB863170.1| GGTTTACCACCGTAAGGTTTCAGAACAACATCGAAAGCGTTTACGCCAACTTCGACTCTC

TUR34|AB863171.1| GGTTTACCACCGTAAGGTTTCAGAACAACATCGAATGCGTTTACGCCAACTTCGACTCTC

TUR50|AB863172.1| GGTTTACCACCGTAAGGTTTCAGAACAACATCGAATGCGTTTACGCCAACTTCGACTCTC

TUR59|AB863173.1| GGTTTACCACCGTAAGGTTTCAGAACAACATCGAATGCGTTTACGCCAACTTCGACTCTC

TUR69|AB863174.1| GGTTTACCACCGTAAGGTTTCAGAACAACATCGAACGCGTTTACGCCAACTTCGACTCTC

TUR81|AB863175.1| GGTTTACCACCGTAAGGTTTCAGAACAACATCGAATGCGTTTACGCCAACTTCGACTCTC

TUR84|AB863176.1| GGTTTACCACCGCAAGGTTTCAGAACAACATCGAATGCGTTTACACCAACTTCGACTCTC

TUR94|AB863177.1| GGTTTACCACCGTAAGGTTTCAGAACAACATCGAATGCGTTTACGCCAACTTCGACTCTC

TUR213|AB863178.1| GGTTTACCACCGTAAGGTTTCAGAACAACATCGAATGCGTTTACGCCAACTTCGACTCTC

TUR214|AB863179.1| GGTTTACCACCGTAAGGTTTCAGAACAACATCGAACGCGTTTACGCCAACTTCGACTCTC

TUR216|AB863180.1| GCACATCCACCGTTGAGTTTCAGAACAACATCGAATGCGTTTACGCCAACTTCGACTCTC

TUR220|AB863181.1| GGTTTACCACCGCAAGGTTTCAGAACAACATCGAATGCGTTTACGCCAACTTCGACTCTC

TUR239|AB863182.1| GGTTTACCACCGTAAGGTTTCAGAACAACATCGAACGCGTTTACGCCAACTTCGACTCTC

TUR244|AB863183.1| GGTTTACCACCGTAAGGTTTCAGAACAACATCGAATGCGTTTACGCCAACTTCGACTCTC

TUR249|AB863184.1| GGTTTACCACCGTAAGGTTTCAGAACAACATCGAATGCGTTTACGCCAACTTCGACTCTC

TUR263|AB863185.1| GGTTTACCACCGTAAGGTTTCAGAACAACATCGAATGCGTTTACGCCAACTTCGACTCTC

TUR278|AB863186.1| GGTTTACCACCGTAAGGTTTCAGAACAACATCGAACGCGTTTACGCCAACTTCGACTCTC

TUR279|AB863187.1| GGTTTACCACCGTAAGGTTTCAGAACAACATCGAATGCGTTTACGCCAACTTCGACTCTC

TUR285|AB863188.1| GGTTTACCACCGTAAGGTTTCAGAACAACATCGAACGCGTTTACGCCAACTTCGACTCTC

TUR289|AB863189.1| GGTTTACCACCGTAAGGTTTCAGAACAACATCGAATGCGTTTACGCCAACTTCGACTCTC

TUR303|AB863190.1| GGTTTACCACCGTAAGGTTTCAGAACAACATCGAATGCGTTTACGCCAACTTCGACTCTC

TUR306|AB863191.1| GGTTTACCACCGTAAGGTTTCAGAACAACATCGAATGCGTTTACGCCAACTTCGACTCTC

Cabb-S|NC_001497.1| AACTCAAGTCGTCGTACGATGGTAGATCTAAAAAGATCAAGACTCTAAGCCTTAAAAATC

CRO180A|AB863192.1| AGCTCAAGTCGTCGTACGATGGTAGATCTAAAAAGATCAAGACTCTAAGCCGTAAAAATC

GRC83|AB863193.1| AACTAAAGTCGTCGTACGATGGTAGATCTAAAAAGATCAAGACTCTAAGCCTTAAAAATC

GRC84B|AB863194.1| AACTAAAGTCGTCGTACGATGGTAGATCTAAAAAGATCAAGACTCTAAGCCTTAAAAATC

GRC86B|AB863195.1| AGCTCAAGTCGTCGTACGATGGAAGATCTAAAAAGATCAAAACTCTAAGTCTTAAAAATC

GRC86D|AB863196.1| AGCTCAAGTCGTTGTACGATGGTAGATCTAAAAAGATCAAGACTCTAAGCCTTAAAAATC

GRC87E|AB863197.1| AGCTCAAGTCGTCGTACGATGGTAGATCTAAAAAGATCAAGACTCTAAGCCTTAAAAATC

GRC87G|AB863198.1| AGCTCAAGTCGTCGTACGATGGTAGATCTAAAAAGATCAAGACTCTAAGCCTTAAAAATC

GRC91B|AB863199.1| AGCTCAAGTCGTCGTACGATGGTAGATCTAAAAAAATCAAGACTCTAAGCCTTAAAAATC

GRC92A|AB863200.1| AGCTCAAGTCGTCGTACGATGGTAGATCTAAAAAGATCAAGACTCTAAGCCTTAAAAATC

GRC92C|AB863201.1| AACTAAAGTCGTCGTACGATGGTAGATCTAAAAAGATCAAGACTCTAAGCCTTAAAAATC

GRC92D|AB863202.1| AGCTCAAGTCGTCGTACGATGGTAGATCTAAAAAGATCAAAACTCTAAGCCTTAAAAATC

IRN1|AB863136.1| AGCTCAAGTCGTCGTACGATGGTAGATCTAAAAAGATCAAGACTCTAAGCCTTAAAAAAC

IRN2|AB863137.1| AGCTCAAGTCGTCGTACGATGGTAGATCTAAAAAGATCAAGACTCTAAGCCTTAAAAATC

IRN3|AB863138.1| AGCTCAAGTCGTCGTACGATGGTAGATCTAAAAAGATCAAGACTCTAAGCCTTAAAAAAC

IRN4|AB863139.1| AGCTCAAGTCGTCGTACGATGGTAGATCTAAAAAGATCAAGAATCTAAGCCTTAAAAATC

IRN5|AB863140.1| AACTCAAGTCGTCGTACGATGGTAGATCTAAAAAGATCAAGAATCTAAGCCTTAAAAATC

IRN6|AB863141.1| AGCTCAAGTCGTCGTACGATGGTAGATCTAAAAAGATCAAGACTCTAAGCCTTAAAAATC

IRN7|AB863142.1| AGCTCAAGTCGTCGTACGATGGTAGATCTAAAAAGATCAAGAATCTAAGCCTTAAAAATC

IRN8|AB863143.1| AACTCAAGTCGTCGTACGATGGTAGATCTAAAAAGATCAAGAATCTAAGCCTTAAAAATC

IRN9|AB863144.1| AGCTCAAGTCGTCGTACGATGGTAGATCTAAAAAGATCAAGAATCTAAGCCTTAAAAATC

IRN10|AB863145.1| AGCTCAAGTCGTCGTACGATGGTAGATCTAAAAAGATCAAGAATCTAAGCCTTAAAAATC

IRN11|AB863146.1| AGCTCAAGTCGTCGTACGATGGTAGATCTAAAAAGATCAAGACTCTAAGCCTTAAAAATC

IRN12|AB863147.1| AGCTCAAGTCGTCGTACGATGGTAGATCTAAAAAGATCAAGAATCTAAGCCTTAAAAATC

IRN13|AB863148.1| AGCTCAAGTCGTCGTACGATGGTAGATCTAAAAAGATCAAGACTCTAAGCCTTAAAAATC

IRN14|AB863149.1| AGCTCAAGTCGTCGTACGATGGTAGATCTAAAAAGATCAAGACTCTAAGCCTTAAAAATC

IRN15|AB863150.1| AGCTCAAGTCGTCGTACGATGGTAGATCTAAAAAGATCAAGAATCTAAGCCTTAAAAATC

IRN16|AB863151.1| AGCTCAAGTCGTCGTACGATGGTAGATCTAAAAAGATCAAGAATCTAAGCCTTAAAAATC

IRN17|AB863152.1| AGCTCAAGTCGTCGTACGATGGTAGATCTAAAAAGATCAAGAATCTAAGCCTTAAAAATC

IRN18|AB863153.1| AGCTCAAGTTGTCGTACGATGGTAGATCTAAAAAGATCAAGAATCTAAGCCTTAAAAATC

IRN19|AB863154.1| AGCTCAAGTCGTCGTACGATGGTAGATCTAAAAAGATCAAGACTCTAAGCCTTAAAAATC

IRN20|AB863155.1| AGCTCAAGTCGTCGTACGATGGTAGATCTAAAAAGATCAAGAATCTAAGCCTTAAAAATC

IRN21|AB863156.1| AGCTCAAGTCGTCGTACGATGGTAGATCTAAAAAGATCAAGACTCTAAGCCTTAAAAATC

JPNHGB340|AB863157.1| AACTAAAGTCGTCGTACGATGGTAGATCTAAAAAGATCAAGAATTTAAGCCTTAAAAATC

JPNKWB778|AB863158.1| AACTCAAGTCGTCGTACGATGGTAGATCTAAAAAGATCAAGAATCTAAGCCTTAAAAATC

JPNM|AB863159.1| AACTCAAGTCGTCGTACGATGGTAGATCTAAAAAGATCAAGAATCTAAGCCTTAAAAATC

JPNN|AB863160.1| AACTAAAGTCGTCGTACGATGGTAGATCTAAAAAGATCAAGAATCTAAGCTTTAAAAAAC

JPNS1|AB863161.1| AACTAAAGTCGTCGTACGATGGTAGATCTAAAAAGATCAAGAATCTAAGCTTTAAAAAAC

JPNS2|AB863162.1| AACTAAAGTCGTCGTACGATGGTAGATCTAAAAAGATCAAGAATCTAAGCTTTAAAAAAC

JPNTKD762|AB863163.1| AACTAAAGTCGTCGTACGATGGTAGATCTAAAAAGATCAAGAATCTAAGCCTTAAAAATC

JPNUV1|AB863164.1| AACTCAAGTCGTCGTACGATGGTAGATCTAAAAAGATCAAGAATCTAAGCCTTAAAAATC

JPNUV26|AB863165.1| AACTCAAGTCGTCGTACGATGGTAGATCTAAAAAGATCAAGAATCTAAGCCTTAAAAATC

TUR1|AB863166.1| AACTCAAGTCGTCGTACGATGGAAGATCTAAAAAGATCAAGAATCTAAGCCTTAAAAATC

TUR2|AB863167.1| AGCTCAAGTCGTCGTACGATGGTAGATCTAAAAAGATCAAAAATCTAAGCCTTAAAAACC

TUR4|AB863168.1| AGCTCAAGTCGTCGTACGATGGTAGATCTAAAAAGATCAAAAATCTAAGTCTTAAAAACC

TUR5|AB863169.1| AGCTCAAGTCGTCGTACGATGGTAGATCTAAAAAGATCAAGAATCTAAGCCTTAAAAATC

TUR12|AB863170.1| AGCTCAAGTCGTCGTACGATGGTAGATCTAAAAAGATCAAGATTCTAAGCCTTAAAAATC

TUR34|AB863171.1| AACTTAAGTCGTCGTACGATGGAAGATCTAAAAAGATCAAGAATCTAACCCTTAAAAATC

TUR50|AB863172.1| AGCTCAAGTCGTCGTACGATGGAAGATCTAAAAAGATCAAGACTCTAAGCCTTAAAAATC

TUR59|AB863173.1| AGCTCAAGTCGTCGTACGATGGTAGATCTAAAAAGATCAAGACTCTAAGCCTTAAAAATC

TUR69|AB863174.1| AGCTCAAGTCGTCGTACGATGGTAGATCTAAAAAGATCAAGAATCTAAGCCTTAAAAATC

TUR81|AB863175.1| AGCTCAAGTCGTCGTACGATGGTAGATCTGAAAAGATCAAAAATCTAAGCCTTAAAAATC

TUR84|AB863176.1| AGCTCAAGTCGTCGTACGATGGTAGATCTGAAAAGATCAAGAATCTAAGCCTTAAAAATC

TUR94|AB863177.1| AGCTCAAGTCGTCGTACGATGGTAGATCTAAAAAGATCAAGAATCTAAGCCTTAAAAATC

TUR213|AB863178.1| AGCTCAAGTCGTCGTACGATGGTAGATCTAAAAAGATCAAGACTCTAAGCCTTAAAAATC

TUR214|AB863179.1| AGCTCAAGTCGTCGTACGATGGTAGATCTAAAAAGATCAAGACTCTAAGCCTTAAAAATC

TUR216|AB863180.1| AGCTCAAGTCGTCGTACGATGGTAGATCTAAAAAGATCAAGAATCTAAGCCTTAAAAATC

TUR220|AB863181.1| AGCTCAAGTCGTCGTACGATGGTAGATCTGAAAAGATCAAGAATCTAAGCCTTAAAAATC

TUR239|AB863182.1| AGCTCAAGTCGTCGTACGATGGAAGATCTAAAAAGATCAAAAATCTAAGCCTTAAAAATC

TUR244|AB863183.1| AACTCAAGTCGTCGTACGATGGAAGATCTAAAAAGATCAAAGATCTAAGCCTTAAAAATC

TUR249|AB863184.1| AGCTCAAGTCGTCGTACGATGGTAGATCTAAAAAGATCAAGAATCTAAGCCTTAAAAATC

TUR263|AB863185.1| AGCTCAAGTCGTCGTACGATGGTAGATCTAAAAAGATCAAAAATCTAAGCCTTAAAAATC

TUR278|AB863186.1| AGCTCAAGTCGTCGTACGATGGTAGATCTAAAAAGATCAAGACTCTAAGCCTTAAAAATC

TUR279|AB863187.1| AGCTCAAGTCGTCGTACGATGGTAGATCTAAAAAGATCAAGAATCTAAGCCTTAAAAATC

TUR285|AB863188.1| AGCTCAAGTCGTCGTACGATGGTAGATCTACAAAGATCAAGAATCTAAGCCTTAAAAATC

TUR289|AB863189.1| AGCTCAAGTCGTCGTACGATGGTAGATCTAAAAAGATCAAGAATCTAAGCCTTAAAAATC

TUR303|AB863190.1| AGCTCAAGTCGTCGTACGATGGTAGATCTAAAAAGATCAAAACTCTAAGCCTTAAAAATC

TUR306|AB863191.1| AGCTCAAGTCGTCGTACGATGGTAGATCTAAAAAGATCAAGAATCTAAGCCTTAAAAATC

Cabb-S|NC_001497.1| TTAGATGTTACGAAGCCTTCCTCAGGAAGTACCTTCTGGAACAATA-AATCTCTCTGAGA

CRO180A|AB863192.1| TTAGATGTTACGAAGCCTTCCTAAGGAAGTACCTTTTGGAACAATAAAATCTCTCTGAGA

GRC83|AB863193.1| TTAGATGTTACGAAGCCTTCCTCAGGAAGTACCTTCTGGAGCAATAAAATCTCTCTGAGA

GRC84B|AB863194.1| TTAGATGTTACGAAGCCTTCCTCAGGAAGTACCTTCTGGAGCAATAAAATCTCTCTGAGA

GRC86B|AB863195.1| TTAGATGTTATGAAGCCTTCCTCAGGAAGTACCTTCTGGAACAATAAAATCTCTCTGAGA

GRC86D|AB863196.1| TTAGATGTTACGAAGCCTTCCTCAGGAAGTACCTTCTGGAACAATAAAATCTCTCTGAGA

GRC87E|AB863197.1| TTAGATGTTACGAAGCCTTCCTCAGGAAGTACCTTCTGGAACAATAAAATCTCTCTGAGA

GRC87G|AB863198.1| TTAGATGTTACGAAGCCTTCCTCAGGAAGTACCTTCTGGAACAATAAAATCTCTCTGAGA

GRC91B|AB863199.1| TTAGATGTTACGAAGCCTTCCTCAGGAAGTACCTTCTGGAACAATAAAATCTCTCTGAGA

GRC92A|AB863200.1| TTAGATGTTACGAAGCCTTCCTCAGGAAGTACCTTCTGGAACAATAAAATCTCTCTGAGA

GRC92C|AB863201.1| TTAGATGTTACGAAGCCTTCCTCAGGAAGTACCTTCTGGAACAATAAAATCTCTCTGAGA

GRC92D|AB863202.1| TTAGATGTTATGAAGCCTTCCTCAGGAAGTACCTTCTGGAACAATAAAATCTCTCTGAGA

IRN1|AB863136.1| TTAGATGTTACGAAGCCTTCTTCAGGAAGTACCTTCTGGAACAATAAAATCTCTCTGAGA

IRN2|AB863137.1| TTAGATGTTACGAAGCCTTCCTCAGGAAGTACCTTCTGGAACAATA-AATCTCTCTGAGA

IRN3|AB863138.1| TTAGATGTTACGAAGCCTTCTTCAGGAAGTACCTTCTGGAACAATAAAATCTCTCTGAGA

IRN4|AB863139.1| TTAGATGCTATGAAGCCTTCCTTAGGAAGTACCTTCTGGAACAATA-AATCTCTCTGAGA

IRN5|AB863140.1| TTAGATGCTACGAAGCCTTCCTCAGGAAGTACCTTCTGGAACAATA-AATCTCTCTGAGA

IRN6|AB863141.1| TTAGATGTTACGAAGCCTTCCTCAGGAAGTACCTTCTGGAACAATA-AATCTCTCTGAGA

IRN7|AB863142.1| TTAGATGCTATGAAGCCTTCCTCAGGAAGTACCTTCTGGAACAATA-AATCTCTCTGAGA

IRN8|AB863143.1| TTAGATGCTACGAAGCCTTCCTCAGGAAGTACCTTCTGGAACAATA-AATCTCTCTGAGA

IRN9|AB863144.1| TTAGATGCTATGAAGCCTTCCTCAGGAAGTACCTTCTGGAACAATA-AATCTCTCTGAGA

IRN10|AB863145.1| TTAGATGTTACGAAGCCTTCCTCAGGAAGTACCTTCTGGAACAATA-AATCTCTCTGAGA

IRN11|AB863146.1| TTAGATGTTACGAAGCCTTCCTCAGGAAGTACCTTCTGGAACAATA-AATCTCTCTGAGA

IRN12|AB863147.1| TTAGATGTTACGAAGCCTTCCTCAGGAAGTACCTTCTGGAACAATA-AATCTCTCTGAGA

IRN13|AB863148.1| TTAGATGTTACGAAGCCTTCCTCAGGAAGTACCTTCTGGAACAATA-AATCTCTCTGAGA

IRN14|AB863149.1| TTAGATGTTACGAAGCCTTCCTCAGGAAGTACCTTCTGGAACAATA-AATCTCTCTGAGA

IRN15|AB863150.1| TTAGATGCTATGAAGCCTTCCTTAGGAAGTACCTTCTGGAACAATA-AATCTCTCTGAGA

IRN16|AB863151.1| TTAGATGCTATGAAGCCTTCCTCAGGAAGTACCTTCTGGAACAATA-AATCTCTCTGAGA

IRN17|AB863152.1| TTAGATGCTATGAAACCTTCCTTAGGAAGTACCTTCTGGAACAATA-AATCTCTCTGAGA

IRN18|AB863153.1| TTAGATGTCACGAAGCCTTCCTCAGGAAGTACCTTCTGGAACAATA-AATCTCTCTGAGA

IRN19|AB863154.1| TTAGATGTTACGAAGCCTTCCTCAGGAAGTACCTTCTGGAACAATA-AATCTCTCTGAGA

IRN20|AB863155.1| TTAGATGCTATGAAGTCTTCCTTAGGAAGTACCTTCTGGAACAATA-AATCTCTCTGAGA

IRN21|AB863156.1| TTAGATGTTACGAAGCCTTCCTCAGGAAGTACCTTCTGGAACAATA-AATCTCTCTGAGA

JPNHGB340|AB863157.1| TTAGATGTTACGAAGCCTTCCTCAGGAAGTACCTTCTGGAACAATA-AATCTCTCTGAGA

JPNKWB778|AB863158.1| TTAGATGTAATGAAGCCTTCCTCAGGAAGTACCTTCTGGAACAATA-AATCTCTCTGAGA

JPNM|AB863159.1| TTAGATGTTACGAAGCCTTCCTCAGGAAGTACCTTCTGGAACAATA-AATCTCTCTGAGA

JPNN|AB863160.1| TTAGATATTATGAAGCCTTCCTCAGGAAGTACCTTCTGGAACAATA-GATC---------

JPNS1|AB863161.1| TTAGATATTATGAAGCCTTCCTCAGGAAGTACCTTCTGGAACAATA-AATC---------

JPNS2|AB863162.1| TTAGATATTATGAAGCCTTCCTCAGGAAGTACCTTCTGGAACAATA-AATC---------

JPNTKD762|AB863163.1| TTAGATGTTACGAGGCCTTCCTCAGGAAGTACCTTCTGGAACAATA-AATCTCTCTGAGA

JPNUV1|AB863164.1| TTAGATGTTACGAAGCCTTCCTCAGGAAGTACCTTCTGGAACAATA-AATCTCTCTGAGA

JPNUV26|AB863165.1| TTAGATGTTACGAAGCCTTCCTCAGGAAGTACCTTCTGGAACAATA-AATCTCTCTGAGA

TUR1|AB863166.1| TTAGATGTTACGAAGCCTTCCTCAGGAAGTACCTTCTGGAACAATA-AATCTCTCTGAGA

TUR2|AB863167.1| TTAGATGCTACGAAACCTTCCTAAGGAAGTACCTTTTGGAACAATAAAATCTCTCTGAGA

TUR4|AB863168.1| TTAGATGCTACGAAACCTTCCTAAGGAAGTACCTTTTGGAACAATAAAATCTCTCTGAGA

TUR5|AB863169.1| TTAGATGTTACGAAGCCTTCCTCAGGAAGTACCTTCTGGAACAATA-AATCTCTCTGAGA

TUR12|AB863170.1| TTAGATGTTACGAAGCTTTCCTAAGGAAGTACTTTCTGGAACAATA-AATCTCTCTGAGA

TUR34|AB863171.1| TTAGATGTTACGAAGCCTTCCTCAGGAAGTACCTTCTGGAACAATAAAATCTCTCTGAGA

TUR50|AB863172.1| TTAGATGTTACGAAGCCTTCCTCAGGAAGTACCTTCTGGAACAATAAAATCTCTCTGAGA

TUR59|AB863173.1| TTAGATGTTACGAAGCCTTCCTCAGGAAGTACCTTCTGGAACAATAAAATCTCTCTGAGA

TUR69|AB863174.1| TTAGATGTTACGAAGCCTTCCTCAGGAAGTACCTTCTAGAACAATAAAATCTCTCTGAGA

TUR81|AB863175.1| TTAGATGTTACGAAGCCTTCCTCAGGAAGTACCTTCTGGAACAATAAAATCTCTCTGAGA

TUR84|AB863176.1| TTAGATGTTACGAAGCCTTCCTCAGGAAGTACCTTCTGGAACAATAAAATCTCTCTGAGA

TUR94|AB863177.1| TTAGATGTTACGAAGCCTTCCTCAGGAAGTACCTTCTGGAACAATA-AATCTCTCTGAGA

TUR213|AB863178.1| TTAGATGTTACGAAACCTTCCTAAGGAAGTACCTTTTGGAACAATAAAATCTCTCTGAGA

TUR214|AB863179.1| TTAGATGTTACGAAACCTTCCTCAGGAAGTACCTTCTGGAACAATA-AATCTCTCTGAGA

TUR216|AB863180.1| TTAGATGTTACGAAGCCTTCCTTAGGAAGTACCTTCTGGAACAATA-AATCTCTCTGAGA

TUR220|AB863181.1| TTAGATGTTACGAAGCCTTCCTCAGGAAGTACCTTCTGGAACAATAAAATCTCTCTGAGA

TUR239|AB863182.1| TTAGATGTTACGAAGCCTTCCTCAGGAAGTACCTTCTGGAACAATA-AATCTCTCTGAGA

TUR244|AB863183.1| TTAGATGTTACGAAGCCTTCCTCAGGAAGTACCTTCTGGAACAATA-AATCTCTCTGAGA

TUR249|AB863184.1| TTAGATGTTATGAAACCTTCCTAAGGAAGTACCTTTTGGAACAATA-AATCTCTCTGAGA

TUR263|AB863185.1| TTAGATGCTACGAAGCCTTCCTAAGGAAGTACCTTTTGGAACAATAAAATCTCTCTAAGA

TUR278|AB863186.1| TTAGATGTTACGAAGCCTTCCTCAGGAAGTACCTTCTGGAACAATAAAATCTCTCTGAGA

TUR279|AB863187.1| TTAGATGTTACGAAGCCTTCCTCAGGAAGTACCTTCTGGAACAATAAAATCTCTCTGAGA

TUR285|AB863188.1| TTAGATGCTACGAAGCCTTCCTAAGGAAGTACCTTCTGGAACAATAAAATCTCTCTGAGA

TUR289|AB863189.1| TTAGATGTTACGAAGCCTTCCTCAGGAAGTACCTTCTGGAACAATAAAATCTCTCTGAGA

TUR303|AB863190.1| TTAGATGTTACGAAGCCTTCCTCAGGAAGTACCTTCTGGAACAATA-AATCTCTCTGAGA

TUR306|AB863191.1| TTAGATGCTACGAAACCTTCCTAAGGAAGTACCTTTTGGAACAATAAAATCTCTCTGAGA

Cabb-S|NC_001497.1| ATAGTACTCTATTGAGTATCCACAGGAAA-AATAACCTTCTGTGTTGAGATGGATTTGTA

CRO180A|AB863192.1| ATAGTACTCTATTGAGTATCCACAGAAAA-AACAATCTTCTGTGTTGAGATGGATTTGTA

GRC83|AB863193.1| ATAGTACTCTATCGAGTATCCACAGAAAA-AATAATCTTCTGTGTTGAGATGGATTTGTA

GRC84B|AB863194.1| ATAGTACTCTATCGAGTATCCACAGAAAA-AATAATCTTCTGTGTTGAGATGGATTTGTA

GRC86B|AB863195.1| ATAGTACTCTATTGAGTATCCACAGAAAAAAATAATCTTCTGTGTTGAGATGGATTTGTA

GRC86D|AB863196.1| ATAGTACTCTATTGAGTATCCACAGAAAA-AATAATCTTCTGTGTTGAGATGGATTTGTA

GRC87E|AB863197.1| ATAGTACTCTATTGAGTATCCACAGAAAA-AATAATCTTCTGTGTTGAGATGGATTTGTA

GRC87G|AB863198.1| ATAGTACTCTATTGAGTATCCACAGAAAA-AATAATCTTCTGTGTTGAGATGGATTTGTA

GRC91B|AB863199.1| ATAGTACTCTATTGAGTATCCACAGAAAA-AATAATCTTCTGTGTTGAGATGGATTTGTA

GRC92A|AB863200.1| ATAGTACTCTATTGAGTATCCACAGAAAA-AATAATCTTCTGTGTTGAGATGGATTTGTA

GRC92C|AB863201.1| ATAGTACTCTATTGAGTATCCACAGAAAA-AATAATCTTCTGTGTTGAGATGGATTTGTA

GRC92D|AB863202.1| ATAGTACTCTATTGAGTATCCACAGAAAA-AATAATCTTCTGTGTTGAGATGGATTTGTA

IRN1|AB863136.1| ATAATACTCTATTGAGTATCCACAGAAAA-AATAACCTTCTGTGTTGAGATGGATTTGTA

IRN2|AB863137.1| ATAGTACTCTATCGAGTATCCACAGAAAA-AATAACCTTCTGTGTTGAGATGGATTTGTA

IRN3|AB863138.1| ATAATACTCTATTGAGTATCCACAGAAAA-AATAACCTTCTGTGTTGAGATGGATTTGTA

IRN4|AB863139.1| ATAGTACTCTATCGAGTATCCACAGAAAA-AATAACCTTCTGTGTTGAGATGGATTTGTA

IRN5|AB863140.1| ATAGTACTCTATCGAGTATCCACAGATAA-AATAATCTTCTGTGTTGAGATGGATTTGTA

IRN6|AB863141.1| ATAGTACTCTATCGAGTATCCACAGATAA-AATAATCTTCTGTGTTGAGATGGATTTGTA

IRN7|AB863142.1| ATAGTACTCTATCGAGTATCCACAGATAA-AATAATCTTCTGTGTTGAGATGGATTTGTA

IRN8|AB863143.1| ATAGTACTCTATCGAGTATCCACAGATAA-AATAATCTTCTGTGTTGAGATGGATTTGTA

IRN9|AB863144.1| ATAGTACTCTATCGAGTATCCACAGATAA-AATAACCTTCTGTGTTGAGATGGATTTGTA

IRN10|AB863145.1| ATAGTACTCTATCGAGTATCCACAGATAA-AATAATCTTCTGTGTTGAGATGGATTTGTA

IRN11|AB863146.1| ATAGTACTCTATCGAGTATCCACAGAAAA-AATAACCTTCTGTGTTGAGATGGATTTGTA

IRN12|AB863147.1| ATAGTACTCTATCGAGTATCCACAGATAA-AATAATCTTCTGTGTTGAGATGGATTTGTA

IRN13|AB863148.1| ATAGTACTCTATCGAGTATCCACAGATAA-AATAATCTTCTGTGTTGAGATGGATTTGTA

IRN14|AB863149.1| ATAGTACTCTATCGAGTATCCACAGAAAA-AATAACCTTCTGTGTTGAGATGGATTTGTA

IRN15|AB863150.1| ATAGTACTCTATCGAGTATCCACAGAAAA-AATAACCTTCTGTGTTGAGATGGATTTGTA

IRN16|AB863151.1| ATAGTACTCTATCGAGTATCCACAGAAAA-AATAACCTTCTGTGTTGAGATGGATTTGTA

IRN17|AB863152.1| ATAGTACTCTATCGAGTATCCACAGAAAA-AATAACCTTCTGTGTTGAGATGGATTTGTA

IRN18|AB863153.1| ATAGTACTCTATCGAGTATCCACAGAAAA-AATAACCTTCTGTGTTGAGATGGACTTGTA

IRN19|AB863154.1| ATAGTACTCTATCGAGTATCCACAGAAAA-AATAACCTTCTGTGTTGAGATGGATTTGTA

IRN20|AB863155.1| ATAGTACTCTATCGAGTATCCACAGAAAA-AATAACCTTCTGTGTTGAGATGGATTTGTA

IRN21|AB863156.1| ATAGTACTCTATCGAGTATCCACAGATAA-AATAATCTTCTGTGTTGAGATGGATTTGTA

JPNHGB340|AB863157.1| ATAGTACTCTATCGAGTATCCACAGAAAA-AACAATCTTCTGTGTTGAGATGGATTTGTA

JPNKWB778|AB863158.1| ATAGTACTCTATTGAGTATCCACAGAAAA-AACAATCTTCTGTGTTGAGATGGACCTGTA

JPNM|AB863159.1| ATAGTACTCTATTGAGTATCCACAGAAAA-AACAATCTTCTGTGTTGAGATGGATCTGTA

JPNN|AB863160.1| -----------------------------------------------CAATGGATTTGTA

JPNS1|AB863161.1| -----------------------------------------------CGATGGATTTGTA

JPNS2|AB863162.1| -----------------------------------------------CGATGGATTTGTA

JPNTKD762|AB863163.1| ATAGTACTCTATTGAGTGTCCACAGAAAA-AACAATCTTCTGTGTTGAGATGGATTTGTA

JPNUV1|AB863164.1| ATAGTACTCTATTGAGTATCCACAGAAAA-AACAATCTTCTGTGTTGAGATGGATCTGTA

JPNUV26|AB863165.1| ATAGTACTCTATTGAGTATCCACAGAAAA-AACAATCTTCTGTGTTGAGATGGATCTGTA

TUR1|AB863166.1| ATAGTACTCTATTGAGTATCCACAGATAA-AATAACCTTCTGTGTTGAGATGGATTTGTA

TUR2|AB863167.1| ATAGTACTCTATTGAGTATCCACAGATAA-AATAATCTTCTGTGTTGAGATGGATTTGTA

TUR4|AB863168.1| ATAGTACTCTATTGAGTATCCACAGATAA-AATAATCTTCTGTGTTGAGATGGATTTGTA

TUR5|AB863169.1| AAAGTACTCTATTGAGTATCCACAGATAA-AATAATCTTCTGTGTTGAGATGGATTTGTA

TUR12|AB863170.1| ATAGTACTCTATTGAGTATCCACAGATAA-AATAATTTTCTGTGTTGAGATGGATTTGTA

TUR34|AB863171.1| ATAGTACTCTATTGAGTATCCACAGATAA-AATAATCTTCTGTGTTGAGATGGATTTGTA

TUR50|AB863172.1| ATAGTACTCTATTGAGTATCCACAGAAAA-AACAATCTTCTGTGTTGAGATGGATTTGTA

TUR59|AB863173.1| ATAGTACTCTACTGAGTATCCACAGATAA-GATAACCTTCTGTGTTGAGATGGATTTGTA

TUR69|AB863174.1| ATAGTACTCTATCGAGTATCCACAGAAAA-AATAATCTTCTGTGTTGAGATGGATTTGTA

TUR81|AB863175.1| ATAGTACTCTATTGAGTATCCACAGAAAA-AATAATCTTCTGTGTTGAGATGGATTTGTA

TUR84|AB863176.1| ATAGTACTCTATTGAGTATCCACAGAAAA-AATAATCTTCTGTGTTGAGATGGACTTGTA

TUR94|AB863177.1| ATAGTACTCTATTGAGTATCCACAGATAA-AATAATCTTCTGTGTTGAGATGGATTTGTA

TUR213|AB863178.1| ATAGTACTCTATCGAGTATCCACAGATAA-AATAATCTTCTGTGTTGAGATGGATTTGTA

TUR214|AB863179.1| ATAGTACTCTATTGAGTATCCACAGATAA-AATAACCTTCTGTGTTGAGATGGATTTGTA

TUR216|AB863180.1| ATAGTAGTCTATTGAGTATCCACAGATAA-AATAATCTTCTGTGTTGAGATGGATTTGTA

TUR220|AB863181.1| ATAGTACTCTATTGAGTATCCACAGAAAA-AATAATCTTCTGTGTTGAGATGGACTTGTA

TUR239|AB863182.1| ATAGTACTCTATTGAGTATCCACAGATAA-AATAACCTTCTGTGTCGAGATGGATCTGTA

TUR244|AB863183.1| ATAGTACTCTATTGAGTATCCACAGATAA-AATAACCTTCTGTGTTGAGATGGATTTGTA

TUR249|AB863184.1| ATAGTACTCTATCGAGTATCCACAGATAA-AATAATCTTCTGTGTTGAGATGGATTTGTA

TUR263|AB863185.1| ATAGTACTCTATCGAGTATCCACAGATAA-AATAATCTTCTGTGTTGAGATGGATTTGTA

TUR278|AB863186.1| ATAGTACTCTATTGAGTATCCACAGAAAA-AACAATCTTCTGTGTTGAGATGGATTTGTA

TUR279|AB863187.1| ATAGTACTCTATTGAGTATCCACAGAAAA-AATAATCTTCTGTGTTGAGATGGATTTGTA

TUR285|AB863188.1| ATAATACTCTATTGAGTATCCACAGATAA-AATAATCTTCTGTGTTGAGATGGATTTGTA

TUR289|AB863189.1| ATAGTACTCTATTGAGTATCCACAGAAAA-AATAATCTTCTGTGTTGAGATGGATTTGTA

TUR303|AB863190.1| ATAGTACTCTATTGAGTATCCACAGATAA-AATAATCTTCTGTGTTGAGATGGATTTGTA

TUR306|AB863191.1| ATAGTACTCTATTGAGTATCCACAGATAA-AATAATCTTCTGTGTTGAGATGGATTTGTA

Cabb-S|NC_001497.1| TCCAGAAGAAAATACCCAAAGCGAGCAATCGCAGAATTCTGAAAATAATATGCAAATATT

CRO180A|AB863192.1| TCCAGAAGAAAACACCCAAAGCGAGCAATCGCAAAATTCTGAAAATAATATGCAAATATT

GRC83|AB863193.1| TCCAGAAGAAAACACCCAAAGCGAGCAATCGCAAAATTCTGAAAATAATATGCAAATATT

GRC84B|AB863194.1| TCCAGAAGAAAACACCCAAAGCGAGCAATCGCAAAATTCTGAAAATAATATGCAAATATT

GRC86B|AB863195.1| TCCAGAAGAAAATACCCAAAGCGAGCAATCGCAAAATTCTGAAAATAATATGCAAATATT

GRC86D|AB863196.1| TCCAGAGGAAAACACCCAAAGCGAGCAATCGCAAAATTCTGAAAATAATATGCAAATATT

GRC87E|AB863197.1| TCCAGAAGAAAACACCCAAAGCGAGCAATCGCAAAATTCTGAAAATAATATGCAAATATT

GRC87G|AB863198.1| TCCAGAAGAAAACACCCAAAGCGAGCAATCGCAAAATTCTGAAAATAATATGCAAATATT

GRC91B|AB863199.1| TCCAGAAGAAAACACCCAAAGCGAGCAATCGCAAAATTCTGAAAATAATATGCAAATATT

GRC92A|AB863200.1| TCCAGAAGAAAACACCCAAAGCGAGCAATCGCAAAATTCTGAAAATAATATGCAAATATT

GRC92C|AB863201.1| TCCAGAAGAAAACACCCAAAGCGAGCAATCGCAAAATTCTGAAAATAATATGCAAATATT

GRC92D|AB863202.1| TCCAGAAGAAAACACCCAAAGCGAGCAATCGCAAAATTCTGAAAATAATATGCAAATATT

IRN1|AB863136.1| TCCAGAAGAAAATACCCAAAGCGAGCAATCGCAAAATTCTGAAAA---TATGCAAATATT

IRN2|AB863137.1| TCCAGAAGAAAATACCCAAAGCGAGCAATCGCAAAATTCTGAAAATAATATGCAAATATT

IRN3|AB863138.1| TCCAGAAGAAAATACCCAAAGCGAGCAATCGCAAAATTCTGAAAA---TATGCAAATATT

IRN4|AB863139.1| TCCAGAAGAAAATACCCAAAGCGAGCAATCGCAAAATTCTGAAAATAATATGCAAATATT

IRN5|AB863140.1| TCCAGAAGAAAACACCCAAAGCGAGCAATCGCAAAATTCTGTAAAAAATATGCAAATATT

IRN6|AB863141.1| TCCAGAAGAAAATACCCAAAGCGAGCAATCGCAAAATTCTGAAAATAATATGCAAATATT

IRN7|AB863142.1| TCCAGAAGAAAATACCCAAAGCGAGCAATCGCAAAATTCTGAAAATAATATGCAAATATT

IRN8|AB863143.1| TCCAGAAGAAAACACCCAAAGCGAGCAATCGCAAAATTCTGTAAATAATATGCAAATATT

IRN9|AB863144.1| TCCAGAAGAAAATACCCAAAGCGAGCAATCGCAAAATTCTGAAAATAATATGCAAATATT

IRN10|AB863145.1| TCCAGAAGAAAATACCCAAAGCGAGCAATCGCAAAATTCTGAAAATAATATGCAAATATT

IRN11|AB863146.1| TCCAAAAGAAAACACCCAAAGCGAGCAATCGCAAAATTCTGAAAATAATATGCAAATATT

IRN12|AB863147.1| TCCAGAAGAAAATACCCAAAGCGAGCAATCGCAAAATTCTGAAAACAATATGCAAATATT

IRN13|AB863148.1| TCCAGAAGAAAATACCCAAAGCGAGCAATCGCAAAATTCTGAAGATAATATGCAAATATT

IRN14|AB863149.1| TCCAGAAGAAAATACCCAAAGCGAGCAATCGCAAAATTCTGAAAATAATATGCAAATATT

IRN15|AB863150.1| TCCAGAAGAAAACACCCAAAGCGAGCAATCGCAAAATTCTGAAAATAATATGCAAATATT

IRN16|AB863151.1| TCCAGAAGAAAACACCCAAAGCGAGCAATCGCAAAATTCTGAAAATAATATGCAAATATT

IRN17|AB863152.1| TCCAGAAGAAAACACCCAAAGCGAGCAATCGCAAAATTCTGAAAATAATATGCAAATATT

IRN18|AB863153.1| TCCAGAAGAAAATACCCAAAGCGAGCAATCGCAAAATTCTGAAAATAATATGCAAATATT

IRN19|AB863154.1| TCCAGAAGAAAATACCCAAAGCGAGCAATCGCAAAATTCTGAAAATAATATGCAAATATT

IRN20|AB863155.1| TCCAGAAGAAAACACCCAAAGCGAGCAATCGCAAAATTCTGAAAATAATATGCAAATATT

IRN21|AB863156.1| TCCAGAAGAAAATATCCAAAGCGAGCAATCGCAAAATTCTGAAAATAATATGCAAATATT

JPNHGB340|AB863157.1| TCCAGAAGAAAACACCCAAAGCGAGCAATCGCAAAATTCTGAAAATAATATGCAAATATT

JPNKWB778|AB863158.1| TCCAGAAGAAAATACCCAAAGCGAGCAATCGCAAAATTCTGAAAATAATATGCAAATATT

JPNM|AB863159.1| TCCAGAAGAAAATACCCAAAGCGAGCAATCGCAAAATTCTGAAAATAATATGCAAATATT

JPNN|AB863160.1| TCCAGAAGAAAATACCCAAAGCGAGCAATCGCAGAATTCTGAAAAAAATATGCAAATATT

JPNS1|AB863161.1| TCCAGAAGAAAATACCCAAAGCGAGCAATCGCAGAATTCTGAAAATAATATGCAAATATT

JPNS2|AB863162.1| TCCAGAAGAAAATACCCAAAGCGAGCAATCGCAGAATTCTGAAAATAATATGCAAATATT

JPNTKD762|AB863163.1| TCCAGAAGAAAATACCCAAAGCGAGCAATCGCAAAATTCTGAAAATAATATGCAAATATT

JPNUV1|AB863164.1| TCCAGAAGAAAATACCCAAAGCGAGCAATCGCAAAATTCTGAAAATAATATGCAAATATT

JPNUV26|AB863165.1| TCCAGAAGAAAATACCCAAAGCGAGCAATCGCAAAATTCTGAAAATAATATGCAAATATT

TUR1|AB863166.1| TCCAGAAGAAAATACCCAAAGCGAGCAATCGCAGAATTCTGAAAATAATATGCAAATATT

TUR2|AB863167.1| TCCAGAAGAAAATACCCAAAGTGAGCAATCGCAAAATTCTGAAAATAATATGCAAATATT

TUR4|AB863168.1| TCCAGAAGAAAATACCCAAAGTGAGCAATCGCAAAATTCTGAAAATAATATGCAAATATT

TUR5|AB863169.1| TCCAGAAGAAAATACCCAAAGCGAGCAATCGCAAAATTCTGAAAATAATATGCAAATATT

TUR12|AB863170.1| TCCAGAAGAAAATACCCAAAGCGAGCAATCGCAGAATTCTGAGAATAATATGCAAATATT

TUR34|AB863171.1| TCCAGAAGAAAATACCCAGAGCGAGCAATCGCAGAATTCTGAAAATAATATGCAAATATT

TUR50|AB863172.1| TCCAGAAGAAAATACCCAGAGCGAGCAATCGCAGAATTCTGAAAATAATATGCAAATATT

TUR59|AB863173.1| TCCAGAAGAAAATACCCAAAGTGAGCAATCGCAGAATTCTGAAAATAATATGCAAATATT

TUR69|AB863174.1| TCCAGAAGAAAATACCCAAAGCGAGCAATCGCAGAATTCTGAAAATAATATGCAAATATT

TUR81|AB863175.1| TCCAGAAGAAAACACCCAAAGCGAGCAATCGCAAAATTCTGAAAATAATATGCAAATATT

TUR84|AB863176.1| TCCAGAAGAAAACACCCAAAGCGAGCAATCGCAAAATTCTGAAAATAATATGCAAATATT

TUR94|AB863177.1| TCCAGAAGAAAATACCCAAAGCGAGCAATCGCAGAATTCTGAAAATAATATGCAAATATT

TUR213|AB863178.1| TCCAGAAGAAATTACCCAAAGCGAGCAATCGCAAAATTCTGAAAATAATATGCAAATATT

TUR214|AB863179.1| TCCAGAAGAAATTACCCAAAGCGAGCAATCGCAAAATTCTGAAAATAATATGCAAATATT

TUR216|AB863180.1| TCCAGAAGAAAATACCCAAAGCGAGCAATCGCATAATTCTGAAAATAATATGCAAATATT

TUR220|AB863181.1| TCCAGAAGAAAACACCCAAAGCGAGCAATCGCAAAATTCTGAAAATAATATGCAAATATT

TUR239|AB863182.1| TCCAGAAGAAAATACCCAAAGCGAGCAATCGCAAAATTCTGAAAATAATATGCAAATATT

TUR244|AB863183.1| TCCAGAAGAAAATACCCAAAGCAAGCAATCGCAAAATTCTGAAAATAATATGCAAATATT

TUR249|AB863184.1| TCCAGAAGAAAATACCCAAAGCGAGCAATCGCAGAATTCTGAAAATAATATGCAAATATT

TUR263|AB863185.1| TCCAGAAGAAAACACCCAAAGCGAGCAATCGCAGAATTCTGAAAATAATATGCAAATATT

TUR278|AB863186.1| TCCAGAAGAAAACACCCAAAGCGAGCAATCGCAAAATTCTGAAAATAATATGCAAATATT

TUR279|AB863187.1| TCCAGAAGAAAACACCCAAAGCGAGCAATCGCAAAATTCTGAAAATAATATGCAAATATT

TUR285|AB863188.1| TCCAGAAGAAAATACCCAAAGCGAGCAATCGCAGAATTCTGAAAATAATATGCAAATATT

TUR289|AB863189.1| TCCAGAAGAAAACACCCAAAGCGAGCAACCGCAAAATTCTGAAAATAATATGCAAATATT

TUR303|AB863190.1| TTCAGAAGAAAATACCCAAAGCGAGCAATCGCAGAATTCTGAAAATAATATGCAAATATT

TUR306|AB863191.1| TCCAGAAGAAAATACCCAAAGCGAGCAATCGCATAATTCTGAAAATAATATGCAAATATT

Cabb-S|NC_001497.1| TAAATCAGAAAATTCGGATGGATTCTCCTCCGATCTAATGATCTCAAACGATCAATTAAA

CRO180A|AB863192.1| TAAATCAGAAACTTCGGATGGATTCTCCTCCGATCTAATGATCTCAAACGATCAATTAAA

GRC83|AB863193.1| TAAATCAGAAAATTCGGATGGATTCTCCTCCGATCTAATGATCTCTAATGATCAATTAAA

GRC84B|AB863194.1| TAAATCAGAAAATTCGGATGGATTCTCCTCCGATCTAATGATCTCTAATGATCAATTAAA

GRC86B|AB863195.1| TAAATCAGAAAATTCGGATGGATTCTCCTCCGATCTAATGATCTCTAATGATCAATTAAA

GRC86D|AB863196.1| TAAATCAGAAACTTCGGATGGATTCTCCTCCGATCTAATGATCTCTAATGATCAATTAAA

GRC87E|AB863197.1| TAAATCAGAAAATTCGGATGGATTCTCCTCCGATCTAATGATCTCAAACGATCAATTAAA

GRC87G|AB863198.1| TAAATCAGAAAATTCGGATGGATTCTCCTCCGATCTAATGATCTCAAACGATCAATTAAA

GRC91B|AB863199.1| TAAATCAGAAAATTCGGATGGATTCTCCTCCGATCTAATGATCTCAAACGATCAATTAAA

GRC92A|AB863200.1| TAAATCAGAAACTTCGGATGGATTCTCCTCCGATCTAATGATCTCAAACGATCAATTAAA

GRC92C|AB863201.1| TAAATCAGAAAATTCGGATGGATTCTCCTCCGATCTAATGATCTCTAATGATCAATTAAA

GRC92D|AB863202.1| TAAATCAGAAAATTCGGATGGATTCTCCTCCGATCTAATGATCTCTAATGATCAATTAAA

IRN1|AB863136.1| TAAATCAGAAAATTCGGATGGATTCTCCTCCGATCTAATGATCTCTAATGATCAATTAAA

IRN2|AB863137.1| TAAATCAGAAACTTCGGATGGATTCTCCTCCGATTTAATGATCTCTAATGATCAATTAAA

IRN3|AB863138.1| TAAATCAGAAAATTCGGATGGATTCTCCTCCGATCTAATGATCTCTAATGATCAATTAAA

IRN4|AB863139.1| TAAATCAGAAACTTCGGATGGATTCTCCTCCGATTTAATGATCTCTAATGATCAATTAAA

IRN5|AB863140.1| TAAATCAGAAACTTCGGATGGATTCTCCTCCGATTTAATGATCTCTAATGATCAATTAAA

IRN6|AB863141.1| TAAATCAGAAACTTCGGATGGATTCTCCTCCGATTTAATGATCTCTAATGATCAATTAAA

IRN7|AB863142.1| TAAATCAGAAACTTCGGATGGATTCTCCTCCGATTTAATGATCTCTAATGATCAATTAAA

IRN8|AB863143.1| TAAATCAGAAACTTCGGATGGATTCTCCTCCGATTTAATGATCTCTAATGATCAATTAAA

IRN9|AB863144.1| TAAATCAGAAACTTCGGATGGATTTTCCTCCGATTTAATGATCTCTAATGATCAATTAAA

IRN10|AB863145.1| TAAATCAGAAACTTCGGATGGATTCTCCTCCGATTTAATGATCTCTAATGATCAATTAAA

IRN11|AB863146.1| TAAATCAGAAACTTCGGATGGATTCTCCTCCGATCTAATGATCTCTAATGATCAATTAAA

IRN12|AB863147.1| TAAATCAGAAACTTTGGATGGATTTTCCTCCAATCTAATGATCTCTAATGATCAATTAAA

IRN13|AB863148.1| TAAATCAGAAACTTCGGATGGATTCTCCTCCGATTTAATGATCTCTAATGATCAATTAAA

IRN14|AB863149.1| TAAATCAGAAACTTCGGATGGATTCTCCTCCGATTTAATGATCTCTAATGATCAATTAAA

IRN15|AB863150.1| TAAATCAGAAACTTCGGATGGATTCTCCTCCGATTTAATGATCTCTAATGATCAATTAAA

IRN16|AB863151.1| TAAATCAGAAACTTCGGATGGATTCTCCTCCGATTTAATGATCTCTAATGATCAATTAAA

IRN17|AB863152.1| TAAATCAGAAACTTCGGATGGATTCTCCTCCGATTTAATGATCTCTAATGATCAATTAAA

IRN18|AB863153.1| TAAATCAGAAAATTCGGATGGATTCTCCTCCGATCTAATGATCTCTAACGATCAATTAAA

IRN19|AB863154.1| TAAATCAGAAAATTCGGATGGATTCTCCTCCGATCTAATGATCTCTAATGATCAATTAAA

IRN20|AB863155.1| TAAATCAGAAACTTCGGATGGATTCTCCTCCGATTTAATGATCTCTAATGATCAATTAAA

IRN21|AB863156.1| TAAATCAGAAACTTCGGATGGATTCTCCTCCGATTTGATGATCTCTAATGATCAATTAAA

JPNHGB340|AB863157.1| TAAGTCAGAAAATTCGGATGGATTCTCCTCCGATCTAATGATCTCAACCGATCAATTAAA

JPNKWB778|AB863158.1| TAAGTCAGAAAATTCGGATGGATTCTCCTCCGATCTAATGATCTCAAACGATCAATTAAA

JPNM|AB863159.1| TAAGTCAGAAAATTCGGATGGATTCTCCTCCGATCTAATGATCTCAAACGATCAATTAAA

JPNN|AB863160.1| TAAATCAGAAAATTCGGACGGATTTTCCTCCGATCTAATGATCTCAAACGATCAATTAAA

JPNS1|AB863161.1| TAAATCAGAAAATTCGGACGGATTTTCCTCCGATCTAATGATCTCAAACGATCAATTAAA

JPNS2|AB863162.1| TAAATCAGAAAATTCGGACGGATTTTCCTCCGATCTAATGATCTCAAACGATCAATTAAA

JPNTKD762|AB863163.1| TAAATCAGAAAATTCGGATGGATTCTCCTCCGATCTAATGATCTCAAACGATCAATTAAA

JPNUV1|AB863164.1| TAAGTCAGAAAATTCGGATGGATTCTCCTCCGATCTAATGATCTCAAACGATCAATTAAA

JPNUV26|AB863165.1| TAAGTCAGAAAATTCGGATGGATTCTCCTCCGATCTAATGATCTCAAACGATCAATTAAA

TUR1|AB863166.1| TAAATCAGAAAATTCGGATGGATTCTCCTCCGATCTAATGATCTCTAATGATCAATTAAA

TUR2|AB863167.1| TAAATCAGAAACTTCGGATGGATTCTCCTCCGATCTAATGATCTCTAATGATCAATTAAA

TUR4|AB863168.1| TAAATCAGAAACTTCGGATGGATTCTCCTCCGATCTAATGATCTCTAATGATCAATTAAA

TUR5|AB863169.1| TAAATCAGAAACTTCGGATGGATTCTCCTCCGATCTAATGATCTCTAATGATCAATTAAA

TUR12|AB863170.1| TAAATCAGAAACTTCGGATGGATTCTCCTCCGATCTAATGATCTCTAATGATCAATTAAA

TUR34|AB863171.1| TAAATCAGAAAATTCAGATGGATTCTCCTCTGACCTAATGATCTCAAACAATCAACTAAA

TUR50|AB863172.1| TAAATCAGAAAATTCAGATGGATTCTCCTCTGACCTAATGATCTCAAACGATCAACTAAA

TUR59|AB863173.1| TAAATCAGAAAATTCGGATGGATTCTCCTCCGATCTAATGATCTCAAACGATCAATTAAA

TUR69|AB863174.1| TAAATCAGAAAATTCGGATGGATTCTCCTCCGATCTAATGATCTCTAATGATCAATTAAA

TUR81|AB863175.1| TAAATCAGAAAATTCGGATGGATTTTCCTCCGATCTAATGATCTCTAATGATCAATTAAA

TUR84|AB863176.1| TAAATCAGAAAATTCGGATGGATTTTCCTCCGATCTAATGATCTCTAATGATCAATTAAA

TUR94|AB863177.1| TAAATCAGAAAATTCGGACGGATTCTCCTCCGATCTAATGATCTCAAACGATCAATTAAA

TUR213|AB863178.1| TAAATCAGAAAATTCGGACGGATTCTCTTCCGATCTAATGATCTCAAACGATCAATTAAA

TUR214|AB863179.1| TAAATCAGAAAATTCGGACGGATTCTCTTCCGATCTAATGATCTCAAACGATCAATTAAA

TUR216|AB863180.1| TAAATCAGAAAATTCGGATGGATTCTCCTCCGATCTAATGATCTCAAACGATCAATTAAA

TUR220|AB863181.1| TAAATCAGAAAATTCGGATGGATTTTCCTCCGATCTAATGATCTCTAATGATCAATTAAA

TUR239|AB863182.1| TAAATCAGAAAATTCGGACGGATTCTCCTCCGATCTAATGATCTCAAACGATCAATTAAA

TUR244|AB863183.1| TAAATCAGAAAATTCGGACGGATTCTCCTCCGATCTAATGATCTCTAACGATCAATTAAA

TUR249|AB863184.1| TAAATCAGAAAATTCGGATGGATTCTCCTCCGATCTAATGATCTCTAACGATCAATTAAA

TUR263|AB863185.1| TAAATCAGAAAATTCGGATGGATTCTCCTCCGATCTAATGATCTCTAATGATCAATTAAA

TUR278|AB863186.1| TAAATCAGAAAATTCGGATGGATTCTCCTCCGATCTAATGATCTCAAACGATCAATTAAA

TUR279|AB863187.1| TAAATCAGAAAATTCGGATGGATTTTCCTCCGATCTAATGATCTCTAATGATCAATTAAA

TUR285|AB863188.1| TAAATCAGAAAATTCGGATGGATTCTCCTCCGATCTAATGATCTCTAACGATCAATTAAA

TUR289|AB863189.1| TAAATCAGAAAATTCGGATGGATTTTCCTCCGATCTAATGATCTCTAATGATCAATTAAA

TUR303|AB863190.1| TAAATCAGAAAATTCAGACGGATTCTCTTCCGATCTAATGATCTCAAACGATCAATTAAA

TUR306|AB863191.1| TAAATCAGAAAATTCGGATGGATTCTCCTCCGATCTAATGATCTCAAACGATCAATTAAA

Cabb-S|NC_001497.1| AAATATCTCTAAAACCCAATTAACCTTGGAGAAAGAAAAGATATTTAAAATGCCTAACGT

CRO180A|AB863192.1| AAATATCTCAAAAACTCAATTAACTTTGGAGAAAGAAAAGATATTTAAAATGCCTAACGT

GRC83|AB863193.1| AAATATCTCAAAAACCCAATTAACTTTGGAGAAAGAAAAGATATTTAAAATGCCTAACGT

GRC84B|AB863194.1| AAATATCTCAAAAACCCAATTAACTTTGGAGAAAGAAAAGATATTTAAAATGCCTAACGT

GRC86B|AB863195.1| AAATATCTCAAAAACCCAATTAACTTTGGAGAAAGAAAAGATATTTAAAATGCCTAACGT

GRC86D|AB863196.1| AAATATCTCAAAAACCCAATTAACTTTGGAGAAAGAAAAGATATTTAAAATGCCTAACGT

GRC87E|AB863197.1| AAATATCTCAAAAACCCAATTAACTTTGGAGAAAGAAAAGATATTTAAAATGCCTAACGT

GRC87G|AB863198.1| AAATATCTCAAAAACCCAATTAACTTTGGAGAAAGAAAAGATATTTAAAATGCCTAACGT

GRC91B|AB863199.1| AAATATCTCAAAAACCCAATTAACTTTGGAGAAAGAAAAGATATTTAAAATGCCTAACGT

GRC92A|AB863200.1| AAATATCTCAAAAACCCAATTAACTTTGGAGAAAGAAAAGATATTTAAAATGCCTAACGT

GRC92C|AB863201.1| AAATATCTCAAAAACCCAATTAACTTTGGAGAAAGAAAAGATATTTAAAATGCCTAACGT

GRC92D|AB863202.1| AAATATCTTAAAAACCCAATTAACTTTGGAGAAAGAAAAGATATTTAAAATGCCTAACGT

IRN1|AB863136.1| AAATATCTCAAAAACCCAATTAACTTTGGAGAAAGAAAAGATATTTAAAATGCCTAACGT

IRN2|AB863137.1| AAATATCTCAAAAACCCAATTAACTTTGGAGAAAGAAAAGATATTTAAAATACCTAACGT

IRN3|AB863138.1| AAATATCTCAAAAACCCAATTAACTTTGGAGAAAGAAAAGATATTTAAAATGCCTAACGT

IRN4|AB863139.1| AAATATCTCAAAAACCCAATTAACCTTGGAGAAAGAAAAGATATTTAAAATGCCCAACGT

IRN5|AB863140.1| AAATATCTCAAAAACCCAATTAACCTTGGAAAAAGATAAGATATTTAAAATGCCTAACGT

IRN6|AB863141.1| AAATATCTCAAAAACCCAATTAACCTTGGAAAAAGAAAAGATATTTAAAATGCCTAACGT

IRN7|AB863142.1| AAATATCTCAAAAACCCAATTAACTTTGGAGAAAGAAAAGATATTTAAAATACCTAACGT

IRN8|AB863143.1| AAATATCTCAAAAACCCAATTAACCTTGGAAAAAGATAAGATATTTAAAATGCCTAACGT

IRN9|AB863144.1| AAATATCTCAAAAACCCAATTAACCTTGGAGAAAGAAAAGATATTTAAAATGCCTAACGT

IRN10|AB863145.1| AAATATCTCAAAAACCCAATTAACTTTGGAGAAAGAAAAGATATTTAAAATGCCTAACGT

IRN11|AB863146.1| AAATATCTCAAAAACCCAATTAACTTTGGAGAAAGAAAAGATATTTAAAATGCCTAACGT

IRN12|AB863147.1| AAATATCTCAAAAACCCAATTAACCTTGGAAAAAGAAAAGATATTTAAAATGCCTAACGT

IRN13|AB863148.1| AAATATCTCAAAAACCCAATTAACCTTGGAAAAAGAGAAGATATTTAAAATGCCTAACGT

IRN14|AB863149.1| AAATATCTCTAAAACCCAATTAACCTTGGAAAAAGAAAAGATATTTAAAATGCCTAACGT

IRN15|AB863150.1| AAATATCTCAAAAACCCAATTAACCTTGGAAAAAGAAAAGATATTTAAAATGCCTAACGT

IRN16|AB863151.1| AAATATCTCAAAAACCCAATTAACCTTGGAAAAAGAAAAGATATTTAAAATGCCTAACGT

IRN17|AB863152.1| AAATATCTCAAAAACCCAATTAACCTTGGAAAAAGAAAAGATATTTAAAATGCCTAACGT

IRN18|AB863153.1| AAATATCTCAAAAACCCAATTAACTTTGGAGAAAGAAAAGATATTTAAAATGCCTAACGT

IRN19|AB863154.1| AAATATCTCAAAAACCCAATTAACTTTGGAGAAAGAAAAGATATTTAAAATGCCTAACGT

IRN20|AB863155.1| AAATATCTCAAAAACCCAATTAACCTTGGAAAAAGAAAAGATATTTAAAATGCCTAACGT

IRN21|AB863156.1| AAATATCTCAAAAACCCAATTAACCTTGGAAAAAGAAAAGATATTTAAAATGCCTAACGT

JPNHGB340|AB863157.1| AAATATCTCTAAAACCCAATTAACTTTGGAAAAAGAAAAGATATTTAAAATGCCTAACGT

JPNKWB778|AB863158.1| AAATATCTCAAAAACCCAATTAACTTTGGAAAAAGAAAAGATATTTAAAATGCCTAACGT

JPNM|AB863159.1| AAATATCTCTAAAACCCAATTAACTTTGGAAAAAGAAAAGATATTTAAAATGCCTAACGT

JPNN|AB863160.1| AAATATCTCTAAAACCCAATTAACTTTGGAAAAAGAAAAGATATTTAAAATGCCTAACGT

JPNS1|AB863161.1| AAATATCTCTAAAACCCAATTAACTTTGGAAAAAGAAAAGATATTTAAAATGCCTAACGT

JPNS2|AB863162.1| AAATATCTCTAAAACCCAATTAACTTTGGAAAAAGAAAAGATATTTAAAATGCCTAACGT

JPNTKD762|AB863163.1| AAATATCTCTAAAACCCAATTAACTTTGGAAAAAGAAAAGATATTTAAAATGCCTAACGT

JPNUV1|AB863164.1| AAATATCTCTAAAACCCAATTAACTTTGGAAAAAGAAAAGATATTTAAAATGCCTAACGT

JPNUV26|AB863165.1| AAATATCTCTAAAACCCAATTAACTTTGGAAAAAGAAAAGATATTTAAAATGCCTAACGT

TUR1|AB863166.1| AAATATCTCAAAAACCCAATTAACTTTGGAAAAAGAAAAGATATTTAAAATGCCTAACGT

TUR2|AB863167.1| AAATATCTCGAAAACCCAATTAACTTTGGAGAAAGAAAAGATATTCAAAATGCCTAACGT

TUR4|AB863168.1| AAATATCTCGAAAACCCAATTAACTTTGGAGAAAGAAAAGATATTCAAAATACCTAACGT

TUR5|AB863169.1| AAATATCTCGAAAACCCAATTAACTTTGGAGAAAGAAAAGATATTCAAAATGCCTAACGT

TUR12|AB863170.1| AAATATCTCAAAAACCCAATTAACTTTGGAAAAAGAAAAGATATTTAAAATGCCTAACGT

TUR34|AB863171.1| AAATATCTCAAAAACCCAATTAACTTTGGAGAAAGAAAAGATATTTAAAATGCCTAACGT

TUR50|AB863172.1| AAATATCTCAAAAACCCAATTAACTTTGGAGAAAGAAAAGATATTTAAAATGCCTAACGT

TUR59|AB863173.1| AAATATCTCAAAAACCCAATTAACTTTGGAAAAAGAAAAGATATTTAAAATGCCTAACGT

TUR69|AB863174.1| AAATATCTCAAAAACCCAATTAACTTTGGAAAAAGAAAAGATATTTAAAATGCCTAACGT

TUR81|AB863175.1| AAATATCTCAAAAACCCAATTAACTTTGGAAAAAGAAAAGATATTTAAAATGCCTAACGT

TUR84|AB863176.1| AAATATCTCAAAAACCCAATTAACTTTGGAAAAAGAAAAGATATTTAAAATGCCTAACGT

TUR94|AB863177.1| AAATATCTCAAAAACCCAATTAACTTTGGAAAAAGAAAAGATATTTAAAATGCCTAACGT

TUR213|AB863178.1| AAATATCTCAAAAACCCAATTAACTTTGGAAAAAGAAAAGATATTTAAAATGCCTAACGT

TUR214|AB863179.1| AAATATCTCAAAAACCCAATTAACTTTGGAAAAAGAAAAGATATTTAAAATGCCTAACGT

TUR216|AB863180.1| AAATATCTCTAAAACCCAATTAACCTTGGAAAAAGAAAAGATATTTAAAATGCCTAACGT

TUR220|AB863181.1| AAATATCTCAAAAACCCAATTAACTTTGGAAAAAGAAAAGATATTTAAAATGCCTAACGT

TUR239|AB863182.1| AAATATCTCAAAAACCCAATTAACTTTGGAAAAAGAAAAGATATTTAAAATGCCTAACGT

TUR244|AB863183.1| AAATATCTCAAAAACCCAATTAACTTTGGAAAAAGAAAAGATATTTAAAATGCCTAACGT

TUR249|AB863184.1| AAATATCTCAAAAACCCAGTTAACTTTGGAAAAAGAAAAGATATTTAAAATGCCTAACGT

TUR263|AB863185.1| AAATATCTCGAAAACCCAATTAACTTTGGAAAAAGAAAAGATATTTAAAATGCCTAACGT

TUR278|AB863186.1| AAATATCTCAAAAACCCAATTAACTTTGGAGAAAGAAAAAATATTTAAAATGCCTAACGT

TUR279|AB863187.1| AAATATCTCAAAAACCCAATTAACTTTGGAAAAAGAAAAGATATTTAAAATGCCTAACGT

TUR285|AB863188.1| AAATATCTCAAAAACCCAATTAACTTTGGAAAAAGAAAAGATATTTAAAATGCCTAACGT

TUR289|AB863189.1| AAATATCTCAAAAACCCAATTAACTTTGGAAAAAGAAAAGATATTTAAAATGCCTAACGT

TUR303|AB863190.1| AAATATCTCAAAAACCCAATTAACTTTGGAGAAAGAAAAGATATTTAAAATGCCTAACGT

TUR306|AB863191.1| AAATATCTCAAAAACCCAATTAACTTTGGAGAAAGAAAAGATATTTAAAATGCCTAACGT

Cabb-S|NC_001497.1| TTTATCTCAAGTTATGAAAAAAGCGTTTAGCAGGAAAAACGAGATTCTCTACTGCGTCTC

CRO180A|AB863192.1| TTTATCTCAAGTTATGAAAAAAGCGTTTAGCAGGAAAAACGAGATTCTCTACTGCGTCTC

GRC83|AB863193.1| TTTATCTCAAGTTATGAAAAAAGCGTTTAGCAGGAAAAACGAGATTCTCTACTGCGTCTC

GRC84B|AB863194.1| TTTATCTCAAGTTATGAAAAAAGCGTTTAGCAGGAAAAACGAGATTCTCTACTGCGTCTC

GRC86B|AB863195.1| TTTATCTCAAGTTATGAAAAAAGCGTTTAGCAGGAAAAACGAGATTCTCTACTGCGTCTC

GRC86D|AB863196.1| TTTATCTCAAGTTATGAAAAAAGCGTTTAGCAGGAAAAACGAGATTCTCTACTGCGTCTC

GRC87E|AB863197.1| TTTATCTCAAGTTATGAAAAAAGCGTTTAGCAGGAAAAACGAGATTCTCTACTGCGTCTC

GRC87G|AB863198.1| TTTATCTCAAGTTATGAAAAAAGCGTTTAGCAGGAAAAACGAGATTCTCTACTGCGTCTC

GRC91B|AB863199.1| TTTATCTCAAGTTATGAAAAAAGCGTTTAGCAGGAAAAACGAGATTCTCTACTGCGTCTC

GRC92A|AB863200.1| TTTATCTCAAGTTATGAAAAAAGCGTTTAGCAGGAAAAACGAGATTCTCTACTGCGTCTC

GRC92C|AB863201.1| TTTATCTCAAGTTATGAAAAAAGCGTTTAGCAGGAAAAACGAGATTCTCTACTGCGTCTC

GRC92D|AB863202.1| TTTATCTCAAGTTATGAAAAAAGCGTTTAGCAGGAAAAACGAGATTCTCTACTGCGTCTC

IRN1|AB863136.1| CTTATCTCAAGTTATGAAAAAAGCGTTTAGCAGGAAAAACGAGATTCTTTACTGCGTCTC

IRN2|AB863137.1| TTTATCTCAAGTTATGAAAAAAGCGTTTAGCAGGAAAAACGAGATTCTCTACTGCGTCTC

IRN3|AB863138.1| CTTATCTCAAGTTATGAAAAAAGCGTTTAGCAGGAAAAACGAGATTCTTTACTGCGTCTC

IRN4|AB863139.1| TTTATCTCAAGTTATGAAAAAAGCGTTTAGCAGGAAAAACGAGATTCTCTACTGCGTCTC

IRN5|AB863140.1| TTTATCTCAAGTTATGAAAAAAGCGTTTAGCAGGAAAAACGAGATTCTCTACTGCGTCTC

IRN6|AB863141.1| TTTATCTCAAGTCATGAAAAAAGCGTTTAGCAGGAAAAACGAGATTCTCTACTGCGTCTC

IRN7|AB863142.1| TTTATCTCAAGTTATGAAAAAAGCGTTTAGCAGGAAAAACGAGATTCTCTACTGCGTCTC

IRN8|AB863143.1| TTTATCTCAAGTTATGAAAAAAGCGTTTAGCAGGAAAAACGAGATTCTCTACTGCGTCTC

IRN9|AB863144.1| TTTATCTCAAGTTATGAAAAAAGCGTTTAGCAGGAAAAATGAGATTCTCTACTGCGTCTC

IRN10|AB863145.1| TTTATCTCAAGTTATGAAAAAAGCGTTTAGCAGGAAAAACGAGATTCTCTACTGTGTCTC

IRN11|AB863146.1| TTTATCTCAAGTTATGAAAAAAGCGTTTAGCAGGAAAAACGAGATTCTTTACTGCGTCTC

IRN12|AB863147.1| TTTATCTCAAGTTATGAAAAAAGCGTTTAGCAGGAAAAACGAGATTCTCTACTGCGTCTC

IRN13|AB863148.1| TTTATCTCAAGTTATGAAAAAAGCGTTTAGCAGGAAAAACGAGATTCTCTACTGCGTCTC

IRN14|AB863149.1| TTTATCTCAAGTTATGAAAAAAGCGTTTAGCAGGAAAAACGAGATTCTCTACTGCGTCTC

IRN15|AB863150.1| TTTATCTCAAGTTATGAAAAAAGCGTTTAGCAGGAAAAACGAGATTCTCTACTGCGTCTC

IRN16|AB863151.1| TTTATCTCAAGTTATGAAAAAAGCGTTTAGCAGGAAAAACGAGATTCTCTACTGCGTCTC

IRN17|AB863152.1| TTTATCTCAAGTTATGAAAAAAGCGTTTAGCAGGAAAAACGAGATTCTCTACTGCGTCTC

IRN18|AB863153.1| TTTATCTCAAGTTATGAAAAAAGCGTTTAGCAGGAAAAACGAGATTCTCTACTGCGTCTC

IRN19|AB863154.1| TTTATCTCAAGTTATGAAAAAAGCGTTTAGCAGGAAAAACGAGATTCTCTACTGCGTCTC

IRN20|AB863155.1| TTTATCTCAAGTTATGAAAAAAGCGTTTAGCAGGAAAAACGAGATTCTCTACTGCGTCTC

IRN21|AB863156.1| TTTATCTCAAGTTATGAAAAAAGCGTTTAGCAGGAAAAACGAGATTCTCTACTGCGTCTC

JPNHGB340|AB863157.1| TTTATCTCAAGTTATGAAAAAAGCGTTTAGCAGGAAAAACGAGATTCTTTACTGCGTCTC

JPNKWB778|AB863158.1| TTTATCTCAAGTTATGAAAAAAGCGTTTAGCAGGAAAAATGAGATTCTTTACTGCGTCTC

JPNM|AB863159.1| TTTATCTCAAGTTATGAAAAAAGCGTTTAGCAGGAAAAATGAGATTCTTTACTGCGTCTC

JPNN|AB863160.1| TTTATCTCAAGTTGTGAAAAAAGCGTTTAGCAGGAAAAACGAGATTCTCTACTGCGTCTC

JPNS1|AB863161.1| TTTATCTCAAGTTATGAAAAAAGCGTTTAGCAGGAAAAACGAGATTCTCTACTGCGTCTC

JPNS2|AB863162.1| TTTATCTCAAGTTATGAAAAAAGCGTTTAGCAGGAAAAACGAGATTCTCTACTGCGTCTC

JPNTKD762|AB863163.1| TTTATCTCAAGTTATGAAAAAAGCGTTTAGCAGGAAAAATGAGATTCTTTACTGCGTCTC

JPNUV1|AB863164.1| TTTATCTCAAGTTATGAAAAAAGCGTTTAGCAGGAAAAATGAGATTCTTTACTGCGTCTC

JPNUV26|AB863165.1| TTTATCTCAAGTTATGAAAAAAGCGTTTAGCAGGAAAAATGAGATTCTTTACTGCGTCTC

TUR1|AB863166.1| TTTATCTCAAGTTATGAAAAAAGCGTTTAGCAGGAAAAACGAGATTCTCTACTGCGTCTC

TUR2|AB863167.1| TTTATCTCAAGTTATGAAAAAAGCGTTTAGCAGGAAAAATGAGATTCTCTACTGCGTCTC

TUR4|AB863168.1| TTTATCTCAAGTTATGAAAAAAGCGTTTAGCAGGAAAAATGAGATTCTCTACTGCGTCTC

TUR5|AB863169.1| TTTATCTCAAGTTATGAAAAAAGCGTTTAGCAGGAAAAACGAGATTCTTTACTGCGTCTC

TUR12|AB863170.1| TTTATCTCAAGTTATGAAAAAAGCGTTTAGCAGGAAAAACGAGATTCTCTACTGCGTCTC

TUR34|AB863171.1| TTTATCTCAAGTTATGAAAAAAGCGTTTAGCAGAAAAAACGAGATTCTTTACTGCGTCTC

TUR50|AB863172.1| TTTATCTCAAGTTATGAAAAAAGCGTTTAGCAGGAAAAACGAGATTCTTTACTGCGTCTC

TUR59|AB863173.1| TTTATCTCAAGTTATGAAAAAAGCGTTTAGCAGGAAAAACGAGATTCTTTACTGCGTCTC

TUR69|AB863174.1| TTTATCTCAAGTTATGAAAAAAGCGTTTAGCAGGAAAAACGAGATTCTTTACTGCGTCTC

TUR81|AB863175.1| TTTATCTCAAGTTATGAAAAAAGCGTTTAGCAGGAAAAACGAGATTCTTTACTGCGTCTC

TUR84|AB863176.1| TTTATCTCAAGTTATGAAAAAAGCGTTTAGCAGGAAAAACGAGATTCTTTACTGCGTCTC

TUR94|AB863177.1| TTTATCTCAAGTTATGAAAAAAGCGTTTAGCAGGAAAAACGAGATTCTTTACTGCGTCTC

TUR213|AB863178.1| TTTATCTCAAGTTATGAAAAAAGCGTTTAGCAGGAAAAACGAGATTCTCTACTGCGTCTC

TUR214|AB863179.1| TTTATCTCAAGTTATGAAAAAAGCGTTTAGCAGGAAAAACGAGATTCTCTACTGCGTCTC

TUR216|AB863180.1| TTTATCTCAAGTTATGAAAAAAGCGTTTAGCAGGAAAAACGAGATTCTTTACTGCGTCTC

TUR220|AB863181.1| TTTATCTCAAGTTATGAAAAAAGCGTTTAGCAGGAAAAACGAGATTCTTTACTGCGTCTC

TUR239|AB863182.1| TTTATCTCAAGTTATGAAAAAAGCGTTTAGCAGGAAAAACGAGATTCTTTACTGCGTCTC

TUR244|AB863183.1| TTTATCTCAAGTTATGAAAAAAGCGTTTAGCAGGAAAAACGAGATTCTTTACTGCGTCTC

TUR249|AB863184.1| TTTATCTCAAGTTATGAAAAAAGCGTTTAGCAGGAAAAACGAGATTCTTTACTGCGTCTC

TUR263|AB863185.1| TTTATCTCAAGTTATGAAAAAAGCGTTTAGCAGGAAAAACGAGATTCTTTACTGCGTCTC

TUR278|AB863186.1| TTTATCTCAAGTTATGAAAAAAGCGTTTAGCAGGAAAAACGAGATTCTCTACTGCGTCTC

TUR279|AB863187.1| TTTATCTCAAGTTATGAAAAAAGCGTTTAGCAGGAAAAACGAGATTCTTTACTGCGTCTC

TUR285|AB863188.1| TTTATCTCAAGTTATGAAAAAAGCGTTTAGCAGGAAAAACGAGATTCTTTACTGCGTCTC

TUR289|AB863189.1| TTTATCTCAAGTTATGAAAAAAGCGTTTAGCAGGAAAAACGAGATTCTTTACTGCGTCTC

TUR303|AB863190.1| TTTATCTCAAGTTATGAAAAAAGCGTTTAGCAGGAAAAACGAGATTCTCTACTGCGTCTC

TUR306|AB863191.1| TTTATCTCAAGTTATGAAAAAAGCGTTTAGCAGGAAAAACGAGATTCTTTATTGCGTCTC

Cabb-S|NC_001497.1| GACAAAAGAATTATCAGTGGACATTCACGATGCCACAGGTAAGGTATATCTTCCCTTAAT

CRO180A|AB863192.1| GACAAAGGAATTATCAGTGGACATTCATGATGCCACAGGTAAGGTATATCTCCCTTTAAT

GRC83|AB863193.1| GACAAAAGAATTGTCAGTGGACATTCACGATGCCACAGGTAAGGTATATCTCCCTTTAAT

GRC84B|AB863194.1| GACAAAAGAATTGTCAGTGGACATTCACGATGCCACAGGTAAGGTATATCTCCCTTTAAT

GRC86B|AB863195.1| GACAAAAGAATTATCAGTGGACATTCACGATGCCACAGGTAAGGTATATCTCCCTTTAAT

GRC86D|AB863196.1| GACAAAAGAATTATCAGTGGACATTCACGATGCCACAGGTAAGGTATATCTCCCTTTAAT

GRC87E|AB863197.1| GACAAAAGAATTATCAGTGGACATTCACGATGCCACAGGTAAAGTATATCTCCCTTTAAT

GRC87G|AB863198.1| GACAAAAGAATTATCAGTGGACATTCACGATGCCACAGGTAAAGTATATCTCCCTTTAAT

GRC91B|AB863199.1| GACAAAAGAATTATCAGTGGACATTCACGATGCCACAGGTAAAGTATATCTTCCTTTAAT

GRC92A|AB863200.1| GACAAAAGAATTATCAGTGGACATTCACGATGCCACAGGTAAGGTATATCTCCCTTTAAT

GRC92C|AB863201.1| GACAAAAGAATTATCAGTGGACATTCACGATGCCACAGGTAAAGTATATCTCCCTTTAAT

GRC92D|AB863202.1| GACAAAAGAATTATCAGTGGACATTCACGATGCCACAGGTAAGGTATATCTCCCTTTAAT

IRN1|AB863136.1| AACAAAAGAACTATCAGTGGACATTCATGATGCCACAGGTAAGGTATATCTTCCTTTGAT

IRN2|AB863137.1| GACAAAAGAATTATCAGTGGACATTCATGATGCCACAGGTAAGGTATATCTCCCTTTAAT

IRN3|AB863138.1| AACAAAAGAACTATCAGTGGACATTCATGATGCCACAGGTAAGGTATATCTTCCTTTGAT

IRN4|AB863139.1| GACAAAAGAATTATCAGTGGACATTCATGATGCCACAGGTAAAGTATATCTCCCTTTAAT

IRN5|AB863140.1| GACAAAAGAATTATCAGTGGACATTCATGATGCCACAGGTAAGGTATATCTCCCTTTAAT

IRN6|AB863141.1| GACAAAAGAATTATCAGTGGACATTCATGATGCCACAGGTAAAGTATATCTCCCTTTAAT

IRN7|AB863142.1| GACAAAAGAATTATCAGTGGACATTCATGATGCCACAGGTAAGGTATATCTCCCTTTAAT

IRN8|AB863143.1| GACAAAAGAATTATCAGTGGACATTCATGATGCCACAGGTAAGGTATATCTCCCTTTAAT

IRN9|AB863144.1| GACAAAGGAATTATCAGTAGACATTCATGATGCCACAGGTAAGGTATATCTCCCTTTAAT

IRN10|AB863145.1| GACAAAAGAATTATCAGTGGACATTCATGATGCCACAGGTAAGGTATATCTCCCTTTAAT

IRN11|AB863146.1| AACAAAAGAACTATCAGTGGACATTCATGATGCCACAGGTAAGGTATATCTCCCTTTAAT

IRN12|AB863147.1| GACAAAAGAATTATCAGTGGACATTCATGATGCCACAGGTAAAGTATATCTCCCTTTAAT

IRN13|AB863148.1| GACAAAAGAATTATCAGTGGACATTCATGATGCCACAGGTAAGGTATATCTCCCTTTAAT

IRN14|AB863149.1| GACAAAAGAATTATCAGTGGACATTCATGATGCCACAGGTAAAGTATATCTCCCTTTAAT

IRN15|AB863150.1| GACAAAGGAATTATCAGTGGACATTCATGATGCCACAGGTAAAGTATATCTCCCTTTAAT

IRN16|AB863151.1| GACAAAAGAATTATCAGTGGACATTCATGATGCCACAGGTAAGGTATATCTCCCTTTAAT

IRN17|AB863152.1| GACAAAGGAATTATCAGTGGACATTCATGATGCCACAGGTAAGGTATATCTCCCTTTAAT

IRN18|AB863153.1| GACAAAAGAATTATCAGTGGACATTCATGATGCCACAGGTAAGGTATATCTTCCCTTAAT

IRN19|AB863154.1| GACAAAGGAATTATCAGTGGACATTCATGATGCCACAGGTAAGGTATATCTCCCTTTAAT

IRN20|AB863155.1| GACAAAGGAATTATCAGTGGACATTCATGATGCCACAGGTAAAGTATATCTCCCTTTAAT

IRN21|AB863156.1| GACAAAAGAATTATCAGTGGACATTCATGATGCCACAGGTAAAGTATATCTCCCTTTAAT

JPNHGB340|AB863157.1| GACAAAAGAATTATCGGTGGACATTCACGATGCCACAGGTAAGGTATATCTTCCTTTAAT

JPNKWB778|AB863158.1| GACAAAAGAATTATCAGTGGACATTCACGATGCCACAGGTAAGGTATATCTTCCTTTAAT

JPNM|AB863159.1| GACAAAAGAATTATCAGTGGACATTCACGATGCCACAGGTAAGGTATATCTTCCTTTAAT

JPNN|AB863160.1| GACAAAAGAGTTATCAGTGGACATCCACGATGCCACAGGTAAGGTATATCTTCCTTTAAT

JPNS1|AB863161.1| GACAAAGGAATTATCAGTGGACATCCACGATGCCACAGGTAAGGTATATCTTCCTTTAAT

JPNS2|AB863162.1| GACAAAGGAATTATCAGTGGACATCCACGATGCCACAGGTAAGGTATATCTTCCTTTAAT

JPNTKD762|AB863163.1| GACAAAAGAATTATCAGTGGACATTCACGATGCCACAGGTAAGGTATATCTTCCTTTAAT

JPNUV1|AB863164.1| GACAAAAGAATTATCAGTGGACATTCACGATGCCACAGGTAAGGTATATCTTCCTTTAAT

JPNUV26|AB863165.1| GACAAAAGAATTATCAGTGGACATTCACGATGCCACAGGTAAGGTATATCTTCCTTTAAT

TUR1|AB863166.1| GACAAAAGAATTATCGGTGGACATTCACGATGCCACAGGTAAGGTATATCTCCCTTTAAT

TUR2|AB863167.1| GACAAAAGAATTATCGGTGGACATTCATGATGCCACAGGTAAGGTATATCTCCCTTTAAT

TUR4|AB863168.1| GACAAAAGAATTATCGGTGGACATTCATGATGCCACAGGTAAGGTATATCTCCCTTTAAT

TUR5|AB863169.1| GACAAAAGAATTATCGGTGGATATTCACGATGCCACAGGTAAAGTATATCTTCCCTTAAT

TUR12|AB863170.1| GACAAAAGAATTATCAGTGGACATTCATGATGCCACAGGTAAAGTATATCTTCCCTTAAT

TUR34|AB863171.1| GACAAAAGAATTATCGGTGGACATTCACGATGCCACAGGTAAAGTATATCTTCCCTTAAT

TUR50|AB863172.1| GACAAAAGAATTGTCGGTGGACATTCACGATGCCACAGGTAAGGTATATCTTCCCTTAAT

TUR59|AB863173.1| GACAAAGGAATTATCGGTGGACATTCACGATGCCACAGGTAAGGTATATCTCCCTTTAAT

TUR69|AB863174.1| GACAAAAGAACTATCGGTGGACATTCACGATGCCACAGGTAAGGTATATCTTCCCTTAAT

TUR81|AB863175.1| GACAAAAGAACTATCGGTGGACATTCACGATGCCACAGGTAAGGTATATCTTCCTTTAAT

TUR84|AB863176.1| GACAAAAGAACTATCGGTGGACATTCACGATGCCACAGGTAAGGTATATCTTCCTTTAAT

TUR94|AB863177.1| GACAAAAGAACTATCGGTGGACATTCACGATGCCACAGGTAAAGTATATCTCCCTTTAAT

TUR213|AB863178.1| GACAAAAGAATTATCAGTGGACATTCATGATGCCACAGGTAAAGTATATCTTCCCTTAAT

TUR214|AB863179.1| AACAAAAGAATTATCAGTGGACATTCACGATGCCACAGGTAAAGTATATCTTCCTTTAAT

TUR216|AB863180.1| GACAAAAGAATTATCGGTGGACATTCACGATGCCACAGGTAAAGTATATCTTCCTTTAAT

TUR220|AB863181.1| GACAAAAGAACTATCGGTGGACATTCACGATGCCACAGGTAAGGTATATCTTCCTTTAAT

TUR239|AB863182.1| GACAAAAGAATTATCGGTGGACATTCACGATGCCACAGGTAAAGTATATCTTCCTTTAAT

TUR244|AB863183.1| GACAAAAGAATTATCGGTGGACATTCACGATGCCACAGGTAAAGTATATCTTCCTTTAAT

TUR249|AB863184.1| GACAAAAGAATTATCGGTGGACATTCACGATGCCACAGGTAAGGTATATCTCCCTTTAAT

TUR263|AB863185.1| GACAAAGGAATTATCGGTGGACATTCACGATGCCACAGGTAAGGTATATCTTCCCTTAAT

TUR278|AB863186.1| GACAAAAGAATTATCAGTGGACATTCACGATGCCACAGGTAAAGTATATCTCCCTTTAAT

TUR279|AB863187.1| GACAAAAGAACTATCGGTGGACATTCACGATGCCACAGGTAAGGTATATCTTCCTTTAAT

TUR285|AB863188.1| AACAAAAGAATTATCGGTGGACATTCACGATGCCACAGGTAAGGTATATCTCCCTTTAAT

TUR289|AB863189.1| GACAAAAGAACTATCGGTGGACATTCACGATGCCACAGGTAAGGTATATCTTCCTTTAAT

TUR303|AB863190.1| GACAAAAGAATTATCAGTGGACATTCACGATGCCACAGGTAAGGTATATCTTCCCTTAAT

TUR306|AB863191.1| GACAAAAGAATTATCGGTGGACATTCACGATGCCACAGGTAAAGTATATCTCCCTTTAAT

Cabb-S|NC_001497.1| CACTAAGGAAGAGATAAATAAAAGACTTTCCAGCTTAAAACCTGAAGTCAGAAAGACCAT

CRO180A|AB863192.1| TACTAGAGAGGAGATAAACAAAAGACTTTCCAGCTTAAAACCTGAAGTCAGAAAGACCAT

GRC83|AB863193.1| CACTAGAGAGGAGATTAATAAAAGACTTTCCAGCTTAAAACCTGAAGTCAGAAAGACCAT

GRC84B|AB863194.1| CACTAGAGAGGAGATTAATAAAAGACTTTCCAGCTTAAAACCTGAAGTCAGAAAGACCAT

GRC86B|AB863195.1| CACTAGAGAGGAGATTAATAAAAGACTTTCCAGCTTAAAACCTGAAGTCAGAAAGACCAT

GRC86D|AB863196.1| CACTAGAGAGGAGATTAATAAAAGACTTTCCAGCTTAAAACCTGAAGTCAGAAAGACCAT

GRC87E|AB863197.1| CACTAGAGAGGAGATTAATAAAAGACTTTCCAGTTTAAAACCTGAAGTTAGAAAGACCAT

GRC87G|AB863198.1| CACTAGAGAGGAGATTAATAAAAGACTTTCCAGTTTAAAACCTGAAGTTAGAAAGACCAT

GRC91B|AB863199.1| CACTAAAGAAGAGATAAACAAAAGACTCTCCAGCTTGAAACCGGAAGTCAGAAAGACCAT

GRC92A|AB863200.1| CACTAGAGAGGAGATAAATAAAAGACTTTCCAGCTTAAAACCTGAAGTCAGAAAGACCAT

GRC92C|AB863201.1| CACTAGAGAGGAGATTAATAAAAGACTTTCCAGCTTAAAACCTGAAGTCAGAAAGACCAT

GRC92D|AB863202.1| CACTAGAGAGGAGATAAATAAAAGACTTTCCAGCTTAAAACCTGAAGTCAGAAAGACCAT

IRN1|AB863136.1| TACTAAAGAGGAGATAAACAAAAGACTTTCCAGCTTAAAACCTGAAGTCAGAAAGACCAT

IRN2|AB863137.1| CACTAAAGAAGAGATAAATAAAAGACTTTCCAGCTTAAAACCTGAAGTCAGAAAGACCAT

IRN3|AB863138.1| TACTAAAGAGGAGATAAACAAAAGACTTTCCAGCTTAAAACCTGAAGTCAGAAAGACCAT

IRN4|AB863139.1| CACTAAAGAAGAGATAAATAAAAGACTTTCCAGTTTAAAACCTGAAGTCAGAAAGACCAT

IRN5|AB863140.1| CACTAAAGAAGAGATAAATAAAAGACTTTCCAGTTTAAAACCTGAAGTCAGAAAGACCAT

IRN6|AB863141.1| CACTAAAGAAGAGATAAATAAAAGACTTTCCAGTTTAAAACCTGAAGTCAGAAAGACCAT

IRN7|AB863142.1| CACTAAAGAAGAGATAAATAAAAGACTTTCCAGTTTAAAACCTGAAGTCAGAAAGACCAT

IRN8|AB863143.1| CACTAAAGAAGAGATAAATAAAAGACTTTCCAGTTTAAAACCTGAAGTCAGAAAGACCAT

IRN9|AB863144.1| CACTAAAGAAGAGATAAATAAAAGACTTTCCAGTTTAAAACCTGAAGTCAGAAAGACCAT

IRN10|AB863145.1| CACTAAAGAAGAGATAAATAAAAGACTTTCCAGTTTAAAACCTGAAGTCAGAAAGACCAT

IRN11|AB863146.1| CACTAAAGAAGAGATAAATAAAAGACTTTCCAGCTTAAAACCTGAAGTCAGAAAGACCAT

IRN12|AB863147.1| CACTAAAGAAGAGGTAAATAAAAGACTTTCCAGTTTAAAACCTGAAGTCAGAAAGACCAT

IRN13|AB863148.1| CACTAAAGAAGAGATAAATAAAAGACTTTCCAGTTTAAAACCTGAAGTCAGAAAGACCAT

IRN14|AB863149.1| CACTAAAGAAGAGATAAATAAAAGACTTTCCAGTTTAAAACCTGAAGTCAGAAAGACCAT

IRN15|AB863150.1| TACTAGAGAGGAGATTAATAAAAGACTTTCCAGTTTAAAATCTGAAGTCAGAAAGACCAT

IRN16|AB863151.1| CACTAAAGAAGAGATAAATAAAAGACTTTCCAGCTTAAAACCTGAAGTCAGAAGAACCAT

IRN17|AB863152.1| CACTAAAGAAGAGATAAATAAAAGACTTTCCAGCTTAAAACCTGAAGTCAGAAGAACCAT

IRN18|AB863153.1| TACTAAGGAGGAGATAAACAAAAGACTTTCCAGCTTGAAACCGGAAGTCAGAAAGACCAT

IRN19|AB863154.1| CACTAAAGAAGAGATAAATAAAAGACTTTCCAGCTTAAAACCTGAAGTCAGAAAGACCAT

IRN20|AB863155.1| TACTAGAGAGGAGATTAATAAAAGACTTTCCAGTTTAAAATCTGAAGTCAGAAAGACCAT

IRN21|AB863156.1| CACTAAAGAAGAGATAAATAAAAGACTTTCCAGTTTAAAATCTGAAGTCAGAAAGACCAT

JPNHGB340|AB863157.1| CACTAAAGAGGAGATAAATAAAAGACTTTCTAGTTTAAAACCTGAAGTCAGAAAGACCAT

JPNKWB778|AB863158.1| CACTAAAGAGGAGATAAATAAAAGACTTTCTAGCTTAAAACCTGAAGTCAGAAAGACCAT

JPNM|AB863159.1| CACTAAAGAGGAGATAAATAAAAGACTTTCTAGCTTAAAACCTGAAGTCAGAAAGACCAT

JPNN|AB863160.1| TACTAAAGAGGAGATAAATAAAAGACTTTCTAGTTTAAAACCTGAAGTCAGAAAGACCAT

JPNS1|AB863161.1| TACTAAAGAGGAGATAAATAAAAGACTTTCTAGTTTAAAACCTGAAGTCAGAAAGACCAT

JPNS2|AB863162.1| TACTAAAGAGGAGATAAATAAAAGACTTTCTAGTTTAAAACCTGAAGTCAGAAAGACCAT

JPNTKD762|AB863163.1| CACTAAAGAGGAGATAAATAAAAGACTTTCTAGCTTAAAACCTGAAGTCAGAAAGACCAT

JPNUV1|AB863164.1| CACTAAAGAGGAGATAAATAAAAGACTTTCTAGCTTAAAACCTGAAGTCAGAAAGACCAT

JPNUV26|AB863165.1| CACTAAAGAGGAGATAAATAAAAGACTTTCTAGCTTAAAACCTGAAGTCAGAAAGACCAT

TUR1|AB863166.1| CACTAAGGAAGAGATAAATAAAAGACTCTCCAGCTTAAAACCTGAAGTCAGAAGAACCAT

TUR2|AB863167.1| CACTAGAGAGGAGATTAATAAAAGACTTTCCAGCTTAAAACCTGAAGTCAGAAGAACCAT

TUR4|AB863168.1| CACTAGAGAGGAGATTAATAAAAGACTTTCCAGCTTAAAACCTGAAGTCAGAAGAACCAT

TUR5|AB863169.1| CACTAAGGAAGAGATAAATAAAAGACTTTCCAGCTTAAAACCTGAAGTCAGAAAGACCAT

TUR12|AB863170.1| CACTAAAGAGGAGATTAACAAAAGACTTTCCAGCTTAAAACCTGAAGTCAGAAAGACCAT

TUR34|AB863171.1| CACTAAGGAGGAGATAAACAAAAGACTTTCCAGCTTAAAACCTGAAGTCAGAAAGACCAT

TUR50|AB863172.1| CACTAAGGAGGAGATAAACAAAAGACTTTCCAGCTTAAAACCTGAAGTCAAAAAGACCAT

TUR59|AB863173.1| CACTAAGGAAGAGATAAATAAAAGACTTTCCAGCTTAAAACCTGAAGTCAGAAGAACCAT

TUR69|AB863174.1| CACTAAAGAGGAGATAAATAAAAGACTTTCCAGTTTAAAACCTGAAGTCAGAAAGACCAT

TUR81|AB863175.1| CACTAAAGAGGAGATAAATAAAAGACTTTCCAGCTTAAAACCTGAAGTCAGAAAGACCAT

TUR84|AB863176.1| TACTAAAGAGGAGATAAATAAAAGACTTTCCAGCTTAAAACCTGAAGTCAGAAAGACCAT

TUR94|AB863177.1| CACTAAGGAAGAGATAAACAAAAGACTTTCCAGCTTAAAACCTGAAGTCAGAAAGACCAT

TUR213|AB863178.1| CACTAAGGAAGAGATAAATAAAAGACTTTCCAGCTTAAAACCTGAAGTCAGAAGAACCAT

TUR214|AB863179.1| CACTAAAGAGGAGATAAATAAAAGACTTTCCAGCTTAAAACCTGAAGTCAGAAAGACCAT

TUR216|AB863180.1| CACTAAAGAGGAGATAAATAAAAGACTTTCCAGCTTAAAACCGGAAGTCAGAAAGACCAT

TUR220|AB863181.1| TACTAAAGAGGAGATAAATAAAAGACTTTCCAGCTTAAAACCTGAAGTCAGAAAGACCAT

TUR239|AB863182.1| CACTAAAGAGGAGATAAATAAAAGACTCTCCAGCTTAAAACCTGAAGTCAGAAAGATCAT

TUR244|AB863183.1| CACTAAAGAGGAGATAAATAAAAGACTCTCCAGCTTAAAACCTGAAGTCAGAAAGACCAT

TUR249|AB863184.1| CACTAAGGAAGAGATAAACAAAAGACTCTCCAGCTTAAAACCTGAAGTCAGAAAGACCAT

TUR263|AB863185.1| CACTAAGGAAGAGATAAACAAAAGACTTTCCAGCTTAAAACCTGAAGTCAGAAGAACCAT

TUR278|AB863186.1| CACTAGAGAGGAGATTAATAAAAGACTTTCCAGCTTAAAACCTGAAGTCAGAAAGACCAT

TUR279|AB863187.1| CACTAAGGAAGAAATAAACAAAAGACTTTCCAGCTTAAAACCTGAAGTCAGAAAGACCAT

TUR285|AB863188.1| CACTAAGGAAGAGATAAACAAAAGACTTTCCAGCTTAAAACCTGAAGTCAGAAAGACCAT

TUR289|AB863189.1| CACTAAAGAGGAGATAAATAAAAGACTTTCCAGCTTAAAACCTGAAGTCAGAAAGACCAT

TUR303|AB863190.1| CACTAAGGAGGAGATAAATAAAAGACTTTCCAGCTTAAAACCTGAAGTCAGAAAGACCAT

TUR306|AB863191.1| CACTAAGGAAGAGATAAATAAAAGACTTTCCAGTTTAAAACCTGAAGTCAGAAAGACCAT

Cabb-S|NC_001497.1| GTCCATGGTTCATCTTGGAGCGGTCAAAATATTGCTTAAAGCTCAATTTCGAAATGGGAT

CRO180A|AB863192.1| GTCCATGGTTCATCTTGGAGCAGTCAAAATATTGCTTAAAGCTCAATTTAGAAATGGGAT

GRC83|AB863193.1| GTCCATGGTTCATCTTGGAGCGGTCAAAATATTGCTTAAAGCTCAATTTAGAAATGGGAT

GRC84B|AB863194.1| GTCCATGGTTCATCTTGGAGCGGTCAAAATATTGCTTAAAGCTCAATTTAGAAATGGGAT

GRC86B|AB863195.1| GTCCATGGTTCATCTTGGAGCGGTCAAAATATTGCTTAAAGCTCAATTTAGAAATGGGAT

GRC86D|AB863196.1| GTCCATGGTTCATCTTGGAGCGGTCAAAATATTGCTTAAAGCTCAATTTAGAAATGGGAT

GRC87E|AB863197.1| GTCCATGGTTCATCTTGGAGCGGTCAAAATATTGCTTAAAGCTCAATTTAGAAATGGGAT

GRC87G|AB863198.1| GTCCATGGTTCATCTTGGAGCGGTCAAAATATTGCTTAAAGCTCAATTTAGAAATGGGAT

GRC91B|AB863199.1| GTCCATAGTTCATCTTGGAGCGGTCAAAATATTGCTTAAAGCTCAATTTAGAAATGGGAT

GRC92A|AB863200.1| GTCCATGGTTCATCTTGGAGCGGTCAAAATATTGCTTAAAGCTCAATTTAGAAATGGGAT

GRC92C|AB863201.1| GTCCATGGTTCATCTTGGAGCGGTCAAAATATTGCTTAAAGCTCAATTTAGAAATGGGAT

GRC92D|AB863202.1| GTCCATGGTTCATCTTGGAGCGGTCAAAATATTGCTTAAAGCTCAATTTAGAAATGGGAT

IRN1|AB863136.1| GTCCATAGTTCATCTTGGAGCGGTCAAAATATTGCTTAAAGCTCAATTTAGAAATGGGAT

IRN2|AB863137.1| GTCCATAGTACATCTTGGAGCGGTCAAAATATTGCTTAAAGCTCAATTTCGAAATGGGAT

IRN3|AB863138.1| GTCCATAGTTCATCTTGGAGCGGTCAAAATATTGCTTAAAGCTCAATTTAGAAATGGGAT

IRN4|AB863139.1| GTCCATGGTTCATCTTGGAGCGGTCAAAATATTGCTTAAAGCTCAATTTAGAAATGGGAT

IRN5|AB863140.1| GTCCATGGTTCATCTTGGAGCGGTCAAAATATTGCTTAAAGCTCAATTTAGAAATGGGAT

IRN6|AB863141.1| GTCCATGATTCATCTTGGAGCGGTCAAAATATTGCTTAAAGCTCAATTTAGAAATGGGAT

IRN7|AB863142.1| GTCCATGGTTCATCTTGGAGCGGTCAAAATATTGCTTAAAGCTCAATTTAGAAATGGGAT

IRN8|AB863143.1| GTCCATGGTTCATCTTGGAGCGGTCAAAATATTGCTTAAAGCTCAATTTAGAAATGGGAT

IRN9|AB863144.1| GTCCATGGTTCATCTTGGAGCGGTCAAAATATTGCTTAAAGCTCAATTTAGAAATGGGAT

IRN10|AB863145.1| GTCCATAGTTCATCTTGGAGCGGTCAAAATATTGCTTAAAGCTCAATTTAGAAATGGGAT

IRN11|AB863146.1| GTCCATAGTACATCTTGGAGCGGTCAAAATATTGCTTAAAGCTCAATTTCGAAATGGGAT

IRN12|AB863147.1| GTCCATGATTCATCTTGGAGCGGTCAAAATATTGCTTAAAGCTCAATTTAGAAATGGGAT

IRN13|AB863148.1| GTCCATGGTTCATCTTGGAGCGGTCAAAATATTGCTTAAAGCTCAATTTAGAAATGGGAT

IRN14|AB863149.1| GTCCATGGTTCATCTTGGAGCGGTCAAAATATTGCTTAAAGCTCAATTTAGAAATGGGAT

IRN15|AB863150.1| GTCCATGGTTCATCTTGGAGCGGTCAAAATATTGCTTAAAGCTCAATTTAGAAATGGGAT

IRN16|AB863151.1| GTCCATGGTCCACTTGGGCGCGGTTAAAATATTGCTTAAAGCTCAATTTAGAAATGGGAT

IRN17|AB863152.1| GTCCATGGTCCACTTGGGCGCGGTCAAAATATTGCTTAAAGCTCAATTTAGAAATGGGAT

IRN18|AB863153.1| GTCCATAGTACATCTTGGAGCGGTCAAAATATTGCTTAAAGCTCAATTTCGAAATGGGAT

IRN19|AB863154.1| GTCCATAGTACATCTTGGAGCGGTCAAAATATTGCTTAAAGCTCAATTTCGAAATGGGAT

IRN20|AB863155.1| GTCCATGGTTCATCTTGGAGCGGTCAAAATATTGCTTAAAGCTCAATTTAGAAATGGGAT

IRN21|AB863156.1| GTCCATGGTTCATCTTGGAGCGGTCAAAATATTGCTTAAAGCTCAATTTAGAAATGGGAT

JPNHGB340|AB863157.1| GTCCATGGTTCATCTTGGAGCGGTCAAAATATTGCTTAAAGCTCAATTTCGAAATGGGAT

JPNKWB778|AB863158.1| GTCCATGGTTCATCTTGGAGCGGTCAAAATATTGCTTAAAGCTCAATTTCGAAATGGGAT

JPNM|AB863159.1| GTCCATGGTTCATCTTGGAGCGGTCAAAATATTGCTTAAAGCTCAATTTCGAAATGGGAT

JPNN|AB863160.1| GTCCATGGTTCACCTTGGAGCGGTCAAAATATTGCTTAAAGCTCAATTTCGAAACGGGAT

JPNS1|AB863161.1| GTCCATGGTTCACCTTGGAGCGGTCAAAATATTGCTTAAAGCTCAATTTCGAAACGGGAT

JPNS2|AB863162.1| GTCCATGGTTCACCTTGGAGCGGTCAAAATATTGCTTAAAGCTCAATTTCGAAACGGGAT

JPNTKD762|AB863163.1| GTCCATGGTTCATCTTGGAGCGGTCAAAATATTGCTTAAAGCTCAATTTCGAAATGGGAT

JPNUV1|AB863164.1| GTCCATAGTTCATCTTGGAGCGGTCAAAATATTGCTTAAAGCTCAATTTCGAAATGGGAT

JPNUV26|AB863165.1| GTCCATGGTTCATCTTGGAGCGGTCAAAATATCGCTTAAAGCTCAATTTCGAAATGGGAT

TUR1|AB863166.1| GTCCATAGTCCACTTGGGCGCGGTAAAAATATTGCTTAAAGCTCAATTTAGAAATGGGAT

TUR2|AB863167.1| GTCCATGGTCCACTTGGGCGCGGTTAAAATATTGCTTAAAGCTCAATTTCGAAATGGGAT

TUR4|AB863168.1| GTCCATGGTCCACTTGGGCGCGGTTAAAATATTGCTTAAAGCTCAATTTCGAAATGGGAT

TUR5|AB863169.1| GTCCATAGTCCATCTTGGAGCGGTCAAAATATTGCTTAAAGCTCAATTTAGAAATGGGAT

TUR12|AB863170.1| GTCCATAGTCCATCTTGGAGCGGTCAAAATATTGCTTAAAGCTCAATTTCGAAATGGGAT

TUR34|AB863171.1| GTCCATAGTTCATCTTGGAGCGGTCAAAATATTGCTTAAAGCTCAATTTCGAAATGGGAT

TUR50|AB863172.1| GTCCATAGTTCATCTTGGAGCGGTCAAAATATTGCTTAAAGCTCAATTTCGAAATGGGAT

TUR59|AB863173.1| GTCCATGGTCCACTTGGGCGCGGTTAAAATATTGCTTAAAGCTCAATTTCGAAATGGGAT

TUR69|AB863174.1| GTCCATAGTCCATCTTGGAGCGGTCAAAATATTGCTTAAAGCTCAATTTCGAAATGGGAT

TUR81|AB863175.1| GTCCATAGTCCATCTGGGCGCGGTTAAAATATTGCTTAAAGCTCAATTTAGAAATGGGAT

TUR84|AB863176.1| GTCCATAGTCCATCTGGGCGCGGTTAAAATATTGCTTAAAGCTCAATTTAGAAATGGGAT

TUR94|AB863177.1| GTCCATTGTTCATCTTGGAGCGGTCAAAATATTGCTTAAAGCTCAATTTAGAAATGGGAT

TUR213|AB863178.1| GTCCATGGTCCACTTGGGCGCGGTTAAAATATTGCTTAAAGCTCAATTTAGAAATGGGAT

TUR214|AB863179.1| GTCCATAGTCCATCTTGGAGCGGTCAAAATATTGCTTAAAGCTCAATTTCGAAACGGGAT

TUR216|AB863180.1| GTCCATTGTTCATCTTGGAGCGGTCAAAATATTGCTTAAAGCTCAATTTCGAAATGGGAT

TUR220|AB863181.1| GTCCATAGTCCATCTGGGCGCGGTTAAAATATTGCTTAAAGCTCAATTTAGAAATGGGAT

TUR239|AB863182.1| GTCCATTGTTCATCTTGGAGCGGTCAAAATATTGCTTAAAGCTCAATTTCGAAATGGGAT

TUR244|AB863183.1| GTCCATTGTTCATCTTGGAGCGGTCAAAATATTGCTTAAAGCTCAATTTCGAAATGGGAT

TUR249|AB863184.1| GTCCATTGTTCATCTTGGAGCGGTCAAAATATTGCTTAAAGCTCAATTTCGAAATGGAAT

TUR263|AB863185.1| GTCCATGGTCCACTTGGGCGCGGTTAAAATATTGCTTAAAGCTCAATTTCGAAATGGGAT

TUR278|AB863186.1| GTCCATAGTCCATCTTGGAGCGGTCAAAATATTGCTTAAAGCTCAATTTAGAAATGGGAT

TUR279|AB863187.1| GTCCATAGTCCATCTTGGAGCGGTCAAAATATTGCTTAAAGCTCAATTTCGAAATGGGAT

TUR285|AB863188.1| GTCCATAGTCCATCTTGGAGCGGTCAAAATATTGCTTAAAGCTCAATTTCGAAATGGGAT

TUR289|AB863189.1| GTCCATAGTCCATCTTGGAGCGGTTAAGATATTGCTTAAAGCTCAATTTAGAAATGGGAT

TUR303|AB863190.1| GTCCATTGTTCATCTTGGAGCGGTCAAAATATTGCTTAAAGCTCAATTTCGAAATGGGAT

TUR306|AB863191.1| GTCCATAGTCCATCTTGGAGCGGTCAAAATATTGCTTAAAGCTCAATTTCGAAATGGGAT

Cabb-S|NC_001497.1| TGATACCCCAATCAAAATTGCTTTAATCGATGATAGAATCAATTCTAGAAGAGATTGTCT

CRO180A|AB863192.1| TGATACCCCAATCAAAATTGCTTTAATCGATGATAGAATCAATTCTAGAAGAGATTGTCT

GRC83|AB863193.1| TGATACCCCAATCAAAATTGCTTTAATCGATGATAGAATCAATTCTAGAAGAGATTGTCT

GRC84B|AB863194.1| TGATACCCCAATCAAAATTGCTTTAATCGATGATAGAATCAATTCTAGAAGAGATTGTCT

GRC86B|AB863195.1| TGATACCCCAATCAAAATTGCTTTAATCGATGATAGAATCAATTCTAGAAGAGATTGTCT

GRC86D|AB863196.1| TGATACCCCAATCAAAATTGCTTTAATCGATAATAGAATCAATTCTAGAAGAGATTGTCT

GRC87E|AB863197.1| TGATACCCCAATCAAAATTGCTTTAATCGATGATAGAATCAATTCTAGAAGAGATTGTCT

GRC87G|AB863198.1| TGATACCCCAATCAAAATTGCTTTAATCGATGATAGAATCAATTCTAGAAGAGATTGTCT

GRC91B|AB863199.1| TGATACCCCAATCAAGATTGCTTTAATCGATGATAGAATCAATTCTAGAAGAGATTGTCT

GRC92A|AB863200.1| TGATACCCCAATCAAAATTGCTTTAATCGATGATAGAATCAATTCTAGAAGAGATTGTCT

GRC92C|AB863201.1| TGATACCCCAATCAAAATTGCTTTAATCGATGATAGAATCAATTCTAGAAGAGATTGTCT

GRC92D|AB863202.1| TGATACCCCAATCAAAATTGCTTTAATCGATGATAGAATCAATTCTAGAAGAGATTGTCT

IRN1|AB863136.1| TGATACCCCAATCAAAATTGCTTTAATCGATGATAGAATCAATTCTAGAAAAGATTGTCT

IRN2|AB863137.1| TGATACCCCAATCAAAATTGCTTTAATCGATGATAGAATTAATTCTAGAAGAGATTGTCT

IRN3|AB863138.1| TGATACCCCAATCAAAATTGCTTTAATCGATGATAGAATCAATTCTAGAAAAGATTGTCT

IRN4|AB863139.1| TGACACCCCAATCAAAATTGCTTTAATCGATGATAGAATCAATTCTAGAAGAGATTGTCT

IRN5|AB863140.1| TGATACCCCAATCAAAATTGCTTTAATCGATGATAGAATCAATTCTAGAAGAGATTGTCT

IRN6|AB863141.1| TGATACCCCAATCAAAATTGCTTTAATCGATGATAGAATCAATTCTAGAAGAGATTGTCT

IRN7|AB863142.1| TGATACCCCAATCAAAATTGCTTTAATCGATGATAGAATCAATTCTAGAAGAGATTGTCT

IRN8|AB863143.1| TGATACCCCAATCAAAATTGCTTTAATCGATGATAGAATCAATTCTAGAAGAGATTGTCT

IRN9|AB863144.1| TGATACCCCAATCAAAATTGCTTTAATCGATGATAGAATCAATTCTAGAAGAGATTGTCT

IRN10|AB863145.1| TGATACCCCAATCAAAATTGCTTTAATCGATGATAGAATCAATTCTAGAAAAGATTGTCT

IRN11|AB863146.1| TGATACCCCAATCAAAATTGCTTTAATCGATGATAGAATTAATTCTAGAAGAGATTGTCT

IRN12|AB863147.1| TGACACCCCAATCAAAATTGCTTTAATCGATGATAGAATCAATTCTAGAAGAGATTGTCT

IRN13|AB863148.1| TGATACCCCAATCAAAATTGCTTTAATCGATGATAGAATCAATTCTAGAAGAGATTGTCT

IRN14|AB863149.1| TGATACCCCAATCAAAATTGCTTTAATCGATGATAGAATCAATTCTAGAAGAGATTGTCT

IRN15|AB863150.1| TGATACCCCAATCAAAATTGCTTTAATCGATGATAGAATCAATTCTAGAAGAGATTGTCT

IRN16|AB863151.1| TGATACCCCAATCAAAATTGCTTTAATCGATGATAGAATCAATTCTAGAAGAGATTGTCT

IRN17|AB863152.1| TGATACCCCAATCAAAATTGCTTTAATCGATGATAGAATCAATTCTAGAAGAGATTGTCT

IRN18|AB863153.1| TGATACCCCAATCAAAATTGCTTTAATCGATGATAGAATTAATTCTAGAAGAGATTGTCT

IRN19|AB863154.1| TGATACCCCAATCAAAATTGCTTTAATCGATGATAGAATTAATTCTAGAAGAGATTGTCT

IRN20|AB863155.1| TGATACCCCAATCAAAATTGCTTTAATCGATGATAGAATCAATTCTAGAAGAGATTGTCT

IRN21|AB863156.1| TGATACCCCAATCAAAATTGCTTTAATCGATGATAGAATCAATTCTAGAAGAGATTGTCT

JPNHGB340|AB863157.1| TGATACCCCAATCAAAATTGCTTTAATCGATGATAGAATTAATTCTAGAAGAGATTGCCT

JPNKWB778|AB863158.1| TGATACCCCAATCAAAATTGCTTTAATCGATGATAGAATTAATTCTAGAAGAGATTGCCT

JPNM|AB863159.1| TGATACCCCAATCAAAATTGCTTTAATCGATGATAGAATTAATTCTAGAAGAGATTGCCT

JPNN|AB863160.1| AGATACCCCGATCAAAATTGCTTTAATCGATGATAGAATTAATTCTAGAAGAGATTGCCT

JPNS1|AB863161.1| AGATACCCCGATCAAAATTGCTTTAATCGATGATAGAATTAATTCTAGAAGAGATTGCCT

JPNS2|AB863162.1| AGATACCCCGATCAAAATTGCTTTAATCGATGATAGAATTAATTCTAGAAGAGATTGCCT

JPNTKD762|AB863163.1| TGATACCCCAATCAAAATTGCTTTAATCGATGATAGAATTAATTCTAGAAGAGATTGCCT

JPNUV1|AB863164.1| TGATACCCCAATCAAAATTGCTTTAATCGATGATAGAATTAATTCTAGAAGAGATTGCCT

JPNUV26|AB863165.1| TGATACCCCAATCAAAATTGCTTTAATCGATGATAGAATTAATTCTAGAAGAGATTGCCT

TUR1|AB863166.1| TGATACCCCAATCAAAATTGCTTTAATCGATGATAGAATCAATTCTAGAAGAGATTGTCT

TUR2|AB863167.1| TGATACCCCAATCAAAATAGCTTTAATCGATGATAGAATCAATTCTAGAAGAGATTGTCT

TUR4|AB863168.1| TGATACCCCAATCAAAATAGCTTTAATCGATGATAGAATCAATTCTAGAAGAGATTGTCT

TUR5|AB863169.1| TGATACCCCAATCAAAATTGCTTTAATCGATGATAGAATCAATTCTAGAAGAGATTGTCT

TUR12|AB863170.1| TGATACCCCAATCAAAATTGCTTTAATCGATGATAGAATTAATTCTAGAAGAGATTGTCT

TUR34|AB863171.1| TGATACCCCAATCAAAATTGCTTTAATCGATGATAGAATCAATTCTAGAAAAGATTGTCT

TUR50|AB863172.1| TGATACCCCAATCAAAATTGCTTTAATCGATGATAGAATCAATTCTAGAAAAGATTGTCT

TUR59|AB863173.1| TGATACCCCAATCAAAATTGCTTTAATCGATGATAGAATCAATTCTAGAAGAGATTGTCT

TUR69|AB863174.1| TGATACCCCAATCAAAATTGCTTTAATCGATGATAGAATCAATTCTAGAAGAGATTGTCT

TUR81|AB863175.1| TGATACCCCAATCAAAATTGCTTTAATCGATGATAGAATCAATTCTAGAAGAGATTGTCT

TUR84|AB863176.1| TGATACCCCGATCAAAATTGCTTTAATCGATGATAGAATCAATTCTAGAAGAGATTGTCT

TUR94|AB863177.1| TGATACCCCAATCAAAATTGCTTTAATCGATGATAGAATCAATTCTAGAAAAGATTGTCT

TUR213|AB863178.1| TGATACCCCAATCAAAATTGCTTTAATCGATGATAGAATCAATTCTAGAAGAGATTGTCT

TUR214|AB863179.1| TGATACCCCAATCAAAATTGCTTTAATCGATGATAGAATCAATTCTAGAAGAGATTGTCT

TUR216|AB863180.1| TGATACCCCAATCAAAATTGCTTTAATCGATGATAGAATCAATTCTAGAAGAGATTGTCT

TUR220|AB863181.1| TGATACCCCGATCAAAATTGCTTTAATCGATGATAGAATCAATTCTAGAAGAGATTGTCT

TUR239|AB863182.1| TGATACCCCAATCAAAATTGCTTTAATCGATGATAGAATCAATTCTAGAAGAGATTGTCT

TUR244|AB863183.1| TGATACCCCAATCAAAATTGCTTTAATCGATGATAGAATCAATTCTAGAAGAGATTGTCT

TUR249|AB863184.1| TGATACTCCAATCAAAATTGCTTTAATCGATGATAGAATCAATTCTAGAAGAGATTGCCT

TUR263|AB863185.1| TGATACCCCAATCAAAATTGCTTTAATCGATGATAGAATCAATTCTAGAAAAGATTGCCT

TUR278|AB863186.1| TGATACCCCAATCAAAATTGCTTTAATCGATGATAGAATCAATTCTAGAAGAGATTGTCT

TUR279|AB863187.1| TGATACCCCAATCAAAATTGCTTTAATCGATGATAGAATCAATTCTAGAAAAGATTGTCT

TUR285|AB863188.1| TGATACCCCAATCAAAATTGCTTTAATCGATGATAGAATCAATTCTAGAAGAGATTGTCT

TUR289|AB863189.1| TGATACCCCAATCAAAATTGCTTTAATCGATGATAGAATCAATTCTAGAAGAGATTGCCT

TUR303|AB863190.1| TGATACCCCAATCAAAATTGCTTTAATCGATGATAGAATCAATTCTAGAAGAGATTGTCT

TUR306|AB863191.1| TGATACCCCAATCAAAATTGCTTTAATCGATGATAGAATCAATTCTAGAAGAGATTGTCT

Cabb-S|NC_001497.1| TCTTGGTGCAGCCAAAG**GT**AATCTAGCATACG**GT**AAGTTTATGTTTACTGTATACCCTAA

**D2**

**D1**

CRO180A|AB863192.1| TCTTGGTGCAGCCAAAGGTAATCTCGCATACGGTAAGTTTATGTTTACTGTATACCCTAA

GRC83|AB863193.1| TCTTGGTGCTGCCAAAGGTAATCTCGCATACGGTAAGTTTATGTTTACTGTATACCCTAA

GRC84B|AB863194.1| TCTTGGTGCTGCCAAAGGTAATCTCGCATACGGTAAGTTTATGTTTACTGTATACCCTAA

GRC86B|AB863195.1| TCTTGGTGCAGCCAAAGGTAATCTCGCATACGGTAAATTTATGTTTACTGTATACCCTAA

GRC86D|AB863196.1| TCTTGGTGCAGCCAAAGGTAATCTCGCATACGGTAAGTTTATGTTTACTGTATACCCTAA

GRC87E|AB863197.1| TCTTGGTGCAGCCAAAGGTAATCTCGCATACGGTAAGTTTATGTTTACTGTATACCCTAA

GRC87G|AB863198.1| TCTTGGTGCAGCCAAAGGTAATCTCGCATACGGTAAGTTTATGTTTACTGTATACCCTAA

GRC91B|AB863199.1| TCTTGGTGCAGCCAAAGGTAATCTCGCATACGGTAAGTTTATGGTTACTGTATACCCTAA

GRC92A|AB863200.1| TCTTGGTGCAGCCAAAGGTAATCTCGCATACGGTAAGTTTATGTTTACTGTATACCCTAA

GRC92C|AB863201.1| TCTTGGTGCAGCCAAAGGTAATCTCGCATACGGTAAGTTTATGTTTACTGTATACCCTAA

GRC92D|AB863202.1| TCTTGGTGCAGCCAAAGGTAATCTCGCATACGGTAAGTTTATGTTTACTGTATACCCTAA

IRN1|AB863136.1| ACTTGGTGCAGCCAAAGGTAATCTAGCATACGGTAAGTTTATGTTTACTGTATACCCTAA

IRN2|AB863137.1| ACTTGGTGCAGCCAAAGGTAATCTAGCATACGGTAAGTTTATGTTTACTGTATACCCTAA

IRN3|AB863138.1| ACTTGGTGCAGCCAAAGGTAATCTAGCATACGGTAAGTTTATGTTTACTGTATACCCTAA

IRN4|AB863139.1| TCTTGGTGCAGCCAAAGGTAATCTAGCATACGGTAAGTTCATGTTTACTGTATACCCTAA

IRN5|AB863140.1| TCTTGGTGCAGCCAAAGGTAATCTAGCATACGGTAAGTTTATGTTTACTGTATACCCTAA

IRN6|AB863141.1| ACTTGGTGCAGCCAAAGGTAATCTAGCATACGGTAAGTTTATGTTTACTGTATACCCTAA

IRN7|AB863142.1| TCTTGGTGCAGCCAAAGGTAATCTAGCATACGGTAAGTTTATGTTTACTGTATACCCTAA

IRN8|AB863143.1| TCTTGGTGCAGCCAAAGGTAATCTAGCATACGGTAAGTTTATGTTTACTGTATACCCTAA

IRN9|AB863144.1| TCTTGGTGCAGCCAAAGGTAATCTAGCATACGGTAAGTTTATGTTTACTGTATACCCTAA

IRN10|AB863145.1| ACTTGGTGCAGCCAAAGGTAATCTAGCATACGGTAAGTTTATGTTTACTGTATACCCTAA

IRN11|AB863146.1| ACTTGGTGCAGCCAAAGGTAATCTAGCATACGGTAAGTTTATGTTTACTGTATACCCTAA

IRN12|AB863147.1| TCTTGGTGCAGCCAAAGGTAATCTAGCATACGGTAAGTTTATGTTTACTGTATACCCTAA

IRN13|AB863148.1| TCTTGGTGCAGCCAAAGGTAATCTAGCATACGGTAAATTTATGTTTACTGTATACCCTAA

IRN14|AB863149.1| TCTTGGTGCAGCCAAAGGTAATCTAGCATACGGTAAGTTTATGTTTACTGTATACCCTAA

IRN15|AB863150.1| TCTTGGTGCAGCCAAAGGTAATCTAGCATACGGTAAGTTTATGTTTACTGTATACCCTAA

IRN16|AB863151.1| TCTTGGTGCAGCCAAAGGTAATCTAGCATACGGTAAGTTTATGTTTACTGTATACCCTAA

IRN17|AB863152.1| TCTTGGTGCAGCCAAAGGTAATCTAGCATACGGTAAGTTTATGTTTACTGTATACCCTAA

IRN18|AB863153.1| TCTTGGTGCAGCCAAAGGTAATCTAGCATACGGTAAGTTTATGTTTACTGTATACCCCAA

IRN19|AB863154.1| ACTTGGTGCAGCCAAAGGTAATCTAGCATACGGTAAGTTTATGTTTACTGTATACCCTAA

IRN20|AB863155.1| TCTTGGTGCAGCCAAAGGTAATCTAGCATACGGTAAGTTTATGTTTACTGTATACCCTAA

IRN21|AB863156.1| ACTTGGTGCAGCCAAAGGTAATCTAGCATACGGTAAGTTTATGTTTACTGTATACCCTAA

JPNHGB340|AB863157.1| TCTCGGTGCAGCCAAAGGTAATCTAGCATACGGTAAGTTTATGTTTACTGTATACCCCAA

JPNKWB778|AB863158.1| TCTCGGTGCAGCCAAAGGTAATCTAGCATACGGTAAGTTTATGTTTACTGTATACCCCAA

JPNM|AB863159.1| TCTCGGTGCAGCCAAAGGTAATCTAGCATACGGTAAGTTTATGTTTACTGTATACCCCAA

JPNN|AB863160.1| TCTCGGTGCAGCCAAAGGTAATCTAGCATACGGTAAGTTTATGTTTACTGTATACCCTAA

JPNS1|AB863161.1| TCTCGGTGCAGCCAAAGGTAATCTAGCATACGGTAAGTTTATGTTTACTGTATACCCCAA

JPNS2|AB863162.1| TCTCGGTGCAGCCAAAGGTAATCTAGCATACGGTAAGTTTATGTTTACTGTATACCCCAA

JPNTKD762|AB863163.1| TCTCGGTGCAGCCAAAGGTAATCTAGCATACGGTAAGTTTATGTTTACTGTATACCCCAA

JPNUV1|AB863164.1| TCTCGGTGCAGCCAAAGGTAATCTAGCATACGGTAAGTTTATGTTTACTGTATACCCCAA

JPNUV26|AB863165.1| TCTCGGTGCAGCCAAAGGTAATCTAGCATACGGTAAGTTTATGTTTACTGTATACCCCAA

TUR1|AB863166.1| TCTTGGTGCAGCCAAAGGTAATCTAGCATACGGTAAGTTTATGTTTACTGTATACCCTAA

TUR2|AB863167.1| TCTTGGTGCAGCCAAAGGTAATCTCGCATACGGTAAGTTTATGTTTACTGTATACCCTAA

TUR4|AB863168.1| TCTTGGTGCAGCCAAAGGTAATCTCGCATACGGTAAGTTTATGTTTACTGTATACCCTAA

TUR5|AB863169.1| TCTTGGTGCAGCCAAAGGTAATCTTGCATACGGTAAGTTCATGTTTACTGTATACCCTAA

TUR12|AB863170.1| ACTTGGTGCTGCCAAAGGTAATCTAGCATACGGTAAGTTTATGTTTACTGTATACCCTAA

TUR34|AB863171.1| TCTTGGTGCAGCCAAAGGTAATCTAGCATACGGTAAGTTTATGTTTACTGTATACCCTAA

TUR50|AB863172.1| TCTTGGTGCAGCCAAAGGTAATCTAGCATACGGTAAGTTTATGTTTACTGTATACCCTAA

TUR59|AB863173.1| TCTTGGTGCAGCCAAAGGTAATCTAGCATACGGTAAG------TTTACTGTATACCCTAA

TUR69|AB863174.1| TCTTGGTGCAGCCAAAGGTAATCTAGCATACGGTAAGTTTATGTTTACTGTATACCCTAA

TUR81|AB863175.1| TCTTGGTGCAGCCAAAGGTAATCTAGCATACGGTAAGTTTATGTTTACTGTATACCCTAA

TUR84|AB863176.1| TCTTGGTGCAGCCAAAGGTAATCTAGCATACGGTAAGTTTATGTTTACTGTATACCCTAA

TUR94|AB863177.1| TCTTGGTGCTGCCAAAGGTAATCTAGCATACGGTAAGTTTATGTTTACTGTATACCCTAA

TUR213|AB863178.1| TCTTGGTGCAGCCAAAGGTAATCTAGCATACGGTAAGTTTATGTTTACTGTATACCCTAA

TUR214|AB863179.1| TCTTGGTGCAGCCAAAGGTAATCTAGCATACGGTAAGTTTATGTTTACTGTATACCCTAA

TUR216|AB863180.1| TCTTGGTGCAGCCAAAGGTAATCTAGCATACGGTAAGTTTATGTTTACTGTATACCCTAA

TUR220|AB863181.1| TCTTGGTGCAGCCAAAGGTAATCTAGCATACGGTAAGTTTATGTTTACTGTATACCCTAA

TUR239|AB863182.1| TCTTGGTGCAGCCAAAGGTAATCTAGCATACGGTAAGTTTATGTTTACTGTATACCCTAA

TUR244|AB863183.1| TCTTGGTGCAGCCAAAGGTAATCTAGCATACGGTAAGTTTATGTTTACTGTATACCCTAA

TUR249|AB863184.1| TCTTGGTGCAGCCAAAGGTAATCTAGCATACGGTAAGTTTATGTTTACTGTATACCCTAA

TUR263|AB863185.1| TCTCGGTGCAGCCAAAGGTAATCTAGCATACGGTAAGTTTATGTTTACTGTATACCCTAA

TUR278|AB863186.1| TCTTGGTGCTGCCAAAGGTAATCTCGCATACGGTAAGTTTATGTTTACTGTATATCCCAA

TUR279|AB863187.1| TCTTGGTGCAGCCAAAGGTAATCTAGCATACGGTAAATTTATGTTTACTGTATACCCTAA

TUR285|AB863188.1| TCTTGGTGCAGCCAAAGGTAATCTAGCATACGGTAAGTTTATGTTTACTGTATACCCTAA

TUR289|AB863189.1| CCTTGGTGCAGCCAAAGGTAATCTAGCTTACGGTAAGTTTATGTTTACTGTATACCCTAA

TUR303|AB863190.1| TCTTGGTGCAGCCAAAGGTAATCTAGCATACGGTAAGTTTATGTTTACTGTATACCCTAA

TUR306|AB863191.1| TCTTGGTGCAGCCAAAGGTAATCTCGCATACGGTAAGTTTATGTTTACTGTATACCCTAA

Cabb-S|NC_001497.1| GTTTGGAATAAGCCTTAACACCCAAAGACTTAACCAAACCCTAAGCCTTATTCATGATTT

CRO180A|AB863192.1| GTTTGGAATAAGCCTTAATACCCAAAGACTTAACCAAACCCTAAGCCTTATTCATGATTT

GRC83|AB863193.1| GTTTGGAATAAGCCTTAATACCCAAAGACTTAACCAAACCCTAAGCCTTATTCATGATTT

GRC84B|AB863194.1| GTTTGGAATAAGCCTTAATACCCAAAGACTTAACCAAACCCTAAGCCTTATTCATGATTT

GRC86B|AB863195.1| GTTTGGAATAAGCCTTAATACCCAAAGACTTAACCAAACCCTAAGCCTTATTCATGATTT

GRC86D|AB863196.1| GTTTGGAATAAGCCTTAATACCCAAAGACTTAACCAGACCCTAAGCCTTATTCATGATTT

GRC87E|AB863197.1| GTTTGGAATAAGCCTTAATACCCAAAGACTTAACCAAACCCTAAGCCTTATTCATGATTT

GRC87G|AB863198.1| GTTTGGAATAAGCCTTAATACCCAAAGACTTAACCAAACCCTAAGCCTTATTCATGATTT

GRC91B|AB863199.1| GTTTGGAATAAGCCTTAATACCCAAAGACTTAACCAAACCCTAAGCCTTATTCATGATTT

GRC92A|AB863200.1| GTTTGGAATAAGCCTTAATACCCAAAGACTTAACCAAACCCTAAGCCTTATTCATGATTT

GRC92C|AB863201.1| GTTTGGAATAAGCCTTAATACCCAAAGACTTAACCAAACCCTAAGCCTTATTCATGATTT

GRC92D|AB863202.1| GTTTGGAATAAGCCTTAACACCCAAAGACTTAACCAAACCCTAAGCCTTATTCATGATTT

IRN1|AB863136.1| GTTTGGAATAAGCCTTAATACCCAAAGACTTAACCAAACCCTAAGCCTAATTCATGATTT

IRN2|AB863137.1| GTTTGGAATAAGCCTTAACACCCAAAGACTTAACCAAACCCTAAGCCTTATTCATGACTT

IRN3|AB863138.1| GTTTGGAATAAGCCTTAATACCCAAAGACTTAACCAAACCCTAAGCCTAATTCATGATTT

IRN4|AB863139.1| GTTTGGAATAAGCCTTAATACCCAAAGACTTAACCAAACCCTAAGCCTAATTCATGATTT

IRN5|AB863140.1| GTTTGGAATAAGCCTTAATACCCAAAGACTTAACCAAACCCTAAGCCTAATTCATGATTT

IRN6|AB863141.1| GTTTGGAATAAGCCTTAATACCCAAAGACTTAACCAAACCCTAAGCCTGATTCATGATTT

IRN7|AB863142.1| GTTTGGAATAAGCCTTAATACCCAAAGACTTAACCAAACCCTAAGCCTAATTCATGATTT

IRN8|AB863143.1| GTTTGGAATAAGCCTTAATACCCAAAGACTTAACCAAACCCTAAGCCTAATTCATGATTT

IRN9|AB863144.1| GTTTGGAATAAGCCTTAATACCCAAAGACTTAACCAAACCCTAAGCCTGATTCATGATTT

IRN10|AB863145.1| GTTTGGAATAAGCCTTAATACCCAAAGACTTAACCAAACCCTAAGCCTAATTCATGATTT

IRN11|AB863146.1| GTTTGGAATAAGCCTTAACACCCAAAGACTTAACCAAACCCTAAGCCTTATTCATGACTT

IRN12|AB863147.1| GTTTGGAATAAGCCTTAATACCCAAAGACTTAACCAAACCCTAAGCCTAATTCATGATTT

IRN13|AB863148.1| GTTTGGAATAAGCCTTAATACCCAAAGACTTAACCAAACCCTAAGCCTAATTCATGATTT

IRN14|AB863149.1| GTTTGGAATAAGCCTCAATACCCAAAGACTTAACCAAACCCTAAGCCTGATTCATGATTT

IRN15|AB863150.1| GTTTGGGATAAGCCTTAATACCCAAAGACTTAACCAAACCCTAAGCCTTATTCATGATTT

IRN16|AB863151.1| GTTTGGAATAAGCCTTAATACCCAAAGACTTAACCAAACCCTAAGCCTTATTCATGATTT

IRN17|AB863152.1| GTTTGGAATAAGCCTTAATACCCAAAGACTTAACCAAACCCTAAGCCTTATTCATGATTT

IRN18|AB863153.1| GTTTGGAATAAGCCTTAATACCCAAAGACTTAACCAAACCCTAAGCCTGATTCATGATTT

IRN19|AB863154.1| GTTTGGAATAAGCCTTAACACCCAAAGACTTAACCAAACCCTAAGCCTTATTCATGACTT

IRN20|AB863155.1| GTTTGGGATAAGCCTTAATACCCAAAGACTTAACCAAACCCTAAGCCTTATTCATGATTT

IRN21|AB863156.1| GTTTGGAATAAGCCTTAATACCCAAAGACTTAACCAAACCCTAAGCCTGATTCATGATTT

JPNHGB340|AB863157.1| GTTTGGAATAAGCCTTAATACCCAAAGACTTAACCAAACCTTAAGCCTTATTCATGATTT

JPNKWB778|AB863158.1| GTTTGGAATAAGCCTTAATACCCAAAGACTTAACCAAACCTTAAGCCTTATTCATGATTT

JPNM|AB863159.1| GTTTGGAATAAGCCTTAATACCCAAAGACTTAACCAAACCTTAAGCCTTATTCATGATTT

JPNN|AB863160.1| GTTTGGAATAAGCCTTAATACCCAAAGACTTAACCAAACCCTAAGCCTTATTCATGATTT

JPNS1|AB863161.1| GTTTGGAATAAGCCTTAATACCCAAAGACTTAACCAAACCTTAAGCCTTATTCATGATTT

JPNS2|AB863162.1| GTTTGGAATAAGCCTTAATACCCAAAGACTTAACCAAACCTTAAGCCTTATTCATGATTT

JPNTKD762|AB863163.1| GTTTGGAATAAGCCTTAATACCCAAAGACTTAACCAAACCTTAAGCCTTATTCATGATTT

JPNUV1|AB863164.1| GTTTGGAATAAGCCTTAATACCCAAAGACTTAACCAAACCTTAAGCCTTATTCATGATTT

JPNUV26|AB863165.1| GTTTGGAATAAGCCTTAATACCCAAAGACTTAACCAAACCTTAAGCCTTATTCATGATTT

TUR1|AB863166.1| GTTTGGAATAAGCCTTAATACCCAAAGACTTAACCAAACCCTAAGCCTTATTCATGATTT

TUR2|AB863167.1| GTTTGGAATAAGCCTTAATACCCAAAGACTTAACCAAACCCTAAGCCTTATTCATGATTT

TUR4|AB863168.1| GTTTGGAATAAGCCTTAATACCCAAAGACTTAACCAAACCCTAAGCCTTATTCATGATTT

TUR5|AB863169.1| GTTTGGAATAAGCCTTAATACCCAAAGACTTAACCAAACCCTAAGCCTTATTCATGATTT

TUR12|AB863170.1| GTTTGGAATAAGCCTTAATACCCAAAGACTTAACCAAACCCTAAGCCTTATTCATGATTT

TUR34|AB863171.1| GTTTGGAATAAGCCTCAATACCCAAAGACTTAACCAAACCTTAAGCCTGATTCATGATTT

TUR50|AB863172.1| GTTTGGAATAAGCCTCAATACCCAAAGACTTAACCAAACCTTAAGCCTGATTCATGATTT

TUR59|AB863173.1| GTTTGGAATAAGCCTTAATACCCAGAGACTTAACCAAACCCTAAGCCTTATTCATGATTT

TUR69|AB863174.1| GTTTGGAATAAGCCTTAATACCCAAAGACTTAACCAAACCCTAAGCCTTATTCATGATTT

TUR81|AB863175.1| GTTTGGAATAAGCCTTAATACCCAAAGACTTAACCAAACCCTAAGCCTTATTCATGATTT

TUR84|AB863176.1| GTTTGGAATAAGCCTTAATACCCAAAGACTTAACCAAACCCTAAGCCTTATTCATGATTT

TUR94|AB863177.1| GTTTGGAATGAGCCTTAATACCCAAAGACTTAACCAAACCCTAAGCCTTATTCATGATTT

TUR213|AB863178.1| GTTTGGAATAAGCCTTAATACCCAAAGACTTAACCAAACCCTAAGCCTTATTCATGATTT

TUR214|AB863179.1| GTTTGGAATAAGCCTTAATACCCAAAGACTTAACCAAACCCTAAGCCTTATTCATGATTT

TUR216|AB863180.1| GTTTGGAATAAGCCTTAATACCCAAAGACTTAACCAAACCCTAAGCCTTATTCACGATTT

TUR220|AB863181.1| GTTTGGAATAAGCCTTAATACCCAAAGACTTAACCAAACCCTAAGCCTTATTCATGATTT

TUR239|AB863182.1| GTTTGGAATAAGCCTTAACACCCAAAGACTTAACCAAACCCTAAGCCTTATTCATGATTT

TUR244|AB863183.1| GTTTGGAATAAGCCTTAACACCCAAAGACTTAACCAAACCCTAAGCCTTATTCATGATTT

TUR249|AB863184.1| GTTTGGAATAAGCCTTAACACCCAAAGACTTAACCAAACCCTAAGCCTTATTCATGATTT

TUR263|AB863185.1| GTTTGGAATAAGCCTTAATACCCAAAGACTTAACCAAACCCTAAGCCTGATTCATGATTT

TUR278|AB863186.1| GTTTGGAATAAGCCTTAATACCCAAAGACTTAACCAAACCCTAAGCCTTATTCATGATTT

TUR279|AB863187.1| GTTTGGAATAAGCCTTAATACCCAGAGACTTAACCAAACCCTAAGCCTTATTCATGATTT

TUR285|AB863188.1| GTTTGGAATAAGCCTTAATACCCAAAGACTTAACCAAACTCTAAGCCTTATTCATGATTT

TUR289|AB863189.1| GTTTGGAATAAGCCTTAATACCCAAAGACTTAACCAAACCCTAAGCCTTATTCATGATTT

TUR303|AB863190.1| GTTTGGAATAAGCCTTAATACCCAAAGACTTAGCCAAACCCTAAGCCTTATTCATGATTT

TUR306|AB863191.1| GTTTGGAATAAGCCTTAGTACCCAAAGACTTAACCAAACCCTAAGCCTTATTCATGATTT

Cabb-S|NC_001497.1| TGAAAATAAAAATCTTATGAATAAAGGTGATAAAGTTATGACCATAACCTATGTCGTAGG

CRO180A|AB863192.1| TGAGAATAAAAATCTTATGAATAAAGGTGATAAAGTTATGACCATAACCTATATCGTAGG

GRC83|AB863193.1| TGAGAATAAAAATCTTATGAATAAAGGTGATAAAGTTATGACCATAACCTATATAGTAGG

GRC84B|AB863194.1| TGAGAATAAAAATCTTATGAATAAAGGTGATAAAGTTATGACCATAACCTATATAGTAGG

GRC86B|AB863195.1| TGAGAATAAAAATCTTATGAATAAAGGTGATAAAGTTATGACCATAACCTATATCGTAGG

GRC86D|AB863196.1| TGAGAATAAAAATCTTATGAATAAAGGTGATAAAGTTATGACCATAACCTATATAGTAGG

GRC87E|AB863197.1| TGAGAATAAAAATCTTATGAATAAAGGTGATAAAGTTATGACCATAACCTATATAGTAGG

GRC87G|AB863198.1| TGAGAATAAAAATCTTATGAATAAAGGTGATAAAGTTATGACCATAACCTATATAGTAGG

GRC91B|AB863199.1| TGAGAACAAAAATCTTATGAATAAAGGTGATAAAGTTATGACCATAACCTATATAGTAGG

GRC92A|AB863200.1| TGAAAATAAAAATCTTATGAATAAAGGTGATAAAGTTATGACCATAACCTATATCGTAGG

GRC92C|AB863201.1| TGAGAATAAAAATCTTATGAATAAAGGTGATAAAGTTATGACCATAACCTATATAGTAGG

GRC92D|AB863202.1| TGAGAATAAAAATCTTATGAATAAAGGTGATAAAGTTATGACCATAACCTATATAGTAGG

IRN1|AB863136.1| TGAGAATAAAAATCTCATGAACAAAGGCGATAAAGTTATGACCATAACCTATATTGTAGG

IRN2|AB863137.1| TGAAAATAAGAATCTCATGAATAAAGGTGATAAAGTTATGACCATAACCTATATTGTAGG

IRN3|AB863138.1| TGAGAATAAAAATCTCATGAACAAAGGCGATAAAGTTATGACCATAACCTATATTGTAGG

IRN4|AB863139.1| TGAGAATAAAAATCTCATGAATAAAGGTGATAAAGTTATGACCATAACCTATATAGTAGG

IRN5|AB863140.1| TGAGAATAAGAATCTCATGAATAAAGGTGATAAAGTTATGACCATAACCTATATAGTAGG

IRN6|AB863141.1| TGAGAATAAAAATCTCATGAACAAAGGCGATAAAGTTATGACCATAACCTATATTGTTGG

IRN7|AB863142.1| TGAGAATAAGAATCTCATGAATAAAGGTGATAAAGTTATGACCATAACCTATATAGTAGG

IRN8|AB863143.1| TGAGAATAAGAATCTCATGAATAAAGGTGATAAAGTTATGACCATAACCTATATAGTAGG

IRN9|AB863144.1| TGAGAATAAAAATCTCATGAACAAAGGCGATAAAGTTATGACTATAACCTATATAGTAGG

IRN10|AB863145.1| TGAAAATAAAAATCTCATGAACAAAGGCGATAAAGTTATGACCATAACCTATATTGTAGG

IRN11|AB863146.1| TGAAAATAAGAATCTCATGAATAAAGGTGATAAAGTTATGACCATAACCTATATTGTAGG

IRN12|AB863147.1| TGAGAATAAGAATCTCATGAATAAAGGTGATAAAGTTATGACCATAACCTATATAGTAGG

IRN13|AB863148.1| TGAGAATAAGAATCTCATGAATAAAGGTGATAAAGTTATGACCATAACCTATATAGTAGG

IRN14|AB863149.1| TGAGAATAAAAATCTCATGAACAAAGGCGATAAAGTTATGACCATAACCTATATTGTTGG

IRN15|AB863150.1| TGAAAATAAAAATCTTATGAATAAAGGTGATAAAGTTATGACCATAACCTATATAGTAGG

IRN16|AB863151.1| TGAAAATAAAAATCTTATGAATAAAGGTGATAAAGTTATGACCATAACCTATATAGTAGG

IRN17|AB863152.1| TGAAAATAAAAATCTTATGAATAAAGGTGATAAAGTTATGACCATAACCTATATAGTAGG

IRN18|AB863153.1| TGAAAATAAAAATCTCATGAACAAAGGTGATAAAGTTATGACCATAACCTATATTGTAGG

IRN19|AB863154.1| TGAAAATAAGAATCTCATGAATAAAGGTGATAAAGTTATGACCATAACCTATATTGTAGG

IRN20|AB863155.1| TGAAAATAAAAATCTTATGAATAAAGGTGATAAAGTTATGACCATAACCTATATAGTAGG

IRN21|AB863156.1| TGAGAATAAAAATCTCATGAACAAAGGCGATAAAGTTATGACCATAACCTATATTGTTGG

JPNHGB340|AB863157.1| TGAGAATAAAAATCTTATGAATAAAGGTGATAAAGTTATGACCATAACCTATATTGTAGG

JPNKWB778|AB863158.1| TGAGAATAAAAATCTTATGAATAAAGGTGATAAAGTTATGACCATAACCTATATCGTAGG

JPNM|AB863159.1| TGAGAATAAAAATCTTATGAATAAAGGTGATAAAGTTATGACCATAACCTATATTGTAGG

JPNN|AB863160.1| TGAGAATAAAAATCTTATGAATAAAGGTGATAAAGTTATGACCATAACCTATATTGTAGG

JPNS1|AB863161.1| TGAGAATAAAAATCTTATGAATAAAGGTGATAAAGTTATGACCATAACCTATATTGTAGG

JPNS2|AB863162.1| TGAGAATAAAAATCTTATGAATAAAGGTGATAAAGTTATGACCATAACCTATATTGTAGG

JPNTKD762|AB863163.1| TGAGAATAAAAATCTTATGAATAAAGGTGATAAAGTTATGACCATAACCTATATCGTAGG

JPNUV1|AB863164.1| TGAGAATAAAAATCTTATGAATAAAGGTGATAAAGTTATGACCATAACCTATATTGTAGG

JPNUV26|AB863165.1| TGAGAATAAAAATCTTATGAATAAAGGTGATAAAGTTATGACCATAACCTATATTGTAGG

TUR1|AB863166.1| TGAGAATAAAAATCTTATGAATAAAGGAGATAAAGTTATGACCATAACCTATATCGTAGG

TUR2|AB863167.1| TGAGAATAAAAATCTTATGAATAAAGGAGATAAAGTTATGACCATAACCTATATTGTAGG

TUR4|AB863168.1| TGAGAATAAAAATCTTATGAATAAAGGAGATAAAGTTATGACCATAACCTATATTGTAGG

TUR5|AB863169.1| TGAGAATAAAAATCTTATGAATAAAGGTGATAAAGTTATGACCATAACCTATATCGTAGG

TUR12|AB863170.1| TGAAAATAAAAATCTTATGAATAAAGGTGATAAAGTTATGACCATAACCTATATCGTAGG

TUR34|AB863171.1| TGAGAATAAAAATCTCATGAACAAAGGTGATAAAGTTATGACCATAACCTATATCGTAGG

TUR50|AB863172.1| TGAAAATAAAAATCTCATGAACAAAGGTGATAAAGTTATGACCATAACCTATATCGTAGG

TUR59|AB863173.1| TGAGAATAAAAATCTTATGAATAAAGGTGATAAAGTTATGACCATAACCTATATTGTAGG

TUR69|AB863174.1| TGAGAATAAAAATCTTATGAATAAAGGTGATAAAGTTATGACCATAACCTATATCGTAGG

TUR81|AB863175.1| TGAGAATAAAAATCTTATGAATAAAGGTGATAAAGTTATGACCATAACCTATATCGTAGG

TUR84|AB863176.1| TGAGAATAAAAATCTTATGAATAAAGGTGATAAAGTTATGACCATAACCTATATCGTAGG

TUR94|AB863177.1| TGAAAATAAAAATCTTATGAATAAAGGTGATAAAGTTATGACCATAACCTATATTGTAGG

TUR213|AB863178.1| TGAGAATAAAAATCTTATGAATAAAGGTGATAAAGTTATGACCATAACCTATATCGTAGG

TUR214|AB863179.1| TGAGAATAAAAATCTTATGAATAAAGGTGATAAAGTTATGACCATAACCTATATCGTAGG

TUR216|AB863180.1| TGAGAATAAAAATCTTATGAATAAAGGTGATAAAGTTATGACCATAACCTATATCGTAGG

TUR220|AB863181.1| TGAGAATAAAAATCTTATGAATAAAGGTGATAAAGTTATGACCATAACCTATATCGTAGG

TUR239|AB863182.1| TGAGAATAAAAATCTTATGAATAAAGGTGATAAAGTTATGACCATAACCTATATCGTAGG

TUR244|AB863183.1| TGAGAATAAAAATCTTATGAATAAAGGTGATAAAGTTATGACCATAACCTATATCGTAGG

TUR249|AB863184.1| TGAGAATAAAAATCTTATGAATAAAGGTGATAAAGTTATGACCATAACCTATATCGTAGG

TUR263|AB863185.1| TGAAAATAAAAATCTAATGAATAAAGGTGATAAAGTTATGACCATAACCTATATTGTAGG

TUR278|AB863186.1| TGAGAATAAAAATCTTATGAATAAAGGTGATAAAGTTATGACCATAACCTATATCGTAGG

TUR279|AB863187.1| TGAGAATAAAAATCTTATGAATAAAGGTGATAAAGTTATGACCATAACCTATATTGTAGG

TUR285|AB863188.1| TGAGAATAAAAATCTTATGAATAAAGGTGATAAAGTTATGACCATAACCTATATCGTAGG

TUR289|AB863189.1| TGAGAACAAAAATCTTATGAATAAAGGTGATAAAGTTATGACCATAACCTATATCGTAGG

TUR303|AB863190.1| TGAGAATAAAAATCTTATGAATAAAGGTGATAAAGTTATGACCATAACCTATATCGTAGG

TUR306|AB863191.1| TGAGAATAAAAATCTTATGAATAAAGGTGATAAAGTTATGACCATAACCTATATCGTAGG

Cabb-S|NC_001497.1| ATATGCATTAACTAATAGTCATCATAGCATAGATTATCAATCAAATGCTACAATTGAACT

CRO180A|AB863192.1| ATATGCATTAACTAATAGTCATCATAGCATAGATTATCAATCGAATGCTACAATTGAACT

GRC83|AB863193.1| ATATGCATTAGCAAATAGTCATCATAGCATAGATTATCAATCGAATGCTACAATTGAACT

GRC84B|AB863194.1| ATATGCATTAACAAATAGTCATCATAGCATAGATTATCAATCGAATGCTACAATTGAACT

GRC86B|AB863195.1| ATATGCATTAACAAATAGTCATCATAGCATAGATTATCAATCGAATGCTACAATTGAACT

GRC86D|AB863196.1| ATATGCATTAACAAATAGTCATCATAGCATAGATTATCAATCGAATGCTACAATTGAACT

GRC87E|AB863197.1| ATATGCATTAACAAATAGTCATCATAGCATAGATTATCAATCGAATGCTACAATTGAACT

GRC87G|AB863198.1| ATATGCATTAACAAATAGTCATCATAGCATAGATTATCAATCGAATGCTACAATTGAACT

GRC91B|AB863199.1| ATATGCATTAACAAATAGTCATCATAGCATAGATTATCAATCGAATGCTACAATTGAACT

GRC92A|AB863200.1| ATATGCATTAACAAATAGTCATCATAGCATAGATTATCAATCGAATGCTACAATTGAACT

GRC92C|AB863201.1| ATATGCATTAACAAATAGTCATCATAGCATAGATTATCAATCGAATGCTACAATTGAACT

GRC92D|AB863202.1| ATATGCATTAACAAATAGTCATCATAGCATAGATTATCAATCGAATGCTACAATTGAACT

IRN1|AB863136.1| ATATGCATTAACTAATAGTCATCATAGCATAGATTATCAATCGAATGCTACAATTGAACT

IRN2|AB863137.1| ATATGCATTAACTAATAGTCATCATAGCATAGATTATCAATCGAATGCTACAATTGAACT

IRN3|AB863138.1| ATATGCATTAACTAATAGTCATCATAGCATAGATTATCAATCGAATGCTACAATTGAACT

IRN4|AB863139.1| ATATGCATTAACAAATAGTCATCATAGCATAGATTATCAATCGAATGCTACAATTAAACT

IRN5|AB863140.1| ATATGCATTAACAAATAGTCATCATAGCATAGATTATCAATCGAATGCTACAATTGAACT

IRN6|AB863141.1| ATATGCATTAACTAATAGTCATCATAGCATAGATTATCAATCGAATGCTACAATTGAACT

IRN7|AB863142.1| ATATGCATTAACAAATAGTCATCATAGCATAGATTATCAATCGAATGCTACAATTGAACT

IRN8|AB863143.1| ATATGCATTAACAAATAGTCATCATAGCATAGATTATCAATCGAATGCTACAATTGAACT

IRN9|AB863144.1| ATATGCATTAACAAATAGTCATTATAGCATAGATTATCAATCGAATGCTACAATTGAACT

IRN10|AB863145.1| ATATGCATTAACTAATAGTCATCATAGCATAGATTATCAATCGAATGCTACAATTGAACT

IRN11|AB863146.1| ATATGCATTAACTAATAGTCATCATAGCATAGATTATCAATCGAATGCTACAATTGAACT

IRN12|AB863147.1| ATATGCATTAACAAATAGTCATCATAGCATAGATTATCAATCGAATGCTACAATTGAACT

IRN13|AB863148.1| ATATGCATTAACAAATAGTCATCATAGCATAGATTATCAATCGAATGCTACAATTGAACT

IRN14|AB863149.1| ATATGCATTAACTAATAGTCATCATAGCATAGATTATCAATCGAATGCTACAATTGAACT

IRN15|AB863150.1| ATATGCATTAACAAATAGTCATCATAGCATAGATTATCAATCGAATGCTACAATTGAACT

IRN16|AB863151.1| ATATGCATTAACAAATAGTCATCATAGCATAGATTATCAATCGAATGCTACAATTGAACT

IRN17|AB863152.1| ATATGCATTAACAAATAGTCATCATAGCATAGATTATCAATCGAATGCTACAATTGAACT

IRN18|AB863153.1| ATATGCATTAACAAATAGTCATCATAGCATAGATTATCAATCGAATGCTACAATTGAACT

IRN19|AB863154.1| ATATGCATTAACTAATAGTCATCATAGCATAGATTATCAATCGAATGCTACAATTGAACT

IRN20|AB863155.1| ATATGCATTAACAAATAGTCATCATAGCATAGATTATCAATCGAATGCTACAATTGAACT

IRN21|AB863156.1| ATATGCATTAACTAATAGTCATCATAGCATAGATTATCAATCGAATGCTACAATTGAACT

JPNHGB340|AB863157.1| ATATGCATTAACTAATAGTCATCATAGCATAGATTATCAATCGAATGCTACAATTGAACT

JPNKWB778|AB863158.1| ATATGCATTAACTAATAGTCATCATAGCATAGATTATCAATCAAATGCTACAATTGAACT

JPNM|AB863159.1| ATATGCATTAACTAATAGTCATCATAGCATAGATTATCAATCGAATGCTACAATTGAACT

JPNN|AB863160.1| ATATGCATTAACTAATAGTCATCATAGTATAGATTATCAATCTAATGCTACGATTGAACT

JPNS1|AB863161.1| ATATGCATTAACTAATAGTCATCATAGCATAGATTATCAATCAAATGCTACGATTGAACT

JPNS2|AB863162.1| ATATGCATTAACTAATAGTCATCATAGCATAGATTATCAATCAAATGCTACGATTGAACT

JPNTKD762|AB863163.1| ATATGCATTAACTAATAGTCATCATAGCATAGATTATCAATCGAATGCTACAATTGAACT

JPNUV1|AB863164.1| ATATGCATTAACTAATAGTCATCATAGCATAGATTATCAATCGAATGCTACAATTGAACT

JPNUV26|AB863165.1| ATATGCATTAACTAATAGTCATCATAGCATAGATTATCAATCGAATGCTACAATTGAACT

TUR1|AB863166.1| ATATGCATTAACTAATAGTCATCATAGCATAGATTATCAATCGAATGCTACAATTGAACT

TUR2|AB863167.1| ATATGCGTTAACAAATAGTCATCATAGCATAGATTATCAATCGAATGCTACAATTGAACT

TUR4|AB863168.1| ATATGCGTTAACAAATAGTCATCATAGCATAGATTATCAATCGAATGCTACAATTGAACT

TUR5|AB863169.1| ATATGCATTAACTAATAGTCATCATAGCATAGATTATCAATCGAATGCTACAATTGAACT

TUR12|AB863170.1| ATATGCATTAACTAATAGTCATCATAGCATAGATTATCAATCGAATGCTATAATTGAACT

TUR34|AB863171.1| ATATGCATTAACAAATAGTCATCATAGCATAGATTATCAATCGAATGCTACAATTGAACT

TUR50|AB863172.1| ATATGCATTAACAAATAGTCATCATAGCATAGATTATCAATCGAATGCTACAATTGAACT

TUR59|AB863173.1| ATATGCATTAACAAATAGTCATCATAGCATAGATTATCAATCGAATGCTACAATTGAACT

TUR69|AB863174.1| ATATGCATTAACTAATAGTCATCATAGCATAGATTATCAATCGAATGCTACAATTGAACT

TUR81|AB863175.1| ATATGCATTAACTAATAGTCATCATAGCATAGATTATCAATCGAATGCTACAATTGAACT

TUR84|AB863176.1| ATATGCATTAACTAATAGTCATCATAGCATAGATTATCAATCGAATGCTACAACTGAACT

TUR94|AB863177.1| ATATGCATTAACTAATAGTCATCATAGCATAGATTATCAATCGAATGCTACAATTGAACT

TUR213|AB863178.1| ATATGCATTAACTAATAGTCATCATAGCATAGATTATCAATCGAATGCTACAATTGAACT

TUR214|AB863179.1| ATATGCATTAACTAATAGTCATCATAGCATAGATTATCAATCAAATGCTACAATTGAACT

TUR216|AB863180.1| ATATGCATTAACAAATAGTCATCATAGCATAGATTATCAATCGAATGCTACAATTGAACT

TUR220|AB863181.1| ATATGCATTAACTAATAGTCATCATAGCATAGATTATCAATCGAATGCTACAATTGAACT

TUR239|AB863182.1| ATATGCATTAACTAATAGTCATCATAGCATAGATTATCAATCGAATGCTACAATTGAACT

TUR244|AB863183.1| ATATGCATTAACTAATAGTCATCATAGCATAGATTATCAATCGAATGCTACAATTGAACT

TUR249|AB863184.1| ATATGCATTAACTAATAGTCATCATAGCATAGATTATCAATCGAATGCTACAATTGAACT

TUR263|AB863185.1| ATATGCATTAACAAATAGTCATCATAGCATAGATTATCAATCGAATGCTACAATTGAACT

TUR278|AB863186.1| ATATGCATTAACTAATAGTCATCATAGCATAGATTATCAATCAAATGCTACAATTGAACT

TUR279|AB863187.1| ATATGCATTAACAAATAGTCATCATAGCATAGATTATCAATCGAATGCTACAATTGAACT

TUR285|AB863188.1| ATATGCATTAACTAATAGTCATCATAGCATAGATTATCAATCGAATGCTACAATTGAACT

TUR289|AB863189.1| ATATGCATTAACTAATAGTCATCATAGCATAGATTATCAATCAAATGCTACAATTGAACT

TUR303|AB863190.1| ATATGCATTAACTAATAGTCATCATAGCATAGATTATCAATCGAATGCTACAATTGAACT

TUR306|AB863191.1| ATATGCATTAACTAATAGTCATCATAGCATAGATTATCAATCGAATGCTACAATTGAACT

Cabb-S|NC_001497.1| AGAAGACGTATTTCAAGAAATTGGAAATGTCCAGCAATCTGAGTTCTGTACAATACAGAA

CRO180A|AB863192.1| AGAAGACGTATTTCAAGAAATTGGAAATGTCCAGCAATCTAATTTCTGTACAATACAGAA

GRC83|AB863193.1| AGAAGACGTATTTCAAGAAATTGGAAATGTCCAGCAATCTGAGTTCTGTACAATACAGAA

GRC84B|AB863194.1| AGAAGACGTATTTCAAGAAATTGGAAATGTCCAGCAATCTGAGTTCTGTACAATACAGAA

GRC86B|AB863195.1| AGAAGACGTATTTCAAGAAATTGGAAATGTCCAGCAATCTGAGTTCTGTACAATACAGAA

GRC86D|AB863196.1| AGAAGACGTATTTCAAGAAATTGGAAATGTCCAGCAATCTGAGTTCTGTACAATACAGAA

GRC87E|AB863197.1| AGAAGACGTATTTCAAGAAATTGGAAATGTCCAGCAATCTGAGTTCTGTACAATACAGAA

GRC87G|AB863198.1| AGAAGACGTATTTCAAGAAATTGGAAATGTCCAGCAATCTGAGTTCTGTACAATACAGAA

GRC91B|AB863199.1| AGAAGACGTATTTCAAGAAATTGGAAATGTCCAGCAATCTGAGTTCTGTACTATACAGAA

GRC92A|AB863200.1| AGAAGACGTATTTCAAGAAATTGGAAATGTCCAGCAATCTGAGTTCTGTACAATACAGAA

GRC92C|AB863201.1| AGAAGACGTATTTCAAGAAATTGGAAATGTCCAGCAATCTGAGTTCTGTACAATACAGAA

GRC92D|AB863202.1| AGAAGACGTATTTCAAGAAATTGGAAATGTCCAGCAATCTGAGTTCTGTACAATACAGAA

IRN1|AB863136.1| AGAAGACGTATTTCAAGAAATTGGAAATGTCCAGCAATCTGAGTTCTGTACAATACAGAA

IRN2|AB863137.1| AGAAGACGTATTTCAAGAAATTGGAAATGTCCAGCAATCTGAGTTCTGTACAATACAGAA

IRN3|AB863138.1| AGAAGACGTATTTCAAGAAATTGGAAATGTCCAGCAATCTGAGTTCTGTACAATACAGAA

IRN4|AB863139.1| AGAAGACGTATTTCAAGAAATTGGAAATGTCCAGCAATCTGAGTTCTGTACAATACAGAA

IRN5|AB863140.1| AGAAGACGTATTTCAAGAAATTGGAAATGTCCAGCAATCTGAGTTTTGTACAATACAGAA

IRN6|AB863141.1| AGAAGACGTATTTCAAGAAATTGGAAATGTCCAGCAATCTGAGTTTTGTACAATACAAAA

IRN7|AB863142.1| AGAAGACGTATTTCAAGAAATTGGAAATGTCCAGCAATCTGAGTTTTGTACAATACAGAA

IRN8|AB863143.1| AGAAGACGTATTTCAAGAAATTGGAAATGTCCAGCAATCTGAGTTTTGTACAATACAGAA

IRN9|AB863144.1| AGAAGACGTATTTCAAGAAATTGGAAATATCCAGCAATCTGAGTTTTGTGCAATACAAAA

IRN10|AB863145.1| AGAAGACGTATTTCAAGAAATTGGAAATGTCCAGCAATCTGAGTTCTGTACAATACAGAA

IRN11|AB863146.1| AGAAGACGTATTTCAAGAAATTGGAAATGTCCAGCAATCTGAGTTCTGTACAATACAGAA

IRN12|AB863147.1| AGAAGACGTATTTCAAGAAATTGGAAATGTCCAGCAATCTGAGTTCTGTACAATACAGAA

IRN13|AB863148.1| AGAAGACGTATTTCAAGAAATTGGAAATGTCCAGCAATCTGAGTTCTGTACAATACAGAA

IRN14|AB863149.1| AGAAGACGTATTTCAAGAAATTGGAAATGTCCAGCAATCTGAGTTTTGTACAATACAAAA

IRN15|AB863150.1| AGAAGACGTATTTCAAGAAATTGGAAATGTCCAGCAATCTGAGTTTTGTACAATACAGAA

IRN16|AB863151.1| AGAAGACGTATTTCAAGAAATTGGAAATATCCAGCAATCTGAGTTTTGTACAATACAAAA

IRN17|AB863152.1| AGAAGACGTATTTCAAGAAATTGGAAATGTCCAGCAATCTGAGTTTTGTACAATACAGAA

IRN18|AB863153.1| AGAAGACGTATTTCAAGAAATTGGAAATGTCCAGCAATCTGAGTTCTGTACAATACAGAA

IRN19|AB863154.1| AGAAGACGTATTTCAAGAAATTGGAAATGTCCAGCAATCTGAGTTCTGTACAATACAGAA

IRN20|AB863155.1| AGAAGACGTATTTCAAGAAATTGGAAATGTCCAGCAATCTGAGTTTTGTACAATACAAAA

IRN21|AB863156.1| AGAAGACGTATTTCAAGAAATTGGAAATGTCCAGCAATCTGAGTTCTGTACAATACAGAA

JPNHGB340|AB863157.1| AGAAGACGTATTTCAAGAAATTGGAAATGTCCAGCAATCTGATTTTTGTACAATACAAAA

JPNKWB778|AB863158.1| AGAAGACGTATTTCAAGAAATTGGAAATGTCCAGCAATCTGATTTTTGTACAATACAAAA

JPNM|AB863159.1| AGAAGACGTATTTCAAGAAATTGGAAATGTCCAGCAATCTGATTTTTGTACAATACAAAA

JPNN|AB863160.1| AGAAGACGTATTTCAAGAAATTGGAAATGTCCAGCAATCTGATTTTTGTACAATACAAAA

JPNS1|AB863161.1| AGAAGACGTATTTCAAGAAATTGGAAATGTCCAGCAATCTGATTTTTGTACAATACAAAA

JPNS2|AB863162.1| AGAAGACGTATTTCAAGAAATTGGAAATGTCCAGCAATCTGATTTTTGTACAATACAAAA

JPNTKD762|AB863163.1| AGAAGACGTATTTCAAGAAATTGGAAATGTCCAGCAATCTGATTTTTGTACAATACAAAA

JPNUV1|AB863164.1| AGAAGACGTATTTCAAGAAATTGGAAATGTCCAGCAATCTGATTTTTGTACAATACAAAA

JPNUV26|AB863165.1| AGAAGACGTATTTCAAGAAATTGGAAATGTCCAGCAATCTGATTTTTGTACAATACAAAA

TUR1|AB863166.1| AGAAGACGTATTTCAAGAAATTGGAAATGTCCAGCAATCTGAGTTCTGTACAATACAGAA

TUR2|AB863167.1| AGAAGACGTATTTCAAGAAATTGGAAATGTCCAGCAATCTGAGTTCTGTACAATACAGAA

TUR4|AB863168.1| AGAAGACGTATTTCAAGAAATTGGAAATGTCCAGCAATCTGAGTTCTGTACAATACAGAA

TUR5|AB863169.1| AGAAGACGTATTTCAAGAAATTGGAAATGTCCAGCAATCTGAGTTCTGTACAATACAGAA

TUR12|AB863170.1| AGAAGACGTATTTCAAGAAATTGGAAATGTCCAGCAATCTGAGTTCTGTACAATACAGAA

TUR34|AB863171.1| AGAAGACGTATTTCAAGAAATTGGAAATGTCCAGCAATCTGAGTTCTGTACAATACAGAA

TUR50|AB863172.1| AGAAGACGTATTTCAAGAAATTGGAAATGTCCAGCAATCTGAGTTCTGTACAATACAGAA

TUR59|AB863173.1| AGAAGACGTATTTCAAGAAATTGGAAATGTCCAGCAATCTGAGTTCTGTACAATACAGAA

TUR69|AB863174.1| AGAAGACGTATTTCAAGAAATTGGAAATGTCCAGCAATCTGAGTTCTGTACAATACAGAA

TUR81|AB863175.1| AGAAGACGTATTTCAAGAAATTGGAAATGTCCAGCAATCTGAGTTCTGTACAATACAGAA

TUR84|AB863176.1| AGAAGACGTATTTCAAGAAATTGGAAATGTCCAGCAATCTGAGTTCTGTACAATACAGAA

TUR94|AB863177.1| AGAAGACGTATTTCAAGAAATTGGAAATGTCCAGCAATCTGAATTCTGTACAATACAGAA

TUR213|AB863178.1| AGAAGACGTATTTCAAGAAATTGGAAATGTCCAGCAATCTGAGTTTTGTACAATACAGAA

TUR214|AB863179.1| AGAAGACGTATTTCAAGAAATTGGAAATGTCCAGCAATCTGAGTTTTGTACAATACAGAA

TUR216|AB863180.1| AGAAGACGTATTTCAAGAAATTGGAAATGTCCAGCAATCTGAATTCTGTACAATACAGAA

TUR220|AB863181.1| AGAAGACGTATTTCAAGAAATTGGAAATGTCCAGCAATCTGAGTTCTGTACAATACAGAA

TUR239|AB863182.1| AGAAGACGTATTTCAAGAAATTGGAAATGTCCAGCAATCTGAGTTTTGTACAATACAGAA

TUR244|AB863183.1| AGAAGACGTATTTCAAGAAATTGGAAATGTCCAGCAATCTGAGTTTTGTACAATACAGAA

TUR249|AB863184.1| AGAAGACGTATTTCAAGAAATTGGAAATGTCCAGCAATCTGAGTTTTGTACAATACAGAA

TUR263|AB863185.1| AGAAGACGTATTTCAAGAAATTGGAAATGTCCAGCAATCTGAGTTCTGTACAATACAGAA

TUR278|AB863186.1| AGAAGACGTATTTCAAGAAATTGGAAATGTCCAGCAATCTGAATTCTGTACAATACAGAA

TUR279|AB863187.1| AGAAGACGTATTTCAAGAAATTGGAAATGTCCAGCAATCTGAGTTCTGTACAATACAGAA

TUR285|AB863188.1| AGAAGACGTATTTCAAGAAATTGGAAATGTCCAGCAATCTGAGTTTTGTACAATACAGAA

TUR289|AB863189.1| AGAAGACGTATTTCAAGAAATTGGAAATGTCCAGCAATCTGAGTTCTGTACAATACAGAA

TUR303|AB863190.1| AGAAGACGTATTTCAAGAAATTGGAAATGTCCAGCAATCTGAGTTCTGTACAATACAGAA

TUR306|AB863191.1| AGAAGACGTATTTCAAGAAATTGGAAATGTCCAGCAATCTGAGTTCTGTACAATACAGAA

Cabb-S|NC_001497.1| TGATGAATGCAATTGGGCCATTGATATAGCCCAAAACAAAGCCTTATTAGGAGCTAAAAC

CRO180A|AB863192.1| TGATGAATGCAATTGGGCCATTGATATAGCCCAAAACAAAGCCTTATTAGGAGCTAAAAC

GRC83|AB863193.1| TGATGAATGCAATTGGGCCATTGATATAGCCCAAAACAAAGCCTTATTAGGAGCTAAAAC

GRC84B|AB863194.1| TGATGAATGCAATTGGGCCATTGATATAGCCCAAAACAAAGCCTTATTAGGAGCTAAAAC

GRC86B|AB863195.1| TGATGAATGCAATTGGGCCATTGATATAGCCCAAAACAAAGCCTTATTAGGAGCTAAAAC

GRC86D|AB863196.1| TGATGAATGCAATTGGGCCATTGATATAGCCCAAAACAAAGCCTTATTAGGAGCTAAAAC

GRC87E|AB863197.1| TGATGAATGCAATTGGGCCATTGATATAGCTCAAAACAAAGCCTTATTAGGAGCTAAAAC

GRC87G|AB863198.1| TGATGAATGCAATTGGGCCATTGATATAGCTCAAAACAAAGCCTTATTAGGAGCTAAAAC

GRC91B|AB863199.1| TGATGAATGCAATTGGGCCATTGATATAGCCCAAAACAAAGCCTTATTAGGAGCTAAAAC

GRC92A|AB863200.1| TGATGAATGCAATTGGGCCATTGATATAGCCCAAAACAAAGCCTTATTAGGAGCTAAAAC

GRC92C|AB863201.1| TGATGAATGCAATTGGGCCATTGATATAGCCCAAAACAAAGCCTTATTAGGAGCTAAAAC

GRC92D|AB863202.1| TGATGAATGCAATTGGGCCATTGATATAGCCCAAAACAAAGCCTTATTAGGAGCTAAAAC

IRN1|AB863136.1| TGATGAATGCAATTGGGCCATTGATATAGCCCAAAACAAAGCCTTATTAGGAGCTAAAAC

IRN2|AB863137.1| TGATGAATGCAATTGGGCCATTGATATAGCCCAAAACAAAGCCTTATTAGGAGCTAAAGC

IRN3|AB863138.1| TGATGAATGCAATTGGGCCATTGATATAGCCCAAAACAAAGCCTTATTAGGAGCTAAAAC

IRN4|AB863139.1| TGATGAATGCAATTGGGCCATTGATATAGCCCAAAACAAAGCCTTATTAGGAGCTAAAAC

IRN5|AB863140.1| TGACGAATGCAATTGGGCCATTGATATAGCCCAAAACAAAGCCTTATTAGGAGCTAAAAC

IRN6|AB863141.1| TGACGAATGCAATTGGGCCATTGATATAGCCCAAAACAAAGCCTTATTAGGAGCTAAAAC

IRN7|AB863142.1| TGACGAATGCAATTGGGCCATTGATATAGCCCAAAACAAAGCCTTATTAGGAGCTAAAGC

IRN8|AB863143.1| TGACGAATGCAATTGGGCCATTGATATAGCCCAAAACAAAGCCTTATTAGGAGCTAAAAC

IRN9|AB863144.1| TGACGAATGCAATTGGGCCATTGATATAGCCCAAAACAAAGCCTTATTAGGAGCTAAAAC

IRN10|AB863145.1| TGATGAATGCAATTGGGCCATTGATATAGCCCAAAACAAAGCCTTATTAGGAGCTAAAGC

IRN11|AB863146.1| TGATGAATGCAATTGGGCCATTGATATAGCCCAAAACAAAGCCTTATTAGGAGCTAAAGC

IRN12|AB863147.1| TGATGAATGCAATTGGGCCATTGATATAGCCCAAAACAAAGCCTTATTAGGAGCTAAAAC

IRN13|AB863148.1| TGATGAATGCAATTGGGCCATTGATATAGCCCAAAACAAAGCCTTATTAGGAGCTAAAAC

IRN14|AB863149.1| TGACGAATGCAATTGGGCCATTGATATAGCCCAAAACAAAGCCTTATTAGGAGCTAAAAC

IRN15|AB863150.1| TGACGATTGTAATTGGGCCATTGATATAGCCCAAAACAAAGCCTTATTAGGAGCTAAAAC

IRN16|AB863151.1| TGACGATTGTAATTGGGTCATTGATATAGCCCAAAACAAAGCCTTATTAGGAGCTAAAAC

IRN17|AB863152.1| TGACGATTGTAATTGGGCCATTGATATAGCCCAAAACAAAGCCTTATTAGGAGCTAAAAC

IRN18|AB863153.1| TGATGAATGCAATTGGGCCATTGATATAGCCCAAAACAAAGCCTTATTAGGAGCTAAAGC

IRN19|AB863154.1| TGATGAATGCAATTGGGCCATTGATATAACCCAAAACAAAGCCTTATTAGGAGTTAAAGC

IRN20|AB863155.1| TGACGATTGTAATTGGGCCATTGATATAGCCCAAAACAAAGCCTTATTAGGAGCTAAAAC

IRN21|AB863156.1| TGATGAATGCAATTGGGCCATCGATATAGCCCAAAACAAAGCCTTATTAGGAGCTAAAAC

JPNHGB340|AB863157.1| TGACGAATGCAATTGGGCCATTGATATAGCCCAAAACAAAGCCTTATTAGGAGCTAAAAC

JPNKWB778|AB863158.1| TGACGAATGCAATTGGGCCATTGATATAGCCCAAAACAAAGCCTTATTAGGAGCTAAAAC

JPNM|AB863159.1| TGATGAATGCAATTGGGCCATTGATATAGCCCAGAACAAAGCCTTATTAGGAGCTAAAAC

JPNN|AB863160.1| TGACGAATGCAATTGGGCCATAGATATAGCCCAAAACAAAACCTTATTAGGAGCTAAAAC

JPNS1|AB863161.1| TGACGAATGCAATTGGGCCATAGATATAGCCCAAAACAAAGCCTTATTAGGAGCTAAAAC

JPNS2|AB863162.1| TGACGAATGCAATTGGGCCATAGATATAGCCCAAAACAAAGCCTTATTAGGAGCTAAAAC

JPNTKD762|AB863163.1| TGACGAATGCAATTGGGCCATTGATATAGCCCAAAACAAAGCCTTATTAGGAGCTAAAAC

JPNUV1|AB863164.1| TGATGAATGCAATTGGGCCATTGATATAGCCCAGAACAAAGCCTTATTAGGAGCTAAAAC

JPNUV26|AB863165.1| TGATGAATGCAATTGGGCCATTAATATAGCCCAGAACAAAGCCTTATTAGGAGCTAAAAC

TUR1|AB863166.1| TGATGAATGTAATTGGGCCATTGATATAGCCCAAAACAAAGCCTTATTAGGAGCTAAAGC

TUR2|AB863167.1| TGATGAATGCAATTGGGCCATTGATATAGCCCAAAACAAAGCCTTATTAGGAGCTAAAAC

TUR4|AB863168.1| TGATGAATGCAATTGGGCCATTGATATAGCCCAAAACAAAGCCTTATTAGGAGCTAAAAC

TUR5|AB863169.1| TGATGAATGCAATTGGGCCATTGATATAGCCCAAAACAAAGCCTTATTAGGAGCTAAAAC

TUR12|AB863170.1| TGATGAATGCAATTGGGCCATTGATATAGCCCAAAACAAAGCCTTATTAGGAGCTAAAAC

TUR34|AB863171.1| TGATGAATGCAATTGGGCCATTGATATAGCCCAAAACAAAGCCTTATTAGGAGCTAAAAC

TUR50|AB863172.1| TGATGAATGCAATTGGGCCATTGATATAGCCCAAAACAAAGCCTTATTAGGAGCTAAAAC

TUR59|AB863173.1| TGATGAATGCAATTGGGCCATTGATATAGCCCAAAACAAAGCCTTATTAGGAGCTAAAAC

TUR69|AB863174.1| TGATGAATGCAATTGGGCCATTGATATAGCCCAAAACAAAGCCTTATTAGGAGGTAAAAC

TUR81|AB863175.1| TGATGAATGCAATTGGGCCATTGATATAGCCCAAAACAAAGCCTTATTAGGAGGTAAAAC

TUR84|AB863176.1| TGATGAATGCAATTGGGCCATTGATATAGCCCAAAACAAAGCCTTATTAGGAGCTAAAAC

TUR94|AB863177.1| TGATGAATGCAATTGGGCCATTGATATAGCCCAAAACAAAGCCTTATTAGGAGCTAAAAC

TUR213|AB863178.1| CGATGAATGCAATTGGGCCATTGATATAGCCCAAAACAAAGCCTTATTAGGAGCTAAAGC

TUR214|AB863179.1| CGATGAATGCAATTGGGCCATTGATATAGCCCAAAACAAAGCCTTATTAGGAGCTAAAGC

TUR216|AB863180.1| TGATGAATGCAATTGGGCCATTGATATAGCCCAAAACAAAGCCTTATTAGGAGCTAAAGC

TUR220|AB863181.1| TGATGAATGCAATTGGGCCATTGATATAGCCCAAAACAAAGCCTTATTAGGAGCTAAAAC

TUR239|AB863182.1| CGATGAATGCAATTGGGCCATTGATATAGCCCAAAACAAAGCCTTATTAGGAGCTAAAGC

TUR244|AB863183.1| CGACGAATGCAATTGGGCCATTGATATAGCCCAAAACAAAGCCTTATTAGGAGCTAAAGC

TUR249|AB863184.1| CGATGAATGCAATTGGGCCATTGATATAGCCCAAAACAAAGCCTTATTAGGAGCTAAAGC

TUR263|AB863185.1| TGATGAATGCAATTGGGCCATTGATATAGCCCAAAACAAAGCCTTATTAGGAGCTAAAAC

TUR278|AB863186.1| TGATGAATGCAATTGGGTCATTGATATAGCCCAAAACAAAGCCTTATTAGGAGCTAAAAC

TUR279|AB863187.1| TGATGAATGCAATTGGGCCATTGATATAGCCCAAAACAAAGCCTTATTAGGAGCTAAAAC

TUR285|AB863188.1| CGATGAATGCAATTGGGCCATTGACATAGCCCAAAACAAAGCCTTATTAGGAGCTAAAAC

TUR289|AB863189.1| TGATGAATGCAATTGGGCCATTGATATAGCCCAAAACAAAGCCTTATTAGGAGCTAAAAC

TUR303|AB863190.1| TGATGAATGCAATTGGGCCATTGATATAGCCCAAAACAAAGCCTTATTAGGAGCTAAAGC

TUR306|AB863191.1| TGATGAATGCAATTGGGCCATTGATATAGCCCAAAACAAAGCCTTATTAGGAGCTAAAAC

Cabb-S|NC_001497.1| CAAGACTCAAATTGGTAATAACCTTCAAATAG**GT**AACAGTGCTTCATCCTCTAATACTGA

**D3**

CRO180A|AB863192.1| CAAAACCCAAATTGGTAATAGTCTTCAAATAGGAAACGGTGCTTCATCCTCTAATACTGA

GRC83|AB863193.1| CAAAACCCAAATTGGTAATAGTCTTCAAATAAGAAACAGCGCTTCATCCTCTAATACTGA

GRC84B|AB863194.1| CAAAACCCAAATTGGTAATAGTCTTCAAATAGGAAACAGCGCTTCATCCTCTAATACTGA

GRC86B|AB863195.1| CAAAACCCAAATTGGTAATAGCCTTCAAATAGGGAACAGCGCTTCATCCTCTAATACTGA

GRC86D|AB863196.1| CAAAACCCAAATTGGCAATAGTCTTCAAATAGGAAACAGCGCTTCATCCTCTAATACTGA

GRC87E|AB863197.1| CAAAACCCAAATTGGTAATAGTCTTCAAATAGGAAACAGCGCTTCATCCTCTAATACTGA

GRC87G|AB863198.1| CAAAACCCAAATTGGTAATAGTCTTCAAATAGGAAACAGCGCTTCATCCTCTAATACTGA

GRC91B|AB863199.1| CAAAACCCAAATTGGTAATAGTCTTCAAATAGGAAACAGCGCTTCATCCTCTAATACTGA

GRC92A|AB863200.1| CAAAACCCAAATTGGTAATAGTCTTCAAATAGGAAACAGCGCTTCATCCTCTAATACTGA

GRC92C|AB863201.1| CAAAACCCAAATTGGTAATAGTCTTCAAATAGGAAACAGCGCTTCATCCTCTAATACTGA

GRC92D|AB863202.1| CAAAACCCAAATTGGTAATAGTCTTCAAATAGGAAACAGCGCTTCATCCTCTAATACTGA

IRN1|AB863136.1| CAAAACCCAAATTGGTAATAATCTTCAAATAGGAAACAGCGCATCATCCTCTAATACTGA

IRN2|AB863137.1| CAAAACCCAAATTGGTAATAGTCTTCAAATAAGTAACAGCGCTTCATCCTCTAATACTGA

IRN3|AB863138.1| CAAAACCCAAATTGGTAATAATCTTCAAATAGGAAACAGCGCATCATCCTCTAATACTGA

IRN4|AB863139.1| CAAAACCCAAATTGGTAATAGTCTTCAAATAGGAAACAGCGCATCATCCTCTAATACTGA

IRN5|AB863140.1| CAAAACCCAAATTGGTAATAGTCTTCAAATAGGAAACAGCGCATCATCCTCTAATACTGA

IRN6|AB863141.1| CAAAACCCAAATTGGTAATAGTCTTCAAATAGGAAACAGTGCTTCATCCTCTAATACTGA

IRN7|AB863142.1| CAAAACCCAAATTGGTAATAGTCTTCAAATAGGAAACAGCGCATCATCCTCTAATACTGA

IRN8|AB863143.1| CAAAACCCAAATTGGTAATAGTCTTCAAATAGGAAACAGCGCATCATCCTCTAATACTGA

IRN9|AB863144.1| CAAAACCCAAATTGGTAATAGTCTTCAAATAGGAAACAGTGCTTCATCCTCTAATACTGA

IRN10|AB863145.1| CAAAACCCAAATTGGTAATAGTCTTCAAATAAGTAACAGCGCTTCATCCTCTAATACTGA

IRN11|AB863146.1| CAAAACCCAAATTGGTAATAGTCTTCAAATAAGTAACAGCGCTTCATCCTCTAATACTGA

IRN12|AB863147.1| CAAAACCCAAATTGGTAATAGTCTTCAAATAGGAAACAGTGCTTCATCCTCTAATACTGA

IRN13|AB863148.1| CAAAACCCAAATTGGTAATAGTCTTCAAATAGGAAACAGTGCTTTATCCTCTAATACTGA

IRN14|AB863149.1| CAAAACCCAAATTGGTAATAGTCTTCAAATAGGAAACAGTGCTTCATCCTCTAATACTGA

IRN15|AB863150.1| CAAAACCCAAATTGGTAATAGTCTTCAAATAGGAAACGGTGCTTTATCCTCTAATACTGA

IRN16|AB863151.1| CAAAACCCAAATTGGTAATAGTCTTCAAATAGGAAACAGTGCTTCATCCTCTAATACTGA

IRN17|AB863152.1| CAAAACCCAAATTGGTAATAGTCTTCAAATAGGAAACGGTGCTTTATCCTCTAATACTGA

IRN18|AB863153.1| CAAAACCCAAATTGGTAATAGTCTTCAAATAGGAAACAGCGCTTCATCCTCTAATACTGA

IRN19|AB863154.1| CAAAACCCAAATTGGTAATAGTCTTCAAATAAGTAACAGCGCTTCAACCTCTAATACTGA

IRN20|AB863155.1| CAAAACCCAAATTGGTAATAGTCTTCAAATAGGAAACAGTGCTTCATCCTCTAATACTGA

IRN21|AB863156.1| CAAAACCCAAATTGGTAATAGTCTTCAAATAGGAAACAGTGCTTCATCCTCTAATACTGA

JPNHGB340|AB863157.1| CCAATCCCAAATTGGTAATAGTCTTCAAATAGGAAACAGTGCTTCATCCTCTAATACTGA

JPNKWB778|AB863158.1| CCAATCCCAAATTGGCAATAGTCTTCAAATAAGAAACAGTGCTTCATCCTCTAATACTGA

JPNM|AB863159.1| CCAATCCCAAATTGGTAATAGTCTTCAAATAGGAAACAGTGCTTCATCCTCTAATACTGA

JPNN|AB863160.1| CAAATCCCAAATTGGTAATAGTCTTCAAATAGGAAATAGTGCTTCATCCTCTAATACTGA

JPNS1|AB863161.1| CAAATCCCAAATTGGTAATAGTCTTCAAATAGGAAACAGTGCTTCATCCTCTAATACTGA

JPNS2|AB863162.1| CAAATCCCAAATTGGTAATAGTCTTCAAATAGGAAACAGTGCTTCATCCTCTAATACTGA

JPNTKD762|AB863163.1| CCAATCCCAAATTGGTAATAGTCTTCAAATAGGAAACAGTGCTTCATCCTCTAATACTGA

JPNUV1|AB863164.1| CCAATCCCAAATTGGTAATAGTCTTCAAATAGGAAACAGTGCTTCATCCTCTAATACTGA

JPNUV26|AB863165.1| CCAATCCCAAATTGGTAATAGTCTTCAAATAGGAAACAGTGCTTCATCCTCTAATACTGA

TUR1|AB863166.1| CAAAACCCAAATTGGTAATAGCCTTCAAATAAGAAACAGCGCTTCATCCTCTAATACTGA

TUR2|AB863167.1| CAAAACCCAAATTGGTAATAGTCTTCAAATAGGGGACAGCGCTTCATCCTCTAATACTGA

TUR4|AB863168.1| CAAAACCCAAATTGGTAATAGTCTTCAAATAGGGGACAGCGCTTCATCCTCTAATACTGA

TUR5|AB863169.1| CAAAACCCAAATTGGTAATAGTCTTCAAATAGGAAACAGTGCTTCATCCTCTAATACTGA

TUR12|AB863170.1| CAAAACCCAAATTGGTAATAGTCTTCAAATAGGAAACAGCGCTTCATCCTCTAATACTGA

TUR34|AB863171.1| CAAAACCCAAATTGGTAATAGCCTTCAAATAGGAAATAGCGCTTCATCCTCTAATACTGA

TUR50|AB863172.1| CAAAACCCAAATTGGTAATAGCCTTCAAATAAGAAACAGCGCTTCATCCTCTAATACTGA

TUR59|AB863173.1| CAAAACCCAAATTGGTAATAGTCTTCAAATAGGAAACAGCGCTTCATCCTCTAATACTGA

TUR69|AB863174.1| CAAAACCCAAATTGGTAATAGCCTTCAAATAGGAAACAGCGCTTCATCCTCTAATACTGA

TUR81|AB863175.1| CAAAACCCAAATTGGTAATAGCCTTCAAATAGGAAACAGCGCTTCATCCTCTAATACTGA

TUR84|AB863176.1| CAAAACCCAAATTGGTAATAGTCTTCAAATAGGAAACAGCGCTTCATCCTCTAATACTGA

TUR94|AB863177.1| CAAAACCCAAATTGGTAATAGTCTTCAAATAGGAAACAGCGCTTCATCCTCTAATACTGA

TUR213|AB863178.1| CAAAACCCAAATTGGTAATAGTCTTCAAATAGGAAACAGTGCTTCATCCTCTAATACTGA

TUR214|AB863179.1| CAAAACCCAAATTGGTAATAGTCTTCAAATAGGAAATAGAGCTTCATCCTCTAATACTGA

TUR216|AB863180.1| CAAAACCCAAATTGGTAATAGTTTTCAAATAGGAAACAGTGCATCATCCTCTAATACTGA

TUR220|AB863181.1| CAAAACCCAAATTGGTAATAGTCTTCAAATAGGAAACAGCGCTTCATCCTCTAATACTGA

TUR239|AB863182.1| CAAAACCCAAATTGGTAATAGTCTTCAAATAAGAAATAGTGCTTCATCCTCTAATACTGA

TUR244|AB863183.1| CAAAACCCAAATTGGTAATAGTCTTCAAATAGGAAATAGTGCTTCATCCTCTAATACTGA

TUR249|AB863184.1| CAAAACCCAAATTGGTAATAGTCTTCAAATAAGAAACAGTGCTTCATCCTCTAATACTGA

TUR263|AB863185.1| CAAAACCCAAATTGGTAATAATCTTCAAATAGGAAACAGCGCTTCATCCTCTAATACTGA

TUR278|AB863186.1| CAAAACCCAAATTGGTAATAGCCTTCAAATAGGAAACAGCGCTTCATCCTCTAATACTGA

TUR279|AB863187.1| CAAAACCCAAATTGGTAATAGTCTTCAAATAGGAAACAGCGCTTCATCCTCTAATACTGA

TUR285|AB863188.1| CAAAACCCAAATTGGTAATAGTCTTCAAATAGGAAACAGCGCTTCATCCTCTAATACTGA

TUR289|AB863189.1| CAAAACCCAAATTGGTAATAGCCTTCAAATAGGAAACAGCGCTTCATCCTCTAATACTGA

TUR303|AB863190.1| CAAAACCCAAATTGGTAATAGTCTTCAAATAGGAAACAATGCTTCATCCTCTAATACTGA

TUR306|AB863191.1| CAAAACCCAAATTGGTAATAGTCTTCAAATAGGAAACAGTGCTTCATCCTCTAATACTGA

Cabb-S|NC_001497.1| AAATGAATTAGCTAGGGTAAGCCAGAACATAGATCTTTTAAAGAATAAATTAAAAGAAAT

CRO180A|AB863192.1| AAATGAATTAGCTAGGGTAAGCCAAAACATAGATCTTTTAAAGAATAAATTAAAAGAAAT

GRC83|AB863193.1| AAATGAGTTAGCTAGGGTAAGCCAAAACATAGATCTTTTAAAGAATAAATTAAAAGAAAT

GRC84B|AB863194.1| AAATGAATTAGCTAGGGTAAGCCAAAACATAGATCTTTTAAAGAATAAATTAAAAGAAAT

GRC86B|AB863195.1| AAATGAATTAGCTAGGGTAAGCCAAAACATAGATCTTTTAAAGAATAAATTAAAAGAAAT

GRC86D|AB863196.1| AAATGAATTAGCTAGGGTAAGCCAAAACATAGATCTTTTAAAGAATAAATTAAAAGAAAT

GRC87E|AB863197.1| AAATGAATTAGCTAGGGTAAGCCAAAACATAGATCTTTTAAAGAATAAATTAAAAGAAAT

GRC87G|AB863198.1| AAATGAATTAGCTAGGGTAAGCCAAAACATAGATCTTTTAAAGAATAAATTAAAAGAAAT

GRC91B|AB863199.1| AAATGAATTAGCTAGGGTAAGCCAAAACATAGATCTTTTAAAGAATAAATTAAAAGAAAT

GRC92A|AB863200.1| AAATGAATTAGCTAGGGTAAGCCAAAACATAGATCTTTTAAAGAGTAAATTAAAAGAAAT

GRC92C|AB863201.1| AAATGAATTAGCTAGGGTAAGCCAAAACATAGATCTTTTAAAGAGTAAATTAAAAGAAAT

GRC92D|AB863202.1| AAATGAATTAGCTAGGGTAAGCCAAAACATAGATCTTTTAAAGAATAAATTAAAAGAAAT

IRN1|AB863136.1| AAGTGAATTAGCAAGGGTGAGCCAAAACATAGATCTTTTAAAGAATAAATTAAAAGAAAT

IRN2|AB863137.1| AAATGAATTAGCTAGGGTAAGCCAAAACATAGATCTTTTAAAGAATAAATTAAAAGAAAT

IRN3|AB863138.1| AAGTGAATTAGCAAGGGTGAGCCAAAACATAGATCTTTTAAAGAATAAATTAAAAGAAAT

IRN4|AB863139.1| AAATGAATTAGCAAGGGTGAGCCAAAACATAGATCTTTTAAAGAATAAATTAAAAGAAAT

IRN5|AB863140.1| AAATGAATTAGCAAGGGTGAGCCAAAACATAGATCTTTTAAAGAATAAATTAAAAGAAAT

IRN6|AB863141.1| AAATGAATTAGCTAGGGTAAGCCAAAACATAGATCTTTTAAAGAATAAATTAAAAGAAAT

IRN7|AB863142.1| AAATGAATTAGCAAGGGTGAGCCAAAACATAGATCTTTTAAAGAATAAATTAAAAGAAAT

IRN8|AB863143.1| AAATGAATTAGCAAGGGTGAGCCAAAACATAGATCTTTTAAAGAATAAATTAAAAGAAAT

IRN9|AB863144.1| AAATGAATTAGCTAGGGTAAGCCAAAACATAGATCTTTTAAAAAATAAATTAAAAGAAAT

IRN10|AB863145.1| AAATGAATTAGCTAGGGTAAGCCAAAACATAGATCTTTTAAAGAATAAATTAAAAGAAAT

IRN11|AB863146.1| AAATGAATTAGCTAGGGTAAGCCAAAACATAGATCTTTTAAAGAATAAATTAAAAGAAAT

IRN12|AB863147.1| AAATGAATTAGCTAGGGTGAGCCAAAACATAGATCTTTTAAAGAATAAATTAAAAGAAAT

IRN13|AB863148.1| AAATGAATTAGCTAGGGTAAGCCAAAACATAGATCTTTTAAAAAATAAATTAAAAGAAAT

IRN14|AB863149.1| AAATGAATTAGCTAGGGTAAGCCAAAACATAGATCTTTTAAAGAATAAATTAAAAGAAAT

IRN15|AB863150.1| AAATGAATTAGCTAGGGTAAGCCAAAACATAGATCTTTTAAAGAATAAATTAAAAGAAAT

IRN16|AB863151.1| AAATGAATTAGCTAGGGTAAGCCAAAACATAGATCTTTTAAAGAATAAATTAAAAGAAAT

IRN17|AB863152.1| AAATGAATTAGCTAGGGTAAGCCAAAACATAGATCTTTTAAAGAATAAATTAAAAGAAAT

IRN18|AB863153.1| AAATGAATTAGCTAGGGTAAGCCAAAACATAGATCTTTTAAAGAATAAATTAAAAGAAAT

IRN19|AB863154.1| AAATGAATTAGCTAGGGTAAGCCAAAACATAGATCTTTTAAAGAATAAATTAAAAGAAAT

IRN20|AB863155.1| AAATGAATTAGCTAGGGTAAGCCAAAACATAGATCTTTTAAAGAATAAATTAAAAGAAAT

IRN21|AB863156.1| AAATGAATTAGCTAGGGTAAGCCAAAACATAGATCTTTTAAAGAATAAATTAAAAGAAAT

JPNHGB340|AB863157.1| AAATGAATTAGCTAGGGTAAGCCAGAACATAGATCTTTTAAAGAATAAATTAAAAGAAAT

JPNKWB778|AB863158.1| AAATGAATTAGCTAGGGTAAGCCAGAACATAGATCTTTTAAAGAATAAATTAAAAGAAAT

JPNM|AB863159.1| AAATGAATTAGTTAGGGTAAGCCAGAACATAGATCTTTTAAAGAATAAATTAAAAGAAAT

JPNN|AB863160.1| AAATGAATTAGCTAGGGTAAGCCAAAACATAGATCTTTTAAAAAATAAATTAAAAGAAAT

JPNS1|AB863161.1| AAATGAATTAGCTAGGGTAAGCCAAAACATAGATCTTTTAAAGAATAAATTAAAAGAAAT

JPNS2|AB863162.1| AAATGAATTAGCTAGGGTAAGCCAAAACATAGATCTTTTAAAGAATAAATTAAAAGAAAT

JPNTKD762|AB863163.1| AAATGAATTAGCTAGGGTAAGCCAGAACATAGATCTTTTAAAGAATAAATTAAAAGAAAT

JPNUV1|AB863164.1| AAATGAATTAGTTAGGGTAAGCCAGAACATAGATCTTTTAAAGAATAAATTAAAAGAAAT

JPNUV26|AB863165.1| AAATGAATTAGTTAGGGTAAGCCAGAACATAGATCTTTTAAAGAATAAATTAAAAGAAAT

TUR1|AB863166.1| AAATGAATTAGCTAGGGTAAGCCAAAACATAGATCTTTTAAAGAATAAATTAAAAGAAAT

TUR2|AB863167.1| AAATGAATTAGCTAGGGTAAGCCAAAACATAGATCTTTTAAAAAATAAATTAAAAGAAAT

TUR4|AB863168.1| AAATGAATTAGCTAGGGTAAGCCAAAACATAGATCTTTTAAAAAATAAATTAAAAGAAAT

TUR5|AB863169.1| AAATGAATTAGCTAGGGTAAGCCAAAACATAGATCTTTTAAAGAATAAATTAAAAGAGAT

TUR12|AB863170.1| AAATGAATTAGCTAGGGTAAGCCAAAACATAGATCTTTTAAAGAATAAATTAAAAGAAAT

TUR34|AB863171.1| AAATGAATTAGCTAGGGTAAGCCAGAACATAGATCTTTTAAAGAATAAATTAAAAGAAAT

TUR50|AB863172.1| AAATGAATTAGCTAGGGTAAGCCAAAACATAGATCTTTTAAAGAATAAATTAAAAGAAAT

TUR59|AB863173.1| AAATGAATTAGCTAGGGTAAGCCAAAACATAGATCTTTTAAAAAATAAATTAAAAGAAAT

TUR69|AB863174.1| AAATGAATTAGCTAGGGTAAGCCAAAACATAGATCTTTTAAAGAATAAATTAAAAGAAAT

TUR81|AB863175.1| AAATGAATTAGCTAGGGTAAGCCAAAACATAGATCTTTTAAAGAATAAATTAAAAGAAAT

TUR84|AB863176.1| AAATGAATTAGCTAGGGTAAGCCAAAACATAGATCTTTTAAAGAATAAATTAAAAGAAAT

TUR94|AB863177.1| AAATGAATTAGCTAGGGTAAGCCAAAACATAGATCTTTTAAAGAATAAATTAAAAGAAAT

TUR213|AB863178.1| AAATGAATTAGCTAGGGTAAGCCAAAACATAGATCTTTTAAAGAATAAATTAAAAGAAAT

TUR214|AB863179.1| AAAAGAATTAGCTAGGGTAAGCCAAAACATAGATCTTTTAAAGAATAAATTAAAAGAAAT

TUR216|AB863180.1| AAATGAATTAGCTAGGGTGAGCCAAAACATAGATCTTTTAAAGAATAAATTAAAAGAAAT

TUR220|AB863181.1| AAATGAATTAGCTAGGGTAAGCCAAAACATAGATCTTTTAAAGAATAAATTAAAAGAAAT

TUR239|AB863182.1| AAATGAATTAGCTAGGGTAAGCCAAAACATAGATCTTTTAAAGAATAAATTAAAAGAAAT

TUR244|AB863183.1| AAATGAATTAGCTAGGGTAAGCCAAAACATAGATCTTTTAAAGAATAAATTAAAAGAAAT

TUR249|AB863184.1| AAATGAATTAGCTAGGGTAAGCCAAAACATAGATCTTTTAAAGAATAAATTAAAAGAAAT

TUR263|AB863185.1| AAATGAATTAGCTAGGGTAAGCCAAAACATAGATCTTTTAAAGAATAAATTAAAAGAAAT

TUR278|AB863186.1| AAATGAATTAGCTAGGGTAAGCCAAAACATAGATCTTTTAAAGAATAAATTAAAAGAAAT

TUR279|AB863187.1| AAATGAATTAGCTAGGGTAAGCCAAAACATAGATCTTTTAAAGAATAAATTAAAAGAAAT

TUR285|AB863188.1| AAATGAATTAGCTAGGGTAAGCCAGAACATAGATCTTTTAAAGAATAAATTAAAAGAAAT

TUR289|AB863189.1| AAATGAATTAGCTAGGGTAAGCCAAAACATAGATCTTTTAAAGAATAAATTAAAAGAAAT

TUR303|AB863190.1| AAATGAATTAGCTAGGGTAAGCCAAAACATAGATCTTTTAAAGAATAAATTAAAAGAAAT

TUR306|AB863191.1| AAATGAATTAGCTAGGGTAAGCCAAAACATAGATCTTTTAAAGAATAAATTAAAAGAGAT

Cabb-S|NC_001497.1| CTGTGGAGAATA-ATATGAGCATTACGGGACAACCGCATGTTTATAAAAAAGATACTATT

CRO180A|AB863192.1| CTGTGGAGAATA-ATATGAGCATTACGGGTCAACCGCATGTTTATAAAAAAGATACTATT

GRC83|AB863193.1| CTGTGGAGA------ATGAGCATTACGGGTCAACCGCATGTTTATAAAAAAGATACTATT

GRC84B|AB863194.1| CTGTGGAGA------ATGAGCATTACGGGTCAACCGCATGTTTATAAAAAAGATACTATT

GRC86B|AB863195.1| CTGTGGAGA------ATGAGCATTACGGGTCAACCGCATGTTTATAAAAAAGATACTATT

GRC86D|AB863196.1| CTGTGGAGA------ATGAGCATTACGGGTCAACCGCATGTTTATAAAAAAGATACTATT

GRC87E|AB863197.1| CTGTGGAGA------ATGAGCATTACGGGTCAACCGCATGTTTATAAAAAAGATACTATT

GRC87G|AB863198.1| CTGTGGAGA------ATGAGCATTACGGGTCAACCGCATGTTTATAAAAAAGATACTATT

GRC91B|AB863199.1| CTGTGGAGA------ATGAGCATTACGGGTCAACCGCATGTTTATAAAAAAGATACTATT

GRC92A|AB863200.1| CTGTGGAGA------ATGAGCATTACGGGTCAACCGCATGTTTATAAAAAAGATACTATT

GRC92C|AB863201.1| CTGTGGAGA------ATGAGCATTACGGGTCAACCGCATGTTTATAAAAAAGATACTATT

GRC92D|AB863202.1| CTGTGGAGA------ATGAGCATTACGGGTCAACCGCATGTTTATAAAAAAGATACTATT

IRN1|AB863136.1| CTGTGGAGA------ATGAGCATTACGGGTCAACCGCATGTTTATAAAAAGGATACTATT

IRN2|AB863137.1| CTGTGGAGA------ATGAGCATTACGGGTCAACCGCATGTTTATAAAAAAGATACTATT

IRN3|AB863138.1| CTGTGGAGA------ATGAGCATTACGGGTCAACCGCATGTTTATAAAAAGGATACTATT

IRN4|AB863139.1| CTGTGGAGA------ATGAGCATTACGGGTCAACCGCATGTTTATAAAAAAGATACTATT

IRN5|AB863140.1| CTGTGGAGA------ATGAGCATTACGGGTCAACCGCATGTTTATAAAAAGGATACTATT

IRN6|AB863141.1| CTGTGGAGA------ATGAGCATTACGGGTCAACCGCATGTTTGTAAAAAAGATACTATT

IRN7|AB863142.1| CTGTGGAGA------ATGAGCATTACGGGTCAACCGCATGTTTATAAAAAGGATACTATT

IRN8|AB863143.1| CTGTGGAGA------ATGAGCATTACGGGTCAACCGCATGTTTATAAAAAGGATACTATT

IRN9|AB863144.1| CTGTGGAGA------ATGAGCATCACGGGTCAACCGCATGTTTATAAAAAAGATACTATT

IRN10|AB863145.1| CTGTGGAGA------ATGAGCATTACGGGTCAACCGCATGTTTATAAAAAGGATACTATT

IRN11|AB863146.1| CTGTGGAGA------ATGAGCATTACGGGTCAACCGCATGTTTATAAAAAAGATACTATT

IRN12|AB863147.1| CTGTGGAGA------ATGAGCATTACGGGTCAACCGCATGTTTATAAAAAGGATACTATT

IRN13|AB863148.1| CTGTGGAGA------ATGAGCATTACGGGTCAACCGCATGTTTATAAAAAGGATACTATT

IRN14|AB863149.1| CTGTGGAGA------ATGAGCATTACGGGTCAACCGCATGTTTATAAAAAAGATACTATT

IRN15|AB863150.1| CTGTGGAGA------ATGAGCATTACGGGTCAACCGCATGTTTATAAAAAAGATACTATT

IRN16|AB863151.1| CTGTGGAGA------ATGAGCATTACGGGTCAACCGCATGTTTATAAAAAAGATACTATT

IRN17|AB863152.1| CTGTGGAGA------ATGAGCATTACGGGTCAACCGCATGTTTATAAAAAAGATACTATT

IRN18|AB863153.1| CTGTGGAGA------ATGAGCATTACGGGTCAACCGCATGTTTATAAAAAAGATACTATT

IRN19|AB863154.1| CTGTGGAGA------ATGAGCATTACGGGTCAACCGCATGTCTATAAAAAAGATACTATT

IRN20|AB863155.1| CTGTGGAGA------ATGAGCATTACGGGTCAACCGCATGTTTATAAAAAAGATACTATT

IRN21|AB863156.1| CTGTGGAGA------ATGAGCATTACGGGTCAACCGCATGTTTATAAAAAAGATACTATT

JPNHGB340|AB863157.1| CTGTGGAGAATAAAAATGAGCATTACGGGTCAACCGCATGTTTATAAAAAAGATACTATT

JPNKWB778|AB863158.1| CTGTGGAGAATA-AAATGAGCATTACGGGTCAACCGCATGTTTATAAAAAAGATACTATT

JPNM|AB863159.1| CTGTGGAGAATA-AAATGAGCATTACGGGTCAACCGCATGTTTATAAAAAAGATACTATT

JPNN|AB863160.1| CTGTGGAGAATA-AAATGAACATTACGGGTCAACCGCATGTTTATAAAAAAGATACTATT

JPNS1|AB863161.1| CTGTGGAGAATA-AAATGAACATTACGGGTCAACCGCATGTTTATAAAAAAGATACTATT

JPNS2|AB863162.1| CTGTGGAGAATA-AAATGAACATTACGGGTCAACCGCATGTTTATAAAAAAGATACTATT

JPNTKD762|AB863163.1| CTGTGGAGAATA-AAATGAGCATTACGGGTCAACCGCATGTTTATAAAAAAGATACTATT

JPNUV1|AB863164.1| CTGTGGAGAATA-AAATGAGCATTACGGGTCAACCGCATGTTTATAAAAAAGATACTATT

JPNUV26|AB863165.1| CTGTGGAGAATA-AAATGAGCATTACGGGTCAACCGCATGTTTATAAAAAATATACTATT

TUR1|AB863166.1| CTGTGGAGA------ATGAGCATTACGGGTCAACCGCATGTTTATAAAAAAGATACTATT

TUR2|AB863167.1| CTGTGGAGA------ATGAGCATTACGGGTCAACCGCATGTTTATAAAAAAGATACTATT

TUR4|AB863168.1| CTGTGGAGA------ATGAGCATTACGGGTCAACCGCATGTTTATAAAAAAGATACTATT

TUR5|AB863169.1| CTGTGGAGA------ATGAGCATTACGGGTCAACCGCATGTTTATAAAAAAGATACTATT

TUR12|AB863170.1| CTGTGGAGA------ATGAGCATTACGGGTCAACCGCATGTTTATAAAAAAGATACTATT

TUR34|AB863171.1| CTGTGGAGA------ATGAGCATTACGGGTCAACCGCATGTTTATAAAAAAGATACTATT

TUR50|AB863172.1| CTGTGGAGA------ATGAGCATTACGGGTCAACCGCATGTTTATAAAAAAGATACTATT

TUR59|AB863173.1| CTGTGGAGA------ATGAGCATTACGGGTCAACCGCATGTTTATAAAAAAGATACTATT

TUR69|AB863174.1| CTGTGGAGA------ATGAGCATTACGGGTCAACCGCATGTTTATAAAAAAGATACTATT

TUR81|AB863175.1| CTGTGGAGA------ATGAGCATCACGGGTCAACCGCATGTTTATAAAAAAGATACTATT

TUR84|AB863176.1| CTGTGGAGA------ATGAGCATCACGGGTCAACCGCATGTTTATAAAAAAGATACTATT

TUR94|AB863177.1| CTGTGGAGA------ATGAGCATTACGGGTCAACCGCATGTTTATAAAAAAGATACTATT

TUR213|AB863178.1| CTGTGGAGA------ATGAGCATTACGGGTCAACCGCATGTTTACAAAAAGGATACTATT

TUR214|AB863179.1| CTGTGGAGA------ATGAGCATTACGGGTCAACCGCATGTTTATAAAAAAGATACTATT

TUR216|AB863180.1| CTGTGGAGA------ATGAGCATTACGGGTCAACCGCATGTTTATAAAAAAGATACTATT

TUR220|AB863181.1| CTGTGGAGA------ATGAGCATCACGGGTCAACCGCATGTTTATAAAAAAGATACTATT

TUR239|AB863182.1| CTGTGGAGA------ATGAGCATCACGGGTCAACCGCATGTTTACAAAAAAGATACTATT

TUR244|AB863183.1| CTGTGGAGA------ATGAGCATTACGGGTCAACCGCATGTTTATAAAAAAGATACTATT

TUR249|AB863184.1| CTGTGGAGA------ATGAGCATTACGGGTCAACCGCATGTTTACAAAAAGGATACTATT

TUR263|AB863185.1| CTGTGGAGA------ATGAGCATTACGGGTCAACCGCATGTTTATAAAAAAGATACTATT

TUR278|AB863186.1| CTGTGGAGA------ATGAGCATCACGGGTCAACCGCATGTTTATAAAAAAGATACTATT

TUR279|AB863187.1| CTGTGGAGA------ATGAGCATCACGGGTCAACCGCATGTTTATAAAAAAGATACTATT

TUR285|AB863188.1| CTGTGGAGA------ATGAGCATTACGGGTCAGCCGCATGTTTATAAAAAAGATACTATT

TUR289|AB863189.1| CTGTGGAGA------ATGAGCATCACGGGTCAACCGCATGTTTATAAAAAAGATACTATT

TUR303|AB863190.1| CTGTGGAGA------ATGAGCATTACGGGTCAACCGCATGTTTATAAAAAAGATACTATT

TUR306|AB863191.1| CTGTGGAGA------ATGAGCATTACGGGTCAACCGCATGTTTATAAAAAAGATACTATT

Cabb-S|NC_001497.1| ATTAGACTAAAACCATTGTCTCTTAATAGTAATAATAGAAGTTATGTTTTTAGTTCCTCA

CRO180A|AB863192.1| ATTAGACTAAAACCATTGTCTCTTAATAGTAATAATAGAAGTTATGTTTTTAGTTCCTCA

GRC83|AB863193.1| ATTAGACTAAAACCATTGTCTCTTAATAGTAATAATAGAAGTTATGTTTTTAGTTCCTCA

GRC84B|AB863194.1| ATTAGACTAAAACCATTGTCTCTTAATAGTAATAATAGAAGTTATGTTTTTAGTTCCTCA

GRC86B|AB863195.1| ATTAGACTAAAACCATTGTCTCTTAATAGTAATAATAGAAGTTATGTTTTTAGTTCCTCA

GRC86D|AB863196.1| ATTAGACTAAAACCATTGTCTCTTAATAGTAATAATAGAAGTTATGTTTTTAGTTCCTCA

GRC87E|AB863197.1| ATTAGACTAAAACCATTGTCTCTTAATAGTAATAATAGAAGTTATGTTTTTAGTTCCTCA

GRC87G|AB863198.1| ATTAGACTAAAACCATTGTCTCTTAATAGTAATAATAGAAGTTATGTTTTTAGTTCCTCA

GRC91B|AB863199.1| ATTAGACTAAAACCATTGTCTCTTAATAGTAATAATAGAAGTTATGTTTTTAGTTCCTCA

GRC92A|AB863200.1| ATTAGACTAAAACCATTGTCTCTTAATAGTAATAATAGAAGTTATGTTTTTAGTTCCTCA

GRC92C|AB863201.1| ATTAGACTAAAACCATTGTCTCTTAATAGTAATAATAGAAGTTATGTTTTTAGTTCCTCA

GRC92D|AB863202.1| ATTAGACTAAAACCATTGTCTCTTAATAGTAATAATAGAAGTTATGTTTTTAGTTCCTCA

IRN1|AB863136.1| ATTAGACTAAAACCATTGTCTCTTAATAGTAACAATAGAAGTTATGTTTTTAGTTCCTCC

IRN2|AB863137.1| ATTAGACTAAAACCATTGTCTCTTAATAGTAACAATAGAAGTTATGTTTTTAGTTCCTCC

IRN3|AB863138.1| ATTAGACTAAAACCATTGTCTCTTAATAGTAACAATAGAAGTTATGTTTTTAGTTCCTCC

IRN4|AB863139.1| ATTAGACTAAAACCACTGTCTCTTAATAGTAACAATAGAAGTTATGTTTTCAGTTCCTCA

IRN5|AB863140.1| ATTAGACTAAAACCATTGTCTCTTAATAGTAACAATAGAAGTTATGTTTTCAGTTCCTCA

IRN6|AB863141.1| ATTAGACTAAAACCATTGTCTCTTAATAGTAACAATAGAAGTTATGTTTTTAGTTCCTCC

IRN7|AB863142.1| ATTAGACTAAAACCATTGTCTCTTAATAGTAACAATAGAAGTTATGTTTTCAGTTCCTCA

IRN8|AB863143.1| ATTAGACTAAAACCATTGTCTCTTAATAGTAACAATAGAAGTTATGTTTTCAGTTCCTCA

IRN9|AB863144.1| ATCAGACTAAAACCATTGTCTCTTAATAGTAACAATAGAAGTTATGTCTTCAGTTCCTCA

IRN10|AB863145.1| ATTAGACTAAAACCATTGTCTCTTAATAGTAATAATAGAAGTTATGTTTTCAGTTCCTCA

IRN11|AB863146.1| ATTAGACTAAAACCATTGTCTCTTAATAGTAACAATAGAAGTTATGTTTTTAGTTCCTCA

IRN12|AB863147.1| ATTAGACTAAAACCATTGTCTCTTAATAGTAACAATAGAAGTTATGTTTTCAGTTCCTCA

IRN13|AB863148.1| ATTAGACTAAAACCATTGTCTCTTAATAGTAACAATAGAAGTTATGTTTTTAGTTCCTCC

IRN14|AB863149.1| ATTAGACTAAAACCATTGTCTCTTAATAGTAACAATAGAAGTTATGTTTTCAGTTCCTCA

IRN15|AB863150.1| ATTAGACTAAAACCATTGTCTCTTAATAGTAATAATAGAAGTTATGTTTTTAGTTCCTCA

IRN16|AB863151.1| ATTAGACTAAAACCATTGTCTCTTAATAGTAATAATAGAAGTTATGTTTTTAGTTCCTCA

IRN17|AB863152.1| ATTAGACTAAAACCATTGTCTCTTAATAGTAATAATAGAAGTTATGTTTTTAGTTCCTCA

IRN18|AB863153.1| ATTAGACTAAAACCATTGTCTCTTAATAGTAATAATAGAAGTTATGTTTTTAGTTCCTCA

IRN19|AB863154.1| ATTAGACTAAAACCATTGTCTCTTAATAGTAACAATAGAAGTTATGTTTTTAGTTCCTCC

IRN20|AB863155.1| ATTAGACTAAAACCATTGTCTCTTAATAGTAATAATAGAAGTTATGTTTTTAGTTCCTCA

IRN21|AB863156.1| ATTAGACTAAAACCATTGTCTCTTAATAGTAACAATAGAAGTTATGTTTTCAGTTCCTCA

JPNHGB340|AB863157.1| ATTAGACTAAAACCATTGTCTCTTAATAGTAATAATAGAACTTATGTTTTTAGTTCCTCA

JPNKWB778|AB863158.1| ATTAGACTAAAACCATTGTCTCTTAATAGTAATAATAGAACTTATGTTTTTAGTTCCTCA

JPNM|AB863159.1| ATTAGACTAAAACCATTGTCTCTTAATAGTAATAATAGAACTTATGTTTTTAGTTCCTCA

JPNN|AB863160.1| ATTAGACTAAAACCATTGTCTCTTAATAGTAATAATAGAAGTTATGTTTTTAGTTCCTCA

JPNS1|AB863161.1| ATTAGACTAAAACCATTGTCTCTTAATAGTAATAATAGAAGTTATGTTTTTAGTTCCTCA

JPNS2|AB863162.1| ATTAGACTAAAACCATTGTCTCTTAATAGTAATAATAGAAGTTATGTTTTTAGTTCCTCA

JPNTKD762|AB863163.1| ATTAGACTAAAACCATTGTCTCTTAATAGTAATAATAGAACTTATGTTTTTAGTTCCTCA

JPNUV1|AB863164.1| ATTAGACTAAAACCATTGTCTCTTAATAGTAATAATAGAACTTATGTTTTTAGTTCCTCA

JPNUV26|AB863165.1| ATTAGACTAAAACCATTGTCTCTTAATAGTAATAATAGAACTTATGTTTTTAGTTCCTCA

TUR1|AB863166.1| ATTAGACTAAAACCATTGTCTCTTAATAGTAATAATAGAAGTTATGTTTTTAGTTCCTCA

TUR2|AB863167.1| ATTAGACTAAAACCATTGTCTCTTAATAGTAATAATAGAAGTTATGTTTTTAGTTCCTCA

TUR4|AB863168.1| ATTAGACTAAAACCATTGTCTCTTAATAGTAATAATAGAAGTTATGTTTTTAGTTCCTCA

TUR5|AB863169.1| ATTAGACTAAAACCATTGTCTCTTAATAGTAATAATAGAAGTTATGTTTTTAGTTCCTCA

TUR12|AB863170.1| ATTAGACTAAAACCATTGTCTCTTAATAGTAATAATAGAAGTTATGTTTTTAGTTCCTCA

TUR34|AB863171.1| ATTAGACTAAAACCATTGTCTCTTAATAGTAACAATAGAAGTTATGTTTTTAGTTCCTCA

TUR50|AB863172.1| ATTAGACTAAAACCATTGTCTCTTAATAGTAACAATAGAAGTTATGTTTTTAGTTCCTCA

TUR59|AB863173.1| ATTAGACTAAAACCATTGTCTCTTAATAGTAATAATAGAAGTTATGTTTTTAGTTCCTCA

TUR69|AB863174.1| ATTAGACTAAAACCATTGTCTCTTAATAGTAATAATAGAAGTTATGTTTTTAGTTCCTCA

TUR81|AB863175.1| ATTAGACTAAAACCATTGTCTCTTAATAGTAATAATAGAAGTTATGTTTTTAGTTCCTCG

TUR84|AB863176.1| ATTAGACTAAAACCATTGTCTCTTAATAGTAATAATAGAAGTTATGTTTTTAGTTCCTCA

TUR94|AB863177.1| ATTAGACTAAAACCATTATCTCTTAATAGTAATAATAGAAGTTATGTTTTTAGTTCCTCA

TUR213|AB863178.1| ATTAGACTAAAACCATTGTCTCTTAATAGTAATAATAGAAGTTATGTTTTTAGTTCCTCA

TUR214|AB863179.1| ATTAGACTAAAACCATTGTCTCTTAATAGTAATAATAGAAGTTATGTTTTTAGTTCCTCA

TUR216|AB863180.1| ATTAGACTAAAAACATTGTCTCTTAATAGTAATAATAGAAGTTATGTTTTTAGTTCCTCA

TUR220|AB863181.1| ATTAGACTAAAACCATTGTCTCTTAATAGTAATAATAGAAGTTATGTTTTTAGTTCCTCA

TUR239|AB863182.1| ATTAGACTAAAACCATTGTCTCTTAATAGTAATAATAGAAGTTATGTTTTTAGTTCCTCA

TUR244|AB863183.1| ATTAGACTAAAACCATTGTCTCTTAATAGTAATAATAGAAGTTATGTTTTTAGTTCCTCA

TUR249|AB863184.1| ATTAGACTAAAACCATTGTCTCTTAATAGTAATAATAGAAGTTATGTTTTTAGTTCCTCA

TUR263|AB863185.1| ATTAGACTAAAACCATTGTCTCTTAATAGTAATAATAGAAGTTATGTTTTTAGTTCCTCC

TUR278|AB863186.1| ATTAGACTAAAACCATTGTCTCTTAATAGTAATAATAGAAGTTATGTTTTCAGTTCCTCA

TUR279|AB863187.1| ATTAGACTAAAACCATTGTCTCTTAATAGTAATAATAGAAGTTATGTTTTTAGTTCCTCA

TUR285|AB863188.1| ATTAGACTAAAACCATTGTCTCTTAATAGTAATAATAGAAGTTATGTTTTTAGTTCCTCA

TUR289|AB863189.1| ATTAGACTAAAACCATTGTCTCTCAATAGTAATAATAGAAGTTATGTTTTTAGTTCCTCA

TUR303|AB863190.1| ATTAGACTAAAACCATTGTCTCTTAATAGTAATAATAGAAGTTATGTTTTTAGTTCCTCA

TUR306|AB863191.1| ATTAGACTAAAACCATTGTCTCTTAATAGTAATAATAGAAGTTATGTTTTTAGTTCCTCA

Cabb-S|NC_001497.1| AAAGGGAACATTCAAAATATAATTAATCATCTTAACAACCTCAATGAGATTGT**AG**GAAGA

**A**

CRO180A|AB863192.1| AAAGGGAACATTCAAAATATAATTAATCATCTTAACAACCTCAATGAGATTGTAGGAAGA

GRC83|AB863193.1| AAAGGGAACATTCAAAATATAATTAACCATCTTAACAACCTCAATGAGATTGTAGGAAGA

GRC84B|AB863194.1| AAAGGGAACATTCAAAATATAATTAATCATCTTAACAACCTCAATGAGATTGTAGGAAGA

GRC86B|AB863195.1| AAAGGGAACATTCAAAATATAATTAATCATCTTAACAACCTCAATGAGATTGTAGGAAGA

GRC86D|AB863196.1| AAAGGGAATATTCAAAATATAATTAACCATCTTAACAACCTCAATGAGATTGTAGGAAGA

GRC87E|AB863197.1| AAAGGGAACATTCAAAATATAATTAATCATCTTAACAACCTCAATGAGATTGTAGGAAGA

GRC87G|AB863198.1| AAAGGGAACATTCAAAATATAATTAACCATCTTAACAACCTCAATGAGATTGTAGGAAGA

GRC91B|AB863199.1| AAAGGGAACATTCAAAATATAATTAATCATCTTAACAACCTCAATGAGATTGTAGGAAGA

GRC92A|AB863200.1| AAAGGGAACATTCAAAATATAATTAATCATCTTAACAACCTCAATGAGATTGTAGGAAGA

GRC92C|AB863201.1| AAAGGGAACATTCAAAATATAATTAATCATCTTAACAACCTCAATGAGATTGTAGGAAGA

GRC92D|AB863202.1| AAAGGGAACATTCAAAATATAATTAATCATCTTAACAACCTCAATGAGATTGTAGGAAGA

IRN1|AB863136.1| AAAGGGAATATTCAAAATATAATTAATCATCTTAACAACCTCAATGAGATTGTAGGAAGA

IRN2|AB863137.1| AAAGGGAACATTCAAAATATAATTAATCATCTTAACAACCTCAATGAGATTGTAGGAAGA

IRN3|AB863138.1| AAAGGGAATATTCAAAATATAATTAATCATCTTAACAACCTCAATGAGATTGTAGGAAGA

IRN4|AB863139.1| AAAGGGAACATTCAAAATATAATTAATCATCTTAACAACCTCAATGAGATTGTAGGAAGA

IRN5|AB863140.1| AAAGGGAACATTCAAAATATAATTAATCATCTTAACAACCTCAATGAGATTGTAGGAAGA

IRN6|AB863141.1| AAAGGGAACATTCAAAATATAATTAATCATCTTAACAACCTCAATGAGATTGTAGGAAGA

IRN7|AB863142.1| AAAGGGAACATTCAAAATATAATTAATCATCTTAACAACCTCAATGAGATTGTAGGAAGA

IRN8|AB863143.1| AAAGGGAACATTCAAAATATAATTAATCATCTTAACAACCTCAATGAGATTGTAGGAAGA

IRN9|AB863144.1| AAAGGGAACATTCAAAATATAATTAATCATCTTAACAACCTCAATGAGATTGTAGGAAGA

IRN10|AB863145.1| AAAGGGAACATTCAAAATATAATTAATCATCTTAACAACCTCAATGAGATTGTAGGAAGA

IRN11|AB863146.1| AAAGGGAACATTCAAAATATAATTAATCATCTTAACAACCTCAATGAGATTGTAGGAAGA

IRN12|AB863147.1| AAAGGGAACATTCAAAATATAATTAATCATCTTAACAACCTCAATGAGATTGTAGGAAGA

IRN13|AB863148.1| AAAGGGAACATTCAAAATATAATTAATCATCTTAACAACCTCAATGAGATTGTAGGAAGA

IRN14|AB863149.1| AAAGGGAACATTCAAAATATAATTAATCATCTTAACAACCTCAATGAGATTGTAGGAAGA

IRN15|AB863150.1| AAAGGGAACATTCAAAATATAATTAATCATCTTAACAACCTCAATGAGATTGTAGGAAGA

IRN16|AB863151.1| AAAGGGAACATTCAAAATATAATTAATCATCTTAACAACCTCAATGAGATTGTAGGAAGA

IRN17|AB863152.1| AAAGGGAACATTCAAAATATAATTAATCATCTTAACAACCTCAATGAGATTGTAGGAAGA

IRN18|AB863153.1| AAAGGGAACATTCAAAATATAATTAATCATCTTAACAACCTCAATGAGATTGTAGGAAGA

IRN19|AB863154.1| AAAGGGAATATTCAAAATATAATTAATCATCTTAACAACCTCAATGAGATTGTAGGAAGA

IRN20|AB863155.1| AAAGGGAACATTCAAAATATAATTAATCATCTTAACAACCTCAATGAGATTGTAGGAAGA

IRN21|AB863156.1| AAAGGGAACATTCAAAATATAATTAATCATCTTAACAACCTCAATGAGATTGTAGGAAGA

JPNHGB340|AB863157.1| AAAGGGAATATTCAAAATATAATTAATCATCTTAACAACCTCAATGAGATTGTAGGAAGA

JPNKWB778|AB863158.1| AAAGGGAATATTCAAAATATAATTAATCATCTTAACAACCTCAATGAGATTGTAGGAAGA

JPNM|AB863159.1| AAAGGGAATATTCAAAATATAATTAATCATCTTAACAACCTCAATGAGATTGTAGGAAGA

JPNN|AB863160.1| AAAGGAAATATTCAAAATATAACTAATCATCTTAACAACCTCAATGAGATTGTAGGAAGA

JPNS1|AB863161.1| AAAGGGAATATTCAAAATATAATTAACCATCTTAACAACCTCAATGAGATTGTAGGAAGA

JPNS2|AB863162.1| AAAGGGAATATTCAAAATATAATTAACCATCTTAACAACCTCAATGAGATTGTAGGAAGA

JPNTKD762|AB863163.1| AAAGGGAATATTCAAAATATAATTAATCATCTTAACAACCTCAATGAGATTGTAGGAAGA

JPNUV1|AB863164.1| AAAGGGAATATTCAAAATATAATTAATCATCTTAACAACCTCAATGAGATTGTAGGAAGA

JPNUV26|AB863165.1| AAAGGGAATATTCAAAATATAATTAATCATCTTAACAACCTCAATGAGATTGTAGGAAGA

TUR1|AB863166.1| AAAGGGAACATTCAAAATATAATTAATCATCTTAACAACCTCAATGAGATTGTAGGAAGA

TUR2|AB863167.1| AAAGGGAACATTCAAAATATAATTAATCATCTTAACAACCTCAATGAGATTGTAGGAAGA

TUR4|AB863168.1| AAAGGGAACATTCAAAATATAATTAATCATCTTAACAACCTCAATGAGATTGTAGGAAGA

TUR5|AB863169.1| AAAGGGAACATTCAAAATATAATTAATCATCTTAACAACCTCAATGAGATTGTAGGAAGA

TUR12|AB863170.1| AAAGGGAACATTCAAAATATAATTAATCATCTTAACAACCTCAATGAGATTGTAGGAAGA

TUR34|AB863171.1| AAAGGGAATATTCAAAATATAATTAATCATCTTAACAACCTCAATGAGATTGTAGGAAGA

TUR50|AB863172.1| AAAGGGAATATTCAAAATATAATTAATCATCTTAACAACCTCAATGAGATTGTAGGAAGA

TUR59|AB863173.1| AAAGGGAACATTCAAAATATAATTAATCATCTTAACAACCTCAATGAGATTGTAGGAAGA

TUR69|AB863174.1| AAAGGGAACATTCAAAATATAATTAATCATCTTAACAACCTCAATGAGATTGTAGGAAGA

TUR81|AB863175.1| AAAGGGAACATTCAAAATATAATTAATCATCTTAACAACCTCAATGAGATTGTAGGAAGA

TUR84|AB863176.1| AAAGGGAACATTCAAAATATAATTAATCATCTTAACAACCTCAATGAGATTGTAGGAAGA

TUR94|AB863177.1| AAAGGGAACATTCAAAATATAATTAATCATCTTAACAACCTCAATGAGATTGTAGGAAGA

TUR213|AB863178.1| AAAGGGAACATTCAAAATATAATTAATCATCTTAACAACCTCAATGAGATTGTAGGAAGA

TUR214|AB863179.1| AAAGGGAACATTCAAAATATAATTAATCATCTTAACAACCTCAATGAGATTGTAGGAAGA

TUR216|AB863180.1| AAAGGGAACATTCAAAATATAATTAATCATCTTAACAACCTCAATGAGATTGTAGGAAGA

TUR220|AB863181.1| AAAGGGAACATTCAAAATATAATTAATCATCTTAACAACCTCAATGAGATTGTAGGAAGA

TUR239|AB863182.1| AAAGGGAACATTCAAAATATAATTAATCATCTTAACAACCTCAATGAGATTGTAGGAAGA

TUR244|AB863183.1| AAAGGGAACATCCAAAATATAATTAATCATCTTAACAACCTCAATGAGATTGTAGGAAGA

TUR249|AB863184.1| AAAGGGAACATTCAAAATATAATTAATCATCTTAACAACCTCAATGAGATTGTAGGAAGA

TUR263|AB863185.1| AAAGGGAACATTCAAAATATAATTAATCATCTTAACAACCTCAATGAGATTGTAGGAAGA

TUR278|AB863186.1| AAAGGGAACATTCAAAATATAATTAATCATCTTAACAACCTCAATGAGATTGTAGGAAGA

TUR279|AB863187.1| AAAGGGAACATTCAAAATATAATTAATCATCTTAACAACCTCAATGAGATTGTAGGAAGA

TUR285|AB863188.1| AAAGGGAACATTCAAAATATAATTAATCATCTTAACAACCTCAATGAGATTGTAGGAAGA

TUR289|AB863189.1| AAAGGGAACATTCAAAATATAATTAATCATCTTAACAACCTCAATGAGATTGTAGGTAGA

TUR303|AB863190.1| AAAGGGAACATTCAAAATATAATTAATCATCTTAACAACCTCAATGAGATTGTAGGAAGA

TUR306|AB863191.1| AAAGGGAACATTCAAAATATAATTAATCATCTTAACAACCTCAATGAGATTGTAGGAAGA

Cabb-S|NC_001497.1| AGCTTACTCGGAATATGGAAGATCAACTCATACTTCGGATTAAGCAAAGACCCTTCGGAG

CRO180A|AB863192.1| AGCTTGCTCGGAATATGGAAGATCAACTCATACTTCGGCTTAAGCAAAGACCCTTCGGAG

GRC83|AB863193.1| AGCTTACTCGGAATATGGAAGATCAACTCATACTTCGGACTAAGCAAAGACCCTTCGGAG

GRC84B|AB863194.1| AGCTTACTCGGAATATGGAAGATCAACTCATACTTCGGACTAAGCAAAGACCCTTCGGAG

GRC86B|AB863195.1| AGCTTACTCGGAATATGGAAGATCAACTCATACTTCGGACTAAGCAAAGACCCTTCGGAG

GRC86D|AB863196.1| AGCTTACTCGGAATATGGAAGATCAACTCATACTTCGGACTAAGCAAAGACCCTTCGGAG

GRC87E|AB863197.1| AGCTTACTCGGAATATGGAAGATCAACTCATACTTCGGACTAAGCAAAGACCCTTCGGAG

GRC87G|AB863198.1| AGCTTACTCGGAATATGGAAGATCAACTCATACTTCGGACTAAGCAAAGACCCTTCGGAG

GRC91B|AB863199.1| AGCTTACTCGGAATATGGAAGATCAACTCATACTTCGGACTAAGCAAAGACCCTTCGGAG

GRC92A|AB863200.1| AGCTTACTCGGAATATGGAAGATCAACTCATACTTCGGACTAAGCAAAGACCCTTCGGAG

GRC92C|AB863201.1| AGCTTACTCGGAATATGGAAGATCAACTCATACTTCGGACTAAGCAAAGACCCTTCGGAG

GRC92D|AB863202.1| AGCTTACTCGGAATATGGAAGATCAACTCATACTTCGGACTAAGCCAAGACCCTTCGGAG

IRN1|AB863136.1| AGCTTGCTCGGAATATGGAAGATCAACTCATACTTCGGCTTAAGCAAAGACCCTTCGGAG

IRN2|AB863137.1| AGCTTACTCGGAATATGGAAGATCAACTCATACTTCGGCTTAAGCAAGGACCCTTCGGAG

IRN3|AB863138.1| AGCTTGCTCGGAATATGGAAGATCAACTCATACTTCGGCTTAAGCAAAGACCCTTCGGAG

IRN4|AB863139.1| AGCTTACTCGGAATATGGAAGATCAACTCATACTTCGGACTAAGCAAAGACCCTTCGGAG

IRN5|AB863140.1| AGCTTACTCGGAATATGGAAGATCAACTCATACTTCGGACTAAGCAAAGACCCTTCGGAG

IRN6|AB863141.1| AGCTTACTCGGAATATGGAAGATCAACTCATACTTCGGACTAAGCAAAGACCCTTCGGAG

IRN7|AB863142.1| AGCTTACTCGGAATATGGAAGATCAACTCATACTTCGGACTAAGCAAAGACCCTTCGGAG

IRN8|AB863143.1| AGCTTACTCGGAATATGGAAGATCAACTCATACTTCGGACTAAGCAAAGACCCTTCGGAG

IRN9|AB863144.1| AGCTTACTCGGAATATGGAAGATCAACTCATACTTCGGACTAAGCAAAGACCCTTCGGAG

IRN10|AB863145.1| AGCTTGCTCGGGATATGGAAGATCAACTCATACTTCGGACTAAGCAAAGACCCTTCGGAG

IRN11|AB863146.1| AGCTTACTCGGAATATGGAAGATCAACTCATACTTCGGACTAAGCAAAGACCCTTCGGAG

IRN12|AB863147.1| AGCTTACTCGGAATATGGAAGATCAACTCATACTTCGGACTAAGCAAAGACCCTTCGGAG

IRN13|AB863148.1| AGCTTACTCGGAATATGGAAGATCAACTCATACTTCGGACTAAGCAAAGACCCTTCGGAG

IRN14|AB863149.1| AGCTTACTCGGAATATGGAAGATCAACTCATACTTCGGACTAAGCAAAGACCCTTCGGAG

IRN15|AB863150.1| AGCTTACTCGGAATATGGAAGATCAACTCATACTTCGGACTAAGCAAAGACCCTTCGGAG

IRN16|AB863151.1| AGCTTACTCGGAATATGGAAGATCAACTCATACTTCGGACTAAGCAAAAACCCTTCGGAG

IRN17|AB863152.1| AGCTTACTCGGAATATGGAAGATCAACTCATACTTCGGACTAAGCAAAGACCCTTCGGAG

IRN18|AB863153.1| AGCTTACTCGGAATATGGAAGATCAACTCATACTTCGGACTAAGCAAAGACCCTTCAGAG

IRN19|AB863154.1| AGCTTGCTCGGAATATGGAAGATCAACTCATACTTCGGCTTAAGCAAGGACCCTTCGGAG

IRN20|AB863155.1| AGCTTACTCGGAATATGGAAGATCAACTCATACTTCGGACTAAGCAAAGACCCTTCGGAG

IRN21|AB863156.1| AGCTTACTCGGAATATGGAAGATCAACTCATACTTCGGACTAAGCAAAGACCCTTCGGAG

JPNHGB340|AB863157.1| AGCTTACTCGGAATATGGAAGATCAACTCATACTTCGGCTTAAGCAAAGACCCTTCGGAG

JPNKWB778|AB863158.1| AGCTTACTCGGAATATGGAAGATCAACTCATACTTCGGCTTAAGCAAAGACCCTTCGGAG

JPNM|AB863159.1| AGCTTACTCGGAATATGGAAGATCAACTCATACTTCGGCTTAAGCAAAGACCCTTCGGAG

JPNN|AB863160.1| AGCTTGCTCGGAATATGGAAGATCAACTCATACTTCGGCTTAAGCAAAGACCCTTCGGAG

JPNS1|AB863161.1| AGCTTGCTCGGAATATGGAAGATCAACTCATACTTCGGCTTAAGCAAAGACCCTTCGGAG

JPNS2|AB863162.1| AGCTTGCTCGGAATATGGAAGATCAACTCATACTTCGGCTTAAGCAAAGACCCTTCGGAG

JPNTKD762|AB863163.1| AGCTTACTCGGAATATGGAAGATCAACTCATACTTCGGCTTAAGCAAAGACCCTTCGGAG

JPNUV1|AB863164.1| AGCTTACTCGGAATATGGAAGATCAACTCATACTTCGGCTTAAGCAAAGACCCTTCGGAG

JPNUV26|AB863165.1| AGCTTACTCGGAATATGGAAGATCAACTCATACTTCGGCTTAAGCAAAGACCCTTCGGAG

TUR1|AB863166.1| AGCTTACTCGGAATATGGAAGATCAACTCATACTTCGGACTAAGCAAAGACCCTTCGGAG

TUR2|AB863167.1| AGCTTGCTCGGAATATGGAAGATCAACTCATACTTCGGCTTAAGCAAAGACCCTTCGGAG

TUR4|AB863168.1| AGCTTGCTCGGAATATGGAAGATCAACTCATACTTCGGCTTAAGCAAAGACCCTTCGGAG

TUR5|AB863169.1| AGCTTACTCGGAATATGGAAGATCAACTCATACTTCGGACTAAGCAAAGACCCTTCGGAG

TUR12|AB863170.1| AGCTTACTCGGAATATGGAAGATCAACTCATACTTCGGACTAAGCAAAGACCCTTCGGAG

TUR34|AB863171.1| AGCTTGCTCGGAATATGGAAGATCAACTCATACTTCGGCTTAAGCAAAGACCCTTCGGAG

TUR50|AB863172.1| AGCTTGCTCGGAATATGGAAGATCAACTCATACTTCGGCTTAAGCAAAGACCCTTCGGAG

TUR59|AB863173.1| AGCTTGCTCGGAATATGGAAGATCAACTCATACTTCGGCTTAAGCAAAGACCCTTCGGAG

TUR69|AB863174.1| AGCTTACTCGGAATATGGAAGATCAACTCATACTTCGGACTAAGCAAAGACCCTTCGGAG

TUR81|AB863175.1| AGCTTACTCGGAATATGGAAGATCAACTCATACTTCGGACTAAGCAAAGACCCTTCGGAG

TUR84|AB863176.1| AGCTTACTCGGAATATGGAAGATCAACTCATACTTCGGACTAAGCAAAGACCCTTCGGAG

TUR94|AB863177.1| AGCTTACTCGGAATATGGAAGATCAACTCATACTTCGGACTAAGCAAAGACCCTTCGGAG

TUR213|AB863178.1| AGCTTACTCGGAATATGGAAGATCAACTCATACTTCGGACTAAGCAAAGACCCTTCGGAG

TUR214|AB863179.1| AGCTTACTCGGAATATGGAAGATCAACTCATACTTCGGACTAAGCAAAGACCCTTCGGAG

TUR216|AB863180.1| AGCTTACTCGGAATATGGAAGATCAACTCATACTTCGGACTAAGCAAAGACCCTTCGGAG

TUR220|AB863181.1| AGCTTACTCGGAATATGGAAGATCAACTCATACTTCGGACTAAGCAAAGACCCTTCGGAG

TUR239|AB863182.1| AGCTTACTCGGAATATGGAAGATCAACTCATACTTCGGACTAAGCAAAGACCCTTCGGAG

TUR244|AB863183.1| AGCTTACTCGGAATATGGAAGATCAACTCATACTTCGGACTAAGCAAAGACCCTTCGGAG

TUR249|AB863184.1| AGCTTACTCGGAATATGGAAGATCAACTCATACTTCGGACTAAGCAAAGACCCTTCGGAG

TUR263|AB863185.1| AGCTTACTCGGAATATGGAAGATCAACTCATACTTCGGCTTAAGCAAAGACCCTTCAGAG

TUR278|AB863186.1| AGCTTACTCGGAATATGGAAGATCAACTCATACTTCGGACTAAGCAAAGACCCTTCGGAG

TUR279|AB863187.1| AGCTTACTCGGAATATGGAAGATCAACTCATACTTCGGACTAAGCAAAGACCCTTCGGAG

TUR285|AB863188.1| AGCTTACTCGGAATATGGAAGATCAACTCATACTTCGGACTAAGCAAAGACCCTTCGGAG

TUR289|AB863189.1| AGCTTACTCGGAATATGGAAGATCAACTCATACTTCGGACTAAGCAAAGACCCTTCGGAG

TUR303|AB863190.1| AGCTTACTCGGAATATGGAAGATCAACTCATACTTCGGACTAAGCAAAGACCCTTCGGAG

TUR306|AB863191.1| AGCTTACTCGGAATATGGAAGATCAACTCATACTTCGGACTAAGCAAAGACCCTTCGGAG

Cabb-S|NC_001497.1| TCCAAATCAAAAAACCCGTCAGTTTTTAATACTGCAAAAACCATTTTTAAGAGTGGGGGG

CRO180A|AB863192.1| TCCAAATCAAAAAACCCGTCAGTTTTTAATACTGCAAAAACCATTTTTAAGAGTGGGGGG

GRC83|AB863193.1| TCCAAATCAAAAAACCCGTCAGTTTTTAATACTGCAAAAACCATTTTTAAGAGTGGGGGG

GRC84B|AB863194.1| TCCAAATCAAAAAACCCGTCAGTTTTCAATACTGCAAAAACCATTTTTAAGAGTGGGGGG

GRC86B|AB863195.1| TCCAAATCAAAAAACCCGTCAGTTTTTAATACTGCAAAAACCATTTTTAAGAGTGGGGGG

GRC86D|AB863196.1| TCCAAATCAAAAAACCCGTCAGTTTTTAATACTGCAAAAACCATTTTTAAGAGTGGGGGG

GRC87E|AB863197.1| TCCAAATCAAAAAACCCGTCAGTTTTTAATACTGCAAAAACCATTTTTAAGAGTGGGGGG

GRC87G|AB863198.1| TCCAAATCAAAAAACCCGTCAGTTTTTAATACTGCAAAAACCATTTTTAAGAGTGGGGGG

GRC91B|AB863199.1| TCCAAATCAAAAAACCCGTCAGTTTTTAATACTGCAAAAACCATTTTTAAGAGTGGGGGG

GRC92A|AB863200.1| TCCAAATCAAAAAACCCGTCAGTTTTCAATACTGCAAAAACCATTTTTAAGAGTGGGGGG

GRC92C|AB863201.1| TCCAAATCAAAAAACCCGTCAGTTTTCAATACTGCAAAAACCATTTTTAAGAGTGGGGGG

GRC92D|AB863202.1| TCCAAATCAAAAAACCCGTCAGTTTTCAATACTGCAAAAACCATTTTTAAGAATGGGGGG

IRN1|AB863136.1| TCCAAATCAAAAAACCCGTCAGTTTTTAATACTGCAAAAACCATTTTTAAGAGTGGGGGG

IRN2|AB863137.1| TCGAAATCAAAAAACCCGTCAGTTTTTAATACTGCAAAAACCATTTTTAAGAGTGGGGGG

IRN3|AB863138.1| TCCAAATCAAAAAACCCGTCAGTTTTTAATACTGCAAAAACCATTTTTAAGAGTGGGGGG

IRN4|AB863139.1| TCCAAATCAAAAAACCCGTCAGTTTTTAATACTGCAAAAACCATTTTTAAGAGTGGGGGG

IRN5|AB863140.1| TCCAAATCAAAAAACCCGTCAGTTTTTAATACTGCAAAAACCATTTTTAAGAGTGGGGGG

IRN6|AB863141.1| TCCAAATCAAAAAACCCGTCAGTTTTTAATACTGCAAAAACCATTTTTAAGAGTGGGGGG

IRN7|AB863142.1| TCCAAATCAAAAAACCCGTCAGTTTTCAATACTGCAAAAACCATTTTTAAGAATGGGGGG

IRN8|AB863143.1| TCCAAATCAAAAAACCCGTCAGTTTTTAATACTGCAAAAACCATTTTTAAGAATGGGGGG

IRN9|AB863144.1| TCCAAATCAAAAAACCCGTCAGTTTTCAATACTGCAAAAACCATTTTTAAGAGTGGGGGG

IRN10|AB863145.1| TCCAAATCAAAAAACCCGTCAGTTTTTAATACTGCAAAAACCATTTTTAAGAGTGGGGGG

IRN11|AB863146.1| TCCAAATCAAAAAACCCGTCAGTTTTTAATACTGCAAAAACCATTTTTAAGAGTGGGGGG

IRN12|AB863147.1| TCCAAATCAAAAAACCCGTCAGTTTTTAATACTGCAAAAACCATTTTTAAGAGTGGGGGG

IRN13|AB863148.1| TCCAAATCAAAAAACCCGTCAGTTTTTAATACTGCAAAAACCATTTTTAAGAGTGGAGGG

IRN14|AB863149.1| TCCAAATCAAAAAACCCGTCAGTTTTTAATACTGCAAAAACCATTTTTAAGAGTGGGGGG

IRN15|AB863150.1| TCCAAATCAAAAAACCCGTCAGTTTTTAATACTGCAAAAACCATTTTTAAGAGTGGGGGG

IRN16|AB863151.1| TCCAAATCAAAAAACCCGTCAGTTTTTAATACTGCAAAAACCATTTTTAAGAGTGGGGGG

IRN17|AB863152.1| TCCAAATCAAAAAACCCGTCAGTTTTTAATACTGCAAAAACCATTTTTAAGAGTGGGGGG

IRN18|AB863153.1| TCCAAATCGAAAAACCCGTCAGTTTTTAATACTGCAAAAACCATTTTTAAGAGTGGGGGG

IRN19|AB863154.1| TCGAAATCAAAAAACCCGTCAGTTTTTAATACTGCAAAAACCATTTTTAAGAGTGGGGGG

IRN20|AB863155.1| TCCAAATCAAAAAACCCGTCAGTTTTTAATACTGCAAAAACCATTTTTAAGAGTGGGGGG

IRN21|AB863156.1| TCCAAATCAAAAAACCCGTCAGTTTTCAATACTGCAAAAACCATTTTTAAGAGTGGGGGG

JPNHGB340|AB863157.1| TCCAAATCAAAAAACCCGTCAGTTTTTAATACTGCAAAAACCATTTTTAAGAGTGGGGGG

JPNKWB778|AB863158.1| TCCAAATCAAAAAACCCGTCAGTTTTTAATACTGCAAAAACCATTTTTAAGAGTGGGGGG

JPNM|AB863159.1| TCCAAATCAAAAAACCCGTCAGTTTTTAATACTGCAAAAACCATTTTTAAGAGTGGGGGG

JPNN|AB863160.1| TCCAAATCAAAAAACCCGTCAGTTTTTAATACTGCAAAAACCATTTTTAAGAGTGGGGGG

JPNS1|AB863161.1| TCCAAATCAAAAAACCCGTCAGTTTTTAATACTGCAAAAACCATTTTTAAGAGTGGGGGG

JPNS2|AB863162.1| TCCAAATCAAAAAACCCGTCAGTTTTTAATACTGCAAAAACCATTTTTAAGAGTGGGGGG

JPNTKD762|AB863163.1| TCCAAATCAAAAAACCCGTCAGTTTTTAATACTGCAAAAACCATTTTTAAGAGTGGGGGG

JPNUV1|AB863164.1| TCCAAATCAAAAAACCCGTCAGTTTTTAATACTGCAAAAACCATTTTTAAGAGTGGGGGG

JPNUV26|AB863165.1| TTCAAATCAAAAAACCCGTCAGTTTTTAATACTGCAAAAACCATTTTTAAGAGTGGGGGG

TUR1|AB863166.1| TCCAAATCAAAAAACCCGTCAGTTTTTAATACTGCAAAAACCATTTTTAAGAGTGGGGGG

TUR2|AB863167.1| TCCAAATCCAAAAACCCGTCAGTTTTTAATACTGCAAAAACCATTTTTAAGAGTGGGGGG

TUR4|AB863168.1| TCCAAATCCAAAAACCCGTCAGTTTTTAATACTGCAAAAACCATTTTTAAGAGTGGGGGG

TUR5|AB863169.1| TCCAAATCAAAAAACCCGTCAGTTTTTAATACTGCAAAAACCATTTTTAAGAGTGGGGGG

TUR12|AB863170.1| TCCAAATCAAAAAACCCGTCAGTTTTTAATACTGCAAAAACCATTTTTAAGAGTGGGGGG

TUR34|AB863171.1| TCCAAATCAAAAAACCCGTCAGTTTTTAATACTGCAAAAACCATTTTTAAGAGTGGGGGG

TUR50|AB863172.1| TCCAAATCAAAAAACCCGTCAGTTTTTAATACTGCAAAAACCATTTTTAAGAGTGGGGGG

TUR59|AB863173.1| TCCAAATCGAAAAACCCGTCAGTTTTTAATACTGCAAAAACCATTTATAAGAGTGGGGGG

TUR69|AB863174.1| TCCAAATCAAAAAACCCGTCAGTTTTTAATACTGCAAAAACCATTTTTAAGAGTGGGGGG

TUR81|AB863175.1| TCCAAATCAAAAAACCCGTCAGTTTTTAATACTGCAAAAACCATTTTTAAGAGTGGGGGG

TUR84|AB863176.1| TCCAAATCAAAAAACCCGTCAGTTTTTAATACTGCAAAAACCATTTTTAAGAGTGGGGGG

TUR94|AB863177.1| TCCAAATCGAAAAACCCGTCAGTTTTTAATACTGCAAAAACCATTTTTAAGAGTGGGGGG

TUR213|AB863178.1| TCCAAATCCAAAAACCCGTCAGTTTTTAATACTGCAAAAACCATTTTTAAGAGTGGGGGG

TUR214|AB863179.1| TCCAAATCAAAAAACCCGTCAGTTTTTAATACTGCAAAAACCATTTTTAAGAGTGGGGGG

TUR216|AB863180.1| TCCAAATCGAAAAACCCGTCAGTTTTTAATACTGCAAAAACCATTTTTAAGAGTGGGGGG

TUR220|AB863181.1| TCCAAATCAAAAAACCCGTCAGTTTTTAATACTGCAAAAACCATTTTTAAGAGTGGGGGG

TUR239|AB863182.1| TCCAAATCCAAAAACCCGTCAGTTTTTAATACTGCAAAAACCATTTTTAAGAGTGGGGGG

TUR244|AB863183.1| TCCAAATCAAAAAACCCGTCAGTTTTTAATACTGCAAAAACCATTTTTAAGAGTGGGGGG

TUR249|AB863184.1| TCCAAATCCAAAAACCCGTCAGTTTTTAATACTGCAAAAACCATTTTTAAGAGTGGGGGG

TUR263|AB863185.1| TCCAAATCGAAAAACCCGTCAGTTTTTAATACTGCAAAAACCATTTTTAAGAGTGGGGGG

TUR278|AB863186.1| TCCAAATCAAAAAACCCGTCAGTTTTTAATACTGCAAAAACCATTTTTAAGAGTGGGGGG

TUR279|AB863187.1| TCCAAATCAAAAAACCCGTCAGTTTTTAATACTGCAAAAACCATTTTTAAGAGTGGGGGG

TUR285|AB863188.1| TCCAAATCAAAAAACCCGTCAGTTTTTAATACTGCAAAAACCATTTTTAAGAGTGGGGGG

TUR289|AB863189.1| TCCAAATCAAAAAACCCGTCAGTTTTTAATACTGCAAAAACCATTTTTAAGAGTGGGGGG

TUR303|AB863190.1| TCCAAATCAAAAAACCCGTCAGTTTTTAATACTGCAAAAACCATTTTTAAGAGTGGGGGG

TUR306|AB863191.1| TCCAAATCCAAAAACCCGTCAGTTTTTAATACTGCAAAAACCATTTTTAAGAGTGGGGGG

Cabb-S|NC_001497.1| GTTGATTACTCGAGCCAACTAAAGGAAATAAAATCCCTTTTAGAAGCTCAAAACACTAGA

CRO180A|AB863192.1| GTTGATTACTCGAGCCAACTAAAGGAAATAAAATCCCTTTTAGAAGCTCAAAATACTAGA

GRC83|AB863193.1| GTTGATTACTCGAGCCAACTAAAAGAAATAAAATCCCTTTTAGAAGCTCAGAATACTAGA

GRC84B|AB863194.1| GTTGATTACTCGAGCCAACTAAAGGAAATAAAATCCCTTTTAGAAGCTCAAAATACTAGA

GRC86B|AB863195.1| GTTGATTACTCGAGCCAACTAAAAGAAATAAAATCCCTTTTGGAAGCTCAAAATACTAGA

GRC86D|AB863196.1| GTTGATTACTCGAGCCAACTAAAGGAAATAAAATCCCTTTTGGAAGCTCAAAATACTAGA

GRC87E|AB863197.1| GTTGATTACTCGAGCCAACTAAAAGAAATAAAATCCCTTTTAGAAGCTCAGAATACTAGA

GRC87G|AB863198.1| GTTGATTACTCGAGCCAACTAAAAGAAATAAAATCCCTTTTAGAAGCTCAGAATACTAGA

GRC91B|AB863199.1| GTTGATTACTCGAGCCAACTAAAGGAAATAAAATCCCTTTTAGAAGCTCAAAATACTAGA

GRC92A|AB863200.1| GTTGATTACTCGAGCCAACTAAAGGAAATAAAATCCCTTTTAGAAGCTCAAAATACTAGA

GRC92C|AB863201.1| GTTGATTACTCGAGCCAACTAAAGGAAATAAAATCCCTTTTAGAAGCTCAAAATACTAGA

GRC92D|AB863202.1| GTTGATTACTCGAGCCAACTAAAGGAAATAAAATCCCTTTTAGAAGCTCAAAATACTAGA

IRN1|AB863136.1| GTTGATTACTCGAGTCAACTAAAAGAAATAAAATCCCTTTTAGAAGCTCAAAATACTAGA

IRN2|AB863137.1| GTTGATTACTCGAGTCAACTAAAAGAAATAAAATCCCTTTTAGAAGCTCAAAATACTAGA

IRN3|AB863138.1| GTTGATTACTCGAGTCAACTAAAAGAAATAAAATCCCTTTTAGAAGCTCAAAATACTAGA

IRN4|AB863139.1| GTTGATTACTCGAGCCAACTAAAGGAAATAAAATCCCTTTTAGAAGCTCAAAATACTAGA

IRN5|AB863140.1| GTTGATTACTCGAGTCAACTAAAAGAAATAAAATCCCTTTTAGAAGCTCAAAGTACTAGA

IRN6|AB863141.1| GTTGATTACTCGAGCCAACTAAAAGAAATAAAATCCCTTTTAGAAGCTCAAAATACTAGA

IRN7|AB863142.1| GTTGATTACTCGAGCCAACTAAAGGAAATAAAATCCCTTTTAGAAGCTCAAAATACTAGA

IRN8|AB863143.1| GTTGATTACTCGAGTCAACTAAAAGAAATAAAATCCCTTTTAGAAGCTCAAAATACTAGA

IRN9|AB863144.1| GTTGATTACTCGAGCCAACTA---------AAATCCCTTTTAGAAGCTCAAAATACTAGA

IRN10|AB863145.1| GTTGATTACTCGAGCCAACTAAAGGAAATAAAATCCCTTTTAGAAGCTCAAAATACTAGA

IRN11|AB863146.1| GTTGATTACTCGAGCCAACTAAAGGAAATAAAATCCCTTTTAGAAGCTCAAAATACTAGA

IRN12|AB863147.1| GTTGATTACTCGAGCCAACTAAAAGAAATAAAATCCCTTTTAGAAGCTCAAAATACTAGA

IRN13|AB863148.1| GTTGATTACTCGAGTCAACTAAAAGAAATAAAATCCCTTTTAGAAGCTCAAAATACTAGA

IRN14|AB863149.1| GTTGATTACTCGAGCCAACTAAAAGAAATAAAATCCCTTTTAGAAGCTCAAAATACTAGA

IRN15|AB863150.1| GTTGATCACTCGAGCCAACTAAAAGAAATAAAATCCCTTTTAGAAGCTCAAAATACTAGA

IRN16|AB863151.1| GTTGATTACTCGAGCCAACTAAAAGAAATAAAATCCCTTTTAGAAGCTCAAAATACTAGA

IRN17|AB863152.1| GTTGATTACTCGAGCCAACTAAAGGAAATAAAATCCCTTTTAGAAGCTCAAAATACTAGA

IRN18|AB863153.1| GTTGATTACTCGAGTCAACTAAAGGAAATAAAATCCCTTTTAGAAGCTCAAAATACTAGA

IRN19|AB863154.1| GTTGATTACTCGAGTCAACTAAAAGAAATAAAATCCCTTTTAGAAGCTCAAAATACTAGA

IRN20|AB863155.1| GTTGATTACTCGAGCCAACTAAAAGAAATAAAATCCCTTTTAGAAGCTCAAAATACTAGA

IRN21|AB863156.1| GTTGATTACTCGAGCCAACTAAAAGAAATAAAATCCCTTTTAGAAGCTCAAAATACTAGA

JPNHGB340|AB863157.1| GTTGATTACTCGAGCCAACTAAAGGAAATAAAATCCCTTTTAGAAGCTCAAAACACTAGA

JPNKWB778|AB863158.1| GTTGATTACTCGAGCCAACTAAAGGAAATAAAATCCCTTTTAGAAGCTCAAAACACTAGA

JPNM|AB863159.1| GTTGATTACTCGAGCCAACTAAAGGAAATAAAATCCCTTTTAGAAGCTCAAAACACTAGA

JPNN|AB863160.1| GTTGATTACTCGAGCCAACTAAAAGATATAAAACCCCTTTTAGAAGCTCAAAACACTAGA

JPNS1|AB863161.1| GTTGATTACTCGAGCCAACTAAAAGATATAAAAACCCTTTTAGAAGCTCAAAACACTAGA

JPNS2|AB863162.1| GTTGATTACTCGAGCCAACTAAAAGATATAAAAACCCTTTTAGAAGCTCAAAACACTAGA

JPNTKD762|AB863163.1| GTTGATTACTCGAGCCAACTAAAAGAAATAAAATCCCTTTTAGAAGCTCAAAACACTAGA

JPNUV1|AB863164.1| GTTGATTACTCGAGCCAACTAAAGGAAATAAAATCCCTTTTAGAAGCTCAAAACACTAGA

JPNUV26|AB863165.1| GTTGATTACTCGAGCCAACTAAAGGAAA---AATACCTTTTAGAAGCTCAAAACACTAGA

TUR1|AB863166.1| GTTGATTACTCGAGCCAACTAAAGGAAATAAAATCCCTTTTAGAAGCTCAAAATACTAGA

TUR2|AB863167.1| GTTGATTATTCGAGCCAACTAAAGGAAATAAAATCCCTTTTAGAAGCTCAAAATACTAGA

TUR4|AB863168.1| GTTGATTATTCGAGCCAACTAAAGGAAATAAAATCCCTTTTAGAAGCTCAAAATACTAGA

TUR5|AB863169.1| GTTGATTATTCGAGCCAACTAAAAGAAATAAAATCCCTTTTAGAAGCTCAAAATACTAGA

TUR12|AB863170.1| GTTGATTACTCGAGCCAACTAAAAGAAATAAAATCCCTTTTAGAAGCTCAAAATACTAGA

TUR34|AB863171.1| GTTGATTACTCGAGCCAACTAAAGGAAATAAAATCCCTTTTAGAAGCTCAAAATACTAGA

TUR50|AB863172.1| GTTGATTACTCGAGTCAACTAAAAGAAATAAAATCTCTTTTAGAAGCTCAAAATACTAGA

TUR59|AB863173.1| GTTGATTACTCGAGCCAACTAAAAGAAATAAAATCCCTTTTAGAAGCTCAAAATACTAGA

TUR69|AB863174.1| GTTGATTACTCGAGCCAACTAAAGGAAATAAAATCCCTTTTAGAAGCTCAAAATACTAGA

TUR81|AB863175.1| GTTGATTATTCGAGCCAACTTAAGGAAATAAAATCCCTTTTAGAAGCTCAAAATACTAGA

TUR84|AB863176.1| GTTGATTATTCGAGCCAACTTAAGGAAATAAAATCCCTTTTAGAAGCTCAAAATACTAGA

TUR94|AB863177.1| GTTGATTACTCGAGCCAACTAAAGGAAATAAAATCCCTTTTGGAAGCTCAAAATACTAGA

TUR213|AB863178.1| GTTGATTATTCGAGCCAACTAAAGGAAATAAAATCCCTTTTAGAAGCTCAAAATACTAGA

TUR214|AB863179.1| GTTGATTATTCGAGCCAACTAAAGGAAATAAAATCCCTTTTGGAAGCTCAAAATACTAGA

TUR216|AB863180.1| GTTGATTATTCGAGCCAATTAAAGGAAATAAAATCCCTTTTAGAAGCTCAAAATACTAGA

TUR220|AB863181.1| GTTGATTATTCGAGCCAACTTAAGGAAATAAAATCCCTTTTAGAAGCTCAAAATACTAGA

TUR239|AB863182.1| GTTGATTACTCGAGCCAACTAAAGGAAATAAAATCCCTTTTAGAAGCTCAAAATACTAGA

TUR244|AB863183.1| GTTGATTACTCGAGCCAACTAAAGGAAATAAAATCCCTTTTAGAAGCTCAAAATACTAGA

TUR249|AB863184.1| GTTGATTATTCGAGCCAACTAAAGGAAATAAAATCCCTTTTAGAAGCTCAAAATACTAGA

TUR263|AB863185.1| GTTGATTACTCGAGCCAACTAAAGGAAATAAAATCCCTTTTAGAAGCTCAAAATACTAGA

TUR278|AB863186.1| GTTGATTACTCGAGCCAACTAAAGGAAATAAAATCCCTTTTAGAAGCTCAAAATACTAGA

TUR279|AB863187.1| GTTGATTATTCGAGCCAACTTAAGGAAATAAAATCCCTTTTAGAAGCTCAAAATACTAAA

TUR285|AB863188.1| GTTGATTACTCGAGCCAATTAAAGGAAATAAAATCCCTTTTAGAAGCTCAAAATACTAGA

TUR289|AB863189.1| GTTGATTACTCGAGCCAACTAAAGGAAATAAAATCCCTTTTAGAAGCTCAAAACACTAGA

TUR303|AB863190.1| GTTGATTATTCGAGCCAACTAAAGGAAATAAAATCCCTTTTAGAAGCTCAAAATACTAGA

TUR306|AB863191.1| GTTGATTATTCGAGCCAACTAAAGGAAATAAAATCCCTTTTAGAAGCTCAAAATACTAGA

Cabb-S|NC_001497.1| ATAAAAAGTCTAGAAAAAGCAATTCAATCCTTAGAAAATAAGATTGAACCAGAGCCCTTA

CRO180A|AB863192.1| ATTAAAAGTCTAGAAAAAGCAATTCAATCCTTAGATGAAAAGATTGAACCAGAGCCCTTA

GRC83|AB863193.1| ATTAAAAGTCTAGAAAAAGCAATTCAATCCTTAAATGAAAAGATTGAACCAGAGCCCTTA

GRC84B|AB863194.1| ATTAAAAGTCTAGAAAAAGCAATTCAATCCTTAGATGAAAAGATTGAACCAGAGCCCTTA

GRC86B|AB863195.1| ATTAAAAGTCTAGAAAAAGCAATTCAATCCTTAGATGAAAAGATTGAACCAGAGCCCTTA

GRC86D|AB863196.1| ATTAAAAGTCTAGAAAAAGCAATTCAATCCTTAGATGAAAAGATTGAACCAGAGCCCTTA

GRC87E|AB863197.1| ATTAAAAGTCTAGAAAAAGCAATTCAATCCTTAGATGAAAAGATTGAACCAGAGCCCTTA

GRC87G|AB863198.1| ATTAAAAGTCTAGAAAAAGCAATTCAATCCTTAGATGAAAAGATTGAACCAGAGCCCTTA

GRC91B|AB863199.1| ATTAAAAGTCTAGAAAAAGCAATTCAATCCTTAGATGAAAAGATTGAACCAGAGCCCTTA

GRC92A|AB863200.1| ATTAAAAGTCTAGAAAAAGCAATTCAATCCTTAGATGAAAAGATTGAACCAGAGCCCTTA

GRC92C|AB863201.1| ATTAAAAGTCTAGAAAAAGCAATTCAATCCTTAGATGAAAAGATTGAACCAGAGCCCTTA

GRC92D|AB863202.1| ATTAAAAGTCTAGAAAAAGCAATTCAATCCTTAGAAAATAAGATTGAACCAGAGCCCTTA

IRN1|AB863136.1| ATTAAAAATCTAGAAAAAGCAATTCAATCCTTAGATAATAAGATTGAACCAGAGCCCTTA

IRN2|AB863137.1| ATTAAAAGTCTAGAAAAAGCAATTCAATCCTTAGATAATAAGATTGAACCAGAGCCCTTA

IRN3|AB863138.1| ATTAAAAATCTAGAAAAAGCAATTCAATCCTTAGATAATAAGATTGAACCAGAGCCCTTA

IRN4|AB863139.1| ATTAAAAGTCTAGAAAAAGCAATTCAATCCTTAGAAAATAAGATTGAACCAGAGCCCTTA

IRN5|AB863140.1| ATTAAAAGTCTAGAAAAAGCAATTCAATCCTTAGAAAATAAGATTGAACCAGAGCCCTTA

IRN6|AB863141.1| A---------------------TTCAATCCTTAGAAAATAAGATTGAACCAGAACCCTTG

IRN7|AB863142.1| ATTAAAAATCTAGAAAAAGCAATTCAATCCTTAGAAAATAAGATTGAACCAGAGCCCTTA

IRN8|AB863143.1| ATTAAAAGTCTAGAAAAAGCAATTCAATCCTTAGAAAATAAGATTGAACCAGAACCCTTA

IRN9|AB863144.1| ATTAAAAGTCTAGAAAAAGCAATTCAATCCTTAGAAAATAAGATTGAACCAGAACCCTTG

IRN10|AB863145.1| ATTAAAAGTCTAGAAAAAGCAATTCAATCCTTAGAAAATAAGATTGAACCAGAGCCCTTA

IRN11|AB863146.1| ATTAAAAATCTAGAAAAAGCAATTCAATCCTTAGATAATAAGATTGAACCAGAGCCCTTA

IRN12|AB863147.1| ATTAAAAGTCTAGAAAAAACAATTCAATCCTTAGAAAATAAGATTGAACCAGAGCCCTTA

IRN13|AB863148.1| ATTAAAAGTCTAGAAAAAGCAATTCAATCCTTAGAAAATAAGATTGAACCAGAACCCTTG

IRN14|AB863149.1| ATTAAAAATCTAGAAAAAGCAATTCAATCCTTAGATAATAAGATTGAACCAGAGCCCTTA

IRN15|AB863150.1| ATTAAAAGTCTAGAAAAAGCAATTCAATCCTTAGATAATAAGATTGAACCAGAGCCCTTA

IRN16|AB863151.1| ATTAAAAGTCTAGAAAAAGCAATTCAATCCTTAGATAATAAGATTGAACCAGAGCCCTTA

IRN17|AB863152.1| ATTAAAAGTCTAGAAAAAGCAATTCAATCCTTAGATAATAAGATTGAACCAGAGCCCTTA

IRN18|AB863153.1| ATTAAAAGTCTAGAAAAAGCAATTCAATCCTTAGATAATAAGATTGAACCAGAGCCCTTA

IRN19|AB863154.1| ATTAAAAGTCTAGAAAAAGCAATTCAATCCTTAGATAATAAGATTGAACCAGAGCCCTTA

IRN20|AB863155.1| ATTAAAAGTCTAGAAAAAGCAATTCAATCCTTAGATAATAAGATTGAACCAGAGCCCTTA

IRN21|AB863156.1| ATTAAAAGTCTAGAAAAAGCAATTCAATCCTTAGATAATAAGATTGAACCAGAGCCCTTA

JPNHGB340|AB863157.1| ATTAAAAATCTAGAAAATGCAATTCAATCCTTAGATAATAAGATTAAACCAGAGCCCTTA

JPNKWB778|AB863158.1| ATTAAAAATCTAGAAAAAGCAATTCAATCCTTAGATAATAAGATTGAACCAGAGCCCTTA

JPNM|AB863159.1| ATTAAAAATCTAGAAAATGCAATTCAATCCTTAGATAATAAGATTGAACCAGAGCCCTTA

JPNN|AB863160.1| ATTAAAAATCTAGAAAATACAATTCAATCCTTAGATAATAAGATTGAACCAGAGCCTTTA

JPNS1|AB863161.1| ATTAAAAATCTAGAAAATGCGATTCAATCCTTAGATAATAAGATTGAACCAGAGCCTTTA

JPNS2|AB863162.1| ATTAAAAATCTAGAAAATGCGATTCAATCCTTAGATAATAAGATTGAACCAGAGCCTTTA

JPNTKD762|AB863163.1| ATTAAAAATCTAGAAAATGCAATTCAATCCTTAGATAATAAGATTGAACCAGAGCCCTTA

JPNUV1|AB863164.1| ATTAAAAATCTAGAAAATGCAATTCAATCCTTAGATAATAAGATTGAACCAGAGCCCTTA

JPNUV26|AB863165.1| ATTAAAAATCTAGAAAATGCAATTCAATCCTTAGATAATAAGATTGAACCAGAGCCCTTA

TUR1|AB863166.1| ATTAAAAGTCTAGAAAAAGCAATTCAATCCTTAGAAAATAAAATTGAACCAGAGCCCTTA

TUR2|AB863167.1| ATTAAAAGTCTAGAAAAAGCAATTCAATCCTTAGAAAATAAGATTGAACCAGAGCCCTTA

TUR4|AB863168.1| ATTAAAAGTCTAGAAAAAGCAATTCAATCCTTAGAAAATAAGATTGAACCAGAGCCCTTA

TUR5|AB863169.1| ATTAAAAGTCTAGAAAAAGCAATTCAATCTTTAGAAAATAAGATTGAACCAGAGCCCTTA

TUR12|AB863170.1| ATTAAAAATCTAGAAAAAGCAATTCAATCCTTAGATGATAAGATTGAACCAGAGCCCTTA

TUR34|AB863171.1| ATTAAAAATCTAGAAAAAGCAATTCAATCCTTAGACAATAAGATTGAACCAGAGCCCTTA

TUR50|AB863172.1| ATTAAAAATCTAGAAAAAGCAATTCAATCCTTAGATAATAAGATTGAACCAGAGCCCTTA

TUR59|AB863173.1| ATTAAAAGTCTAGAAAAAGCAATTCAATCCTTAGAAAATAAGATTGAACCAGAGCCCTTA

TUR69|AB863174.1| ATTAAAAGTCTAGAAAAAGCAATTCAATCCTTAGAAAATAAGATTGAACCAGAGCCCTTA

TUR81|AB863175.1| ATTAAAAGTCTAGAAAAAGCAATTCAATCCTTAGAAAATAAGATTGAACCAGAGCCCTTA

TUR84|AB863176.1| ATTAAAAGTCTAGAAAAAGCAATTCAATCCTTAGAAAATAAGATTGAACCAGAGCCCTTA

TUR94|AB863177.1| ATTAAAACTCTAGAAAAAGCAATTCAATCCTTAGATGATAAGATTGAACCAGAGCCCTTA

TUR213|AB863178.1| ATTAAAAGTCTAGAAAAAGCAATTCAATCCTTAGAAAATAAGATTGAACCAGAGCCCTTA

TUR214|AB863179.1| ATTAAAAGTCTAGAAAAAGCAATTCAATCCTTAGAAAACAAAATTGAACCAGAGCCCTTA

TUR216|AB863180.1| ATTAAAAGTCTAGAAAAAGCGATTCAATCCTTAGAAAATAAGATTGAACCAGAGCCCTTA

TUR220|AB863181.1| ATTAAAAGTCTAGAAAAAGCAATTCAATCCTTAGAAAATAAGATTGAACCAGAGCCCTTA

TUR239|AB863182.1| ATCAAAAGTCTAGAAAAAGCAATTCAATCCTTAGATGAAAAGATTGAACCAGAGCCCTTA

TUR244|AB863183.1| ATTAAAAGTCTAGAAAAAGCAATTCAATCCTTAGAAAATAAGATTGAACCAGAGCCCTTA

TUR249|AB863184.1| ATTAAAAGTCTAGAAAAAGCAATTCAATCCTTAGAAAATAAGATTGAACCAGAGCCCTTA

TUR263|AB863185.1| ATTAAAAGTCTAGAAAAAGCAATTCAATCCTTAGAAAATAAGATTGAACCAGAGCCCTTA

TUR278|AB863186.1| ATTAAAGGTCTAGAAAAAGCAATTCAATCCTTAGAAAATAAGATTGAACCAGAGCCCTTA

TUR279|AB863187.1| ATTAAAAGTCTAGAAAAAGCAATTCAATCCTTAGAAAATAAGATTGAACCAGAGCCCTTA

TUR285|AB863188.1| ATTAAAAGTCTAGAAAAAGCAATTCAATCCTTAGAAAATAAGATTGAACCAGAGCCCTTA

TUR289|AB863189.1| GTTAAAAGTCTAGAAAAAGCAATTCAATCCTTAGAGAATAAGATTGAACCAGAGCCCTTA

TUR303|AB863190.1| ATTAAAAGTCTAGAAAAAGCAATTCAATCCTTAGAAAATAAGATTGAACCAGAGCCCTTA

TUR306|AB863191.1| ATTAAAAGTCTAGAAAAAGCAATTCAATCCTTAGAAAATAAGATTGAACCAGAGCCCTTA

Cabb-S|NC_001497.1| ACTAAAGAGGAAGTTAAAGAGCTAAAAGAATCGATTAACTCGATCAAAGAAGGATTAAAG

CRO180A|AB863192.1| ACTAAAGAAGAAGTTAAAGAGCTTAAAGAATCGATTAACTCGATCAAAGAAGGATTAAAG

GRC83|AB863193.1| ACTAAAGAAGAAGTTAAAGAGCTAAAAGAATCGATAAATTCGATCAAAGAAGGATTAAAG

GRC84B|AB863194.1| ACTAAAGAAGAAGTTAAAGAGCTAAAAGAATCGATAAACTCGATCAAAGAAGGATTAAAG

GRC86B|AB863195.1| ACTAAAGAAGAAGTTAAAGAGCTAAAAGAATCGATTAACTCGATCAAAGAAGGATTAAAG

GRC86D|AB863196.1| ACTAAAGAAGAAGTTAAAGAGCTAAAAGAATCGATAAACTCGATCAAAGAAGGATTAAAG

GRC87E|AB863197.1| ACTAAAGAAGAAGTTAAAGAGCTAAAAGAATCGATAAATTCGATCAAAGAAGGATTAAAG

GRC87G|AB863198.1| ACTAAAGAAGAAGTTAAAGAGCTAAAAGAATCGATAAATTCGATCAAAGAAGGATTAAAG

GRC91B|AB863199.1| ACTAAAGAAGAAGTTAAAGAGCTAAAAGAATCGATAAACTCGATCAAAGAAGGATTAAAG

GRC92A|AB863200.1| ACTAAAGAAGAAGTTAAAGAGCTAAAAGAATCGATTAACTCGATCAAAGAAGGATTAAAG

GRC92C|AB863201.1| ACTAAAGAAGAAGTTAAAGAGCTAAAAGAATCGATTAACTCGATCAAAGAAGGATTAAAG

GRC92D|AB863202.1| ACTAAAGAAGAAGTTAAAGAGCTTAAAGAATCGATTAACTCGATCAAAGAAAGATTAAAG

IRN1|AB863136.1| ACTAAAGAAGAAGTTAAAGAGCTAAAAGAATCGATTAATTCGATCAAAGAAGGATTAAAG

IRN2|AB863137.1| ACTAAAGAAGAAGTTAAAGAGCTTAAAGAATCGATTAACTCGATCAAAGAAGGATTAAAG

IRN3|AB863138.1| ACTAAAGAAGAAGTTAAAGAGCTAAAAGAATCGATTAATTCGATCAAAGAAGGATTAAAG

IRN4|AB863139.1| ACTAAAGAAGAAGTTAAAGAGCTAAAAGAATCGATTAATTCGATCAAAGAAGGATTAAAG

IRN5|AB863140.1| ACTAAAGAAGAAGTTAAAGAGCTAAAAGAATCGATTAATTCGATCAAAGAAGGATTAAAG

IRN6|AB863141.1| ACTAAAGAAGAAGTTAAGGAACTTAAAGAATCGATTAACTCGATCAAAGAAGGATTAAAG

IRN7|AB863142.1| ACTAAAGAAGAAGTTAAAGAGCTAAAAGAATCGAT---------TAAAGAAGGATTAAAG

IRN8|AB863143.1| ACTAAAGAAGAAGTTAAGGAGCTAAAAGAATCGATTAATTCGATCAAAGAAGGATTAAAG

IRN9|AB863144.1| ACTAAAGAAGAAGTTAAGGAACTTAAAGAATCGATTAACTCGATCAAAGAAGGATTAAAG

IRN10|AB863145.1| ACTAAAGAAGAAGTTAAAGAGCTAAAAGAATCGATTAATTCGATCAAAGAAGGATTAAAG

IRN11|AB863146.1| ACTAAAGAAGAAGTTAAAGAGCTAAAAGAATCGATTAATTCGATCAAAGAAGGATTAAAG

IRN12|AB863147.1| ACTAAAGAAGAAGTTAAAGAGCTAAAAGAATCGATTAATTCGATCAAAGAAGGATTAAAG

IRN13|AB863148.1| ACTAAAGAAGAAGTTAAGGAACTTAAAGAATCGATTAACTCGATCAAAGAAGGATTAAAG

IRN14|AB863149.1| ACTAAAGAAGAAGTTAAAGAGCTAAAAGAATCGATTAATTCGATCAAAGAAGGATTAAAG

IRN15|AB863150.1| ACTAAAGAAGAAGTTAAAGAGCTTAAAGAATCGATTAACTCGATCAAAGAAGGATTAAAG

IRN16|AB863151.1| ACTAAAGAAGAAGTTAAAGAGCTTAAAGAATCGATTAACTCGATCAAAGAAGGATTAAAG

IRN17|AB863152.1| ACTAAAGAAGAAGTTAAAGAGCTTAAAGAATCGATTAACTCGATCAAAGAAGGATTAAAG

IRN18|AB863153.1| ACTAAAGAAGAAGTTAAAGAGCTAAAAGAATCGATTAATTCGATCAAAGAAGGATTAAAA

IRN19|AB863154.1| ACTAAAGAAGAAGTTAAAGAGCTAAAAGAATCGATTAATTCGATCAAAGAAGGATTAAAG

IRN20|AB863155.1| ACTAAAGAAGAAGTTAAAGAGCTTAAAGAATCGATTAACTCGATCAAAGAAGGATTAAAG

IRN21|AB863156.1| ACTAAAGAAGAAGTTAAAGAGCTAAAAGAATCGATTAACTCGATCAAAGAAGGATTAAAG

JPNHGB340|AB863157.1| ACTAAAGAAGAAGTTAAAGAGCTAAAAGAATCGATTAACTCGATCAAAGAAGGATTAAAG

JPNKWB778|AB863158.1| ACTAAAGAAGAAGTTAAAAAGCTAAAAGAATCGATTAACTCGATCAAAGAAGGATTAAAG

JPNM|AB863159.1| ACTAAAGAAGAAGTTAAAGAGCTAAAAGAATCGATTAACTCGATCAAAGAAGGATTAAAG

JPNN|AB863160.1| ACTAAAAAAGAAGTTAAAGAGCTAAAAGAATCGATTAACTCGATCAAAGAAGGATTAAAG

JPNS1|AB863161.1| ACTAA---AGAAGTTAAAGAGCTAAAAGAATCGATTAACTCGATCAAAGAAGGATTAAAG

JPNS2|AB863162.1| ACTAA---AGAAGTTAAAGAGCTAAAAGAATCGATTAACTCGATCAAAGAAGGATTAAAG

JPNTKD762|AB863163.1| ACTAAAGAAGAAGTTAAAGAGCTAAAAGAATCGATTAACTCGATCAAAGAAGGATTAAAG

JPNUV1|AB863164.1| ACTAAAGAAGAAGTTAAAGAGCTAAAAGAATCGATTAACTCGATCAAAGAAGGATTAGAG

JPNUV26|AB863165.1| ACTAAAGAAGAAGTTAAAGAGCTAAAAGAATCGATTAACTCGATCAAAGAAGGATTAAAG

TUR1|AB863166.1| ACTAAAGAAGAAGTTAAAGAGCTAAAAGAATCGATAAACTCGATCAAAGAAGGATTAAAG

TUR2|AB863167.1| ACTAAAGAAGAAGTTAAAGAGCTAAAAGAATCGATAAACTCGATCAAAGAAGGATTAAAG

TUR4|AB863168.1| ACTAAAGAAGAAGTTAAAGAGCTAAAAGAATCGATAAACTCGATCAAAGAAGGATTAAAG

TUR5|AB863169.1| ACTAAAGAAGAAGTTAAAGAGCTTAAAGAATCGATTAACTCGATCAAAGAAGGATTAAAG

TUR12|AB863170.1| ACTAAAGAAGAAGTTAAAGAGCTTAAAGAATCGATAAATTCGATCAAAGAAGGATTAAAG

TUR34|AB863171.1| ACTAAAGAAGAAGTTAAAGAGCTAAAAGAATCGATAAACTCGATCAAAGAAGGATTAAAG

TUR50|AB863172.1| ACTAAAGAAGAAGTTAAAGAGCTAAAAGAATCGATTAACTCGATCAAAGAAGGATTAAAG

TUR59|AB863173.1| ACTAAAGAAGAAGTTAAAGAGCTAAAAGAATCGATAAACTCGATCAAAGAAGGATTAAAG

TUR69|AB863174.1| ACTAAAGAAGAAGTTAAAGAGCTAAAAGAATCGATAAACTCGATCAAAGAAGAATTAAAG

TUR81|AB863175.1| ACTAAAGAAGAAGTTAAAGAGCTAAAAGAATCGATAAACTCGATCAAAGAAGGATTAAAG

TUR84|AB863176.1| ACTAAAGAAGAAGTTAAAGAGCTAAAAGAATCGATAAACTCGATCAAAGAAGGATTAAAG

TUR94|AB863177.1| ACTAAAGAAGAAGTTAAAGAGCTAAAAGAATCGATTAACTCGATCAAAGAAGGATTAAAG

TUR213|AB863178.1| ACTAAAGAAGAAGTTAAAGAGCTAAAAGAATCGATAAACTCGATCAAAGAAGGATTAAAG

TUR214|AB863179.1| ACTAAAGAAGAAGTTAAAGAGCTAAAAGAATCGATAAACTCGATCAAAGAAGGATTAAAG

TUR216|AB863180.1| ACTAAAGAAGAAGTTAAAGAGCTAAAAGAATCGATTAACTCGATCAAAGAAGGATTAAAG

TUR220|AB863181.1| ACTAAAGAAGAAGTTAAAGAGCTAAAAGAATCGATAAACTCGATCAAAGAAGGATTAAAG

TUR239|AB863182.1| ACTAAAGAAGAAGTTAAAGAGCTAAAAGAATCGATAAACTCGATCAAAGAAGGATTAAAG

TUR244|AB863183.1| ACTAAAGAAGAAGTTAAAGAGCTAAAAGAATCGATAAACTCGATCAAAGAAGGATTAAAG

TUR249|AB863184.1| ACTAAAGAAGAAGTTAAAGAGCTAAAAGAATCGATAAACTCGATCAAAGAAGGATTAAAG

TUR263|AB863185.1| ACTAAAGAAGAAGTTAAAGAGCTAAAAGAATCGATAAACTCGATCAAAGAAGGATTAAAG

TUR278|AB863186.1| ACTAAAGAAGAAGTTAAAGAGCTAAAAGAATCGATAAACTCGATCAAAGAAGGATTAAAG

TUR279|AB863187.1| ACTAAAGAAGAAGTTAAAGAGCTAAAAGAATCGATAAACTCGATCAAAGAAGGATTAAAG

TUR285|AB863188.1| ACTAAAGAAGAAGTTAAAGAGCTAAAAGAATCGATAAATTCGATCAAAGAAGGATTAAAG

TUR289|AB863189.1| ACTAAAGAAGAAGTTAAAGAGCTAAAAGAATCGATAAACTCGATCAAAGAAGGATTAAAG

TUR303|AB863190.1| ACTAAAGAGCTAA---AAGAATCGATAAACTCGAT---------CAAAGAAGGATTAAAG

TUR306|AB863191.1| ACTAAAGAAGAAGTTAAAGAGCTAAAAGAATCGATAAATTCGATCAAAGAAGGATTAAAG

Cabb-S|NC_001497.1| AATATTATTGGCTA-AAATGGCTAATCTTAATCAGATCCAAAAAGAAGTCTCTGAAATCC

CRO180A|AB863192.1| AATATTATTGGCTG-AAATGGCTAATCTTAATCAAATCCAAAAAGAAGTCTCTGAAATCC

GRC83|AB863193.1| AATATTATTGGCTA-AAATGGCTAATCTTAATCAAATCCAAAAAGAAGTCTCTGAAATCC

GRC84B|AB863194.1| AATATTATTGGCTG-AAATGGCTAATCTTAATCAAATCCAAAAAGAAGTCTCTGAAATCC

GRC86B|AB863195.1| AATATTATTGGCTA-AAATGGCTAATCTTAATCAAATCCAAAAAGAAGTCTCTGAAATCC

GRC86D|AB863196.1| AATATTATTGGCTA-AAATGGCTAATCTTAATCAAATCCAAAAAGAAGTCTCTGAAATCC

GRC87E|AB863197.1| AATATTATTGGCTA-AAATGGCTAATCTTAATCAAATCCAAAAAGAAGTCTCTGAAATCC

GRC87G|AB863198.1| AATATTATTGGCTA-AAATGGCTAATCTTAATCAAATCCAAAAAGAAGTCTCTGAAATCC

GRC91B|AB863199.1| AATATTATTGGCT-GAAATGGCTAATCTTAATCAAATCCAAAAAGAAGTCTCTGAAATCC

GRC92A|AB863200.1| AATATTATTGGCTA-AAATGGCTAATCTTAATCAAATCCAAAAAGAAGTCTCTGAAATCC

GRC92C|AB863201.1| AATATTATTGGCTA-AAATGGCTAATCTTAATCAAATCCAAAAAGAAGTCTCTGAAATCC

GRC92D|AB863202.1| AATATTATTGGCTAGAAATGGCTAATCTTAATCAAATCCAAAAAGAAGTCTCTGAAATCC

IRN1|AB863136.1| AATATTATTGGCT-AAAATGGCTAATCTTAATCAAATCCAAAAAGAAGTCTCTGAAATCC

IRN2|AB863137.1| AATATTATTGGCT-AAAATGGCTAATCTTAATCAAATCCAAAAAGAAGTCTCTGAAATCC

IRN3|AB863138.1| AATATTATTGGCT-AAAATGGCTAATCTTAATCAAATCCAAAAAGAAGTCTCTGAAATCC

IRN4|AB863139.1| AATATTATTGGCT-AAAATGGCTAATCTTAATCAAATCCAAAAAGAAGTCTCTGAAATCC

IRN5|AB863140.1| AATATTATTGGCT-AAAATGGCTAATCTTAATCAAATCCAAAAAGAAGTCTCTGAAATCC

IRN6|AB863141.1| AATATTATTGGCTA-AAATGGCTAATCTTAATCAAATCCAAAAAGAAGTCTCTGAAATCC

IRN7|AB863142.1| AATATTATTGGCT-AAAATGGCTAATCTTAATCAAATCCAAAAAGAAGTCTCTGAAATCC

IRN8|AB863143.1| AATATTATTGGCT-AAAATGGCTAATCTTAATCAAATCCAAAAAGAAGTCTCTGAAATCC

IRN9|AB863144.1| AATATTATTGGCT-AAAATGGCTAATCTTAATCAAATCCAAAAAGAAGTCTCTGAAATCC

IRN10|AB863145.1| AATATTATTGGCT-AAAATGGCTAATCTTAATCAAATCCAAAAAGAAGTCTCTGAAATCC

IRN11|AB863146.1| AATATTATTGGCT-AAAATGGCTAATCTTAATCAAATCCAAAAAGAAGTCTCTGAAATCC

IRN12|AB863147.1| AATATTATTGGCT-AAAATGGCTAATCTTAATCAAATCCAAAAAGAAGTCTCTGAAATCC

IRN13|AB863148.1| AATATTATTGGCT-AAAATGGCTAATCTTAATCAAATCCAAAAAGAAGTCTCTGAAATCC

IRN14|AB863149.1| AATATTATTGGCT-AAAATGGCTAATCTTAATCAAATCCAAAAAGAAGTCTCTGAAATCC

IRN15|AB863150.1| AATATTATTGGCT-AAAATGGCTAATCTTAATCAAATCCAAAAAGAAGTCTCTGAAATCC

IRN16|AB863151.1| AATATTATTGGCTA-AAATGGCTAATCTTAATCAAATCCAAAAAGAAGTCTCTGAAATCC

IRN17|AB863152.1| AATATTATTGGCTA-AAATGGCTAATCTTAATCAAATCCAAAAAGAAGTCTCTGAAATCC

IRN18|AB863153.1| AATATTATTGGCT-AAAATGGCTAATCTTAATCAAATCCAAAAAGAAGTCTCTGAAATCC

IRN19|AB863154.1| AATATTATTGGCT-AAAATGGCTAATCTTAATCAAATCCAAAAAGAAGTCTCTGAAATCC

IRN20|AB863155.1| AATATTATTGGCTA-AAATGGCTAATCTTAATCAAATCCAAAAAGAAGTCTCTGAAATCC

IRN21|AB863156.1| AATATTATTGGCT-AAAATGGCTAATCTTAATCAAATCCAAAAAGAAGTCTCTGAAATCC

JPNHGB340|AB863157.1| AATATTATTGGCTG-AAATGGCTAATCTTAATCAAATCCAGAAAGAAGTCTCTGAAATCC

JPNKWB778|AB863158.1| AATATTATTGGCTG-AAATGGCTAATCTTAATCAAATCCAGAAAGAAGTCTCTGAAATCC

JPNM|AB863159.1| AATATTATTGGCTG-AAATGGCTAATCTTAATCAAATCCAGAAAGAAGTCTCTGAAATCC

JPNN|AB863160.1| ATTATTATTGGCTG-AAATGGCTAATCTTAATCAGATCCAGAAAGAAGTCTCTGAAATCC

JPNS1|AB863161.1| ATTATTATTGGCTA-AAATGGCTAATCTTAATCAAATCCAGAAAGAAGTCTCTGAAATCC

JPNS2|AB863162.1| ATTATTATTGGCTA-AAATGGCTAATCTTAATCAAATCCAGAAAGAAGTCTCTGAAATCC

JPNTKD762|AB863163.1| AATATTATTGGCTG-AAATGGCTAATCTTAATCAAATCCAGAAAGAAGTCTCTGAAATCC

JPNUV1|AB863164.1| AATATTATTGGCTG-AAATGGCTAATCTTAATCAAATCCAGAAAGAAGTCTCTGAAATTC

JPNUV26|AB863165.1| AATATTATTGGCTG-AAATGGCTAATCTTAATCAAATCCAGAAAGAAGTCTCTGAAATTC

TUR1|AB863166.1| AATATTATTGGCT-GAAATGGCTAATCTTAATCAAATCCAAAAAGAAGTCTCTGAAATCC

TUR2|AB863167.1| AATATAATTGGCT-GAAATGGCTAATCTTAATCAAATCCAAAAAGAAGTCTCTGAAATCC

TUR4|AB863168.1| AATATTATTGGCT-GAAATGGCTAATCTTAATCAAATCCAAAAAGAAGTCTCTGAAATCC

TUR5|AB863169.1| AATATTATTGGCT-GAAATGGCTAATCTTAATCAAATCCAAAAAGAAGTCTCTGAAATCC

TUR12|AB863170.1| AATATTATTGGCT-GAAATGGCTAATCTTAATCAAATCCAAAAAGAAGTCTCTGAAATCC

TUR34|AB863171.1| AATATTATTGGCT-GAAATGGCTAATCTTAATCAAATCCAAAAAGAAGTCTCTGAAATTC

TUR50|AB863172.1| AATATTATTGGCT-AAAATGGCTAATCTTAATCAAATCCAAAAAGAAGTCTCTGAAATCC

TUR59|AB863173.1| AATATTATTGGCTG-AAATGGCTAATCTTAATCAAATCCAAAAAGAAGTCTCTGAAATCC

TUR69|AB863174.1| AATATTATTGGCTA-AAATGGCTAATCTTAATCAAATCCAAAAAGAAGTCTCTGAAATCC

TUR81|AB863175.1| AATATTATTGGCT-GAAATGGCTAATCTTAATCAAATCCAAAAAGAAGTCTCTGAAATCC

TUR84|AB863176.1| AATATTATTGGCT-GAAATGGCTAATCTTAATCAAATCCAAAAAGAAGTCTCTGAAATCC

TUR94|AB863177.1| AATATTATTGGCTA-AAATGGCTAATCTTAATCAAATCCAAAAAGAAGTCTCTGAAATCC

TUR213|AB863178.1| AATATTATTGGCT-GAAATGGCTAATCTTAATCAAATCCAAAAAGAAGTCTCTGAAATCC

TUR214|AB863179.1| AATATTATTGGCT-GAAATGGCTAATCTTAATCAAATCCAAAAAGAAGTCTCTGAAATCC

TUR216|AB863180.1| AATATTATTGGCT-AAAATGGCTAATCTTAATCAAATCCAAAAAGAAGTCTCTGAAATCC

TUR220|AB863181.1| AATATTATTGGCT-GAAATGGCTAATCTTAATCAAATCCAAAAAGAAGTCTCTGAAATCC

TUR239|AB863182.1| AATATTATTGGCTA-AAATGGCTAATCTTAATCAAATCCAAAAAGAAGTCTCTGAAATCC

TUR244|AB863183.1| AATATTATTGGCT-GAAATGGCTAATCTTAATCAAATCCAAAAAGAAGTCTCTGAAATCC

TUR249|AB863184.1| AATATTATTGGCT-GAAATGGCTAATCTTAATCAAATCCAAAAAGAAGTCTCTGAAATCC

TUR263|AB863185.1| AATATTATTGGCTA-AAATGGCTAATCTTAATCAAATCCAAAAAGAAGTCTCTGAAATCC

TUR278|AB863186.1| AATATTATTGGCT-GAAATGGCTAATCTTAATCAAATCCAAAAAGAAGTCTCTGAAATCC

TUR279|AB863187.1| AATATTATTGGCT-GAAATGGCTAATCTTAATCAAATCCAAAAAGAAGTCTCTGAAATCC

TUR285|AB863188.1| AATATTATTGGCTG-AAATGGCTAATCTTAATCAAATCCAAAAAGAAGTCTCTGAAATCC

TUR289|AB863189.1| AATATTATTGGCT-GAAATGGCTAATCTTAATCAAATCCAAAAAGAAGTCTCTGAAATCC

TUR303|AB863190.1| AATATTATTGGCT-GAAATGGCTAATCTTAATCAAATCCAAAAAGAAGTCTCTGAAATCC

TUR306|AB863191.1| AATATTATTGGCT-GAAATGGCTAATCTTAATCAAATCCAAAAAGAAGTCTCTGAAATCC

Cabb-S|NC_001497.1| TCAGTGACCAAAAATCCATGAAAGCGGATATAAAAGCTATCTTAGAATTATTAGGATCCC

CRO180A|AB863192.1| TCAGTGACCAAAAATCCATGAAAGCGGATATAAAAGCTATCTTAGAATTATTAGGATCCC

GRC83|AB863193.1| TCAGTGACCAAAAATCCATGAAAACGGATATAAAAGCTATCTTAGAACTATTAGGATCCC

GRC84B|AB863194.1| TCAGTGACCAAAAATCCATGAAAGCGGATATAAAAGCTATCTTAGAATTATTAGGATCCC

GRC86B|AB863195.1| TCAGTGACCAAAAATCCATGAAAGCGGATATAAAAGCTATCTTAGAATTATTAGGATCCC

GRC86D|AB863196.1| TCAGTGACCAAAAATCCATGAAAGCGGATATAAAAGCTATCTTAGAATTATTAGGATCCC

GRC87E|AB863197.1| TCAGTGACCAAAAATCCATGAAAGCGGATATAAAAGCTATCTTAGAACTATTAGGATCCC

GRC87G|AB863198.1| TCAGTGACCAAAAATCCATGAAAGCGGATATAAAAGCTATCTTAGAACTATTAGGATCCC

GRC91B|AB863199.1| TCAGTGACCAAAAATCCATGAAAGCGGATATAAAAGCTATCTTAGAATTATTAGGATCCC

GRC92A|AB863200.1| TCAGTGACCAAAAATCCATGAAAACGGATATAAAAGCTATCTTAGAATTATTAGGATCCC

GRC92C|AB863201.1| TCAGTGACCAAAAATCCATGAAAACGGATATAAAAGCTATCTTAGAATTATTAGGATCCC

GRC92D|AB863202.1| TCAGTGACCAAAAATCCATGAAAACGGATATAAAAGCTATCTTAGAATTATTAGGATCCC

IRN1|AB863136.1| TCAGTGACCAGAAATCCATGAAAATGGATATAAAAGCTATCTTAGATATATTAGGATCTC

IRN2|AB863137.1| TCAGCGACCAGAAATCCATGAAAATGGATATAAAAGCTATCTTAGATATATTAGGATCTC

IRN3|AB863138.1| TCAGTGACCAGAAATCCATGAAAATGGATATAAAAGCTATCTTAGATATATTAGGATCTC

IRN4|AB863139.1| TCAGTGACCAGAAATCCATGAAAATGGATATAAAAGCTATCTTAGATATATTAGGATCTC

IRN5|AB863140.1| TCAGTGACCAGAAATCCATGAAAATGGATATAAAAGCTATCTTAGATATATTAGGATCTC

IRN6|AB863141.1| TCAGTGACCAAAAATCCATGAAATCGGATATAAAAGCTATCTTAGAATTATTAGGATCCC

IRN7|AB863142.1| TCAGTGACCAGAAATCCATGAAAATGGATATAAAAGCTATCTTAGATATATTAGGATCTC

IRN8|AB863143.1| TCAGTGACCAGAAATCCATGAAAATGGATATAAAAGCTATCTTAGATATATTAGGATCTC

IRN9|AB863144.1| TCAGTGACCAAAAATCCATGAAATCGGATATAAAAGCTATCTTAGAATTATTAGGATCCC

IRN10|AB863145.1| TCAGTGACCAGAAATCCATGAAAATGGATATAAAAGCTATCTTAGATATATTAGGATCTC

IRN11|AB863146.1| TCAGTGACCAGAAATCCATGAAAATGGATATAAAAGCTATCTTAGATATATTAGGATCTC

IRN12|AB863147.1| TCAGTGACCAGAAATCCATGAAAATGGATATAAAAGCTATCTTAGATATATTAAGATCTC

IRN13|AB863148.1| TCAGTGACCAAAAATCCATGAAATCGGATATAAAAGCTATCTTAGAATTATTAGGATCCC

IRN14|AB863149.1| TCAGTGACCAAAAATCCATGAAATCGGATATAAAAGCTATCTTAGAATTATTAGGATCCC

IRN15|AB863150.1| TCAGTGACCAAAAATCCATGAAAACGGATATAAAAGCTATCTTAGAATTATTAGGATCCC

IRN16|AB863151.1| TCAGTGACCAAAAATCCATGAAAGCGGATATAAAAGCTATCTTAGAATTAATAGGATCCC

IRN17|AB863152.1| TCAGTGACCAAAAATCCATGAAAGCGGATATAAAAGCTATCTTAGAATTATTAGGATCCC

IRN18|AB863153.1| TCAGTGACCAGAAATCCATGAAAATGGATATAAAAGCTATCTTAGATATATTAGGATCTC

IRN19|AB863154.1| TCAGTGACCAGAAATCCATAAAAATGGATATAAAAGCTATCTTAGATATATTAGGATCTC

IRN20|AB863155.1| TCAGTGACCAAAAATCCATGAAAACGGATATAAAAGCTATCTTAGAATTATTAGGATCCC

IRN21|AB863156.1| TCAGTGACCAAAAATCCATGAAATCGGATATAAAAGCTATCTTAGAATTATTAGGATCCC

JPNHGB340|AB863157.1| TCAGCGACCAAAAATCCATGAAATCGGATATAAAAGCTATCTTAGAATTGCTAGGATCCC

JPNKWB778|AB863158.1| TCAGCGACCAAAAATCCATGAAATCGGATATAAAAGCTATCTTAGAATTGCTAGGATCCC

JPNM|AB863159.1| TCAGCGACCAAAAATCCATGAAATCGGATATAAAAGCTATCTTAGAATTGCTAGGATCCC

JPNN|AB863160.1| TCAGTGACTTAAAATCCATGAAAACGGATATAAAAGCTATCTTAGAATTGCTAGGATCCC

JPNS1|AB863161.1| TCAGTGACTTAAAATCCATGAAATTGGATATAAAAGCTATCTTAGAATTGCTAGGATCCC

JPNS2|AB863162.1| TCAGTGACTTAAAATCCATGAAATTGGATATAAAAGCTATCTTAGAATTGCTAGGATCCC

JPNTKD762|AB863163.1| TCAGCGACCAAAAATCCATGAAATCGGATATAAAAGCTATCTTAGAATTGCTAGGATCCC

JPNUV1|AB863164.1| TCAGCGACCAAAAATCCATGAAATCGGATATAAAAGCTATCTTAGAATTGCTAGGATCCC

JPNUV26|AB863165.1| TCAGCGACCAAAAATCCATGAAATCGGATATAAAAGCTATCTTAGAATTGCTAGGATCCC

TUR1|AB863166.1| TCAGCGACCAAAAATCCATGAAAACGGATATAAAAGCTATCTTAGAAATATTAGGATCCC

TUR2|AB863167.1| TCAGTGACCAAAAATCCATGAAAACGGATATAAAAGCTATCTTAGATTTATTAGGATCTC

TUR4|AB863168.1| TCAGTGACCAAAAATCCATGAAAACGGATATAAAAGCTATCTTAGATTTATTAGGATCTC

TUR5|AB863169.1| TCAGCGACCAAAAATCCATGAAAGCGGATATAAAAGCTATCTTAGATTTATTAGGATCCC

TUR12|AB863170.1| TCAGTGACCAGAAGTCCATGAAAATGGATATAAAAGCTATCTTAGATATATTAGGATCTC

TUR34|AB863171.1| TCAGTGACCAAAAATCCATGAAAACGGATATAAAAGCTATCTTAGAATTATTAGGATCCC

TUR50|AB863172.1| TCAGTGACCAAAAATCCATGAAAGCGGATATAAAAGCTATCTTAGAATTATTAGGATCCC

TUR59|AB863173.1| TCAGTGACCAGAAGTCCATGAAAATGGATATAAAAGCTATCTTAGAATTATTAGGATCCC

TUR69|AB863174.1| TCAGTGACCAGAAGTCCATGAAAATGGATATAAGAGCTATCTTAGATATATTAGGATCTC

TUR81|AB863175.1| TCAGTGACCAAAAATCCATGAAGACGGATATAAAAGCTATCTTAGAAATACTAGGATCTC

TUR84|AB863176.1| TCAGTGACCAAAAATCCATGAAAACGGATATAAAAGCTATCTTAGAAATATTAGGATCTC

TUR94|AB863177.1| TCAGTGACCAGAAGTCCATGAAAATGGATATAAAAGCTATCTTAGATATATTAGGATCCC

TUR213|AB863178.1| TCAGCGACCAAAAATCCATGAAAGCGGATATAAAAGCTATCTTAGATATATTAGGATCCC

TUR214|AB863179.1| TCAGCGACCAAAAATCCATGAAAGCGGATATAAAAGCTATCTTAGAATTATTAGGATCCC

TUR216|AB863180.1| TCAGTGACCAAAAATCCATGAAAATGGATATAAAAGCTATCTTAGATATATTAGGATCTC

TUR220|AB863181.1| TCAGTGACCAAAAATCCATGAAAACGGATATAAAAGCTATCTTAGAAATATTAGGATCTC

TUR239|AB863182.1| TCAGTGACCAAAAATCCATGAAAGCGGATATAAAAGCTATCTTAGAGTTATTAGGATCCC

TUR244|AB863183.1| TCAGTGACCAAAAATCCATGAAAGCGGATATAAAAGCTATCTTAGATATATTAGGATCTC

TUR249|AB863184.1| TCAGCGACCAAAAATCCATGAAAGCGGATATAAAAGCTATCTTAGATATATTAGGATCCC

TUR263|AB863185.1| TCAGTGACCAGAAGTCCATGAAAATGGATATAAAAGCTATCTTAGACATATTAGGATCTC

TUR278|AB863186.1| TCAGTGACCAAAAATCCATGAAAACGGATATAAAAGCTATCTTAGAAATATTAGGATCTC

TUR279|AB863187.1| TCAGTGACCAAAAATCCATGAAAACGGATATAAAAGCTATCTTAGAAATATTAGGATCTC

TUR285|AB863188.1| TCAGTGACCAAAAATCCATGAAAACGGATATAAAAACTATCTTAGAATTATTAGGATCCC

TUR289|AB863189.1| TCAGTGACCAAAAATCCATGAAAGCGGATATAAAAGCTATCTTAGAATTATTAGGATCCC

TUR303|AB863190.1| TCAGTGACCAAAAATCCATGAAAACGGATATAAAAGCTATCTTAGATATATTAGGATCTC

TUR306|AB863191.1| TCAGCGACCAAAAATCCATGAAAGCGGATATAAAAGCTATCTTAGATATATTAGGATCTC

Cabb-S|NC_001497.1| AAAATCCTATTAAAGAAAGCTTAGAAACCGTTGCAGCAAAAATCGTTAATGACTTAACCA

CRO180A|AB863192.1| AAAATCCTATTAAAGAAAGCTTAGAAGCCGTTGCAGCGAAAATCGTTAATGACTTAACCA

GRC83|AB863193.1| AAAATCCTATTAAAGAAAGCTTAGAAGCTGTTGCAGCGAAAATCGTTAATGACTTAACCA

GRC84B|AB863194.1| AAAATCCTATTAAAGAAAGCTTAGAAGCCGTTGCAGCAAAAATCGTTAATGACTTAACCA

GRC86B|AB863195.1| AAAATCCTATTAAAGAAAGCTTAGAAGCCGTTGCAGCGAAAATCGTTAATGACTTAACCA

GRC86D|AB863196.1| AAAATCCTATTAAAGAAAGCTTAGAAGCCGTTGCAGCGAAAATCGTTAATGACTTAACCA

GRC87E|AB863197.1| AAAATCCTATTAAAGAAAGCTTAGAAGCTGTTGCAGCGAAAATCGTTAATGACTTAACCA

GRC87G|AB863198.1| AAAATCCTATTAAAGAAAGCTTAGAAGCTGTTGCAGCGAAAATCGTTAATGACTTAACCA

GRC91B|AB863199.1| AAAATCCTATTAAAGAAAGCTTAGAAGCTGTTGCAGCGAAAATCGTTAATGACTTAACCA

GRC92A|AB863200.1| AAAACCCTAATAAAGAAAGCTTAGAAGCTGTTGCAGCGAAAATCGTTAATGACTTAACCA

GRC92C|AB863201.1| AAAACCCTAATAAAGAAAGCTTAGAAGCTGTTGCAGCGAAAATCGTTAATGACTTAACCA

GRC92D|AB863202.1| AAAATCCTATTAAAGAAAGCTTAGAAACCGTTGCAGCGAAAATCGTTAATGACTTAACCA

IRN1|AB863136.1| AAAACCTTAATAAAGAAAGCTTAGAAGCTGTTGCAGCGAAAATTGTTAATGACTTAACCA

IRN2|AB863137.1| AAAACCCTAATAAAGAAAGCTTAGAAGCCGTTGCAGCGAAAATCGTTAATGACTTAACCA

IRN3|AB863138.1| AAAACCTTAATAAAGAAAGCTTAGAAGCTGTTGCAGCGAAAATTGTTAATGACTTAACCA

IRN4|AB863139.1| AAAATCCTAATAAAGAAAGCTTAGAAGCTGTTGCAGCGAAAATTGTTAATGACTTAACCA

IRN5|AB863140.1| AAAACCCTAATAAAGAAAGCTTAGAAGCTGTTGCAGCGAAAATTGTTAATGACTTAACCA

IRN6|AB863141.1| AAAATCCTATTAAAGAAAGCTTAGAAACCGTTGCAGCGAAAATCGTTAATGACTTAACCA

IRN7|AB863142.1| AAAACCCTAATAAAGAAAGCTTAGAAGCTGTTGCAGCGAAAATTATTAATGACTTAACCA

IRN8|AB863143.1| AAAACCCTAATAAAGAAAGCTTAGAAGCTGTTGCAGCGAAAATTGTTAATGACTTAACCA

IRN9|AB863144.1| AAAATCCTATTAAAGAAAGCTTGGAAACCGTTGCAGCGAAAATCGTTAATGACTTAACCA

IRN10|AB863145.1| AAAACCCTAATAAAGAAAGCTTAGAAGCTGTTGCAGCGAAAATTGTTAATGACTTAACCA

IRN11|AB863146.1| AAAACCCTAATAAAGAAAGCTTAGAAGCCGTTGCAGCGAAAATCGTTAATGACTTAACCA

IRN12|AB863147.1| AAAACCCTAATAAAGAAAGCTTAGAAGCTGTTGCAGCGAAAATTGTTAATGACTTAACCA

IRN13|AB863148.1| AAAATCCTATTAAAGAAAGCTTAGAAACCGTTGCAGCGAAAATCGTTAATGACTTAACCA

IRN14|AB863149.1| AAAATCCTATTAAAGAAAGCTTAGAAACCGTTGCAGCGAAAATCGTTAATGACTTAACCA

IRN15|AB863150.1| AAAATCCTATTAAAGAAAGCTTAGAAGCCGTTGCAGCGAAAATCGTTAATGACTTAACCA

IRN16|AB863151.1| AAAATCCTATTAAAGAAAGCTTAGAAGCCGTTGCAGCGAAAATCGTTAATGACTTAACCA

IRN17|AB863152.1| AAAATCCTATTAAAGAAAGCTTAGAAGCCGTTGCAGCGAAAATCGTTAATGACTTAACCA

IRN18|AB863153.1| AAAACCCTAATAAAGAAAGCTTAGAAGCCGTTGCAGCGAAAATCGTTAATGACTTAACCA

IRN19|AB863154.1| AAAACCCTAATAAAGAAAGCTTAGAAGCCGTTGCAGCGAAAATCGTTAATGACTTAACCA

IRN20|AB863155.1| AAAATCCTATTAAAGAAAGCTTAGAAGCCGTTGCAGCGAAAATCGTTAATGACTTAACCA

IRN21|AB863156.1| AAAATCCTATTAAAGAAAGCTTAGAAACCGTTGCAGCGAAAATCGTTAATGACTTAACCA

JPNHGB340|AB863157.1| AAAATCCTACTAAAGAAAGCTTAGAAGCCGTTGCAGCGAAAATCGTTAATGACTTAACCA

JPNKWB778|AB863158.1| AAAATCCTACTAAAGAAAGCTTAGAAGCCGTTGCAGCGAAAATCGTTAATGACTTAACCA

JPNM|AB863159.1| AAAATCCTACTAAAGAAAGCTTAGAAGCCGTTGCAGCGAAAATCGTTAATGACTTAACCA

JPNN|AB863160.1| AAAATCCTGCTAAAGAAAGCTTAGAAACCGTTGCAGCGAAAATCGTTAATGACTTAACTA

JPNS1|AB863161.1| AAAATCCTACTAAAGAAAGCTTAGAAACCGTTGCAGCGAAAATCGTTAATGACTTAACCA

JPNS2|AB863162.1| AAAATCCTACTAAAGAAAGCTTAGAAACCGTTGCAGCGAAAATCGTTAATGACTTAACCA

JPNTKD762|AB863163.1| AAAATCCTACTAAAGAAAGCTTAGAAGCCGTTGCAGCGAAAATCGTTAATGACTTAACCA

JPNUV1|AB863164.1| AAAATCCTACTAAAGAAAGCTTAGAAGCCGTTGCAGCGAAAATCGTTAATGACTTAACCA

JPNUV26|AB863165.1| AAAATCCTACTAAAGAAAGCTTAGAAGCCGTTGCAGCGAAAATCGTTAATGACTTAACCA

TUR1|AB863166.1| AAAATCCTATTAAAGAAAGCTTAGAAACCGTTGCAGCGAAAATCGTTAATGACTTAACCA

TUR2|AB863167.1| AAAACCCTAATAAAGAAAGCTTAGAAGCTGTTGCAGCGAAAATCGTTAATGACTTAACCA

TUR4|AB863168.1| AAAACCCTAATAAAGAAAGCTTAGAAGCTGTTGCAGCAAAAATCGTTAATGACTTAACCA

TUR5|AB863169.1| AAAATCCTATCAAAGAAAGCTTAGAATCTGTTGCAGCGAAAATCGTTAATGACTTAACCA

TUR12|AB863170.1| AAAACCCTAATAAAGAAAGCTTAGAAGCCGTTGCAGCGAAAATCGTTAATGACTTAACCA

TUR34|AB863171.1| AAAATCCTATTAAAGAAAGCTTAGAAGCTGTTGCAGCGAAGATCGTTAATGACTTAAGCA

TUR50|AB863172.1| AAAATCCTATTAAAGAAAGCTTAGAAGCTGTTGCAGCGAAAATCGTTAATGACTTAACCA

TUR59|AB863173.1| AAAATCCTATTAAAGAAAGCTTAGAAACCGTTGCAGCGAAAATCGTTAATGACTTAACCA

TUR69|AB863174.1| AAAACCCTAATAAAGAAAGCTTAGAAGCCGTTGCAGCGAAAATCGTTAATGACTTAACCA

TUR81|AB863175.1| AAAACCCTAATAAAGGAAGCTTAGAAGCTGTTGCAGCGAAAATCGTTAATGACTTAACCA

TUR84|AB863176.1| AAAACCCTAATAAAGGAAGCTTAGAAGCTGTTGCAGCGAAAATCGTTAATGACTTAACCA

TUR94|AB863177.1| AAAACCCTAATAAAGAAAGCTTAGAAGCCGTTGCAGCGAAAATCGTTAATGACTTAACCA

TUR213|AB863178.1| AAAACCCTAATAAAGAAAGCTTAGAAGCTGTTGCAGCGAAAATCGTTAATGACTTAACCA

TUR214|AB863179.1| AAAATCCTATTAAAGAAAGCTTAGAAGCCGTTGCAGCGAAAATCGTTAATGACTTAACCA

TUR216|AB863180.1| AAAATCCTAATAAAGAAAGCTTAGAAGCTGTTGCAGCGAAAATCGTTAATGACTTAACCA

TUR220|AB863181.1| AAAACCCTAATAAAGGAAGCTTAGAAGCTGTTGCAGCGAAAATCGTTAATGACTTAACCA

TUR239|AB863182.1| AAAATCCTATTAAAGAAAGCTTAGAAACCGTTGCAGCGAAAATCGTTAATGACTTAACCA

TUR244|AB863183.1| AAAACCCTAATAAAGAAAGCTTAGAAGCTGTTGCAGCGAAAATCGTTAATGACTTAACCA

TUR249|AB863184.1| AAAACCCTAATAAAGAAAGCTTAGAAGCTGTTGCAGCGAAAATCGTTAATGACTTAACCA

TUR263|AB863185.1| AAAACCCTAATAAAGAAAGCTTAGAAGCCGTTGCAGCGAAAATCGTTAATGACTTAACCA

TUR278|AB863186.1| AAAACCCTAATAAAGGAAGCTTAGAAGCTGTTGCAGCGAAAATCGTTAATGACTTAACCA

TUR279|AB863187.1| AAAACCCTAATAAAGGAAGCTTAGAAGCTGTTGCAGCAAAAATCGTTAATGACTTAACCA

TUR285|AB863188.1| AAAATCCTATTAAAGAAAGCTTAGAAACCGTTGCAGCGAAAATCGTTAATGACTTAACCA

TUR289|AB863189.1| AAAATCCTATTAAAGAAAGCTTAGAAACCGTTGCAGCGAAAATCGTTAATGACTTAACCA

TUR303|AB863190.1| AAAACCCTAATAAAGAAAGCTTAGAAGCTGTTGCAGCGAAAATCGTTAATGACTTAACCA

TUR306|AB863191.1| AAAACCCTAATAAAGAAAGCTTAGAAGCTGTTGCAGCGAAAATCGTTAATGACTTAACCA

Cabb-S|NC_001497.1| AGCTCATCAATGATTGTCCTTGTAACAAAGAGATATTAGAAGCCTTAGGTACCCAACCTA

CRO180A|AB863192.1| AGCTCATCAATGATTGTCCTTGTAACAAAGAAATATTAGAAGCCTTAGGCAACCAACCTA

GRC83|AB863193.1| AGCTCATCAATGATTGTCCTTGTAACAAAGAAATATTAGAAGCCTTAGGCAACCAACCTA

GRC84B|AB863194.1| AGCTCATCAATGATTGTCCTTGCAACAAAGAAATATTAGAAGCCTTAGGCAACCAACCTA

GRC86B|AB863195.1| AGCTCATCAATGATTGTCCTTGTAACAAAGAAATATTAGAAGCCTTAGGCAACCAACCTA

GRC86D|AB863196.1| AGCTCATCAATGATTGTCCTTGTAACAAAGAAATATTAGAAGCCTTAGGCAACCAACCTA

GRC87E|AB863197.1| AGCTCATCAATGATTGTCCTTGTAACAAAGAAATATTAGAAGCCTTAGGCAACCAACCTA

GRC87G|AB863198.1| AGCTCATCAATGATTGTCCTTGTAACAAAGAAATATTAGAAGCCTTAGGCAACCAACCTA

GRC91B|AB863199.1| AGCTCATCAATGATTGTCCTTGTAACAAAGAAATATTAGAAGCCTTAGGCAACCAACCTA

GRC92A|AB863200.1| AGCTCATCAATGATTGTCCTTGTAACAAAGAGATATTAGAAGCCTTAGGCAACCAACCTA

GRC92C|AB863201.1| AGCTCATCAATGATTGTCCTTGTAACAAAGAGATATTAGAAGCCTTAGGCAACCAACCTA

GRC92D|AB863202.1| AGCTCATCAATGATTGTCCTTGTAACAAAGAGATATTAGAAGCCTTAGGCAACCAACCTA

IRN1|AB863136.1| AGCTCATCAACGATTGTCCTTGTAACAAAGAAATATTAGAAGCCTTAGGAAATCAACCTA

IRN2|AB863137.1| AGCTCATCAATGATTGTCCTTGTAACAAAGAAATATTAGAAGCCTTAGGCAACCAGCCTA

IRN3|AB863138.1| AGCTCATCAACGATTGTCCTTGTAACAAAGAAATATTAGAAGCCTTAGGAAATCAACCTA

IRN4|AB863139.1| AGCTCATCAACGATTGTCCTTGTAACAAAGAAATATTAGAAGCCTTAAGAAATCAACCTA

IRN5|AB863140.1| AGCTCATCAACGATTGTCCTTGTAACAAAGAAATATTAGAAGCCTTAGGAAATCAACCTA

IRN6|AB863141.1| AGCTCATCAATGATTGTCCTTGTAACAAAGAAATATTAGAAGCCTTAGGAAATCAACCTA

IRN7|AB863142.1| AGCTCATCAACAATTGTCCTTGTAACAAAGAAATATTAGAAGCCTTAGGAAATCAACCTA

IRN8|AB863143.1| AGCTCATCAACGATTGTCCTTGTAACAAAGAAATATTAGAAGCCTTAGGAAATCAACCTA

IRN9|AB863144.1| AGCTCATCACCGATTGTCCTTGTAACAAAGAAATATTAGAAGCCTTAGGAAATCAACCTA

IRN10|AB863145.1| AGCTCATCAACGATTGTCCTTGTAACAAAGAAATATTAGAAGCCTTAGGAAATCAACCTA

IRN11|AB863146.1| AGCTCATCAATGATTGTCCTTGTAACAAAGAAATATTAGAAGCCTTAGGCAACCAGCCTA

IRN12|AB863147.1| AGCTCATCAACGATTGTCCTTGTAACAAAGAAATATTAGAAGCCTTAGGAAATCAACCTA

IRN13|AB863148.1| AGCTCATCAATGATTGTCCTTGTAACAAAGAAATACTAGAGGCCTTAGGTAAGCAACCTA

IRN14|AB863149.1| AGCTCATCAATGATTGTCCTTGTAACAAAGAAATATTAGAAGCCTTAGGAAATCAACCTA

IRN15|AB863150.1| AGCTCATCAATGATTGTCCTTGTAACAAAGAAATACTAGAAGCCTTAGGTAAACAACCTA

IRN16|AB863151.1| AGCTCATCAATGATTGTCCTTGTAACAAAGAAATACTAGAAGCCTTAGGTAAACAACCTA

IRN17|AB863152.1| AGCTCATCAATGATTGTCCTTGTAACAAAGAAATACTAGAAGCCTTAGGTAAACAACCTA

IRN18|AB863153.1| AGCTCATCAATGATTGTCCTTGTAACAAAGAAATATTAGAAGCCTTAGGCAACCAGCCTA

IRN19|AB863154.1| AGCTCATCAATGATTGTCCCTGTAACAAAGAAATATTAGAAGCCTTAGGCAATCAGCCTA

IRN20|AB863155.1| AGCTCATCAATGATTGTCCTTGTAACAAAGAGATACTAGAGGCCTTAGGTAAACAACCTA

IRN21|AB863156.1| AGCTCATCAATGATTGTCCTTGTAACAAAGAAATATTAGAAGCCTTAGGAAATCAACCTA

JPNHGB340|AB863157.1| AGCTCATCAATGATTGTCCTTGTAACAAAGAGATATTAGAAGCCTTAGGCAATCAGCCTA

JPNKWB778|AB863158.1| AGCTCATCAATGATTGTCCTTGTAACAAAGAGATATTAGAAGCCTTAGGCAATCAGCCTA

JPNM|AB863159.1| AGCTCATCAATGATTGTCCTTGTAACAAAGAGATATTAGAGGCCTTAGGCAATCAGCCTA

JPNN|AB863160.1| AGCTCATCAATGATTGTCCTTGTAACAAAGAAATATTAGAAGCCTTAGGCAATCAACCTA

JPNS1|AB863161.1| AGATCATCAATGATTGTCCTTGTAACAAAGAAATATTAGAAGCCTTAGGCAATCAACCTA

JPNS2|AB863162.1| AGATCATCAATGATTGTCCTTGTAACAAAGAAATATTAGAAGCCTTAGGCAATCAACCTA

JPNTKD762|AB863163.1| AGCTCATCAATGATTGTCCTTGTAACAAAGAAATATTAGAGGCCTTGGGCAATCAGCCCA

JPNUV1|AB863164.1| AGCTCATCAATGATTGTCCTTGTAACAAAGAGATATTAGAGGCCTTAGGCAATCAGCCTA

JPNUV26|AB863165.1| AGCTCATCAATGATTGTCCTTGTAACAAAGAGATATTAGAGGCCTTAGGCAATCAGCCTA

TUR1|AB863166.1| AGCTCATCAATGATTGTCCTTGTAACAAAGAAATACTAGAAGCCTTAGGTAAACAACCTA

TUR2|AB863167.1| AGCTCATCAATGATTGTCCTTGTAACAAAGAAATATTAGAAGCCCTAGGTAACCAACCTA

TUR4|AB863168.1| AGCTCATCAATGATTGTCCTTGTAACAAAGAAATATTAGAAGCCCTAGGTAACCAACCTA

TUR5|AB863169.1| AGCTCATCAATGATTGTCCTTGTAACAAAGAAATATTAGAAGCCTTAGGCAACCAACCTA

TUR12|AB863170.1| AGCTCATCAATGATTGTCCTTGTAACAAAGAAATATTAGAAGCCTTAGGCAACCAGCCTA

TUR34|AB863171.1| AGCTCATCAATGATTGTCCTTGTAACAAAGAAATATTAGAAGCCCTAGGTAACCAACCTA

TUR50|AB863172.1| AGCTCATCAATGATTGTCCTTGTAACAAAGAAATATTAGAAGCCCTAGGTAACCAACCTA

TUR59|AB863173.1| AGCTCATCAATGATTGTCCTTGTAACAAAGAAATACTAGAGGCCTTAGGCAACCAACCTA

TUR69|AB863174.1| AGCTCATCAATGATTGTCCTTGTAACAAAGAAATATTAGAAGCCTTAGGCAACCAACCTA

TUR81|AB863175.1| AGCTCATCAATGATTGTCCTTGTAACAAAGAAATATTAGAAGCCTTAGGCAACCAACCTA

TUR84|AB863176.1| AGCTCATCAATGATTGTCCTTGTAACAAAGAAATATTAGAAGCCTTAGGCAACCAACCTA

TUR94|AB863177.1| AGCTCATCAATGATTGTCCTTGTAACAAAGAAATATTAGAAGCCTTAGGCAATCAGCCTA

TUR213|AB863178.1| AGCTCATCAATGATTGTCCTTGTAACAAAGAAATAATAGAAGCCCTAGGTAACCAACCTA

TUR214|AB863179.1| AGCTCATCAATGATTGTCCTTGTAACAAAGAGATACTAGAGGCCTTAGGTAAACAACCTA

TUR216|AB863180.1| AGCTCATCAATGATTGTCCTTGTAACAAAGAAATATTAGAAGCCCTAGGTAACCAACCTA

TUR220|AB863181.1| AGCTCATCAATGATTGTCCTTGTAACAAAGAAATATTAGAAGCCTTAGGCAACCAACCTA

TUR239|AB863182.1| AGCTCATTAATGATTGTCCTTGTAACAAAGAGATATTAGAAGCCTTAGGCAACCAACCTA

TUR244|AB863183.1| AGCTCATCAATGATTGTCCTTGTAACAAAGAAATATTAGAAGCCCTAGGTAACCAACCTA

TUR249|AB863184.1| AGCTCATCAATGATTGTCCTTGTAACAAAGAAATAATAGAAGCCCTAGGTAACCAACCTA

TUR263|AB863185.1| AGCTCATCAATGATTGTCCTTGTAACAAAGAGATATTAGAAGCCTTAGGCAACCAGCCTA

TUR278|AB863186.1| AGCTCATCAATGATTGTCCTTGTAACAAAGAAATATTAGAAGCCTTAGGCAACCAACCTA

TUR279|AB863187.1| AGCTCATCAATGATTGTCCTTGTAACAAAGAAATATTAGAAGCCTTAGGCAACCAACCTA

TUR285|AB863188.1| AGCTCATCAATGATTGTCCTTGTAACAAAGAAATACTAGAAGCCTTAGGTAAACAACCTA

TUR289|AB863189.1| AGCTCATCAATGATTGTCCTTGTAACAAAGAAATACTAGAAGCCTTAGGTAAACAACCTA

TUR303|AB863190.1| AGCTCATCAATGATTGTCCTTGTAACAAAGAAATATTAGAAGCCCTAGGTAACCAACCTA

TUR306|AB863191.1| AGCTCATCAATGATTGTCCTTGTAACAAAGAAATATTAGAAGCCCTAGGTAACCAACCTA

Cabb-S|NC_001497.1| AAGAGCAACTAATAGAACAACCTAAAGAAAAAGGTAAAGGCCTTAACTTAGGAAAATACT

CRO180A|AB863192.1| AAGAGCAACTAATAGAACAACCTAAAGAAAAAGGCAAAGGCCTTAACTTGGGAAAATACT

GRC83|AB863193.1| ATGAGCAACTAATAGAACAACCTAAAGAAAAAGGCAAAGGCCTTAATCTTGGAAAATACT

GRC84B|AB863194.1| AAGAGCAACTAATAGAACAACCTAAAGAAAAAGGCAAAGGCCTTAATCTTGGAAAATACT

GRC86B|AB863195.1| AAGGGCAACTAATAGAACAACCTAAAGAAAAAGGCAAAGGCCTTAATCTTGGAAAATACT

GRC86D|AB863196.1| AAGAGCAACTAATAGAACAACCTAAAGAAAAAGGCAAAGGCCTTAATCTTGGAAAATACT

GRC87E|AB863197.1| ATGAGCAACTAATAGAACAACCTAAAGAAAAAGGCAAAGGCCTTAATCTTGGAAAATACT

GRC87G|AB863198.1| ATGAGCAACTAATAGAACAACCTAAAGAAAAAGGCAAAGGCCTTAATCTTGGAAAATACT

GRC91B|AB863199.1| AAGAGCAACTAATAGAACAACCTAAAGAAAAAGGCAAAGGCCTTAATCTTGGAAAATACT

GRC92A|AB863200.1| AAGAGCAACTAATAGAACAACCCAAAGAAAAAGGCAAAGGCCTTAATCTTGGAAAATACT

GRC92C|AB863201.1| AAGAGCAACTAATAGAACAACCCAAAGAAAAAGACAAAGGCCTTAATTTTGGAAAATACT

GRC92D|AB863202.1| AAGAGCAACTAATAGAACAACCCAAAGAAAAAGGCAAAGGCCTTAATCTTGGAAAATACT

IRN1|AB863136.1| AAGAGCAACTAATAGAACAACCTAAAGAAAAAGGCAAAGGCCTTAATCTAGGAAAATACT

IRN2|AB863137.1| AAGAGCAACTAATAGAACAACCTAAAGAAAAAGGCAAAGGCCTTAATCTAGGAAAATACT

IRN3|AB863138.1| AAGAGCAACTAATAGAACAACCTAAAGAAAAAGGCAAAGGCCTTAATCTAGGAAAATACT

IRN4|AB863139.1| AAGAGCAACTAATAGAACAACCTAAAGAAAAAGGCAAAGGCTTTAATCTAGGAAAATACT

IRN5|AB863140.1| AAGAGCAACTAATAGAACAACCTAAAGAAAAAGGCAAAGGCCTTAATCTAGGAAAATACT

IRN6|AB863141.1| AAGAGCAACTAATAGAACAACCTAAAGAAAAAGGCAAAGGCCTTAATCTAGGAAAATACT

IRN7|AB863142.1| AAGAGCAACTAATAGAACAACCTAAAGAAAAAGGCAAAGGCCTTAATCTAGGAAAATACT

IRN8|AB863143.1| AAGAGCAACTAATAGAACAACCTAAAGAAAAAGGCAAAGGCCTTAATCTAGGAAAATACT

IRN9|AB863144.1| AAGAGCAACTAATAGAACAACCTAAAGAAAAAGGCAAAGGCCTTAACTTAGGAAAATACT

IRN10|AB863145.1| AAGAGCAACTAATAGAACAACCTAAAGAAAAAGGCAAAGGCCTTAATCTAGGAAAATACT

IRN11|AB863146.1| AAGAGCAACTAATAGAACAACCTAAAGAAAAAGGCAAAGGCCTTAATCTTGGAAAATACT

IRN12|AB863147.1| AAGAGCAACTAATAGAACAACCTAAAGAAAAAGGCAAAGGCCTTAATCTAGGAAAATACT

IRN13|AB863148.1| AAGACCAGCTAGTAGAACAACCTAAAGAAAAAGGCAAAGGCCTTAACTTAGGAAAATACT

IRN14|AB863149.1| AAGAGCAACTAATAGAACAACCTAAAGAAAAAGGCAAAAGCCTTAATCTAGGAAAATACT

IRN15|AB863150.1| AAGACCAACTAGTAGAACAACCTAAAGAAAAAGGCAAAGGCCTTAATCTTGGAAAATACT

IRN16|AB863151.1| AAGACCAACTAGTAGAACAACCTAAAGAAAAAGGCAAAGGCCTTAATCTTGGAAAATACT

IRN17|AB863152.1| AAGACCAACTAGTAGAACAACCTAAAGAAAAAGGCAAAGGCCTTAATCTTGGAAAATACT

IRN18|AB863153.1| AAGAGCAACTAATAGAACAACCTAAAGAAAAAGGCAAAGGCCTTAATCTTGGAAAATACT

IRN19|AB863154.1| AAGAGCAACTAATAGAACAACCTAAAGAAAAAGGCAAAGGCCTTAATCTTGGAAAATACT

IRN20|AB863155.1| AAGACCAACTAGTAGAACAACCTAAAGAAAAAGGCAAAGGCCTTAATCTTGGAAAATACT

IRN21|AB863156.1| AAGAGCAACTAATAGAACAACCTAAAGAAAAAGGCAAAGGCCTTAATCTAGGAAAATACT

JPNHGB340|AB863157.1| AAGAGCAACTAATAGAACAACCTAAAGAAAAAGGCAAAGGCCTTAATCTAGGAAAATACT

JPNKWB778|AB863158.1| AAGAGCAACTAATAGAACAACCTAAAGAAAAAGGCAAAGGCCTTAATCTAAGAAAATACT

JPNM|AB863159.1| AAGAGCAACTAATAGAACAACCTAAAGAAAAAGGCAAAGGCCTTAATCTAGGAAAATACT

JPNN|AB863160.1| AAGAGCAACTAATAGAACAACCTAAAGAAAAAGGCAAAGGCCTTAATCTAGGAAAATACT

JPNS1|AB863161.1| AAGAGCAACTAATAGAACAACCTAAAGAAAAAGGCAAAGGCCTTAATCTAGGAAAATATT

JPNS2|AB863162.1| AAGAGCAACTAATAGAACAACCTAAAGAAAAAGGCAAAGGCCTTAATCTAGGAAAATATT

JPNTKD762|AB863163.1| AAGAGCAACTAATAGAACAACCTAAAGAAAAAGGCAAAGGCCTTAATCTAGGAAAATACT

JPNUV1|AB863164.1| AAGAGCAACTAATAGAACAACCTAAAGAAAAAGGCAAAGGCCTTAATCTAGGAAAATACT

JPNUV26|AB863165.1| AAGAGCAACTAATAGAACAACCTAAAGAAAAAGGCAAAGGCCTTAATCTAGGAAAATACT

TUR1|AB863166.1| AAGACCAACTAGTAGAACAACCTAAAGAAAAAGGCAAAGGCCTTAACTTAGGAAAATACT

TUR2|AB863167.1| AAGAGCAACTAATAGAACAACCTAAAGAAAAAGGCAAAGGCCTTAACTTGGGAAAATACT

TUR4|AB863168.1| AAGAGCAACTAATAGAACAACCTAAAGAAAAAGGCAAAGGCCTTAACTTGGGAAAATACT

TUR5|AB863169.1| AAGAGCAACTAATAGAACAACCTAAAGAAAAAGGCAAAGGCCTTAATCTTGGAAAATACT

TUR12|AB863170.1| ATGAGCAACTAATAGAACAACCTAAAGAAAAAGGCAAAGGTCTTAACTTAGGAAAATACT

TUR34|AB863171.1| AAGAGCAACTAATAGAACAACCTAAAGAAAAAGGCAAAGGCCTTAACTTAGGAAAATACT

TUR50|AB863172.1| AAGAGCAACTAATAGAACAACCTAAAGAAAAAGGCAAAGGCCTTAACTTAGGAAAATACT

TUR59|AB863173.1| AAGACCAACTAGTAGAACAACCTAAAGAAAAAGGCAAAGGCCTTAATCTTGGAAAATACT

TUR69|AB863174.1| AAGAGCAACTAATAGAACAACCTAAAGAAAAAGGCAAAGGTCTTAATCTTGGAAAATACT

TUR81|AB863175.1| AAGAGCAACTAATAGAACAACCTAAAGAAAAAGGCAAAGGCCTTAATCTTGGAAAATACT

TUR84|AB863176.1| AAGAGCAACTAATAGAACAACCTAAAGAAAAAGGCAAAGGCCTTAATCTTGGAAAATACT

TUR94|AB863177.1| AAGAGCAACTAATAGAACAACCTAAAGAAAAAGGCAAAGGCCTTAATCTTGGAAAATACT

TUR213|AB863178.1| AAGAGCAACTAATAGAACAACCTAAAGAAAAAGGCAAAGGCCTTAACTTGGGAAAATACT

TUR214|AB863179.1| AAGACCAACTAGTAGAACAACCTAAAGAAAAAGGCAAAGGCCTTAACTTAGGAAAATACT

TUR216|AB863180.1| AAGAGCAACTAATAGAACAACCTAAAGAAAAAGGCAAAGGCCTTAACTTGGGAAAATACT

TUR220|AB863181.1| AAGAGCAACTAATAGAACAACCTAAAGAAAAAGGCAAAGGCCTTAATCTTGGAAAATACT

TUR239|AB863182.1| AAGAGCAACTAATAGAACAACCTAAAGAAAAAGGCAAAGGCCTTAATCTTGGAAAATACT

TUR244|AB863183.1| AAGAGCAACTAATAGAACAACCTAAAGAAAAAGGCAAAGGCCTTAACTTGGGAAAATACT

TUR249|AB863184.1| AAGAGCAACTAATAGAACAACCTAAAGAAAAAGGCAAAGGCCTTAACTTGGGAAAATACT

TUR263|AB863185.1| AAGTGCAACTAATAGAACAACCCAAAGAAAAAGGCAAAGGCCTTAATCTTGGAAAATACT

TUR278|AB863186.1| AAGAGCAACTAATAGAACAACCTAAAGAAAAAGGCAAAGGCCTTAATCTTGGAAAATACT

TUR279|AB863187.1| AAGAGCAACTAATAGAACAACCTAAAGAAAAAGGCAAAGGCCTTAATCTTGGAAAATACT

TUR285|AB863188.1| AAGACCAACTAGTAGAACAACCTAAAGAAAAAGGCAAAGGCCTTAACTTAGGAAAATACT

TUR289|AB863189.1| AAGACCAACTAGTAGAACAACCTAAAGAAAAAGGCAAAGGCCTTAACTTAGGAAAATACT

TUR303|AB863190.1| AAGAGCAACTAATAGAACAACCTAAAGAAAAAGGCAAAGGCCTTAATCTTGGAAAATACT

TUR306|AB863191.1| AAGAGCAACTAATAGAACAACCTGAAGAAAAAGGCAAAGGCCTTAATCTTGGAAAATACT

Cabb-S|NC_001497.1| CTTACCCCAATTACGGAGTAGGAAATGAAGAATTAGGATCCTCTGGAAACCCTAAAGCTT

CRO180A|AB863192.1| CTTACCCCAATTACGGAGTAGGAAATGAAGAATTAGGATCCTCTGGAAACCCTAAAGCTT

GRC83|AB863193.1| CTTACCCCAACTACGGAGTAGGAAATGAAGAATTAGGATCCTCTGGAAACCCTAAAGCTT

GRC84B|AB863194.1| CTTACCCCAATTACGGAGTAGGAAATGAAGAATTAGGATCCTCTGGAAACCCTAAAGCTT

GRC86B|AB863195.1| CTTACCCCAATTACGGAGTAGGAAATGAAGAATTAGGATCCTCTGGAAACCCTAAAGCTT

GRC86D|AB863196.1| CTTACCCCAATTACGGAGTAGGAAATGAAGAATTAGGATCCTCTGGAAACCCTAAAGCTT

GRC87E|AB863197.1| CTTACCCCAACTACGGAGTAGGAAATGAAGAATTAGGATCCTCTGGAAACCCTAAAGCTT

GRC87G|AB863198.1| CTTACCCCAACTACGGAGTAGGAAATGAAGAATTAGGATCCTCTGGAAACCCTAAAGCTT

GRC91B|AB863199.1| CTTACCCCAATTACGGAGTAGGAAATGAAGAATTAGGATCCTCTGGAAACCCTAAAGCTT

GRC92A|AB863200.1| CTTACCCCAATTACGGAGTAGGAAATGAAGAATTAGGATCCTCTGGAAACCCTAAAGCTT

GRC92C|AB863201.1| CTTACCCCAATTACGGAGTAGGAAATGAAGAATTAGGATCCTCTGGAAACCCTAAAGCTT

GRC92D|AB863202.1| CTTACCCCAATTACGGAGTAGGAAATGAAGAATTAGGATCCTCTGGAAACCCTAAAGCTT

IRN1|AB863136.1| CTTACCCCAATTACGGAGTAGGAAATGAAGAATTAGGATCCTCTGGAAACCCTAAAGCTT

IRN2|AB863137.1| CTTACCCCAATTACGGTGTAGGAAATGAAGAATTAGGATCCTCTGGAAACCCTAAAGCTT

IRN3|AB863138.1| CTTACCCCAATTACGGAGTAGGAAATGAAGAATTAGGATCCTCTGGAAACCCTAAAGCTT

IRN4|AB863139.1| CTTACCCCAATTACGGAGTAGGAAATGAAGAATTAGGATCCTCTGGAAACCCTAAAGCTT

IRN5|AB863140.1| CTTACCCCAATTACGGTGTAGGAAATGAAGAATTAGGATCCTCTGGAAACCCTAAAGCTT

IRN6|AB863141.1| CTTACCCCAATTACGGTGTAGGAAATGAAGAATTAGGATCCTCTGGAAACCCTAAAGCTT

IRN7|AB863142.1| CTTACCCCAATTACGGTGTAGGAAATGAAGAATTAGGATCCTCTGGAAACCCTAAAGCTT

IRN8|AB863143.1| CTTACCCCAATTACGGTGTAGGAAATGAAGAATTAGGATCCTCTGGAAACCCTAAAGCTT

IRN9|AB863144.1| CTTACCCCAATTACGGAGTAGGAAATGAAGAATTAGGATCCTCTGGAAACCCTAAAGCTT

IRN10|AB863145.1| CTTACCCCAATTACGGTGTAGGAAATGAAGAATTAGGATCCTCTGGAAACCCTAAAGCTT

IRN11|AB863146.1| CTTACCCCAATTACGGAGTAGGAAATGAAGAATTAGGATCCTCTGGAAACCCTAAAGCTT

IRN12|AB863147.1| CTTACCCCAATTACGGAGTAGGAAATGAAGAATTAGGATCCTCTGGAAACCCTAAAGCTT

IRN13|AB863148.1| CTTACCCCAATTACGGAGTAGGAAATGAAGAATTAGGATCCTCTGGAAACCCTAAAGCTT

IRN14|AB863149.1| CTTACCCCAATTACGGTGTAGGAAATGAAGAATTAGGATCCTCTGGAAACCCTAAAGCTT

IRN15|AB863150.1| CTTACCCCAATTACGGAGTAGGAAATGAAGAATTAGGATCCTCTGGAAACCCTAAAGCTT

IRN16|AB863151.1| CTTACCCCAATTACGGAGTAGGAAATGAAGAATTAGGATCCTCTGGAAACCCTAAAGCTT

IRN17|AB863152.1| CTTACCCCAATTACGGAGTAGGAAATGAAGAATTAGGATCCTCTGGAAACCCTAAAGCTT

IRN18|AB863153.1| CTTACCCCAATTACGGAGTAGGAAATGAAGAATTAGGATCCTCTGGAAACCCTAAAGCTT

IRN19|AB863154.1| CTTACCCCAATTACGGAGTAGGAAATGAAGAATTAGGATCCTCTGGAAACCCTAAAGCTT

IRN20|AB863155.1| CTTACCCCAATTACGGAGTAGGAAATGAAGAATTAGGATCCTCTGGAAACCCTAAAGCTT

IRN21|AB863156.1| CTTACCCCAATTACGGTGTAGGAAATGAAGAATTAGGATCCTCTGGAAACCCTAAAGCTT

JPNHGB340|AB863157.1| CTTACCCCAATTACGGAGTAGGAAATGAAGAATTAGGATCCTCTGGAAACCCTAAAGCTT

JPNKWB778|AB863158.1| CTTACCCCAATTACGGAGTAGGAAATGAAGAATTAGGATCCTCTGGAAACCCTAAAGCTT

JPNM|AB863159.1| CTTACCCCAATTACGGAGTAGGAAATGAAGAATTAGGATCCTCTGGAAACCCTAAAGCTT

JPNN|AB863160.1| CTTACCCCAATTACGGCGTAGGAAATGAAGAATTAGGATCCTCTGGAAACCCTAAAGCTT

JPNS1|AB863161.1| CTTACCCCAATTACGGCGTAGGAAATGAAGAATTAGGATCCTCTGGAAACCCTAAAGCTT

JPNS2|AB863162.1| CTTACCCCAATTACGGCGTAGGAAATGAAGAATTAGGATCCTCTGGAAACCCTAAAGCTT

JPNTKD762|AB863163.1| CTTACCCCAATTACGGAGTAGGAAATGAAGAATTAGGATCCTCTGGAAACCCTAAAGCTT

JPNUV1|AB863164.1| CTTACCCCAATTACGGAGTAGGAAATGAAGAATTAGGATCCTCTGGAAACCCTAAAGCTT

JPNUV26|AB863165.1| CTTACCCCAATTACGGAGTAGGAAATGAAGAATTAGGATCCTCTGGAAACCCTAAAGCTT

TUR1|AB863166.1| CTTACCCCAATTACGGAGTAGGAAATGAAGAATTAGGATCCTCTGGAAACCCTAAAGCTT

TUR2|AB863167.1| CTTACCCCAATTACGGAGTAGGAAATGAAGAATTAGGATCCTCTGGAAACCCTAAAGCTT

TUR4|AB863168.1| CTTACCCCAATTACGGAGTAGGAAATGAAGAATTAGGATCCTCTGGAAACCCTAAAGCTT

TUR5|AB863169.1| CTTACCCCAATTACGGAGTAGGAAATGAAGAATTAGGATCCTCTGGAAACCCTAAAGCTT

TUR12|AB863170.1| CTTACCCCAATTACGGAGTAGGAAATGAAGAATTAGGATCCTCTGGAAACCCTAAAGCTT

TUR34|AB863171.1| CTTACCCCAATTACGGAGTAGGAAATGAAGAATTAGGATCCTCTGGAAACCCTAAAGCTT

TUR50|AB863172.1| CTTACCCCAATTACGGAGTAGGAAATGAAGAATTAGGATCCTCTGGAAACCCTAAAGCTT

TUR59|AB863173.1| CTTACCCCAATTACGGAGTAGGAAATGAAGAATTAGGATCCTCTGGAAACCCTAAAGCTT

TUR69|AB863174.1| CTTACCCCAATTACGGAGTAGGAAATGAAGAATTAGGATCCTCTGGAAACCCTAAAGCTT

TUR81|AB863175.1| CTTACCCCAATTACGGAGTAGGAAATGAAGAATTAGGATCCTCTGGAAACCCTAAAGCTT

TUR84|AB863176.1| CTTACCCCAATTACGGAGTAGGAAATGAAGAATTAGGATCCTCTGGAAACCCTAAAGCTT

TUR94|AB863177.1| CTTACCCCAATTACGGAGTAGGAAATGAAGAATTAGGATCCTCTGGAAACCCTAAAGCTT

TUR213|AB863178.1| CTTACCCCAATTACGGAGTAGGAAATGAAGAATTAGGATCCTCTGGAAACCCTAAAGCTT

TUR214|AB863179.1| CTTACCCCAATTACGGAGTAGGAAATGAAGAATTAGGATCCTCTGGAAACCCTAAAGCTT

TUR216|AB863180.1| CTTACCCCAATTACAGAGTAGGAAATGAAGAATTAGGATCCTCTGGAAACCCTAAAGCTT

TUR220|AB863181.1| CTTACCCCAATTACGGAGTAGGAAATGAAGAATTAGGATCCTCTGGAAACCCTAAAGCTT

TUR239|AB863182.1| CTTACCCCAATTACGGAGTAGGAAATGAAGAATTAGGATCCTCTGGAAACCCTAAAGCTT

TUR244|AB863183.1| CTTACCCCAATTACGGAGTAGGAAATGAAGAATTAGGATCCTCTGGAAACCCTAAAGCTT

TUR249|AB863184.1| CTTACCCCAATTACGGAGTAGGAAATGAAGAATTAGGATCCTCTGGAAACCCTAAAGCTT

TUR263|AB863185.1| CTTACCCCAATTACGGAGTAGGAAATGAAGAATTAGGATCCTCTGGAAACCCTAAAGCTT

TUR278|AB863186.1| CTTACCCCAATTACGGAGTAGGAAATGAAGAATTAGGATCCTCTGGAAACCCTAAAGCTT

TUR279|AB863187.1| CTTACCCCAATTACGGAGTAGGAAATGAAGAATTAGGATCCTCTGGAAACCCTAAAGCTT

TUR285|AB863188.1| CTTACCCCAATTACGGAGTAGGAAATGAAGAATTAGGATCCTCTGGAAACCCTAAAGCTT

TUR289|AB863189.1| CTTACCCTAATTACGGAGTAGGAAATGAAGAATTAGGATCCTCTGGAAACCCTAAAGCTT

TUR303|AB863190.1| CTTACCCCAATTACGGAGTAGGAAATGAAGAATTAGGATCCTCTGGAAACCCTAAAGCTT

TUR306|AB863191.1| CTTACCCCAATTACGGAGTAGGAAATGAAGAATTAGGATCCTCTGGAAACCCTAAAGCTT

Cabb-S|NC_001497.1| TAACCTGGCCCTTCAAAGCTCCAGCAGGATGGCCGAATCAATTTTAGACAGAACCATTAA

CRO180A|AB863192.1| TAACCTGGCCCTTCAAAGCTCCAGCAGGATGGCCGAATCAATATTAGACCGAACTATTAA

GRC83|AB863193.1| TAACCTGGCCCTTCAAAGCTCCAGCAGGATGGCCGAATCAATTTTAGACCGAACTATTAA

GRC84B|AB863194.1| TAACCTGGCCCTTCAAAGCTCCAGCAGGATGGCCGAATCAATTTTAGACCGAACCATTAA

GRC86B|AB863195.1| TAACCTGGCCCTTCAAAGCTCCAGCAGGATGGCCGAATCAATTTTAGACCGAACCATTAA

GRC86D|AB863196.1| TAACCTGGCCCTTCAAAGCTCCAGCAGGATGGCCGAATCAATTTTAGACCGAACTATTAA

GRC87E|AB863197.1| TAACCTGGCCCTTCAAAGCTCCAGCAGGATGGCCGAATCAATTTTAGACCGAACTATTAA

GRC87G|AB863198.1| TAACCTGGCCCTTCAAAGCTCCAGCAGGATGGCCGAATCAATTTTAGACCGAACTATTAA

GRC91B|AB863199.1| TAACCTGGCCCTTCAAAGCTCCAGCAGGATGGCCGAATCAATTTTAGACCGAACTATTAA

GRC92A|AB863200.1| TAACCTGGCCCTTCAAAGCTCCAGCAGGATGGCCGAATCAATTTTAGACCGAACTATTAA

GRC92C|AB863201.1| TAACCTGGCCCTTCAAAGCTCCAGCAGGATGGCCGAATCAATTTTAGACCGAACTATTAA

GRC92D|AB863202.1| TAACCTGGCCCTTCAAAGCTCCAGCAGGATGGCCGAATCAATTTTAGACCGAACTATTAA

IRN1|AB863136.1| TAACCTGGCCCTTCAAAGCTCCAGCAGGATGGCCGAATCAATTTTAGACCGAACTATTAA

IRN2|AB863137.1| TAACCTGGCCCTTCAAAGCTCCAGCAGGATGGCCGAATCAATTTTAGACCGAACTATTAA

IRN3|AB863138.1| TAACCTGGCCCTTCAAAGCTCCAGCAGGATGGCCGAATCAATTTTAGACCGAACTATTAA

IRN4|AB863139.1| TAACCTGGCCCTTCAAAGCTCCAGCAGGATGGCCGAATCAATTTTAGACAGAACTATTAA

IRN5|AB863140.1| TAACCTGGCCCTTCAAAGCTCCAGCAGGATGGCCGAATCAATTTTAGACCGAACTATTAA

IRN6|AB863141.1| TAACCTGGCCCTTCAAAGCTCCAGCAGGATGGCCGAATCAATTTTAGACCGAACTATTAA

IRN7|AB863142.1| TAACCTGGCCCTTCAAAGCTCCAGCAGGATGGCCGAATCAATTTTAGACCGAACTATTAA

IRN8|AB863143.1| TAACCTGGCCCTTCAAAGCTCCAGCAGGATGGCCGAATCAATTTTAGACCGAACTATTAA

IRN9|AB863144.1| TAACCTGGCCCTTCAAAGCTCCAGCAGGATGGCCGAATCAATTTTAGACCGAACTATTAA

IRN10|AB863145.1| TAACCTGGCCCTTCAAAGCTCCAGCAGGATGGCCGAATCAATTTTAGACCGAACTATTAA

IRN11|AB863146.1| TAACCTGGCCCTTCAAAGCTCCAGCAGGATGGCCGAATCAATTTTAGACCGAACTATTAA

IRN12|AB863147.1| TAACCTGGCCCTTCAAAGCTCCAGCAGGATGGCCGAATCAATTTTAGACCGAACTATTAA

IRN13|AB863148.1| TAACCTGGCCCTTCAAAGCTCCAGCAGGATGGCCGAATCAATTTTAGACCGAACTATTAA

IRN14|AB863149.1| TAACCTGGCCCTTCAAAGCTCCAGCAGGATGGCCGAATCAATTTTAGACCGAACTATTAA

IRN15|AB863150.1| TAACCTGGCCCTTCAAAGCTCCAGCAAGATGGCCGAATCAATTTTAGACCGAACTATTAA

IRN16|AB863151.1| TAACCTGGCCCTTCAAAGCTCCAGCAGGATGGCCGAATCAATTTTAGACCGAACTATTAA

IRN17|AB863152.1| TAACCTGGCCCTTCAAAGCTCCAGCAGGATGGCCGAATCAATTTTAGACCGAACTATTAA

IRN18|AB863153.1| TAACCTGGCCCTTCAAAGCTCCAGCAGGATGGCCGAATCAATTTTAGACCGAACTATTAA

IRN19|AB863154.1| TAACCTGGCCCTTCAAAGCTCCAGCAGGATGGCCGAATCAATTTTAGACCGAACTATTAA

IRN20|AB863155.1| TAACCTGGCCCTTCAAAGCTCCAGCAGGATGGCCGAATCAGTTTTAGACCGAACTATTAA

IRN21|AB863156.1| TAACCTGGCCCTTCAAAGCTCCAGCAGGATGGCCGAATCAATTTTAGACCGAACTATTAA

JPNHGB340|AB863157.1| TAACTTGGCCCTTCAAAGCTCCAGCAGGATGGCCGAATCAATTTTAGACAGAACCATTAA

JPNKWB778|AB863158.1| TAACTTGGCCCTTCAAAGCTCCAGCAGGATGGCCGAATCAATTTTAGACAGAACCATTAA

JPNM|AB863159.1| TAACTTGGCCCTTCAAAGCTCCAGCAGGATGGCCGAATCAATTTTAGACAGAACCATTAA

JPNN|AB863160.1| TAACTTGGCCTTTCAAAGCACCAGAAGGATGGCCAAACCAATTCTAGACGAAACCATTAA

JPNS1|AB863161.1| TAACTTGGCCTTTCAAAGCACCAGAAGGATGGCCGAATCAATTTTAGATAAAACCATTAA

JPNS2|AB863162.1| TAACTTGGCCTTTCAAAGCACCAGAAGGATGGCCGAATCAATTTTAGATAAAACCATTAA

JPNTKD762|AB863163.1| TAACTTGGCCCTTCAAAGCTCCAGCAGGATGGCCGAATCAATTTTAGACAGAACCATTAA

JPNUV1|AB863164.1| TAACTTGGCCCTTCAAAGCTCCAGCAGGATGGCCGAATCAATTTTAGACAGAACCATTAA

JPNUV26|AB863165.1| TAACTTGGCCCTTCAAAGCTCCAGCAGGATGGCCGAATCAATTTTAGACAGAACCATTAA

TUR1|AB863166.1| TAACCTGGCCCTTCAAAGCTCCAGCAGGATGGCCGAATCAATTTTAGACAGAACCATTAA

TUR2|AB863167.1| TAACTTGGCCCTTCAAAGCTCCAGCAGGATGGCCGAATCAATTTTAGACAGAACCATTAA

TUR4|AB863168.1| TAACTTGGCCCTTCAAAGCTCCAGCAGGATGGCCGAATCAATTTTAGACAGAACCATTAA

TUR5|AB863169.1| TAACCTGGCCCTTCAAAGCTCCAGCAGGATGGCCGAATCAATTTTAGACCGAACTATTAA

TUR12|AB863170.1| TAACCTGGCCCTTCAAAGCTCCAGCAGGATGGCCGAATCAATTTTAGACCGAACTATTAA

TUR34|AB863171.1| TAACCTGGCCCTTCAAAGCTCCAGCAGGATGGCCGAATCAATTTTAGACCGAACTATTAA

TUR50|AB863172.1| TAACCTGGCCCTTCAAAGCTCCAGCAGGATGGCCGAATCAATTTTAGACCGAACTATTAA

TUR59|AB863173.1| TAACCTGGCCCTTCAAAGCTCCAGCAGGATGGCCGAATCAATTTTAGACCGGACCATTAA

TUR69|AB863174.1| TAACCTGGCCCTTCAAAGCTCCAGCAGGATGGCCGAATCAATTTTAGACAGAACCATTAA

TUR81|AB863175.1| TAACCTGGCCCTTCAAAGCTCCAGCAAGATGGCCGAATCAATTTTAGACAGAACCATTAA

TUR84|AB863176.1| TAACCTGGCCCTTCAAAGCTCCAGCAGGATGGCCGAATCAATTTTAGACAGAACCATTAA

TUR94|AB863177.1| TAACCTGGCCCTTCAAAGCTCCAGCAGGATGGCCGAATCAATTTTAGACCGAACTATTAA

TUR213|AB863178.1| TAACCTGGCCCTTCAAAGCTCCAGCAGGATGGCCGAATCAATTTTAGACAGAACCATTAA

TUR214|AB863179.1| TAACCTGGCCCTTCAAAGCTCCAGCAGGATGGCCGAATCAATTTTAGACCGAACTATTAA

TUR216|AB863180.1| TAACCTGGCCCTTCAAAGCTCCAGCAGGATGGCCGAATCAATTTTAGACAGAACCATTAA

TUR220|AB863181.1| TAACCTGGCCCTTCAAAGCTCCAGCAGGATGGCCGAATCAATTTTAGACAGAACCATTAA

TUR239|AB863182.1| TAACCTGGCCCTTCAAAGCTCCAGCAGGATGGCCGAATCAATTTTAGACAGAACCATTAA

TUR244|AB863183.1| TAACCTGGCCCTTCAAAGCTCCAGCAGGATGGCCGAATCAATTTTAGACAGAACCATTAA

TUR249|AB863184.1| TAACCTGGCCCTTCAAAGCTCCAGCAGGATGGCCGAATCAATTTTAGACAGAACCATTAA

TUR263|AB863185.1| TAACCTGGCCCTTCAAAGCTCCAGCAGGATGGCCGAATCAATTTTAAACCGAACTATTAA

TUR278|AB863186.1| TAACCTGGCCCTTCAAAGCTCCAGCAGGATGGCCGAATCAATTTTAGACAGAACCATTAA

TUR279|AB863187.1| TAACCTGGCCCTTCAAAGCTCCAGCAGGATGGCCGAATCAATTTTAGACAGAACCATTAA

TUR285|AB863188.1| TAACCTGGCCCTTCAAAGCTCCAGCAGGATGGCCGAATCAATTTTAGACCGAACTATTAA

TUR289|AB863189.1| TAACCTGGCCCTTCAAAGCTCCAGCAGGATGGCCGAATCAATTTTAGACAGAACCATTAA

TUR303|AB863190.1| TAACCTGGCCCTTCAAAGCTCCAGCAGGATGGCCGAATCAATTTTAGACAGAACCATTAA

TUR306|AB863191.1| TAACCTGGCCCTTCAAAGCTCCAGCAGGATGGCCGAATCAATTTTAGACAGAACCATTAA

Cabb-S|NC_001497.1| TAGGTTTTGGTATAATCTGGGAGAAGATTGTCTCTCAGAAAGTCAATTCGATCTTATGAT

CRO180A|AB863192.1| TAGGTTCTGGTATAAACTGGGAGATGATTGTCTCTCAGAAAGTCAATTTGACCTTATGAT

GRC83|AB863193.1| TAGGTTCTGGTATAAACTGGGAGATGATTGTCTCTCAGAAAGTCAATTTGACCTTATGAT

GRC84B|AB863194.1| TAGGTTCTGGTATAAACTGGGAGATGATTGTCTCTCAGAAAGTCAATTTGACCTTATGAT

GRC86B|AB863195.1| TAGGTTCTGGTATAAACTGGGAGATGATTGTCTCTCAGAAAGTCAATTTGACCTTATGAT

GRC86D|AB863196.1| TAGGTTCTGGTATAAACTGGGAGATGATTGTCTCTCAGAAAGTCAATTTGACCTTATGAT

GRC87E|AB863197.1| TAGGTTCTGGTATAAACTGGGAGATGATTGTCTCTCAGAAAGTCAATTTGACCTTATGAT

GRC87G|AB863198.1| TAGGTTCTGGTATAAACTGGGAGATGATTGTCTCTCAGAAAGTCAATTTGACCTTATGAT

GRC91B|AB863199.1| TAGGTTCTGGTATAAACTGGGAGATGATTGTCTCTCAGAAAGTCAATTTGACCTTATGAT

GRC92A|AB863200.1| TAGGTTCTGGTATAAACTGGGAGATGATTGTCTCTCAGAAAGTCAATTTGACCTTATGAT

GRC92C|AB863201.1| TAGGTTCTGGTATAAACTGGGAGATGATTGTCTCTCAGAAAGTCAATTTGACCTTATGAT

GRC92D|AB863202.1| CAGGTTCTGGTATAAACTGGGAGATGATTGTCTCTCAGAAAGTCAATTTGACCTTATGAT

IRN1|AB863136.1| TAGGTTCTGGTATAAACTGGGAGATGATTGTCTCTCAGAAAGTCAATTTGACCTTATGAT

IRN2|AB863137.1| TAGGTTCTGGTATAAACTGGGAGATGATTGTCTCTCAGAAAGTCAATTTGACCTTATGAT

IRN3|AB863138.1| TAGGTTCTGGTATAAACTGGGAGATGATTGTCTCTCAGAAAGTCAATTTGACCTTATGAT

IRN4|AB863139.1| TAGGTTCTGGTATAAACTGGGAGATAACTGTCTCTCAGAAAGTCAATTTGACCTTATGAT

IRN5|AB863140.1| TAGGTTCTGGTATAAACTGGGAGATGATTGTCTCTCAGAAAGTCAATTTGACCTTATGAT

IRN6|AB863141.1| TAGGTTCTGGTATAAACTGGGAGACGATTGTCTCTCAGAAAGTCAATTTGACCTTATGAT

IRN7|AB863142.1| TAGGTTCTGGTATAAACTGGGAGATGACTGTCTCTCAGAAAGTCAATTTGACCTTATGAT

IRN8|AB863143.1| TAGGTTCTGGTATAAACTGGGAGATGATTGTCTCTCAGAAAGTCAATTTGACCTTATGAT

IRN9|AB863144.1| TAGGTTCTGGTATAAACTGGGAGATGATTGTCTCTCAGAAAGTCAATTTGACCTTATGAT

IRN10|AB863145.1| TAGGTTCTGGTATAAACTGGGAGATGATTGTCTCTCAGAAAGTCAATTTGACCTTATGAT

IRN11|AB863146.1| TAGGTTCTGGTATAAACTGGGAGACGATTGCCTCTCAGAAAGTCAATTTGACCTTATGAT

IRN12|AB863147.1| TAGGTTCTGGTATAAACTGGGAGATGATTGTCTCTCAGAAAGTCAATTTGACCTTATGAT

IRN13|AB863148.1| TAGGTTCTGGTATAAACTGGGAGATGATTGTCTCTCAGAAAGTCAATTTGACCTTATGAT

IRN14|AB863149.1| TAGGTTCTGGTATAAACTGGGAGACGATTGTCTCTCAGAAAGTCAATTTGACCTTATGAT

IRN15|AB863150.1| TAGGTTCTGGTATAAACTGGGAGATGATTGTCTCTCAGAAAGTCAATTTGACCTTATGAT

IRN16|AB863151.1| TAGGTTCTGGTATAAACTGGGAGATGATTGTCTCTCAGAAAGTCAATTTGACCTTATGAT

IRN17|AB863152.1| TAGGTTCTGGTATAAACTGGGAGATGATTGTCTCTCAGAAAGTCAATTTGACCTTATGAT

IRN18|AB863153.1| TAGGTTCTGGTATAAACTGGGAGATGATTGTCTCTCAGAAAGTCAATTTGACCTCATGAT

IRN19|AB863154.1| TAGGTTCTGGTATAAACTGGGAGACGACTGTCTTTCAGAAAGTCAATTTGACCTTATGAT

IRN20|AB863155.1| TAGGTTCTGGTATAAACTGGGAGATGATTGTCTCTCAGAAAGTCAATTTGACCTTATGAT

IRN21|AB863156.1| TAGGTTCTGGTATAAACTGGGAGACGATTGTCTCTCAGAAAGTCAATTTGACCTTATGAT

JPNHGB340|AB863157.1| CAGGTTTTGGTATAATCTGGGAGATGATTGTCTCTCAGAAAGTCAATTTGACCTTATGAT

JPNKWB778|AB863158.1| CAGGTTTTGGTATAATCTGGGAGATGATTGTCTCTCAGAAAGTCAATTTGACCTTATGAT

JPNM|AB863159.1| CAGGTTTTGGTATAATCTGGGAGATGATTGTCTCTCAGAAAGTCAATTTGACCTTATGAT

JPNN|AB863160.1| TAGGTTTTGGTATAATCTGGGAGATGATTGTCTCTCAGAAAGTCAATTTGACCTTATGAT

JPNS1|AB863161.1| TAATTTTTGGTATAATCTGGGAGATAATTGTCTCTCAGAAAGTCAATTTGACCTTATGAT

JPNS2|AB863162.1| TAATTTTTGGTATAATCTGGGAGATAATTGTCTCTCAGAAAGTCAATTTGACCTTATGAT

JPNTKD762|AB863163.1| CAGGTTTTGGTATAATCTGGGAGATGATTGTCTCTCAGAAAGTCAATTTGACCTTATGAT

JPNUV1|AB863164.1| CAGGTTTTGGTATAATCTGGGAGATGATTGTCTCTCAGAAAGTCAATTTGACCTTATGAT

JPNUV26|AB863165.1| CAGGTTTTGGTATAATCTGGGAGATGATTGTCTCTCAGAAAGTCAATTTGACCTTATGAT

TUR1|AB863166.1| CCGGTTTTGGTATAATCTGGGAGATGATTGTCTCTCAGAAAGTCAATTTGACCTTATGAT

TUR2|AB863167.1| CCGGTTTTGGTATAATCTGGGAGAAGATTGTCTCTCAGAAAGTCAATTCGATCTTATGAT

TUR4|AB863168.1| CCGGTTTTGGTATAATCTGGGAGAAGATTGTCTCTCAGAAAGTCAATTCGATCTTATGAT

TUR5|AB863169.1| TAGGTTCTGGTATAAACTGGGAGATGATTGTCTCTCAGAAAGTCAATTTGACCTTATGAT

TUR12|AB863170.1| TAGGTTCTGGTATAAACTGGGAGATGATTGTCTCTCAGAAAGTCAATTTGACCTTATGAT

TUR34|AB863171.1| TAGGTTCTGGTATAAACTGGGAGATGATTGTCTCTCAGAAAGTCAATTTGACCTTATGAT

TUR50|AB863172.1| TAGGTTCTGGTATAAACTGGGAGATGATTGTCTCTCAGAAAGTCAATTTGACCTTATGAT

TUR59|AB863173.1| TAGGTTTTGGTATAATCTGGGAGAAGATTGTCTCTCAGAAAGTCAATTTAACCTTATGAT

TUR69|AB863174.1| CCGGTTTTGGTATAGACTGGGAGATGATTGTCTCTCAGAAAGTCAATTTGACCTTATGAT

TUR81|AB863175.1| TCGGTTTTGGTATAATCTGGGAGATGATTGTCTCTCAGAAAGTCAATTTGACCTTATGAT

TUR84|AB863176.1| TCGGTTTTGGTATAATCTGGGAGATGATTGTCTCTCAGAAAGTCAATTTGACCTTATGAT

TUR94|AB863177.1| TAGGTTCTGGTATAGACTGGGAGATGATTGTCTCTCAGAAAGTCAATTTGACCTTATGAT

TUR213|AB863178.1| CCGGTTTTGGTATAAACTGGGAGATGATTGTCTCTCAGAAAGTCAATTTGACCTTATGAT

TUR214|AB863179.1| TAGGTTCTGGTATAAACTGGGAGATGATTGTCTCTCAGAAAGTCAATTTGACCTTATGAT

TUR216|AB863180.1| TAGGTTTTGGTATAATCTGGGAGAAGATTGTCTCTCAGAAAGTCAATTCGATCTTATGAT

TUR220|AB863181.1| TCGGTTTTGGTATAATCTGGGAGATGATTGTCTCTCAGAAAGTCAATTTGACCTTATGAT

TUR239|AB863182.1| CCGGTTTTGGTATAAACTGGGAGATGATTGTCTCTCAGAAAGTCAATTTGACCTTATGAT

TUR244|AB863183.1| CCGGTTTTGGTATAAACTGGGAGATGATTGTCTCTCAGAAAGTCAATTTGACCTTATGAT

TUR249|AB863184.1| CCGGTTTTGGTATAAACTGGGAGATGATTGTCTCTCAGAAAGTCAATTTGACCTTATGAT

TUR263|AB863185.1| TAGGTTCTGGTATAGACTGGGAGATGATTGTCTCTCAGAAAGTCAATTTGACCTTATGAT

TUR278|AB863186.1| TCGGTTTTGGTATAATCTGGGAGATGATTGTCTCTCAGAAAGTCAATTTGACCTTATGAT

TUR279|AB863187.1| CCGGTTTTGGTATAGACTGGGAGATGATTGTCTCTCAGAAAGTCAATTTGACCTTATGAT

TUR285|AB863188.1| TAGGTTCTGGTATAGACTGGGAGATGATTGTCTCTCAGAAAGTCAATTTGACCTTATGAT

TUR289|AB863189.1| CCGGTTTTGGTATAGACTGGGAGATGATTGTCTCTCAGAAAGTCAATTTGACCTTATGAT

TUR303|AB863190.1| CCGGTTTTGGTATAATCTGGGAGATGATTGTCTCTCAGAAAGTCAATTTGACCTTATGAT

TUR306|AB863191.1| TCGGTTTTGGTATAATCTGGGAGATGATTGTCTCTCAGAAAGTCAATTTGACCTTATGAT

Cabb-S|NC_001497.1| AAGATTGATGGAAGAGTCCCTTGACGGGGACCAAATTATTGATCTAACCTCTCTACCTAG

CRO180A|AB863192.1| AAGGTTAATGGAAGAGTCCCTTGACGGGGACCAAGTTATTGATCTAACCTCTCTACCTAG

GRC83|AB863193.1| AAGGTTAATGGAAGAGTCCCTTGACGGGGACCAAATTATTGATCTAACCTCTCTACCTAG

GRC84B|AB863194.1| AAGGTTAATGGAAGAGTCCCTTGACGGGGACCAAATTATTGATCTAACTTCTCTACCTAG

GRC86B|AB863195.1| AAGGTTAATGGAAGAGTCCCTCGACGGGGACCAAATTATTGATCTAACCTCTCTACCTAG

GRC86D|AB863196.1| AAGGTTAATGGAAGAGTCCCTTGACGGGGACCAAATTATTGATCTAACCTCTCTACCTAG

GRC87E|AB863197.1| AAGGTTAATGGAAGAGTCCCTTGACGGGGACCAAATTATTGATCTAACCTCTCTACCTAG

GRC87G|AB863198.1| AAGGTTAATGGAAGAGTCCCTTGACGGGGACCAAATTATTGATCTAACCTCTCTACCTAG

GRC91B|AB863199.1| AAGGTTAATGGAAGAGTCCCTTGACGGGGACCAAATTATTGATCTAACCTCTCTACCTAG

GRC92A|AB863200.1| AAGGTTAATGGAAGAGTCCCTTGACGGGGACCAAATTATTGATCTAACCTCTCTACCTAG

GRC92C|AB863201.1| AAGGTTAATGGAAGAGTCCCTTGACGGGGACCAAATTATTGATCTAACCTCTCTACCTAG

GRC92D|AB863202.1| AAGGTTAATGGAAGAGTCCCTTGACGGGGACCAAATTATTGATCTAACCTCTCTACCTAG

IRN1|AB863136.1| AAGGTTAATGGAAGAGTCCCTTGACGGGGACCAAATTATTGATCTAACCTCTCTACCTAG

IRN2|AB863137.1| AAGGTTAATGGAAGAGTCCCTTGACGGGGACCAAATTATTGATCTAACCTCTCTACCTAG

IRN3|AB863138.1| AAGGTTAATGGAAGAGTCCCTTGACGGGGACCAAATTATTGATCTAACCTCTCTACCTAG

IRN4|AB863139.1| AAGGCTAATGGAAGAGTCCCTTGACGGGGACCAAATTATTGATCTAACCTCTCTACCTAG

IRN5|AB863140.1| AAGGTTAATGGAAGAGTCCCTTGACGGGGACCAAATTATTGATCTAACCTCTCTACCTAG

IRN6|AB863141.1| AAGGTTAATGGAAGAATCCCTTGACGGGGACCAAATTATTGATCTAACCTCTCTACCTAG

IRN7|AB863142.1| AAGGTTAATGGAAGAGTCCCTTGACGGGGACCAAATTATTGATCTAACCTCTCTACCTAG

IRN8|AB863143.1| AAGGTTAATGGAAGAGTCCCTTGACGGGGACCAAATTATTGATCTAACCTCTCTACCTAG

IRN9|AB863144.1| AAGGTTAATGGAAGAGTCCCTTGACGGGGACCAAATTATTGATCTAACCTCTCTACCTAG

IRN10|AB863145.1| AAGGTTAATGGAAGAGTCCCTCGACGGGGACCAAATTATTGATCTAACCTCTCTACCTAG

IRN11|AB863146.1| AAGGTTAATGGAAGAGTCCCTTGACGGGGACCAAATTATTGATCTAACCTCTCTACCTAG

IRN12|AB863147.1| AAGGTTAATGGAAGAGTCCCTTGACGGGGACCAAATTATTGATCTAACCTCTCTACCTAG

IRN13|AB863148.1| AAGGTTAATGGAAGAGTCCCTTGACGGGGACCAAATTATTGATCTAACCTCTCTACCTAG

IRN14|AB863149.1| AAGGTTAATGGAAGAGTCCCTTGACGGGGACCAAATTATTGATCTAACCTCTCTACCTAG

IRN15|AB863150.1| AAGGTTAATGGAAGAGTCCCTCGACGGGGACCAAATTATTGATCTAACCTCTCTACCTAG

IRN16|AB863151.1| AAGATTAATGGAAGAGTCCCTCGACGGGGACCAAATTATTGATCTAACCTCTCTACCTAG

IRN17|AB863152.1| AAGGTTAATGGAAGAGTCCCTCGACGGGGACCAAATTATTGATCTAACCTCTCTACCTAG

IRN18|AB863153.1| AAGGTTAATGGAAGAGTCCCTTGACGGGGACCAAATTATTGATCTAACCTCTCTACCTAG

IRN19|AB863154.1| AAGGTTAATGGAAGAGTCCCTTGACGGGGACCAAATTATTGATCTAACCTCTCTACCTAG

IRN20|AB863155.1| AAGGTTAATGGAAGAGTCCCTCGACGGGGACCAAATTATTGATCTAACCTCTCTACCTAG

IRN21|AB863156.1| AAGGTTAATGGAAGAGTCCCTTGACGGGGACCAAATTATTGATCTAACCTCTCTACCTAG

JPNHGB340|AB863157.1| AAGGTTAATGGAAGAGTCCCTTGACGGGGACCAAATAATTGATCTAACCTCTCTACCTAG

JPNKWB778|AB863158.1| AAGGTTAATGGAAGAGTCCCTTGACGGGGACCAAATTATTGATTTAACCTCTCTACCTAG

JPNM|AB863159.1| AAGGTTAATGGAAGAGTCCCTTGACGGGGACCAAATAATTGATCTAACCTCTCTACCTAG

JPNN|AB863160.1| AAGGTTAATGGAAGAGTCCCTTGACGGGGACCAAATAATTGATCTAACCTCTCTACCTAG

JPNS1|AB863161.1| AAGGTTAATGGAAGAGTCCCTTGACGGGGACCAAATAATTGATCTAACCTCTCTACCTAG

JPNS2|AB863162.1| AAGGTTAATGGAAGAGTCCCTTGACGGGGACCAAATAATTGATCTAACCTCTCTACCTAG

JPNTKD762|AB863163.1| AAGGTTAATGGAAGAGTCCCTTGACGGGGACCAAATTATTGATCTGACCTCTCTACCTAG

JPNUV1|AB863164.1| AAGGTTAATGGAAGAGTCCCTCGACGGGGACCAAATAATTGATCTAACCTCTCTACCTAG

JPNUV26|AB863165.1| AAGGTTAATGGAAGAGTCCCTTGACGGGGACCAAATAATTGATCTAACCTCTCTACCTAG

TUR1|AB863166.1| AAGGTTAATGGAAGAGTCCCTTGACGGGGACCAAATTATTGATCTAACCTCTCTACCTAG

TUR2|AB863167.1| AAGATTGATGGAAGAGTCCCTTGACGGGGACCAAATTATTGATCTAACCTCTCTACCTAG

TUR4|AB863168.1| AAGATTGATGGAAGAGTCCCTTGACGGGGACCAAATTATTGATCTAACCTCTCTACCTAG

TUR5|AB863169.1| AAGGTTAATGGAAGAGTCCCTTGACGGGGACCAAATTATTGATCTAACCTCTCTACCTAG

TUR12|AB863170.1| AAGGTTAATGGAAGAGTCCCTTGACGGGGACCAAATTATTGATCTAACCTCTCTACCTAG

TUR34|AB863171.1| AAGGTTAATGGAAGAGTCCCTGGACGGGGACCAAATTATTGATCTAACCTCTCTACCTAG

TUR50|AB863172.1| AAGGTTAATGGAAGAGTCCCTTGACGGGGACCAAATTATTGATCTAACCTCTCTACCTAG

TUR59|AB863173.1| AAGGTTAATGGAAGAGTCCCTGGACGGGGACCAAATTATTGATCTAACCGCTCTACCTAG

TUR69|AB863174.1| AAGGTTAATGGAAGAGTCCCTCGACGGGGACCAAATTATTGATCTAACCTCTCTACCTAG

TUR81|AB863175.1| AAGGTTAATGGAAGAGTCCCTTGACAGGGACCAAATTATTGATCTAACCTCTCTACCTAG

TUR84|AB863176.1| AAGGTTAATGGAAGAGTCCCTTGACGGGGACCAAATTATTGATCTAACCTCTCTACCTAG

TUR94|AB863177.1| AAGGTTAATGGAAGAGTCCCTCGACGGGGACCAAATTATTGATCTAACCTCTCTACCTAG

TUR213|AB863178.1| AAGGTTAATGGAAGAGTCCCTTGACGGGGACCAAATAATTGATCTAACCTCTCCACCTAG

TUR214|AB863179.1| AAGGTTAATGGAAGAGTCCCTTGACGGGGACCAAATTATTGATCTAACCTCTCTACCTAG

TUR216|AB863180.1| AAGATTGATGGAAGAGTCCCTTGACGGGGACCAAATTATTGATCTAACCTCTCTACCTAG

TUR220|AB863181.1| AAGGTTAATGGAAGAGTCCCTTGACGGGGACCAAATTATTGATCTAACCTCTCTACCTAG

TUR239|AB863182.1| AAGGTTAATGGAAGAGTCCCTTGACGGGGACCAAATTATTGATCTGACCTCTCTACCTAG

TUR244|AB863183.1| AAGGTTAATGGAAGAGTCCCTCGACGGGGACCAAATTATTGATCTAACCTCTCTACCTAG

TUR249|AB863184.1| AAGGTTAATGGAAGAGTCCCTTGACGGGGACCAAATAATTGATCTAACCTCTCTACCTAG

TUR263|AB863185.1| AAGGTTAATGGAAGAGTCCCTTGACGGGGACCAAATTATTGATCTAACCTCTCTACCTAG

TUR278|AB863186.1| AAGGTTAATGGAAGAGTCCCTTGACGGGGACCAAATTATTGATCTAACCTCTCTACCTAG

TUR279|AB863187.1| AAGGTTAATGGAAGAGTCCCTCGACGGGGACCAAATTATTGATCTAACCTCTCTACCTAG

TUR285|AB863188.1| AAGGTTAATGGAAGAGTCCCTTGACAGGGACCAAATTATTGATCTAACCTCTCTACCTAG

TUR289|AB863189.1| AAGGTTAATGGAAGAGTCCCTTGACGGGGACCAAATTATTGATCTAACCTCTCTACCTAG

TUR303|AB863190.1| AAGGTTAATGGAAGAGTCCCTTGACGGGGACCAAATTATTGATCTAACCTCTCTACCTAG

TUR306|AB863191.1| AAGGTTAATGGAAGAGTCCCTTGACGGGGACCAAATTATTGATCTAACCTCTCTACCTAG

Cabb-S|NC_001497.1| TGATAATTTGCAGGTTGAACAGGTTATGACAACT---ACCGAAGACTCAATCTCGGAAGA

CRO180A|AB863192.1| TGACAATTTGCAGGTCGAACAGGTTATGACAACA---ACCGAAGACTCGATCTCGGAAGA

GRC83|AB863193.1| TGATAATTTGCAGGTTGAACAGGTTATGACAACT---ACCGACGACTCGATCTCGGAAGA

GRC84B|AB863194.1| TGATAATTTGCAGGTTGAACAGGTTATGACAACT---ACCGACGACTCGATCTCGGAAGA

GRC86B|AB863195.1| TAATAATTGGCAGGTTGAACAGGTTATGACAACA---ACCGAAGACTCGATCTCGGATGA

GRC86D|AB863196.1| TGATAATTTGCAGGTTGAACAGGTTATGACAACT---ACCGACGACTCGATCTCGGAAGA

GRC87E|AB863197.1| TGATAATTTGCAGGTTGAACAGGTTATGACAACT---ACCGACGACTCGATCTCGGAAGA

GRC87G|AB863198.1| TGATAATTTGCAGGTTGAACAGGTTATAACAACT---ACCGACGACTCGATCTCGGAAGA

GRC91B|AB863199.1| TGATAATTTGCAGGTTGAACAGGTTATGACAACT---ACCGACGACTCGATCTCGGAAAA

GRC92A|AB863200.1| TGATAATTTGCAGGTTGAACAGGTTATGACAACT---ACCGACGACTCGATCTCGGAAGA

GRC92C|AB863201.1| TGATAATTTGCAGGTTGAACAGGTTATGACAACT---ACCGACGACTCGATCTCGGAAGA

GRC92D|AB863202.1| TGATAATTTGCAGGTTGAACAGGTTATGACAACT---ACCGACGACTCGATCTCGGAAGA

IRN1|AB863136.1| TGATAATTTGCAGGTTGAACAGGTTATGACAACT---ACCGAAGACTCGATCTCGGA---

IRN2|AB863137.1| TGATAATTTGCAGGTTGAACAGGTTACGACAACT---ACCGAAGACTGGATCTCGGA---

IRN3|AB863138.1| TGATAATTTGCAGGTTGAACAGGTTATGACAACT---ACCGAAGACTCGATCTCGGA---

IRN4|AB863139.1| TGATAATTTGCAGGTTGAACAGGTTATGACAACT---ACCGAAGACTCGATCTCGGA---

IRN5|AB863140.1| TGATAATTTGCAGGTTGAACAGGTTATGACAACT---ACCGAAGACTCGATCTCGGA---

IRN6|AB863141.1| TGATAATTTGCAGGTTGAACAGGTTATGACAACT---ACCGAAGACTCGATCTCGGAAG-

IRN7|AB863142.1| TGATAATTTGCAGGTTGAACAGGTTATGACAACT---ACCGAAGACTCGATCTCGGA---

IRN8|AB863143.1| TGATAATTTGCAGGTTGAACAGGTTATGACAACT---ACCGAAGACTCGATCTCGGA---

IRN9|AB863144.1| TGATAATTTGCAGGTTGAACAGGTTAGGACAACT---ACCGAAGACTCGATCTCGGA---

IRN10|AB863145.1| TGATAATTTGCAGGTTGAACAGGTTACGACAACT---ACCGAAGACTGGATCTCGGA---

IRN11|AB863146.1| TGATAATTTGCAGGTTGAACAGGTTATGACAACT---ACCGAAGACTCGATCTCGGA---

IRN12|AB863147.1| TGATAATTTGCAGGTTGAACAGGTTATGACAACT---ACCGAAGACTCGATCTCGGA---

IRN13|AB863148.1| TGATAATTTGCAGGTTGAACAGGTTATGACAACT---ACCGAAGACTCGATCTCGGA---

IRN14|AB863149.1| TGATAATTTGCAGGTTGAACAGGTTATGACAACT---ACCGAAGACTCGATCTCGGA---

IRN15|AB863150.1| TGATAACTTGCAGGTTGAACAGGTTACGACAATC---ACCGAAGACTCGATCTCGGA---

IRN16|AB863151.1| TGATAACTTGCAGGTTGAACAGGTTACGACAACC---ACCGAAGACTCGATCTCGGAAGA

IRN17|AB863152.1| TGATAATTTGCAGGTTGAACAGGTTACAACAACT---ACCGAAGACTCGTTCTCGGAAGA

IRN18|AB863153.1| TGATAATTTGCAGGTTGAACAGGTTATGACAACT---ACCGAAGACTCAATCTCGGA---

IRN19|AB863154.1| TGATAATTTGCAGGTTGAACAGGTTACGACAACT---ACCGAAGACTGGATCTCGGA---

IRN20|AB863155.1| TGATAATTTGCAGGTTGAACAGGTTACAACAACT---ACCGAAGACTCGTTCTCGGAAGA

IRN21|AB863156.1| TGATAATTTGCAGGTTGAACAGGTTATGACAACT---ACCGAAGACTCGATCTCGGA---

JPNHGB340|AB863157.1| TGATAATTTGCAGGTCGAACAGGTTATGACAACT---ACCGACGACTCGATCTCGGAAG-

JPNKWB778|AB863158.1| TGATAATTTGCAGGTCGAACAGGTTATGACAACT---ACCGACGACTCGATCTCGGAA--

JPNM|AB863159.1| TGATAATTTGCAGGTCGAACAGGTTATGACAACT---ACCGACGACTCGATCTCGGAA--

JPNN|AB863160.1| TGACAATTTGCAGGTTGAACAGGTTATGACAAC---TACCGAAGACTCGATCTCGGAA--

JPNS1|AB863161.1| TGATAATTTGCAGGTCGAACAGGTTATGACAACCACCACCGAAGACTCGATCTCGGAA--

JPNS2|AB863162.1| TGATAATTTGCAGGTCGAACAGGTTATGACAACCACCACCGAAGACTCGATCTCGGAA--

JPNTKD762|AB863163.1| TGATAATTTGCAGGTCGAACAGGTTATGACAACT---ACCGACGACTCGATCTCGGAA--

JPNUV1|AB863164.1| TGATAATTTGCAGGTCGAACAGGTTATGACAACT---ACCGACGACTCGATCTCGGAA--

JPNUV26|AB863165.1| TGATAATTTGCAGGTTGAACAGGTTATGACAACT---ACCGACGACTCGATCTCGGAAG-

TUR1|AB863166.1| TGATAATTTGCAGGTTGAACAGGTTACGACAACA---ACCGAAGACGCGATCTCGG----

TUR2|AB863167.1| TGATAATTTGCAGGTCGAACAGGTTACGACGACT---ACCGAAGACTCGATCTCGGA---

TUR4|AB863168.1| TGATAATTTGCAGGTCGAACAGGTTACGACGACT---ACCGAAGACTCGATCTCGGA---

TUR5|AB863169.1| TGACAACTTGCAGGTTGAACAGGTTATGACAACA---ACCGAAGACTCGATCTCGGA---

TUR12|AB863170.1| TGATAATTTGCAGGTCGAACAGGTTATGACAACT---ACCGAAGACTCAATCTCGGA---

TUR34|AB863171.1| TGATAATTTGCAGGTCGAACAGGTTATGACAACT---ACCGAAGACTCGATCTCGGA---

TUR50|AB863172.1| TGATAATTTGCAGATCGAACAGGTTATGACAACT---ACCGAAGACTCGATCTCGGA---

TUR59|AB863173.1| TGATAATTTGCAGGTCGAACAGGTTATGACAACT---ACCGAAGACTCGATCTCGGAAGA

TUR69|AB863174.1| TGATAATTTGCAGGTCGAACAGGTTATGACAACT---ACCGAAGACTCGATCTCGGAAGA

TUR81|AB863175.1| TGATAATTTGCAGGTCGAACAGGTTATGACCACT---ACCGAAGACTCGATCTCGGA---

TUR84|AB863176.1| TGATAATTTGCAGGTTGAACAGGTTATGACCACT---ACCGAAGACTCGATCTCGG----

TUR94|AB863177.1| TGATAATTTGCAGGTCGAACAGGTTATGACAACT---ACCGAAGACTCGATCTCGGAAGA

TUR213|AB863178.1| TGATAATTTGCAGGTTGAACAGGTTATGACAACA---ACCGAAGACTCGATCTCGGA---

TUR214|AB863179.1| TGATAATTTGCAGGTCGAACAGGTTATGACAACT---ACCGAAGACTCGATCTCGGAAGA

TUR216|AB863180.1| TGATAATTTGCAGGTTGAACAGGTTATGACAACA---ACCGAAGACTCGATCTCAGA---

TUR220|AB863181.1| TGATAATTTGCAGGTTGAACAGGTTATGACCACT---ACCGAAGACTCGATCTCGG----

TUR239|AB863182.1| TGATAATTTGCAGGTCGAACAGGTTACGACGACT---ACCGAAGACTCGATCTCGGAAGA

TUR244|AB863183.1| TGATAATTTGCAGGTTGAACAGGTTATGACAACT---ACCGAAGACTCGATCTCGGAAGA

TUR249|AB863184.1| TGATAATTTGCAGGTTGAACAGGTTATGACAACA---ACCGAAGACTCGATCTCGGA---

TUR263|AB863185.1| TGATAATTTGCAGGTCGAACAGGTTATGACAACT---ACCGAAGACTCGATCTCGGAAGA

TUR278|AB863186.1| TGATAATTTGCAGGTTGAACAGGTTATGACAACG---ACCGAAGACTCGATCTCGGA---

TUR279|AB863187.1| TGATAATTTGCAGGTTGAACAGGTTATGACCACT---ACCGAAGACTCGATCTCGGA---

TUR285|AB863188.1| TGATAATTTGCAGGTTGAACAGGTTATGACAACT---ACCGAAGACTCGATCTCGGAAGA

TUR289|AB863189.1| TGATAATTTGCAGGTCGAACAGGTTATGACAACT---ACCGAAGACTCGATCTCGGAAGA

TUR303|AB863190.1| TGATAATTTGCAGGTTGAACAGGTTATGACAACA---ACCGAAGACTCGATCTCGGA---

TUR306|AB863191.1| TGATAATTTGCAGGTTGAACAGGTTATGACCACT---ACCGAAGACTCAATCTCGG----

Cabb-S|NC_001497.1| A---------------------------------------GAATCAGAATTCCTTCTAGC

CRO180A|AB863192.1| A---------------------------------------GAATCAGAATTCCTTCTAGC

GRC83|AB863193.1| ATCAGAA---------------------------------CTATCAGAATTCCTTCTAGC

GRC84B|AB863194.1| ATCAGAG---------------------------------CTATCAGAGTTCCTTCTAGC

GRC86B|AB863195.1| ATCAGAA---------------------------------CTATCAGAGTTCCTTCTAGC

GRC86D|AB863196.1| ATCAGAA---------------------------------CTATCAGAGTTCCTTCTAGC

GRC87E|AB863197.1| ATCAGAA---------------------------------CTATCAGAGTTCCTTCTAGC

GRC87G|AB863198.1| ATCAGAA---------------------------------CTATCAGAATTCCTTCTAGC

GRC91B|AB863199.1| ATCAGAA---------------------------------CTATCGGAGTTCCTTCTAGC

GRC92A|AB863200.1| ATCAGAA---------------------------------CTATCAGAATTCCTTCTAGC

GRC92C|AB863201.1| ATCAGAA---------------------------------CTATCAGAGTTCCTTCTAGC

GRC92D|AB863202.1| ATCAGAT---------------------------------CTATCAGAGTTTCTTCTAGC

IRN1|AB863136.1| ------A---------------------------------GAATCAGAATTCCTTCTAGC

IRN2|AB863137.1| ------A---------------------------------GAATCAGAATTCCTTCTAGC

IRN3|AB863138.1| ------A---------------------------------GAATCAGAATTCCTTCTAGC

IRN4|AB863139.1| ------A---------------------------------GAATCAGAATTCCTTCTAGC

IRN5|AB863140.1| ------A---------------------------------GAATCAGAATTCCTTCTAGC

IRN6|AB863141.1| -----------------------------------------AATCAGAATTCCTTCTAGC

IRN7|AB863142.1| ------A---------------------------------GAATCAGAATTCCTTCTAGC

IRN8|AB863143.1| ------A---------------------------------GAATCAGAATTCCTTCTAGC

IRN9|AB863144.1| ------A---------------------------------GAATCAGAATTTCTTCTAGC

IRN10|AB863145.1| ------A---------------------------------GAATCAGAATTCCTTCTAGC

IRN11|AB863146.1| ------A---------------------------------GAATCAGAATTCCTTCTAGC

IRN12|AB863147.1| ------A---------------------------------GAATCAGAATTCCTTCTAGC

IRN13|AB863148.1| ------A---------------------------------GAATCAGAATTCCTTCTAGC

IRN14|AB863149.1| ------A---------------------------------GAATCAGAATTCCTTCTAGC

IRN15|AB863150.1| ------A---------------------------------GAATCAGAATTCCTTCTAGC

IRN16|AB863151.1| ATCAGAATTCCTTCTAGCAATAGGAGAAACAACTGAAGAAGAATCAGAATTCCTTCTAGC

IRN17|AB863152.1| ATCAGAATTCCTCCTAGCAATAGGAGAAACATCTGAAGACGAATCAGAATTCCTTCTAGC

IRN18|AB863153.1| ------A---------------------------------GAATCAGAATTCCTTCTAGC

IRN19|AB863154.1| ------A---------------------------------GAATCAGAATTCCTTCTAGC

IRN20|AB863155.1| ATCAGAATTCCTCCTAGCAATAGGAGAAACATCTGAAGACGAATCAGAATTCCTTCTAGC

IRN21|AB863156.1| ------A---------------------------------GAATCAGAATTCCTTCTAGC

JPNHGB340|AB863157.1| -----------------------------------------AATCAGAATTCCTCCTAGC

JPNKWB778|AB863158.1| ----------------------------------------GAATCAGAATTCCTTCTAGC

JPNM|AB863159.1| ----------------------------------------GAATCAGAATTCCTTCTAGC

JPNN|AB863160.1| ----------------------------------------AGAACAGAAACTCTTCTAGC

JPNS1|AB863161.1| ----------------------------------------AGAACAGAAATTCTTCTAGC

JPNS2|AB863162.1| ----------------------------------------AGAACAGAAATTCTTCTAGC

JPNTKD762|AB863163.1| ----------------------------------------GAATCAGAATTCCTTCTAGC

JPNUV1|AB863164.1| ----------------------------------------GAATCAGAATTCCTTCTAGC

JPNUV26|AB863165.1| -----------------------------------------AATCAGAATTCCTTCTAGC

TUR1|AB863166.1| -----AC---------------------------------GAATCAGAGTTCCTTCTAGC

TUR2|AB863167.1| ------A---------------------------------GAATCAGAATTCCTTCTAGC

TUR4|AB863168.1| ------A---------------------------------GAATCAGAATTCCTTCTAGC

TUR5|AB863169.1| ------T---------------------------------GAATCAGAATTCCTTCTAGC

TUR12|AB863170.1| ATCAGAC---------------------------------TTGTCAGAATTCCTTCTAGC

TUR34|AB863171.1| ATCAGAC---------------------------------TTGTCAGAATTCCTTCTAGC

TUR50|AB863172.1| ------T---------------------------------GAATCAGAATTCCTTCTAGC

TUR59|AB863173.1| TTCAGAT---------------------------------CTATCAGAATTCCTTCTAGC

TUR69|AB863174.1| ATCAGAA---------------------------------TTATCAGAATTCCTTCTAGC

TUR81|AB863175.1| ------C---------------------------------GAATCAGAGTTCCTTCTAGC

TUR84|AB863176.1| -----AC---------------------------------GAATCAGAGTTCCTTCTAGC

TUR94|AB863177.1| TTCAGAT---------------------------------CTATCAGAATTCCTTCTAGC

TUR213|AB863178.1| ------C---------------------------------GAATCAGAGTTCCTTCTAGC

TUR214|AB863179.1| ATCAGAT---------------------------------CTATCAGAATTCCTTCTAGC

TUR216|AB863180.1| ------C---------------------------------GAATCAGAGTTCCTTCTAGC

TUR220|AB863181.1| -----AC---------------------------------GAATCAGAGTTCCTTCTAGC

TUR239|AB863182.1| ATCAGAATTCCTTCTAGCTATAGGAGAGACGTCTGAAGAAGAATCAGAATTTCTTCTAGC

TUR244|AB863183.1| TTCAGAT---------------------------------CTATCAGAATTCCTTCTAGC

TUR249|AB863184.1| ------C---------------------------------GAATCAGAGTTCCTTCTAGC

TUR263|AB863185.1| TTCAGAT---------------------------------CTATCAGAATTCCTTCTAGC

TUR278|AB863186.1| ------T---------------------------------GAATCAGAGTTCCTTCTAGC

TUR279|AB863187.1| ------C---------------------------------GAATCAGAGTTCCTTCTAGC

TUR285|AB863188.1| TTCGGAT---------------------------------CTATCAGAATTCCTTCTAGC

TUR289|AB863189.1| TTCAGAT---------------------------------CTATCAGAATTCCTTCTAGC

TUR303|AB863190.1| ------C---------------------------------GAGTCAGAGTTCCTTCTAGC

TUR306|AB863191.1| -----AC---------------------------------GAATCAGAATTCCTTCTAGC

Cabb-S|NC_001497.1| AATAGGAGAAACATCTGAAGAAGAAAGCGATTCAGGAGAAGAACCTGAATTCGAGCAAGT

CRO180A|AB863192.1| AATAGGAGAAACGTCTGAAGAAGAAAGCGATTCAGGAGAAGAACCTGAATTCGAACAAGT

GRC83|AB863193.1| CATAGGAGAAACATCTGAAGACGAAAGCGATTCAGGAGAGGAACCTGAATTCGAACAAGT

GRC84B|AB863194.1| CATAGGAGAAACATCTGAAGACGAAAGCGATTCAGGAGAGGAACCTGAATTCGAACAAGT

GRC86B|AB863195.1| CATAGGAGAAACATCTGAAGAAGAAAGCGATTCAGGAGAGGAACCTGAATTCGAACAAGT

GRC86D|AB863196.1| TATAGGAGAAACATCTGAAGACGAAAGCGATTCAGGAGAAGAACCTGAATTCGAACAAGT

GRC87E|AB863197.1| CATAGGAGAAACATCTGAAGACGAAAGCGATTCAGGAGAGGAACCTGAATTCGAACAAGT

GRC87G|AB863198.1| CATAGGAGAAACATCTGAAGACGAAAGCGATTCAGGAGAGGAACCTGAATTCGAACAAGT

GRC91B|AB863199.1| CATAGGAGAAACATCTGAAGACGAAAGCGATTCAGGAGAAGAACCTGAATTCGAACAAGT

GRC92A|AB863200.1| CATAGGAGAAACATCTGAAGACGAAAGCGATTCAGGAGAGGAACCTGAATTCGAACAAGT

GRC92C|AB863201.1| CATAGGAGAAACATCTGAAGACGAAAGCGATTCAGGAGAAGAACCTGAATTCGAACAAGT

GRC92D|AB863202.1| CATAGGAGAAACATCTGAAGACGAAAGCGATTCAGGAGAAGAACCTGAATTCGAACAAGT

IRN1|AB863136.1| AATAGGAGAAACTTCTGAAGACGAAAGCGATTCAGGAGAAGAACCTGAATTCGAACAAGT

IRN2|AB863137.1| AATAGGAGAAACATCTGAAGACGAAAGCGATTCAGGAGAAGAACCTGAATTCGAACAAGT

IRN3|AB863138.1| AATAGGAGAAACTTCTGAAGACGAAAGCGATTCAGGAGAAGAACCTGAATTCGAACAAGT

IRN4|AB863139.1| AATAGGAGAAACATCTGAAGACGAAAGCGATTCAGGAGAAGAACCTGAATTCGAACAAGT

IRN5|AB863140.1| AATAGGAGAAACATCTGAAGACGAAAGCGATTCAGGAGAAGAACCTGAATTCGAACAAGT

IRN6|AB863141.1| AATAGGAGAAACATCTGAAGAAGAAAGCGATTCAGGAGAGGAACCTGAATTCGAACAAGT

IRN7|AB863142.1| AATAGGAGAAACATCTGAAGACGAAAGCGATTCAGGAGAAGAACCTGAATTCGAACAAAT

IRN8|AB863143.1| AATAGGAGAAACATCTGAAGACGAAAGCGATTCAGGAGAAGAACCTGAATTCGAACAAGT

IRN9|AB863144.1| AATAGGAGAAACATCTGAAGACGAAAGCGATTCAGGAGAAGAACCTGAATTCGAACAAGT

IRN10|AB863145.1| AATAGGAGAAACATCTGAAGACGAAAGCGATTCAGGAGAAGAACCTGAATTCGAACAAGT

IRN11|AB863146.1| AATAGGAGAAACATCTGAAGACGAAAGCGATTCAGGAGAAGAACCTGAATTCGAGCAAGT

IRN12|AB863147.1| AATAGGAGAAACATCTGAAGACGAAAGCGATTCAGGAGAAGAACCTGAATTCGAACAAGT

IRN13|AB863148.1| AATAGGAGAAACATCTGAAGACGAAAGCGATTCAGGAGAAGAACCTGAATTCGAACAAGT

IRN14|AB863149.1| AATAGGAGAAACATCTGAAGACGAAAGCGATTCAGGAGAAGAACCTGAATTCGAACAAGT

IRN15|AB863150.1| GATAGGAGAAACGTCTGAAGAAGAGAGTGATTCAGGAGAAGAACCTGAATTCGAACAAGT

IRN16|AB863151.1| GATAGGAGAAACGTCTGAAGAAGAAAGTGATTCAGGAGAAGAACCTGAATTCGAACAAGT

IRN17|AB863152.1| AATAGGAGAAACGTCTGAAGAAGAAAGTGATTCAGGAGAAGAACCTGAATTCGAACAAGT

IRN18|AB863153.1| AATAGGAGAAACATCTGAAGACGAAAGCGATTCAGGAGAAGAACCTGAATTCGAACAAGT

IRN19|AB863154.1| AATAGGAGAAACATCTGAAGACGAAAGCGATTCAGGAGAAGAACCTGAATTCGAACAAGT

IRN20|AB863155.1| AATAGGAGAAACATCTGAAGAAGAAAGTGATTCAGGAGAAGAACCTGAATTCGAACAAGT

IRN21|AB863156.1| AATAGGAGAAACATCTGAAGACGAAAGCGATTCAGGAGAAGAACCTGAATTCGAACAAGT

JPNHGB340|AB863157.1| AATAGGAGAAACATCTGAAGACGAAAGTGATTCAGGAGAAGAACCTGAATTCGAACAAGT

JPNKWB778|AB863158.1| AATAGGAGAAACATCTGAAGACGAAAGTGATTCAGGAGAAGAACCTGAATTCGAGCAAGT

JPNM|AB863159.1| AATAGGAGAAACATCTGAAGACGAAAGTGATTCAGGAGAAGAACCTGAATTCGAACAAGT

JPNN|AB863160.1| AATAGGAGAAACATCTGAAGACGAAAGCGATTCAGGAGAAGAACCTGAATTCGAACAAGT

JPNS1|AB863161.1| AATAGGAGAAACATCTGAAGACGAAAGCGATTCAGGAGAAGAACCTGAATTCGAACAAGT

JPNS2|AB863162.1| AATAGGAGAAACATCTGAAGACGAAAGCGATTCAGGAGAAGAACCTGAATTCGAACAAGT

JPNTKD762|AB863163.1| AATAGGAGAAACATCTGAAGACGAAAGTGATTCAGGAGAAGAACCTGAATTCGAACAAGT

JPNUV1|AB863164.1| AATAGGAGAAACATCTGAAGACGAAAGTGATTCAGGAGAAGAACCTGAATTCGAACAAGT

JPNUV26|AB863165.1| AATAGGAGAAACATCTGAAGACGAAAGTGATTCAGGAGAAGAACCTGAATTCGAACAAGT

TUR1|AB863166.1| AATAGGAGAAACATCTGAAGAAGAAAGCGATTCAGGCGAAGAACCTGAGTTCGAACAAGT

TUR2|AB863167.1| AATAGGAGAAACATCTGAAGACGAAAGTGATTCAGGAGAAGAACCTGAATTCGAACAAGT

TUR4|AB863168.1| AATAGGAGAAACATCTGAAGACGAAAGTGATTCAGGAGAAGAACCTGAATTCGAACAAGT

TUR5|AB863169.1| AATAGGAGAAACATCTGAAGAAGAAAGCGATTCAGGCGAAGAACCTGAATTCGAACAAGT

TUR12|AB863170.1| AATAGGGGAAACGTCTGAAGAAGAAAGCGATTCAGGAGAAGAACCTGAATTCGAACAAGT

TUR34|AB863171.1| AATAGGAGAAACGTCTGAAGAAGAAAGCGATTCAGGAGAAGAACCTGAATTCGAACAAGT

TUR50|AB863172.1| AATAGGAGAAACGTCTGAAGAAGAAAGCGATTCAGGAGAAGAACCTGAGTTCGAACAAGT

TUR59|AB863173.1| AATAGGAGAAACATCTGAAGAAGAAAGCGATTCAGGCGAAGAACCTGAATTCGAACAAGT

TUR69|AB863174.1| GATAGGAGAAACATCTGAAGAAGAAAGCGATTCAGGAGAAGAACCTGAATTCGAACAAGT

TUR81|AB863175.1| AATAGGAGAAACATCTGAAGAAGAAAGCGATTCAGGCGAAGAACCTGAATTCGAACAAGT

TUR84|AB863176.1| AATAGGAGAAACATCTGAAGAAGAAAGCGATTCAGGCGAAGAACCTGAATTCGAACAAGT

TUR94|AB863177.1| AATAGGAGAAACGTCTGAAGAAGAAAGCGATTCAGGAGAAGAACCTGAATTCGAACAAGT

TUR213|AB863178.1| AATAGGGGAAACATCTGAAGAAGAAAGCGATTCAGGCGAAGAACCTGAGTTCGAACAAGT

TUR214|AB863179.1| AATAGGGGAAACGTCTGAAGAAGAAAGCGACTCAGGAGAAGAACCTGAATTCGAACAAGT

TUR216|AB863180.1| AATAGGAGAAACATCTGAAGAAGAAAGCGATTCAGGCGAAGAACCTGAATTCGAACAAGT

TUR220|AB863181.1| AATAGGAGAAACATCTGAAGAAGAAAGCGATTCAGGCGAAGAACCTGAATTCGAACAAGT

TUR239|AB863182.1| AATAGGAGAAACGTCTGAAGAAGAAAGCGATTCAGGAGAAGAACCTGAATTCGAACAAGT

TUR244|AB863183.1| AATAGGAGAAACGTCTGAAGAAGAAAGCGATTCAGGAGAAGAACCTGAATTCGAACAAGT

TUR249|AB863184.1| AATAGGAGAAACATCTGAAGAAGAAAGCGATTCAGGCGAAGAACCTGAGTTCGAACAAGT

TUR263|AB863185.1| AATAGGAGAAACGTCTGAAGAAGAAAGCGACTCAGGAGAAGAACCTGAATTCGAACAAGT

TUR278|AB863186.1| AATAGGAGAAACATCTGAAGAAGAAAGCGATTCAGGCGAAGAACCTGAATTCGAACAAGT

TUR279|AB863187.1| AATAGGAGAAACATCTGAAGAAGAAAGCGATTCAGGCGAAGAACCTGAATTCGAACAAGT

TUR285|AB863188.1| AATAGGAGAAACATCTGAAGAAGAAAGCGATTCAGGAGAAGAACCTGAATTCGAACAAGT

TUR289|AB863189.1| AATAGGAGAAACGTCTGAAGAAGAAAGCGACTCAGGAGAAGAACCTGAATTCGAACAAGT

TUR303|AB863190.1| AATAGGAGAAACATCTGAAGAAGAAAGCGATTCAGGCGAAGAACCTGAGTTCGAACAAGT

TUR306|AB863191.1| AATAGGAGAAACATCTGAAGAAGAAAGCGATTCAGGCGAAGAACCTGAATTCGAACAAGT

Cabb-S|NC_001497.1| TCGAATGGATCGAACAGGAGGAACGGAGATTCCAAAAGAAGAAGATGGTGAAGGACCATC

CRO180A|AB863192.1| TCGAATGGATCGAACAGGAGGAACGGAGATTCCTAAAGAAGAAGATGGTGGAGAACCATC

GRC83|AB863193.1| TCGCATGGATCGAACAGGAGGAACGGAGATTCCCAAAGAAGAAGATGGCGGAGAACCATC

GRC84B|AB863194.1| TCGCATGGATCGAACAGGAGGAACGGAGATTCCCAAAGAAGAAGATGGCGGAGAACCATC

GRC86B|AB863195.1| TCGAATGGATCGAACAGGAGGAACGGAGATTCCCAAAGAAGAAGATGGCGGAGAACCATC

GRC86D|AB863196.1| TCGCATGGACCGAACAGGAGGAACGGAGATTCCCAAAGAAGAAGATGGCGGAGAACCATC

GRC87E|AB863197.1| TCGAATGGATCGAACAGGAGGAACGGAGATTCCCAAAGAAGAAGATGGCGGAGAACCATC

GRC87G|AB863198.1| TCGCATGGATCGAACAGGAGGAACGGAGATTCCCAAAGAAGAAGATGGCGGAGAACCATC

GRC91B|AB863199.1| TCGAATGGATCGAACAGGAGGAACGGAGATTCCCAAAGAAGAAGATGGCGGAGAACCATC

GRC92A|AB863200.1| TCGCATGGATCGAACAGGAGGAACGGAGATTCCCAAAGAAGAAGATGGCGGAGAACCATC

GRC92C|AB863201.1| TCGAATGGATCGAACAGGAGGAACGGAGATTCCCAAAGAAGAAGATGGCGGAGAACCATC

GRC92D|AB863202.1| TCGCATGGATCGAACAGGAGGAACGGAGATTCCTAAAGAAGAAGATGGTGGAGAACCATC

IRN1|AB863136.1| TCGAATGGATCGAACAGGAGGAACGGAGATTCCCAAAGAAGAAGATGGTGAAGGACCATC

IRN2|AB863137.1| TCGTATGGATCGAACAGGAGGAACGGAGATTCCTAAAGAAGAAGATGGCGGAGAACCATC

IRN3|AB863138.1| TCGAATGGATCGAACAGGAGGAACGGAGATTCCCAAAGAAGAAGATGGTGAAGGACCATC

IRN4|AB863139.1| TCGTATGGATCGAACAG---GAACGGAGATTCCCAAAGAAGAAGATGGTGAAGGACCATC

IRN5|AB863140.1| TCGTATGGATCGAACAGGAGGAACAGAGATTCCCAAAGAAGAAGATGGTGAAGGACCATC

IRN6|AB863141.1| TCGTATGGATCGAACAGGAGGAACGGAGATTCCCAAAGAAGAAGATGGTGAAGGACCATC

IRN7|AB863142.1| TCGTATGGATCGAACAGGAGGAACAGAGATTCCCAAAGAAGAAGATGGTGAAGGACCATC

IRN8|AB863143.1| TCGTATGGATCGAACAGGAGGAACAGAGATTCCCAAAGAAGAAGATGGTGAAGGACCATC

IRN9|AB863144.1| TCGTATGGATCGAACAGGAGGAACGGAGATTCCCAAAGAAGAAGATGGTGAAGGACCATC

IRN10|AB863145.1| TCGTATGGATCGAACAGGAGGAACGGAGATTCCCAAAGAAGAAGATGGTGAAGGACCATC

IRN11|AB863146.1| TCGTATGGATCGAACAGGAGGAACGGAGATTCCCAAAGAAGAAGATGGTGAAGGACCATC

IRN12|AB863147.1| TCGTATGGATCGAACAGGAGGAACGGAGATTCCCAAAGAAGAAGATGGTGAAGGACCATC

IRN13|AB863148.1| TCGTATGGATCGAACAGGAGGAACGGAGATTCCCAAAGAAGAAGATGGTGAAGGACCATC

IRN14|AB863149.1| TCGTATGGATCGAACAGGAGGAACGGAGATTCCTAAAGAAGAAGATGGTGAAGGACCATC

IRN15|AB863150.1| TCGTATGGATCGAACAGGAGGAACGGAGATTCCTAAAGAAGAAGATGGCGGAGAACCATC

IRN16|AB863151.1| TCGTATGGATCGAACAGGAGGAACGGAGATTCCTAAAGAAGAAGATGGCGGAGAACCATC

IRN17|AB863152.1| TCGTATGGATCGAACAGGAGGAACGGAGATTCCCAAAGAAGAAGATGGCGGAGAACCATC

IRN18|AB863153.1| TCGTATGGATCGAACAGGAGGAACGGAGATTCCCAAAGAAGAAGATGGCGGAGAACCATC

IRN19|AB863154.1| TCGTATGGATCGAACAGGAGGAACGGAGATTCCTAAAGAAGAAGATGGCGGAGAACCATC

IRN20|AB863155.1| TCGTATGGATCGAACAGGAGGAACGGAGATTCCCAAAGAAGAAGATGGTGAAGGACCATC

IRN21|AB863156.1| TCGTATGGATCGAACAGGAGGAACGGAGATTCCTAAAGAAGAAGATGGTGAAGGACCATC

JPNHGB340|AB863157.1| TCGAATGGATCGAACAGGAGGAACGGAGATTCCCAAAGAAGAAGATGGT---GAACCATC

JPNKWB778|AB863158.1| TCGAATGGATCGAACAGGAGGAACGGAGATTCCCAAAGAAGAAGATGGT---GAACCATC

JPNM|AB863159.1| TCGAATGGATCGAACAGGAGGAACGGAGATTCCCAAAGAAGAAGATGGT---GAACCATC

JPNN|AB863160.1| TCGAATGGATCGAACAAGAGGAACGGAGTTTCCCAAAGGAGAAGATGGTGAAGGACCATC

JPNS1|AB863161.1| TCGAATGGATCGAACAGGAGGAACGGAGTTTCCCAAAGGAGAAGATGGTGAAGGACCATC

JPNS2|AB863162.1| TCGAATGGATCGAACAGGAGGAACGGAGTTTCCCAAAGGAGAAGATGGTGAAGGACCATC

JPNTKD762|AB863163.1| TCGAATGGATCGAACAGGAGGAACGGAGATTCCCAAAGAAGAAGATGGT---GAACCATC

JPNUV1|AB863164.1| TCGAATGGATCGAACAGGAGGAACGGAGATTCCCAAAGAAGAAGATGGT---GAACCATC

JPNUV26|AB863165.1| TCGAATGGATCGAACAGGAGGAACGGAGATTCCCAAAGAAGAAGATGGT---GAACCATC

TUR1|AB863166.1| TCGTATGGATCGAACAGGAGGAACAGAGATTCCCAAAGAAGAAGATGGCGGAGAACCATC

TUR2|AB863167.1| TCGAATGGATCGAACAGGAGGAACGGAGATTCCTAAAGAAGAAGATGGCGGAGAACCATC

TUR4|AB863168.1| TCGAATGGATCGAACAGGAGGAACGGAGATTCCTAAAGAAGAAGATGGCGGAGAACCATC

TUR5|AB863169.1| TCGTATGGATCGAACAGGAGGAACGGAGATTCCCAAAGAAGAAGATGGCGGAGAACCATC

TUR12|AB863170.1| TCGAATGGATCGAACAGGAGGAACGGAGATTCCCAAAGAAGAGGAT---GGTGAACCATC

TUR34|AB863171.1| TCGAATGGATCGAACAGGAGGAACGGAGATTCCCAAAGAAGAGGAT---GGTGAACCATC

TUR50|AB863172.1| TCGAATGGATCGAACAGGAGGAACGGAGATTCCCAAAGAAGAGGAT---GGTGAACCATC

TUR59|AB863173.1| TCGTATGGATCGAACAGGAGGAACGGAGATTCCCAAAGAAGAAGATGGCGGAGAACCATC

TUR69|AB863174.1| TCGAATGGATCGAACAGGAGGAACGGAGATTCCCAAAGAAGAGGAT---GGTGAACCATC

TUR81|AB863175.1| TCGTATGGATCGAACAGGAGGAACGGAGATTCCCAAAGAAGAAGATGGCGGAGAACCATC

TUR84|AB863176.1| TCGTATGGATCGAACAGGAGGAACGGAGATTCCCAAAGAAGAAGATGGCGGAGAACCATC

TUR94|AB863177.1| TCGCATGGATCGAACACAAGGAACGGAGATTCCCAAAGAAGAGGAT---GGTGAACCATC

TUR213|AB863178.1| TCGTATGGATCGAACAGGAGGAACAGAGATTCCCAAAGAAGAAGAT---GGCGGACCATC

TUR214|AB863179.1| TCGAATGGATCGAACAGGAGGAACGGAGATTCCCAAAGAAGAAGATGGCGGAGAACCATC

TUR216|AB863180.1| TCGAATGGATCGAACAGGAGGAACGGAGATTCCCAAAAAAGAAGATGGCGGAGAACCATC

TUR220|AB863181.1| TCGTATGGATCGAACAGGAGGAACGGAGATTCCCAAAGAAGAAGATGGCGGAGAACCATC

TUR239|AB863182.1| TCGAATGGATCGAACAGGAGGAACAGAGATTCCCAAAGAAGAAGATGGCGGAGAACCATC

TUR244|AB863183.1| TCGAATGGATCGAACAGGAGGAACGGAGATTCCCAAAGAAGAGGATG---GCGAACCATC

TUR249|AB863184.1| TCGTATGGATCGAACAGGAGGAACGGAGATTCCCAAAGAAGAAGATGGCGGAGAACCATC

TUR263|AB863185.1| TCGAATGGATCGAACAGGAGGAACGGAGATTCCCAAAGAAGAGGATGGC---GAACCATC

TUR278|AB863186.1| TCGTATGGATCGAACAGGAGGAACGGAGATTCCCAAAGAAGAAGATGGCGGAGAACCATC

TUR279|AB863187.1| TCGTATGGATCGAACAGGAGGAACGGAGATTCCCAAAGAAGAAGATGGCGGAGAACCATC

TUR285|AB863188.1| TCGTATGGATCGAACAG---GAACGGAGATTCCCAAAGAAGAAGATGGCGGAGAACCATC

TUR289|AB863189.1| TCGCATGGATCGAACACAAGGAACGGAGATTCCTAAAGAAGAAGATG---GTGAACCATC

TUR303|AB863190.1| TCGTATGGATCGAACAGGAGGAACAGAGATTCCCAAAGAAGAAGATGGCGGAGAACCATC

TUR306|AB863191.1| TCGTATGGATCGAACAGGAGGAACGGAGATTCCCAAAGAAGAAGATGGCGGAGAACCATC

Cabb-S|NC_001497.1| TAGATACAATGAGAGAAAGAGAAAGACCCCGGAGGACCGGTACTTTCCAACTCAACCAAA

CRO180A|AB863192.1| TAGATACAATGAGAGAAAGAGAAAGACCACTGAAGACCGGTACTTTCCAACTCAACCAAA

GRC83|AB863193.1| TAGATACAATGAGAGAAAGAGAAAGACCACTGAAGATCGGTACTTTCCAACTCAACCAAA

GRC84B|AB863194.1| TAGATACAATGAGAGAAAGAGAAAGACCACTGAAGATCGGTACTTTCCAACTCAACCAAA

GRC86B|AB863195.1| TAGATACAATGAGAGAAAGAGAAAGACCACTGAAGATCGGTACTTTCCAACTCAACCAAA

GRC86D|AB863196.1| TAGATACAATGAGAGAAAGAGAAAGAGCACTGAAGATCGGTACTTTCCAACTCAACCAAA

GRC87E|AB863197.1| TAGATACAATGAGAGAAAGAGAAAGACCACTGAAGATCGGTACTTTCCAACTCAACCAAA

GRC87G|AB863198.1| TAGATACAATGAGAGAAAGAGAAAGACCACTGAAGATCGGTACTTTCCAACTCAACCAAA

GRC91B|AB863199.1| TAGATACAATGAGAGAAAGAGAAAGACCACTGAAGATCGGTACTTTCCAACTCAACCAAA

GRC92A|AB863200.1| TAGATACAATGAGAGAAAGAGAAAGACCACTGAAGATCGGTATTTTCCAACTCAACCAAA

GRC92C|AB863201.1| TAGATACAATGAGAGAAAGAGAAAGACCACTGAAGATCGGTACTTTCCAACTCAACCAAA

GRC92D|AB863202.1| TAGATACAATGAGAGAAAAAGAAAGACCACTGAAGATCGGTACTTTCCAACTCAACCAAA

IRN1|AB863136.1| TAGATACAATGAGAGAAAGAGAAAGACCACAGAAGATCGGTACTTTCCAACTCAACCAAA

IRN2|AB863137.1| TAGATACAATGAGAGAAAGAGAAAGACCACTGAAGATCGGTACTTTCCAACTCAACCAAA

IRN3|AB863138.1| TAGATACAATGAGAGAAAGAGAAAGACCACAGAAGATCGGTACTTTCCAACTCAACCAAA

IRN4|AB863139.1| TAGATACAATGAGAGAAAGAGAAAGACCACAGAAGATCGGTACTTTCCAACTCAACCAAA

IRN5|AB863140.1| TAGATACAATGAGAGAAAGAGAAAGACCACAGAAGATCGGTACTTTCCAACTCAACCAAA

IRN6|AB863141.1| TAGATACAATGAGAGAAAGAGAAAGACCACAGAAGATCGGTACTTTCCAACTCAACCAAA

IRN7|AB863142.1| TAGATACAACGAGAGAAAGAGAAAGACCACAGAAGATCGGTACTTTCCAACTCAACCAAA

IRN8|AB863143.1| TAGATACAATGAGAGAAAGAGAAAGACCACAGAAGATCGGTACTTTCCAACTCAACCAAA

IRN9|AB863144.1| TAGATACAATGAGAGAAAGAGAAAGACCACAGAAGATCGGTACTTTCCAACTCAACCAAA

IRN10|AB863145.1| TAGATACAATGAGAGAAAGAGAAAGACCACAGAAGATCGGTACTTTCCAACTCAACCAAA

IRN11|AB863146.1| TAGATATAATGAGAGAAAGAGAAAGACCACAGAAGATCGGTACTTTCCAACTCAACCAAA

IRN12|AB863147.1| TAGATACAATGAGAGAAAGAGAAAGACCACAGAAGATCGGTACTTTCCAACTCAACCAAA

IRN13|AB863148.1| TAGATACAATGAGAGAAAGAGAAAGACCACAGAAGATCGGTACTTTCCAACTCAACCAAA

IRN14|AB863149.1| TAGATACAATGAGAGAAAGAGAAAGACCACAGAAGATCGGTACTTTCCAACTCAACCAAA

IRN15|AB863150.1| TAGATACAATGAGAGAAAGAGAAAGACCACTGAAGATCGGTACTTTCCAACTCAACCAAA

IRN16|AB863151.1| TAGATACAATGAGAGAAAGAGAAAGACCACTGAAGATCGGTACTTTCCAACTCAACCAAA

IRN17|AB863152.1| TAGATACAATGAGAGAAAGAGAAAGACCACTGAAGATCGGTACTTTCCAACTCAACCGAA

IRN18|AB863153.1| TAGATACAATGAGAGAAAGAGAAAGACCACAGAAGATCGGTACTTTCCAACTCAACCAAA

IRN19|AB863154.1| TAGATACAATGAGAGAAAGAGAAAGACCACTGAAGATCGGTACTTTCCAACTCAACCAAA

IRN20|AB863155.1| TAGATACAATGAGAGAAAGAGAAAAACCACAGAAGATCGGTACTTTCCAACTCAACCAAA

IRN21|AB863156.1| TAGATACAATGAGAGAAAGAGAAAGACCACAGAAGATCGGTACTTTCCAACTCAACCAAA

JPNHGB340|AB863157.1| TAGATACAATGAGAGAAAGAGAAAGACCCCGGAGGACCGGTACTTTCCAACTCAACCAAA

JPNKWB778|AB863158.1| TAGATACAATGAGAGAAAGAGAAAGACCACGGAGGACCGGTACTTTCCAACTCAACCAAA

JPNM|AB863159.1| TAGATACAATGAGAGAAAGAGAAAGACCCCGGAGGACCGGTACTTTCCAACTCAACCAAA

JPNN|AB863160.1| TAGATACAATGAGAGAAAGAGAAAGACCCCAGAAGATCGGTACTTTCCAACTCAACCAAA

JPNS1|AB863161.1| TAGATACAATGAGAGAAAGAGAAAGACCCCGGAAGACCGGTACTTTCCAACTCAACCAAA

JPNS2|AB863162.1| TAGATACAATGAGAGAAAGAGAAAGACCCCGGAAGACCGGTACTTTCCAACTCAACCAAA

JPNTKD762|AB863163.1| TAGATACAACGAGAGAAAGAGAAAGACCCCGGAGGACCGGTACTTTCCAACTCAACCAAA

JPNUV1|AB863164.1| TAGATACAATGAGAGAAAGAGAAAGACCCCGGAGGACCGGTACTTTCCAACTCAACCAAA

JPNUV26|AB863165.1| TAGATACAATGAGAGAAAGAGAAAGACCCCGGAGGACCGGTACTTTCCAACTCAACCAAA

TUR1|AB863166.1| TAGATACAATGAGAGAAAAAGAAAGACCACGGAAGATCGGTACTTTCCAACTCAACCAAA

TUR2|AB863167.1| TAGATACAATGAGAGAAAGAGAAAGACCACGGAAGATCGGTACTTTCCAACTCAACCAAA

TUR4|AB863168.1| TAGATACAATGAGAGAAAGAGAAAGACCACGGAAGATCGGTACTTTCCAACTCAACCAAA

TUR5|AB863169.1| TAGATACAATGAGAGAAAGAGAAAGACCACTGAAGATCGGTACTTTCCAACTCAACCAAA

TUR12|AB863170.1| CAGATATAATGAGAGAAAGAGAAAGACCCCGGAAGACCGGTACTTTCCAACTCAACCAAA

TUR34|AB863171.1| CAGATACAATGAGAGAAAGAGAAAGACCACTGAAGATCGGTACTTTCCAACTCAACCAAA

TUR50|AB863172.1| CAGATACAATGAGAGAAAGAGAAAGACCACTGAAGATCGGTACTTTCCAACTCAACCAAA

TUR59|AB863173.1| TAGATACAATGAGAGAAAGAGAAAGACCACTGAAGATCGGTACTTTCCAACTCAACCAAA

TUR69|AB863174.1| CAGATACAATGAGAGAAAGAGAAAGACCACGGAAGATCGGTACTTTCCAACTCAACCAAA

TUR81|AB863175.1| TAGATACAATGAGAGAAAGAGAAAGACCACGGAAGATCGGTACTTTCCAACTCAACCAAA

TUR84|AB863176.1| TAGATACAATGAGAGAAAGAGAAAGACCACGGAAGATCGGTACTTTCCAACTCAACCAAA

TUR94|AB863177.1| CAGATATAATGAGAGAAAGAGAAAGACCACGGAGGACCGGTACTTTCCAACTCAACCAAA

TUR213|AB863178.1| TAGATACAATGAGAGAAAAAGAAAGACCACGGAAGATCGGTACTTTCCAACTCAACCAAA

TUR214|AB863179.1| TAGATACAATGAGAGAAAGAGAAAGACCACGGAAGATCGGTACTTTCCAACTCAACCAAA

TUR216|AB863180.1| TAGATACAATGAGAGAAAGAGAAAGACCACTGAAGATCGGTACTTTCCAACTCAACCAAA

TUR220|AB863181.1| TAGATACAATGAGAGAAAGAGAAAGACCACGGAAGATCGGTACTTTCCAACTCAACCAAA

TUR239|AB863182.1| TAGATACAATGAGAGAAAGAGAAAGACCACTGAAGATCGGTACTTTCCAACTCAACCAAA

TUR244|AB863183.1| CAGATATAATGAGAGAAAGAGAAAGACCCCGGAGGACCGGTACTTTCCAACTCAACCAAA

TUR249|AB863184.1| TAGATACAATGAGAGAAAGAGAAAGACCACGGAAGATCGGTACTTTCCAACTCAACCAAA

TUR263|AB863185.1| CAGATACAATGAGAGAAAGAGAAAGACCCCGGAAGACCGGTACTTTCCAACTCAACCAAA

TUR278|AB863186.1| TAGATACAATGAGAGAAAGAGAAAGACCACTGAAGATCGGTACTTTCCAACTCAACCAAA

TUR279|AB863187.1| TAGATACAATGAGAGAAAGAGAAAGACCACGGAAGATCGGTACTTTCCAACTCAACCAAA

TUR285|AB863188.1| TAGATATAATGAGAGAAAGAGAAAGACCACTGAAGACCGGTACTTTCCAACTCAACCAAA

TUR289|AB863189.1| CAGATACAATGAGAGAAAGAGAAAGACCCCGGAAGACCGGTACTTTCCAACTCAACCAAA

TUR303|AB863190.1| TAGATACAATGAGAGAAAGAGAAAGACCACGGAAGATCGGTACTTTCCAACTCAACCAAA

TUR306|AB863191.1| TAGATACAATGAGAGAAAGAGAAAGACCACGGAAGATCGGTACTTTCCAACTCAACCAAA

Cabb-S|NC_001497.1| GACCATTCCAGGACAAAAGCAAACGTCTATGGGAATGCTCAACATTGACTGCCAAACCAA

CRO180A|AB863192.1| GACCATTCCCGGACAAAAGCAAACGACCATGGGAATGCTCAACATTGACTGCCAAGCCAA

GRC83|AB863193.1| GACCATTCCCGGCCAGAAACAAACGACCATGGGAATGCTCAACATTGACTGCCAAGCCAA

GRC84B|AB863194.1| GACCATCCCAGGCCAAAAGCAAACGACCATGGGAATGCTCAATATTGACTGCCAAGCCAA

GRC86B|AB863195.1| GACCATCCCAGGCCAAAAGCAAACGACCATGGGAATGCTCAACATTGACTGCCAAGCCAA

GRC86D|AB863196.1| GACCATTCCCGGCCAAAAACAAACGACCATGGGAATGCTCAATATTGACTGCCAAGCCAA

GRC87E|AB863197.1| GACCATTCCAGGCCAAAAGCAAACGACCATGGGAATGCTCAATATTGACTGCCAAGCCAA

GRC87G|AB863198.1| GACCATTCCAGGCCAAAAGCAAACGACCATGGGAATGCTCAATATTGACTGCCAAGCCAA

GRC91B|AB863199.1| GACCATCCCAGGCCAAAAACAAACGACCATGGGAATGCTCAACATTGACTGCCAAGCCAA

GRC92A|AB863200.1| AACCATTCCAGGTCAAAAGCAAACGACCATGGGAATGCTCAATATTGACTGCCAAGCCAA

GRC92C|AB863201.1| GACCATTCCCGGCCAAAAGCAAACGACCATGGGAATGCTCAATATTGACTGCCAAGCCAA

GRC92D|AB863202.1| GACCATTCCCGGCCAAAAGCAAACGACCATGGGAATGCTCAACATTGACTGCCAAGCCAA

IRN1|AB863136.1| GACCATTCCAGGCCAAAAGCAAACGACCATGGGAATGCTCAACATTGACTGCCAAGCCAA

IRN2|AB863137.1| GACCATCCCAGGTCAAAAACAAACGACCATGGGAATGCTCAACATTGACTGCCAAGCCAA

IRN3|AB863138.1| GACCATTCCAGGCCAAAAGCAAACGACCATGGGAATGCTCAACATTGACTGCCAAGCCAA

IRN4|AB863139.1| GACCATTCCAGGCCAAAAGCAAACGACCATGGGAATGCTCAACATTGACTGCCAAGCCAA

IRN5|AB863140.1| GACCATTCCAGGCCAAAAGCAAACGACCATGGGAATGCTCAACATTGACTGCCAAGCCAA

IRN6|AB863141.1| GACCATTCCAGGCCAAAAGCAAACGACCATGGGAATGCTCAACATTGACTGCCAAGCCAA

IRN7|AB863142.1| GACCATTCCAGGCCAAAAGCAAACGACCATGGGAATGCTCAACATTGACTGCCAAGCCAA

IRN8|AB863143.1| GACCATTCCAGGCCAAAAGCAAACGACCATGGGAATGCTCAACATTGACTGCCAAGCCAA

IRN9|AB863144.1| GACCATTCCAGGCCAAAAACAAACGACCATGGGAATGCTCAACATTGACTGCCAAGCCAA

IRN10|AB863145.1| GACCATCCCAGGTCAAAAGCAAACGACCATGGGAATGCTCAACATTGACTGCCAAGCCAA

IRN11|AB863146.1| GACCATCCCAGGTCAAAAACAAACGACCATGGGAATGCTCAACATTGACTGCCAAGCCAA

IRN12|AB863147.1| GACCATTCCAGGCCAAAAGCAAACGACCGTGGGAATGCTCAACATTGACTGCCAAGCCAA

IRN13|AB863148.1| GACCATTCCAGGCCAAAAGCAAACGACCATGGGAATGCTCAACATTGACTGCCAAGCCAA

IRN14|AB863149.1| GACCATTCCAGGCCAAAAACAAACGACCATGGGAATGCTCAACATTGACTGCCAAGCCAA

IRN15|AB863150.1| GACCATCCCAGGTCAAAAACAAACGACCATGGGAATGCTCAACATTGACTGCCAAGCCAA

IRN16|AB863151.1| GACCATTCCAGGTCAAAAACAAACGACCATGGGAATGCTCAACATTGACTGCCAAGCCAA

IRN17|AB863152.1| GACCATCCCAGGTCAAAAACAAACGACCATGGGAATGCTCAACATTGACTGCCAAGCCAA

IRN18|AB863153.1| GACCATTCCAGGCCAAAAGCAAACGACCATGGGAATGCTCAATATTGACTGCCAAGCCAA

IRN19|AB863154.1| GACCATCCCAGGTCAAAAACAAACGACCATGGGAATGCTCAACATTGACTGCCAAGCCAA

IRN20|AB863155.1| GACCATCCCAGGTCAAAAACAAACGACCATGGGAATGCTCAACATTGACTGCCAAGCCAA

IRN21|AB863156.1| GACCATTCCAGGCCAAAAGCAAACGACCATGGGAATGCTCAACATTGACTGCCAAGCCAA

JPNHGB340|AB863157.1| GACCATCCCAGGACAAAAGCAAACGTCTATGGGAATGCTCAACATTGACTGCCAAACCAA

JPNKWB778|AB863158.1| GACCATCCCAGGACAAAAGCAAACGTCTATGGGAATGCTCAACATTGACTGCCAAACCAA

JPNM|AB863159.1| GACCATCCCAGGACAAAAGCAAACGTCTATGGGAATGCTCAACATTGACTGCCAAACCAA

JPNN|AB863160.1| GACCATTCCAGGACAAAAGCAAACGTCTATGGGAATGCTCAACATTGACTGCCAGACCAA

JPNS1|AB863161.1| GACCATTCCAGGACAAAAGCAAACGTCTATGGGAATGCTCAACATTGACTGCCAAACCAA

JPNS2|AB863162.1| GACCATTCCAGGACAAAAGCAAACGTCTATGGGAATGCTCAACATTGACTGCCAAACCAA

JPNTKD762|AB863163.1| GACCATCCCAGGACAAAAGCAAACGTCTATGGGAATGCTCAACATTGACTGCCAAACCAA

JPNUV1|AB863164.1| GACCATCCCAGGACAAAAGCAAACGTCTATGGGAATGCTCAACATTGACTGCCAAACCAA

JPNUV26|AB863165.1| GACCATCCCAGGACAAAAGCAAACGTCTATGGGAATGCTCAACATTGACTGCCAAACCAA

TUR1|AB863166.1| GACCATTCCAGGCCAAAAGCAAACGACCATGGGAATGCTCAACATTGACTGCCAAGCCAA

TUR2|AB863167.1| GACCATCCCAGGACAAAAGCAAACGACCATGGGAATGCTCAACATTGACTGCCAAGCCAA

TUR4|AB863168.1| GACCATCCCAGGACAAAAGCAAACGACCATGGGAATGCTCAACATTGACTGCCAAGCCAA

TUR5|AB863169.1| GACCATTCCTGGCCAAAAGCAAACGACCATGGGAATACTCAACATTGACTGCCAAGCCAA

TUR12|AB863170.1| GACCATTCCAGGCCAAAAGCAAACAACCATAGGAATGCTCAACATTGACTGCCAAGCCAA

TUR34|AB863171.1| GACCATTCCTGGCCAAAAGCAAACGACCATGGGAATGCTCAACATTGACTGCCAAGCCAA

TUR50|AB863172.1| GACCATTCCCGGCCAAAAGCAAACGACCATGGGAATGCTCAACATTGACTGCCAAACCAA

TUR59|AB863173.1| GACCATTCCAGGCCAAAAGCAAACGACCATGGGAATGCTCAACATTGACTGCCAAGCCAA

TUR69|AB863174.1| GACCATTCCAGGCCAAAAGCAAACGACCATGGGAATGCTCAACATTGACTGCCAAGCCAA

TUR81|AB863175.1| GACCATTCCAGGCCAAAAGCAAACGACCATGGGAATGCTCAACATTGACTGTCAAGCCAA

TUR84|AB863176.1| GACCATTCCAGGCCAAAAGCAAACGACCATGGGAATGCTCAACATTGACTGCCAAGCCAA

TUR94|AB863177.1| GACCATCCCAGGTCAGAAGCAAACGACCATGGGAATGCTCAACATTGACTGCCAAGCCAA

TUR213|AB863178.1| GACCATTCCAGGCCAAAAGCAAACGACCATGGGAATGCTCAACATTGACTGCCAAGCCAA

TUR214|AB863179.1| GACCATTCCAGGTCAAAAACAAACGACCATGGGAATGCTCAACATTGACTGCCAAGCCAA

TUR216|AB863180.1| GACCATTCCAGGCCAAAAGCAAACGACCATGGGAATGCTCAACATTGACTGCCAAGCCAA

TUR220|AB863181.1| GACCATTCCAGGCCAAAAGCAAACGACCATGGGAATGCTCAACATTGACTGCCAAGCCAA

TUR239|AB863182.1| AACCATTCCAGGCCAAAAGCAAACGACCATGGGAATGCTCAACATTGACTGCCAAGCCAA

TUR244|AB863183.1| GACCATTCCAGGCCAAAAGCAAACGACCATTGGAATGCTCAACATTGACTGCCAAGCCAA

TUR249|AB863184.1| GACCATTCCAGGCCAAAAGCAAACGACCATGGGAATGCTCAACATTGACTGCCAAGCCAA

TUR263|AB863185.1| GACCATTCCAGGCCAAAAGCAAACGACCATAGGAATGCTCAACATTGACTGCCAAGCCAA

TUR278|AB863186.1| GACCATTCCAGGCCAAAAGCAAACGACCATGGGAATGCTCAACATTGACTGCCAAGCCAA

TUR279|AB863187.1| GACCATTCCAGGCCAAAAGCAAACGACCATAGGAATGCTCAACATTGACTGCCAAGCCAA

TUR285|AB863188.1| GACCATCCCAGGTCAGAAGCAAACGACCATGGGAATGCTCAACATTGACTGCCAAGCTAA

TUR289|AB863189.1| GACCATTCCAGGCCAAAAGCAAACGACCATAGGAATGCTCAACATTGACTGCCAAGCCAA

TUR303|AB863190.1| GACCATTCCAGGCCAAAAGCAAATGACCATGGGAATGCTCAACATTGACTGCCAAGCCAA

TUR306|AB863191.1| GACCATTCCAGGCCAAAAGCAAACGACCATGGGAATGCTCAACATTGACTGCCAAGCCAA

Cabb-S|NC_001497.1| TCGAAGAACTCTAATCGACGACTGGGCAGCAGAAATCGGATTGATAGTCAAGACCAATAG

CRO180A|AB863192.1| TCGAAGAACTCTAATCGATGATTGGGCAGCAGAAATTGGATTGATAGTCAAGACCAATAG

GRC83|AB863193.1| TCGAAGAACTCTAATCGACGATTGGGCAGCAGAAATCGGATTGATAGTCAAGACCAATAG

GRC84B|AB863194.1| TCGAAGAACTCTAATCGACGATTGGGCAGCAGAGATCGGATTGATAGTCAAAACAAACAG

GRC86B|AB863195.1| TCGAAGAACTCTAATCGACGATTGGGCAGCAGAGATCGGATTGATAGTCAAAACAAACAG

GRC86D|AB863196.1| TCGAAGAACTCTAATCGACGATTGGGCAGCAGAGATCGGATTGATAGTCAAAACAAACAG

GRC87E|AB863197.1| TCGAAGAACTCTAATAGACGATTGGGCAGCAGAGATCGGATTGATAGTCAAAACAAACAG

GRC87G|AB863198.1| TCGAAGAACTCTAATAGACGATTGGGCAGCAGAGATCGGATTGATAGTCAAAACAAACAG

GRC91B|AB863199.1| TCGAAGAACTCTAATCGACGATTGGGCAGCAGAGATCGGATTGATAGTCAAAACCAACAG

GRC92A|AB863200.1| TCGAAGAACTCTAATCGACGATTGGGCAGCAGAGATCGGATTGATAGTCAAAACAAACAG

GRC92C|AB863201.1| TCGAAGAACTCTAATCGACGATTGGGCAGCAGAAATCGGATTAATAGTCAAGACCAATAG

GRC92D|AB863202.1| TCGAAGAACTCTAATCGACGATTGGGCAGCAGAAATCGGATTGATAGTCAAGACCAACAG

IRN1|AB863136.1| TCGAAGAACTCTAATCGACGACTGGGCAGCAGAAATCGGATTGATAGTCAAGACCAACAG

IRN2|AB863137.1| TCGAAGAACTCTAATCGACGACTGGGCAGCAGAAATCGGATTGATAGTCAAGACCAATAG

IRN3|AB863138.1| TCGAAGAACTCTAATCGACGACTGGGCAGCAGAAATCGGATTGATAGTCAAGACCAACAG

IRN4|AB863139.1| TCGTAGAACTCTAATCGACGACTGGGCAGCAGAAATCGGATTGATAGTCAAGACCAACAG

IRN5|AB863140.1| TCGAAGAACTCTAATCGACGACTGGGCAGCAGAAATCGGATTGATAGTCAAGACCAACAG

IRN6|AB863141.1| TCGAAGAACTCTAATCGACGACTGGGCAGCAGAAATCGGATTGATAGTCAAGACCAACAG

IRN7|AB863142.1| TCGAAGAACTCTAATCGACGACTGGGCAGCAGAAATCGGATTGATAGTCAAAACCAACAG

IRN8|AB863143.1| TCGAAGAACTCTAATCGACGACTGGGCAGCAGAAATCGGATTGATAGTCAAGACCAACAG

IRN9|AB863144.1| TCGAAGAACTCTAATCGACGACTGGGCAGCAGAAATCGGATTGATAGTCAAGACCAACAA

IRN10|AB863145.1| TCGAAGAACTCTAATCGACGACTGGGCAGCAGAAATCGGATTGATAGTCAAGACCAACAG

IRN11|AB863146.1| TCGAAGAACTTTAATCGATGATTGGGCAGCAGAAATCGGATTGATAGTCAAGACCAATAG

IRN12|AB863147.1| TCGAAGAACTCTAATCGACGACTGGGCAGCAGAAATCGGATTGATAGTCAAGACCAACAG

IRN13|AB863148.1| TCGAAGAACTCTAATCGACGACTGGGCAGCAGAAATCGGATTGATAGTCAAGACCAACAG

IRN14|AB863149.1| TCGAAGAACTCTAATCGACGACTGGGCAGCAGAAATCGGATTGATAGTCAAAACCAACAG

IRN15|AB863150.1| TCGAAGAACTCTAATCGACGATTGGGCAGCAGAAATCGGATTGATAGTCAAGACCAATAG

IRN16|AB863151.1| TCGAAGAACTCTAATCGATGATTGGGCAGCAGAAATCGGATTGATAGTCAAGACCAATAG

IRN17|AB863152.1| TCGAAGAACTCTAATCGACGATTGGGCAGCAGAAATCGGATTGATAGTCAAGACCAATAG

IRN18|AB863153.1| TCGAAGAACTCTAATCGACGACTGGGCAGCAGAAATCGGATTGATAGTCAAAACCAATAG

IRN19|AB863154.1| TCGAAGAACTCTAATCGATGATTGGGGAGCAGAAATCGGATTGATAGTCAAGACCAATAG

IRN20|AB863155.1| TCGAAGAACTCTAATCGACGATTGGGCAGCAGAAATCGGATTGATAGTCAAGACCAATAG

IRN21|AB863156.1| TCGAAGAACTCTAATCGACGACTGGGCAGCAGAAATCGGATTGATAGTCAAAACCAACAG

JPNHGB340|AB863157.1| TCGAAGAACCTTAATCGATGATTGGGCGGCAGAAATCGGATTGATAGTCAAAACCAATAG

JPNKWB778|AB863158.1| TCGAAGAACCTTAATCGATGATTGGGCAGCAGAAATCGGATTGATAGTCAAAACCAATAG

JPNM|AB863159.1| TCGAAGAACCTTAATCGATGATTGGGCAGCAGAAATCGGATTGATAGTCAAAACCAATAG

JPNN|AB863160.1| TCGAAGAACTTTGATCGATGATTGGGCAGCAGAAATCGGATTGATAGTCAAAACCAACAG

JPNS1|AB863161.1| TCGAAGAACTTTGATCGATGATTGGGCAGCAGAAATCGGATTGATAGTCAAAACCAACAG

JPNS2|AB863162.1| TCGAAGAACTTTGATCGATGATTGGGCAGCAGAAATCGGATTGATAGTCAAAACCAACAG

JPNTKD762|AB863163.1| TCGAAGAACCTTAATCGATGATTGGGCAGCAGAAATCGGATTGATAGTCAAAACCAATAG

JPNUV1|AB863164.1| TCGAAGAACCTTAATCGATGATTGGGCAGCAGAAATCGGATTGATAGTCAAAACCAATAG

JPNUV26|AB863165.1| TCGAAGAACCTTAATCGATGATTGGGCAGCAGAAATCGGATTGATAGTCAAAACCAATAG

TUR1|AB863166.1| TCGAAGAACTCTAATCGACGATTGGGCAGCAGAAATTGGATTGATAGTCAAAACCAATAG

TUR2|AB863167.1| TCGAAGAACTCTAATCGACGACTGGGCAGCAGAAATTGGATTGATAGTCAAAACCAATAG

TUR4|AB863168.1| TCGAAGAACTCTAATCGACGACTGGGCAGCAGAAATTGGATTGATAGTCAAAACCAATAG

TUR5|AB863169.1| TCGAAGAACTCTAATCGATGATTGGGCAGCAGAAATCGGATTGATAGTCAAAACAAACAG

TUR12|AB863170.1| TCGAAGAACTCTAATCGACGATTGGGCAGCAGAAATTGGATTGATAGTCAAAACCAATAG

TUR34|AB863171.1| TCGAAGAACTCTAATCGACGACTGGGCAGCAGAAATTGGATTGATAGTCAAAACCAATAG

TUR50|AB863172.1| TCGAAGAACTCTAATCGACGATTGGGCAGCAGAAATTGGATTGATAGTCAAAACCAATAG

TUR59|AB863173.1| CCGAAGAACTCTAATCGACGATTGGGCAGCAGAAATTGGATTGATAGTCAAAACCAATAG

TUR69|AB863174.1| TCGAAGAACTCTAATCGACGATTGGGCAGCAGAAATTGGATTGATAGTCAAAACCAATAG

TUR81|AB863175.1| TCGAAGAACTCTAATCGACGATTGGGCAGCAGAAATTGGATTGATAGTCAAAACCAATAG

TUR84|AB863176.1| TCGAAGAACTCTAATCGACGATTGGGCAGCAGAAATTGGATTGATAGTCAAAACCAATAG

TUR94|AB863177.1| TCGAAGAACTCTAATCGACGATTGGGCAGCAGAGATCGGATTGATAGTCAAGACCAATAG

TUR213|AB863178.1| TCGAAGAACTCTAATCGACGATTGGGCAGCAGAAATTGGATTGATAGTCAAAACCAATAG

TUR214|AB863179.1| TCGAAGAACTCTAATCGACGATTGGGCAGCAGAAATTGGATTAATAGTCAAAACCAATAG

TUR216|AB863180.1| CCGAAGAACTCTAATCGACGATTGGGCAGCAGAAATAGGATTGATAGTCAAAACCAACAG

TUR220|AB863181.1| TCGAAGAACTCTAATCGACGATTGGGCAGCAGAAATTGGATTGATAGTCAAAACCAATAG

TUR239|AB863182.1| TCGAAGAACTCTAATCGATGACTGGGCAGCAGAAATTGGATTGATAGTCAAAACCAATAG

TUR244|AB863183.1| TCGAAGAACTCTAATCGACGACTGGGCAGCAGAAATTGGATTGATAGTCAAAACCAATAG

TUR249|AB863184.1| TCGAAGAACTCTAATCGACGATTGGGCAGCAGAAATTGGATTGATAGTCAAAACCAACAG

TUR263|AB863185.1| CCGAAGAACTCTAATCGACGATTGGGCAGCAGAAATTGGATTGATAGTCAAAACCAATAG

TUR278|AB863186.1| TCGAAGAACTTTAATCGACGACTGGGCAGCAGAAATTGGATTGATAGTCAAAACCAATAG

TUR279|AB863187.1| TCGAAGAACTCTAATCGACGATTGGGCAGCAGAAATTGGATTGATAGTCAAAACCAATAG

TUR285|AB863188.1| TCGAAGAACTCTAATCGATGATTGGGCAGCAGAAATTGGATTGATAGTCAAAACCAATAG

TUR289|AB863189.1| TCGAAGAACTCTAATCGACGATTGGGCAGCAGAAATTGGATTAATAGTCAAAACCAATAG

TUR303|AB863190.1| TCGAAGAACTCTAATCGACGATTGGGCAGCAGAAATTGGATTGATAGTCAAAACCAATAG

TUR306|AB863191.1| TCGAAGAATTCTAATCGACGATTGGGCAGCAGAAATTGGATTGATAGTCAAAACCAATAG

Cabb-S|NC_001497.1| AGAAGACTATCTCGATCCAGAAACAATTCTACTCTTGATGGAACACAAAACATCAGGAAT

CRO180A|AB863192.1| AGAAGACTATCTTGATCCAGAAACAATCCTACTTCTGATGGAACATAAAACATCAGGAAT

GRC83|AB863193.1| AGAAGACTATCTTGATCCAGAAACAATCCTACTTCTGATGGAACACAAAACATCAGGAAT

GRC84B|AB863194.1| AGAAGACTATCTCGATCCAGAAACAATCCTACTTCTGATGGAACACAAAACATCAGGAAT

GRC86B|AB863195.1| AGAAGACTATCTCGATCCAGAAACAATTCTACTTCTGATGGAACACAAAACATCAGGAAT

GRC86D|AB863196.1| AGAAGACTATCTCGATCCGGAAACAATCCTACTTCTGATGGAACACAAAACATCAGGAAT

GRC87E|AB863197.1| AGAAGACTATCTCGATCCAGAAACAATCCTACTTCTGATGGAACACAAAACATCAGGAAT

GRC87G|AB863198.1| AGAAGACTATCTCGATCCAGAAACAATCCTACTTCTGATGGAACACAAAACATCAGGAAT

GRC91B|AB863199.1| GGAAGACTATCTCGATCCAGAAACAATTCTACTTCTGATGGAACACAAAACATCAGGAAT

GRC92A|AB863200.1| AGAAGACTATCTCGATCCAGAAACAATCCTACTTCTGATGGAACACAAAACATCAGGAAT

GRC92C|AB863201.1| AGAAGACTATCTTGATCCAGAAACAATCCTACTTCTGATGGAACACAAAACATCAGGAAT

GRC92D|AB863202.1| AGAAGACTATCTCGATCCAGAAACAATCCTACTTCTGATGGAACACAAAACATCAGGAAT

IRN1|AB863136.1| AGAAGACTATCTTGATCCAGAAACAATACTACTCTTGATGGAACACAAAACATCAGGAAT

IRN2|AB863137.1| AGAAGACTATCTTGATCCAGAAACAATCCTACTTCTGATGGAACATAAAACATCAGGAAT

IRN3|AB863138.1| AGAAGACTATCTTGATCCAGAAACAATACTACTCTTGATGGAACACAAAACATCAGGAAT

IRN4|AB863139.1| AGAAGACTATCTTGATCCAGAAACAATACTACTCTTGATGGAACACAAAACATCAGGAAT

IRN5|AB863140.1| AGAAGACTATCTTGATCCAGAAACAATACTACTCTTGATGGAACACAAAACATCAGGAAT

IRN6|AB863141.1| AGAAGACTATCTTGATCCAGAAACAATACTACTCTTGATGGAACACAAAACATCAGGAAT

IRN7|AB863142.1| AGAAGACTATCTTGATCCAGAAACAATACTACTCTTGATGGAACACAAAACATCAGGAAT

IRN8|AB863143.1| AGAAGACTATCTTGATCCAGAAACAATACTACTCTTGATGGAACACAAAACATCAGGAAT

IRN9|AB863144.1| AGAAGACTATCTAGATCCAGAAACAATCCTACTTCTGATGGAACATAAAACATCAGGAAT

IRN10|AB863145.1| AGAAGACTATCTTGATCCAGAAACAATACTACTCTTGATGGAACACAAAACATCAGGAAT

IRN11|AB863146.1| AGAAGACTATCTTGATCCAGAAACAATCCTACTTCTGATGGAACATAAAACATCAGGAAT

IRN12|AB863147.1| AGAAGACTATCTTGATCCAGAAACAATACTACTCTTGATGGAACACAAAACATCAGGAAT

IRN13|AB863148.1| AGAAGACTATCTTGATCCAGAAACAATACTACTCTTGATGGAACACAAAACATCAGGAAT

IRN14|AB863149.1| AGAAGACTATCTTGATCCAGAAACAATACTACTCTTGATGGAACACAAAACATCAGGAAT

IRN15|AB863150.1| AGAAGACTATCTAGATCCAGAAACAATCCTACTTCTGATGGAACACAAAACATCAGGAAT

IRN16|AB863151.1| AGAAGACTATCTAGATCCAGAAACAATCCTACTTCTGATGGAACACAAAACATCAGGAAT

IRN17|AB863152.1| AGAAGACTATCTAGATCCAGAAACAATCCTACTTCTGATGGAACACAAAACATCAGGAAT

IRN18|AB863153.1| AGAAGACTATCTTGATCCAGAAACAATACTACTCTTGATGGAACATAAAACATCAGGAAT

IRN19|AB863154.1| AGAAGACTATCTTGATCCAGAAACAATCCTACTTCTGATGGAACATAAAACATCAGGAAT

IRN20|AB863155.1| AGAAGACTATCTAGATCCAGAAACAATCCTACTTCTGATGGAACACAAAACATCAGGAAT

IRN21|AB863156.1| AGAAGACTATCTTGATCCAGAAACAATACTACTCTTGATGGAACACAAAACATCAGGAAT

JPNHGB340|AB863157.1| AGAAGACTATCTCGATCCAGAAACAATACTACTCTTGATGGAACACAAAACATCAGGAAT

JPNKWB778|AB863158.1| AGAAGACTATCTCGATCCAGAAACAATACTACTCTTGATGGAACACAAAACATCAGGAAT

JPNM|AB863159.1| AGAAGACTATCTCGATCCAGAAACAATTCTACTCTTGATGGAACACAAAACATCAGGAAT

JPNN|AB863160.1| AGAAGACTATCTTGATCCAGAAACAATACTACTCTTGATGGAGCACAAGACATCGGGAAT

JPNS1|AB863161.1| AGAAGACTATCTTGATCCAGAAACAATACTACTCTTGATGGAACACAAGACATCGGGAAT

JPNS2|AB863162.1| AGAAGACTATCTTGATCCAGAAACAATACTACTCTTGATGGAACACAAGACATCGGGAAT

JPNTKD762|AB863163.1| AGAAGACTATCTCGATCCAGAAACAATACTACTCTTGATGGAACACAAAACATCAGGAAT

JPNUV1|AB863164.1| AGAAGACTATCTTGATCCAGAAACAATACTACTCTTGATGGAACACAAAACATCAGGAAT

JPNUV26|AB863165.1| AGAAGACTATCTCGATCCAGAAACAATACTACTCTTGATGGAACACAAAACATCAGGAAT

TUR1|AB863166.1| AGAAGACTATCTTGATCCAGAAACAATTCTACTCTTGATGGAACACAAAACATCAGGAAT

TUR2|AB863167.1| AGAAGACTATCTCGATCCAGAAACAATTCTACTCTTGATGGAGCATAAAACATCAGGAAT

TUR4|AB863168.1| AGAAGACTATCTCGATCCAGAAACAATTCTACTCTTGATGGAGCATAAAACATCAGGAAT

TUR5|AB863169.1| AGAAGACTATCTTGATCCAGAAACAATCCTACTTCTGATGGAACACAAAACATCAGGAAT

TUR12|AB863170.1| AGAAGACTATCTTGATCCAGAAACAATTCTACTTCTGATGGAACACAAAACATCAGGAAT

TUR34|AB863171.1| AGAAGACTATCTTGATCCGGAAACAATTCTACTTCTGATGGAACACAAAACATCAGGAAT

TUR50|AB863172.1| AGAAGACTATCTGGATCCAGAAACAATCCTACTCCTGATGGAACACAAAACATCAGGAAT

TUR59|AB863173.1| AGAAGACTATCTCGATCCAGAAACAATTCTACTCTTGATGGAACACAAAACATCAGGGAT

TUR69|AB863174.1| AGAAGACTATCTTGATCCAGAAACAATTCTACTCTTGATGGAACACAAAACATCAGGAAT

TUR81|AB863175.1| AGAAGACTATCTTGATCCAGAAACAATTCTACTCTTGATGGAACACAAAACATCAGGAAT

TUR84|AB863176.1| AGAAGACTATCTTGATCCAGAAACAATTCTACTCTTGATGGAACACAAAACATCAGGAAT

TUR94|AB863177.1| AGAAGACTATCTTGATCCAGAAACAATTCTACTTCTGATGGAACACAAAACATCAGGAAT

TUR213|AB863178.1| AGAAGACTATCTTGATCCAGAAACAATTCTACTCTTGATGGAACACAAAACATCAGGAAT

TUR214|AB863179.1| AGAAGACTATCTCGATCCAGAAACAATTCTACTCTTGATGGAGCATAAAACATCAGGAAT

TUR216|AB863180.1| AGAAGACTATCTTGATCCAGAAACAATTCTACTCTTGATGGAACACAAAACATCAGGAAT

TUR220|AB863181.1| AGAAGACTATCTTGATCCAGAAACAATTCTACTCTTGATGGAACACAAAACATCAGGAAT

TUR239|AB863182.1| AGAAGACTATCTCGATCCAGAAACAATTCTACTCTTGATGGAGCATAAAACATCAGGAAT

TUR244|AB863183.1| AGAAGACTATCTTGATCCAGAAACAATTCTACTCTTGATGGAACACAAAACATCAGGAAT

TUR249|AB863184.1| AGAAGACTATCTTGATCCAGAAACAATTCTACTCTTGATGGAACACAAAACATCAGGAAT

TUR263|AB863185.1| AGAAGACTATCTCGATCCAGAAACAATTCTACTCTTGATGGAACACAAAACATCAGGAAT

TUR278|AB863186.1| AGAAGACTATCTTGATCCAGAAACAATTCTACTCTTGATGGAACACAAAACATCAGGAAT

TUR279|AB863187.1| AGAAGACTATCTTGATCCAGAAACAATCCTACTCTTGATGGAACACAAAACATCAGGAAT

TUR285|AB863188.1| AGAAGACTATCTCGATCCAGAAACAATCCTACTTCTGATGGAACATAAAACATCAGGAAT

TUR289|AB863189.1| AGAAGACTATCTTGATCCAGAAACAATCCTACTCTTGATGGAACACAAAACATCAGGAAT

TUR303|AB863190.1| AGAAGACTATCTTGATCCAGAAACAATTCTACTCTTGATGGAACACAAAACATCAGGAAT

TUR306|AB863191.1| AGAAGACTATCTTGATCCAGAAACAATTCTACTCTTGATGGAACACAAAACATCAGGAAT

Cabb-S|NC_001497.1| AGCCAAGGAGTTAATCCGAAATACAAGATGGAACCGCACTACCGGAGACATCATAGAACA

CRO180A|AB863192.1| AGCCAAGGAGTTAATCCGAAACACAAGATGGAACCGCACTACCGGCGACATCATAGAACA

GRC83|AB863193.1| AGCCAAGGAGTTAATCCGAAACACAAGATGGAACCGCACTACCGGCGACATCATAGAACA

GRC84B|AB863194.1| AGCCAAGGAGTTAATCCGAAATACAAGATGGAACCGCACTACCGGCGACATCATAGAACA

GRC86B|AB863195.1| AGCCAAGGAGTTAATCCGAAACACAAGATGGAACCGCACTACCGGCGACATCATAGAACA

GRC86D|AB863196.1| AGCCAAGGAGTTAATCCGAAACACAAGATGGAACCGCACTACCGGCGACATCATAGAACA

GRC87E|AB863197.1| AGCCAAGGAGTTAATCCGAAACACAAGATGGAACCGCACTACCGGCGACATCATAGAACA

GRC87G|AB863198.1| AGCCAAGGAGTTAATCCGAAACACAAGATGGAACCGCACTACCGGCGACATCATAGAACA

GRC91B|AB863199.1| AGCCAAGGAGTTAATCCGAAACACAAGATGGAACCGCACTACCGGCGACATCATAGAACA

GRC92A|AB863200.1| AGCCAAGGAGTTAATCCGAAACACAAGATGGAACCGCACTACCGGCGACATCATAGAACA

GRC92C|AB863201.1| AGCCAAGGAGTTAATCCGAAACACAAGATGGAACCGCACTACCGGCGACATCATAGAACA

GRC92D|AB863202.1| AGCCAAGGAGTTAATCCGAAACACAAGATGGAACCGCACTACCGGCGACATCATAGAACA

IRN1|AB863136.1| AGCCAAGGAGTTAATCCGAAATACAAGATGGAACCGCACTACCGGCGATATCGTAGAACA

IRN2|AB863137.1| AGCCAAGGAGTTAATCCGAAACACAAGATGGAACCGCACTACCGGCGACATCATAGAACA

IRN3|AB863138.1| AGCCAAGGAGTTAATCCGAAATACAAGATGGAACCGCACTACCGGCGATATCGTAGAACA

IRN4|AB863139.1| AGCCAAGGAGTTAATCCGAAATACAAGATGGAACCGTACTACCGGCAATATCTTAGAACA

IRN5|AB863140.1| AGCCAAGGAGTTAATCCGAAATACAAGATGGAACCGTACTACCGGCGATATCATAGAACA

IRN6|AB863141.1| AGCCAAGGAGTTAATCCGAAATACAAGATGGAACCGTACTACCGGCGATATCATAGAACA

IRN7|AB863142.1| AGCCAAGGAGTTAATCCGAAATACAAGATGGAACCGTACTACCGGCGATATCATAGAACA

IRN8|AB863143.1| AGCCAAGGAGTTAATCCGAAATACAAGATGGAACCGTACTACCGGCGATATCATAGAACA

IRN9|AB863144.1| AGCCAAGGAGTTAATCCGAAACACAAGATGGAACCGCACTACCGGCGACATCATAGAACA

IRN10|AB863145.1| AGCCAAGGAGTTAATCCGAAATACAAGATGGAACCGTACTACCGGCGATATCATAGAACA

IRN11|AB863146.1| AGCCAAGGAGTTAATCCGAAACACAAGATGGAACCGCACTACCGGCGACATCATAGAACA

IRN12|AB863147.1| AGCCAAGGAGTTAATCCGAAATACAAGATGGAACCGTACTACCGGCGATATCATAGAACA

IRN13|AB863148.1| AGCCAAGGAGTTAATCCGAAATACAAGATGGAACCGTACTACCGGCGATATCATAGAACA

IRN14|AB863149.1| AGCCAAGGAGTTAATCCGAAATACAAGATGGAACCGTACTACCGGCGATATCATAGAACA

IRN15|AB863150.1| AGCCAAGGAGTTAATCCGAAACACAAGATGGAACCGCACTACCGGCGACATCATAGAACA

IRN16|AB863151.1| AGCCAAGGAGTTAATCCGAAACACAAGATGGAACCGCACTACCGGCGACATCATAGAACA

IRN17|AB863152.1| AGCCAAGGAGTTAATCCGAAACACAAGATGGAACCGCACTACCGGCGACATCATAGAACA

IRN18|AB863153.1| AGCCAAGGAGTTAATCCGAAACACAAGATGGAACCGCACTACCGGCGATATCATAGAACA

IRN19|AB863154.1| AGCCAAGGAGTTAATCCGAAACACAAGATGGAACCGCACTACCGGCGACATCATAGAACA

IRN20|AB863155.1| AGCCAAGGAGTTAATCCGAAACACAAGATGGAACCGCACTACCGGCGACATCATAGAACA

IRN21|AB863156.1| AGCCAAGGAGTTAATCCGAAATACAAGATGGAACCGTACTACCGGAGATATCATAGAACA

JPNHGB340|AB863157.1| AGCCAAGGAGTTAATCCGAAATACAAGATGGAACCGTACTACCGGCGATATCATAGAACA

JPNKWB778|AB863158.1| AGCCAAGGAGTTAATCCGAAATACAAGATGGAACCGTACTACCGGCGATATCATAGAACA

JPNM|AB863159.1| AGCCAAGGAGTTAATCCGAAATACAAGATGGAACCGTACTACCGGCGATATCATAGAACA

JPNN|AB863160.1| AGCCAAGGAATTAATCCGAAATACAAGATGGAACCGGACTACTGGAGACATCTTAGAACA

JPNS1|AB863161.1| AGCCAAGGAATTAATCCGAAATACAAGATGGAACCGGACTACTGGAGACATCTTAGAACA

JPNS2|AB863162.1| AGCCAAGGAATTAATCCGAAATACAAGATGGAACCGGACTACTGGAGACATCTTAGAACA

JPNTKD762|AB863163.1| AGCCAAGGAGTTAATCCGAAATACAAGATGGAACCGTACTACCGGCGATATCATAGAACA

JPNUV1|AB863164.1| AGCCAAGGAGTTAATCCGAAATACAAGATGGAACCGTACTACCGGCGATATCATAGAACA

JPNUV26|AB863165.1| AGCCAAGGAGTTAATCCGAAATACAAGATGGAACCGTACTACCGGCGATATCATAGAACA

TUR1|AB863166.1| AGCCAAGGAGTTAATCCGAAATACAAGATGGAACCGTACCACCGGCGACATCATAGAACA

TUR2|AB863167.1| AGCCAAGGAGTTAATCCGAAATACAAGATGGAACCGCACTACCGGCGACATCATAGAACA

TUR4|AB863168.1| AGCCAAGGAGTTAATCCGAAATACAAGATGGAACCGCACTACCGGCGACATCATAGAACA

TUR5|AB863169.1| AGCCAAGGAGCTAATCCGAAACACAAGATGGAACCGCACTACCGGCGACATCATAGAACA

TUR12|AB863170.1| AGCCAAGGAGTTAATCCGAAACACAAGATGGAACCGCACTACCGGCGACATCATAGAACA

TUR34|AB863171.1| AGCCAAGGAGTTAATCCGAAACACAAGATGGAACCGCACTACCGGCGACATCATAGAACA

TUR50|AB863172.1| AGCCAAGGAGTTAATCCGAAACACAAGATGGAACCGTACTACCGGCGACATCATAGAACA

TUR59|AB863173.1| AGCCAAGGAGTTAATCCGAAACACAAGATGGAACCGCACTACCGGCGACATCATAGAACA

TUR69|AB863174.1| AGCCAAGGAGTTAATCCGAAATACAAGATGGAACCGCACTACCGGCGACATCATAGAACA

TUR81|AB863175.1| AGCCAAGGAGTTAATCCGAAATACAAGATGGAACCGCACTACCGGCGACATCATAGAACA

TUR84|AB863176.1| AGCCAAGGAGTTAATCCGAAATACAAGATGGAACCGCACTACCGGCGACATCATAGAACA

TUR94|AB863177.1| AGCCAAGGAGTTAATCCGAAACACAAGATGGAACCGCACTACCGGCGACATCATAGAACA

TUR213|AB863178.1| AGCCAAGGAGTTAATCCGAAATACAAGATGGAACCGCACTACTGGCGACATCATAGAACA

TUR214|AB863179.1| AGCCAAGGAGTTAATCCGAAATACAAGATGGAACCGCACTACCGGCGACATCATAGAGCA

TUR216|AB863180.1| AGCCAAGGAGTTAATCCGAAACACAAGATGGAACCGTACCACCGGCGATATCATAGAACA

TUR220|AB863181.1| AGCCAAGGAGTTAATCCGAAATACAAGATGGAACCGCACTACCGGCGACATCATAGAACA

TUR239|AB863182.1| AGCCAAGGAGTTAATCCGAAATACAAGATGGAACCGCACTACCGGCGACATCATAGAGCA

TUR244|AB863183.1| AGCCAAGGAGTTAATCCGAAATACAAGATGGAACCGTACTACCGGCGACATCATAGAACA

TUR249|AB863184.1| AGCCAAGGAGTTAATCCGAAATACAAGATGGAACCGCACTACCGGCGACATCATAGAACA

TUR263|AB863185.1| AGCCAAGGAGTTAATCCGAAATACAAGATGGAACCGTACTACCGGCGACATCATAGAACA

TUR278|AB863186.1| AGCCAAGGAGTTAATCCGAAATACAAGATGGAACCGCACTACCGGCGACATCATAGAACA

TUR279|AB863187.1| AGCCAAGGAGTTAATCCGAAATACAAGATGGAACCGCACTACCGGCGACATCATAGAACA

TUR285|AB863188.1| AGCCAAGGAGTTAATCCGAAACACAAGATGGAACCGCACTACCGGCGACATCATAGAACA

TUR289|AB863189.1| AGCCAAGGAGTTAATCCGAAATACAAGATGGAACCGCACTACCGGCGACATCATAGAACA

TUR303|AB863190.1| AGCCAAGGAGTTAATCCGAAATACAAGATGGAACCGCACTACCGGCGACATCATAGAACA

TUR306|AB863191.1| AGCCAAGGAGTTAATCCGAAATACAAGATGGAACCGTACCACTGGCGACATCATAGAACA

Cabb-S|NC_001497.1| GGTGATCGATGCGATGTACACCATGTTCTTAGGACTAAACTACTCCGACAACAAAGTTGC

CRO180A|AB863192.1| GGTGATCGATGCGATGTACACCATGTTCTTAGGATTAAATTACTCCGACAACAAGGTTGC

GRC83|AB863193.1| GGTGATCGATGCAATGTACACCATGTTCTTAGGATTAAACTACTCCGACAACAAGGTTGC

GRC84B|AB863194.1| GGTGATCGATGCAATGTACACCATGTTCTTAGGATTAAACTACTCCGACAACAAGGTTGC

GRC86B|AB863195.1| GGTGATCGATGCAATGTACACCATGTTCTTAGGATTAAACTACTCCGACAACAAGGTTGC

GRC86D|AB863196.1| GGTGATCAATGCAATGTACACCATGTTCTTAGGATTAAACTACTCCGACAACAAGGTTGC

GRC87E|AB863197.1| GGTGATCAATGCAATGTACACCATGTTCTTAGGATTAAACTACTCCGACAACAAGGTTGC

GRC87G|AB863198.1| GGTGATCGATGCAATGTACACCATGTTCTTAGGATTAAACTACTCCGACAACAAGGTTGC

GRC91B|AB863199.1| GGTGATCAATGCCATGTACACCATGTTCTTAGGATTAAACTACTCCGACAACAAGGTTGC

GRC92A|AB863200.1| GGTGATCAATGCAATGTACACCATGTTCTTAGGATTAAACTACTCCGACAACAAGGTTGC

GRC92C|AB863201.1| GGTGATCGATGCAATGTACACCATGTTCTTAGGATTAAATTACTCCGACAACAAGGTTGC

GRC92D|AB863202.1| GGTGATCGATGCAATGTACACCATGTTCTTAGGATTAAACTACTCCGACAACAAGGTTGC

IRN1|AB863136.1| GGTGATCGATGCAATGTACACCATGTTCTTAGGACTTAACTACTCCGACAACAAGGTTGC

IRN2|AB863137.1| GGTGATCGATGCGATGTACACCATGTTCTTAGGATTAAACTACTCCGACAACAAGGTTGC

IRN3|AB863138.1| GGTGATCGATGCAATGTACACCATGTTCTTAGGACTTAACTACTCCGACAACAAGGTTGC

IRN4|AB863139.1| GGTGATCGATGCGATGTACACCATGTTCTTAGGACTTAACTACTCCGACAACAAGGTTGC

IRN5|AB863140.1| GGTGATCAATGCGATGTACACCATGTTCTTAGGACTTAACTACTCCGACAACAAGGTTGC

IRN6|AB863141.1| GGTGATCGATGCGATGTACACCATGTTCTTAGGACTTAACTACTCCGACAACAAGGTTGC

IRN7|AB863142.1| GGTGATCGATGCGATGTACACCATGTTCTTAGGACTTAACTACTCCGACAACAAGGTTGC

IRN8|AB863143.1| GGTGATCGATGCGATGTACACCATGTTCTTAGGACTTAACTACTCCGACAACAAGGTTGC

IRN9|AB863144.1| GGTGATCGATGCAATGTACACAATGTTCTTAGGATTAAACTACTCCGACAACAAGGTTGC

IRN10|AB863145.1| GGTGATCGATGCGATGTACACCATGTTCTTAGGACTTAACTACTCCGACAACAAGGTTGC

IRN11|AB863146.1| GGTGATCGATGCGATGTACACCATGTTCTTAGGATTAAACTACTCCGACAACAAGGTTGC

IRN12|AB863147.1| GGTGATCGATGCGATGTACACCATGTTCTTAGGACTTAACTACTCCGACAACAAGGTTGC

IRN13|AB863148.1| GGTGATCGATGCGATGTACACCATGTTCTTAGGACTTAACTACTCCGACAACAAGGTTGC

IRN14|AB863149.1| GGTGATCGATGCGATGTACACCATGTTCTTAGGACTTAACTACTCCGACAACAAGGTTGC

IRN15|AB863150.1| GGTGATCGATGCAATGTACACCATGTTCTTAGGATTAAACTACTCCGACAACAAGGTTGC

IRN16|AB863151.1| GGTGATCGATGCAATGTACACCATGTTCTTAGGATTTAACTACTCCGACAACAAGGTTGC

IRN17|AB863152.1| GGTGATCGATGCAATGTACACCATGTTCTTAGGATTAAACTACTCCGACAACAAGGTTGC

IRN18|AB863153.1| GGTGATCGATGCGATGTACACCATGTTCTTAGGACTTAACTACTCCGACAACAAGGTTGC

IRN19|AB863154.1| GGTGATCGATGCGATGTACACCATGTTCTTAGGATTAAACTACTCCGACAACAAGGTTGC

IRN20|AB863155.1| GGTGATCGATGCAATGTACACCATGTTCTTAGGATTAAACTACTCCGACAACAAGGTTGC

IRN21|AB863156.1| GGTGATCGATGCGATGTACACCATGTTCTTAGGACTTAACTACTCCGACAACAAGGTTGC

JPNHGB340|AB863157.1| GGTGATCAATGCGATGTACACCATGTTCTTAGGACTTAACTACTCCGACAACAAGGTTGC

JPNKWB778|AB863158.1| GGTGATCGATGCAATGTACACCATGTTCTTAGGACTTAACTACTCCGACAACAAGGTTGC

JPNM|AB863159.1| GGTGATCAATGCAATGTACACCATGTTCTTAGGACTTAACTACTCCGACAACAAGGTTGC

JPNN|AB863160.1| GGTGATCGATGCGATGTACACCATGTTCTTAGGACTTAACTACTCCGACAACAAGGTTGC

JPNS1|AB863161.1| GGTGATCGATGCGATGTACACCATGTTCTTAGGACTTAACTACTCCGACAACAAGGTTGC

JPNS2|AB863162.1| GGTGATCGATGCGATGTACACCATGTTCTTAGGACTTAACTACTCCGACAACAAGGTTGC

JPNTKD762|AB863163.1| GGTGATCGATGCAATGTACACCATGTTCTTAGGACTTAACTACTCCGACAACAAGGTTGC

JPNUV1|AB863164.1| GGTGATCAATGCAATGTACACCATGTTCTTAGGACTTAACTACTCCGACAACAAGGTTGC

JPNUV26|AB863165.1| GGTGATCAATGCAATGTACACCATGTTCTTAGGACTTAACTACTCCGACAACAAGGTTGC

TUR1|AB863166.1| GGTGATCAATGCAATGTACACCATGTTCTTAGGATTGAACTACTCCGACAACAAGGTTGC

TUR2|AB863167.1| GGTGATCGATGCGATGTACACCATGTTCCTAGGATTAAACTACTCCGACAACAAGGTTGC

TUR4|AB863168.1| GGTGATCGATGCGATGTACACCATGTTCCTAGGATTAAACTACTCCGACAACAAGGTTGC

TUR5|AB863169.1| GGTGATCAATGCGATGTACACCATGTTCTTAGGATTAAACTACTCCGACAACAAGGTTGC

TUR12|AB863170.1| AGTGATCGATGCAATGTACACCATGTTCTTAGGATTAAACTACTCCGACAACAAGGTTGC

TUR34|AB863171.1| GGTGATCGATGCAATGTACACCATGTTCTTAGGATTAAACTACTCCGACAACAAGGTTGC

TUR50|AB863172.1| GGTGATCGATGCGATGTACACCATGTTCTTAGGATTAAACTACTCCGACAACAAGGTTGC

TUR59|AB863173.1| GGTGATCGATGCGATGTACACCATGTTCTTAGGATTAAACTACTCCGACAACAAGGTTGC

TUR69|AB863174.1| AGTGATCGATGCGATGTACACCATGTTCTTAGGATTAAACTACTCCGACAACAAGGTTGC

TUR81|AB863175.1| GGTGATCGATGCGATGTACACCATGTTCTTAGGATTAAACTACTCCGACAACAAGGTTGC

TUR84|AB863176.1| AGTGATCGATGCGATGTACACCATGTTCTTAGGATTAAACTACTCCGACAACAAGGTTGC

TUR94|AB863177.1| GGTGATCGATGCGATGTACACCATGTTCTTAGGATTAAACTACTCCGACAACAAGGTTGC

TUR213|AB863178.1| GGTGATCGATGCGATGTACACCATGTTCTTAGGATTAAACTACTCCGACAACAAGGTTGC

TUR214|AB863179.1| GGTGATCGATGCGATGTACACCATGTTCTTAGGATTAAATTACTCCGACAACAAGGTTGC

TUR216|AB863180.1| GGTGATCGATGCAATGTACACCATGTTCTTAGGATTAAACTACTCCGACAACAAGGTTGC

TUR220|AB863181.1| GGTGATCGATGCGATGTACACCATGTTCTTAGGATTAAACTACTCCGACAACAAGGTTGC

TUR239|AB863182.1| GGTGATCGATGCGATGTACACCATGTTCTTAGGATTAAATTACTCCGACAACAAGGTTGC

TUR244|AB863183.1| GGTGATCAATGCAATGTACACCATGTTCTTAGGATTAAACTACTCCGACAACAAGGTTGC

TUR249|AB863184.1| GGTGATCAATGCGATGTACACCATGTTCCTAGGATTAAACTACTCCGACAACAAGGTTGC

TUR263|AB863185.1| GGTGATCGATGCGATGTACACCATGTTCTTAGGATTAAACTACTCCGACAACAAGGTTGC

TUR278|AB863186.1| GGTGATCGATGCGATGTACACCATGTTCTTAGGATTAAACTACTCCGACAACAAGGTTGC

TUR279|AB863187.1| GGTGATCGATGCGATGTACACCATGTTCTTAGGATTAAACTACTCCGACAACAAGGTTGC

TUR285|AB863188.1| GGTGATCGATGCAATGTACACCATGTTCTTAGGATTAAACTACTCCGACAACAAGGTTGC

TUR289|AB863189.1| GGTGATCGATGCGATGTACACCATGTTCTTAGGATTAAACTACTCCGACAACAAGGTTGC

TUR303|AB863190.1| GGTGATCGATGCGATGTACACCATGTTCTTAGGATTAAACTACTCCGACAACAAGGTTGC

TUR306|AB863191.1| GGTGATCGATGCAATGTACACCATGTTCTTAGGATTGAACTACTCCGACAACAAGGTTGC

Cabb-S|NC_001497.1| TGAGAAGATTGACGAGCAAGAGAAGGCCAAGATCAGAATGACCAAGCTCCAGCTCTGCGA

CRO180A|AB863192.1| TGAGAAGATCGAAGAGCAAGAGAAGGCCAAAATCAGAATGACCAAGCTTCAGCTCTGCGA

GRC83|AB863193.1| TGAAAAGATCGAAGAGCAAGAGAAGGCCAAAATCAGAATGACCAAGCTTCAGCTCTGCGA

GRC84B|AB863194.1| TGAGAAGATCGAAGAGCAAGAGAAGGCCAAAATCAGAATGACCAAGCTTCAGCTCTGCGA

GRC86B|AB863195.1| TGAGAAGATCGAAGAGCAAGAGAAGGCCAAAATCAGAATGACCAAGCTTCAGCTCTGCGA

GRC86D|AB863196.1| TGAGAAGATCGAAGAGCAAGAGAAGGCCAAAATCAGAATGACCAAGCTTCAGCTCTGCGA

GRC87E|AB863197.1| TGAGAAGATCGAAGAGCAAGAGAAGGCCAAAATCAGAATGACCAAGCTTCAGCTCTGCGA

GRC87G|AB863198.1| TGAGAAGATCGAAGAGCAAGAGAAGGCCAAAATCAGAATGACCAAGCTTCAGCTCTGCGA

GRC91B|AB863199.1| TGAGAAGATCGAAGAGCAAGAAAAGGCCAAAATCAGAATGACCAAGCTTCAGCTCTGCGA

GRC92A|AB863200.1| TGAGAAGATCGAAGAGCAAGAGAAGGCCAAAATCAGAATGACCAAGCTTCAGCTCTGCGA

GRC92C|AB863201.1| TGAGAAGATCGAAGAGCAAGAGAAGGCCAAAATCAGAATGACCAAGCTTCAGCTCTGCGA

GRC92D|AB863202.1| TGAGAAGATCGAAGAGCAAGAGAAGGCCAAAATCAGAATGACCAAGCTTCAGCTCTGCGA

IRN1|AB863136.1| CGAGAAGATTGAAGAGCAAGAGAAGGCCAAGATCAGAATGACCAAGCTTCAGCTCTGCGA

IRN2|AB863137.1| TGAGAAGATCGAAGAGCAAGAGAAGGCCAAAATCAGAATGACCAAGCTTCAGCTCTGCGA

IRN3|AB863138.1| CGAGAAGATTGAAGAGCAAGAGAAGGCCAAGATCAGAATGACCAAGCTTCAGCTCTGCGA

IRN4|AB863139.1| CGAAAAGATTGAAGAGCAAGAGAAGGCCAAGATCAGAATGACCAAACTCCAGCTCTGCGA

IRN5|AB863140.1| CGAGAAGATTGAAGAGCAAGAGAAGGCCAAGATCAGAATGACCAAACTCCAGCTCTGCGA

IRN6|AB863141.1| CGAGAAGATTGAAGAGCAAGAGAAGGCCAAGATCAGAATGACCAAACTCCAGCTCTGCGA

IRN7|AB863142.1| CGAGAAGATTGAAGAGCAAGAGAAGGCCAAGATCAGAATGACCAAACTCCAGCTCTGCGA

IRN8|AB863143.1| CGAGAAGATTGAAGAGCAAGAGAAGGCCAAGATCAGAATGACCAAACTCCAGCTCTGCGA

IRN9|AB863144.1| CGAGAAGATTGAAGAGCAAGAAAAGGCCAAGATCAGAATGACCAAACTCCAGCTCTGCGA

IRN10|AB863145.1| CGAGAAGATTGAAGAGCAAGAGAAGGCCAAGATCAGAATGACCAAACTCCAGCTCTGCGA

IRN11|AB863146.1| TGAGAAGATCGAAGAGCAAGAGAAGGCCAAAATCAGAATGACCAAGCTTCAGCTCTGCGA

IRN12|AB863147.1| CGAGAAGATTGAAGAGCAAGAGAAGGCCAAGATAAGAATGACCAAACTCCAGCTCTGCGA

IRN13|AB863148.1| CGAGAAGATTGAAGAGCAAGAGAAGGCCAAGATCAGAATGACCAAACTCCAGCTCTGCGA

IRN14|AB863149.1| CGAGAAGATTGAAGAGCAAGAGAAGGCCAAGATCAGAATGACCAAACTCCAGCTCTGCGA

IRN15|AB863150.1| TGAGAAGATCGAAGAGCAAGAGAAGGCCAAGATTAGAATGACCAAGCTTCAGCTCTGCGA

IRN16|AB863151.1| TGAGAAGATCGAAGAGCAAGAGAAGGCCAAGATTAGAATGACCAAGCTTCAGCTCTGCGA

IRN17|AB863152.1| TGAGAAGATCGAAGAGCAAGAGAAGGCCAAGATTAGAATGACCAAGCTTCAGCTCTGCGA

IRN18|AB863153.1| CGAGAAGATTGAAGAGCAAGAGAAGGCCAAGATCAGAATGACCAAGCTTCAGCTCTGCGA

IRN19|AB863154.1| TGAGAAGATCGAAGAGCAAGAGAAGGCCAAAATCAGAATGACCAAGCTTCAGCTCTGCGA

IRN20|AB863155.1| TGAGAAGATCGAAGAGCAAGAGAAGGCCAAGATTAGAATGACCAAGCTTCAGCTCTGCGA

IRN21|AB863156.1| CGAGAAGATTGAAGAGCAAGAGAAGGCCAAGATCAGAATGACCAAACTCCAGCTCTGCGA

JPNHGB340|AB863157.1| TGAGAAGATAGACGAGCAAGAGAAGGCCAAGATCAGAATGACCAAGCTCCAGCTCTGCGA

JPNKWB778|AB863158.1| TGAGAAGATAGACGAGCAAGAGAAGGCCAAGATCAGAATGACCAAGCTCCAGCTCTGCGA

JPNM|AB863159.1| TGAGAAGATAGACGAGCAAGAGAAGGCCAAGATCAGAATGACCAAGCTCCAGCTCTGCGA

JPNN|AB863160.1| TGAAAAGATAGACGAGCAAGAGAAGGCCAAGATCAGAATGACCAAGCTCCAGCTCTGCGA

JPNS1|AB863161.1| TGAAAAGATAGACGAGCAAGAGAAGGCCAAGATCAGAATGACCAAGCTCCAGCTCTGCGA

JPNS2|AB863162.1| TGAAAAGATAGACGAGCAAGAGAAGGCCAAGATCAGAATGACCAAGCTCCAGCTCTGCGA

JPNTKD762|AB863163.1| TGAGAAGATAGAAGAGCAAGAGAAGGCCAAGATCAGAATGACCAAGCTCCAGCTCTGCGA

JPNUV1|AB863164.1| TGAGAAGATAGACGAGCAAGAGAAGGCCAAGATCAGAATGACCAAGCTCCAGCTCTGCGA

JPNUV26|AB863165.1| TGAGAAGATAGACGAGCAAGAGAAGGCCAAGATCAGAATGACCAAGCTCCAGCTCTGCGA

TUR1|AB863166.1| TGAGAAGATCGAAGAGCAAGAGAAGGCCAAAATCAGAATGACCAAACTCCAGCTCTGCGA

TUR2|AB863167.1| TGAGAAGATCGAAGAGCAAGAAAAGGCCAAAATCAGGATGACCAAGCTTCAGCTCTGCGA

TUR4|AB863168.1| TGAGAAGATCGAAGAGCAAGAAAAGGCCAAAATCAGGATGACCAAGCTTCAGCTCTGCGA

TUR5|AB863169.1| CGAGAAAATCGAAGAGCAAGAGAAGGCCAAGATTAGAATGACCAAGCTCCAGCTCTGCGA

TUR12|AB863170.1| TGAGAAGATTGAAGAGCAAGAGAAGGCCAAGATCAGAATGACCAAGCTTCAGCTCTGCGA

TUR34|AB863171.1| TGAGAAGATTGAAGAGCAAGAGAAGGCCAAGATCAGAATGACCAAACTCCAGCTCTGCGA

TUR50|AB863172.1| TGAAAAAATCGAAGAGCAAGAGAAGGCCAAAATCAGAATGACCAAGCTTCAGCTCTGCGA

TUR59|AB863173.1| TGAGAAGATTGAAGAGCAAGAGAAGGCCAAGATCAGAATGACCAAACTCCAGCTCTGCGA

TUR69|AB863174.1| TGAGAAAATCGAAGAGCAAGAGAAGGCCAAGATCAGAATGACCAAACTCCAGCTCTGCGA

TUR81|AB863175.1| TGAAAAAATTGAAGAGCAAGAAAAGGCCAAGATCAGAATGACCAAACTCCAGCTCTGCGA

TUR84|AB863176.1| TGAAAAAATTGAAGAGCAAGAGAAGGCCAAGATCAGAATGACCAAACTCCAGCTCTGCGA

TUR94|AB863177.1| TGAGAAGATCGAAGAGCAAGAGAAGGCCAAGATCAGAATGACCAAGCTTCAGCTCTGCGA

TUR213|AB863178.1| TGAGAAGATCGAAGAGCAAGAGAAGGCCAAGATCAGAATGACCAAACTCCAGCTCTGCGA

TUR214|AB863179.1| TGAGAAGATCGAAGAGCAAGAAAAGGCCAAAATCAGAATGACCAAGCTTCAGCTCTGCGA

TUR216|AB863180.1| TGAGAAGATCGAAGAACAAGAGAAAGCCAAAATCAGAATGACCAAGCTTCAGCTCTGCGA

TUR220|AB863181.1| TGAAAAAATTGAAGAGCAAGAGAAGGCCAAGATCAGAATGACCAAACTCCAGCTCTGCGA

TUR239|AB863182.1| TGAGAAGATCGAAGAGCAAGAAAAGGCCAAAATCAGAATGACCAAACTACAGCTCTGCGA

TUR244|AB863183.1| TGAGAAGATCGAAGAGCAAGAAAAGGCCAAAATCAGAATGACCAAGCTCCAGCTCTGCGA

TUR249|AB863184.1| GGAAAAGATCGAAGAGCAAGAGAAGGCCAAAATCAGAATGACCAAACTTCAGCTCTGCGA

TUR263|AB863185.1| TGAGAAGATCGAAGAGCAAGAGAAGGCCAAGATCAGAATGACCAAGCTTCAGCTCTGCGA

TUR278|AB863186.1| TGAAAAAATTGAAGAGCAAGAGAAGGCCAAGATCAGAATGACCAAACTCCAGCTCTGCGA

TUR279|AB863187.1| TGAGAAGATTGAAGAGCAAGAGAAGGCCAAGATCAGAATGACCAAACTCCAGCTCTGCGA

TUR285|AB863188.1| TGAGAAGATTGAAGAGCAAGAGAAGGCCAAGATCAGAATGACCAAGCTCCAGCTCTGCGA

TUR289|AB863189.1| TGAGAAGATTGAAGAGCAAGAGAAGGCCAAGATCAGAATGACCAAACTCCAGCTCTGCGA

TUR303|AB863190.1| TGAAAAGATCGAAGAGCAAGAAAAGGCCAAAATCAGAATGACCAAGCTCCAGCTCTGCGA

TUR306|AB863191.1| TGAGAAAATCGAAGAGCAAGAGAAGGCCAAGATTAGAATGACCAAGCTCCAGCTCTGTGA

Cabb-S|NC_001497.1| CATCTGCTACCTTGAGGAATTTACATGTGATTATGAAAAGAACATGTATAAGACAGAACT

CRO180A|AB863192.1| CATCTGCTACCTTGAAGAATTTACATGTGATTATGAGAAGAACATGTACAAGACGGAACT

GRC83|AB863193.1| CATCTGCTACCTTGAAGAATTTACATGTGATTATGAGAAGAACATGTACAAGACGGAACT

GRC84B|AB863194.1| CATCTGCTACCTTGAAGAATTTACATGTGATTATGAGAAGAACATGTACAAGACGGAACT

GRC86B|AB863195.1| CATCTGCTACCTTGAAGAATTTACATGTGATTATGAGAAGAACATGTATAAGACGGAACT

GRC86D|AB863196.1| CATCTGCTACCTTGAAGAATTTACATGTGATTATGAGAAGAACATGTACAAGACGGAACT

GRC87E|AB863197.1| CATCTGCTACCTTGAAGAATTTACATGTGATTATGAGAAGAACATGTACAAGACGGAACT

GRC87G|AB863198.1| CATCTGCTACCTTGAAGAATTTACATGTGATTATGAGAAGAACATGTACAAGACGGAACT

GRC91B|AB863199.1| CATCTGCTACCTTGAAGAATTTACATGTGATTATGAAAAGAACATGTACAAGACGGAACT

GRC92A|AB863200.1| CATCTGCTACCTTGAAGAATTTACATGTGATTATGAGAAAAACATGTACAAGACGGAACT

GRC92C|AB863201.1| CATCTGCTACCTTGAAGAATTTACATGTGATTATGAGAAGAACATGTACAAGACAGAACT

GRC92D|AB863202.1| CATCTGCTACCTTGAAGAATTTACATGTGATTATGAGAAGAACATGTACAAGACGGAACT

IRN1|AB863136.1| CATCTGCTACCTTGAAGAATTTACATGTGATTATGAGAAAAACATGTATAAGACGGAACT

IRN2|AB863137.1| TATCTGCTACCTTGAAGAATTTACATGTGATTATGAGAAGAACATGTACAAGACGGAACT

IRN3|AB863138.1| CATCTGCTACCTTGAAGAATTTACATGTGATTATGAGAAAAACATGTATAAGACGGAACT

IRN4|AB863139.1| TATCTGCTACCTTGAAGAATTTACATGTGACTATGAAAAGAACATGTACAAGACGGAACT

IRN5|AB863140.1| TATCTGCTACCTTGAAGAATTTACATGTGATTATGAGAAGAACATGTACAAGACGGAACT

IRN6|AB863141.1| TATCTGCTACCTTGAAGAATTTACATGTGATTACGAGAAGAACATGTACAAGACGGAACT

IRN7|AB863142.1| TATCTGCTACCTTGAAGAATTTACATGTGATTATGAGAAGAACATGTACAAGACGGAACT

IRN8|AB863143.1| TATCTGCTACCTTGAAGAATTTACATGTGATTATGAGAAGAACATGTACAAGACGGAACT

IRN9|AB863144.1| TATCTGCTACCTTGAAGAATTTACATGTGATTATGAGAAGAACATGTACAAGACGGAACT

IRN10|AB863145.1| TATCTGCTACCTTGAAGAATTTACATGCGATTATGAAAAGAACATGTACAAGACGGAACT

IRN11|AB863146.1| TATCTGCTACCTTGAAGAATTTACATGTGATTATGAGAAGAACATGTACAAGACGGAACT

IRN12|AB863147.1| TATCTGCTACCTTGAAGAATTTACATGTGATTACGAGAAGAACATGTACAAGACGGAACT

IRN13|AB863148.1| TATCTGCTACCTTGAAGAATTTACATGTGATTATGAGAAGAACATGTACAAGACGGAACT

IRN14|AB863149.1| TATCTGCTACCTTGAAGAATTTACATGTGATTATGAGAAGAACATGTACAAGACGGAACT

IRN15|AB863150.1| CATTTGCTACCTTGAAGAATTTACATGTGATTATGAGAAGAACATGTACAAGACGGAACT

IRN16|AB863151.1| CATCTGCTACCTTGAAGAATTTACATGTGATTATGAGAAAAACATGTACAAGACGGAACT

IRN17|AB863152.1| CATCTGCTACCTTGAAGAATTTACATGTGATTATGAGAAGAACATGTACAAGACGGAACT

IRN18|AB863153.1| CATCTGCTACCTTGAAGAATTTACATGTGATTATGAGAAGAACATGTACAAGACGGAACT

IRN19|AB863154.1| TATTTGCTACCTTGAAGAATTTACATGTGATTATGAGAAGAACATGTACAAGACGGAACT

IRN20|AB863155.1| CATCTGCTACCTTGAAGAATTTACATGTGATTATGAGAAAAACATGTACAAGACGGAACT

IRN21|AB863156.1| TATCTGCTACCTTGAAGAATTTACATGTGATTATGAGAAGAACATGTACAAGACGGAACT

JPNHGB340|AB863157.1| CATCTGCTACCTTGAAGAATTTACATGTGACTATGAAAAGAACATGTACAAGACGGAACT

JPNKWB778|AB863158.1| CATCTGCTACCTTGAAGAATTTACATGTGATTATGAAAAGAACATGTACAAGACGGAACT

JPNM|AB863159.1| CATCTGCTACCTTGAAGAATTTACATGTGATTATGAAAAGAACATGTACAAGACGGAACT

JPNN|AB863160.1| CATCTGCTACCTTGAAGAATTTACATGTGACTACGAAAAGAACATGTACAAGACGGAACT

JPNS1|AB863161.1| CATCTGCTACCTTGAAGAATTTACATGTGATTATGAAAAGAACATGTACAAGACGGAACT

JPNS2|AB863162.1| CATCTGCTACCTTGAAGAATTTACATGTGATTATGAAAAGAACATGTACAAGACGGAACT

JPNTKD762|AB863163.1| CATCTGCTACCTTGAAGAATTTACATGTGATTATGAAAAGAACATGTACAAGACGGAACT

JPNUV1|AB863164.1| CATCTGCTACCTTGAAGAATTTACATGTGATTATGAAAAGAACATGTACAAGACGGAACT

JPNUV26|AB863165.1| CATCTGCTACCTTGAAGAATTTACATGTGATTATGAAAAGAACATGTACAAGACGGAACT

TUR1|AB863166.1| TATCTGCTACCTTGAAGAATTTACATGTGACTATGAAAAGAACATGTACAAGACGGAACT

TUR2|AB863167.1| CATCTGCTACCTTGAAGAATTTACATGTGACTATGAAAAGAACATGTACAAGACGGAACT

TUR4|AB863168.1| CATCTGCTACCTTGAAGAATTTACATGTGACTATGAAAAGAACATGTACAAGACGGAACT

TUR5|AB863169.1| TATCTGCTACCTTGAAGAATTTACATGTGATTATGAGAAGAACATGTACAAGACGGAACT

TUR12|AB863170.1| CATCTGCTACCTTGAAGAATTTACATGTGATTACGAAAAAAACATGTACAAGACGGAACT

TUR34|AB863171.1| TATCTGCTACCTTGAAGAATTTACATGTGATTATGAAAAGAACATGTACAAGACGGAACT

TUR50|AB863172.1| CATCTGCTACCTTGAAGAATTTACATGTGATTATGAAAAGAACATGTACAAGACGGAACT

TUR59|AB863173.1| TATCTGCTACCTTGAAGAATTTACATGTGATTATGAAAAGAACATGTACAAGACGGAACT

TUR69|AB863174.1| TATCTGCTACCTTGAAGAATTTACATGTGATTATGAAAAGAACATGTACAAGACGGAACT

TUR81|AB863175.1| TATCTGCTACCTTGAAGAATTTACATGTGATTATGAGAAGAACATGTATAAGACGGAACT

TUR84|AB863176.1| TATCTGCTACCTTGAAGAATTTACATGTGATTACGAAAAGAACATGTACAAGACGGAACT

TUR94|AB863177.1| CATCTGCTACCTTGAAGAATTTACATGTGATTATGAGAAAAACATGTACAAGACGGAACT

TUR213|AB863178.1| TATCTGCTACCTTGAAGAATTTACATGTGATTATGAGAAGAACATGTACAAGACGGAACT

TUR214|AB863179.1| CATCTGCTACCTTGAAGAATTTACATGTGATTATGAGAAGAACATGTACAAGACGGAACT

TUR216|AB863180.1| CATCTGCTACCTTGAAGAATTCACATGTGATTATGAAAAGAACATGTACAAGACGGAACT

TUR220|AB863181.1| TATCTGCTACCTTGAAGAATTTACATGTGATTACGAAAAGAACATGTACAAGACGGAACT

TUR239|AB863182.1| CATCTGCTACCTTGAAGAATTTACATGTGACTATGAAAAGAACATGTACAAGACGGAACT

TUR244|AB863183.1| CATCTGCTACCTTGAAGAATTTACATGTGATTATGAGAAGAACATGTACAAAACGGAACT

TUR249|AB863184.1| CATCTGCTACCTTGAAGAATTTACATGTGATTATGAGAAGAACATGTACAAGACGGAACT

TUR263|AB863185.1| CATATGCTACCTTGAAGAATTTACATGTGATTATGAAAAGAACATGTACAAGACGGAACT

TUR278|AB863186.1| TATCTGCTACCTTGAAGAATTTACATGTGATTACGAAAAGAACATGTACAAGACGGAACT

TUR279|AB863187.1| TATCTGCTACCTTGAAGAATTTACATGTGATTATGAGAAGAACATGTACAAGACGGAACT

TUR285|AB863188.1| TATCTGCTACCTTGAAGAATTTACATGTGATTATGAAAAGAACATGTACAAGACGGAACT

TUR289|AB863189.1| TATCTGCTACCTTGAAGAATTTACATGTGATTATGAGAAGAACATGTACAAGACGGAACT

TUR303|AB863190.1| CATCTGTTACCTTGAAGAATTTACATGTGATTATGAGAAGAACATGTACAAGACGGAACT

TUR306|AB863191.1| CATCTGCTACCTTGAAGAATTTACATGTGATTATGAAAAGAACATGTACAAGACGGAACT

Cabb-S|NC_001497.1| GGCGGATTTCCCAGGATATATCAACCAGTACCTGTCAAAAATCCCCATCATTGGAGAAAA

CRO180A|AB863192.1| GGCGGATTTCCCAGGATATATCAACCAGTACCTGTCAAAAATCCCCATCATTGGAGAAAA

GRC83|AB863193.1| GGCGGATTTCCCAGGATATATCAACCAGTACCTGTCAAAAATCCCCATCATTGGAGAAAA

GRC84B|AB863194.1| GGCGGATTTCCCAGGATATATCAACCAGTACCTGTCAAAAATCCCCATCATTGGAGAAAA

GRC86B|AB863195.1| GGCGGATTTCCCAGGATATATCAACCAGTACCTGTCAAAAATTCCCATCATTGGAGAAAA

GRC86D|AB863196.1| GGCGGATTTCCCAGGATATATCAACCAGTACCTGTCAAAAATCCCCATCATTGGAGAAAA

GRC87E|AB863197.1| GGCGGATTTCCCTGGATATATCAACCAGTACCTGTCAAAAATCCCCATCATTGGAGAAAA

GRC87G|AB863198.1| GGCGGATTTCCCTGGATATATCAACCAGTACCTGTCAAAAATCCCCATCATTGGAGAAAA

GRC91B|AB863199.1| GGCGGATTTCCCAGGATATATCAACCAGTACTTGTCAAAAATCCCCATCATTGGAGAAAA

GRC92A|AB863200.1| GGCGGATTTCCCAGGATATATCAACCAGTACCTGTCAAAAATCCCCATCATTGGAGAAAA

GRC92C|AB863201.1| GGCGGATTTCCCAGGATATATCAACCAGTACCTGTCAAAAATCCCCATCATTGGAGAGAA

GRC92D|AB863202.1| GGCGGATTTCCCAGGATATATCAACCAGTACCTGTCAAAAATCCCCATCATTGGAGAAAA

IRN1|AB863136.1| GGCGGATTTCCCAGGATATATCAACCAGTACCTGTCAAAAATCCCCATCATTGGAGAAAA

IRN2|AB863137.1| GGCGGATTTCCCAGGATATATCAACCAGTACCTGTCAAAAATCCCCATCATTGGAGAAAA

IRN3|AB863138.1| GGCGGATTTCCCAGGATATATCAACCAGTACCTGTCAAAAATCCCCATCATTGGAGAAAA

IRN4|AB863139.1| GGCGGATTTCCCAGGATATATCAACCAGTACCTGTCAAAAATTCCCATCATTGGAGAAAA

IRN5|AB863140.1| GGCGGATTTCCCAGGATATATCAACCAGTACCTGTCAAAAATCCCCATCATTGGAGAAAA

IRN6|AB863141.1| GGCGGATTTCCCAGGATATATCAACCAGTACCTGTCAAAAATCCCCATCATAGGAGAAAA

IRN7|AB863142.1| GGCGGATTTCCCAGGATATATCAACCAGTACCTGTCAAAAATCCCCATCATTGGAGAAAA

IRN8|AB863143.1| GGCGGATTTCCCAGGATATATCAACCAGTACCTGTCAAAAATCCCCATCATTGGAGAAAA

IRN9|AB863144.1| GGCGGATTTCCCAGGATATATCAACCAGTACCTGTCAAAAATCCCCATCATTGGAGAAAA

IRN10|AB863145.1| GGCGGACTTCCCAGGATATATCAACCAGTACCTGTCAAAAATCCCCATCATTGGAGAAAA

IRN11|AB863146.1| GGCGGATTTCCCAGGATATATCAACCAGTACCTGTCAAAAATCCCCATCATTGGAGAAAA

IRN12|AB863147.1| GGCGGATTTCCCAGGATATATCAACCAGTACCTGTCAAAAATCCCCATCATTGGAGAAAA

IRN13|AB863148.1| GGCGGATTTCCCAGGATATATCAACCAGTACCTGTCAAAAATCCCCATCATAGGAGAAAA

IRN14|AB863149.1| GGCGGATTTCCCAGGATATATCAACCAGTACCTGTCAAAAATCCCCATAATAGGAGAAAA

IRN15|AB863150.1| GGCGGATTTCCCAGGATATATCAACCAGTACCTGTCAAAAATCCCCATCATTGGAGAAAA

IRN16|AB863151.1| GGCGGATTTCCCAGGATATATCAACCAGTACCTGTCAAAAATCCCCATCATTGGAGAAAA

IRN17|AB863152.1| GGCGGATTTCCCAGGATATATCAACCAGTACCTGTCAAAAATCCCCATCATTGGAGAAAA

IRN18|AB863153.1| GGCGGATTTCCCAGGATATATCAACCAGTACCTATCAAAAATCCCCATCATTGGAGAAAA

IRN19|AB863154.1| GGCGGATTTCCCAGGATATATCAACCAGTACCTGTCAAAAATCCCCATCATTGGAGAAAA

IRN20|AB863155.1| GGCGGATTTCCCAGGATATATCAACCAGTACCTGTCAAAAATCCCCATCATTGGAGAAAA

IRN21|AB863156.1| GGCAGATTTCCCAGGATATATCAACCAGTACCTGTCAAAAATCCCCATCATTGGAGAAAA

JPNHGB340|AB863157.1| GGCAGATTTCCCAGGATATATCAACCAGTACCTGTCAAAAATCCCCATCATTGGAGAAAA

JPNKWB778|AB863158.1| GGCAGATTTCCCAGGATATATCAACCAGTACCTGTCAAAAATCCCCATCATTGGAGAAAA

JPNM|AB863159.1| GGCAGATTTCCCAGGATATATCAACCAGTACCTGTCAAAAATCCCCATCATTGGAGAAAA

JPNN|AB863160.1| GGCAGATTTCCCAGGATACATCAACCAGTATCTGTCAAAAATCCCCATCATTGGAGAAAA

JPNS1|AB863161.1| GGCAGATTTCCCAGGATACATCAACCAGTACCTGTCAAAAATCCCCATCATAGGAGAAAA

JPNS2|AB863162.1| GGCAGATTTCCCAGGATACATCAACCAGTACCTGTCAAAAATCCCCATCATAGGAGAAAA

JPNTKD762|AB863163.1| GGCAGATTTCCCAGGATATATCAACCAGTACCTGTCAAAAATCCCCATCATTGGAGAAAA

JPNUV1|AB863164.1| GGCAGATTTCCCAGGATATATCAACCAGTACCTGTCAAAAATCCCCATCATTGGAGAAAA

JPNUV26|AB863165.1| GGCAGATTTCCCAGGATATATCAACCAGTACCTGTCAAAAATCCCCATCATTGGAGAAAA

TUR1|AB863166.1| GGCGGATTTCCCAGGATATATCAACCAGTACCTGTCAAAAATCCCCATCATTGGAGAAAA

TUR2|AB863167.1| GGCGGATTTCCCAGGATATATCAACCAGTACCTGTCAAAAATCCCCATCATTGGAGAAAA

TUR4|AB863168.1| GGCGGATTTCCCAGGATATATCAACCAGTACCTGTCAAAAATCCCCATCATTGGAGAAAA

TUR5|AB863169.1| GGCGGATTTCCCAGGGTATATCAACCAGTACTTGTCAAAAATCCCCATCATTGGAGAAAA

TUR12|AB863170.1| GGCGGATTTCCCAGGATATATCAACCAGTACCTGTCAAAAATCCCCATCATTGGAGAAAA

TUR34|AB863171.1| GGCGGATTTCCCAGGATATATCAACCAGTACCTGTCAAAAATCCCCATCATTGGAGAAAA

TUR50|AB863172.1| GGCGGATTTCCCAGGATATATCAACCAGTACCTGTCAAAAATCCCCATCATTGGAGAAAA

TUR59|AB863173.1| GGCGGATTTCCCAGGATATATCAACCAGTACCTGTCAAAAATCCCCATCATTGGAGAAAA

TUR69|AB863174.1| GGCAGATTTCCCAGGATATATCAACCAGTACCTGTCAAAAATCCCCATCATTGGAGAAAA

TUR81|AB863175.1| GGCGGATTTCCCAGGATATATCAACCAGTACCTGTCAAAAATCCCCATCATTGGAGAAAA

TUR84|AB863176.1| GGCGGATTTCCCAGGATATATCAACCAGTACCTGTCAAAAATCCCCATCATTGGAGAAAA

TUR94|AB863177.1| GGCGGATTTCCCAGGATATATCAACCAGTACCTGTCAAAAATCCCCATCATTGGAGAAAA

TUR213|AB863178.1| GGCGGATTTCCCAGGATATATCAACCAGTACCTGTCAAAAATCCCCATCATTGGAGAAAA

TUR214|AB863179.1| GGCGGATTTCCCAGGATATATCAACCAGTACCTGTCAAAAATCCCCATCATTGGAGAAAA

TUR216|AB863180.1| GGCGGATTTCCCAGGATATATCAACCAGTACCTGTCAAAAATCCCCATCATTGGAGAAAA

TUR220|AB863181.1| GGCGGATTTCCCAGGATATATCAACCAGTACCTGTCAAAAATCCCCATCATTGGAGAAAA

TUR239|AB863182.1| GACGGATTTCCCAGGATATATCAACCAGTACCTGTCAAAAATCCCCATCATTGGAGAAAA

TUR244|AB863183.1| GGCGGATTTCCCAGGATATATCAACCAGTACCTGTCAAAAATCCCCATCATTGGAGAAAA

TUR249|AB863184.1| GGCGGATTTCCCAGGATATATCAACCAGTACCTGTCAAAAATCCCCATCATTGGAGAAAA

TUR263|AB863185.1| GGCGGATTTCCCAGGATATATCAACCAGTACCTGTCAAAGATCCCCATCATTGGAGAAAA

TUR278|AB863186.1| GGCGGATTTCCCAGGATATATCAACCAGTACCTGTCAAAAATCCCCATCATTGGAGAAAA

TUR279|AB863187.1| GGCGGATTTCCCAGGATATATCAACCAGTACCTATCAAAAATCCCCATCATTGGAGAAAA

TUR285|AB863188.1| GGCGGATTTCCCAGGATATATCAACCAGTACCTGTCAAAAATCCCCATCATTGGAGAAAA

TUR289|AB863189.1| GGCGGATTTCCCAGGATATATCAACCAGTACCTATCAAAAATCCCCATCATTGGAGAAAA

TUR303|AB863190.1| GGCGGATTTCCCAGGATATATCAACCAATACCTGTCAAAAATCCCCATCATTGGAGAAAA

TUR306|AB863191.1| GGCGGATTTCCCAGGATATATCAACCAGTACCTGTCAAAAATCCCCATCATTGGAGAAAA

Cabb-S|NC_001497.1| AGCGTTAACACGCTTTAGGCATGAAGCTAACGGAACCAGCATCTACAGTTTAGGTTTCGC

CRO180A|AB863192.1| AGCGTTAACACGCTTTAGGCATGAAGCCAACGGAACCAGCATCTACAGTTTAGGTTTCGC

GRC83|AB863193.1| AGCGCTAACACGCTTTAGGCATGAAGCCAACGGAACCAGCATCTACAGCTTAGGTTTCGC

GRC84B|AB863194.1| AGCGCTAACACGCTTTAGGCATGAAGCCAACGGAACCAGCATCTACAGCTTAGGTTTCGC

GRC86B|AB863195.1| AGCGCTAACACGCTTTAGGCATGAAGCCAACGGAACCAGCATCTACAGCTTAGGTTTCGC

GRC86D|AB863196.1| AGCGCTAACACGCTTTAGGCATGAAGCCAACGGAACCAGCATCTACAGCTTAGGTTTCGC

GRC87E|AB863197.1| AGCGCTAACACGCTTTAGGCATGAAGCCAACGGAACCAGCATCTACAGCTTAGGTTTCGC

GRC87G|AB863198.1| AGCGCTAACACGCTTTAGGCATGAAGCCAACGGAACCAGCATCTACAGCTTAGGTTTCGC

GRC91B|AB863199.1| AGCGCTAACACGCTTTAGGCACGAAGCCAACGGAACCAGCATCTACAGCTTAGGTTTCGC

GRC92A|AB863200.1| AGCGCTAACACGCTTTAGGCATGAAGCCAACGGAACCAGCATCTACAGTTTAGGTTTCGC

GRC92C|AB863201.1| AGCGCTAACACGCTTTAGGCATGAAGCCAACGGAACCAGCATCTACAGCTTAGGTTTCGC

GRC92D|AB863202.1| GGCGTTAACACGCTTTAGGCATGAAGCCAACGGAACCAGCATCTACAGCTTAGGTTTCGC

IRN1|AB863136.1| AGCGCTAACACGCTTTAGACATGAAGCCAACGGAACCAGCATCTACAGCTTAGGTTTTGC

IRN2|AB863137.1| AGCGCTAACACGCTTTAGACATGAAGCCAACGGAACCAGCATCTACAGCTTAGGTTTTGC

IRN3|AB863138.1| AGCGCTAACACGCTTTAGACATGAAGCCAACGGAACCAGCATCTACAGCTTAGGTTTTGC

IRN4|AB863139.1| AGCGCTAACACGCTTTAGACATGAAGCCAATGGAACCAGCATCTACAGCTTAGGTTTTGC

IRN5|AB863140.1| AGCGCTAACACGCTTTAGACATGAAGCCAACGGAACCAGCATCTACAGCTTAGGTTTTGC

IRN6|AB863141.1| AGCATTAACACGCTTTAGACATGAAGCCAACGGAACCAGCATCTACAGCTTAGGTTTTGC

IRN7|AB863142.1| AGCGCTAACACGCTTTAGACATGAAGCCAACGGAACCAGCATCTACAGCTTAGGTTTTGC

IRN8|AB863143.1| AGCGCTAACACGCTTTAGACATGAAGCCAACGGAACCAGCATCTACAGCTTAGGTTTTGC

IRN9|AB863144.1| AGCGCTAACACGCTTTAGGCATGAAGCCAACGGAACCAGCACATACAGCTTAGGTTTCGC

IRN10|AB863145.1| AGCGCTAACACGCTTTAGGCACGAAGCCAACGGAACCAGCATATACAGCTTAGGTTTCGC

IRN11|AB863146.1| AGCGCTAACACGCTTTAGACATGAAGCCAACGGAACCAGCATCTACAGCTTAGGTTTTGC

IRN12|AB863147.1| AGCGCTAACACGCTTTAGGCATGAAGCCAACGGAACCAGCATTTACAGTTTAGGTTTCGC

IRN13|AB863148.1| AGCATTAACACGCTTTAGACATGAAGCCAACGGAACCAGCATCTACAGCTTAGGTTTTGC

IRN14|AB863149.1| AGCATTAACACGCTTTAGACATGAAGCCAACGGAACCAGCATCTACAGCTTAGGTTTTGC

IRN15|AB863150.1| AGCGTTAACACGCTTTAGACATGAAGCCAACGGAACCAGCATCTACAGCTTAGGTTTCGC

IRN16|AB863151.1| AGCGCTAACACGCTTTAGGCATGAAGCCAACGGAACCAGCATCTACAGCTTAGGTTTCGC

IRN17|AB863152.1| AGCGCTAACACGCTTTAGGCATGAAGCCAACGGAACCAGCATCTACAGCTTAGGTTTCGC

IRN18|AB863153.1| AGCGCTAACACGCTTTAGGCATGAAGCCAACGGAACCAGCATCTACAGCTTAGGTTTTGC

IRN19|AB863154.1| AGCGCTAACACGCTTTAGACATGAAGCCAACGGAACCAGCATCTACAGCTTAGGTTTTGC

IRN20|AB863155.1| AGCGCTAACACGCTTTAGGCATGAAGCCAATGGAACCAGCATATACAGCTTAGGTTTCGC

IRN21|AB863156.1| AGCATTAACACGCTTTAGACATGAAGCCAACGGAACCAGCATCTACAGCTTAGGTTTTGC

JPNHGB340|AB863157.1| AGCATTAACACGCTTTAGACATGAAGCTAACGGAACCAGCATCTACAGCTTAGGTTTCGC

JPNKWB778|AB863158.1| AGCATTAACACGCTTTAGACATGAAGCTAACGGAACCAGCATCTACAGCTTAGGTTTCGC

JPNM|AB863159.1| AGCATTAACACGCTTTAGACATGAAGCTAACGGAACCAGCATCTACAGCTTAGGTTTCGC

JPNN|AB863160.1| AGCGCTAACACGCTTTAGGCATGAAGCCAACGGAACCAGCATCTACAGCTTAGGTTTCGC

JPNS1|AB863161.1| AGCGCTAACACGCTTTAGGCATGAAGCCAACGGAACCAGCATCTACAGCTTAGGTTTCGC

JPNS2|AB863162.1| AGCGCTAACACGCTTTAGGCATGAAGCCAACGGAACCAGCATCTACAGCTTAGGTTTCGC

JPNTKD762|AB863163.1| AGCATTAACACGCTTTAGACATGAAGCTAACGGAACCAGCATCTACAGCTTAGGTTTCGC

JPNUV1|AB863164.1| AGCATTAACACGCTTTAGACATGAAGCTAACGGAACCAGCATCTACAGCTTAGGTTTCGC

JPNUV26|AB863165.1| AGCATTAACACGCTTTAGACATGAAGCTAACGGAACCAGCATCTACAGCTTAGGTTTCGC

TUR1|AB863166.1| AGCGTTAACACGCTTTAGACATGAAGCCAACGGAACCAGCATCTACAGCTTAGGTTTCGC

TUR2|AB863167.1| AGCGTTAACACGTTTTAGGCATGAAGCCAACGGAACTAGCATCTACAGCTTAGGTTTCGC

TUR4|AB863168.1| AGCGTTAACACGTTTTAGGCATGAAGCCAACGGAACTAGCATCTACAGCTTAGGTTTCGC

TUR5|AB863169.1| AGCGCTAACACGCTTTAGGCATGAAGCCAACGGAACCAGCATCTACAGCTTAGGTTTCGC

TUR12|AB863170.1| AGCGTTAACACGCTTTAGGCATGAAGCCAACGGAACCAGCATCTACAGCTTAGGTTTCGC

TUR34|AB863171.1| AGCGCTAACACGCTTTAGACATGAAGCCAACGGAACCAGCATCTACAGCTTAGGTTTTGC

TUR50|AB863172.1| AGCGTTAACACGCTTTAGACATGAAGCCAACGGAACCAGCATCTACAGCTTAGGTTTCGC

TUR59|AB863173.1| AGCGCTAACACGCTTTAGGCATGAAGCCAACGGAACCAGCATCTACAGCTTAGGTTTCGC

TUR69|AB863174.1| AGCGCTAACACGCTTTAGGCATGAAGCCAACGGAACCAGCATCTACAGCTTAGGTTTCGC

TUR81|AB863175.1| AGCGTTAACACGCTTTAGACATGAAGCCAACGGAACCAGCATCTACAGCTTAGGTTTTGC

TUR84|AB863176.1| AGCGCTAACACGCTTTAGGCATGAGGCCAACGGAACCAGCATCTACAGCTTAGGTTTCGC

TUR94|AB863177.1| AGCGCTAACACGCTTTAGGCATGAAGCCAACGGAACCAGCATTTACAGCTTAGGTTTCGC

TUR213|AB863178.1| AGCGTTAACACGCTTTAGACATGAAGCCAACGGAACCAGCATCTACAGCTTAGGTTTCGC

TUR214|AB863179.1| AGCGTTAACACGCTTTAGGCATGAAGCCAACGGAACCAGCATCTACAGCTTAGGTTTCGC

TUR216|AB863180.1| AGCGTTAACACGCTTTAGGCATGAAGCCAACGGAACCAGCATCTACAGCTTAGGTTTCGC

TUR220|AB863181.1| AGCGCTAACACGCTTTAGGCATGAGGCCAACGGAACCAGCATCTACAGCTTAGGTTTCGC

TUR239|AB863182.1| AGCGTTAACACGTTTTAGGCATGAAGCCAACGGAACTAGCATCTACAGCTTAGGTTTCGC

TUR244|AB863183.1| AGCGCTAACACGCTTTAGGCATGAAGCCAACGGAACCAGCATCTACAGCTTAGGTTTCGC

TUR249|AB863184.1| AGCGTTAACACGCTTTAGACATGAAGCCAACGGAACCAGCATCTACAGCTTAGGTTTCGC

TUR263|AB863185.1| AGCGCTAACACGCTTTAGGCATGAAGCCAACGGAACCAGCATTTACAGCTTAGGTTTCGC

TUR278|AB863186.1| AGCGCTAACACGCTTTAGGCATGAAGCCAACGGAAGCAGCATCTACAGCTTAGGTTTCGC

TUR279|AB863187.1| AGCGTTAACACGCTTTAGGCATGAAGCCAACGGAACCAGCATCTACAGCTTAGGTTTCGC

TUR285|AB863188.1| AGCGCTAACACGCTTTAGGCATGAAGCCAACGGAACCAGCATCTACAGCTTAGGTTTCGC

TUR289|AB863189.1| AGCGCTAACACGCTTTAGGCATGAAGCCAACGGAACCAGCATCTACAGCTTAGGTTTCGC

TUR303|AB863190.1| AGCACTAACACGCTTTAGGCATGAAGCCAACGAAACCAGCATCTACAGCTTAGGTTTCGC

TUR306|AB863191.1| AGCATTAACACGCTTTAGGCATGAAGCCAACGGAACCAGCATCTACAGCTTAGGTTTCGC

Cabb-S|NC_001497.1| GGCAAAGATAGTCAAAGAAGAACTATCTAAAATCTGCGACTTATCCAAGAAGCAGAAGAA

CRO180A|AB863192.1| GGCAAAGATAGTAAAAGAAGAACTATCTAAAATCTGCGACTTATCCAAGAAGCAGAAGAA

GRC83|AB863193.1| GGCAAAGATAGTAAAAGAAGAGCTATCTAAAATCTGCGACTTATCCAAGAAGCAGAAAAA

GRC84B|AB863194.1| GGCAAAGATAGTAAAAGAAGAGCTATCTAAAATCTGCGACTTATCCAAGAAGCAGAAGAA

GRC86B|AB863195.1| GGCAAAGATAGTAAAAGAAGAGCTATCTAAAATCTGCGACTTATCCAAGAAGCAGAAGAA

GRC86D|AB863196.1| GGCAAAGATAGTAAAAGAAGAGCTATCTAAAATCTGCGACTTATCCAAGAAGCAGAAGAA

GRC87E|AB863197.1| GGCAAAGATAGTAAAAGAAGAGCTATCTAAAATCTGCGACTTATCCAAGAAGCAGAAGAA

GRC87G|AB863198.1| GGCAAAGATAGTAAAAGAAGAGCTATCTAAAATCTGCGACTTATCCAAGAAGCAGAAGAA

GRC91B|AB863199.1| GGCGAAGATAGTAAAAGAAGAACTATCTAAAATCTGCGACTTATCTAAGAAGCAGAAGAA

GRC92A|AB863200.1| GGCAAAGATAGTAAAAGAAGAGCTATCTAAAATCTGCGACTTATCCAAGAAGCAGAAGAA

GRC92C|AB863201.1| GGCAAAGATAGTAAAAGAAGAGCTATCTAAAATCTGCGACTTATCCAAGAAGCAGAAGAA

GRC92D|AB863202.1| GGCGAAGATAGTAAAAGAAGAACTATCTAAAATCTGCGACTTATCTAAGAAGCAGAAGAA

IRN1|AB863136.1| GGCGAAGATAGTCAAAGAAGAACTATCTAAAATCTGCGACTTATCCAAGAAGCAGAAGAA

IRN2|AB863137.1| GGCGAAGATAGTCAAAGAAGAACTATCTAAAATCTGCGACTTATCCAAGAAGCAGAAGAA

IRN3|AB863138.1| GGCGAAGATAGTCAAAGAAGAACTATCTAAAATCTGCGACTTATCCAAGAAGCAGAAGAA

IRN4|AB863139.1| GGCGAAGATAGTCAAAGAAGAACTATCTAAAATCTGCGACTTATCCAAGAAGCAGAAGAA

IRN5|AB863140.1| GGCAAAGATAGTCAAAGAAGAACTATCTAAAATCTGCGACTTATCCAAGAAGCAGAAGAA

IRN6|AB863141.1| GGCGAAGATAGTCAAAGAAGAACTATCTAAAATCTGCGACTTATCCAAGAAGCAGAAGAA

IRN7|AB863142.1| GGCGAAGATAGTCAAAGAAGAACTATCTAAAATTTGCGACTTATCCAAGAAGCAGAAGAA

IRN8|AB863143.1| GGCAAAGATAGTCAAAGAAGAACTATCTAAAATCTGCGACTTATCCAAGAAGCAGAAGAA

IRN9|AB863144.1| AGCGAAGATAGTAAAAGAAGAACTATCTAAAATCTGCGACTTATCCAAGAAGCAGAAGAA

IRN10|AB863145.1| GGCAAAGATAGTCAAAGAAGAACTATCTAAAATCTGCGACTTATCCAAGAAGCAGAAGAA

IRN11|AB863146.1| GGCGAAGATAGTCAAAGAAGAACTATCTAAAATCTGTGACTTATCCAAGAAGCAGAAGAA

IRN12|AB863147.1| GGCGAAGATAGTCAAAGAAGAACTATCTAAAATCTGCGACTTATCCAAGAAGCAGAAGAA

IRN13|AB863148.1| GGCAAAGATAGTCAAAGAAGAACTATCTAAAATCTGCGACTTATCCAAGAAGCAGAAGAA

IRN14|AB863149.1| GGCAAAGATAGTCAAAGAAGAACTATCTAAAATCTGCGACTTATCCAAGAAGCAGAAGAA

IRN15|AB863150.1| GGCAAAGATAGTAAAAGAAGAACTATCTAAAATCTGCGACTTATCCAAGAAGCAGAAAAA

IRN16|AB863151.1| GGCGAAGATAGTAAAAGAAGAACTATCTAAAATCTGCGACTTATCAAAGAAGCAGAAGAA

IRN17|AB863152.1| AGCGAAGATAGTAAAAGAAGAACTATCTAAAATCTGCGACTTATCAAAGAAGCAGAAGAA

IRN18|AB863153.1| GGCGAAGATAGTCAAAGAAGAACTATCTAAAATCTGCGACTTATCCAAGAAGCAGAAGAA

IRN19|AB863154.1| GGCGAAGATAGTCAAAGAAGAACTATCTAAAATCTGCGACTTATCCAAGAAGCAGAAGAA

IRN20|AB863155.1| GGCGAAGATAGTAAAGGAAGAACTATCTAAAATCTGCGACTTATCAAAGAAGCAGAAGAA

IRN21|AB863156.1| GGCGAAGATAGTCAAAGAAGAACTATCTAAAATCTGCGACTTATCCAAGAAGCAGAAGAA

JPNHGB340|AB863157.1| GGCGAAGATAGTAAAAGAGGAACTATCTAAAATCTGCGACTTATCCAAGAAGCAGAAGAA

JPNKWB778|AB863158.1| GGCGAAGATAGTAAAAGAGGAACTATCTAAAATCTGCGACTTATCCAAGAAGCAGAAGAA

JPNM|AB863159.1| GGCGAAGATAGTAAAAGAGGAACTATCTAAAATCTGCGACTTATCCAAGAAGCAGAAGAA

JPNN|AB863160.1| GGCGAAGATAGTAAAAGAAGAACTATCTAAAATCTGCGACTTATCCAAGAAGCAGAAGAA

JPNS1|AB863161.1| GGCGAAGATAGTAAAAGAAGAACTATCTAAAATCTGCGACTTATCCAAGAAGCAGAAGAA

JPNS2|AB863162.1| GGCGAAGATAGTAAAAGAAGAACTATCTAAAATCTGCGACTTATCCAAGAAGCAGAAGAA

JPNTKD762|AB863163.1| GGCGAAGATAGTAAAAGAGGAACTATCTAAAATCTGCGACTTATCCAAAAAGCAGAAGAA

JPNUV1|AB863164.1| GGCGAAGATAGTAAAAGAGGAACTATCTAAAATCTGCGACTTATCCAAGAAGCAGAAGAA

JPNUV26|AB863165.1| GGCGAAGATAGTAAAAGAGGAACTATCTAAAATCTGCGACTTATCCAAGAAGCAGAAGAA

TUR1|AB863166.1| GGCAAAGATAGTAAAAGAAGAACTATCTAAAATCTGCGACTTATCCAAGAAGCAGAAGAA

TUR2|AB863167.1| GGCAAAGATAGTAAAAGAAGAACTATCTAAAATTTGCGACTTATCCAAGAAGCAGAAGAA

TUR4|AB863168.1| GGCAAAGATAGTAAAAGAAGAACTATCTAAAATTTGCGACTTATCCAAGAAGCAGAAGAA

TUR5|AB863169.1| GGCGAAGATAGTAAAAGAAGAACTATCTAAAATCTGCGACTTATCCAAGAAGCAGAAGAA

TUR12|AB863170.1| GGCAAAGATAGTAAAAGAAGAACTATCTAAAATCTGCGACTTAACTAAGAAGCAGAAGAA

TUR34|AB863171.1| GGCAAAGATAGTAAAAGAAGAACTATCTAAAATCTGCGACTTAACTAAGAAGCAGAAGAA

TUR50|AB863172.1| GGCGAAGATAGTAAAGGAAGAACTATCTAAAATCTGCGACTTAACTAAGAAGCAGAAGAA

TUR59|AB863173.1| GGCAAAGATAGTAAAAGAAGAACTATCTAAAATCTGCGACTTATCCAAGAAGCAGAAGAA

TUR69|AB863174.1| GGCAAAGATAGTAAAAGAAGAACTATCTAAAATCTGCGACTTATCCAAGAAGCAGAAAAA

TUR81|AB863175.1| GGCAAAGATAGTAAAGGAAGAACTATCCAAAATCTGCGACTTATCCAAGAAGCAGAAGAA

TUR84|AB863176.1| GGCAAAGATAGTAAAAGAAGAACTATCTAAAATCTGCGACTTATCCAAGAAGCAGAAGAA

TUR94|AB863177.1| GGCGAAGATAGTAAAAGAAGAACTATCTAAAATCTGCGACTTGTCCAAGAAGCAGAAGAA

TUR213|AB863178.1| GGCAAAGATAGTAAAAGAAGAACTATCTAAAATCTGCGACTTATCCAAGAAACAGAAGAA

TUR214|AB863179.1| GGCAAAGATAGTAAAAGAAGAACTATCTAAAATCTGCGACTTATCCAAGAAGCAGAAGAA

TUR216|AB863180.1| AACAAAGATAGTAAAAGAAGAACTATCTAAGATCTGCGACTTAACCAAGAAGCAGAAGAA

TUR220|AB863181.1| GGCAAAGATAGTAAAAGAAGAACTATCTAAAATCTGCGACTTATCCAAGAAGCAGAAGAA

TUR239|AB863182.1| GGCAAAGATAGTAAAAGAAGAACTATCTAAAATCTGCGACTTATCCAAGAAGCAGAAGAA

TUR244|AB863183.1| GGCAAAGATAGTAAAAGAAGAACTATCTAAAATCTGCGACTTATCCAAGAAGCAGAAGAA

TUR249|AB863184.1| GGCAAAGATAGTAAAAGAAGAACTATCTAAAATCTGCGACTTATCCAAGAAGCAGAAGAA

TUR263|AB863185.1| GGCAAAGATAGTAAAAGAAGAACTATCTAAAATCTGCGACTTATCCAAGAAGCAGAAGAA

TUR278|AB863186.1| GGCAAAGATAGTAAAAGAAGAACTATCTAAAATCTGCGACTTATCCAAGAAGCAGAAAAA

TUR279|AB863187.1| GGCGAAGATAGTAAAAGAAGAACTATCTAAAATCTGCGACTTATCCAAGAAGCAGAAGAA

TUR285|AB863188.1| GGCGAAGATAGTAAAAGAAGAACTATCTAAAATCTGCGACTTATCCAAGAAGCAGAAGAA

TUR289|AB863189.1| GGCAAAGATAGTAAAAGAAGAACTATCTAAAATCTGCGACTTATCCAAGAAGCAGAAGAA

TUR303|AB863190.1| GGCAAAGATAGTAAAAGAAGAACTATCTAAAATCTGCGACTTATCCAAGAAGCAGAAGAA

TUR306|AB863191.1| GGCGAAGATAGTAAAAGAAGAACTATCTAAAATCTGCGACTTATCCAAGAAGCAGAAGAA

Cabb-S|NC_001497.1| GTTGAAGAAATTCAACAAGAAGTGTTGTAGCATCGGAGAAGCTTCAACAGAATATGGATG

CRO180A|AB863192.1| GTTGAAGAAATTCAACAAGAAATGCTGTAGCATCGGAGAAGCTTCAGTAGAATATGGATG

GRC83|AB863193.1| GTTGAAGAAATTCAACAAGAAGTGCTGCAGCATCGGAGAAGCTTCAGTAGAATATGGATG

GRC84B|AB863194.1| GTTGAAGAAATTCAACAAGAAGTGCTGCAGCATCGGAGAAGCTTCAGTAGAATATGGATG

GRC86B|AB863195.1| GTTGAAGAAATTCAACAAGAAGTGCTGCAGCATCGGAGAAGCTTCAGTAGAATATGGATG

GRC86D|AB863196.1| GTTGAAGAAATTCAACAAGAAGTGCTGCAGCATCGGAGAAGCTTCAGTAGAATATGGATG

GRC87E|AB863197.1| GTTGAAGAAATTCAACAAGAAGTGCTGCAGCATCGGAGAAGCTTCAGTAGAATATGGATG

GRC87G|AB863198.1| GTTGAAGAAATTCAACAAGAAGTGCTGCAGCATCGGAGAAGCTTCAGTAGAATATGGATG

GRC91B|AB863199.1| GTTGAAGAAATTCAACAAGAAGTGCTGTAGCATCGGAGAAGCTTCAGTAGAATATGGATG

GRC92A|AB863200.1| GTTGAAGAAATTCAACAAAAAGTGCTGCAGCATCGGAGAAGCTTCAGTAGAATATGGATG

GRC92C|AB863201.1| GTTGAAGAAATTCAACAAGAAGTGCTGCAGCATCGGAGAAGCTTCAGTAGAATATGGATG

GRC92D|AB863202.1| GTTGAAGAAATTCAACAAGAAATGCTGTAGCATCGGAGAAGCTTCAGTAGAATATGGATG

IRN1|AB863136.1| GTTGAAGAAATTCAACAAGAAGTGCTGTAGCATCGGAGAAGCTTCAGTAGAATATGGATG

IRN2|AB863137.1| GTTGAAAAAATTCAACAAGAAGTGCTGCAGCATCGGAGAAGCTTCAGTAGAATACGGATG

IRN3|AB863138.1| GTTGAAGAAATTCAACAAGAAGTGCTGTAGCATCGGAGAAGCTTCAGTAGAATATGGATG

IRN4|AB863139.1| GTTGAAGAAATTCAACAAGAAGTGCTGTAGCATCGGAGAAGCTTCAGTAGAATATGGATG

IRN5|AB863140.1| GTTGAAGAAATTCAACAAAAAGTGCTGCAGCATCGGAGAAGCTTCAGTAGAATATGGATG

IRN6|AB863141.1| GTTGAAGAAATTCAACAAGAAGTGCTGCAGCATCGGAGAAGCTTCAGTAGAATATGGATG

IRN7|AB863142.1| GTTGAAGAAATTCAACAAAAAGTGCTGCAGCATCGGAGAAGCTTCAGTAGAATATGGATG

IRN8|AB863143.1| GTTGAAGAAATTCAACAAAAAGTGCTGCAGCATCGGAGAAGCTTCAGTAGAATATGGATG

IRN9|AB863144.1| GTTGAAGAAATTCAATAAGAAGTGCTGTAGCATCGGAGAAGCTTCAGTAGAATATGGATG

IRN10|AB863145.1| GTTGAAGAAATTCAACAAGAAGTGCTGTAACATCGGAGAAGCTTCAGTAGAATATGGATG

IRN11|AB863146.1| GTTGAAGAAATTCAACAAGAAGTGCTGCAGCATCGGAGAAGCTTCAGTAGAATATGGATG

IRN12|AB863147.1| GTTGAAGAAATTCAACAAGAAGTGCTGCAGCATCGGAGAAGCTTCAGTAGAATATGGATG

IRN13|AB863148.1| GTTGAAGAAATTCAACAAGAAGTGCTGTAGCATCGGAGAAGCTTCAATAGAATATGGATG

IRN14|AB863149.1| GTTGAAGAAATTCAACAAGAAGTGCTGCAGCATCGGAGAAGCTTCAGTAGAATATGGATG

IRN15|AB863150.1| GTTGAAGAAATTCAACAAGAAATGCTGTAGCATCGGAGAAGCTTCAGTAGAATATGGATG

IRN16|AB863151.1| GTTGAAGAAATTCAACAAGAAGTGCTGTAGCATCGGAGAAGCTTCAGTAGAATATGGATG

IRN17|AB863152.1| GTTGAAAAAATTCAACAAGAAGTGCTGTAGCATCGGAGAAGCTTCAGTAGAATATGGATG

IRN18|AB863153.1| GTTGAAGAAATTCAACAAGAAGTGCTGTAGCATCGGAGAAGCTTCAGTAGAATATGGATG

IRN19|AB863154.1| GTTGAAGAAATTCAACAAGAAGTGCTGTAGCATCGGAGAAGCTTCAGTAGAATATGGATG

IRN20|AB863155.1| GTTGAAGAAATTCAACAAGAAGTGCTGTAGCATCGGAGAAGCTTCAGTAGAATATGGATG

IRN21|AB863156.1| GTTGAAGAAATTCAACAAAAAGTGCTGCAGCATCGGAGAAGCTTCAGTAGAATATGGATG

JPNHGB340|AB863157.1| GTTGAAGAAATTCAACAAAAAATGCTGCAGCATCGGCGAAGCTTCAGTAGAATATGGATG

JPNKWB778|AB863158.1| GTTGAAGAAATTCAACAAGAAATGCTGCAGCATCGGCGAAGCTTCAGTAGAATATGGATG

JPNM|AB863159.1| GCTGAAGAAATTCAACAAGAAATGCTGCAGCATCGGCGAAGCTTCAGTAGAATATGGATG

JPNN|AB863160.1| GTTGAAGAAATTCAGCAAAAAGTGCTGTAGCATCGGAGAAGCTTCAGCAGAGTATGGGTG

JPNS1|AB863161.1| GTTGAAGAAATTCAGCAAAAAGTGCTGTAGCATCGGAGAAGCTTCAGCAGAATATGGATG

JPNS2|AB863162.1| GTTGAAGAAATTCAGCAAAAAGTGCTGTAGCATCGGAGAAGCTTCAGCAGAATATGGATG

JPNTKD762|AB863163.1| GTTGAAGAAATTCAACAAGAAGTGCTGCAGCATCGGCGAAGCTTCAGTAGAATATGGATG

JPNUV1|AB863164.1| GCTGAAGAAATTCAACAAGAAATGCTGCAGCATCGGCGAAGCTTCAGTAGAATATGGATG

JPNUV26|AB863165.1| GCTGAAGAAATTCAACAAGAAATGCTGCAGCATCGGCGAAGCTTCAGTAGAATATGGATG

TUR1|AB863166.1| GTTGAAGAAATTCAACAAGAAATGCTGTAGCATCGGAGAAGCTTCAGTAGAATATGGATG

TUR2|AB863167.1| GTTGAAGAAATTCAACAAGAAGTGCTGTAGCATCGGAGAAGCTTCAGTAGAATATGGATG

TUR4|AB863168.1| GTTGAAGAAATTCAACAAGAAGTGCTGTAGCATCGGAGAAGCTTCAGTAGAATATGGACG

TUR5|AB863169.1| GTTGAAGAAATTCAACAAGAAGTGCTGTAGCATCGGAGAAGCTTCAGTAGAATATGGGTG

TUR12|AB863170.1| GTTGAAGAAATTCAACAAGAAGTGCTGTAGCATCGGAGAAGCTTCAGTAGAATATGGATG

TUR34|AB863171.1| GTTGAAGAAATTCAACAAGAAGTGCTGTAGCATCGGAGAAGCTTCAGTAGAATATGGATG

TUR50|AB863172.1| GTTGAAGAAATTCAACAAGAAGTGCTGTAGCATCGGAGAAGCTTCAGTAGAATATGGATG

TUR59|AB863173.1| GTTGAAGAAATTCAACAAGAAGTGCTGTAGCATCGGAGAAGCTTCAGTAGAATACGGATG

TUR69|AB863174.1| GTTGAAGAAATTCAACAAGAAGTGCTGTAGCATCGGAGAAGCTTCAGTAGAATATGGATG

TUR81|AB863175.1| GTTGAAGAAATTCAACAAGAAGTGCTGTAGCATCGGCGAAGCTTCAGTAGAATATGGATG

TUR84|AB863176.1| GTTGAAGAAATTCAACAAGAAGTGCTGTAGCATCGGAGAAGCTTCAGTAGAATATGGATG

TUR94|AB863177.1| GTTGAAGAAATTCAACAAGAAGTGCTGTAGCATCGGAGAAGCTTCAGTAGAATACGGATG

TUR213|AB863178.1| GTTGAAGAAATTCAACAAGAAATGCTGTAGCATCGGAGAAGCTTCAGTAGAATATGGATG

TUR214|AB863179.1| GTTGAAGAAATTCAACAAGAAATGCTGTAGCATCGGAGAAGCTTCAGTAGAATATGGATG

TUR216|AB863180.1| GTTGAAGAAATTCAACAAAAAGTGCTGTAGCATCGGAGAAGCTTCAGTAGAATACGGATG

TUR220|AB863181.1| GTTGAAGAAATTCAACAAGAAGTGCTGTAGCATCGGAGAAGCTTCAGTAGAATATGGATG

TUR239|AB863182.1| GTTGAAGAAATTCAACAAGAAGTGCTGTAGCATCGGAGAAGCTTCAGTAGAATATGGGTG

TUR244|AB863183.1| GTTGAAGAAATTCAACAAAAAGTGCTGTAGCATCGGAGAAGCTTCAGTAGAATATGGATG

TUR249|AB863184.1| GTTGAAGAAATTCAACAAGAAGTGCTGTAGCATCGGAGAAGCTTCAGTAGAATATGGATG

TUR263|AB863185.1| GTTGAAGAAATTCAACAAGAAGTGCTGTAGCATCGGAGAAGCTTCAGTAGAATATGGATG

TUR278|AB863186.1| GTTGAAGAAATTCAACAAGAAGTGCTGTAGCATCGGAGAAGCTTCAGTAGAATATGGATG

TUR279|AB863187.1| GTTGAAGAAATTCAACAAGAAGTGCTGTAGCATCGGAGAAGCTCCAGTAGAATATGGATG

TUR285|AB863188.1| GTTGAAGAAATTCAACAAAAAGTGCTGTAGCATCGGAGAAGCTTCAGTAGAATATGGATG

TUR289|AB863189.1| GTTGAAGAAATTCAACAAAAAGTGCTGTAGCATCGGAGAAGCTTCAGTAGAATATGGATG

TUR303|AB863190.1| GTTGAAGAAATTCAGCAAAAAGTGCTGTAGCATCGGAGAAGCTTCAGTAGAATATGGATG

TUR306|AB863191.1| GTTGAAGAAATTCAACAAGAAGTGCTGTAGCATCGGAGAAGCTTCAGTAGAATATGGGTG

Cabb-S|NC_001497.1| CAAGAAGACATCCACAAAGAAGTATCACAAGAAGCGATACAAGAAAAAATATAAGGCTTA

CRO180A|AB863192.1| CAAGAAGACATCCAAGAAGAAGTATCATA---AAAGATACAAGAAAAAATATAAGGCTTA

GRC83|AB863193.1| CAAGAAGACATCCAAGAAGAAGTATCATA---AGAGATACAAGAAAAAATATAAGGCTTA

GRC84B|AB863194.1| CAAGAAGACATCCAAGAAGAAGTATCATA---AGAGATACAAGAAAAAATATAAGGTTTA

GRC86B|AB863195.1| CAAGAAGACATCCAAGAAGAAGTATCATA---AGAGATACAAGAAAAAATATAAGGCTTA

GRC86D|AB863196.1| CAAGAAGACATCCAAGAAGAAGTATCATA---AGAGATACAAGAAAAAATATAAGGCTTA

GRC87E|AB863197.1| CAAGAAGACATCCAAGAAGAAGTATCATA---AGAGATACAAGAAAAAATATAAGGCTTA

GRC87G|AB863198.1| CAAGAAGACATCCAAGAAGAAGTATCATA---AGAAATACAAGAAAAAATATAAGGCTTA

GRC91B|AB863199.1| CAAGAAGACATCCAAGAAGAAGTATCATA---AGAGATACAAGAAAAAATATAAGGCTTA

GRC92A|AB863200.1| CAAGAAAACATCCAAGAAGAAGTATCATA---AGAGATACAAGAAAAAATATAAGGCTTA

GRC92C|AB863201.1| CAAGAAGACATCCAAGAAGAAGTATCATA---AGAGATACAAGAAAAAATATAAGGCTTA

GRC92D|AB863202.1| CAAGAAGACATCCAAGAAGAAGTATCATA---AGAGATACAAGAAAAAATATAAGGCTTA

IRN1|AB863136.1| CAAGAAGACATCCAAGAAGAAGTATCATA---AAAGATACAAGAAAAAATATAAGGCTTA

IRN2|AB863137.1| CAAGAAAACATCCAAGAAAAAGTATCACA---AACGATACAAGAAGAAATATAAGGTCTA

IRN3|AB863138.1| CAAGAAGACATCCAAGAAGAAGTATCATA---AAAGATACAAGAAAAAATATAAGGCTTA

IRN4|AB863139.1| CAAGAAGACATCCAAGAAGAAGTATCATA---AAAGATACAAGAAAAAATATAAGGCTTA

IRN5|AB863140.1| CAAGAAGACATCCAAGAAAAAGTATCATA---AAAGATACAAGAAAAAATATAAGGCTTA

IRN6|AB863141.1| CAAGAAAACATCCAAGAAAAAGTATCACA---AACGATACAAGAAGAAATATAAGGTCTA

IRN7|AB863142.1| CAAGAAGACATCCAAGAAGAAGTATCATA---AAAGATACAAGAAAAAATATAAGGCTTA

IRN8|AB863143.1| CAAGAAGACATCCAAGAAAAAGTATCATA---AAAGATACAAGAAAAAATATAAGGCTTA

IRN9|AB863144.1| CAAGAAGACATCCAAGAAGAAGTATCATA---AAAGATACAAGAAGAAATATAAGGTCTA

IRN10|AB863145.1| CAAGAAGACATCCAAGAAGAAGTATCATA---AAAGATACAAGAAGAAATATAAGGCTTA

IRN11|AB863146.1| CAAGAAAACATCCAAGAAAAAGTATCACA---AACGATACAAGAAGAAATATAAGGTCTA

IRN12|AB863147.1| CAAGAAGACATCCAAGAAGAAGTATCATA---AAAGATACAAGAAAAAATATAAGGCTTA

IRN13|AB863148.1| CAAGAAGACATCCAAGAAGAAGTATCATA---AAAGATACAAGAAAAAATATAAGGCTTA

IRN14|AB863149.1| CAAGAAGACATCCAAGAAGAAGTATCATA---AAAGATACAAGAAGAAATATAAGGTCTA

IRN15|AB863150.1| CAAGAAGACATCCAAGAAGAAGTATCATA---AGAGATACAAGAAAAAATATAAGGCTTA

IRN16|AB863151.1| CAAGAAGACATCCAAGAAGAAGTATCATA---AGAGATACAAGAAAAAATATAAGGCTTA

IRN17|AB863152.1| CAAGAAGACATCCAAGAAAAAGTATCATA---AGAGATACAAGAAAAAATATAAGGCTTA

IRN18|AB863153.1| CAAGAAAACATCCAAGAAAAAGTATCACA---AACGATACAAGAAGAAATATAAGGTCTA

IRN19|AB863154.1| CAAGAAAACATCCAAGAAAAAGTATCACA---AACGATACAAGAAGAAATATAAGGTCTA

IRN20|AB863155.1| CAAGAAGACATCCAAGAAGAAGTATCATA---AGAGATACAAGAAAAAATATAAGGCTTA

IRN21|AB863156.1| CAAGAAAACATCCAAGAAAAAGTATCACA---AACGATACAAGAAGAAGTATAAGGTCTA

JPNHGB340|AB863157.1| CAAGAAAACATCCAAGAAGAAGTATCACA---AGCGATACAAGAAAAAATATAAGGTCTA

JPNKWB778|AB863158.1| CAAGAAAACATCCAAGAAGAAGTATCACA---AGCGATACAAGAAAAAATATAAGGTCTA

JPNM|AB863159.1| CAAGAAAACATCCAAGAAGAAGTATC---ATAAGCGATACAAGAAAAAATATAAGGTCTA

JPNN|AB863160.1| TAAGAAGACATCCACCAAGAAGTATCACAAGAAGCGATACAGAAAAAAATATAAGGCTTA

JPNS1|AB863161.1| CAAGAAGACATCTACCAAGAAGTATCACAAGAAGCGATACAGGAAAAAATATAAGGCCTA

JPNS2|AB863162.1| CAAGAAGACATCTACCAAGAAGTATCACAAGAAGCGATACAGGAAAAAATATAAGGCCTA

JPNTKD762|AB863163.1| CAAGAAAACATCCAAGAAGAAGTATCGCA---AGCGATACAAGAAAAAATATAAGGAATA

JPNUV1|AB863164.1| CAAGAAAACATCCAAGAAGAAGTATC---ATAAGCGATACAAGAAAAAATATAAGGTCTA

JPNUV26|AB863165.1| CAAGAAAACATCCAAGAAGAAGTATCATA---AGCGATACAAGAAAAAATATAAGGTCTA

TUR1|AB863166.1| CAAGAAAACATCCAAGAAGAAGTATCACA---AGCGATACAAGAAAAAATATAAGGCTTA

TUR2|AB863167.1| CAAGAAAACATCCAAGAAGAAGTATCACA---AGCGATACAAAAAGAAATATAAGGCCTA

TUR4|AB863168.1| CAAGAAAACATCCAAGAAGAAGTATCACA---AGCGATACAAAAAGAAATATAAGGCCTA

TUR5|AB863169.1| CAAAAAGACATCCAAGAAGAAGTATCATA---AGAGATACAAGAAAAAATATAAGACTTA

TUR12|AB863170.1| CAAGAAGACATCTAAGAAGAAGTATCATA---AGAAATACAAGAAAAAATATAAGGCTTA

TUR34|AB863171.1| CAAGAAGACATCCAAGAAGAAGTATCATA---AGAGATACAAGAAAAAATATAAGACTTA

TUR50|AB863172.1| CAAGAAGACATCCAAGAAGAAGTATCATA---AGAGATACAAGAAAAAATATAAGGCTTA

TUR59|AB863173.1| CAAGAAGACATCCAAGAAGAAGTATCATA---AGCGATACAAGAAAAAATATAAGGCTTA

TUR69|AB863174.1| CAAGAAAACATCCAAGAAGAAGTATCACA---AGCGATACAAAAAGAAATATAAGGCTTA

TUR81|AB863175.1| CAAGAAAACATCCAAGAAGAAGTATCACA---AGCGATACAAAAAGAAATATAAGGCTTA

TUR84|AB863176.1| CAAGAAAACATCCAAGAAGAAGTATCACA---AGCGATACAAAAAGAAATATAAGGCTTA

TUR94|AB863177.1| CAAGAAGACATCCAAGAAGAAGTATCATA---AGAGATACAAGAAAAAATATAAGGCTTA

TUR213|AB863178.1| CAAGAAAACATCCAAGAAGAAGTATCACA---AGCGATACAAGAAAAAATATAAGGCTTA

TUR214|AB863179.1| CAAGAAAACATCCAAGAAGAAGTATCACA---AGCGATACAAAAAGAAATATAAGGCTTA

TUR216|AB863180.1| CAAGAAGACGTCCCCTAAGAAGTATCACA---AGCGATACAAGAAGAAATATAAGGCTTA

TUR220|AB863181.1| CAAGAAAACATCCAAGAAGAAGTATCACA---AGCGATACAAAAAGAAATATAAGGCTTA

TUR239|AB863182.1| CAAGAAGACATCCAAGAAGAAGTATCATA---AGAGATACAAGAAAAAATATAAGGCTTA

TUR244|AB863183.1| CAAGAAAACATCCAAGAAGAAGTATCACA---AGCGATACAAAAAGAAATATAAGGCCTA

TUR249|AB863184.1| CAAGAAAACATCCAAGAAGAAGTATCACA---AGCGATACAAAAAGAAATATAAGGCCTA

TUR263|AB863185.1| CAAGAAGACATCCAAAAAGAAGTATCATA---AAAGATACAAGAAAAAATATAAGGCTTA

TUR278|AB863186.1| CAAGAAAACATCCAAGAAGAAGTATCACA---AGCGATACAAAAAGAAATATAAGGCTTA

TUR279|AB863187.1| CAAGAAAACATCCAAGAAGAAGTATCACA---AGCGATACAAAAAGAAATATAAGGCTTA

TUR285|AB863188.1| CAAGAAGACATCCAAGAAGAAGTATCATA---AGAGATACAAGAAAAAATATAAGGCTTA

TUR289|AB863189.1| CAAGAAAACATCCAAGAAGAAGTATCATA---AGAGATACAAGAAAAAATATAAGGCTTA

TUR303|AB863190.1| CAAGAAAACATCCAAGAAGAAGTATCACA---AGCGATACAAAAAGAAATATAAGGCTTA

TUR306|AB863191.1| CAAGAAGACATCCAAGAAGAAGTATCATA---AGAGATACAAGAAAAAATATAAGGCTTA

Cabb-S|NC_001497.1| CAAACCTTATAAGAAGAAAAAGAAGTTCCGATCAGGAAAATACTTCAAGCCCAAAGAAAA

CRO180A|AB863192.1| CAAACCTTATAAGAAGAAGAAGAAATTCCGGTCAGGAAAATACTTCAAGCCCAAAGAAAA

GRC83|AB863193.1| CAAACCTTATAAGAAGAAGAAGAAATTCCGTTCAGGAAAATACTTCAAGCCCAAAGAGAA

GRC84B|AB863194.1| CAAACCTTATAAGAAGAAGAAGAAATTCCGGTCAGGAAAATACTTCAAGCCCAAAGAGAA

GRC86B|AB863195.1| CAAACCTTATAAGAAGAAGAAGAAATTCCGGTCAGGAAAATACTTCAAGCCCAAAGAGAA

GRC86D|AB863196.1| CAAACCTTATAAGAAGAAGAAGAAATTCCGTTCAGGAAAATACTTCAAGCCCAAAGAGAA

GRC87E|AB863197.1| CAAACCTTATAAGAAGAAGAAGAAATTCCGTTCAGGAAAATACTTCAAGCCCAAAGAGAA

GRC87G|AB863198.1| CAAACCTTATAAGAAGAAGAAGAAATTCCGGTCAGGAAAATACTTCAAGCCCAAAGAGAA

GRC91B|AB863199.1| CAAACCTTATAAGAAGAAGAAGAAATTCCGGTCAGGAAAATACTTCAAGCCCAAAGAGAA

GRC92A|AB863200.1| CAAACCTTATAAGAAGAAGAAGAAATTCCGGTCAGGAAAATACTTCAAGCCCAAAGAGAA

GRC92C|AB863201.1| CAAACCTTATAAGAAGAAGAAGAAATTCCGGTCAGGAAAATACTTCAAGCCCAAAGAGAA

GRC92D|AB863202.1| TAAACCTTATAAGAAGAAGAAGAAATTCCGGTCAGGAAAATACTTCAAGCCCAAAGAGAA

IRN1|AB863136.1| TAAACCTTATAAGAAGAAGAAGAAATTCCGATCCGGAAAATACTTCAAGCCCAAAGAAAA

IRN2|AB863137.1| TAAACCTTATAAGAAGAAGAAGAAATTCCGATCCGGAAAATACTTCAAGCCCAAAGAGAA

IRN3|AB863138.1| TAAACCTTATAAGAAGAAGAAGAAATTCCGATCCGGAAAATACTTCAAGCCCAAAGAAAA

IRN4|AB863139.1| TAAACCTTATAAGAAGAAGAAGAAATTCCGATCCGGAAAATACTTCAAGCCCAAAGAAAA

IRN5|AB863140.1| TAAACCTTATAAGAAGAAGAAGAAATTCCGATCCGGAAAATACTTCAAGCCCAAAGAAAA

IRN6|AB863141.1| TAAACCTTATAAGAAGAAGAAGAAATTCCGATCCGGAAAATACTTCAAGCCCAAAGAGAA

IRN7|AB863142.1| TAAACCTTATAAGAAGAAGAAGAAATTCCGATCCGGAAAATACTTCAAGCCCAAAGAAAA

IRN8|AB863143.1| TAAACCTTATAAGAAGAAGAAGAAATTCCGATCCGGAAAATACTTCAAGCCCAAAGAAAA

IRN9|AB863144.1| TAAACCTTATAAGAAGAAGAAGAAATTCCGATCCGGAAAATACTTCAAGCCCAAAGAGAA

IRN10|AB863145.1| TAAACCTTATAAGAAGAAGAAGAAATTCCGATCCGGAAAATACTTCAAGCCTAAAGAAAA

IRN11|AB863146.1| TAAACCTTATAAGAAGAAGAAGAAATTCCGATCCGGAAAATACTTCAAGCCCAAAGAGAA

IRN12|AB863147.1| TAAACCTTATAAGAAGAAGAAGAAATTCCGATCCGGAAAATACTTCAAGCCCAAAGAGAA

IRN13|AB863148.1| TAAACCTTATAAGAAGAAGAAGAAATTCCGATCCGGAAAATACTTCAAGCCCAAAGAGAA

IRN14|AB863149.1| TAAACCTTATAAGAAGAAGAAGAAATTCCGATCCGGAAAATACTTCAAGCCCAAAGAGAA

IRN15|AB863150.1| CAAACCTTATAAGAAGAAGAAGAAATTCCGGTCAGGAAAATACTTCAAGCCCAAAGAGAA

IRN16|AB863151.1| CAAACCTTATAAGAAGAAGAAGAAATTCCGGTCAGGAAAATACTTCAAGCCCAAAGAGAA

IRN17|AB863152.1| CAAACCTTATAAGAAGAAGAAGAAATTCCGGTCAGGAAAATACTTCAAGCCCAAAGAGAA

IRN18|AB863153.1| TAAACCTTATAAGAAGAAGAAGAAATTCCGATCCGGAAAATACTTCAAGCCCAAAGAGAA

IRN19|AB863154.1| TAAACCTTATAAGAAGAAGAAGAAATTCCGATCCGGAAAATACTTCAAGCCCAAAGAGAA

IRN20|AB863155.1| CAAACCTTATAAGAAGAAGAAGAAATTCCGGTCAGGAAAATACTTCAAGCCCAAAGAGAA

IRN21|AB863156.1| TAAACCTTATAAAAAGAAGAAGAAATTCCGATCCGGAAAATACTTCAAGCCCAAAGAGAA

JPNHGB340|AB863157.1| TAAACCTTATAAGAAGAAGAAGAAATTCCGATCCGGAAAATACTTCA------AAGAAAA

JPNKWB778|AB863158.1| TAAACCTTATAAGAAGAAGAAGAAATTCCGATCCGGAAAATACTTCAAGCCCAAAGAAAA

JPNM|AB863159.1| TAAACCTTATAAGAAGAAGAAGAAATTCCGATCCGGAAAATACTTCAAGCCCAAAGAAAA

JPNN|AB863160.1| TAAACCTTATAAGAAGAAGAAGAAATTCCGATCAGGAAAATACTTCAAGCCCAAGGAGAA

JPNS1|AB863161.1| TAAACCTTATAAGAAGAAGAAGAAATTCCGATCAGGAAAATACTTCAAGCCCAAGGAGAA

JPNS2|AB863162.1| TAAACCTTATAAGAAGAAGAAGAAATTCCGATCAGGAAAATACTTCAAGCCCAAGGAGAA

JPNTKD762|AB863163.1| TAAACCTTATAAGAAGAAGAAGAAATTCCGATCCGGAAAATACTTCAAGCCCAAAGAAAA

JPNUV1|AB863164.1| TAAACCTTATAAGAAGAAGAAGAAATTCCGATCCGGAAAATACTTCAAGCCCAAAGAAAA

JPNUV26|AB863165.1| TAAACCTTATAAGAAGAAGAAGAAATTCCGATCCGGAAAATACTTCAAGCCCAAAGAAAA

TUR1|AB863166.1| CAAACCTTATAAGAAGAAAAAGAAATTCCGGTCAGGAAAATACTTCAAGCCCAAAGAGAA

TUR2|AB863167.1| TAAACCTTATAAGAAGAAGAAGAAATTCCGGTCAGGAAAATACTTCAAGCCCAAAGAGAA

TUR4|AB863168.1| TAAACCTTATAAGAAGAAGAAGAAATTCCGGTCAGGAAAATACTTCAAGCCCAAAGAGAA

TUR5|AB863169.1| CAAACCTTATAAGAAGAAGAAGAAATTCCGATCAGGAAAATACTTCAAGCCCAAAGAGAA

TUR12|AB863170.1| TAAACCTTATAAAAAGAAGAAGAAATTCCGATCAGGAAAATACTTCAAGCCCAAAGAGAA

TUR34|AB863171.1| CAAACCTTATAAGAAGAAGAAGAAATTCCGGTCAGGAAAATACTTCAAGCCCAAAGAGAA

TUR50|AB863172.1| CAAACCTTATAAGAAGAAGAAGAAATTCCGTTCAGGAAAATACTTCAAGCCCAAAGAGAA

TUR59|AB863173.1| CAAACCTTATAAGAAAAAGAAGAAATTCCGGTCAGGAAAATACTTCAAGCCCAAAGAGAA

TUR69|AB863174.1| CAAACCTTATAAGAAGAAGAAGAAATTCCGATCAGGAAAATACTTCAAGCCCAAAGAGAA

TUR81|AB863175.1| CAAACCTTATAAGAAGAAGAAGAAATTCCGGTCAGGAAAATACTTCAAGCCCAAAGAGAA

TUR84|AB863176.1| CAAACCTTATAAGAAGAAGAAGAAATTCCGATCAGGAAAATACTTCAAGCCCAAAGAGAA

TUR94|AB863177.1| CAAACCTTATAAGAAGAAGAAGAAATTCCGGTCAGGAAAATACTTCAAGCCCAAAGAGAA

TUR213|AB863178.1| CAAACCTTATAAGAAGAAGAAGAAATTCCGGTCAGGAAAATACTTCAAGCCCAAAGAGAA

TUR214|AB863179.1| CAAACCTTATAAGAAGAAGAAGAAATTCCGGTCAGGAAAATACTTCAAGCCCAAAGAGAA

TUR216|AB863180.1| TAAACCTTATAAGAAGAAGAAGAAATTCCGATCCGGAAAATACTTCAAGCCCAAAGAGAA

TUR220|AB863181.1| CAAACCTTATAAGAAGAAGAAGAAATTCCGATCAGGAAAATACTTCAAGCCCAAAGAGAA

TUR239|AB863182.1| CAAACCTTATAAGAAGAAGAAGAAATTCCGATCAGGAAAATACTTCAAGCCCAAAGAGAA

TUR244|AB863183.1| TAAACCTTATAAGAAGAAGAAGAAATTCCGGTCAGGAAAATACTTCAAGCCCAAAGAGAA

TUR249|AB863184.1| TAAACCTTATAAGAAGAAGAAGAAATTCCGGTCAGGAAAATACTTCAAGCCCAAAGAGAA

TUR263|AB863185.1| TAAACCTTATAAGAAGAAGAAGAAATTCCGATCCGGAAAATACTTCAAGCCCAAAGAGAA

TUR278|AB863186.1| CAAACCTTATAAGAAGAAGAAGAAATTCCGATCAGGAAAATACTTCAAGCCCAAAGAGAA

TUR279|AB863187.1| CAAACCTTATAAGAAGAAGAAGAAATTCCGATCAGGAAAATACTTCAAGCCCAAAGAGAA

TUR285|AB863188.1| CAAACCTTATAAGAAAAAGAAGAAATTCCGGTCAGGAAAATACTTCAAGCCCAAAGAGAA

TUR289|AB863189.1| CAAACCTTATAAAAAGAAGAAGAAATTCCGATCAGGAAAATACTTCAAGCCCAAAGAGAA

TUR303|AB863190.1| CAAACCTTATAAGAAGAAGAAGAAATTCCGGTCAGGAAAATACTTCAAGCCCAAAGAGAA

TUR306|AB863191.1| CAAACCTTATAAGAAGAAGAAGAAATTCCGATCAGGAAAATACTTCAAGCCCAAAGAGAA

Cabb-S|NC_001497.1| GAAGGGCTCAAAGCAAAAGTATTGCCCAAAAGGCAAGAAAGATTGCAGATGTTGGATCTG

CRO180A|AB863192.1| GAAGGGCTCTAAGCAAAAGTACTGCCCAAAAGGCAAGAAAGACTGCAGATGTTGGATCTG

GRC83|AB863193.1| GAAGGGCTCCAAGCAAAAATATTGCCCAAAAGGCAAGAAAGATTGCAGATGTTGGATCTG

GRC84B|AB863194.1| GAAGGGCTCAAAACAAAAGTATTGCCCAAAAGGCAAGAAAGATTGCAGATGTTGGATCTG

GRC86B|AB863195.1| GAAGGGCTCAAAACAAAAGTATTGCCCAAAAGGCAAGAAAGACTGCAGATGTTGGATCTG

GRC86D|AB863196.1| GAAGGGCTCCAAGCAAAAATATTGCCCAAAAGGCAAGAAAGATTGCAGATGTTGGATCTG

GRC87E|AB863197.1| GAAGGGCTCAAAGCAAAAGTATTGCCCAAAAGGCAAGAAAGACTGCAGATGTTGGATCTG

GRC87G|AB863198.1| GAAGGGCTCAAAGCAAAAGTATTGCCCAAAAGGCAAGAAAGACTGCAGATGTTGGATCTG

GRC91B|AB863199.1| GAAGGGCTCAAAGCAAAAGTATTGCCCAAAAGGCAAGAAAGACTGCAGATGTTGGATCTG

GRC92A|AB863200.1| GAAGGGCTCAAAACAAAAGTATTGCCCAAAAGGCAAGAAAGATTGCAGATGTTGGATCTG

GRC92C|AB863201.1| GAAGGGCTCAAAACAAAAGTATTGCCCAAAAGGCAAGAAAGATTGCAGATGTTGGATCTG

GRC92D|AB863202.1| GAAGGGCTCAAAACAAAAGTATTGCCCAAAAGGCAAGAAAGACTGCAGATGTTGGATCTG

IRN1|AB863136.1| GAAGGGCTCAAAGCAAAAATATTGCCCAAAAGGCAAGAAAGATTGCAGATGTTGGATCTG

IRN2|AB863137.1| GAAGGGCTCAAAACAAAAGTATTGCCCGAAAGGCAAGAAAGACTGCAGATGTTGGATCTG

IRN3|AB863138.1| GAAGGGCTCAAAGCAAAAATATTGCCCAAAAGGCAAGAAAGATTGCAGATGTTGGATCTG

IRN4|AB863139.1| GAAAGGCTCAAAGCAAAAGTATTGCCCAAAAGGCAAGAAAGATTGCAGATGTTGGATCTG

IRN5|AB863140.1| GAAGGGTTCAAAGCAAAAGTATTGCCCAAAAGGCAAGAAAGATTGCAGATGTTGGATCTG

IRN6|AB863141.1| GAAGGGCTCAAAACAAAAGTATTGCCCAAAAGGCAAGAAAGACTGCAGATGTTGGATCTG

IRN7|AB863142.1| GAAGGGCTCAAAACAAAAGTACTGCCCAAAAGGCAAGAAAGACTGCAGATGTTGGATCTG

IRN8|AB863143.1| GAAGGGTTCAAAGCAAAAGTATTGCCCAAAAGGCAAGAAAGATTGCAGATGTTGGATCTG

IRN9|AB863144.1| GAAGGGCTCAAAGCAAAAGTATTGCCCAAAAGGCAAGAAAGATTGCAGATGTTGGATCTG

IRN10|AB863145.1| GAAGGGCTCAAAACAAAAGTATTGCCCAAAAGGCAAGAAGGATTGCAGATGTTGGATCTG

IRN11|AB863146.1| GAAGGGCTCAAAACAAAAGTATTGCCCAAAAGGCAAGAAAGACTGCAGATGTTGGATCTG

IRN12|AB863147.1| GAAGGGCTCAAAGCAAAAGTATTGCCCAAAAGGCAAGAAAGATTGCAGATGTTGGATCTG

IRN13|AB863148.1| GAAGGGCTCAAAGCAAAAGTATTGCCCAAAAGGCAAGAAAGATTGCAGATGTTGGATCTG

IRN14|AB863149.1| GAAGGGCTCAAAACAAAAGTATTGCCCAAAAGGCAAGAAAGACTGCAGATGTTGGATCTG

IRN15|AB863150.1| GAAGGGCTCTAAGCAAAAGTATTGCCCAAAAGGCAAGAAAGATTGCAGATGTTGGATCTG

IRN16|AB863151.1| GAAGGGCTCTAAGCAAAAGTATTGCCCAAAAGGCAAGAAAGATTGCAGATGTTGGATCTG

IRN17|AB863152.1| GAGGGGCTCTAAGCAAAAGTATTGCCCAAAAGGCAAGAAAGATTGCAGATGTTGGATCTG

IRN18|AB863153.1| GAAGGGCTCAAAACAAAAGTATTGCCCAAAAGGCAAGAAAGACTGCAGATGTTGGATCTG

IRN19|AB863154.1| GAAGGGCTCAAAACAAAAGTATTGCCCAAAAGGCAAGAAAGACTGCAGATGTTGGATCTG

IRN20|AB863155.1| GAAGGGCTCTAAGCAAAAGTATTGCCCAAAAGGCAAGAAAGATTGCAGATGTTGGATCTG

IRN21|AB863156.1| GAAGGGCTCAAAACAAAAGTATTGCCCAAAAGGCAAGAAAGACTGCAGATGTTGGATCTG

JPNHGB340|AB863157.1| GAAGGGCTCAAAGCAAAAGTATTGCCCAAAAGGCAAGAAAGATTGCAGATGTTGGATCTG

JPNKWB778|AB863158.1| GAAGGGCTCAAAGCAAAAGTATTGCCCAAAAGGCAAGAAAGACTGCAGATGTTGGATCTG

JPNM|AB863159.1| GAAGGGCTCAAAGCAAAAGTATTGCCCAAAAGGCAAGAAAGACTGCAGATGTTGGATCTG

JPNN|AB863160.1| GAAGGGCTTAAAGCAAAAGTATTGCCCAAAAGGCAAGAAAGATTGCAGATGTTGGATCTG

JPNS1|AB863161.1| GAAGGGCTCAAAGCAAAAGTATTGCCCAAAAGGCAAGAAAGACTGCAGATGTTGGATCTG

JPNS2|AB863162.1| GAAGGGCTCAAAGCAAAAGTATTGCCCAAAAGGCAAGAAAGACTGCAGATGTTGGATCTG

JPNTKD762|AB863163.1| GAAGGGCTCAAAGCAAAAGTATTGCCCAAAAGGCAAGAAAGACTGCAGATGTTGGATCTG

JPNUV1|AB863164.1| GAAGGGCTCAAAGCAAAAGTATTGCCCAAAAGGCAAGAAAGACTGCAGATGTTGGATCTG

JPNUV26|AB863165.1| GAAGGGCTCAAAGCAAAAGTATTGCCCAAAAGGCAAGAAAGACTGCAGATGTTGGATCTG

TUR1|AB863166.1| GAAGGGCTCAAAACAAAAGTATTGCCCTAAAGGCAAGAAAGACTGCAGATGTTGGATCTG

TUR2|AB863167.1| GAAGGGCTCAAAGCAAAAATATTGCCCTAAAGGCAAGAAAGACTGCAGATGTTGGATCTG

TUR4|AB863168.1| GAAGGGCTCAAAGCAAAAATATTGCCCTAAAGGCAAGAAAGACTGCAGATGTTGGATCTG

TUR5|AB863169.1| GAAGGGCTCAAAGCAAAAGTATTGCCCAAAAGGCAAGAAAGACTGCAGATGTTGGATCTG

TUR12|AB863170.1| GAAGGGCTCAAAGCAAAAGTACTGCCCTAAAGGCAAGAAAGACTGCAGATGTTGGATCTG

TUR34|AB863171.1| GAAGGGCTCAAAGCAAAAGTATTGCCCAAAAGGCAAGAAAGATTGCAGATGTTGGATCTG

TUR50|AB863172.1| GAAGGGCTCAAAGCAAAAGTATTGCCCAAAAGGCAAGAAAGATTGCAGATGTTGGATCTG

TUR59|AB863173.1| GAAGGGCTCAAAACAAAAGTATTGTCCAAAAGGCAAGAAAGACTGCAGATGTTGGATCTG

TUR69|AB863174.1| GAAGGGCTCAAAACAAAAGTATTGCCCAAAAGGCAAGAAAGACTGCAGATGTTGGATCTG

TUR81|AB863175.1| GAAGGGCTCAAAACAAAAGTATTGCCCAAAAGGCAAGAAAGACTGCAGATGTTGGATCTG

TUR84|AB863176.1| GAAGGGCTCAAAACAAAAGTATTGCCCAAAAGGCAAGAAAGACTGCAGATGTTGGATCTG

TUR94|AB863177.1| GAAGGGCTCTAAGCAAAAGTATTGCCCAAAAGGCAAGAAAGACTGCAGATGTTGGATCTG

TUR213|AB863178.1| GAAGGGCTCAAAACAAAAGTATTGCCCTAAAGGCAAGAAAGACTGCAGATGTTGGATCTG

TUR214|AB863179.1| GAAGGGCTCAAAACAAAAGTATTGCCCAAAAGGCAAGAAAGACTGCAGATGTTGGATCTG

TUR216|AB863180.1| AAAGGGCTCAAAGCAAAAGTATTGCCCGAAAGGCAAGAAAGACTGCAGATGTTGGATCTG

TUR220|AB863181.1| GAAGGGCTCAAAACAAAAGTATTGCCCAAAAGGCAAGAAAGACTGCAGATGTTGGATCTG

TUR239|AB863182.1| GAAGGGCTCTAAGCAAAAGTATTGCCCAAAAGGCAAGAAAGATTGCAGATGCTGGATCTG

TUR244|AB863183.1| GAAGGGCTCAAAGCAAAAGTATTGCCCTAAAGGCAAGAAAGACTGCAGATGTTGGATCTG

TUR249|AB863184.1| GAAGGGCTCAAAGCAAAAGTATTGCCCTAAAGGCAAGAAAGACTGCAGATGTTGGATCTG

TUR263|AB863185.1| GAAGGGCTCAAAGCAAAAGTATTGCCCTAAAGGCAAGAAAGACTGCAGATGTTGGATCTG

TUR278|AB863186.1| GAAGGGCTCAAAACAAAAGTATTGCCCAAAAGGCAAGAAAGACTGCAGATGTTGGATCTG

TUR279|AB863187.1| GAAGGGCTCAAAACAAAAGTATTGCCCAAAAGGCAAGAAAGACTGCAGATGTTGGATCTG

TUR285|AB863188.1| GAAGGGCTCAAAACAAAAATATTGCCCAAAAGGCAAGAAAGACTGCAGATGTTGGATCTG

TUR289|AB863189.1| GAAGGGCTCAAAACAAAAGTATTGCCCAAAAGGCAAGAAAGACTGCAGATGTTGGATCTG

TUR303|AB863190.1| GAAGGGCTCAAAGCAAAAGTATTGCCCTAAAGGCAAGAAAGACTGCAGATGTTGGATCTG

TUR306|AB863191.1| GAAGGGCTCTAAGCAAAAGTATTGCCCAAAAGGCAAGAAAGATTGCAGATGCTGGATCTG

Cabb-S|NC_001497.1| CAACATTGAAGGCCATTACGCCAACGAATGTCCTAATCGACAAAGCTCGGAGAAGGCTCA

CRO180A|AB863192.1| CAATATCGAAGGCCATTACGCCAACGAATGTCCTAATCGACAAAGCTCGGAGAAGGCTCA

GRC83|AB863193.1| CAATATCGAAGGCCATTACGCCAACGAATGTCCTAATCGACAAAGCTCGGAGAAGGCTCA

GRC84B|AB863194.1| CAATATCGAAGGCCATTACGCCAACGAATGTCCTAATCGACAAAGCTCGGAGAAGGCTCA

GRC86B|AB863195.1| CAATATCGAAGGCCATTACGCCAACGAATGTCCTAATCGACAAAGCTCGGAGAAGGCTCA

GRC86D|AB863196.1| CAATATCGAAGGCCATTACGCCAACGAATGTCCTAATCGACAAAGCTCGGAGAAGGCTCA

GRC87E|AB863197.1| CAATATCGAAGGCCATTACGCCAACGAATGTCCTAATCGACAAAGCTCGGAGAAGGCTCA

GRC87G|AB863198.1| CAATATCGAAGGCCATTACGCCAACGAATGTCCTAATCGACAAAGCTCGGAGAAGGCTCA

GRC91B|AB863199.1| CAATATCGAAGGCCATTACGCCAACGAATGTCCTAATCGACAAAGCTCGGAGAAGGCTCA

GRC92A|AB863200.1| CAATATCGAAGGCCATTACGCCAACGAATGTCCTAATCGACAAAGCTCGGAGAAGGCTCA

GRC92C|AB863201.1| CAATATCGAAGGCCATTACGCCAACGAATGTCCTAATCGACAAAGCTCAGAGAAGGCTCA

GRC92D|AB863202.1| CAATATCGAAGGCCATTACGCCAACGAATGTCCTAATCGACAAAGCTCGGAGAAGGCTCA

IRN1|AB863136.1| CAACATAGAAGGCCATTACGCCAACGAATGTCCTAATCGACAAAGCTCGGAGAAGGCTCA

IRN2|AB863137.1| CAATATCGAAGGCCATTACGCCAACGAATGTCCTAATCGACAAAGCTCAGAGAAGGCTCA

IRN3|AB863138.1| CAACATAGAAGGCCATTACGCCAACGAATGTCCTAATCGACAAAGCTCGGAGAAGGCTCA

IRN4|AB863139.1| CAACATAGAAGGCCATTACGCCAACGAATGTCCTAATCGACAAAGCTCGGAGAAGGCTCA

IRN5|AB863140.1| CAACATAGAAGGCCATTACGCCAACGAATGTCCTAATCGACAAAGCTCGGAGAAGGCTCA

IRN6|AB863141.1| CAATATCGAAGGCCATTACGCCAACGAATGTCCTAATCGACAAAGCTCGGAGAAGGCTCA

IRN7|AB863142.1| CAATATCGAAGGCCATTACGCCAACGAATGTCCTAATCGACAAAGCTCGGAGAAGGCTCA

IRN8|AB863143.1| CAACATAGAAGGCCATTACGCCAACGAATGTCCTAATCGACAAAGCTCGGAGAAGGCTCA

IRN9|AB863144.1| CAACATAGAAGGCCATTACGCCAACGAATGTCCTAACCGACAAAGCTCGGAGAAGGCTCA

IRN10|AB863145.1| CAATATCGAAGGCCATTACGCCAACGAATGTCCTAATCGACAAAGCTCGGAGAAGGCTCA

IRN11|AB863146.1| CAATATCGAAGGCCATTACGCCAACGAATGTCCTAATCGACAAAGCTCAGAGAAGGCTCA

IRN12|AB863147.1| CAACATAGAAGGCCATTACGCCAACGAATGTCCTAATCGACAAAGCTCGGAGAAGGCTCA

IRN13|AB863148.1| CAACATAGAAGGCCATTACGCCAACGAATGTCCTAATCGACAAAGCTCGGAGAAGGCTCA

IRN14|AB863149.1| CAATATCGAAGGCCATTACGCCAACGAATGTCCTAATCGACAAAGCTCGGAGAAGGCTCA

IRN15|AB863150.1| CAACATAGAAGGCCATTACGCCAACGAATGTCCTAATCGACAAAGCTCGGAGAAGGCTCA

IRN16|AB863151.1| CAACATAGAAGGTCATTACGCCAACGAATGTCCTAATCGACAAAGCTCGGAGAAGGCTCA

IRN17|AB863152.1| CAACATAGAAGGCCATTACGCCAACGAATGTCCTAATCGACAAAGCTCGGAGAAGGCTCA

IRN18|AB863153.1| CAATATCGAAGGCCATTACGCCAACGAATGTCCTAATCGACAAAGCTCAGAGAAGGCTCA

IRN19|AB863154.1| CAATATCGAAGGCCATTACGCCAACGAATGTCCTAATCGACAAAGCTCAGAGAAGGCTCA

IRN20|AB863155.1| CAACATAGAAGGCCATTACGCCAACGAATGTCCTAATCGACAAAGCTCGGAGAAGGCTCA

IRN21|AB863156.1| CAATATCGAAGGCCATTACGCCAACGAATGTCCTAATCGACAAAGCTCGGAGAAGGCTCA

JPNHGB340|AB863157.1| CAATATCGAAGGCCATTACGCCAACGAATGTCCTAATCGACAAAGCTCGGAGAAGGCTCA

JPNKWB778|AB863158.1| CAATATCGAAGGCCATTACGCCAACGAATGTCCTAATCGACAAAGCTCGGAGAAGGCTCA

JPNM|AB863159.1| CAATATCGAAGGTCATTACGCCAACGAATGTCCTAATCGACAAAACTCGGAGAAGGCTCA

JPNN|AB863160.1| CAATATCGAAGGCCATTACGCCAACGAATGTCCTAATCGACAAAGCTCGGAGAAGGCTCA

JPNS1|AB863161.1| CAATATCGAAGGCCATTACGCCAACGAATGTCCTAATCGACAAAGCTCGGAGAAGGCTCA

JPNS2|AB863162.1| CAATATCGAAGGCCATTACGCCAACGAATGTCCTAATCGACAAAGCTCGGAGAAGGCTCA

JPNTKD762|AB863163.1| CAATATAGAAGGCCATTACGCCAACGAATGTCCTAATCGACAAAGCTCGGAGAAGGCTCA

JPNUV1|AB863164.1| CAATATCGAAGGTCATTACGCCAACGAATGTCCTAATCGACAAAACTCGGAGAAGGCTCA

JPNUV26|AB863165.1| CAATATCGAAGGTCATTACGCCAACGAATGTCCTAATCGACAAAACTCGGAGAAGGCTCA

TUR1|AB863166.1| CAATATCGAAGGCCATTACGCCAACGAATGTCCTAATCGACAAAGCTCGGAGAAGGCTCA

TUR2|AB863167.1| CAATATCGAAGGCCATTACGCCAACGAATGTCCTAATCGACAAAGCTCAGAGAAGGCTCA

TUR4|AB863168.1| CAATATCGAAGGCCATTACGCCAACGAATGTCCTAATCGACAAAGCTCAGAGAAGGCTCA

TUR5|AB863169.1| CAATATCGAAGGCCATTACGCCAACGAATGTCCTAATCGACAAAGCTCGGAAAAGGCTCA

TUR12|AB863170.1| CAATATCGAAGGCCATTACGCCAACGAATGTCCTAATCGACAAAGCTCGGAGAAGGCTCA

TUR34|AB863171.1| CAACATAGAAGGCCATTACGCCAACGAATGTCCTAATCGACAAAGCTCGGAGAAGGCTCA

TUR50|AB863172.1| CAACATAGAAGGCCATTACGCCAACGAATGTCCTAATCGACAAAGCTCGGAGAAGGCTCA

TUR59|AB863173.1| CAATATCGAAGGCCATTACGCCAACGAATGTCCTAATCGACAAAGCTCGGAGAAGGCTCA

TUR69|AB863174.1| CAATATCGAAGGCCATTACGCCAACGAATGTCCTAATCGACAAAGCTCGGAAAAGGCTCA

TUR81|AB863175.1| CAATATCGAAGGCCATTACGCCAACGAATGTCCTAATCGACAAAGCTCGGAGAAGGCTCA

TUR84|AB863176.1| CAATATCGAAGGCCATTACGCCAACGAATGTCCTAATCGACAAAGCTCGGAGAAGGCTCA

TUR94|AB863177.1| CAATATCGAAGGCCATTACGCCAACGAATGTCCTAATCGACAAAGCTCGGAGAAGGCTCA

TUR213|AB863178.1| CAATATCGAAGGCCATTACGCCAACGAATGTCCTAATCGACAAAGCTCGGAGAAGGCTCA

TUR214|AB863179.1| CAATATCGAAGGCCATTACGCCAACGAATGTCCTAATCGACAAAGCTCGGAGAAGGCTCA

TUR216|AB863180.1| CAATATCGAAGGCCATTACGCCAACGAATGTCCTAATCGACAAAGCTCGGAGAAGGCTCA

TUR220|AB863181.1| CAATATCGAAGGCCATTACGCCAACGAATGTCCTAATCGACAAAGCTCGGAGAAGGCTCA

TUR239|AB863182.1| CAATATCGAAGGCCATTACGCCAACGAATGTCCTAATCGACAAAGCTCGGAGAAGGCTCA

TUR244|AB863183.1| CAATATCGAAGGCCATTACGCCAACGAATGTCCTAATCGACAAAGCTCGGAGAAGGCTCA

TUR249|AB863184.1| CAATATCGAAGGCCATTACGCCAACGAATGTCCTAATCGACAAAGCTCGGAGAAGGCTCA

TUR263|AB863185.1| CAATATCGAAGGCCATTACGCCAACGAATGTCCTAATCGACAAAGCTCGGAGAAGGCTCA

TUR278|AB863186.1| TAATATCGAAGGCCATTACGCCAACGAATGTCCTAATCGACAAAGCTCGGAGAAGGCTCA

TUR279|AB863187.1| CAATATCGAAGGCCATTACGCCAACGAATGTCCTAATCGACAAAGCTCGGAAAAGGCTCA

TUR285|AB863188.1| CAATATCGAAGGTCATTACGCCAACGAATGTCCTAATCGACAAAGCTCGGAGAAGGCTCA

TUR289|AB863189.1| CAATATCGAAGGCCATTACGCCAACGAATGTCCTAATCGACAAAGCTCGGAGAAGGCTCA

TUR303|AB863190.1| CAATATCGAAGGCCATTACGCCAACGAATGTCCTAATCGACAAAGCTCGGAGAAGGCTCA

TUR306|AB863191.1| CAATATCGAAGGCCATTACGCCAACGAATGTCCTAATCGACAAAGCTCGGAGAAGGCTCA

Cabb-S|NC_001497.1| CATCCTTCAACAAGCAGAAAAATTGGGTCTCCAGCCCATTGAAGAACCCTATGAAGGAGT

CRO180A|AB863192.1| TATCCTTCAACAAGCAGAGAAATTGGGCCTCCAGCCCATCGAAGAACCCTATGAAGGAGT

GRC83|AB863193.1| CATCCTTCAACAAGCAGAGAAATTGGGCCTCCAGCCCATAGAAGAACCCTACGAAGGAGT

GRC84B|AB863194.1| CATCCTTCAACAAGCAGAGAAATTGGGCCTCCAGCCCATAGAAGAACCCTACGAAGGAGT

GRC86B|AB863195.1| CATCCTTCAACAAGCAGAGAAATTGGGCCTCCAGCCCATAGAAGAACCCTACGAAGGAGT

GRC86D|AB863196.1| CATCCTTCAACAAGCAGAGAAATTGGGCCTCCAGCCCATAGAAGAACCCTACGAAGGAGT

GRC87E|AB863197.1| CATCCTTCAACAAGCAGAGAAATTGGGCCTCCAGCCCATAGAAGAACCCTACGAAGGAGT

GRC87G|AB863198.1| CATCCTTCAACAAGCAGAGAAATTGGGCCTCCAGCCCATAGAAGAACCCTACGAAGGAGT

GRC91B|AB863199.1| CATCCTTCAACAAGCAGAAAAATTGGGTCTCCAGCCCATAGAAGAACCCTACGAAGGAGT

GRC92A|AB863200.1| CATCCTTCAACAAGCAGAGAAATTGGGCCTCCAGCCCATAGAAGAACCCTACGAAGGAGT

GRC92C|AB863201.1| CATCCTTCAACAAGCAGAAAAATTGGGTCTCCAGCCCATAGAAGAACCCTACGAAGGAGT

GRC92D|AB863202.1| CATCCTTCAACAAGCAGAGAAATTGGGCCTCCAGCCCATAGAAGAACCCTACGAAGGAGT

IRN1|AB863136.1| CATCCTTCAACAAGCAGAAAAATTGGGCCTCCAGCCCATAGAAGAACCCTACGAAGGAGT

IRN2|AB863137.1| CGTCCTTCAACAAGCAGAAAAATTGGGCCTCCAGCCCATAGAAGAACCCTACGAAGGAGT

IRN3|AB863138.1| CATCCTTCAACAAGCAGAAAAATTGGGCCTCCAGCCCATAGAAGAACCCTACGAAGGAGT

IRN4|AB863139.1| CATCCTTCAACAAGCAGAAAAATTGGGCCTCCAGCCCATAGAAGAACCCTACGAAGGAGT

IRN5|AB863140.1| CATCCTCCAACAAGCAGAAAAATTGGGCCTCCAGCCCATAGAAGAACCCTACGAGGGAGT

IRN6|AB863141.1| CATCCTTCAACAAGCAGAGAAATTGGGCCTCCAGCCCATAGAAGAACCCTACGAAGGAGT

IRN7|AB863142.1| CATCCTTCAACAAGCAGAAAAATTGGGCCTCCAGCCCATAGAAGAACCCTACGAGGGAAT

IRN8|AB863143.1| CATCCTCCAACAAGCAGAAAAATTGGGCCTCCAGCCCATAGAAGAACCCTACGAGGGAGT

IRN9|AB863144.1| CATCCTTCAACAAGCAGAAAAATTGGGCCTCCAGCCCATAGAAGAACCCTACGAAGGAGT

IRN10|AB863145.1| CATCCTTCAACAAGCAGAAAAATTGGGCCTCCAGCCCATAGAAGAACCCTACGAAGGAGT

IRN11|AB863146.1| CGTCCTTCAACAAGCAGAAAAATTGGGCCTCCAGCCCATAGAAGAACCCTACGAAGGAGT

IRN12|AB863147.1| CATCCTTCAACAAGCAGAAAAATTGGGCCTCCAGCCCATAGAAGAACCCTACGAAGGAGT

IRN13|AB863148.1| CATCCTTCAACAAGCAGAAAAATTGGGCCTCCATCCCATAGAAGAACCCTACGAAGGAGT

IRN14|AB863149.1| CATCCTTCAACAAGCAGGAAAATTGGGCCTCCAGCCCATAGAAGAACCCTATGAAGGAGT

IRN15|AB863150.1| CATCCTTCAACAAGCAGAAAAATTGGGTCTCCAGCCCATAGAAGAACCCTACGAAGGAGT

IRN16|AB863151.1| CATCCTTCAACAAGCAGAAAAATTGGGTCTCCAGCCCATAGAAGAACCCTACGAAGGAGT

IRN17|AB863152.1| CATCCTTCAACAAGCAGAAAAATTGGGTCTCCAGCCCATAGAAGAACCCTACGAAGGAGT

IRN18|AB863153.1| CGTCCTTCAACAAGCAGAAAAATTGGGCCTCCAGCCCATAGAAGAACCCTACGAAGGAGT

IRN19|AB863154.1| CGTCCTTCAACAAGCAGAGAAATTGGGCCTCCAGCCCATAGAAGAACCCTACGAAGGAGT

IRN20|AB863155.1| CATCCTTCAACAAGCAGAAAAATTGGGTCTCCAGCCCATAGAAGAACCCTACGAAGGAGT

IRN21|AB863156.1| CATCCTTCAACAAGCAGAAAAATTGGGCCTCCAGCCCATAGAAGAACCCTATGAAGGAGT

JPNHGB340|AB863157.1| CATCCTTCAACAAGCAAAGAATTTGGGTCTCCAGCCCATTGAAGAACCCTATGAAGGAGT

JPNKWB778|AB863158.1| CATCCTTCAACAAGCAGAGAATTTGGGTCTCCAGCCCATTGAAGAACCCTATGAAGGAGT

JPNM|AB863159.1| CATCCTTCAACAAGCAGAGAATTTGGGTCTCCAGCCCATTGAAGAACCCTATGAAGGAGT

JPNN|AB863160.1| CATCCTTCAACAAGCAGAGAAGTTGGGCCTCCAGCCCATAGAAGAACCCTATGAAGGAGT

JPNS1|AB863161.1| CATCCTTCAACAAGCAGAAAAGTTGGGCCTCCAGCCCATTGAAGAACCCTACGAAGGAGT

JPNS2|AB863162.1| CATCCTTCAACAAGCAGAAAAGTTGGGCCTCCAGCCCATTGAAGAACCCTACGAAGGAGT

JPNTKD762|AB863163.1| CATCCTTCAACAAGCAGAGAATTTGGGTCTCCAGCCCATTGAAGAACCCTATGAAGGAGT

JPNUV1|AB863164.1| CATCCTTCAACAAGCAGAGAATTTGGGTCTCCAGCCCATTGAAGAACCCTATGAAGGAGT

JPNUV26|AB863165.1| CATCCTTCAACAAGCAGAGAATTTGGGTCTCCAGCCCATTGAAGAACCCTATGAAGGAGT

TUR1|AB863166.1| CATCCTTCAACAAGCAGAAAAATTGGGTCTCCAGCCCATCGAAGAACCCTATGAAGGAGT

TUR2|AB863167.1| CATCCTTCAACAAGCAGAAAAATTGGGTCTCCAGCCCATCGAAGAACCCTATGAAGGAGT

TUR4|AB863168.1| CATCCTTCAACAAGCAGAAAAATTGGGTCTCCAGCCCATCGAAGAACCCTATGAAGGAGT

TUR5|AB863169.1| CATCCTTCAACAAGCAGAGAAATTGGGCCTCCAGCCCATAGAAGAACCCTATGAAGGAGT

TUR12|AB863170.1| CATCCTTCAACAAGCAGAAAAATTAGGCCTCCAGCCCATAGAAGAACCCTACGAAGGAGT

TUR34|AB863171.1| CATCCTTCAACAAGCAGAGAAATTGGGCCTCCAGCCCATTGAAGAACCCTACGAAGGAGT

TUR50|AB863172.1| CATCCTTCAACAAGCAGAGAAATTGGGCCTCCAGCCCATAGAAGAACCCTACGAAGGAGT

TUR59|AB863173.1| CATCCTTCAACAAGCAGAAAAATTGGGCCTCCAGCCCATAGAAGAACCCTACGAAGGAGT

TUR69|AB863174.1| CATCCTTCAACAAGCAGAAAAATTGGGCCTCCAGCCCATAGAAGAACCCTACGAAGGAGT

TUR81|AB863175.1| CATCCTTCAACAAGCAGAAAAATTGGGCCTCCAGCCCATAGAAGAACCCTACGAAGGAGT

TUR84|AB863176.1| CATCCTTCAACAAGCAGAAAAATTGGGCCTCCAGCCCATAGAAGAACCCTACGAAGGAGT

TUR94|AB863177.1| CATCCTTCAACAAGCAGAGAAATTGGGCCTCCAGCCCATAGAAGAACCCTACGAAGGAGT

TUR213|AB863178.1| CATCCTTCAACAAGCAGAAAAATTGGGTCTCCAGCCCATCGAAGAACCCTATGAAGGAGT

TUR214|AB863179.1| CATCCTTCAACAAGCAGAAAAATTGGGTCTCCAGCCCATCGAAGAACCCTATGAAGGAAT

TUR216|AB863180.1| TATCCTTCAACAAGCAGAGAAATTGGGCCTCCAGCCCATCGAAGAACCCTACGAAGGAGT

TUR220|AB863181.1| CATCCTTCAACAAGCAGAAAAATTGGGCCTCCAGCCCATAGAAGAACCCTACGAAGGAGT

TUR239|AB863182.1| CATCCTTCAACAAGCAGAAAAATTGGGCCTCCAGCCCATAGAAGAACCCTACGAAGGAGT

TUR244|AB863183.1| CATCCTTCAACAAGCAGAAAAATTGGGTCTCCAGCCCATCGAAGAACCCTATGAAGGAGT

TUR249|AB863184.1| CATCCTTCAACAAGCAGAAAAATTGGGTCTCCAGCCCATCGAAGAACCCTATGAAGGAGT

TUR263|AB863185.1| CATCCTTCAACAAGCAGAGAAATTGGGTCTCCAGCCCATAGAAGAACCCTACGAAGGAGT

TUR278|AB863186.1| CATCCTTCAACAAGCAGAGAAATTGGGCCTCCAGCCCATAGAAGAACCCTACGAAGGAGT

TUR279|AB863187.1| CATCCTTCAACAAGCAGAAAAATTGGGCCTCCAGCCCATAGAAGAACCCTACGAAGGAGT

TUR285|AB863188.1| CATCCTTCAACAAGCAGAAAAATTGGGCCTCCAGCCCATAGAAGAACCCTACGAAGGAGT

TUR289|AB863189.1| CATCCTTCAACAAGCAGAAAAATTGGGCCTCCAGCCCATAGAAGAACCCTACGAAGGAGT

TUR303|AB863190.1| CATCCTTCAACAAGCAGAGAAATTGGGCCTCCAGCCCATAGAAGAACCCTACGAAGGAGT

TUR306|AB863191.1| CATCCTTCAACAAGCAGAGAAATTGGGCCTCCAGCCCATAGAAGAACCCTACGAAGGAGT

Cabb-S|NC_001497.1| TCAAGAAGTATTCATTCTAGAATACAAAGAAGAGGAAGAAGAAACCTCTACAGAAGAAAG

CRO180A|AB863192.1| TCAAGAAGTATTCATTCTAGAATACAAAGAAGAGGAAGAAGAAACCTCTACAGAAGAA--

GRC83|AB863193.1| TCAAGAAGTATTCATTCTAGAATACAAAGAAGAGGAAGAAGAAACCTCTACAGAAGAA--

GRC84B|AB863194.1| TCAAGAAGTATTCATTCTAGAATACAAAGAAGAGGAAGAAGAAACCTCTACAGAAGAA--

GRC86B|AB863195.1| TCAAGAAGTATTCATTCTAGAATACAAAGAAGAGGAAGAAGAAACCTCTACAGAAGAA--

GRC86D|AB863196.1| TCAAGAAGTATTCATTCTAGAATACAAAGAAGAGGAAGAAGAAACCTCTACAGAAGAA--

GRC87E|AB863197.1| TCAAGAAATATTCATTCTAGAATACAAAGAAGAGGAAGAAGAAACCTCTACAGAAGAA--

GRC87G|AB863198.1| TCAAGAAGTATTCATTCTAGAATACAAAGAAGAGGAAGAAGAAACCTCTACAGAAGAA--

GRC91B|AB863199.1| TCAAGAAGTATTCATTCTAGAATACAAAGAAGAGGAAGAAGAAACCTCTACCGAAGAA--

GRC92A|AB863200.1| TCAAGAAGTATTCATTCTAGAATACAAAGAAGAGGAAGAAGAAACCTCTACCGAAGAA--

GRC92C|AB863201.1| TCAAGAAGTATTCATTCTAGAATACAAAGAAGAGGAAGAAGAAACCTCTACAGAAGAA--

GRC92D|AB863202.1| TCAAGAAGTATTCATTCTAGAATACAAAGAAGAGGAAGAAGAAACCTCTACCGAAGAA--

IRN1|AB863136.1| TCAAGAAGTATTCATTCTAGAATACAAAGAAGAGGAAGAAGAAACCTCTACCGAAGAA--

IRN2|AB863137.1| TCAAGAAGTATTCATTCTAGAATACAAAGAAGAGGAAGAAGAGACCTCTACCGAAGAA--

IRN3|AB863138.1| TCAAGAAGTATTCATTCTAGAATACAAAGAAGAGGAAGAAGAAACCTCTACCGAAGAA--

IRN4|AB863139.1| TCAAGAAGTATTCATTCTAGAATACAAAGAAGAGGAAGAAGAAACCTCTACCGAAGAA--

IRN5|AB863140.1| TCAAGAAGTATTCATTCTAGAATACAAAGAAGAGGAAGAAGAAACCTCTACCGAAGAA--

IRN6|AB863141.1| TCAAGAAGTATTCATTCTAGAATACAAAAAAGAGGAAGAAGAAACCTCTACCGAAGAA--

IRN7|AB863142.1| TCAAGAAGTATTCATTCTAGAATACAAAGAAGAGGAAGAAGAAACCTCTACCGAAGAA--

IRN8|AB863143.1| TCAAGAAGTATTCATTCTAGAATACAAAGAAGAGGAAGAAGAAACCTCTACCGAAGAA--

IRN9|AB863144.1| TCAAGAAGTATTCATTCTAGAATACAAAGAAGAGGAAGAAGAAACCTCTACCGAAGAA--

IRN10|AB863145.1| TCAAGAAGTATTCATTCTAGAATACAAAGAAGAGGAAGAAGAAACTTCTACCGAAGAA--

IRN11|AB863146.1| TCAAGAAGTATTCATTCTAGAATACAAAGAAGAGGAAGAAGAAACCTCTACCGAAGAA--

IRN12|AB863147.1| TCAAGAAGTATTCATTCTAGAATACAAAGAAGAGGAAGAAGAAACCTCTACCGAAGAA--

IRN13|AB863148.1| TCAAGAAGTATTCATTCTAGAATACAAAGAAGAGGAAGAAGAAACCTCTACCGAAGAA--

IRN14|AB863149.1| TCAAGAAGTATTCATTCTAGAATACAAAGAAGAGGAAGAAGAAACCTCTACCGAAGAA--

IRN15|AB863150.1| TCAAGAAGTATTCATTCTAGAATACAAAGAAGAGGAAGAAGAAACCTCTACCGAAGAA--

IRN16|AB863151.1| TCAAGAAGTATTCATTCTAGAATACAAAGAAGAGGAAGAAGAAACCTCTACCGAAGAA--

IRN17|AB863152.1| TCAAGAAGTATTCATTCTAGAATACAAAGAAGAGGAAGAAGAAACCTCTACCGAAGAA--

IRN18|AB863153.1| TCAAGAAGTATTCATTCTAGAATACAAAGAAGAGGAAGAAGAGACCTCTACCGAAGAA--

IRN19|AB863154.1| TCAAGAAGTATTCATTCTAGAATACAAAGAAGAGGAAGAAGAAACCTCTACCGAAGAA--

IRN20|AB863155.1| TCAAGAAGTATTCATTTTAGAATACAAAGAAGAGGAAGAAGAAACCTCTACCGAAGAA--

IRN21|AB863156.1| TCAAGAAGTATTCATTCTAGAATACAAAGAAGAGGAAGAAGAAACCTCTACCGAAGAA--

JPNHGB340|AB863157.1| TCAAGAAGTATTCATCTTAGAATACAAAGAAGAGGAAGAAGAAACCTCTACAGAAGAAAG

JPNKWB778|AB863158.1| TCAAGAAGTATTCATCCTAGAATACAAAAAAGAGGAAGAAGAAACCTCTACAGAAGAAAG

JPNM|AB863159.1| TCAAGAGGTATTCGTCTTAGAATACAAAGAAGAGGAAGAAGAGACCTCTACAGAAGAAAG

JPNN|AB863160.1| TCAAGAAGTATTCATTTTAGAATACGAAGAAGAGGAAGAAGAAACCTCTACAGAAGAAAG

JPNS1|AB863161.1| TCAAGAAGTATTCATTTTAGAATACAAAGAAGAGGAAGAAGAAACCTCTACAGAAGAAAA

JPNS2|AB863162.1| TCAAGAAGTATTCATTTTAGAATACAAAGAAGAGGAAGAAGAAACCTCTACAGAAGAAAA

JPNTKD762|AB863163.1| TCAAGAGGTATTCATCTTAGAATACAAAGAAGAGGAAGAAGAAACCTCTACAGAAGAAAG

JPNUV1|AB863164.1| TCAAGAGGTATTCGTCTTAGAATACAAAGAAGAGGAAGAAGAGACCTCTACAGAAGAAAG

JPNUV26|AB863165.1| TCAAGAGGTATTCGTCTTAGAATACAAAGAAGAGGAAGAAGAGACCTCTACAGAAGAAAG

TUR1|AB863166.1| TCAAGAAGTATTCATCCTAGAATACAAAGAAGAGGAAGAAGAAACCTCTACAGAAGAA--

TUR2|AB863167.1| TCAAGAAGTATTCATCCTAGAATACAAAGAAGAGGAAGAAGAAACCTCTACAGAAGAA--

TUR4|AB863168.1| TCAAGAAGTATTCATCCTAGAATACAAAGAAGAGGAAGAAGAAACCTCTACAGAAGAA--

TUR5|AB863169.1| TCAAGAAGTATTCATTCTAGAATACAAAGAAGAGGAAGAAGAAACCTCTACCGAAGAA--

TUR12|AB863170.1| TCAAGAAGTATTCATTCTAGAATACAAAGAAGAGGAAGAAGAAACCTCTACCGAAGAA--

TUR34|AB863171.1| TCAAGAAGTATTCATTCTAGAATACAAAGAAGAGGAAGAAGAAACCTCTACCGAAGAA--

TUR50|AB863172.1| TCAAGAAGTATTCATTCTAGAATACAAAGAAGAGGAAGAAGAAACCTCTACCGAAGAA--

TUR59|AB863173.1| TCAAGAAGTATTCATTCTAGAATACAAAGAAGAGGAAGAAGAAACCTCTACCGAAGAA--

TUR69|AB863174.1| TCAAGAAGTATTCATCTTAGAATACAAAGAAGAGGAAGAAGAAACCTCTACCGAAGAA--

TUR81|AB863175.1| TCAAGAAGTATTCATCTTAGAATACAAAGAAGAGGAAGAAGAAACCTCTACCGAAGAA--

TUR84|AB863176.1| TCAAGAAGTATTCATCTTAGAATACAAAGAAGAGGAAGAAGAAACCTCTACCGAAGAA--

TUR94|AB863177.1| TCAAGAAGTATTCATTTTAGAATACAAAGAAGAGGAAGAAGAAACCTCTACCGAAGAA--

TUR213|AB863178.1| TCAAGAAGTATTCATTCTAGAATACAAAGAAGAGGAAGAAGAAACCTCTACCGAAGAA--

TUR214|AB863179.1| TCAAGAAGTATTCATCCTAGAATACAAAGAAGAGGAAGAAGAAACCTCTACCGAAGAA--

TUR216|AB863180.1| TCAAGAAGTATTCATTCTAGAATATAAAGAAGAGGAAGAAGAAACCTCTACCGAAGAA--

TUR220|AB863181.1| TCAAGAAGTATTCATCTTAGAATACAAAGAAGAGGAAGAAGAAACCTCTACCGAAGAA--

TUR239|AB863182.1| TCGAGAAGTATTCATTCTAGAATACAAAGAAGAGGAAGAAGAAACCTCTACCGAAGAA--

TUR244|AB863183.1| TCAAGAAGTATTCATCCTAGAATACAAAGAAGAGGAAGAAGAAACCTCTACAGAAGAA--

TUR249|AB863184.1| TCAAGAAGTATTCATCCTAGAATACAAAGAAGAGGAAGAAGAAACCTCTACAGAAGAA--

TUR263|AB863185.1| TCAAGAAGTATTCATTCTAGAATACAAAGAAGAGGAAGAAGAAACCTCTACCGAAGAA--

TUR278|AB863186.1| TCAAGAAGTATTCATCTTAGAATACAAAGAAGAGGAAGAAGAAACCTCTACCGAAGAA--

TUR279|AB863187.1| TCAAGAAGTATTCATCTTAGAATACAAAGAAGAGGAAGAAGAAACCTCTACCGAAGAA--

TUR285|AB863188.1| TCAAGAAGTATTCATTCTAGAATACAAAGAAGAGGAAGAAGAAACCTCTACCGAAGAA--

TUR289|AB863189.1| TCAAGAAGTATTCATCTTAGAATACAAAGAAGAGGAAGAAGAAACCTCTACCGAAGAA--

TUR303|AB863190.1| TCAAGAAGTATTCATTCTAGAATACAAAGAAGAGGAAGAAGAAACCTCTACCGAAGAA--

TUR306|AB863191.1| TCAAGAAGTATTCATTCTAGAATACAAAGAAGAGGAAGAAGAAACCTCTACCGAAGAA--

Cabb-S|NC_001497.1| TG---ATGGATCATCTACTTCTGAAGACTCA------GACTCAGACTGAGCAGGTGATGA

CRO180A|AB863192.1| -GATGATGGATCATCTACTTCAGAAGACTCAGATTCAGACTCAGACTGAGCAGGTGATGA

GRC83|AB863193.1| -GATGATGGATCATCTACTTCAGAAGACTCAGATTCAGACTCAGACTGAGCAGGTGATGA

GRC84B|AB863194.1| -GATGATGGATCATCTACTTCAGAAGACTCAGATTCAGACTCAGACTGAGCAGGTGATGA

GRC86B|AB863195.1| -GATGATGGATCATCTACTTCAGAAGACTCAGATTCAGACTCAGACTGAGCAGGTGATGA

GRC86D|AB863196.1| -GATGATGGATCATCTACTTCAGAAGACTCAGATTCAGACTCAGACTGAGCAGGTGATGA

GRC87E|AB863197.1| -GATGATGGATCATCTACTTCAGAAGACTCAGATTCAGACTCAGACTGAGCAGGTGATGA

GRC87G|AB863198.1| -GATGATGGATCATCTACTTCAGAAGACTCAGATTCAGACTCAGACTGAGCAGGTGATGA

GRC91B|AB863199.1| -GATGATGGATCATCTACTTCAGAAGACTCAGATTCAGAATCAGACTGAGCAGGTGATGA

GRC92A|AB863200.1| -GATGATGGATCATCTACTTCAGAAGACTCAGATACAGAATCAGACTGAGCAGGTGATGA

GRC92C|AB863201.1| -GATGATGGATCATCTACTTCAGAAGACTCAGATTCAGACTCAGACTGAGCAGGTGATGA

GRC92D|AB863202.1| -GATGATGGATCATCTACTTCAGAAGACTCAGATTCAGACTCAGACTGAGCAGGTGATGA

IRN1|AB863136.1| -GATGATGGATCATCTACTTCAGAAGACTCAGATTCAGAATCAGACTGAGCAGGTGATGA

IRN2|AB863137.1| -GATGATGGATCATCTACTTCAGAAGACTCAGATTCAGAATCAGACTGAGCAGGTGATGA

IRN3|AB863138.1| -GATGATGGATCATCTACTTCAGAAGACTCAGATTCAGAATCAGACTGAGCAGGTGATGA

IRN4|AB863139.1| -GATGATGGATCATCTACTTCAGAAGACTCAGATTCAGAATCAGACTGAGCAGGTGATGA

IRN5|AB863140.1| -GATGATGGATCATCTACTTCAGAAGACTCAGATTCAGAATCAGACTGAGCAGGTGATGA

IRN6|AB863141.1| -GATGATGGATCATCTACTTCAGAAGACTCAGATTCAGAATCAGACTGAGCAGGTGATGA

IRN7|AB863142.1| -GATGATGGATCATCTACTTCAGAAGACTCAGATTCAGAATCAGACTGAGCAGGTGATGA

IRN8|AB863143.1| -GATGATGGATCATCTACTTCAGAAGACTCAGATTCAGAATCAGACTGAGCAGGTGATGA

IRN9|AB863144.1| -GATGATGGATCATCTACTTCAGAAGACTCAGATTCAGAATCAGACTGAGCAGGTGATGA

IRN10|AB863145.1| -GATGATGGATCATCTACTTCAGAAGACTCAGATTCAGAATCAGACTGAGCAGGTGATGA

IRN11|AB863146.1| -GATGATGGATCATCTACTTCAGAAGACTCAGATTCAGAATCAGACTGAGCAGGTGATGA

IRN12|AB863147.1| -GATGATGGATCATCTACTTCAGAAGACTCAGATTCAGAGTCAGACTGAGCAGGTGATGA

IRN13|AB863148.1| -GATGATGGATCATCTACTTCAGAAGACTCAGATTCAGAATCAGACTGAGCAGGTGATGA

IRN14|AB863149.1| -GATGATGGATCATCTACTTCAGAAGACTCAGATTCAGAATCAGACTGAGCAGGTGATGA

IRN15|AB863150.1| -GATGATGGATCATCTACTTCAGAAGACTCAGATTCAGAATCAGACTGAGCAGGTGATGA

IRN16|AB863151.1| -GATGATGGATCATCTACTTCAGAAGACTCAGATTCAGAATCAGACTGAGCAGGTGATGA

IRN17|AB863152.1| -GATGATGGATCATCTACTTCAGAAGACTCAGATTCAGAATCAGACTGAGCAGGTGATGA

IRN18|AB863153.1| -GATGATGGATCATCTACTTCAGAAGACTCAGATTCAGAATCAGACTGAGCAGGTGATGA

IRN19|AB863154.1| -GATGATGGATCATCTACTTCAGAAGACTCAGATTCAGAATCAGACTGAGCAGGTGATGA

IRN20|AB863155.1| -GATGATGGATCATCTACTTCAGAAGACTCAGATTCAGAATCAGACTGAGCAGGTGATGA

IRN21|AB863156.1| -GATGATGGATCATCTACTTCAGAAGACTCAGATTCAGAATCAGACTGAGCAGGTGATGA

JPNHGB340|AB863157.1| CGATGATGGATCATCTACTTCTGAAGACTCA------GACTCAGACTGAGCAGGTGATGA

JPNKWB778|AB863158.1| CGATGATGGATCATCTACTTCTGAAGACTCA------GACTCAGACTGAGCAGGTGATGA

JPNM|AB863159.1| TGACGATGGATCATCTACTTCTGAAGACTCA------GACTCAGACTGAGCAGGTGATGA

JPNN|AB863160.1| TG---ATGGATCATCTACTTCTGAAGACTCA------GACTCAGACTGAGCAGGTGATGA

JPNS1|AB863161.1| TG---ATGAATCATCTACTTCTGAAGACTCA------GACTCAGACTGAGCAGGTGATGA

JPNS2|AB863162.1| TG---ATGAATCATCTACTTCTGAAGACTCA------GACTCAGACTGAGCAGGTGATGA

JPNTKD762|AB863163.1| TGACGATGGATCATCTACTTCTGAAGACTCA------GACTCAGACTGAGCAGGTGATGA

JPNUV1|AB863164.1| TGACGATGGATCATCTACTTCTGAAGACTCA------GACTCAGACTGAGCAGGTGATGA

JPNUV26|AB863165.1| TGACGATGGATCATCTACTTCTGAAGACTCA------GACTCAGACTGAGCAGGTGATGA

TUR1|AB863166.1| -GATGATGGATCATCTACTTCAGAAGACTCAGATTCAGACTCAGACTGAGCAGGTGATGA

TUR2|AB863167.1| -GACGATGGATCATCTACTTCAGAAGACTCAGATTCAGACTCAGACTGAGCAGGTGATGA

TUR4|AB863168.1| -GACGATGGATCATCTACTTCAGAAGACTCAGATTCAGACTCAGACTGAGCAGGTGATGA

TUR5|AB863169.1| -GATGATGGATCATCTACTTCAGAAGACTCAGATTCAGACTCAGACTGAGCAGGTGATGA

TUR12|AB863170.1| -GATGATGGATCATCTACTTCAGAAGACTCAGATTCAGACTCAGACTGAGCAGGTGATGA

TUR34|AB863171.1| -GATGATGGATCATCTACTTCAGAAGACTCAGATTCAGACTCAGACTGAGCAGGTGATGA

TUR50|AB863172.1| -GATGATGGATCATCTACTTCAGAAGACTCAGATTCAGACTCAGACTGAGCAGGTGATGA

TUR59|AB863173.1| -GATGATGGATCATCTACTTCAGAAGACTCAGATTCAGACTCAGACTGAGCAGGTGATGA

TUR69|AB863174.1| -GATGATGGATCATCTACTTCAGAAGACTCAGATTCAGACTCAGACTGAGCAGGTGATGA

TUR81|AB863175.1| -GATGATGGATCATCTACTTCAGAAGACTCAGATTCAGACTCAGACTGAGCAGGTGATGA

TUR84|AB863176.1| -GATGATGGATCATCTACTTCAGAAGACTCAGATTCAGACTCAGACTGAGCAGGTGATGA

TUR94|AB863177.1| -GATGATGGATCATCTACTTCAGAAGACTCAGATTCAGACTCAGACTGAGCAGGTGATGA

TUR213|AB863178.1| -GATGATGGATCATCTACTTCAGAAGACTCAGATTCAGATTCAGACTGAGCAGGTGATGA

TUR214|AB863179.1| -GATGATGGATCATCTACTTCAGAAGACTCAGATTCAGACTCAGACTGAGCAGGTGATGA

TUR216|AB863180.1| -GATGATGGATCATCTACTTCAGAAGACTCA------GACTCAGACTGAGCAGGTGATGA

TUR220|AB863181.1| -GATGATGGATCATCTACTTCAGAAGACTCAGATTCAGACTCAGACTGAGCAGGTGATGA

TUR239|AB863182.1| -GATGATGGATCATCTACTTCAGAAGACTCAGATTCAGACTCAGACTGAGCAGGTGATGA

TUR244|AB863183.1| -GATGATGGATCATCTACTTCAGAAGACTCAGATTCAGACTCAGACTGAGCAGGTGATGA

TUR249|AB863184.1| -GATGATGGATCATCTACTTCAGAAGACTCAGATTCAGACTCAGACTGAGCAGGTGATGA

TUR263|AB863185.1| -GATGATGGATCATCTACTTCAGAAGACTCAGATTCAGACTCAGACTGAGCAGGTGATGA

TUR278|AB863186.1| -GATGATGGATCATCTACTTCAGAAGACTCAGATTCAGACTCAGACTGAGCAGGTGATGA

TUR279|AB863187.1| -GATGATGGATCATCTACTTCAGAAGACTCAGATTCAGACTCAGACTGAGCAGGTGATGA

TUR285|AB863188.1| -GATGATGGATCATCTACTTCAGAAGACTCAGATTCAGACTCAGACTGAGCAGGTGATGA

TUR289|AB863189.1| -GATGATGGATCATCTACTTCAGAAGACTCAGATTCAGACTCAGACTGAGCAGGTGATGA

TUR303|AB863190.1| -GATGATGGATCATCTACTTCAGAAGACTCAGATTCAGACTCAGACTGAGCAGGTGATGA

TUR306|AB863191.1| -GATGATGGATCATCTACTTCAGAAGACTCAGATTCAGACTCAGACTGAGCAGGTGATGA

Cabb-S|NC_001497.1| ACGTCACCAATCCCAATTCGATCTACATCAAGGGAAGACTCTACTTCAAGGGATACAAGA

CRO180A|AB863192.1| ACATCACCAATCCCAATTCGATCTACATCAAGGGAAGACTCTACTTCAAGGGATACAAGA

GRC83|AB863193.1| ACATCACCAATCCCAATTCGATCTACATCAAGGGAAGACTCTACTTCAAGGGATACAAGA

GRC84B|AB863194.1| ACATCACCAATCCCAATTCGATCTACATCAAGGGAAGACTCTACTTCAAGGGATACAAGA

GRC86B|AB863195.1| ACATCACCAATCCCAATTCGATCTACATCAAGGGAAGACTCTACTTCAAGGGATACAAGA

GRC86D|AB863196.1| ACATCACCAATCCCAATTCGATCTACATCAAGGGAAGACTCTACTTCAAGGGATACAAGA

GRC87E|AB863197.1| ACATCACCAATCCCAATTCGATCTACATCAAGGGAAGACTCTACTTCAAGGGATACAAGA

GRC87G|AB863198.1| ACATCACCAATCCCAATTCGATCTACATCAAGGGAAGACTCTACTTCAAAGGATACAAGA

GRC91B|AB863199.1| ACATCACCAATCCCAATTCGATCTACATCAAGGGAAGACTCTACTTCAAAGGATACAAGA

GRC92A|AB863200.1| ACATCACCAATCCCAATTCGATCTACATCAAGGGAAGACTCTACTTCAAAGGATACAAGA

GRC92C|AB863201.1| ACATCACCAATCCCAATTCGATCTACATCAAGGGAAGACTCTACTTCAAGGGATACAAGA

GRC92D|AB863202.1| ACATCACCAATCCCAATTCGATCTACATCAAGGGAAGATTCTACTTCAAAGGATACAAGA

IRN1|AB863136.1| ACATCACCAATCCCAATTCGATCTACATCAAGGGAAGACTCTACTTCAAGGGATACAAGA

IRN2|AB863137.1| ACATCACCAATCCCAATTCGATCTACATCAAGGGAAGACTCTACTTCAAGGGATACAAGA

IRN3|AB863138.1| ACATCACCAATCCCAATTCGATCTACATCAAGGGAAGACTCTACTTCAAGGGATACAAGA

IRN4|AB863139.1| ACATCACCAATCCCAATTCGATCTACATCAAGGGAAGACTCTACTTCAAGGGATACAAGA

IRN5|AB863140.1| ACATCACCAATCCCAATTCGATCTACATCAAGGGAAGACTCTACTTCAAGGGATACAAGA

IRN6|AB863141.1| ACATCACCAATCCCAATTCGATCTACATCAAGGGAAGACTCTACTTCAAGGGATACAAGA

IRN7|AB863142.1| ACATCACCAATCCCAATTCGATCTACATCAAGGGAAGACTCTACTTCAAGGGATACAAGA

IRN8|AB863143.1| ACATCACCAATCCCAATTCGATCTACATCAAGGGAAGACTCTACTTCAAGGGATACAAGA

IRN9|AB863144.1| ACATCACCAATCCCAATTCGATCTACATCAAGGGAAGACTCTACTTCAAGGGATACAAGA

IRN10|AB863145.1| ACATCACCAATCCCAATTCGATCTACATCAAGGGAAGACTCTACTTCAAGGGATACAAGA

IRN11|AB863146.1| ACATCACCAATCCCAATTCGATCTACATCAAGGGAAGACTCTACTTCAAGGGATACAAGA

IRN12|AB863147.1| ACATCACCAATCCCAATTCGATCTACATTAAGGGAAGACTCTACTTCAAGGGATACAAGA

IRN13|AB863148.1| ACATCACCAATCCCAATTCGATCTACATCAAGGGAAGACTCTACTTCAAGGGATACAAGA

IRN14|AB863149.1| ACATCACCAATCCCAATTCGATCTACATCAAGGGAAGACTCTACTTCAAGGGATACAAGA

IRN15|AB863150.1| ACATCACCAATCCCAATTCGATCTACATCAAGGGAAGACTCTATTTCAAGGGATACAAGA

IRN16|AB863151.1| ACATCACCAATCCCAATTCGATCTACATCAAGGGAAGACTCTATTTCAAGGGATACAAGA

IRN17|AB863152.1| ACATCACCAATCCCAATTCGATCTACATCAAAGGAAGACTCTATTTCAAGGGATACAAGA

IRN18|AB863153.1| ACATCACCAATCCCAATTCGATCTACATCAAGGGAAGACTCTACTTCAAGGGATACAAGA

IRN19|AB863154.1| ACATCACCAATCCCAATTCGATCTACATCAAGGGAAGACTCTACTTCAAGGGATACAAGA

IRN20|AB863155.1| ACATCACCAATCCCAATTCGATCTACATCAAGGGAAGACTCTATTTCAAGGGATACAAGA

IRN21|AB863156.1| ACATCACCAATCCCAATTCAATCTACATCAAGGGAAGACTCTACTTCAAGGGATACAAGA

JPNHGB340|AB863157.1| ACATCACCAATCCCAATTCGATTTACATCAAGGGAAGACTCTACTTCAAGGGATACAAGA

JPNKWB778|AB863158.1| ACGTCACCAATCCCAATTCGATTTACATCAAGGGAAGACTCTACTTCAAAGGATACAAGA

JPNM|AB863159.1| ACGTCACCAATCCCAATTCGATTTACATCAAGGGAAGACTCTACTTCAAGGGATACAAGA

JPNN|AB863160.1| ATGTCACCAATCCCAATTCGATCTACATCAAAGGGAGACTCTACTTCAAAGGATACAAGA

JPNS1|AB863161.1| ATGTCACCAATCCCAATTCGATCTACATCAAAGGGAGACTCTACTTCAAGGGATACAAGA

JPNS2|AB863162.1| ATGTCACCAATCCCAATTCGATCTACATCAAAGGGAGACTCTACTTCAAGGGATACAAGA

JPNTKD762|AB863163.1| ACGTCACCAATCCCAATTCGATTTACATCAAGGGAAGACTCTACTTCAAGGGATACAAGA

JPNUV1|AB863164.1| ACGTCACCAATCCCAATTCGATTTACATCAAGGGAAGACTCTACTTCAAGGGATACAAGA

JPNUV26|AB863165.1| ACGTCACCAATCCCAATTCGATTTACATCAAGGGAAGACTCTACTTCAAGGGATACAAGA

TUR1|AB863166.1| ACATCACCAACCCCAATTCGATCTACATCAAGGGAAGACTCTACTTCAAGGGATACAAGA

TUR2|AB863167.1| ACATCACCAATCCCAATTCGATCTACATCAAGGGAAGACTCTACTTTAAGGGATACAAGA

TUR4|AB863168.1| ACATCACCAATCCCAATTCGATCTACATCAAGGGAAGACTCTACTTTAAGGGATACAAGA

TUR5|AB863169.1| ACATCACCAATCCCAATTCGATCTACATCAAGGGAAGACTCTACTTCAAGGGATACAAGA

TUR12|AB863170.1| ACATCACCAATCCCAATTCGATCTACATCAAAGGAAGACTCTACTTCAAGGGATACAAGA

TUR34|AB863171.1| ACATCACCAATCCCAATTCGATCTACATCAAGGGAAGACTCTACTTCAAGGGATACAAGA

TUR50|AB863172.1| ACATCACCAATCCCAATTCGATCTACATCAAGGGAAGACTCTACTTCAAGGGATACAAGA

TUR59|AB863173.1| ACATCACCAATCCCAATTCGATCTACATCAAGGGAAGACTCTACTTCAAGGGATACAAGA

TUR69|AB863174.1| ACATCACCAATCCCAATTCGATCTACATCAAGGGAAGACTCTACTTCAAGGGATACAAGA

TUR81|AB863175.1| ACATCACCAATCCCAATTCGATCTACATCAAGGGAAGACTCTACTTCAAGGGATACAAGA

TUR84|AB863176.1| ACATCACCAATCCCAATTCGATCTACATCAAGGGAAGACTCTACTTCAAGGGATACAAGA

TUR94|AB863177.1| ACATCACCAATCCCAATTCGATCTACATCAAGGGAAGACTCTACTTCAAGGGATACAAGA

TUR213|AB863178.1| ACATCACCAATCCCAATTCGATCTACATCAAGGGAAGACTCTACTTTAAGGGATACAAGA

TUR214|AB863179.1| ACATCACCAATCCCAATTCGATCTACATCAAGGGAAGACTCTACTTTAAGGGATACAAGA

TUR216|AB863180.1| ACGTCACCAATCCCAATTCGATCTACATCAAGGGAAGACTCTATTTCAAAGGATACAAGA

TUR220|AB863181.1| ACATCACCAATCCCAATTCGATCTACATCAAGGGAAGACTCTACTTCAAGGGATACAAGA

TUR239|AB863182.1| ACATCACCAATCCCAATTCGATCTACATCAAGGGAAGACTCTATTTCAAGGGATACAAGA

TUR244|AB863183.1| ACATCACCAATCCCAATTCAATCTACATCAAGGGAAGACTCTACTTTAAGGGATACAAGA

TUR249|AB863184.1| ACATCACCAATCCCAATTCGATCTACATCAAGGGAAGACTCTACTTTAAGGGATACAAGA

TUR263|AB863185.1| ACATCACCAATCCCAATTCGATCTACATCAAGGGAAGACTCTACTTTAAGGGATACAAGA

TUR278|AB863186.1| ACATCACCAATCCCAATTCGATCTACATCAAGGGAAGACTCTACTTCAAGGGATACAAGA

TUR279|AB863187.1| ACATCACCAATCCCAATTCAATCTACATCAAGGGAAGACTCTACTTCAAGGGATACAAGA

TUR285|AB863188.1| ACATCACCAATCCCAATTCGATCTACATCAAGGGAAGACTCTACTTCAAGGGATACAAGA

TUR289|AB863189.1| ACATCACCAATCCCAATTCGATCTACATCAAGGGAAGACTCTACTTCAAGGGATACAAGA

TUR303|AB863190.1| ATATCACCAATCCTAATTCGATCTACATCAAGGGAAGACTCTACTTTAAGGGATACAAGA

TUR306|AB863191.1| ACATCACCAATCCCAATTCGATCTACATCAAGGGAAGACTCTACTTTAAGGGATACAAGA

Cabb-S|NC_001497.1| AGATAGAACTTCACTGTTTCGTAGACACGGGAGCAAGCCTATGCATAGCATCCAAGTTCG

CRO180A|AB863192.1| AGATAGAGCTTCACTGTTTTGTAGACACGGGAGCAAGTTTATGCATAGCATCCAAATTCG

GRC83|AB863193.1| AGATAGAGCTTCATTGTTTTGTAGACACGGGAGCAAGTTTATGCATAGCATCCAAGTTCG

GRC84B|AB863194.1| AGATAGAGCTTCATTGTTTTGTAGACACGGGAGCAAGTTTATGCATAGCATCCAAGTTCG

GRC86B|AB863195.1| AGATAGAGCTTCACTGTTTTGTAGACACGGGAGCAAGTTTATGCATAGCATCCAAGTTCG

GRC86D|AB863196.1| AGATAGAGCTTCATTGTTTTGTAGACACGGGAGCAAGTTTATGCATAGCATCCAAGTTCG

GRC87E|AB863197.1| AGATAGAACTTCACTGTTTTGTAGACACGGGAGCAAGTCTATGTATAGCATCCAAATTCG

GRC87G|AB863198.1| AGATAGAACTTCACTGTTTTGTAGACACGGGAGCAAGTTTATGCATAGCATCCAAGTTCG

GRC91B|AB863199.1| AGATAGAGCTTCATTGTTTTGTAGACACGGGAGCAAGTTTATGCATAGCATCCAAATTCG

GRC92A|AB863200.1| AGATAGAGCTTCATTGTTTTGTAGACACGGGAGCAAGTTTATGCATAGCATCCAAATTCG

GRC92C|AB863201.1| AGATAGAGCTTCATTGTTTTGTAGACACGGGAGCAAGTTTATGCATAGCATCCAAGTTCG

GRC92D|AB863202.1| AGATAGAACTTCATTGTTTTGTAGACACGGGAGCAAGTTTATGCATAGCATCCAAGTTCG

IRN1|AB863136.1| AAATAGAACTTCACTGCTTTGTAGACACGGGAGCAAGTTTATGCATAGCATCCAAATTCG

IRN2|AB863137.1| AGATAGAGCTTCACTGCTTTGTAGACACGGGAGCAAGTTTATGCATAGCATCCAAATTCG

IRN3|AB863138.1| AAATAGAACTTCACTGCTTTGTAGACACGGGAGCAAGTTTATGCATAGCATCCAAATTCG

IRN4|AB863139.1| AGATAGAACTTCACTGCTTTGTAGACACGGGAGCAAGTTTATGCATAGCATCCAAATTCG

IRN5|AB863140.1| AGATAGAACTTCACTGCTTTGTAGACACGGGAGCAAGTTTATGCATAGCATCCAAATTCG

IRN6|AB863141.1| AGATAGAACTTCACTGCTTTGTAGACACGGGAGCAAGTTTATGCATAGCATCCAAATTCG

IRN7|AB863142.1| AGATAGAACTTCACTGCTTTGTAGACACGGGAGCAAGTTTATGCATAGCATCCAAATTCG

IRN8|AB863143.1| AGATAGAACTTCACTGCTTTGTAGACACGGGAGCAAGTTTATGCATAGCATCCAAATTCG

IRN9|AB863144.1| AGATAGAACTTCACTGCTTTGTAGACACGGGAGCAAGTTTATGCATAGCATCCAAATTCG

IRN10|AB863145.1| AGATAGAGCTTCACTGCTTTGTAGACACGGGAGCAAGTTTATGCATAGCATCCAAATTCG

IRN11|AB863146.1| AGATAGAGCTTCACTGCTTTGTAGACACGGGAGCAAGTTTATGCATAGCATCCAAATTCG

IRN12|AB863147.1| AGATAGAACTTCACTGCTTTGTAGACACGGGAGCAAGTTTATGCATAGCATCCAAATTCG

IRN13|AB863148.1| AGATAGAACTTCACTGCTTTGTAGACACGGGAGCAAGTTTATGCATAGCATCCAAATTCG

IRN14|AB863149.1| AGATAGAACTTCACTGCTTTGTAGACACGGGAGCAAGTTTATGCATAGCATCCAAATTCG

IRN15|AB863150.1| AGATAGAGCTTCACTGCTTTGTAGACACGGGAGCAAGTTTATGCATAGCATCCAAATTCG

IRN16|AB863151.1| AGATAGAGCTTCACTGCTTTGTAGACACGGGAGCAAGTTTATGCATAGCATCCAAATTCG

IRN17|AB863152.1| AGATAGAGCTTCACTGCTTTGTAGACACGGGAGCAAGTTTATGCATAGCATCCAAATTCG

IRN18|AB863153.1| AGATAGAGCTTCACTGCTTTGTAGACACGGGAGCAAGTTTATGCATAGCATCCAAATTCG

IRN19|AB863154.1| AGATAGAGCTTCACTGCTTTGTAGACACGGGAGCAAGTTTATGCATAGCATCCAAATTCG

IRN20|AB863155.1| AGATAGAGCTTCACTGCTTTGTAGACACGGGAGCAAGTTTATGCATAGCATCCAAATTCG

IRN21|AB863156.1| AGATAGAACTTCACTGTTTTGTAGACACGGGAGCAAGTTTATGCATAGCATCCAAATTCG

JPNHGB340|AB863157.1| AGATAGAGCTTCACTGTTTTGTAGACACGGGAGCAAGCTTATGCATAGCATCCAAGTTCG

JPNKWB778|AB863158.1| AGATAGAGCTTCACTGTTTTGTAGACACGGGAGCAAGCTTATGCATAGCATCCAAGTTCG

JPNM|AB863159.1| AGATAGAGCTTCACTGTTTTGTAGACACGGGAGCAAGCTTATGCATAGCATCCAAGTTCG

JPNN|AB863160.1| AGATAGAGCTTCACTGTTTTGTAGACACGGGAGCTAGCTTATGCATAGCATCCAAGTTCG

JPNS1|AB863161.1| AGATAGAGCTTCACTGTTTTGTAGACACGGGAGCTAGCTTATGCATAGCATCCAAGTTCG

JPNS2|AB863162.1| AGATAGAGCTTCACTGTTTTGTAGACACGGGAGCTAGCTTATGCATAGCATCCAAGTTCG

JPNTKD762|AB863163.1| AGATAGAGCTTCACTGTTTTGTAGACACGGGAGCAAGCTTATGCATAGCATCCAAGTTCG

JPNUV1|AB863164.1| AGATAGAGCTTCACTGTTTTGTAGACACGGGAGCAAGCTTATGCATAGCATCCAAGTTCG

JPNUV26|AB863165.1| AGATAGAGCTTCACTGTTTTGTAGACACGGGAGCAAGCTTATGCATAGCATCCAAGTTCG

TUR1|AB863166.1| AGATAGAACTTCATTGTTTTGTAGACACGGGAGCAAGTTTATGCATAGCATCCAAATTCG

TUR2|AB863167.1| AGATAGAGCTTCACTGCTTTGTAGACACGGGAGCAAGTTTATGCATAGCATCCAAATTCG

TUR4|AB863168.1| AGATAGAGCTTCACTGCTTTGTAGACACGGGAGCAAGTTTATGCATAGCATCCAAATTCG

TUR5|AB863169.1| AGATAGAACTTCACTGTTTCGTAGACACGGGAGCAAGTTTATGCATAGCATCCAAATTCG

TUR12|AB863170.1| AGATAGAGCTTCACTGCTTTGTAGACACGGGAGCAAGTTTATGCATAGCATCCAAATTCG

TUR34|AB863171.1| AGATAGAGCTTCACTGCTTTGTAGACACGGGAGCAAGTTTATGCATAGCATCCAAATTCG

TUR50|AB863172.1| AGATAGAGCTTCACTGCTTTGTAGACACGGGAGCAAGTTTATGCATAGCATCCAAATTCG

TUR59|AB863173.1| AGATAGAACTTCATTGTTTCGTAGACACGGGAGCAAGTTTATGCATAGCATCCAAATTCG

TUR69|AB863174.1| AGATAGAGCTTCATTGTTTCGTAGACACGGGAGCAAGCTTATGCATAGCATCCAAATTCG

TUR81|AB863175.1| AGATAGAGCTTCATTGTTTCGTAGACACGGGAGCAAGCTTATGCATAGCATCCAAATTCG

TUR84|AB863176.1| AGATAGAGCTTCATTGTTTCGTAGACACGGGAGCAAGCTTATGCATAGCATCCAAATTCG

TUR94|AB863177.1| AGATAGAACTTCATTGTTTTGTAGACACGGGAGCAAGTTTATGCATAGCATCCAAATTCG

TUR213|AB863178.1| AGATAGAGCTTCACTGCTTCGTAGACACGGGAGCAAGTTTATGCATAGCATCCAAATTCG

TUR214|AB863179.1| AGATAGAGCTTCACTGCTTTGTAGACACGGGAGCAAGTTTATGCATAGCATCCAAATTCG

TUR216|AB863180.1| AGATAGAGCTTCATTGCTTTGTAGACACGGGAGCAAGTTTATGCATAGCGTCCAAATTCG

TUR220|AB863181.1| AGATAGAGCTTCATTGTTTCGTAGACACGGGAGCAAGCTTATGCATAGCATCCAAATTCG

TUR239|AB863182.1| AGATAGAGCTTCACTGCTTTGTAGACACGGGAGCAAGTTTATGCATAGCATCCAAGTTCG

TUR244|AB863183.1| AGATAGAGCTTCACTGCTTTGTAGACACGGGAGCAAGTTTATGCATAGCATCCAAATTCG

TUR249|AB863184.1| AGATAGAGCTTCACTGCTTTGTAGACACGGGAGCAAGTTTATGCATAGCATCCAAATTCG

TUR263|AB863185.1| AGATAGAGCTTCACTGTTTTGTAGACACGGGAGCAAGTTTATGCATAGCATCCAAATTCG

TUR278|AB863186.1| AGATAGAGCTTCATTGTTTCGTAGACACGGGAGCAAGCTTATGCATAGCATCCAAATTCG

TUR279|AB863187.1| AGATAGAGCTTCATTGTTTCGTAGACACGGGAGCAAGCTTATGCATAGCATCCAAATTCG

TUR285|AB863188.1| AGATAGAGCTTCACTGTTTTGTAGACACGGGAGCAAGTTTATGCATAGCATCCAAATTCG

TUR289|AB863189.1| AGATAGAGCTTCATTGTTTCGTAGACACGGGAGCAAGCTTATGCATAGCATCCAAATTCG

TUR303|AB863190.1| AGATAGAGCTTCACTGCTTTGTAGACACGGGAGCAAGTTTATGCATAGCATCCAAATTCG

TUR306|AB863191.1| AGATAGAGCTTCACTGCTTTGTAGACACGGGAGCAAGTTTATGCATAGCATCCAAATTCG

Cabb-S|NC_001497.1| TCATACCAGAAGAACATTGGGTCAATGCAGAAAGACCAATTATGGTCAAAATAGCAGATG

CRO180A|AB863192.1| TCATACCAGAAGAACATTGGATCAATGCAGAAAGACCAATCATGGTCAAAATTGCAGATG

GRC83|AB863193.1| TCATACCAGAAGAACATTGGATCAATGCAGAAAGACCAATCATGGTCAAAATTGCAGATG

GRC84B|AB863194.1| TCATACCAGAAGAACATTGGATCAATGCAGAAAGACCAATCATGGTCAAAATTGCAGATG

GRC86B|AB863195.1| TTATACCAGAAGAACATTGGATCAATGCAGAAAGACCAATCATGGTCAAAATTGCAGATG

GRC86D|AB863196.1| TCATACCAGAAGAACATTGGATCAATGCAGAAAGACCAATCATGGTCAAAATTGCAGATG

GRC87E|AB863197.1| TCATACCAGAAGAACATTGGATCAATGCAGAAAGACCAATCATGGTCAAAATAGCAGATG

GRC87G|AB863198.1| TCATACCAGAAGAACATTGGATCAATGCAGAAAGACCAATTATGGTCAAAATTGCAGATG

GRC91B|AB863199.1| TCATACCAGAAGAACATTGGATCAATGCAGAAAGACCAATCATGGTCAAAATTGCAGATG

GRC92A|AB863200.1| TCATACCAGAAGAACATTGGATCAATGCAGAAAGACCAATCATGGTCAAAATTGCAGATG

GRC92C|AB863201.1| TCATACCAGAAGAACATTGGATCAATGCAGAAAGACCAATCATGGTCAAAATTGCAGATG

GRC92D|AB863202.1| TCATACCAGAAGAACATTGGATCAATGCAGAAAGACCAATCATGGTCAAAATTGCAGATG

IRN1|AB863136.1| TCATACCAGAAGAACATTGGATCAATGCAGAAAGACCAATCATGGTCAAAATTGCAGATG

IRN2|AB863137.1| TCATACCAGAAGAACATTGGATCAATGCAGAAAGACCAATCATGGTCAAAATTGCAGATG

IRN3|AB863138.1| TCATACCAGAAGAACATTGGATCAATGCAGAAAGACCAATCATGGTCAAAATTGCAGATG

IRN4|AB863139.1| TCATACCAGAAGAACATTGGATTAATGCAGAAAGACCAATCATGGTCAAAATTGCAGATG

IRN5|AB863140.1| TCATACCAGAAGAACATTGGATCAATGCAGAAAGACCAATCATGGTCAAAATTGCAGATG

IRN6|AB863141.1| TCATACCAGAAGAACATTGGATCAATGCAGAAAGACCAATCATGGTCAAAATTGCAGATG

IRN7|AB863142.1| TCATACCAGAAGAACATTGGATCAATGCAGAAAGACCAATCATGGTCAAAATTGCAGATG

IRN8|AB863143.1| TCATACCAGAAGAACATTGGATCAATGCAGAAAGACCAATCATGGTCAAAATTGCAGATG

IRN9|AB863144.1| TCATACCAGAAGAACATTGGATCAATGCAGAAAGACCAATCATGGTCAAAATTGCAGATG

IRN10|AB863145.1| TCATACCAGAAGAACATTGGATCAATGCAGAAAGACCAATCATGGTCAAAATTGCAGATG

IRN11|AB863146.1| TCATACCAGAAGAACATTGGATCAATGCAGAAAGACCAATCATGGTCAAAATTGCAGATG

IRN12|AB863147.1| TCATACCAGAAGAACATTGGATCAATGCAGAAAGACCAATCATGGTCAAAATTGCAGATG

IRN13|AB863148.1| TCATACCAGAAGAACATTGGATCAATGCAGAAAGACCAATTATGGTCAAAATTGCAGATG

IRN14|AB863149.1| TCATACCAGAAGAACATTGGATCAATGCAGAAAGACCAATCATGGTCAAAATTGCAGATG

IRN15|AB863150.1| TCATACCAGAAGAACATTGGATCAATGCAGAAAGACCAATCATGGTCAAAATTGCAGATG

IRN16|AB863151.1| TCATACCAGAAGAACATTGGATCAATGCAGAAAGACCAATCATGGTCAAAATTGCAGATG

IRN17|AB863152.1| TCATACCAGAAGAACATTGGATCAATGCAGAAAGACCAATCATGGTCAAAATTGCAGATG

IRN18|AB863153.1| TCATACCAGAAGAACATTGGATCAATGCAGAAAGACCAATCATGGTCAAAATTGCAGATG

IRN19|AB863154.1| TCATACCAGAAGAACATTGGATCAATGCAGAAAGACCAATCATGGTCAAAATTGCAGATG

IRN20|AB863155.1| TCATACCAGAAGAACATTGGATCAATGCAGAAAGACCAATCATGGTCAAAATTGCAGATG

IRN21|AB863156.1| TCATACCAGAAGAACATTGGATCAATGCAGAAAGACCAATCATGGTCAAAATTGCAGATG

JPNHGB340|AB863157.1| TCATTCCAGAAGAACATTGGGTCAATGCAGAAAGACCAATAATGGTCAAAATAGCAGATG

JPNKWB778|AB863158.1| TCATTCCAGAAGAACATTGGGTCAATGCAGAAAGACCAATAATGGTCAAAATAGCAGATG

JPNM|AB863159.1| TCATTCCAGAAGAACATTGGGTCAATGCAGAAAGACCAATAATGGTCAAAATAGCAGATG

JPNN|AB863160.1| TCATTCCAGAAGAACATTGGGTCAATGCAGAAAGACCAATAATGGTCAAAATAGCAGATG

JPNS1|AB863161.1| TCATTCCAGAAGAACATTGGGTCAATGCAGAAAGACCAATACTGGTCAAAATAGCAGATG

JPNS2|AB863162.1| TCATTCCAGAAGAACATTGGGTCAATGCAGAAAGACCAATACTGGTCAAAATAGCAGATG

JPNTKD762|AB863163.1| TCATTCCAGAAGAACATTGGGTCAATGCAGAAAGACCAATAATGGTCAAAATAGCAGATG

JPNUV1|AB863164.1| TCATTCCAGAAGAACATTGGGTCAATGCAGAAAGACCAATAATGGTCAAAATAGCAGATG

JPNUV26|AB863165.1| TCATTCCAGAAGAACATTGGGTCAATGCAGAAAGACCGATAATGGTCAAAATAGCAGATG

TUR1|AB863166.1| TCATACCAGAAGAACATTGGATCAATGCAGAAAGACCAATCATGGTCAAAATAGCAGATG

TUR2|AB863167.1| TCATACCAGAAGAACATTGGATCAATGCAGAAAGACCAATCATGGTCAAAATAGCAGATG

TUR4|AB863168.1| TCATACCAGAAGAACATTGGATCAATGCAGAAAGACCAATCATGGTCAAAATAGCAGATG

TUR5|AB863169.1| TCATACCAGAAGAACATTGGATCAATGCAGAAAGACCAATCATGGTCAAAATTGCAGATG

TUR12|AB863170.1| TCATACCAGAAGAACATTGGATCAATGCAGAAAGACCAATCATGGTCAAAATAGCAGATG

TUR34|AB863171.1| TCATACCAGAAGAACATTGGATCAATGCAGAAAGACCAATCATGGTCAAAATAGCAGATG

TUR50|AB863172.1| TCATACCAGAAGAACATTGGATCAATGCAGAAAGACCAATCATGGTCAAAATAGCAGATG

TUR59|AB863173.1| TCATACCAGAAGAACATTGGATCAATGCAGAAAGACCAATCATGGTCAAAATTGCAGATG

TUR69|AB863174.1| TCATACCAGAAGAACATTGGATCAATGCAGAAAGACCAATCATGGTCAAAATAGCAGATG

TUR81|AB863175.1| TCATACCAGAAGAACATTGGATCAATGCAGAAAGACCAATCATGGTCAAAATAGCAGATG

TUR84|AB863176.1| TCATACCAGAAGAACATTGGATCAATGCAGAAAGACCAATCATGGTCAAAATAGCAGATG

TUR94|AB863177.1| TCATACCAGAAGAACATTGGATCAATGCAGAAAGACCAATCATGGTCAAAATTGCAGATG

TUR213|AB863178.1| TCATACCAGAAGAACATTGGATCAATGCAGAAAGACCAATCATGGTCAAAATAGCAGATG

TUR214|AB863179.1| TCATACCAGAAGAACATTGGATCAATGCAGAAAGACCAATCATGGTCAAAATAGCAGATG

TUR216|AB863180.1| TCATTCCTGAAGAACATTGGATCAATGCAGAAAGACCAATAATGGTTAAAATTGCAGATG

TUR220|AB863181.1| TCATACCAGAAGAACATTGGATCAATGCAGAAAGACCAATCATGGTCAAAATAGCAGATG

TUR239|AB863182.1| TCATACCAGAAGAACATTGGATCAATGCAGAAAGACCAATCATGGTCAAAATTGCAGATG

TUR244|AB863183.1| TCATACCAGAAGAACATTGGATCAATGCAGAAAGACCAATCATGGTCAAAATAGCAGATG

TUR249|AB863184.1| TCATACCAGAAGAACATTGGATCAATGCAGAAAGACCAATCATGGTCAAAATAGCAGATG

TUR263|AB863185.1| TCATACCAGAAGAACATTGGATCAATGCAGAAAGACCAATCATGGTCAAAATTGCAGATG

TUR278|AB863186.1| TCATACCAGAAGAACATTGGATCAATGCAGAAAGACCAATCATGGTCAAAATAGCAGATG

TUR279|AB863187.1| TCATACCAGAAGAACATTGGATCAATGCAGAAAGACCAATCATGGTCAAAATAGCAGATG

TUR285|AB863188.1| TCATACCAGAAGAACATTGGGTCAATGCAGAAAGATCAATCATGGTCAAAATAGCAGATG

TUR289|AB863189.1| TCATACCAGAAGAACATTGGATCAATGCAGAAAGACCAATCATGGTCAAAATAGCAGATG

TUR303|AB863190.1| TCATACCAGAAGAACATTGGATCAATGCAGAAAGACCAATCATGGTCAAAATAGCAGATG

TUR306|AB863191.1| TCATACCAGAAGAACATTGGATCAATGCAGAAAGACCAATCATGGTCAAAATAGCAGATG

Cabb-S|NC_001497.1| GAAGCTCAATCACCATCAGCAAAGTCTGCAAAGACATAGACTTGATCATAGCCGGCGAGA

CRO180A|AB863192.1| GAAGTTCGATCACCATCAACAAAGTCTGCAGAGACATTGACCTGATCATAGCCGGAGAGA

GRC83|AB863193.1| GAAGTTCGATCACCATCAACAAAGTCTGCAGAGACATTAACCTGATCATAGCCGGAGAAA

GRC84B|AB863194.1| GAAGTTCGATCACCATCAACAAAGTCTGCAGAGACATTGACCTGATCATAGCCGGAGAAA

GRC86B|AB863195.1| GAAGTTCGATCACCATCAACAAAGTCTGCAGAGACATTGACCTGATCATAGCCGGAGAAA

GRC86D|AB863196.1| GAAGTTCGATCACCATCAACAAAGTCTGCAGAGACATTGACCTGATCATAGCCGGAGAAA

GRC87E|AB863197.1| GAAGTTCGATCACCATCAACAAAGTCTGCAGAGACATTGACCTGATCATAGCCGGAGAAA

GRC87G|AB863198.1| GAAGTTCGATTACCATCAACAAAGTCTGCAGAGACATTGACCTGATCATAGCCGGAGAAA

GRC91B|AB863199.1| GAAGTTCGATTACCATCAACAAAGTCTGCAGAGACATTGACCTGATCATAGCCGGAGAAA

GRC92A|AB863200.1| GAAGTTCGATCACCATCAACAAAGTCTGCAGAGACATTGACCTGATCATAGCCGGAGAAA

GRC92C|AB863201.1| GAAGTTCGATCACCATCAACAAAGTCTGCAGAGACATTGACCTGATCATAGCCGGAGAAA

GRC92D|AB863202.1| GAAGTTCGATCACCATCAACAAAGTCTGCAGAGACATTGACCTGATCATAGCCGGAGAAA

IRN1|AB863136.1| GAAGCTCGATTACCATCAACAAAGTCTGCAGAGACATTGACCTGATCATAGCCGGAGAAA

IRN2|AB863137.1| GAAGCTCGATTACCATCAACAAAGTCTGCAGAGACATTGACCTGATCATAGCCGGAGAAA

IRN3|AB863138.1| GAAGCTCGATTACCATCAACAAAGTCTGCAGAGACATTGACCTGATCATAGCCGGAGAAA

IRN4|AB863139.1| GAAGCTCGATTACCATCAACAAAGTCTGCAGAGACATTGACCTGATCATAGCCGGAGAAA

IRN5|AB863140.1| GAAGTTCGATTACCATCAACAAAGTCTGCAGAGACATTGACCTGATCATAGCCGGAGAAA

IRN6|AB863141.1| GAAGCTCGATTACCATCAACAAAGTCTGCAGAGACATTGACCTGATCATAGCCGGAGAAA

IRN7|AB863142.1| GAAGCTCGATTACCATCAACAAAGTCTGCAGAGACATTGACCTGATCATAGCCGGAGAAA

IRN8|AB863143.1| GAAGTTCGATTACCATCAACAAAGTCTGCAGAGACATTGACCTGATCATAGCCGGAGAAA

IRN9|AB863144.1| GAAGCTCGATCACCATCAACAAAGTCTGCAGAGACATTGACCTGATCATAGCCGGAGAAA

IRN10|AB863145.1| GAAGCTCGATTACCATCAACAAAGTCTGCAGAGACATTGACCTGATCATAGCCGGAGAAA

IRN11|AB863146.1| GAAGCTCGATCACCATCAACAAAGTCTGCAGAGACATCGACCTGATCATAGCCGGAGAAA

IRN12|AB863147.1| GAAGCTCGATTACCATCAACAAAGTCTGCAGAGACATTGACCTGATCATAGCCGGAGAAA

IRN13|AB863148.1| GAAGTTCGATCACCATCAACAAAGTCTGCAGAGACATTGACCTGATCATAGCCGGAGAAA

IRN14|AB863149.1| GAAGCTCGATTACCATCAACAAAGTCTGCAGAGACATTGACCTGATCATAGCCGGAGAAA

IRN15|AB863150.1| GAAGCTCGATCACCATCAACAAAGTCTGCAGAGACATTGACCTGATCATAGCCGGAGAAA

IRN16|AB863151.1| GAAGCTCGATCACCATCAACAAAGTCTGCAGAGACATTGACCTGATCATAGCCGGAGAAA

IRN17|AB863152.1| GAAGCTCGATCACCATCAACAAAGTCTGCAGAGACATTGACCTGATCATAGCCGGAGAAA

IRN18|AB863153.1| GAAGCTCGATCACCATCAACAAAGTCTGCAGAGACATTGACCTGATCATAGCCGGAGAAA

IRN19|AB863154.1| GAAGCTCGATTACCATCAACAAAGTCTGCAGAGACATTGACCTAATCATAGCCGGAGAAA

IRN20|AB863155.1| GAAGCTCGATCACCATCAACAAAGTCTGCAGAGACATTGACCTGATCATAGCCGGAGAAA

IRN21|AB863156.1| GAAGCTCGATCACCATCAACAAAGTCTGCAGAGACATTGACCTAATCATAGCCGGAGAAA

JPNHGB340|AB863157.1| GAAGCTCAATCACCATCAGCAAAGTCTGCAAAGACATAGACTTGATCATAGCCGGCGAGA

JPNKWB778|AB863158.1| GAAGTTCAATCACCATCAGCAAAGTCTGCAAAGACATAGACTTGATTATAGCCGGCGAGA

JPNM|AB863159.1| GAAGTTCAATCACCATCAGCAAAGTCTGCAAAGACATAGACTTGATCATAGCCGGCGAGA

JPNN|AB863160.1| GAAGTTCAATCACCATCAGCAAAGTCTGCAAAGACATAGACTTGATCATAGCCGGCGAAA

JPNS1|AB863161.1| GAAGCTCGATCACCATCAGCAAAGTCTGCAAAGATATAGACTTGATCATAGCCGGCGAGA

JPNS2|AB863162.1| GAAGCTCGATCACCATCAGCAAAGTCTGCAAAGATATAGACTTGATCATAGCCGGCGAGA

JPNTKD762|AB863163.1| GAAGTTCAATCACCATCAGCAAAGTCTGCAAAGACATAGACTTGATCATAGCCGGCGAGA

JPNUV1|AB863164.1| GAAGTTCAATCACCATCAGCAAAGTCTGCAAAGACATAGACTTGATCATAGCCGGCGAGA

JPNUV26|AB863165.1| GAAGTTCAATCACCATCAGCAAAGTCTGCAAAGACATAGACTTGATCATAGCCGGCGAGA

TUR1|AB863166.1| GAAGTTCGATCACCATCAACAAAGTCTGCAGAGACATTGACCTGATCATAGCCGGAGAAA

TUR2|AB863167.1| GAAGTTCGATCACCATCAACAAAGTCTGCAGAGACATTGACCTAATCATAGCCGGAGAAA

TUR4|AB863168.1| GAAGTTCGATCACCATCAACAAAGTCTGCAGAGACATTGACCTAATCATAGCCGGAGAAA

TUR5|AB863169.1| GAAGTTCGATTACCATCAACAAAGTCTGCAGAGACATTGACCTGATCATAGCCGGAGAAA

TUR12|AB863170.1| GAAGTTCGATCACCATCAACAAAGTCTGCAAAGACATTGACCTGATCATAGCCGGAGAAA

TUR34|AB863171.1| GAAGTTCGATCACCATCAACAAAGTCTGCAGAGACATTGACCTGATCATAGCCGGAGAAA

TUR50|AB863172.1| GAAGTTCGATCACCATCAACAAAGTCTGCAGAGACATTGACCTGATCATAGCCGGAGAAA

TUR59|AB863173.1| GAAGTTCGATCACCATCAACAAAGTCTGCAGAGACATTGACCTGATCATAGCCGGAGAAA

TUR69|AB863174.1| GAAGTTCGATCACCATCAACAAAGTCTGCAGAGACATTGACCTGATCATAGCCGGAGAAA

TUR81|AB863175.1| GAAGTTCGATCACCATCAACAAAGTCTGCAGAGACATTGACCTGATCATAGCCGGAGAAA

TUR84|AB863176.1| GAAGTTCGATCACCATCAACAAAGTCTGCAGAGACATTGACCTGATCATAGCCGGAGAAA

TUR94|AB863177.1| GAAGTTCGATCACCATCAACAAAGTCTGCAGAAACATTGACCTGATCATAGCCGGAGAAA

TUR213|AB863178.1| GAAGTTCGATCACCATCAACAAAGTCTGCAGAGACATTGACCTGATCATAGCCGGAGAAA

TUR214|AB863179.1| GAAGTTCGATCACCATCAACAAAGTCTGTAGAGACATCGACCTGATCATAGCCGGAGAAA

TUR216|AB863180.1| GAAGTTCGATTACCATCAACAAAGTCTGCAGAGACATTGACTTGATCATAGCCGGCGAAA

TUR220|AB863181.1| GAAGTTCGATCACCATCAACAAAGTCTGCAGAGACATTGACCTGATCATAGCCGGAGAAA

TUR239|AB863182.1| GAAGTTCGATCACCATCAACAAAGTCTGCAGAGACATTGACCTGATCATAGCCGGAGAAA

TUR244|AB863183.1| GAAGTTCGATCACCATCAACAAAGTCTGCAAAGACATTGACCTGATCATAGCCGGAGAAA

TUR249|AB863184.1| GAAGTTCGATCACCATCAACAAAGTCTGCAAAGACATTGACCTGATCATAGCCGGAGAAA

TUR263|AB863185.1| GAAGTTCGATTACCATCAACAAAGTCTGCAGAGACATTGACCTGATCATAGCCGGAGAAA

TUR278|AB863186.1| GAAGTTCGATCACCATCAACAAAGTCTGCAGAGACATTGACCTGATCATAGCCGGAGAAA

TUR279|AB863187.1| GAAGTTCGATCACCATCAACAAAGTCTGCAGAGACATTGACCTGATCATAGCCGGAGAAA

TUR285|AB863188.1| GAAGTTCGATCACCATCAACAAAGTCTGCAGAGACATTGATCTGATCATAGCCGGAGAAA

TUR289|AB863189.1| GAAGTTCGATCACCATCAACAAAGTCTGCAGAGACATTGACCTGATCATAGCCGGAGAAA

TUR303|AB863190.1| GAAGTTCGATCACCATCAACAAAGTCTGCAGAGACATTGACCTGATCATAGCCGGAGAGA

TUR306|AB863191.1| GAAGTTCGATCACCATCAACAAGGTCTGCAAAGACATTGACCTGATCATAGCCGGAGAAA

Cabb-S|NC_001497.1| TATTCAGAATTCCCACCGTCTATCAGCAAGAAAGTGGCATCGATTTCATTATCGGCAACA

CRO180A|AB863192.1| TATTCCATATTCCCACCGTCTATCAACAGGAAAGTGGAATCGATTTCATCATCGGCAACA

GRC83|AB863193.1| TATTCCATATTCCTACCGTCTATCAGCAGGAAAGTGGAATCGATTTCATCATCGGCAACA

GRC84B|AB863194.1| TATTTCATATTCCTACCGTCTATCAGCAGGAAAGTGGAATCGATTTCATCATCGGCAACA

GRC86B|AB863195.1| TATTTCATATTCCTACCGTCTATCAGCAGGAAAGTGGAATCGATTTCATCATCGGCAACA

GRC86D|AB863196.1| TATTCCATATTCCTACCGTCTATCAGCAGGAAAGTGGAATCGATTTCATCATCGGCAACA

GRC87E|AB863197.1| TATTTCATATTCCTACCGTCTATCAACAGGAAAGTGGAATCGATTTCATCATCGGCAACA

GRC87G|AB863198.1| TATTTCATATTCCTACCGTCTATCAGCAGGAAAGTGGAATCGATTTCATCATCGGCAACA

GRC91B|AB863199.1| TATTTCATATTCCTACCGTCTATCAGCAGGAAAGTGGAATCGATTTCATCATCGGCAACA

GRC92A|AB863200.1| TATTTCATATTCCTACCGTCTATCAGCAGGAAAGTGGAATCGATTTCATCATCGGCAACA

GRC92C|AB863201.1| TATTTCATATTCCTACCGTCTATCAGCAGGAAAGTGGAATCGATTTCATCATCGGCAACA

GRC92D|AB863202.1| TATTCCATATTCCTACCGTCTATCAACAGGAAAGTGGAATCGATTTCATCATCGGCAACA

IRN1|AB863136.1| TATTCCATATTCCTACCGTCTATCAACAGGAAAGTGGAATCGATTTCATCATCGGCAACA

IRN2|AB863137.1| TATTCCATATTCCTACCGTCTATCAACAGGAAAGTGGAATCGATTTCATCATCGGCAACA

IRN3|AB863138.1| TATTCCATATTCCGACCGTCTATCAACAGGAAAGTGGAATCGATTTCATCATCGGCAACA

IRN4|AB863139.1| TATTTCATATTCCTACCGTCTATCAACAGGAAAGTGGAATCGATTTCATCATCGGCAACA

IRN5|AB863140.1| TATTCCATATTCCTACCGTCTATCAACAGGAAAGTGGAATCGATTTCATCATCGGCAACA

IRN6|AB863141.1| TATTCCATATTCCTACCGTCTATCAACAGGAAAGTGGAATCGATTTCATCATCGGCAACA

IRN7|AB863142.1| TATTCCATATTCCTACCGTCTATCAACAGGAAAGTGGAATCGATTTCATCATCGGCAACA

IRN8|AB863143.1| TATTCCATATTCCTACCGTCTATCAACAGGAAAGTGGAATCGATTTCATCATCGGCAACA

IRN9|AB863144.1| TATTCCATATTCCTACCGTCTATCAACAGGAAAGTGGAATCGATTTCATCATCGGCAACA

IRN10|AB863145.1| TATTCCATATTCCTACCGTCTATCAACAGGAAAGTGGAATCGATTTCATCATCGGCAACA

IRN11|AB863146.1| TATTCCATATTCCTACCGTCTATCAACAGGAAAGTGGAATCGATTTCATCATCGGCAACA

IRN12|AB863147.1| TATTCCATATTCCTACCGTCTATCAGCAGGAAAGTGGAATCGATTTCATCATCGGCAACA

IRN13|AB863148.1| TATTCCATATTCCTACCGTCTATCAACAGGAAAGTGGAATCGATTTCATCATCGGCAACA

IRN14|AB863149.1| TATTTCATATTCCTACCGTCTATCAACAGGAAAGTGGAATCGATTTCATCATCGGCAACA

IRN15|AB863150.1| TATTTCATATTCCTACCGTCTATCAACAGGAAAGTGGAATCGATTTCATCATCGGCAACA

IRN16|AB863151.1| TATTCCATATTCCTACCGTCTATCAACAGGAAAGTGGAATCGATTTCATCATCGGCAACA

IRN17|AB863152.1| TATTCCATATTCCTACCGTCTATCAACAGGAAAGTGGAATCGATTTCATCATCGGCAACA

IRN18|AB863153.1| TATTCCATATTCCTACCGTCTATCAACAGGAAAGTGGAATCGATTTCATCATCGGCAACA

IRN19|AB863154.1| TATTCCATATTCCTACCGTCTATCAACAGGAAAGTGGAATCGATTTCATCATCGGCAACA

IRN20|AB863155.1| TATTTCATATTCCTACCGTCTATCAACAGGAAAGTGGAATCGATTTCATCATCGGCAACA

IRN21|AB863156.1| TATTCCATATTCCTACCGTCTATCAGCAGGAAAGTGGAATCGATTTCATCATCGGCAACA

JPNHGB340|AB863157.1| TATTCAAAATTCCCACCGTCTATCAGCAAGAAAGTGGCATCGATTTCATAATCGGCAACA

JPNKWB778|AB863158.1| TATTCAAAATTCCCACCGTCTATCAGCAAGAAAGTGGCATCGATTTCATAATCGGCAACA

JPNM|AB863159.1| TATTCAAAATTCCGACCGTCTATCAGCAAGAAAGTGGCATCGATTTCATAATCGGCAACA

JPNN|AB863160.1| TATTCAAAATTCCCACCGTCTATCAGCAAGAAAGTGGCATCGATTTCATCATCGGCAACA

JPNS1|AB863161.1| TATTCAAAATTCCCACCGTCTATCAGCAAGAAAGTGGCATCGATTTCATCATCGGCAACA

JPNS2|AB863162.1| TATTCAAAATTCCCACCGTCTATCAGCAAGAAAGTGGCATCGATTTCATCATCGGCAACA

JPNTKD762|AB863163.1| TATTCAAAATTCCCACCGTCTATCAGCAAGAAAGTGGCATCGATTTCATAATCGGCAACA

JPNUV1|AB863164.1| TATTCAAAATTCCCACCGTCTATCAGCAAGAAAGTGGCATCGATTTCATAATCGGCAACA

JPNUV26|AB863165.1| TATTCAAAATTCCGACCGTCTATCAGCAAGAAAGTGGCATCGATTTCATAATCGGCAACA

TUR1|AB863166.1| TATTCCATATTCCTACCGTCTATCAACAGGAAAGTGGAATCGATTTCATCATCGGCAACA

TUR2|AB863167.1| TATTTCATATTCCTACCGTCTATCAACAGGAGAGTGGAATCGATTTCATCATCGGCAACA

TUR4|AB863168.1| TATTTCATATTCCTACCGTCTATCAACAGGAGAGTGGAATCGATTTCATCATCGGCAACA

TUR5|AB863169.1| TATTCCATATTCCTACCGTCTATCAACAGGAAAGTGGAATCGACTTCATCATCGGCAACA

TUR12|AB863170.1| TATTCCATATTCCTACCGTCTATCAGCAGGAAAGTGGAATCGATTTCATCATCGGTAACA

TUR34|AB863171.1| TATTCCATATTCCTACCGTCTATCAGCAGGAAAGTGGAATCGATTTCATCATCGGCAACA

TUR50|AB863172.1| TATTCCATATTCCCACCGTCTATCAGCAGGAAAGTGGAATCGATTTCATCATCGGCAACA

TUR59|AB863173.1| TATTCCATATTCCTACCGTCTATCAACAGGAAAGTGGAATCGATTTCATCATCGGCAACA

TUR69|AB863174.1| TATTCCATATTCCTACCGTCTATCAACAGGAAAGTGGAATCGATTTCATCATCGGCAACA

TUR81|AB863175.1| TATTCCATATTCCTACCGTCTATCAACAGGAAAGTGGAATCGATTTCATCATCGGCAACA

TUR84|AB863176.1| TATTCCATATTCCTACCGTCTATCAACAGGAAAGTGGAATCGATTTCATCATCGGCAACA

TUR94|AB863177.1| TATTCCATATTCCTACCGTCTATCAACAGGAAAGTGGAATCGATTTCATCATCGGCAACA

TUR213|AB863178.1| TATTCCATATTCCTACCGTCTATCAACAGGAAAGTGGAATCGATTTCATCATCGGCAACA

TUR214|AB863179.1| TATTCCATATTCCTACCGTCTATCAACAGGAAAGTGGAATCGATTTCATCATCGGCAACA

TUR216|AB863180.1| TATTTCATATTCCAACCGTCTATCAACAAGAAAGTGGAATCGATTTCATCATCGGCAACA

TUR220|AB863181.1| TATTCCATATTCCTACCGTCTATCAACAGGAAAGTGGAATCGATTTCATCATCGGCAACA

TUR239|AB863182.1| TATTCCATATTCCTACCGTCTATCAACAGGAAAGTGGAATCGATTTCATCATCGGCAACA

TUR244|AB863183.1| TATTTCATATTCCTACCGTCTACCAACAGGAAAGTGGAATCGATTTCATCATCGGCAACA

TUR249|AB863184.1| TATTTCATATTCCTACCGTCTATCAACAGGAAAGTGGAATCGATTTCATCATCGGCAACA

TUR263|AB863185.1| TATTTCATATTCCTACCGTCTATCAACAGGAAAGTGGAATCGATTTCATCATCGGCAACA

TUR278|AB863186.1| TATTCCATATTCCTACCGTCTATCAACAGGAAAGTGGGATCGATTTCATCATCGGCAACA

TUR279|AB863187.1| TATTCCATATTCCTACCGTCTATCAACAGGAAAGTGGAATCGATTTCATCATCGGCAACA

TUR285|AB863188.1| TATTCCATATTCCTACCGTCTATCAGCAGGAAAGTGGAATCGATTTCATCATCGGCAACA

TUR289|AB863189.1| TATTCCATATTCCTACCGTCTATCAACAGGAAAGTGGAATCGATTTCATCATCGGCAACA

TUR303|AB863190.1| TATTCCATATTCCTACCGTCTATCAACAGGAAAGTGGAATCGATTTCATCATCGGCAACA

TUR306|AB863191.1| TATTCCATATTCCTACCGTCTATCAACAGGAAAGTGGAATCGATTTCATCATCGGCAACA

Cabb-S|NC_001497.1| ACTTCTGTCAGCTGTATGAACCATTCATACAGTTTACGGATAGAGTTATCTTCACAAAGA

CRO180A|AB863192.1| ACTTCTGTCAGTTGTATGAACCTTTCATACAATTTACAGATAGAGTTATCTTCACAAAGG

GRC83|AB863193.1| ACTTCTGTCAGTTGTATGAACCCTTCATACAATTTACAGATAGAGTTATCTTCACAAAGG

GRC84B|AB863194.1| ACTTCTGTCAGTTGTATGAACCCTTCATACAATTTACAGATAGAGTTATCTTCACAAAGG

GRC86B|AB863195.1| ACTTCTGTCAGTTGTATGAACCCTTCATACAATTTACAGATAGAGTTATCTTCACAAAGG

GRC86D|AB863196.1| ACTTCTGTCAGTTGTATGAACCCTTCATACAATTTACAGATAGAGTTATCTTCACAAAGG

GRC87E|AB863197.1| ACTTCTGTCAGTTGTATGAACCCTTCATACAATTTACAGATAGAGTTATCTTCACAAAGG

GRC87G|AB863198.1| ACTTCTGTCAGTTGTATGAACCCTTCATACAATTTACAGATAGAGTTATCTTCACAAAGG

GRC91B|AB863199.1| ACTTCTGTCAGTTGTATGAACCCTTCATACAATTTACAGATAGAGTTATCTTCACAAAGG

GRC92A|AB863200.1| ACTTCTGTCAGTTGTATGAACCCTTCATACAATTTACAGATAGAGTTATCTTCACAAAGG

GRC92C|AB863201.1| ACTTCTGTCAGTTGTATGAACCCTTCATACAATTTACAGATAGAGTTATCTTCACAAAGG

GRC92D|AB863202.1| ACTTCTGTCAGTTGTATGAACCCTTCATACAATTTACAGATAGAGTTATCTTCACAAAGG

IRN1|AB863136.1| ACTTCTGTCAGTTGTATGAACCCTTCATACAATTTACGGATAGAGTTATCTTCACAAAGG

IRN2|AB863137.1| ACTTCTGTCAGTTGTATGAACCTTTCATACAATTTACAGATAGAGTTATCTTCACAAAGG

IRN3|AB863138.1| ACTTCTGTCAGTTGTATGAACCCTTCATACAATTTACGGATAGAGTTATCTTCACAAAGG

IRN4|AB863139.1| ACTTCTGTCAGTTGTATGAACCTTTCATACAATTTACAGATAGAGTTATCTTCACAAAGG

IRN5|AB863140.1| ACTTCTGTCAGTTGTATGAACCTTTCATACAATTTACAGATAGAGTTATCTTCACAAAGG

IRN6|AB863141.1| ACTTCTGTCAGTTGTATGAACCTTTCATACAATTTACAGATAGAGTTATCTTCACAAAGG

IRN7|AB863142.1| ACTTCTGTCAGTTGTATGAACCTTTCATACAATTTACAGATAGAGTTATCTTCACAAAGG

IRN8|AB863143.1| ACTTCTGTCAGTTGTATGAACCTTTCATACAATTTACAGATAGAGTTATCTTCACAAAGG

IRN9|AB863144.1| ACTTCTGTCAGTTGTATGAACCTTTCATACAATTTACAGATAGAGTTATCTTCACAAAGG

IRN10|AB863145.1| ACTTCTGTCAGTTGTATGAGCCTTTCATACAATTTACAGATAGAGTTATCTTCACAAAGG

IRN11|AB863146.1| ACTTCTGTCAATTGTATGAACCTTTCATACAATTTACAGATAGAGTTATCTTCACAAAGG

IRN12|AB863147.1| ACTTCTGTCAGTTGTATGAACCTTTCATACAATTTACAGATAGAGTTATCTTCACAAAGG

IRN13|AB863148.1| ACTTCTGTCAGTTGTATGAACCTTTCATACAATTTACAGATAGAGTTATCTTCACAAAGG

IRN14|AB863149.1| ACTTCTGTCAGTTGTATGAACCTTTCATACAATTTACAGATAGAGTCATCTTCACAAAAG

IRN15|AB863150.1| ACTTCTGTCAGTTGTATGAACCTTTCATACAATTCACAGATAGAGTTATCTTCACAAAGG

IRN16|AB863151.1| ATTTCTGTCAGTTGTATGAACCTTTCATACAATTCACAGATAGAGTTATCTTCACAAAGG

IRN17|AB863152.1| ACTTCTGTCAGTTGTATGAACCTTTCATACAATTCACAGATAGAGTTATCTTCACAAAGG

IRN18|AB863153.1| ACTTCTGTCAATTGTATGAACCTTTCATACAATTTACAGATAGAGTTATCTTCACAAAGG

IRN19|AB863154.1| ACTTCTGTCAGTTGTATGAACCTTTCATACAATTTACAGATAGAGTTATCTTCACAAAAG

IRN20|AB863155.1| ACTTCTGTCAGTTGTATGAACCTTTCATACAATTCACAGATAGAGTTATCTTCACAAAGG

IRN21|AB863156.1| ACTTCTGTCAGTTGTATGAACCTTTCATACAATTTACAGATAGAGTCATCTTCACAAAGG

JPNHGB340|AB863157.1| ACTTTTGTCAGCTGTATGAACCATTCATACAGTTTACAGATAGAGTTATCTTCACAAAGA

JPNKWB778|AB863158.1| ACTTTTGTCAGCTGTATGAACCATTCATACAGTTTACAGATAGAGTTATCTTCACAAAGA

JPNM|AB863159.1| ACTTTTGTCAGCTGTATGAACCATTCATACAGTTTACAGATAGAGTTATCTTCACAAAGA

JPNN|AB863160.1| ACTTCTGTCAGTTGTATGAACCTTTCATACAATTTACAGATAGAGTTATCTTCACAAAGA

JPNS1|AB863161.1| ACTTCTGTCAGTTGTATGAACCTTTCATACAATTTACAGATAGAGTTATCTTCACAAAGA

JPNS2|AB863162.1| ACTTCTGTCAGTTGTATGAACCTTTTATACAATTTACAGATAGAGTTATCTTCACAAAGA

JPNTKD762|AB863163.1| ACTTTTGTCAGCTGTATGAACCATTCATACAGTTTACAGATAGAGTTATCTTCACAAAGA

JPNUV1|AB863164.1| ACTTCTGTCAGCTGTATGAACCATTCATACAGTTTACAGATAGAGTTATCTTCACAAAGA

JPNUV26|AB863165.1| ACTTTTGTCAGCTGTATGAACCATTCATACAGTTTACAGATAGAGTTATCTTCACAAAGA

TUR1|AB863166.1| ACTTCTGTCAGTTGTATGAACCTTTCATACAATTCACAGATAGAGTTATCTTCACAAAGG

TUR2|AB863167.1| ACTTCTGTCAGTTGTATGAACCTTTCATACAATTCACAGATAGAGTTATCTTCACAAAAG

TUR4|AB863168.1| ACTTCTGTCAGTTGTATGAACCTTTCATACAATTCACAGATAGAGTTATCTTCACAAAAG

TUR5|AB863169.1| ACTTCTGTCAGTTGTATGAACCTTTCATACAATTCACAGATAGAGTTATCTTCACAAAGG

TUR12|AB863170.1| ACTTCTGTCAGTTGTATGAACCATTCATACAATTTACAGATAGAGTTATCTTCACAAAGG

TUR34|AB863171.1| ACTTCTGTCAGTTGTATGAACCTTTCATACAATTCACAGATAGAGTTATCTTCACAAAGG

TUR50|AB863172.1| ACTTCTGTCAGTTGTATGAACCTTTCATACAATTCACAGATAGAGTTATCTTCACAAAGG

TUR59|AB863173.1| ACTTCTGTCAGTTGTATGAACCTTTCATACAATTTACAGATAGAGTTATCTTCACAAAGG

TUR69|AB863174.1| ACTTCTGTCAGTTGTATGAACCTTTCATACAATTCACAGATAGAGTTATCTTCACAAAGG

TUR81|AB863175.1| ACTTCTGTCAGTTGTATGAACCTTTCATACAATTCACAGATAGAGTTATCTTCACAAAGG

TUR84|AB863176.1| ACTTCTGTCAGTTGTATGAACCTTTCATACAATTCACAGATAGAGTTATCTTCACAAAGG

TUR94|AB863177.1| ACTTCTGTCAGTTGTATGAACCTTTCATACAATTCACAGATAGAGTTATCTTCACAAAGG

TUR213|AB863178.1| ACTTCTGTCAGTTGTATGAACCTTTCATACAATTCACAGATAGAGTTATCTTCACAAAGG

TUR214|AB863179.1| ACTTCTGTCAGTTGTATGAACCTTTCATACAATTCACAGATAGAGTTATCTTCACAAAAG

TUR216|AB863180.1| ACTTCTGTCAGTTGTATGAACCCTTCATACAATTTACAGATAGAGTTATCTTCACAAAGG

TUR220|AB863181.1| ACTTCTGTCAGTTGTATGAACCTTTCATACAATTCACAGATAGAGTTATCTTCACAAAGG

TUR239|AB863182.1| ACTTCTGTCAGTTGTATGAACCTTTCATACAATTTACAGATAGAGTTATCTTCACAAAGG

TUR244|AB863183.1| ACTTCTGTCAGTTGTATGAACCTTTCATACAATTCACAGATAGAGTTATCTTCACAAAAG

TUR249|AB863184.1| ACTTCTGTCAGTTGTATGAACCTTTCATACAATTCACAGATAGAGTTATCTTCACAAAGG

TUR263|AB863185.1| ACTTCTGTCAGTTGTATGAACCTTTCATACAATTTACAGATAGAGTTATCTTCACAAAGG

TUR278|AB863186.1| ACTTCTGTCAGTTGTATGAACCTTTCATACAATTCACAGATAGAGTTATTTTCACAAAGG

TUR279|AB863187.1| ACTTCTGTCAGTTGTATGAACCTTTCATACAATTCACAGATAGAGTTATCTTCACAAAGG

TUR285|AB863188.1| ACTTCTGTCAGTTGTATGAACCATTCATACAATTTACAGATAGAGTTATCTTCACAAAGG

TUR289|AB863189.1| ACTTCTGTCAGTTGTATGAACCTTTCATACAATTCACAGATAGAGTTATCTTCACAAAGG

TUR303|AB863190.1| ACTTCTGTCAGTTGTATGAACCTTTCATACAATTCACAGATAGAGTTATCTTCACAAAGG

TUR306|AB863191.1| ACTTCTGTCAGTTGTATGAACCGTTCATACAATTCACAGATAGAGTTATCTTCACAAAGG

Cabb-S|NC_001497.1| ACAAGTCTTATCCTGTTCATATTGCGAAGCTAACCAGAGCAGTGCGAGTAGGCACCGAAG

CRO180A|AB863192.1| ACAGAACATACCCTGTTCATATTGCGAAGCTAACAAGAGCAGTGCGAGTAGGCACCGAAG

GRC83|AB863193.1| ACAGAACATATCCTGTTCATATTGCGAAGCTAACAAGAGCAGTGCGAGTAGGCACCGAAG

GRC84B|AB863194.1| ACAGAACATACCCTGTTCATATTGCGAAGCTAACAAGAGCAGTGCGAGTAGGCACCAAAG

GRC86B|AB863195.1| ACAGAACATATCCTGTTCATATTGCGAAGCTAACAAGAGCAGTGCGAGTAGGCACCGAAG

GRC86D|AB863196.1| ACAGAACATATCCTGTTCATATTGCGAAGCTAACAAGAGCAGTGCGAGTAGGCACCGAAG

GRC87E|AB863197.1| ACAGAACATACCCTGTTCATATTGCGAAGCTAACAAGAGCAGTGCGAGTAGGCACCGAAG

GRC87G|AB863198.1| ACAGAACATATCCTGTTCATATTGCGAAGCTAACAAGAGCAGTGCGAGTAGGCACCGAAG

GRC91B|AB863199.1| ACAGAACATATCCTGTTCATATTGCGAAGCTAACAAGAGCAGTGCGAGTAGGCACCGAAG

GRC92A|AB863200.1| ACAGAACATATCCTGTTCATATTGCGAAGCTAACAAGAGCAGTGCGAGTAGGCACCGAAG

GRC92C|AB863201.1| ACAGAACATATCCTGTTCATATTGCGAAGCTAACAAGAGCAGTGCGAGTAGGCACCGAAG

GRC92D|AB863202.1| ACAGAACATATCCTGTTCATATTGCGAAGCTAACAAGAGCAGTGCGAGTAGGCACCGAAG

IRN1|AB863136.1| ACAGAACATACCCTGTTCATATTGCGAAGCTAACAAGAGCAGTGCGAGTAGGCACCGAAG

IRN2|AB863137.1| ACAGAACATACCCTGTTCATATTGCGAAGCTAACAAGAGCAGTGCGAGTAGGCACCGAAG

IRN3|AB863138.1| ACAGAACATACCCTGTTCATATTGCGAAGCTAACAAGAGCAGTGCGAGTAGGCACCGAAG

IRN4|AB863139.1| ACAGAACATACCCTGTTCATATTGCGAAGTTAACAAGAGCAGTGCGAGTAGGCACCGAAG

IRN5|AB863140.1| ACAGAACATACCCTGTTCATATTGCGAAGCTAACAAGAGCAGTGCGAGTAGGCACCGAAG

IRN6|AB863141.1| ACAGAACATACCCTGTTCATATTGCGAAGCTAACAAGAGCAGTGCGAGTAGGCACCGAAG

IRN7|AB863142.1| ACAGAACATACCCTGTTCATATTACGAAGCTAACAAGAGCAGTGCGAGTAGGCACCGAAG

IRN8|AB863143.1| ACAGAACATACCCTGTTCATATTGCGAAGCTAACAAGAGCAGTGCGAGTAGGCACCGAAG

IRN9|AB863144.1| ACAGAACATACCCTGTTCATATTGCGAAGCTAACAAGAGCAGTGCGAGTAGGCACCGAAG

IRN10|AB863145.1| ACAGAACATACCCCGTTCATATTGCGAAGCTAACAAGAGCAGTGCGAGTAGGCACCGAAG

IRN11|AB863146.1| ACAGAACATACCCTGTTCATATTGCGAAGCTAACAAGAGCAGTGCGAGTAGGCACCGAAG

IRN12|AB863147.1| ACAGAACATACCCTGTTCATATTGCGAAGCTAACAAAAGCAGTGCGAGTAGGCACCGAAG

IRN13|AB863148.1| ACAGAACATACCCTGTTCATATTGCGAAGCTAACAAGAGCAGTGCGAGTAGGCACCGAAG

IRN14|AB863149.1| ACAGAACATATCCTGTTCATATTGCGAAGTTAACAAGAGCAGTGCGAGTAGGCACCGAAG

IRN15|AB863150.1| ACAGAACATACCCTGTTCATATTGCGAAGCTAACAAGAGCAGTGCGAGTAGGCACCGAAG

IRN16|AB863151.1| ACAGAACATACCCTGTTCATATTGCGAAGCTAACAAGAGCAGTGCGAGTAGGCACCGAAG

IRN17|AB863152.1| ACAGAACATACCCTGTTCATATTGCGAAACTAACAAGAGCAGTGCGAGTAGGCACCGAAG

IRN18|AB863153.1| ACAGAACATACCCTGTTCATATTGCGAAGCTAACAAGAGCAGTGCGGGTAGGCACCGAAG

IRN19|AB863154.1| ACAGAACATACCCTGTTCATATTGCGAAGCTAACAAGAGCAGTGCGAGTAGGCACCGAAG

IRN20|AB863155.1| ACAGAACATACCCTGTTCATATTGCGAAGCTAACAAGAGCAGTGCGAGTAGGCACCGAAG

IRN21|AB863156.1| ACAGAACATACCCTGTTCATATTGCGAAGCTAACAAGAGCAGTGCGAGTAGGCACCGAAG

JPNHGB340|AB863157.1| ACAAATCCTATCCTGTTCATATTGCGAAGCTAACAAGAGCGATGCGAGTAGGCACCGAAG

JPNKWB778|AB863158.1| ACAAATCCTATCCTGTTCATATTGCGAAGCTAACAAGAGCGATGCGAGTAGGCACCGAAG

JPNM|AB863159.1| ACAAATCCTATCCTGTTCATATTGCGAAGCTAACAAGAGCAGTGCGAGTAGGCACCGAAG

JPNN|AB863160.1| ACAAGACTTATCCTGTTCATATTGCGAAGCTAACAAAAGCAGTGCGAATAGGCACCGAAG

JPNS1|AB863161.1| ACAAGACTTATCCTGTTCATATTGCGAAGCTAACAAAAGCAGTGCGAGTAGGCACCGAAG

JPNS2|AB863162.1| ACAAGACTTATCCTGTTCATATTGCGAAGCTAACAAAAGCAGTGCGAGTAGGCACCGAAG

JPNTKD762|AB863163.1| ACAAATCCTATCCTGTTCATATTGCGAAGATAACAAGAGCAGTGCGAGTAGGCACCGAAG

JPNUV1|AB863164.1| ACAAATCCTATCCTGTTCATATTGCGAAGCTAACAAGAGCAGTGCGAGTAGGCACCGAAG

JPNUV26|AB863165.1| ACAAATCCTATCCTGTTCATATTGCGAAGCTAACAAGAGCAGTGCGAGTAGGCACCGAAG

TUR1|AB863166.1| ACAGAACATACCCTGTTCATATTGCGAAGCTAACAAGAGCAGTGCGAGTGGGCACCGAAG

TUR2|AB863167.1| ACAGAACATACCCTGTTCATATTGCGAAGCTAACAAGAGCAGTGCGAGTAGGCACCGAAG

TUR4|AB863168.1| ACAGAACATACCCTGTTCATATTGCGAAGCTAACAAGAGCAGTGCGAGTAGGCACCGAAG

TUR5|AB863169.1| ACAGAACATACCCTGTTCATATTGCGAAGCTAACAAGAGCAGTGCGAGTAGGCACCGAAG

TUR12|AB863170.1| ACAGAACATACCCTGTTCATATTGCAAAGCTAACAAGAGCAGTGCGAGTAGGCACCGAAG

TUR34|AB863171.1| ACAGAACATACCCTGTTCATATTGCGAAGCTAACAAGAGCAGTGCGAGTAGGCACCGAAG

TUR50|AB863172.1| ACAGAACATACCCTGTTCATATTGCGAAGCTAACAAGAGCAGTGCGAGTAGGCACCGAAG

TUR59|AB863173.1| ACAGAACATACCCTGTTCATATTGCGAAGCTAACAAGAGCAGTGCGAGTAGGCACCGAAG

TUR69|AB863174.1| ACAGAACATACCCTGTTCATATTGCGAAGCTAACAAGAGCAGTGCGAGTAGGCACCGAAG

TUR81|AB863175.1| ACAGAACATACCCTGTTCATATTGCGAAGCTAACAAGAGCAGTGCGAGTAGGCACCGAAG

TUR84|AB863176.1| ACAGAACATACCCTGTTCATATTGCGAAGCTAACAAGAGCAGTGCGAGTAGGCACCGAAG

TUR94|AB863177.1| ACAGAACATACCCTGTTCATATTGCGAAGCTAACAAGAGCAGTGCGAGTAGGCACCGAAG

TUR213|AB863178.1| ACAGAACATACCCTGTTCATATTGTGAAGCTAACAAGAGCAGTGCGAGTAGGCACCGAAG

TUR214|AB863179.1| ACAGAACATACCCTGTTCATATTGCGAAGCTAACAAGAGCAGTGCGAGTAGGCACCGAAG

TUR216|AB863180.1| ACAAGACGTATCCTGTCCATATAGCGAAGTTAACAAGAGCAGTGCGAGTAGGCACAGAAG

TUR220|AB863181.1| ACAGAACATACCCTGTTCATATTGCGAAGCTAACAAGAGCAGTGCGAGTAGGCACCGAAG

TUR239|AB863182.1| ACAGAACATACCCTGTTCATATTGCGAAGCTAACAAGAGCAGTGCGAGTAGGCACCGAAG

TUR244|AB863183.1| ACAGAACATACCCTGTTCATATTGCGAAGCTAACAAGAGCAGTGCGAGTAGGCACCGAAG

TUR249|AB863184.1| ACAGAACATACCCTGTTCATATTGCGAAGCTAACAAGAGCAGTGCGAGTAGGCACCGAAG

TUR263|AB863185.1| ACAGAACATACCCTGTTCATATTGCGAAGCTAACAAGAGCAGTGCGAGTAGGCACCGAAG

TUR278|AB863186.1| ACAGAACATACCCTGTTCACATTGCGAAGCTAACAAGAGCAGTGCGAGTAGGCACCGAAG

TUR279|AB863187.1| ACAGAACATACCCTGTTCATATTGCGAAGCTAACAAGAGCAGTGCGAGTAGGCACCGAAG

TUR285|AB863188.1| ACAGAACATACCCTGTTCATATTGCGAAGCTAACAAGAGCAGTGCGAGTAGGCACCGAAG

TUR289|AB863189.1| ACAGAACATACCCTGTTCATATTGTGAAGCTAACAAGAGCAGTGCGAGTAGGCACCGAAG

TUR303|AB863190.1| ACAGAACATACCCTGTTCATATTGCGAAGCTAACAAGAGCAGTGCGAGTAGGCACCGAAG

TUR306|AB863191.1| ACAGAACATACCCTGTTCATATTGCGAAGCTAACAAGAGCAGTGCGAGTAGGCACCGAAG

Cabb-S|NC_001497.1| GATTTCTTGAATCAATGAAGAAACGTTCAAAAACTCAACAACCAGAGCCAGTGAACATTT

CRO180A|AB863192.1| GATTCTTAGAATCCATGAAGAAACGTTCAAAGACTCAGCAACCGGAGCCAGTGAACATTT

GRC83|AB863193.1| GTTTCCTAGAATCCATGAAGAAACGTTCAAAGACTCAGCAACCGGAGCCAGTGAACATTT

GRC84B|AB863194.1| GATTCCTAGAATCCATGAAGAAACGTTCAAAGACTCAGCAACCGGAGCCAGTGAACATTT

GRC86B|AB863195.1| GATTCTTAGAATCCATGAAGAAACGTTCAAAGACTCAGCAACCGGAGCCTGTGAACATTT

GRC86D|AB863196.1| GTTTCCTAGAATCCATGAAGAAACGTTCAAAGACTCAGCAACCGGAGCCAGTGAACATTT

GRC87E|AB863197.1| GATTCCTAGAATCCATGAAGAAACGTTCAAAGACTCAACAACCGGAGCCAGTGAACATTT

GRC87G|AB863198.1| GATTCCTAGAATCCATGAAGAAACGTTCAAAGACTCAACAACCGGAGCCAGTGAACATTT

GRC91B|AB863199.1| GATTTCTAGAATCCATGAAGAAACGTTCAAAGACTCAACAACCGGAGCCAGTGAACATTT

GRC92A|AB863200.1| GATTCCTAGAATCCATGAAGAAACGTTCAAAAACTCAGCAACCGGAGCCAGTGAACATTT

GRC92C|AB863201.1| GATTCCTAGAATCCATGAAGAAACGTTCAAAGACTCAGCAACCGGAGCCAGTGAACATTT

GRC92D|AB863202.1| GATTCTTAGAATCCATGAAGAAACGTTCAAAGACTCAACAACCGGAGCCAGTGAACATTT

IRN1|AB863136.1| GATTCCTAGAATCCATGAAGAAACGTTCGAAGACTCAGCAACCGGAGCCAGTGAACATTT

IRN2|AB863137.1| GATTCCTAGAATCCATGAAGAAACGTTCGAAGACTCAGCAACCGGAGCCAGTGAACATTT

IRN3|AB863138.1| GATTCCTAGAATCCATGAAGAAACGTTCGAAGACTCAGCAACCGGAGCCAGTGAACATTT

IRN4|AB863139.1| GATTCCTAGAATCTATGAAGAAACGTTCAAAGACTCATCAACCGGAGCCAGTGAACATTT

IRN5|AB863140.1| GATTCCTAGAATCCATGAAGAAACGTTCGAAGACTCAGCAACCGGAGCCTGTGAACATTT

IRN6|AB863141.1| GATTCCTAGAATCCATGAAGAAACGTTCGAAGACTCAGCAACCGGAGCCAGTGAACATTT

IRN7|AB863142.1| GATTCCTAGAATCCATGAAGAAACGTTCGAAGACTCAGCAACCGGAGCCAGTGAACATTT

IRN8|AB863143.1| GATTCCTAGAATCCATGAAGAAACGTTCGAAGACTCAGCAACCGGAGCCTGTGAACATTT

IRN9|AB863144.1| GATTCCTAGAATCCATGAAGAAACGTTCGAAGACTCAGCAACCGGAGCCAGTAAACATTT

IRN10|AB863145.1| GATTCCTAGAATCCATGAAGAAACGTTCGAAGACTCAGCAACCGGAGCCAGTGAACATTT

IRN11|AB863146.1| GATTCCTAGAATCCATGAAGAAACGTTCGAAGACTCAGCAACCGGAGCCAGTGAACATTT

IRN12|AB863147.1| GATTCCTAGAATCCATGAAGAAACGTTCGAAGACTCAGCAACCGGAGCCAGTGAACATTT

IRN13|AB863148.1| GATTCCTAGAATCCATGAAGAAACGTTCGAAGACTCAGCAACCGGAGCCAGTGAACATTT

IRN14|AB863149.1| GATTCCTAGAATCCATGAAGAAACGTTCGAAGACTCAGCAACCGGAGCCAGTGAACATTT

IRN15|AB863150.1| GATTTCTAGAATCCATGAAGAAACGTTCAAAGACTCAGCAACCGGAGCCAGTGAACATTT

IRN16|AB863151.1| GATTTCTAGAATCCATGAAGAAACGTTCAAAGACTCAGCAACCGGAGCCAGTGAACATTT

IRN17|AB863152.1| GATTTCTAGAATCCATGAAGAAACGTTCAAAGACTCAGCAACCGGAGCCAGTGAACATTT

IRN18|AB863153.1| GATTCCTAGAATCCATGAAGAAACGTTCAAAGACTCAGCAACCGGAGCCAGTGAACATTT

IRN19|AB863154.1| GATTCCTAGAATCCATGAAGAAACGTTCGAAGACTCAGCAACCGGAGCCAGTGAACATTT

IRN20|AB863155.1| GATTTCTAGAATCCATGAAGAAACGTTCAAAGACTCAGCAACCGGAGCCAGTGAACATTT

IRN21|AB863156.1| GATTCCTAGAATCCATGAAGAAACGTTCGAAGACTCAGCAACCGGAGCCAGTGAACATTT

JPNHGB340|AB863157.1| GATTTCTTGAATCAATGAAGAAACGTTCAAAGACTCAACAACCAGAGCCAGTGAACATCT

JPNKWB778|AB863158.1| GATTTCTTGAATCAATGAAGAAACGTTCAAAGACTCAACAACCAGAGCCAGTGAACATTT

JPNM|AB863159.1| GATTTCTTGAATCAATGAAGAAACGTTCAAAGACTCAACAACCAGAGCCAGTGAACATTT

JPNN|AB863160.1| GATTTCTAGACTCTATGAAGAAACGTTCAAAGACTCAACAACCAGAGCCGGTGAACATTT

JPNS1|AB863161.1| GATTTCTAGACTCTATGAAGAAACGTTCAAAGACTCAACAACCAGAGCCAGTGAACATTT

JPNS2|AB863162.1| GATTTCTAGACTCTATGAAGAAACGTTCAAAGACTCAACAACCAGAGCCAGTGAACATTT

JPNTKD762|AB863163.1| GATTTCTTGAATCAATGAAGAAACGTTCAAAGACTCAACAACCAGAGCCAGTGAACATTT

JPNUV1|AB863164.1| GATTTCTTGAATCAATGAAGAAACGTTCAAAGACTCAACAACCAGAGCCAGTGAACATTT

JPNUV26|AB863165.1| GATTTCTTGAAGCAATGAAGAAACGTTCAAAGACTCAACAACCAGAGCCAGTGAACATTT

TUR1|AB863166.1| GATTCCTAGAATCCATGAAGAAACGTTCAAAAACTCAGCAACCGGAGCCAGTGAACATTT

TUR2|AB863167.1| GATTTCTAGAATCCATGAAGAAACGTTCAAAGACTCAGCAACCGGAGCCAGTGAACATTT

TUR4|AB863168.1| GATTTCTAGAATCCATGAAGAAACGTTCAAAGACTCAGCAACCGGAGCCAGTGAACATTT

TUR5|AB863169.1| GATTTTTAGAATCCATGAAGAAACGTTCAAAGACTCAGCAACCGGAGCCAGTGAACATTT

TUR12|AB863170.1| GATTCCTAGAATCCATGAAGAAACGTTCAAAGACTCAGCAACCAGAGCCGGTGAACATTT

TUR34|AB863171.1| GATTCCTAGAATCCATGAAGAAACGTTCAAAGACTCAGCAACCGGAGCCAGTGAACATTT

TUR50|AB863172.1| GATTCTTAGAATCCATGAAGAAACGTTCAAAGACTCAGCAACCGGAGCCAGTGAACATTT

TUR59|AB863173.1| GATTCCTAGAATCCATGAAGAAACGTTCAAAGACTCAGCAACCGGAGCCAGTGAACATTT

TUR69|AB863174.1| GATTTCTAGAATCCATGAAGAAACGTTCAAAGACTCAGCAACCAGAGCCAGTGAACATTT

TUR81|AB863175.1| GATTTCTAGAATCCATGAAGAAACGTTCAAAGATTCAGCAACCAGAGCCAGTGAACATTT

TUR84|AB863176.1| GATTTCTAGAATCCATGAAGAAACGTTCAAAGACTCAGCAACCAGAGCCAGTGAACATTT

TUR94|AB863177.1| GTTTTCTAGAATCCATGAAGAAACGTTCAAAGACTCAGCAACCGGAGCCAGTGAACATTT

TUR213|AB863178.1| GATTTCTAGAATCCATGAAGAAACGTTCAAAGACTCAACAACCGGAGCCAGTGAACATTT

TUR214|AB863179.1| GATTTCTAGAATCCATGAAGAAACGTTCAAAAACTCAGCAACCGGAGCCAGTGAACATTT

TUR216|AB863180.1| GATTCCTAGAATCCATGAAGAAGCGTTCAAAGACTCAACAACCAGAGCCTGTGAACATTT

TUR220|AB863181.1| GATTTCTAGAATCCATGAAGAAACGTTCAAAGACTCAGCAACCAGAGCCAGTGAACATTT

TUR239|AB863182.1| GATTCCTAGAATCCATGAAGAAACGTTCAAAGACTCAGCAACCGGAGCCAGTGAACATTT

TUR244|AB863183.1| GATTTCTAGAATCCATGAAGAAACGTTCAAAAACTCAGCAACCGGAGCCAGTAAACATTT

TUR249|AB863184.1| GATTTCTAGAATCCATGAAGAAACGTTCAAAGACTCAGCAACCGGAGCCAGTGAACATTT

TUR263|AB863185.1| GATTTCTAGAATCCATGAAGAAACGTTCAAAGACTCAGCAACCGGAGCCAGTGAACATTT

TUR278|AB863186.1| GATTTCTAGAATCCATGAAGAAACGTTCAAAGACTCATCAACCAGAGCCAGTGAACATTT

TUR279|AB863187.1| GATTTCTAGAATCCATGAAGAAACGTTCAAAGACTCAGCAACCAGAGCCAGTGAACATTT

TUR285|AB863188.1| GATTCCTAGAATCCATGAAGAAACGTTCAAAGACTCAGCAACCAGAGCCGGTGAACATTT

TUR289|AB863189.1| GATTTCTAGAATCCATGAAGAAACGTTCAAAGACTCAGCAACCAGAGCCAGTGAACATTT

TUR303|AB863190.1| GATTTCTAGAATCCATGAAGAAACGTTCAAAGACTCAGCAACCGGAGCCAGTGAACATTT

TUR306|AB863191.1| GATTTCTAGAATCCATGAAGAAACGTTCAAAGACTCAGCAACCGGAGCCAGTGAACATTT

Cabb-S|NC_001497.1| CTACAAACAAGATAGAAAATCCACTAGAAGAAATTGCTATTCTTTCAGAGGGGAGGAGGT

CRO180A|AB863192.1| CAACAAATAA---------------------AATTGCTATTCTTTCAGAGGGGAGGAGGT

GRC83|AB863193.1| CAACAAATAA---------------------AATTGCTATTCTTTCAGAGGGGAGGAGGT

GRC84B|AB863194.1| CAACAAATAA---------------------AATTGCTATTCTTTCAGAGGGGAGGAGGT

GRC86B|AB863195.1| CAACAAACAA---------------------AATTGCTATTCTTTCAGAGGGGAGGAGGT

GRC86D|AB863196.1| CAACAAATAA---------------------AATTGCTATTCTTTCAGAGGGGAGGAGGT

GRC87E|AB863197.1| CAACAAATAA---------------------AATTGCTATTCTTTCAGAGGGGAGGAGGT

GRC87G|AB863198.1| CAACAAATAA---------------------AATTGCTATTCTTTCAGAGGGGAGGAGGT

GRC91B|AB863199.1| CAACAAATAA---------------------AATTGCTATTCTTTCAGAGGGGAGGAGGT

GRC92A|AB863200.1| CAACAAATAA---------------------AATTGCTATTCTTTCAGAGGGGAGGAGGT

GRC92C|AB863201.1| CAACAAATAA---------------------AATTGCTATTCTTTCAGAGGGGAGGAGGT

GRC92D|AB863202.1| CAACAAACAA---------------------AATTGCTATTCTTTCAGAGGGGAGGAGGT

IRN1|AB863136.1| CAACAAACAA---------------------AATTGCTATTCTTTCAGGGGGGAGGAGGT

IRN2|AB863137.1| CAACAAATAA---------------------AATTGCTATTCTTTCAGAGGGGAGGAGGT

IRN3|AB863138.1| CAACAAACAA---------------------AATTGCTATTCTTTCAGGGGGGAGGAGGT

IRN4|AB863139.1| CAACAAATAA---------------------AATTGCTATTCTTTCAGAGGGGAGGAGGT

IRN5|AB863140.1| CAACAAACAA---------------------AATTGCTATTCTTTCAGAGGGGAGGAGGT

IRN6|AB863141.1| CAACAAAT---------------------AAAATTGCTATTCTTTCAGAGGGGAGGAGGT

IRN7|AB863142.1| CAACAAATAA---------------------AATTGCTATTCTTTCAGAGGGGAGGAGGT

IRN8|AB863143.1| CAACAAACAA---------------------AATTGCTATTCTTTCAGAGGGGAGGAGGT

IRN9|AB863144.1| CAACAAATAA---------------------AATTGCTATTCTTTCAGAGGGGAGGAGGT

IRN10|AB863145.1| CAACAAATAA---------------------AATTGCTATTCTTTCAGAGGGGAGGAGGT

IRN11|AB863146.1| CAACAAATAA---------------------AATTGCTATTCTTTCAGAGGGGAGGAGGT

IRN12|AB863147.1| CAACAAATAA---------------------AATTGCTATTCTTTCAGAGGGGAGGAGGT

IRN13|AB863148.1| CAACAAATAA---------------------AATTGCTATTCTTTCAGAGGGGAGGAGGT

IRN14|AB863149.1| CAACAAATAA---------------------AATTGCTATTCTTTCAGAGGGGAGGAGGT

IRN15|AB863150.1| CAACAAACAA---------------------AATTGCTATTCTTTCAGAGGGGAGGAGGT

IRN16|AB863151.1| CAACAAATAA---------------------AATTGCTATTCTTTCAGAGGGGAGGAGGT

IRN17|AB863152.1| CAACAAATAA---------------------AATTGCTATTCTTTCAGAGGGGAGGAGGT

IRN18|AB863153.1| CAACAAATAA---------------------AATTGCTATTCTTTCAGAGGGGAGGAGGT

IRN19|AB863154.1| CAACAAATAA---------------------AATTGCTATTCTTTCAGAGGGGAGGAGGT

IRN20|AB863155.1| CAACAAACAA---------------------AATTGCTATTCTTTCAGAGGGGAGGAGGT

IRN21|AB863156.1| CAACAAATAA---------------------AATTGCTATTCTTTCAGAGGGGAGGAGGT

JPNHGB340|AB863157.1| CTACAAACAAGATAGAAAATCCACTAGAAGAAATTGCTATTCTTTCAGAGGGGAGGAGGT

JPNKWB778|AB863158.1| CTACAAACAAGATAGAAAATCCACTAGAAGAAATTGCTATTCTTTCAGAGGGGAGGAGGT

JPNM|AB863159.1| CTACAAACAAGATAGAAAATCCACTAGAAGAAATTGCTATTCTTTCAGAGGGGAGGAGGT

JPNN|AB863160.1| CTACGAATAAGATAGAAAATCCACTAGAAGAAATTGCTATTCTTTCAGAGGGGAGGAGGT

JPNS1|AB863161.1| CTACGAACAAGATAGAAAATCCACTAGAAGAAATTGCTATTCTTTCAGAGGGGAGGAGGT

JPNS2|AB863162.1| CTACGAACAAGATAGAAAATCCACTAGAAGAAATTGCTATTCTTTCAGAGGGGAGGAGGT

JPNTKD762|AB863163.1| CTACAAACAAGATAGAAAATCCACTAGAAGAAATTGCTATTCTTTCAGAGGGGAGGAGGT

JPNUV1|AB863164.1| CTACAAACAAGATAGAAAATCCACTAGAAGAAATTGCTATTCTTTCAGAGGGGAGGAGGT

JPNUV26|AB863165.1| CTACAAACAAGATAGAAAATCCACTAGAAGAAATTGCTATTCTTTCAGAGGGGAGGAGGT

TUR1|AB863166.1| CAACAAATAA---------------------AATTGCTGTTCTTTCAGAGGGGAGGAGGT

TUR2|AB863167.1| CAACAAATAA---------------------AATTGCTATTCTTTCAGAGGGGAGGAGGT

TUR4|AB863168.1| CAACAAATAA---------------------AATTGCTATTCTTTCAGAGGGGAGGAGGT

TUR5|AB863169.1| CAACAAATAA---------------------AATCGCTATTCTTTCAGAGGGGAGGAGGT

TUR12|AB863170.1| CAACAAATAA---------------------AATTGCTATTCTTTCAGAGGGGAGGAGGT

TUR34|AB863171.1| CAACAAATAA---------------------AATTGCTATTCTTTCAGAGGGGAGGAGGT

TUR50|AB863172.1| CAACAAATAA---------------------AATTGCTATTCTTTCAGAGGGGAGGAGGT

TUR59|AB863173.1| CAACAAATAA---------------------AATTGCTGTTCTTTCAGAGGGGAGGAGGT

TUR69|AB863174.1| CAACAAATAA---------------------AATTGCTGTTCTTTCAGAGGGGAGGAGGT

TUR81|AB863175.1| CAACAAATAA---------------------AATTGCTGTTCTTTCAGAGGGGAGGAGGT

TUR84|AB863176.1| CAACAAATAA---------------------AATTGCTGTTCTTTCAGAGGGGAGGAGGT

TUR94|AB863177.1| CAACAAATAA---------------------AATTGCTGTTCTTTCAGAGGGGAGGAGGT

TUR213|AB863178.1| CAACAAATAA---------------------AATTGCTGTTCTTTCAGAGGGGAGGAGGT

TUR214|AB863179.1| CAACAAATAA---------------------AATTGCTGTTCTTTCAGAGGGGAGGAGGT

TUR216|AB863180.1| CAACAAATAA---------------------AATTGCTGTTCTTTCAGAGGGGAGGAGGT

TUR220|AB863181.1| CAACAAATAA---------------------AATTGCTGTTCTTTCAGAGGGGAGGAGGT

TUR239|AB863182.1| CAACAAATAA---------------------AATTGCTATTCTTTCAGAGGGGAGGAGGT

TUR244|AB863183.1| CAACAAATAA---------------------AATTGCTGTTCTTTCAGAGGGGAGGAGAC

TUR249|AB863184.1| CAACAAATAA---------------------AATTGCTGTTCTTTCAGAGGGGAGGAGGT

TUR263|AB863185.1| CAACAAATAA---------------------AATTGCTGTTCTTTCAGAGGGGAGGAAGT

TUR278|AB863186.1| CAACAAATAA---------------------AATTGCTATTCTTTCAGAGGGGAGGAGGT

TUR279|AB863187.1| CAACAAATAA---------------------AATTGCTGTTCTTTCAGAGGGGAGGAGGT

TUR285|AB863188.1| CAACAAATAA---------------------AATTGCTATTCTTTCAGAGGGGAGGAGGT

TUR289|AB863189.1| CAACAAATAA---------------------AATTGCTGTTCTTTCAGAGGGGAGGAGGT

TUR303|AB863190.1| CAACAAATAA---------------------AATTGCTGTTCTTTCAGAGGGGAGGAGGT

TUR306|AB863191.1| CAACAAATAA---------------------AATTGCTGTTCTTTCAGAGGGGAGGAGGT

Cabb-S|NC_001497.1| TATCAGAAGAAAAACTCTTTATCACTCAACAAAGAATGCAAAAAATCGAAGAACTACTTG

CRO180A|AB863192.1| TATCAGAAGAAAAACTTTTCATCACTCAGCAAAGAATGCAAAAAATCGAAGAACTACTTG

GRC83|AB863193.1| TATCAGAAGAAAAACTTTTCATCACTCAGCAAAGAATGCAAAAAATCGAAGAACTACTTG

GRC84B|AB863194.1| TATCAGAAGAAAAACTTTTCATCACTCAGCAAAGAATGCAAAAAATCGAAGAACTACTTG

GRC86B|AB863195.1| TATCAGAAGAAAAACTTTTCATCACTCAGCAAAGAATGCAAAAAATCGAAGAACTACTTG

GRC86D|AB863196.1| TATCAGAAGAAAAACTTTTCATCACTCAGCAAAGAATGCAAAAAATCGAAGAACTACTTG

GRC87E|AB863197.1| TATCAGAAGAAAAACTTTTCATCACTCAGCAAAGAATGCAAAAAATTGAAGAACTACTTG

GRC87G|AB863198.1| TATCAGAAGAAAAACTTTTCATCACTCAGCAAAGAATGCAAAAAATCGAAGAACTACTTG

GRC91B|AB863199.1| TATCAGAAGAAAAACTTTTCATCACTCAGCAAAGAATGCAAAAAATCGAAGAACTACTTG

GRC92A|AB863200.1| TATCAGAAGAAAAACTTTTCATCACTCAGCAAAGAATGCAAAAAATCGAAGAACTACTTG

GRC92C|AB863201.1| TATCAGAAGAAAAACTTTTCATCACTCAGCAAAGAATGCAAAAAATCGAAGAACTACTTG

GRC92D|AB863202.1| TATCAGAAGAAAAACTTTTCATCACTCAGCAAAGAATGCAAAAAATCGAAGAACTGCTTG

IRN1|AB863136.1| TATCAGAAGAAAAACTTTTCATCACTCAACAAAGAATGCAAAAAATCGAAGAACTACTTG

IRN2|AB863137.1| TATCAGAAGAAAAACTTTTCATCACTCAGCAAAGAATGCAAAAAATCGAAGAACTACTTG

IRN3|AB863138.1| TATCAGAAGAAAAACTTTTCATCACTCAACAAAGAATGCAAAAAATCGAAGAACTACTTG

IRN4|AB863139.1| TATCAGAAGAAAAACTTTTCATCACTCAACAAAGAATGCAAAAAATCGAAGAACTACTTG

IRN5|AB863140.1| TATCAGAAGAAAAACTTTTCATCACTCAACAAAGAATGCAAAAAATCGAAGAACTACTTG

IRN6|AB863141.1| TATCAGAAGAAAAACTTTTCATCACTCAACAAAGAATGCAAAAAATCGAAGAACTACTTG

IRN7|AB863142.1| TATCAGAAGAAAAACTTTTCATCACTCAGCAAAGAATGCAAAAAATCGAAGAACTACTTG

IRN8|AB863143.1| TATCAGAAGAAAAACTTTTCATCACTCAACAAAGAATGCAAAAAATCGAAGAACTACTTG

IRN9|AB863144.1| TATCAGAAGAAAAACTTTTCATCACTCAGCAAAGAATGCAAAAAATCGAAGAACTACTTG

IRN10|AB863145.1| TATCAGAAGAAAAACTTTTCATCACTCAGCAAAGAATGCAAAAAATCGAAGAACTACTTG

IRN11|AB863146.1| TATCAGAAGAAAAACTTTTCATCACTCAACAAAGAATGCAAAAAATCGAAGAACTACTTG

IRN12|AB863147.1| TATCAGAAGAAAAACTTTTCATCACTCAGCAAAGAATGCAAAAAATCGAAGAACTACTTG

IRN13|AB863148.1| TATCAGAAGAAAAACTTTTCATCACTCAGCAAAGAATGCAAAAAATCGAAGAACTACTTG

IRN14|AB863149.1| TATCAGAAGAAAAACTTTTCATCACTCAGCAAAGAATGCAAAAAATCGAAGAACTACTCG

IRN15|AB863150.1| TATCAGAAGAAAAACTTTTCATCACTCAGCAAAGAATGCAAAAAATCGAAGAACTACTTG

IRN16|AB863151.1| TATCAGAAGAAAAACTTTTCATCACTCAGCAAAGAATGCAAAAAATCGAAGAACTACTTG

IRN17|AB863152.1| TATCAGAAGAAAAACTTTTCATCACTCAGCAAAGAATGCAAAAAATCGAAGAACTACTTG

IRN18|AB863153.1| TATCAGAAGAAAAACTTTTCATCACTCAGCAAAGAATGCAAAAAATCGAAGAACTACTTG

IRN19|AB863154.1| TATCAGAAGAAAAACTTTTCATCACTCAGCAAAGAATGCAAAAAATCGAAGAACTACTTG

IRN20|AB863155.1| TATCAGAAGAAAAACTTTTCATCACTCAGCAAAGAATGCAAAAAATCGAAGAACTACTTG

IRN21|AB863156.1| TATCAGAAGAAAAACTTTTCATCACTCAGCAAAGAATGCAAAAAATCGAAGAACTACTTG

JPNHGB340|AB863157.1| TATCAGAAGAAAAACTTTTCATCACTCAACAAAGAATGCAAAAAATCGAAGAACTACTTG

JPNKWB778|AB863158.1| TATCAGAAGAAAAACTTTTCATCACTCAACAAAGAATGCAAAAAATCGAAGAACTACTTG

JPNM|AB863159.1| TATCAGAAGAAAAACTTTTCATCACTCAACAAAGAATGCAAAAAATCGAAGAACTACTTG

JPNN|AB863160.1| TA---GAAGAAAAACTCTTCATCACTCAACAAAGAATGCAAAAAATCGAAGAACTACTTG

JPNS1|AB863161.1| TA---GAAGAAAAACTCTTCATCACTCAACAAAGAATGCAAAAAATCGAAGAACTACTTG

JPNS2|AB863162.1| TA---GAAGAAAAACTCTTCATCACTCAACAAAGAATGCAAAAAATCGAAGAACTACTTG

JPNTKD762|AB863163.1| TATCAGAAGAAAAACTTTTCATCACTCAACAAAGAATGCAAAAAATCGAAGAACTACTTG

JPNUV1|AB863164.1| TATCAGAAGAAAAACTTTTCATCACTCAACAAAGAATGCAAAAAATCGAAGAACTACTTG

JPNUV26|AB863165.1| TATCAGAAGAAAAACTTTTCATCACTCAACAAAGAATGCAAAAAATCGAAGAACTACTTG

TUR1|AB863166.1| TATCAGAAGAAAAACTTTTCATCACTCAGCAAAGAATGCAAAAAATCGAAGAACTACTCG

TUR2|AB863167.1| TATCAGAAGAAAAACTTTTCATCACTCAGCAAAGAATGCAAAAAATCGAAGAACTACTCG

TUR4|AB863168.1| TATCAGAAGAAAAACTTTTCATCACTCAGCAAAGAATGCAAAAAATCGAAGAACTACTCG

TUR5|AB863169.1| TATCAGAAGAAAAACTTTTCATCACTCAGCAAAGAATGCAAAAAATCGAAGAACTACTTG

TUR12|AB863170.1| TATCAGAAGAAAAACTTTTCATCACTCAGCAAAGAATGCAAAAAATCGAAGAACTACTTG

TUR34|AB863171.1| TATCAGAAGAAAAACTCTTCATCACTCAACAAAGAATGCAAAAAATCGAAGAACTACTCG

TUR50|AB863172.1| TATCAGAAGAAAAACTTTTCATCACTCAGCAAAGAATGCAAAAAATCGAAGAACTACTCG

TUR59|AB863173.1| TATCAGAAGAAAAACTTTTCATCACTCAGCAAAGAATGCAAAAAATTGAAGAACTACTCG

TUR69|AB863174.1| TATCAGAAGAAAAACTTTTCATCACTCAGCAAAGAATGCAAAAAATCGAAGAACTACTCG

TUR81|AB863175.1| TATCAGAAGAAAAACTTTTCATCACTCAGCAAAGAATGCAAAAGATTGAAGAACTACTCG

TUR84|AB863176.1| TATCAGAAGAAAAACTTTTCATCACTCAGCAAAGAATGCAAAAAATCGAAGAACTACTCG

TUR94|AB863177.1| TATCAGAAGAAAAACTTTTCATCACTCAACAAAGAATGCAAAAAATCGAAGAACTACTTG

TUR213|AB863178.1| TATCAGAAGAAAAACTTTTCATCACTCAGCAAAGAATGCAAAAAATCGAAGAACTACTCG

TUR214|AB863179.1| TATCAGAAGAAAAACTTTTCATCACTCAGCAAAGAATGCAAAAAATCGAAGAACTACTCG

TUR216|AB863180.1| TT---GGAGAAAAACTCTTCATCACTCAACAAAGAATGCAAAAAATCGAAGAACTACTTG

TUR220|AB863181.1| TATCAGAAGAAAAACTTTTCATCACTCAGCAAAGAATGCAAAAAATCGAAGAACTACTCG

TUR239|AB863182.1| TATCAGAAGAAAAACTTTTCATCACTCAGCAAAGAATGCAAAAAATCGAAGAACTACTTG

TUR244|AB863183.1| TATCAGAAGAAAAACTTTTCATCACTCAGCAAAGAATGCAAAAAATCGAAGAACTACTCG

TUR249|AB863184.1| TATCAGAAGAAAAACTTTTCATCACTCAGCAAAGAATGCAAAAAATCGAAGAACTACTCG

TUR263|AB863185.1| TATCAGAAGAAAAACTTTTCATCACTCAACAAAGAATGCAAAAAATCGAAGAACTACTTG

TUR278|AB863186.1| TATCAGAAGAAAAACTTTTCATCACTCAACAAAGAATGCAAAAAATCGAAGAACTACTCG

TUR279|AB863187.1| TATCAGAAGAAAAACTTTTCATCACTCAGCAAAGAATGCAAAAAATCGAAGAACTACTCG

TUR285|AB863188.1| TATCAGAAGAAAAACTTTTCATCACTCAGCAAAGAATGCAAAAAATCGAAGAACTACTTG

TUR289|AB863189.1| TATCAGAAGAAAAACTTTTCATCACTCAGCAAAGAATGCAAAAAATCGAAGAACTACTCG

TUR303|AB863190.1| TATCAGAAGAAAAACTTTTCATCACTCAGCAAAGAATGCAAAAAATCGAAGAACTACTCG

TUR306|AB863191.1| TATCAGAAGAAAAACTTTTCATCACTCAGCAAAGAATGCAAAAAATCGAAGAACTACTCG

Cabb-S|NC_001497.1| AGAAAGTATGTTCAGAAAATCCATTAGATCCTAACAAGACTAAGCAATGGATGAAAGCTT

CRO180A|AB863192.1| AGAAAGTATGTTCTGAAAATCCATTAGATCCTAACAAGACTAAGCAATGGATGAAAGCTT

GRC83|AB863193.1| AGAAAGTATGTTCAGAAAATCCATTAGATCCTAACAAGACTAAGCAATGGATGAAAGCTT

GRC84B|AB863194.1| AGAAAGTATGTTCAGAAAATCCATTAGATCCTAACAAGACTAAGCAATGGATGAAAGCTT

GRC86B|AB863195.1| AGAAAGTATGTTCAGAAAATCCATTAGATCCTAACAAGACTAAGCAATGGATGAAAGCTT

GRC86D|AB863196.1| AGAAAGTATGTTCAGAAAATCCATTAGATCCTAACAAGACTAAGCAATGGATGAAAGCTT

GRC87E|AB863197.1| AAAAAGTATGTTCAGAAAATCCATTAGATCCTAACAAGACTAAGCAATGGATGAAAGCTT
[truncated: 347,903 more chars]
